# The Influence of Branch Order on Optimal Leaf Vein Geometries: Murray’s Law and Area Preserving Branching

**DOI:** 10.1371/journal.pone.0085420

**Published:** 2013-12-31

**Authors:** Charles A. Price, Sarah-Jane C. Knox, Tim J. Brodribb

**Affiliations:** 1 School of Plant Biology, University of Western Australia, Perth, Western Australia, Australia; 2 School of Plant Science, University of Tasmania, Hobart, Tasmania, Australia; Centrum Wiskunde & Informatica (CWI) & Netherlands Institute for Systems Biology, Netherlands

## Abstract

Models that predict the form of hierarchical branching networks typically invoke optimization based on biomechanical similitude, the minimization of impedance to fluid flow, or construction costs. Unfortunately, due to the small size and high number of vein segments found in real biological networks, complete descriptions of networks needed to evaluate such models are rare. To help address this we report results from the analysis of the branching geometry of 349 leaf vein networks comprising over 1.5 million individual vein segments. In addition to measuring the diameters of individual veins before and after vein bifurcations, we also assign vein orders using the Horton-Strahler ordering algorithm adopted from the study of river networks. Our results demonstrate that across all leaves, both radius tapering and the ratio of daughter to parent branch areas for leaf veins are in strong agreement with the expectation from Murray’s law. However, as veins become larger, area ratios shift systematically toward values expected under area-preserving branching. Our work supports the idea that leaf vein networks differentiate roles of leaf support and hydraulic supply between hierarchical orders.

## Introduction

The transport of fluid in biological organisms from a single point source to distributed sinks via a hierarchical branching network is a recurrent pattern across multi-cellular clades [1]. In light of the ubiquity of this phenomenon, and its importance in influencing organism form and function, numerous authors have offered theoretical models to predict the form of network branching [2-7]. These approaches typically invoke optimization as the principle force shaping network evolution, an intuitively satisfying approach due to its obvious conceptual links with natural selection. In plants, branching networks serve both resource delivery and structural roles and consequently most models consider either the internal branching network (e.g. mammal veins) [8], or the external network (e.g. tree branches) [9], or both internal and external networks simultaneously [3,10]. 

 Among the more successful models of internal branching is that of animal physiologist C.D. Murray. In 1926, Murray considered the geometry of branching junctions in mammalian cardiovascular networks reasoning that millions of years of natural selection should have resulted in efficient vein networks [8,11]. Murray proposed two primary costs: i) the cost of building and maintaining the network, which for a unit length is proportional to the square of the radius, and ii) the cost of transporting fluid through the network, which for laminar flow in a cylindrical tube is given by Poiseuille’s law and is inversely proportional to radius to the fourth power. Within this framework Murray solved for the branching junction geometry that simultaneously minimizes both construction and resistance costs, with minimization of resistance equivalent to maximizing conductance (resistance=1/conductance). The resulting prediction is known as Murray’s law, and in its most commonly encountered form states that the sum of the radii cubed remains constant across branching generations; *r*_*k*_^3^=Σ *r*_*k+1*_^3^, where *r* is radius, and *k* and *k*+1 refer to the parent and daughter branches respectively. For a symmetrical bifurcating network, Murray’s law predicts the ratio of daughter to parent cross sectional areas will be 2*ak+1*/ *a*_*k*_ ≈ 2^1/3^ ≈ 1.25. Murray’s law assumes that vessels do not provide structural support in the form of resistance to tensile or compressive forces [12]. However, as noted by Sherman [11], for an idealized network in which transmural pressure is the only force acting on the vessel wall, the Young-Laplace law predicts that vessel wall radii should be linearly proportional to the radius of the vessels themselves, a prediction that holds equally for a single vessel or bundle of closely packed vessels.

Murray’s law has received considerable attention in mammal arterial systems, where it is generally supported in all but the largest vessels such as the aorta and subsequent vessels leaving the heart, which are closer to an area-preserving relationship, *r*_*k*_^2^=Σ *r*_*k+1*_^2^ [11,13]. Area-preserving branching can be explained by the need to match impedances resulting from wave reflections at junctions [2], or to maintain a constant flow velocity, which need not be mutually exclusive selection principles. Analyses of the external stem branching patterns suggest that tree branches largely follow area-preserving branching (a.k.a. DaVinci’s rule) [14], and it has recently been shown that the ratio of conducting to non-conducting area remains constant across branching orders in several tree species, thus internal and external branching exhibit a proportional scaling [10].

In plants, Murray’s law has received limited attention, having been evaluated in samples of plant internal conduit networks in trees [12], in the conduits in petiolules and petioles of compound leaves [15], and in 863 veins from within a small (1 cm^2^) subsection of a single sunflower leaf [16,17]. McCulloh and collaborators examined the dimensions of xylem in cross section. In contrast, Wang and Canny examined the dimensions of veins (vessel bundles). Nonetheless, both studies have found support for Murray’s law in leaves. Due to the time consuming nature of measuring xylem dimensions in cross section or with measuring large numbers of vein junctions, we have little sense of the robustness of these results across species, the variability in vein measures within and across leaves, and the extent to which internal (xylem conduit) or external (whole vein) branching dimensions depend on vein size and/or order. 

In mammals, structural demands are met by the skeletal system and vessel networks offer little if any structural support. In plants however, the vascular tissue is reinforced by high density compounds such as cellulose, hemi-cellulose and lignin to resist very large internal compression forces generated by xylem water tension [18]. These same compounds, in association with the plant resource delivery network, also provide the biomechanical support to resist both tensile and compressive forces produced by gravity [19]. McMahon and Kronauer [9] considered how the demands of self loading and lateral displacement might influence the scaling of limb dimensions in trees. Their resulting elastic similarity model has been thoroughly examined in trees, and while tree branches are subject to systematic variation in their biomechanical properties both within and across plants and species [19-22], elastic similarity remains a valuable point of departure for attempts to predict the scaling of plant form based on physical first principles [23]. For example, a well known fractal branching model invokes elastic similarity as one of its principle assumptions [2,3], and when combined with the assumption that networks are “volume filling”, such networks should also exhibit an area-preserving branching architecture [3], 2*ak+1*/ *a*_*k*_ ≈ 1. Note that volume filling as defined by West et al [3], means that *N*_*k*_ *l*_*k*_^3^ = *N*_*k+1*_ *l*_*k+1*_^3^, where *N*_*k*_ and *N*_*k+1*_ are the number of branches or conduits in each level and *l*_*k*_ and *l*_*k+1*_ are the lengths of branches or conduits in that level. Thus, when vein networks serve little to no biomechanical support role, Murray’s law might be expected to hold, whereas if there is a need to maintain a constant flow velocity, or veins are elastically similar *and* “volume-filling”, area-preserving branching might be observed. 

**Figure 1:**
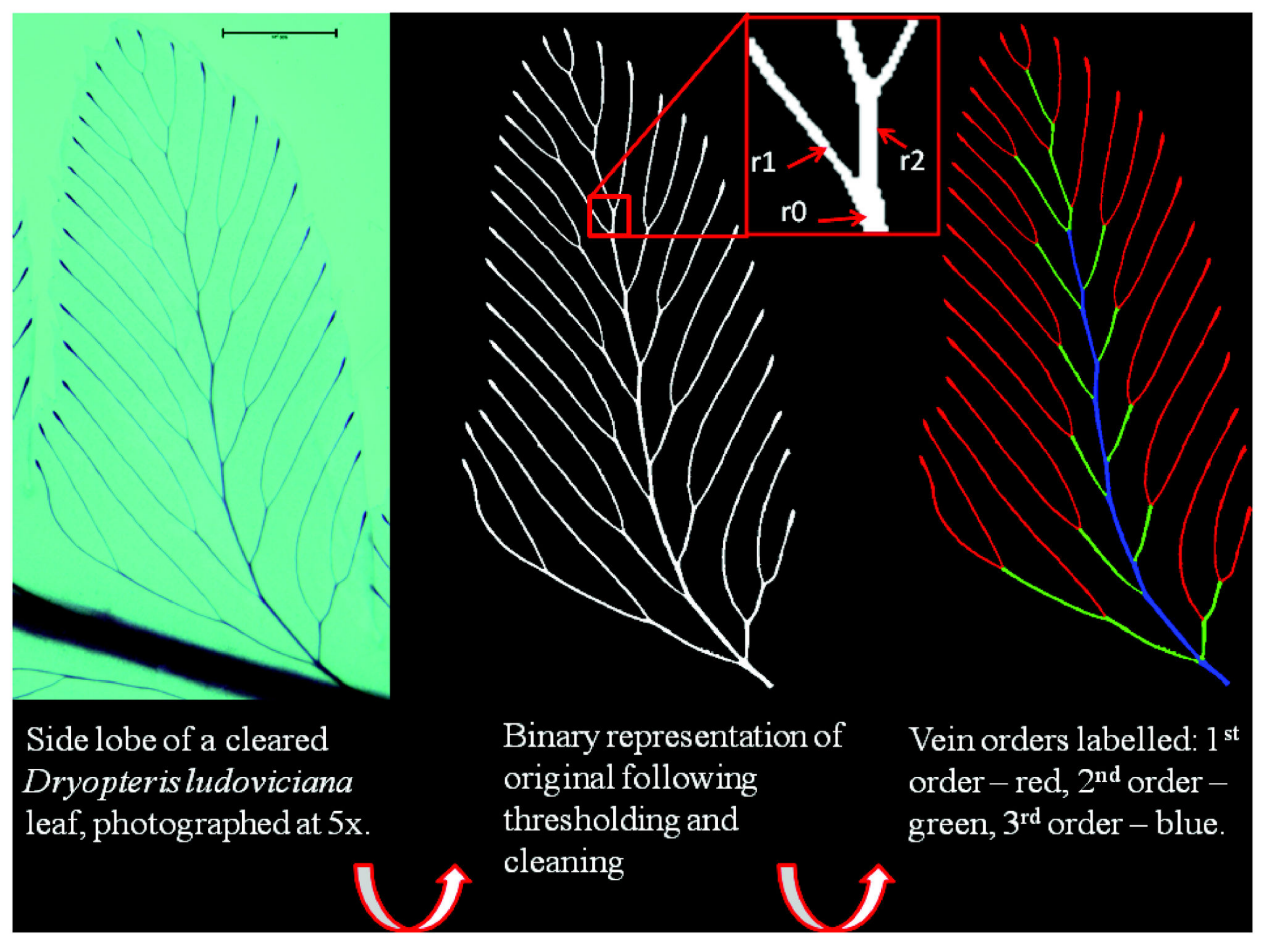
Image series illustrating some of the steps involved in going from an image of a cleared leaf (left panel), to a binary representation (middle panel), to one in which vein orders have been assigned (right panel). The central inset shows a vein bifurcation point; The radius of the parent branch (r0) and daughter branches (r1 and r2) at all branching points are determined, and used to determine cross sectional area (e.g. *A* = *πr*_*0*_^2^), and to solve for α in the equation r_0_^α^=r_1_^α^ + r_2_^α^ (see Materials and Methods).

With individual xylem cells reaching astronomical numbers in trees, complete descriptions of geometry and topology of complex plant conduit networks are exceedingly difficult to obtain. Most current approaches rely on sub-sampling portions of networks [10,15]. Here we take advantage of the high visibility of leaf veins in cleared leaves to consider the contrasting predictions of both Murray’s law and area-preserving branching for the dimensions of vein networks in leaves. Leaf veins serve the multiple demands of delivering water and nutrients through xylem to mesophyll, carrying photosynthetic products through phloem to the rest of the plant, and providing structural support, therefore it is unclear if models based on single optimization criteria can capture the full complexity of leaf vein architecture. Ideally, measuring the dimensions of both conducting and non-conducting portions of leaf veins across branching orders within a leaf, and across numerous species, would provide the strongest test of the aforementioned models. Unfortunately current imaging technology does not allow visualization of entire vein networks in cross-section, and compiling such a library of images manually would be prohibitively time consuming. Recent developments do however allow the vein network to be digitized in paradermal view (parallel to the epidermis, Figure 1), and this perspective allows detailed examination of branching topology and geometry of whole leaf veins, a subject that has garnered increased attention [24-28].

To evaluate the aforementioned model predictions, we quantified the dimensions of veins in 349 leaves, representing 349 species, constituting over 1.5 million individual vein segments in total. Focusing on veins in images of cleared leaves, allows us to generate a large volume of data quickly and the results using this approach can both inform and constrain models that attempt to capture the full complexity of leaf vein architecture.

We examined the relationships among and between the following five properties of leaf vein networks: i) Radius taper exponent (α) – which describes the tapering of vein radii via the solution to the equation: r_0_^α^=r_1_^α^ + r_2_^α^ , where *r*_*0*_ refers to the radii of the parent branch, and *r*_*1*_and *r*_*2*_ refer to the radii of the daughter branches; ii) Area ratio - the sum of daughter branch areas over the parent branch area; iii) Asymmetry – the ratio of the smaller daughter branch to the larger daughter branch; iv) The size of parent branches; v) Branch order following a Horton-Strahler ordering scheme (where the smallest branch is defined as order 1). These five metrics allow us to evaluate model predictions, and to explore how vein size, order, and branching symmetry influence branching geometry.

## Results

### Tapering of vein radii is consistent with Murray's law

**Figure 2:**
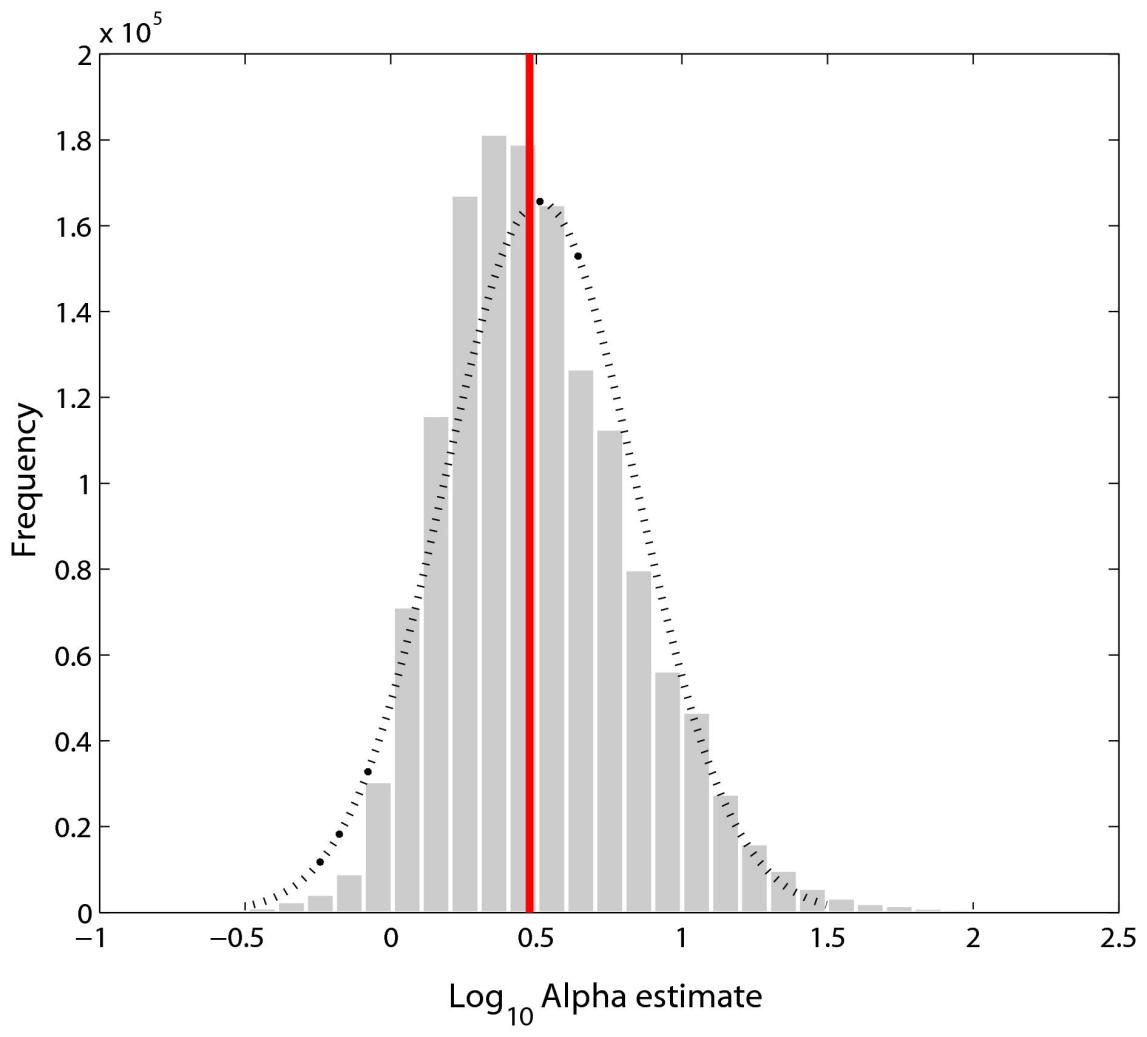
Frequency distribution for the value of α approximated by solving r_0_^α^=r_1_^α^ + r_2_^α^, for α (see Methods) for 1,514,771 individual vein junctions across all 349 leaves. Note that while the distribution fails a normality test, it is well approximated by a normal curve (hashed line) and strongly overlaps the expectation from Murray’s law, log_10_ (3) = 0.49 (red vertical line), with a mean value of 0.52. Thus, the distribution of estimated α values is strongly consistent with the expectation for Murray’s law.

The distribution of α values across all vein junctions and all leaves has a strong right skew, and while failing a test for normality (the Kolmogorov-Smirnov test is notoriously sensitive), visual inspection of Figure 2 suggests the distribution is reasonably well approximated by a normal curve in logarithmic space (base 10). The median non-transformed value for α is 2.96 (Murray’s law predicts 3), and the mean in log-space is 0.52 (Murray’s law predicts log_10_ (3) ≈ 0.48), thus the distribution of values of α, and thus the ratio of vein radii are generally consistent with Murray’s law. This distribution is heavily weighted by the numerical dominance of smaller vein orders. 

### Tapering of vein cross sectional areas is consistent with Murray's law

**Figure 3:**
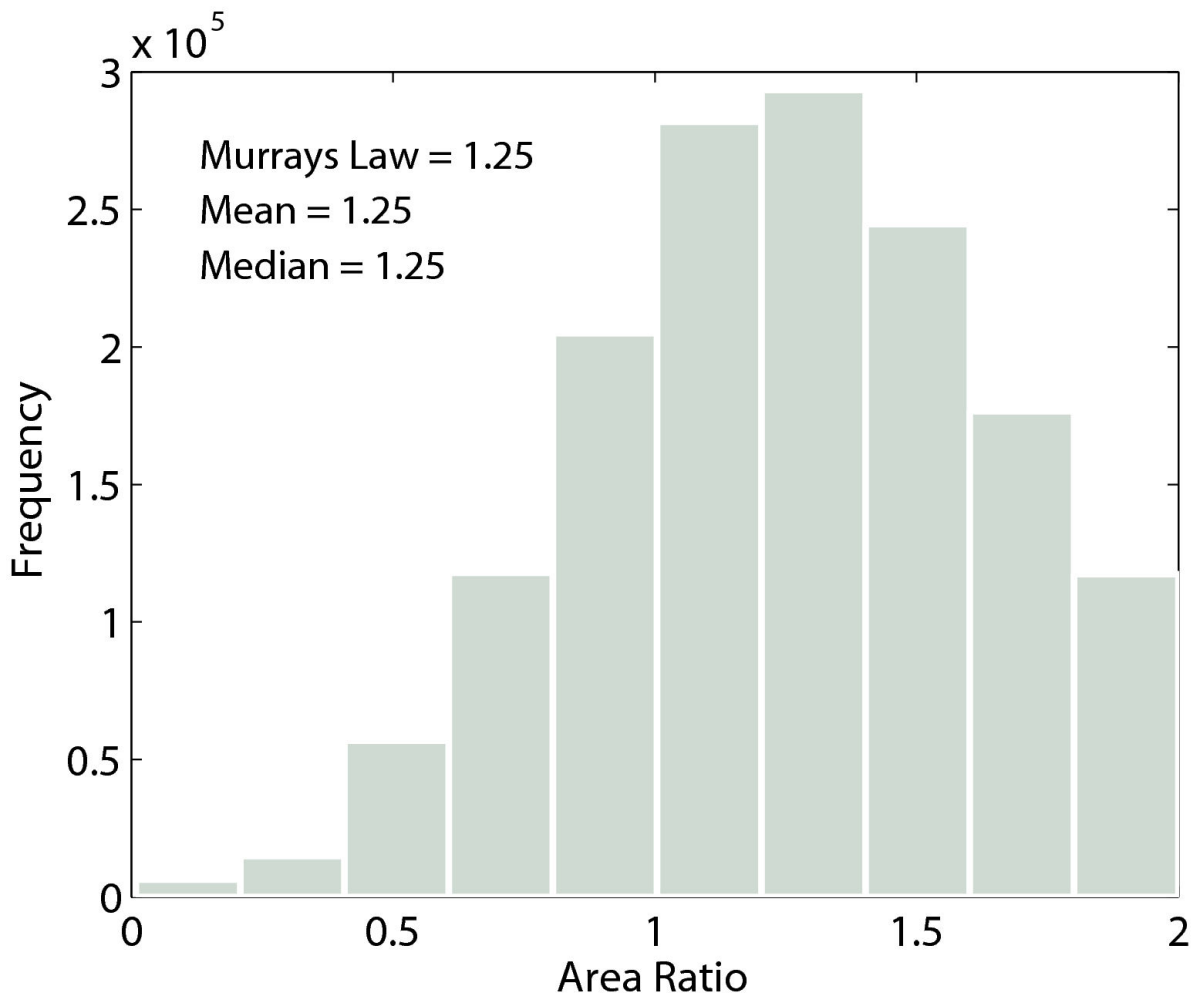
Frequency distribution of the area ratio for 1,514,771 individual vein junctions across all leaves. The mean and median are equal to the expectation for Murray’s law when daughter branches are symmetric. The distribution spans the range of expected values from 0 to 2, which includes area-preserving branching as well.

The mean area ratio across all branching junctions and across all leaves was 1.25 (Figure 3). The distribution of area ratios ranges from 0 to 2, its defined bounds. Individual leaf level mean area ratios ranged from 0.99 to 1.58, with a mean of 1.26 and a median of 1.23. 

### Parent-daughter area ratios change with order

As branch order increases (equivalent to decreasing “vein order”, according to the traditional nomenclature of leaf venation), the ratio of the total daughter branch area to the parent branch area decreases. The distributions for the smaller veins overlap more strongly with the expectation for Murray’s law, while the larger, higher order branches overlap the expectation for area-preserving branching (Figure 4, Figures S1-S349 in File S1).

**Figure 4:**
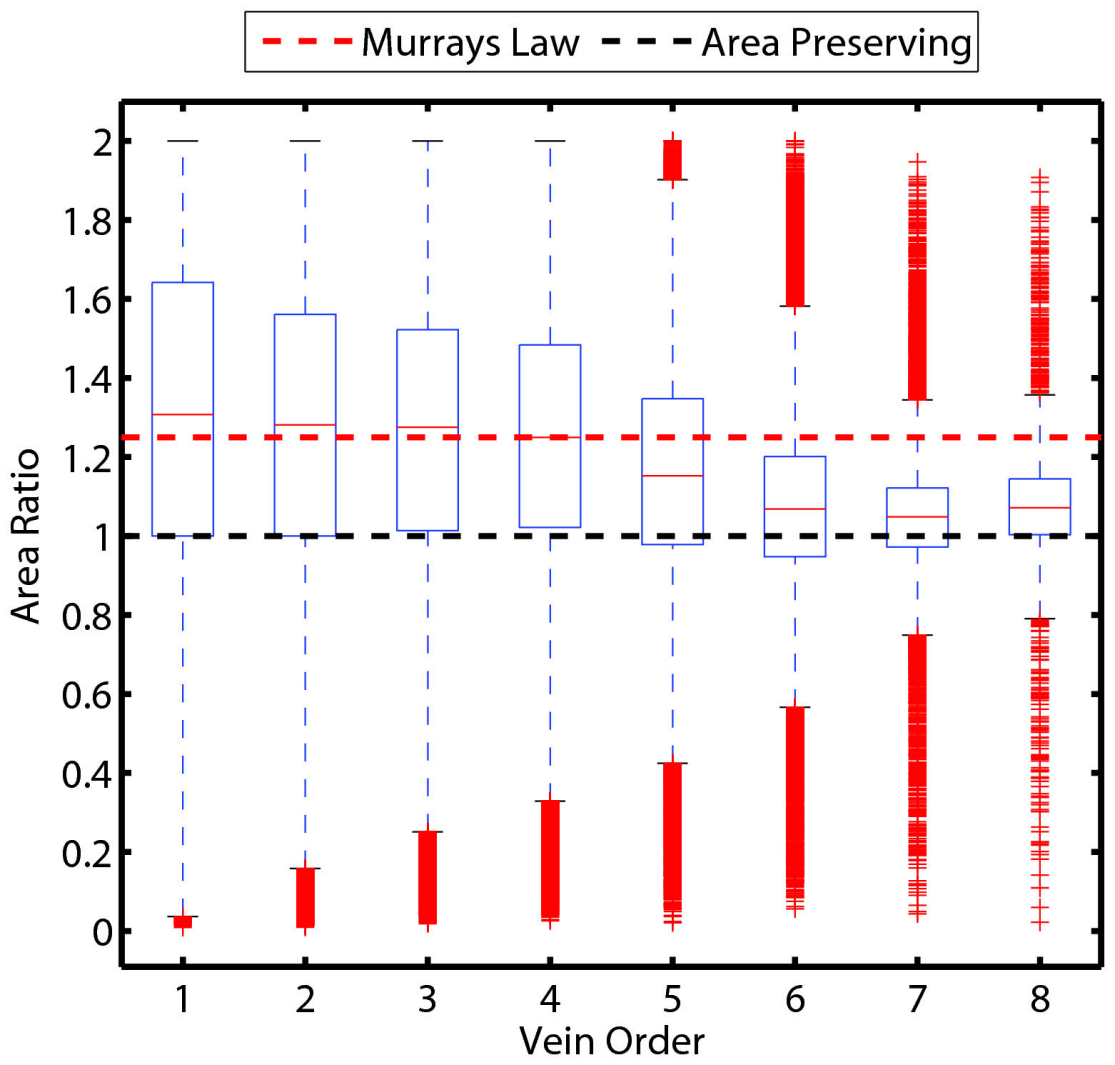
Box and whisker plot of the distribution of area ratios as a function of node order. Within each box, the central red mark is the median value, the box edges represent the 25th and 75th percentiles, the whiskers extend to the most extreme data points not considered outliers, and outliers (red plus symbols) are plotted individually. Outliers are considered those values which are larger than P_75_ + 1.5(P_75_-P_25_) or smaller than P_25_ - 1.5(P_75_-P_25_), where P_75_ and P_25_ are the 75^th^ and 25^th^ percentiles, respectively [53]. We classify data points as outliers only for the purposes of visualization; no data points were removed from our analyses. The red and black dashed lines are the expectations from Murray’s law and elastic similarity, respectively. Note that for vein orders 1-4 agreement with the expectation for Murray’s law is strong, but begins to depart as branch order increases, moving closer to the expectation for area-preserving branching. Note that in contrast to the convention used by leaf anatomists, here first order veins are the smallest, “terminal” veins in the network (see Methods).

### The area ratio distribution changes shape with order

**Figure 5:**
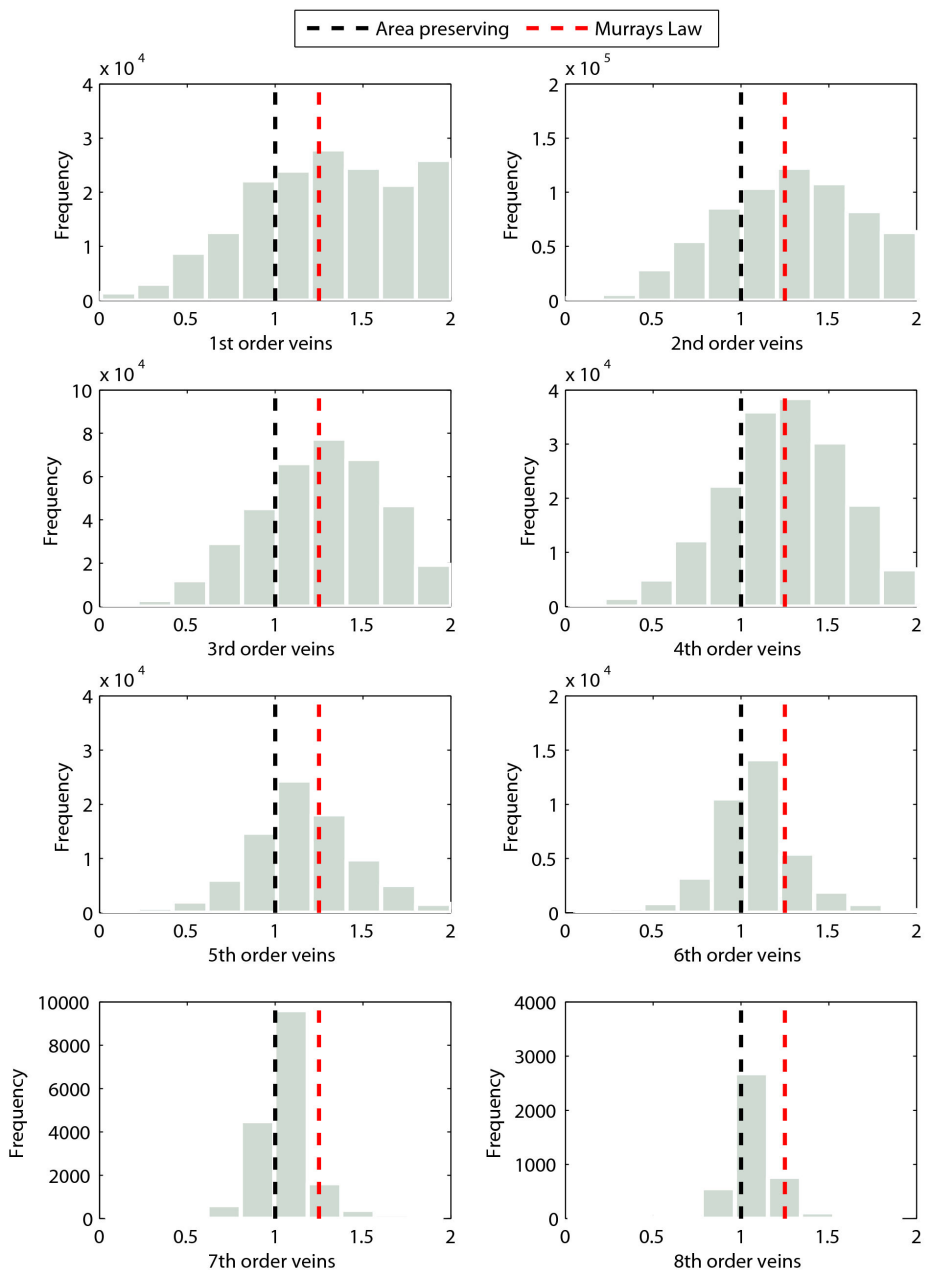
Frequency distributions for area ratios for each branch order 1-8. Note that as in Figure 4, the mode of distribution shifts from overlapping Murray’s law in the lowest order branches, to overlapping area preserving branching in the highest order branches. In addition, the shape of the distribution changes with increasing branch order, decreasing in variance and becoming increasingly leptokurtic (see Results).

As branch order increases, the distribution of area ratios within that order decreases in variance: 0.203, 0.162, 0.129, 0.115, 0.092, 0.060, 0.030, 0.028 (1^st^ through 8^th^ order respectively). Similarly the kurtosis of each distribution increases: 2.41, 2.51, 2.69, 2.90, 3.36, 4.61, 8.07, 9.94 (1^st^ through 8^th^ order respectively). Thus, along with a shift in the mean of the distribution, the overall shape of the distribution changes, becoming tighter (Figure 5).

### Branching is largely symmetric but asymmetry increases with size

**Figure 6:**
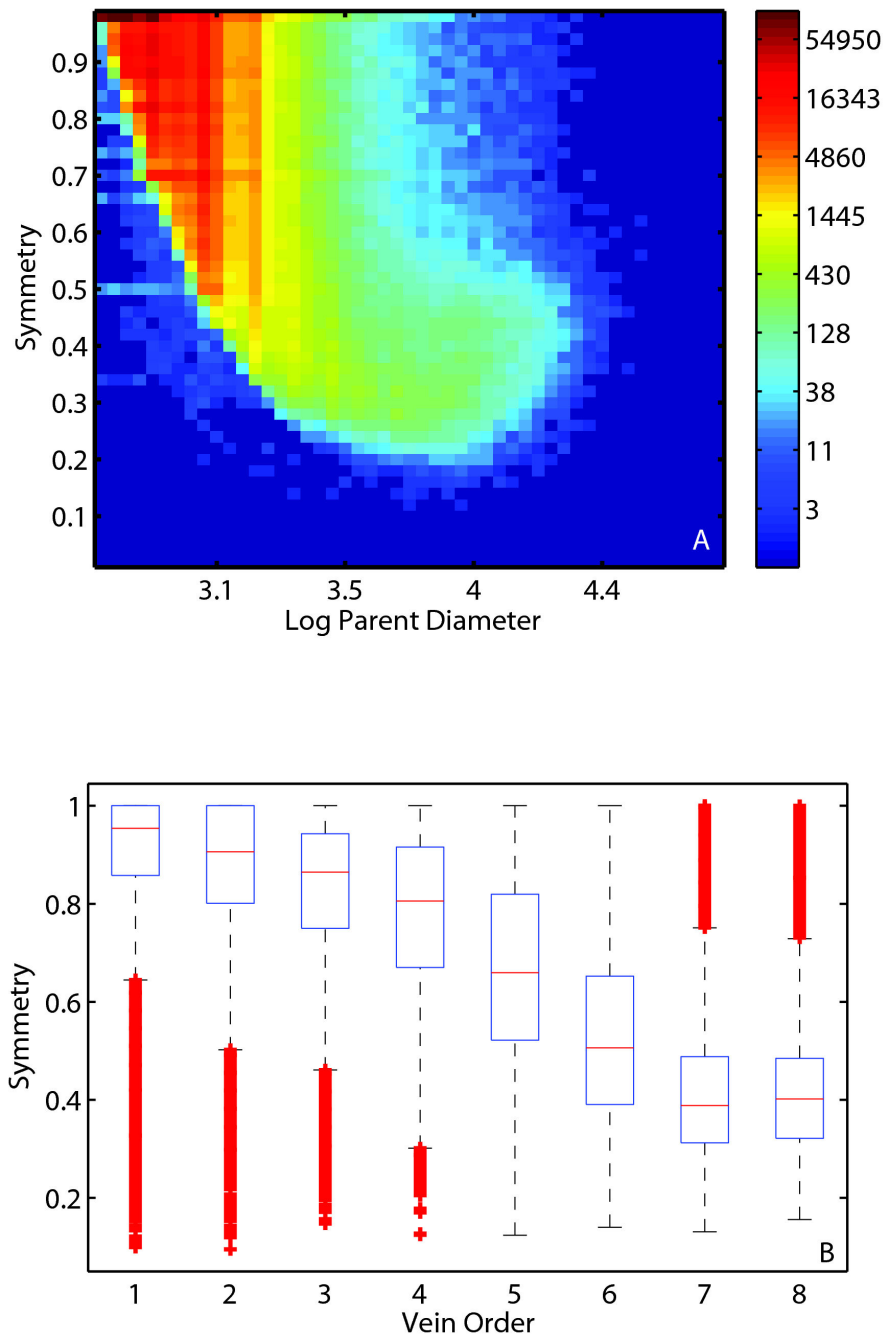
The influence of parent diameter and order on vein symmetry. Panel A: Heat map showing daughter branch symmetry, the ratio of the smaller daughter branch to the larger daughter branch, as a function of parent branch size. As parent branch diameter increases, the ratio of parent to daughter branch diameters becomes more asymmetric. Note that the abundance values for the 2D histogram are log transformed for image clarity, but the values on the key are not transformed. Panel B: Box and whisker plot of the decrease in symmetry with increasing vein orders. Within each box, the central red mark is the median value, the box edges represent the 25th and 75th percentiles, the whiskers extend to the most extreme data points not considered outliers, and outliers (red plus symbols) are plotted individually (see Fig. 4 caption for the definition of outliers). First order veins are largely symmetric, but symmetry decreases as vein order increases.

The frequency of measured symmetries is strongly left skewed being bounded by 1, with 18% of the measured values equaling one, and 90% greater than 0.8. Thus, most daughter branches in leaves are symmetric, or nearly so (Figure 6a). The degree of asymmetry between daughter branches increases with the size of the parent branch (Figure 6b). 

## Discussion

Across all leaves and all species, both the value of the radius tapering exponent (α) and the area ratio are in strong agreement with the predictions of Murray’s law for a symmetric network (Figures 2 and 3). To some extent, this is surprising because some of the assumptions underlying Murray’s law may not be supported in leaves. For example, Murray’s law assumes veins do not provide structural support and that fluid volume is not lost to transmural flow [11,29]. However, it is likely that most of the mechanical load in leaves is borne by the major veins (higher order under the Horton-Strahler scheme), with the numerically dominant minor veins providing little, if any structural support. Further, it has been shown that leaf vein networks are “leaky” with transmural loss particularly in the smaller veins [30]. Sensitivity analyses have shown that branching junctions that depart from the theoretical optimum in Murray’s law suffer small costs (~5%) in increased energy requirements. It may be that violating the model assumption of mass conservation across levels (through transmural loss) may have minor energetic costs relative to the theoretical optimum [31]. These caveats aside, our data indicate that leaves may be able to simultaneously satisfy competing optimization problems of support and water supply by producing a vein network that is hierarchically differentiated into regimes that satisfy primarily support and supply (larger veins), and supply functions (smaller veins) [32-34].

Leaf veins are comprised of vessel bundles which are a collection of different cell types including, xylem, phloem, and associated bundle sheath cells. The extent to which the dimensions of leaf veins can be used to evaluate optimization models for conduit geometry, such as Murray’s law, depends on the extent to which there is consistent proportionality between mean xylem dimensions and vessel bundle dimensions across branching orders. Unfortunately, due to the challenges associated with sectioning and imaging very small leaf veins, it is not currently known if such proportionality exists. In theory, the thickness of conduit wall required to resist collapse under capillary tension in the xylem is a linear function of its internal radius because the Laplace-Young law states that for a given transmural pressure, the force needed to counteract that pressure is proportional to the conduit radius [11,18]. Thus, under the Laplace constraint, the radius of the conduit wall will be in direct proportion to the radius of the conduit itself [18]. Further, recent work on tree branches has shown that the tapering of xylem that occurs from basal to distal branches is coincident with an increase in the number of individual xylem conduits such that the ratio of conducting to non-conducting area remains constant across branches of varying size [10]. This relationship emerges from a “packing rule” for xylem [35] where the number of xylem conduits (*N*) trades off with the mean diameter of conduits (d) such that *N* ≈ d^-2^, or with total vessel area (*A*), *N* ≈ A^-1^ [36]. Coomes et al. [37] report a consistent tapering of xylem across vein orders in several Oak species, but do not report xylem number or mean area as a function of vein order, so it is not clear if this tradeoff holds in leaf veins as well. Of course the larger veins in leaves clearly serve biomechanical support roles and are subject to both twisting and bending forces, thus the fraction of non-conducting tissue in vessel bundles devoted to support, and how that fraction varies with vein order and/or leaf size, is an area in need of further inquiry. 

Despite these caveats, a Murray’s law type scaling is strongly supported by our data, particularly in smaller vein orders in leaves. Noting that branching “order” used here starts with the smallest units called “first order”, (which is opposite to the botanical convention of defining the largest vein as the first order vein) we show that as vein order increases (veins get larger), the area ratio appears to depart systematically from Murray’s law and approaches that expected for area-preserving branching (Figure 4 and Figures S1-349 in File S1). This is consistent with the idea that larger veins play a greater role in supporting the leaf. In fact the proportional investment in larger veins (i.e. midrib) increases with leaf size in both simple and compound leaves [33]. In support of this idea, McCulloh et al. found greater agreement with Murray’s law in plants in which branches do not contribute much to structural support, such as vines, hydrostatically supported compound leaves [12], or small stem photosynthesizers [29]. The functional differentiation of major and minor veins suggested here from network scaling, are consistent with the developmental differentiation of vein orders, in particular the delayed intrusion of minor veins into the leaf mesophyll [34]. Despite the systematic departure from Murray’s law with increasing vein order, the global mean area ratio found in leaves here (Figure 3) is in strong agreement with Murray’s law due to the numerical dominance of the lowest order (minor) veins. 

In addition to a shift in the mean area ratio with changing vein order, we also observe a systematic shift in the shape of the distribution within each order. Figure 5 shows that the lowest order veins (minor veins), have wide distributions with a high variance, and that as vein order increases, the variance decreases with the distributions becoming more leptokurtic (see Results). This helps to explain why the higher order veins in Figure 4 have more outliers. The reasons for this systematic change are unknown. However, given the fourth power dependence of conductance on xylem radius, we speculate that there may be stronger pressure to keep area ratios close to an optimal value in larger veins. Future anatomical work exploring the ratio of conducting to non-conducting area, across vein orders in leaves can help to answer this and other questions.

As seen in Figure 6, as both the size and order of the parent vein increases, the asymmetry in daughter branches increases. Visual inspection of leaves and the decrease in symmetry with vein order both indicate that this pattern is largely driven by side branching found along the larger vein courses (higher order veins following the Strahler ordering algorithm). Despite the existence of asymmetry throughout, the overwhelming majority of vein bifurcations have daughter branch ratios that are close to symmetrical with over 70% of ratios being greater than 0.9 and over 90% greater than 0.8. Thus it is a small fraction of vein bifurcations that are strongly asymmetric, and thus comparing the area ratios we report to the expectation for Murray’s law under symmetric branching seems reasonable. Several authors have argued that the reticulate pattern found in leaf veins evolved to allow redundancy in flow paths [27,38,39], yet the side branching aspect of this pattern is rarely considered in theoretical approaches (but see ref. 40) [40]. 

 The transition from area-preserving to Murray’s law branching in leaf veins is superficially similar to that found in mammalian cardiovascular systems [13,41]. As the last common ancestor of plants and animals was unicellular, branching systems for each group arose independently, and may have arrived at comparable solutions for the problem of efficient resource distribution. Simultaneous measures of both internal and external network geometry across multiple vein orders may serve to confirm this result. However, the area-preserving nature of major artery branching in mammals is unlikely to represent an adaptive response to biomechanical demands as it is in the larger veins in the leaf vein network. Area preserving branching in arterial branching more likely reflects selection to preserve flow velocity or match impedances due to wave reflections resulting from pulsatile flow [2]. Recent work has demonstrated that entire leaf vein networks are not the type of volume filling fractals assumed in previous work [6], but rather exhibit an exponential distribution of vein lengths which is consistent with a characteristic length scale, such as that found in river networks, due to the numerical dominance of the lowest order veins [5]. It remains to be seen however, if certain higher order vein courses (major veins), such as the lateral veins emerging from the midrib, exhibit the type of self-similar “volume filling” [3,6], that would lead to the area-preserving branching we observe.

Our data demonstrate that networks of leaf veins and blood vessels exhibit similarity in scaling characteristics but presumably for different reasons. The leaf venation of woody plants achieves an optimal solution to the problems of tissue support and transport by scaling the large mass-bearing ranks of the venation network consistent with a biomechanical optimum, while the smaller veins, involved in distributing water as close as possible to the evaporation sinks [42], follow a Murray’s law pattern. This pattern was revealed by looking beyond the initial agreement with Murray’s law scaling in leaves, and emphasizes the importance of testing for systematic deviation from general scaling exponents within hierarchies of multipurpose biological networks.

## Materials and Methods

We analyzed 349 leaves in total. 339 of the leaves we used for our analysis come from the cleared leaf image collection at the Smithsonian Institution. The collection, including the images used in this study, are currently available via an online database of cleared leaf images (http://www.clearedleavesdb.org/). We went through the entire image collection, selecting only the best images for our analyses based on three criteria: 1) leaf networks were relatively free from tears or other damage; 2) image resolution was sufficient to resolve many or most of the minor veins, and; 3) the contrast between leaf veins, areoles and background were significant enough for the LEAF GUI network extraction algorithms to resolve their structure. To confirm that our results were not specific to this group of leaf images we have also analyzed a 10 additional cleared leaves collected locally in Western Australia (Methods and Figures S340-351 in File S1). 

To quantify the dimension of leaf networks we utilized the recently released software package, LEAF GUI [43]. LEAF GUI is designed specifically for the analysis of leaf vein images. Extensive descriptions of the underlying algorithms can be found in [43] and on (www.leafgui.org) with several worked examples in Price [44]. The LEAF GUI software returns a characterization of the leaf as a weighted graph comprised of nodes and edges, where an edge is defined as a vein segment, and nodes are defined as the intersection of two or more edges. In addition, LEAF GUI extracts metric and positional information for each edge, such that each edge has an associated vector of weights including length, width, surface area, or volume.

To determine daughter to parent branch ratios as defined above, for each node in the network, we identified the branch with the largest radius and assumed it was the parent branch. The remaining two branches are then assumed to be daughter branches. Determination of branch cross sectional areas is based on assuming a cylindrical shape and thus area is simply a function of branch radius. To compare to the predictions from Murray’s law and area-preserving branching, we summed the areas of the two daughter branches, and divided that number by the parent branch area. Because we have assumed the largest branch is the parent branch, the maximum possible value for the sum of the daughter branch areas is twice that of the parent, i.e. if all branches were of equal radius and cross sectional area. The minimum value for the area ratio approaches zero, thus by definition the area ratio is bounded by zero and two.

We define branching symmetry as the ratio of the smallest daughter branch radius to the largest daughter branch radius, thus in a perfectly symmetric branching network, symmetry would equal one. Asymmetry occurs when one daughter branch is significantly larger than the other, as might occur with side branches along leaf midribs.

### Murray’s Law and Area Increasing Branching

In Murray’s formulation, the total power (*P*_*t*_) to construct and maintain a conduit of unit length is given by, Pt=(8*η*/*πr* ^4^)+ *πmr* ^2^, where *r* is conduit radius, η is the dynamic viscosity of the fluid and *m* is the metabolic cost coefficient [11]. The first term reflects the resistance costs and the second term reflects construction costs and thus changes in radius affect total power in opposite ways. 

As mentioned, Murray’s derivation leads to the well known prediction that the sum of the conduit radii cubed remains constant, or r_0_^3^ = r_1_^3^ + r_2_^3^. Estimating the radius tapering exponent requires finding the value of α in the following equation, r_0_^α^ = r_1_^α^ + r_2_^α^, that minimizes the difference between the right and left hand sides of the equation, or equivalently r_0_^α^- r_1_^α^ - r_2_^α^ = 0. To determine this value we used the function *fsolve* in Matlab which searches algorithmically for the zero (or root) of a function near a constant value, which we chose as three. However, when the size of daughter branches approach that of the parent branch, the estimation of α diverges, leading to a strongly right skewed distribution of α values, which includes much higher values than expected [45]. Rather than invoke an arbitrary cutoff for values of α [15,45], we simply report the distribution of α values, and note that the median value of α, and the mean in logarithmic space, are consistent with theoretical expectations. For a bifurcating network in which daughter branches are symmetrical, this rule can also be expressed as the ratio of daughter (*k+1*) and parent (k) areas (*A*), 2*A*_k+1_/*A*_*k*_ ≈ 1.25.

### Elastic Similarity and Area-Preserving Branching

Following West et al. [3], for a hierarchically branching network with branches indexed as (*k*), one can derive relationships between the radius (r), length (l) and number (n) of branches. The ratio of daughter (*r*_*k+1*_) to parent (*r*_*k*_) branch radii can be expressed as a function of the number of daughter (*n*_*k+1*_) branches per parent (*n*_*k*_), the furcation number (*n* = *nk+1*/ *n*_*k*_), or *rk+1*/*r*_*k*_ = *n* ^-a/2^. If branching networks are optimally designed to resist buckling there exists some relationship between the length and radius, *l* ≈ *r* ^*σ*^. Combining these we have *a* = 2/3σ. For a network that is volume filling, specifically *N*_*k*_ *l*_*k*_^3^ = *N*_*k+1*_ *l*_*k+1*_^3^, and elastically similar, σ = 2/3, leading to *a* = 1 which is the condition for area-preserving branching, or *r*_*0*_^2^ = *r*_1_^2^ + *r*_2_^2^. Thus for a bifurcating network as is typically found in leaves (*n* = 2), 2*A*_k+1_/*A*_*k*_ ≈ 1.

### Maximum Spanning Tree and Horton-Strahler Ordering

All of the aforementioned theories have been developed for open networks such as trees, however, most broad leaf angiosperm leaf networks are reticulate, meaning they contain loops. Despite this, a hierarchical structure is clearly evident in most leaves, and leaf classification schemes utilize this hierarchy, relying on the concept of vein order [46]. To unambiguously assign orders, we first utilize a pruning technique based on a topological concept, the Maximal Spanning Tree (MST)[47]. The MST is then subjected to an ordering algorithm developed for the study of river networks [48,49]. The MST is the network structure that most closely resembles the original leaf network and connects all nodes (bifurcation points), but that is strictly hierarchical. The determination of the MST is equivalent to pruning veins computationally in such a way that the resulting network is both strictly hierarchical and has functional properties (such as hydraulic conductance) or material properties (such as total volume or vein length) that preserve network hierarchy, and are as close to the original hierarchical network as possible. To find the MST we employed Prim’s algorithm [47] on the largest connected component of the graph returned from the LEAF GUI software, and selected the node closest to the point of petiole attachment as the root node. As we use only the largest connected component in our analysis, i.e. the vein network connected to the major veins, the omission of some small disconnected regions of minor veins is unlikely to have a strong influence the summary statistics we measure here.

The MST is a strictly hierarchical network (i.e., with no loops) which connects all vertices while maximizing some objective function. We maximized our trees for theoretical conductivity, which is proportional to *r* ^4^, as this was found to return network hierarchy with the greatest fidelity. This approach is robust to different exponent values such as *r* ^3^, or *r* ^5^, so long as there is a greater weighting on veins with larger radii. Thus, the MST is that which connects all of these nodes without forming loops, thereby preserving vein hierarchy and ensuring supply to mesophyll without being redundant.

 Once the MST is determined, we assigned orders to all branching levels using the Horton-Strahler ordering scheme originally developed for the study of river networks [48,49]. Both centripetal and centrifugal ordering schemes have been developed for the study of dendritic networks [50,51]. Centripetal schemes, such as Horton-Strahler ordering, start at the tips and number progressively down the tree. In contrast, centrifugal approaches start at the “trunk” (midrib/petiole) and increase in order towards the periphery. A goal of ordering algorithms generally is to classify branches into orders based on their functional similarities [50,51], and with respect to leaves both approaches have advantages and disadvantages. One might be interested in petioles as the functionally similar unit across leaves and employ a centrifugal scheme. This has the disadvantage however of assigning different orders to the terminal (minor) veins in a leaf. In a large leaf, this could mean that functionally equivalent minor veins could differ by many orders. We chose to view the terminal veins as the functionally equivalent units, i.e. the minor most veins are all 1^st^ order, based on the idea that the physical properties of the minor veins are more or less functionally equivalent within a species, and similar across species [5,42]. While the petioles of leaves are functionally equivalent in terms of being the point of attachment to the leaf, the biomechanical and hydrodynamic properties of petioles will change as a function of their size (and order) across leaves within a single species, or across different species.

Under the Horton-Strahler scheme, if a branch is terminal (has no daughters), its Strahler number is one. If a branch has one daughter with Strahler number *i*, and all other daughters have Strahler numbers less than *i*, then the Strahler number of the branch is *i* again. If a branch has two or more daughters with Strahler number *i*, and no daughters with greater number, then the Strahler number of the node is *i* +1. In this way, we assigned order to all branches within the MST. Note that this scheme is in contrast to the methodology currently used by leaf morphologists who typically refer to the leaf midrib as the primary vein, large vein courses emerging from the primary as secondary, etc. [46,52].

## Supporting Information

File S1 **Supporting methods.** Figures S1-S339, Area ratio as a function of vein order for each of the 339 leaves from the Smithsonian leaf collection analysed in this study. Figure S340, Frequency distribution of the area ratio for 191,769 individual vein junctions across the additional 10 leaves that analysed as described in the “10 Leaves Results” above. The mean and median are nearly equal to the expectation for Murray’s law when daughter branches are symmetric. Figures S341-S351, Area ratio as a function of vein order for each of the 10 additional leaves as described in the Supplemental Material. The box and whisker plots for these 10 leaves are entirely consistent with those from the Smithsonian leaf collection (Figures S1-S339). Lower order veins overlap more strongly with the expectation from Murray’s law and higher order veins overlap more strongly with the expectation for area preserving branching or elastic similarity.
(PDF)
