# Supplementary material for: The Influence of Branch Order on Optimal Leaf Vein Geometries: Murray’s Law and Area Preserving Branching
Source: PLoS One. 2013 Dec 31;8(12):e85420. doi: 10.1371/journal.pone.0085420 (PMC3877374; doi:10.1371/journal.pone.0085420)
Supplement: File S1 — Supporting methods. Figures S1-S339, Area ratio as a function of vein order for each of the 339 leaves from the Smithsonian leaf collection analysed in this study. Figure S340, Frequency distribution of the area ratio for 191,769 individual vein junctions across the additional 10 leaves that analysed as described in the “10 Leaves Results” above. The mean and median are nearly equal to the expectation for Murray’s law when daughter branches are symmetric. Figures S341-S351, Area ratio as a function of vein order for each of the 10 additional leaves as described in the Supplemental Material. The box and whisker plots for these 10 leaves are entirely consistent with those from the Smithsonian leaf collection (Figures S1-S339). Lower order veins overlap more strongly with the expectation from Murray’s law and higher order veins overlap more strongly with the expectation for area preserving branching or elastic similarity. (PDF) [file pone.0085420.s001.pdf]

Supporting Information

Methods: Cleared Leaves Analyses

Previous work using this same collection of leaf images has shown that their measured vein densities (vein length/leaf area) overlap, but are slightly lower than those reported previously using magnified images, and thus some leaves may under-represent the minor most veins. To validate that our results are not dependent on the use of these images, we cleared and scanned at high resolution, vein networks from an additional 10 leaves from the following species: *Scaevola crassifolia* Labill., *Banksia arborea* (C.A.Gardner) A.R.Mast & K.R.Thiele, *Banksia quercifolia* R.Br., *Dampiera teres* Lindl., *Azalea sp.*, *Eremophila denticulata* F.Muell., *Diplolaena grandiflora* Desf., *Grevillea pimeleoides* W.Fitzg. All leaves were collected locally in the Perth region, Western Australia, and in all leaves the minor most veins are clearly visible. As the leaf network images for these leaves were obtained using different methods than the Smithsonian collection, and serve only a confirmatory role in our analysis, we have analysed and presented these results here.

The 10 leaves constitute an additional 191,769 individual vein measurements. As seen in Fig. S340, the distribution of area ratios is striking similar to that found in Fig. 1 in the main manuscript with a mean of 1.25 and a median of 1.26. Moreover, boxplots of the area ratios as a function of vein order (Figs. S341-S351) reveal patterns fully consistent those found in the 339 leaves from the Smithsonian collection. Namely, the frequency distribution for orders 1-4 tend to overlap more strongly with the expectation from Murray’s law, while the higher order veins shift toward area-preserving branching.

Figures S1-S339: Area ratio as a function of vein order for each of the 339 leaves from the Smithsonian leaf collection analysed in this study.

Figure S340: Frequency distribution of the area ratio for 191,769 individual vein junctions across the additional 10 leaves that analysed as described in the “10 Leaves Results” above. The mean and median are nearly equal to the expectation for Murray’s law when daughter branches are symmetric.

Figures S341-S351: Area ratio as a function of vein order for each of the 10 additional leaves as described in the Supplemental Material. The box and whisker plots for these 10 leaves are entirely consistent with those from the Smithsonian leaf collection (Figs. S1-S339). Lower order veins overlap more strongly with the expectation from Murray’s law and higher order veins overlap more strongly with the expectation for area preserving branching or elastic similarity.

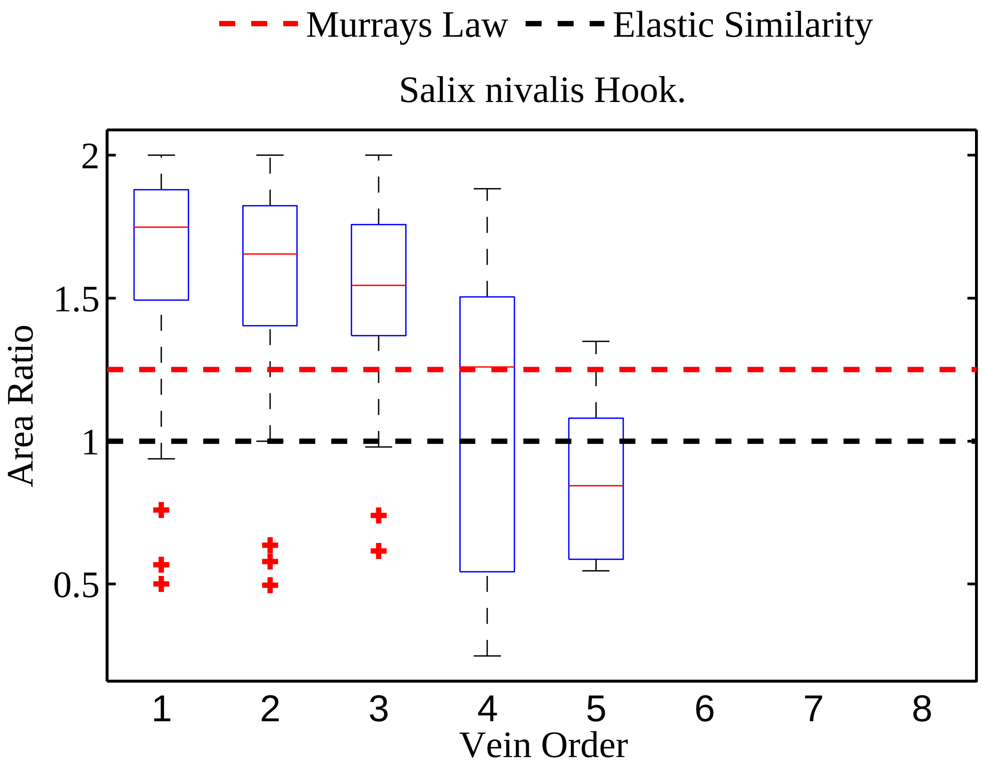
Murrays Law
Elastic Similarity
Salix nivalis Hook.
Area Ratio
2
1.5
1
0.5
1
2
3
4
5
6
7
8
Vein Order

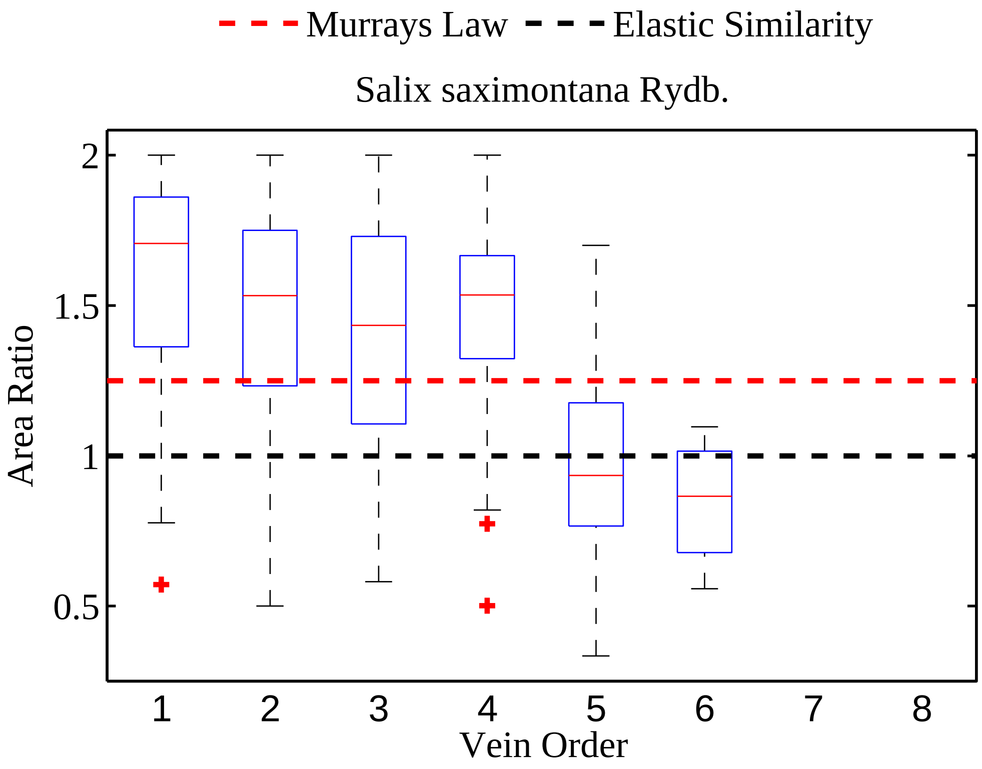

Murrays Law
Elastic Similarity
Salix saximontana Rydb.
Area Ratio
2
1.5
1
0.5
1
2
3
4
5
6
7
8
Vein Order

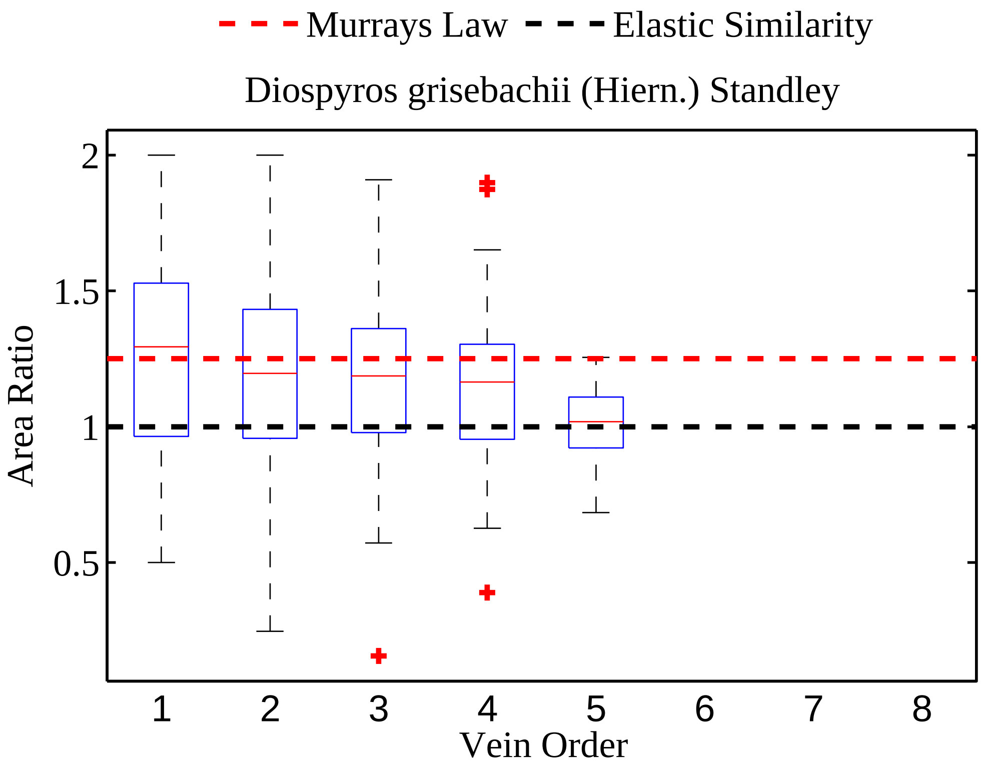
Murrays Law
Elastic Similarity
Diospyros grisebachii (Hiern.) Standley
Area Ratio
2
1.5
1
0.5
1
2
3
4
5
6
7
8
Vein Order

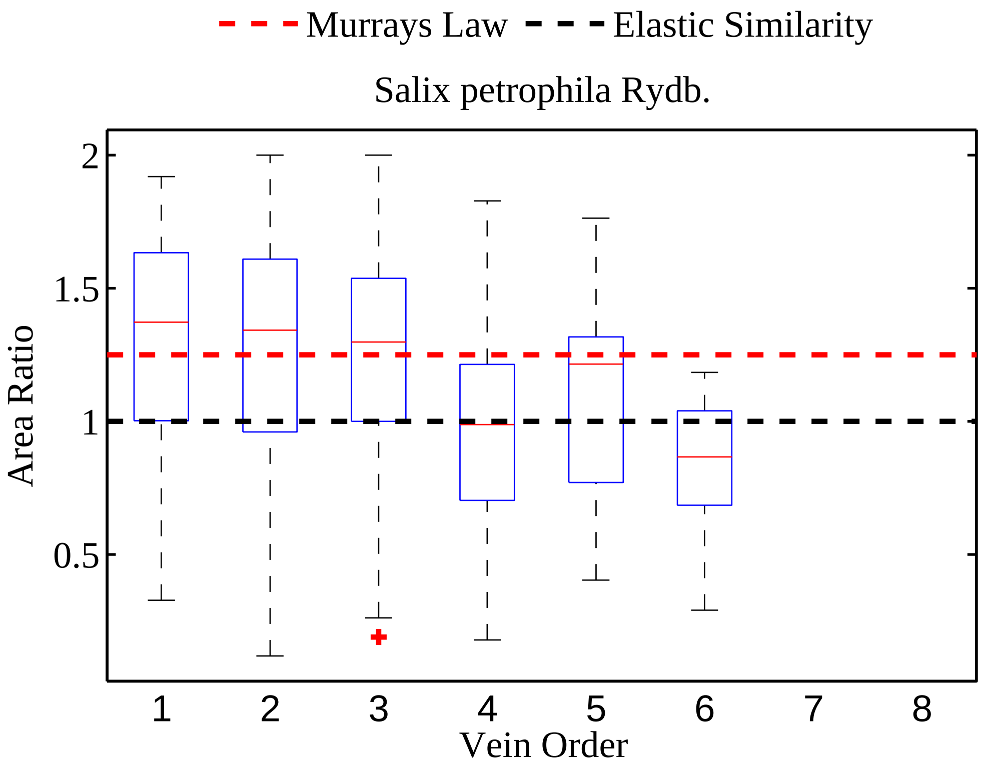

Murrays Law
Elastic Similarity
Salix petrophila Rydb.
Area Ratio
2
1.5
1
0.5
1
2
3
4
5
6
7
8
Vein Order

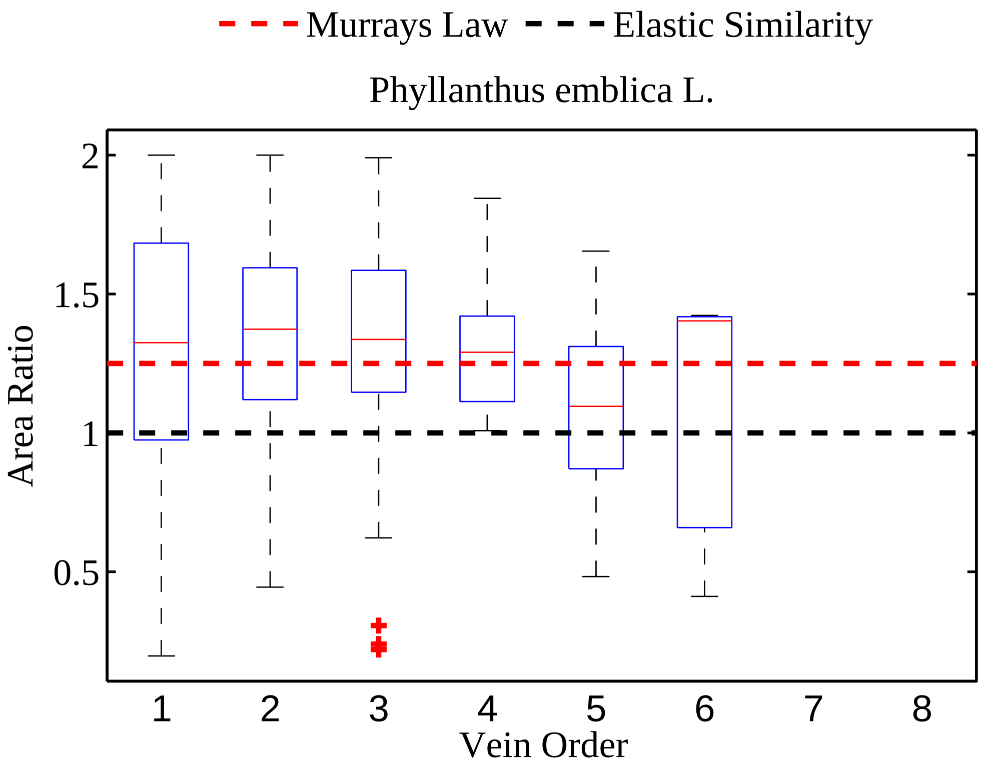

Murrays Law
Elastic Similarity
Phyllanthus emblica L.
Area Ratio
2
1.5
1
0.5
1
2
3
4
5
6
7
8
Vein Order

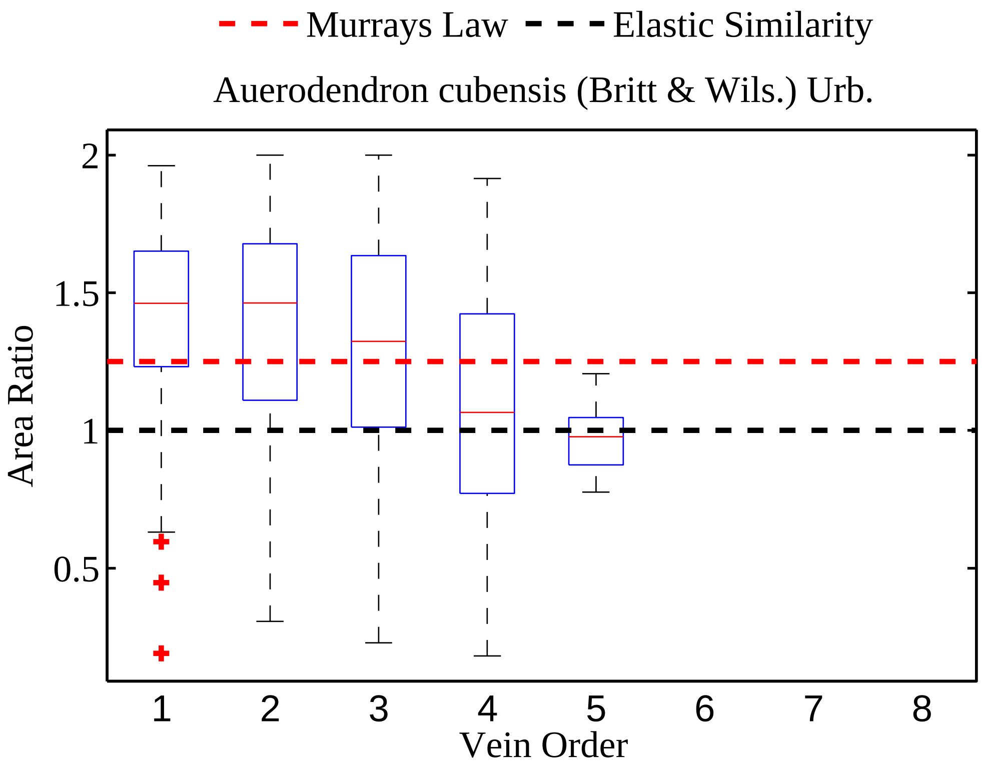

Murrays Law
Elastic Similarity
Auerodendron cubensis (Britt & Wils.) Urb.
Area Ratio
2
1.5
1
0.5
1
2
3
4
5
6
7
8
Vein Order

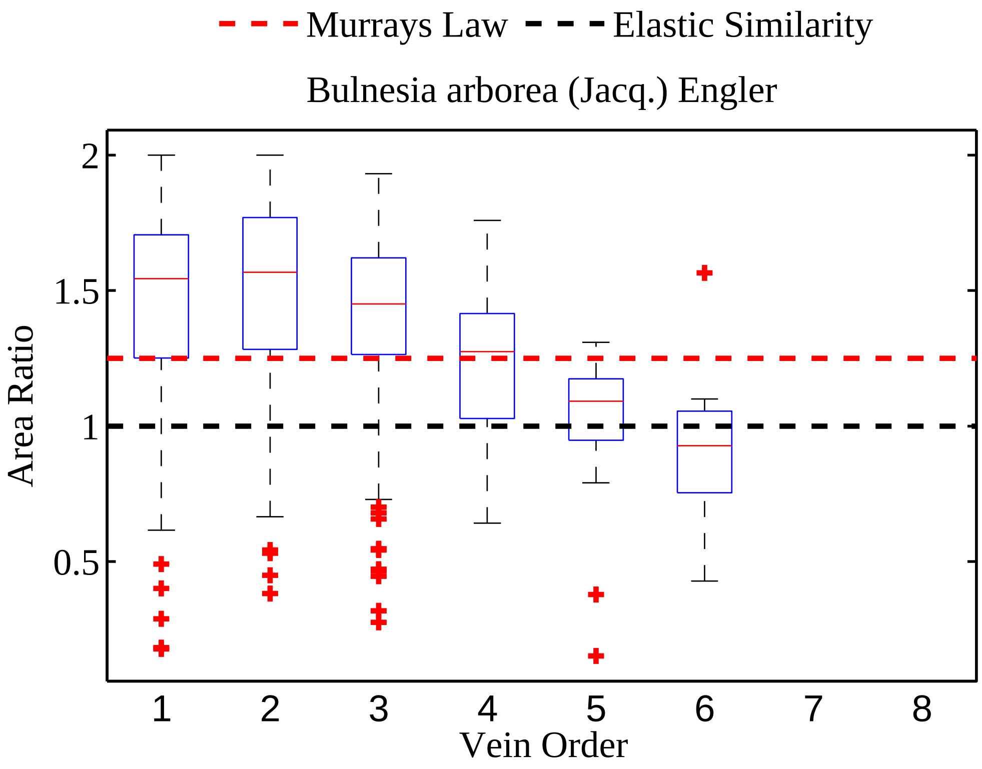

Murrays Law
Elastic Similarity
Bulnesia arborea (Jacq.) Engler
Area Ratio
2
1.5
1
0.5
1
2
3
4
5
6
7
8
Vein Order

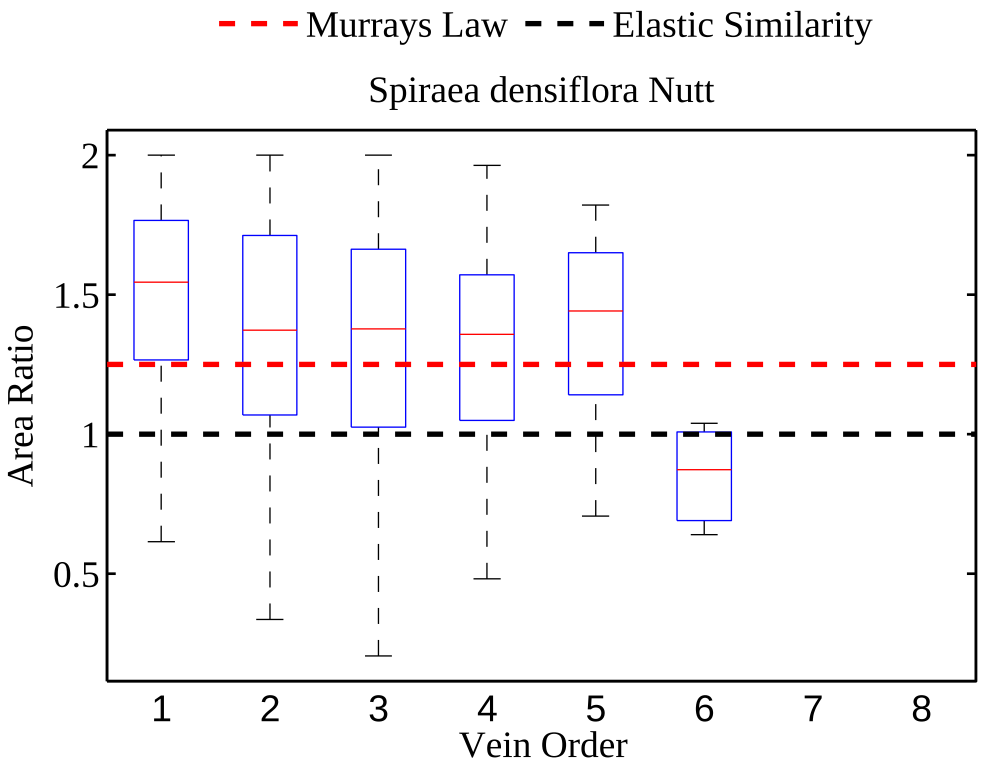
Murrays Law
Elastic Similarity
Spiraea densiflora Nutt
Area Ratio
2
1.5
1
0.5
1
2
3
4
5
6
7
8
Vein Order

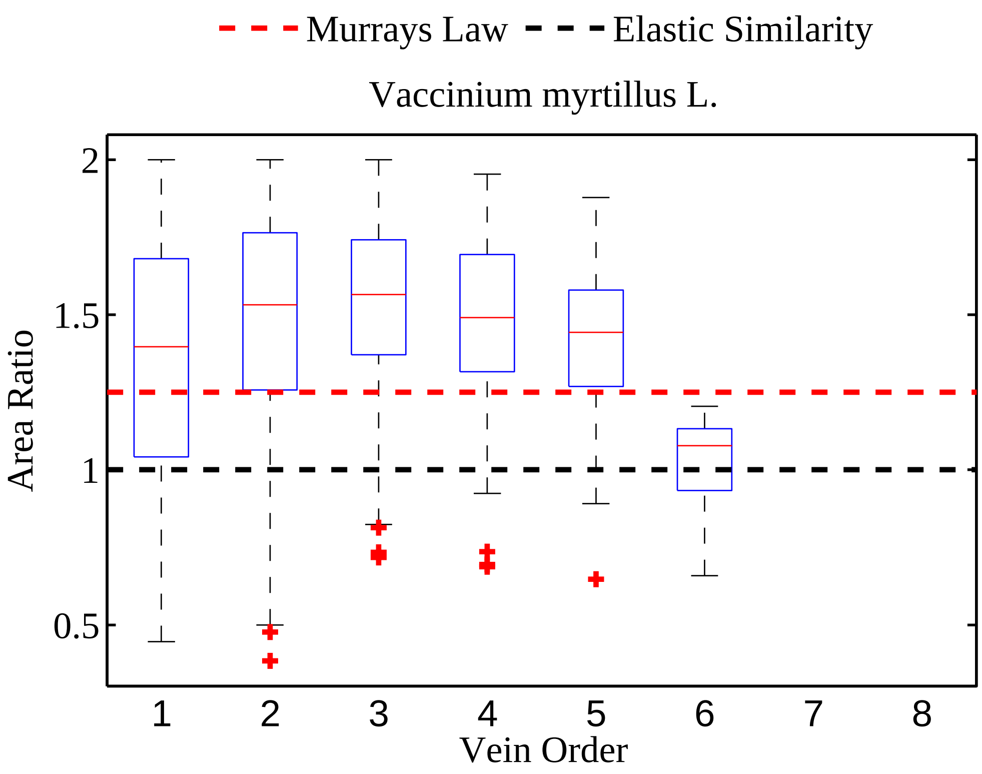

Murrays Law
Elastic Similarity
Vaccinium myrtillus L.
Area Ratio
2
1.5
1
0.5
1
2
3
4
5
6
7
8
Vein Order

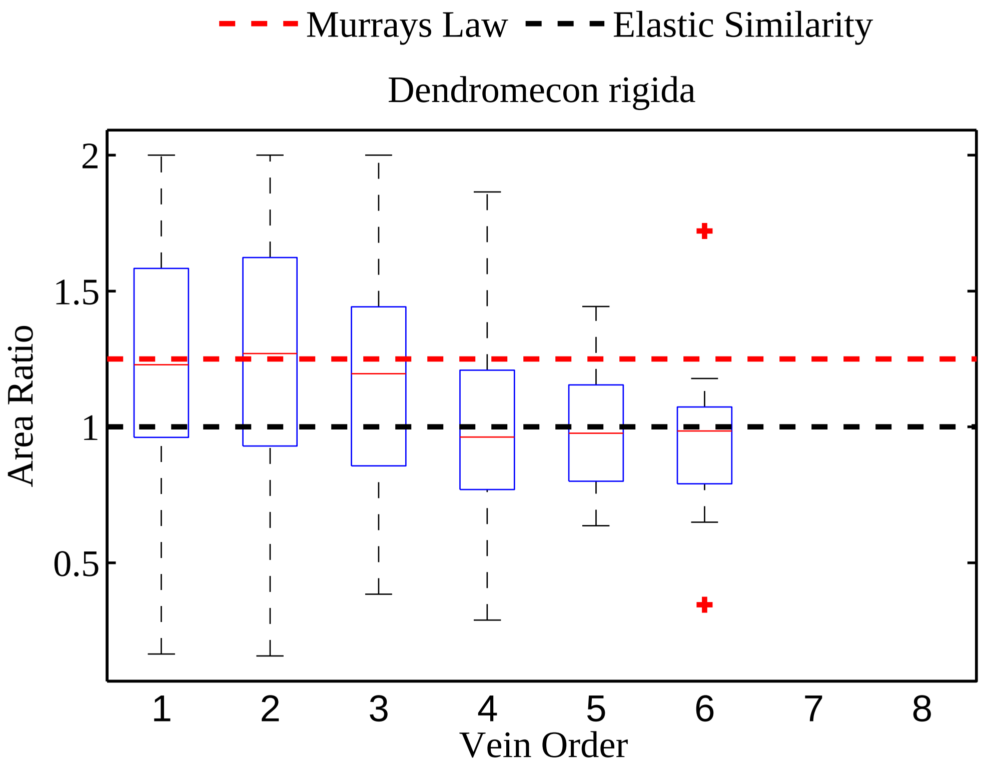

Murrays Law
Elastic Similarity
Dendromecon rigida
Area Ratio
2
1.5
1
0.5
1
2
3
4
5
6
7
8
Vein Order

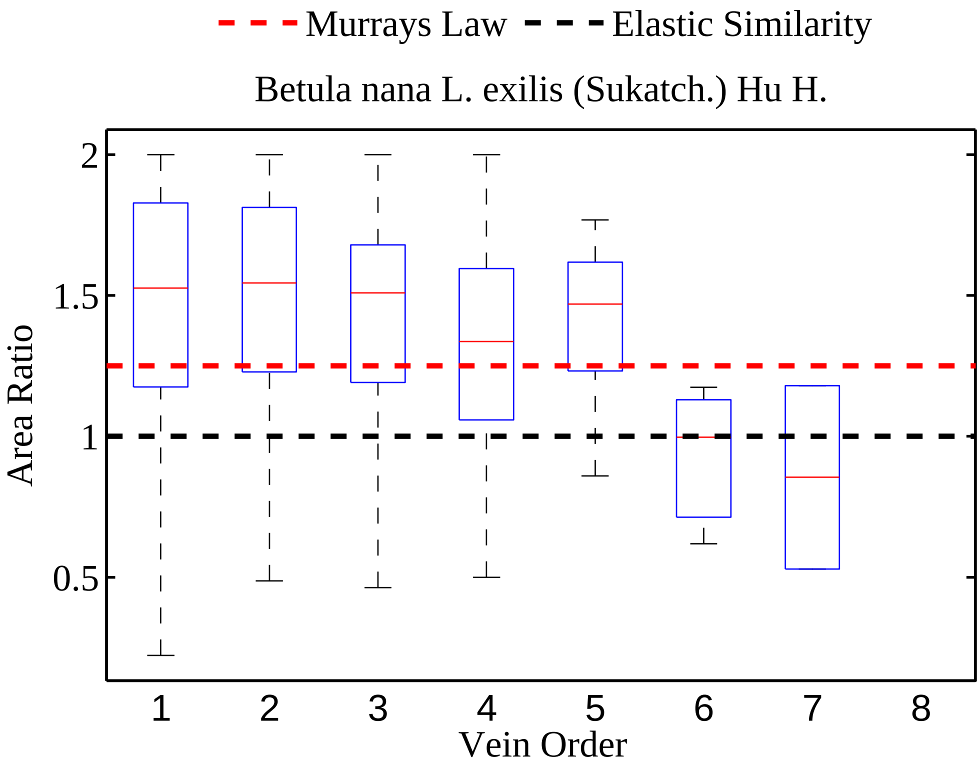

Murrays Law
Elastic Similarity
Betula nana L. exilis (Sukatch.) Hu H.
Area Ratio
2
1.5
1
0.5
1
2
3
4
5
6
7
8
Vein Order

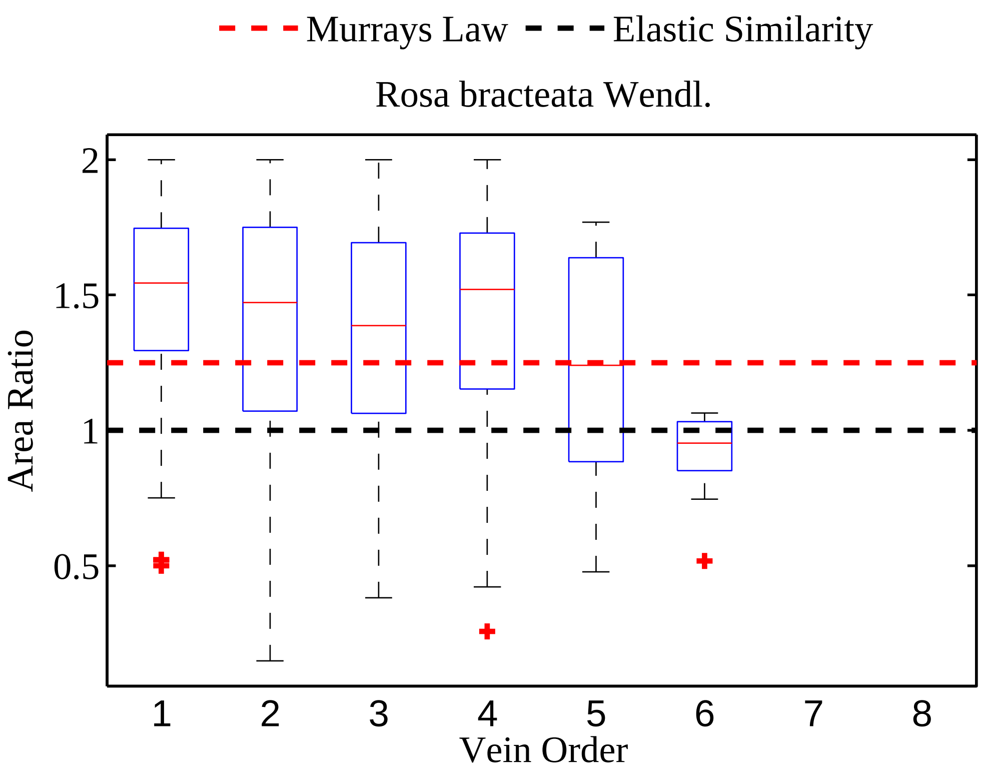

Murrays Law
Elastic Similarity
Rosa bracteata Wendl.
Area Ratio
2
1.5
1
0.5
1
2
3
4
5
6
7
8
Vein Order

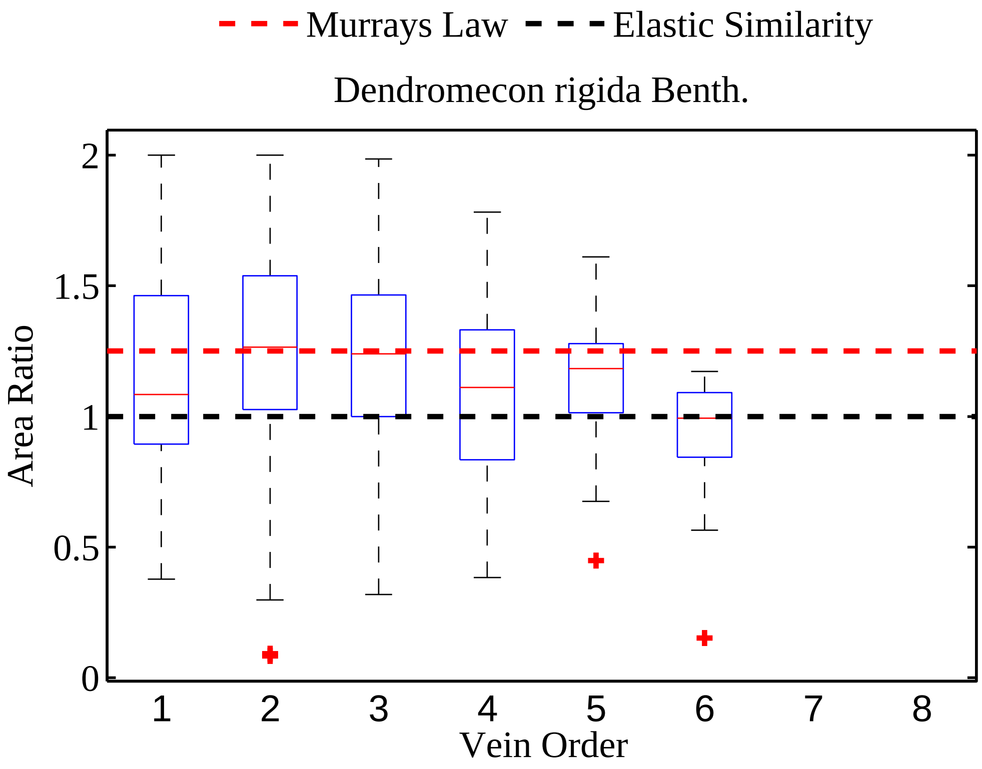

Murrays Law
Elastic Similarity
Dendromecon rigida Benth.
Area Ratio
0
0.5
1
1.5
2
1
2
3
4
5
6
7
8
Vein Order

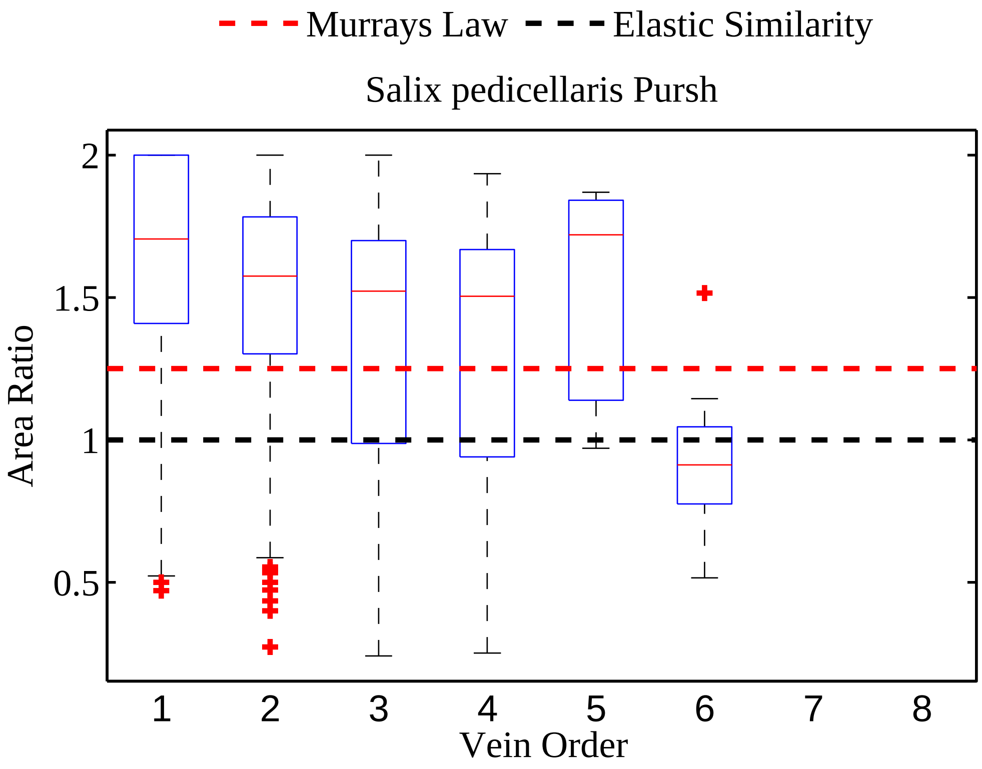

Murrays Law
Elastic Similarity
Salix pedicellaris Pursh
Area Ratio
2
1.5
1
0.5
1
2
3
4
5
6
7
8
Vein Order

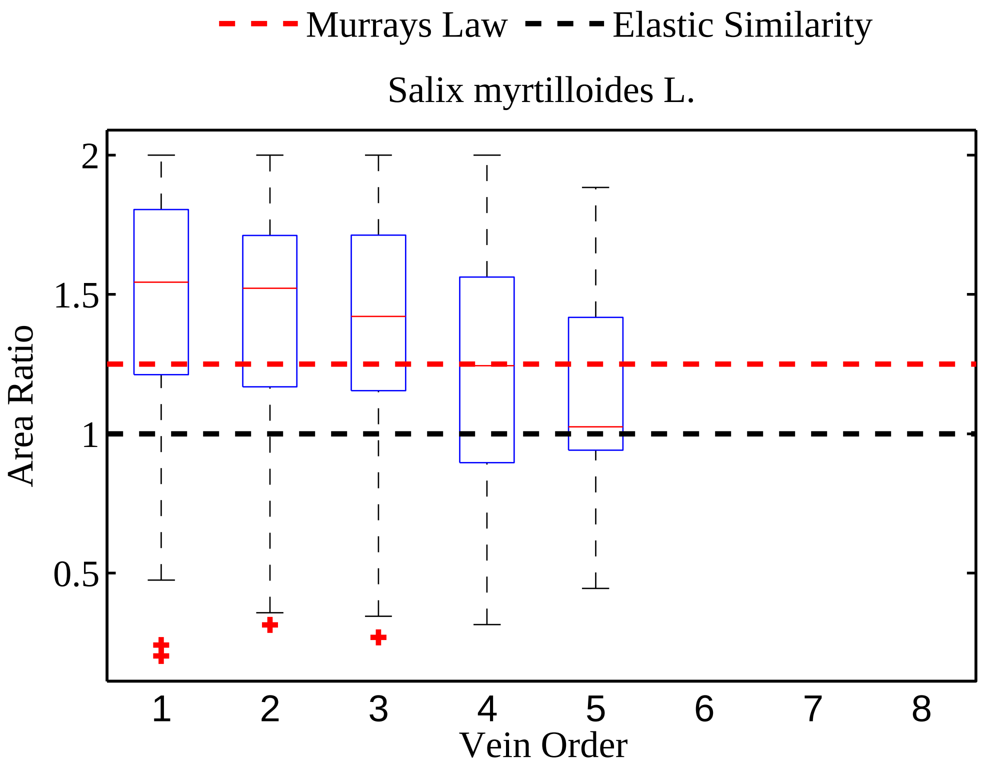

Murrays Law
Elastic Similarity
Salix myrtilloides L.
Area Ratio
2
1.5
1
0.5
1
2
3
4
5
6
7
8
Vein Order

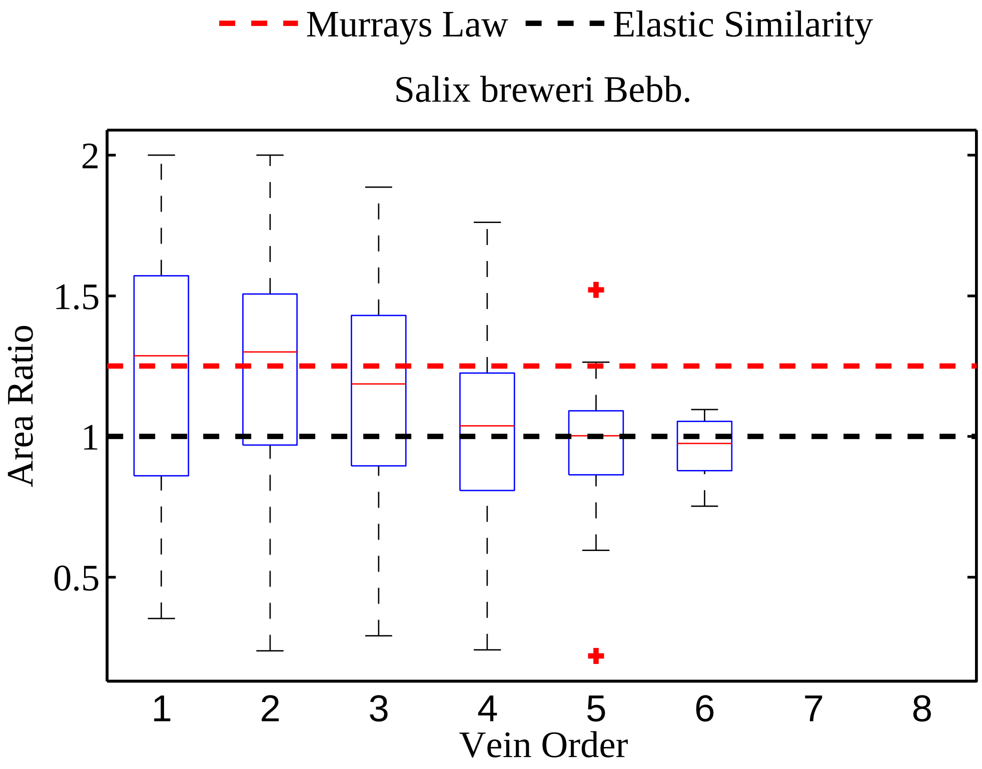

Murrays Law
Elastic Similarity
Salix breweri Bebb.
Area Ratio
2
1.5
1
0.5
1
2
3
4
5
6
7
8
Vein Order

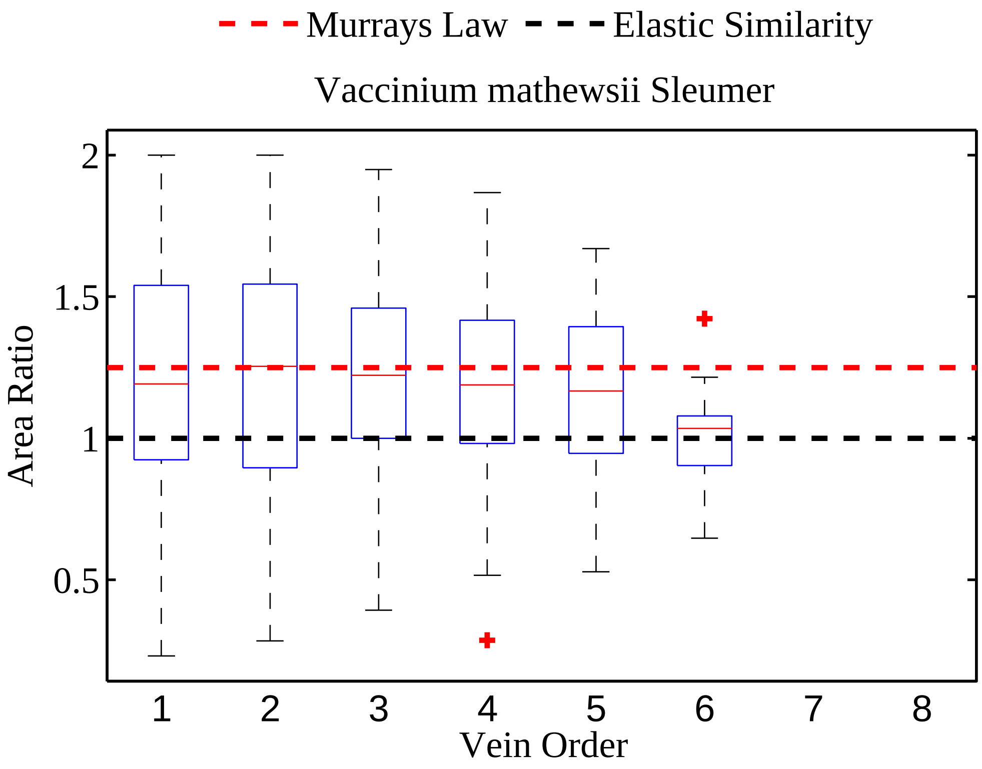

Murrays Law
Elastic Similarity
Vaccinium mathewsii Sleumer
Area Ratio
2
1.5
1
0.5
1
2
3
4
5
6
7
8
Vein Order

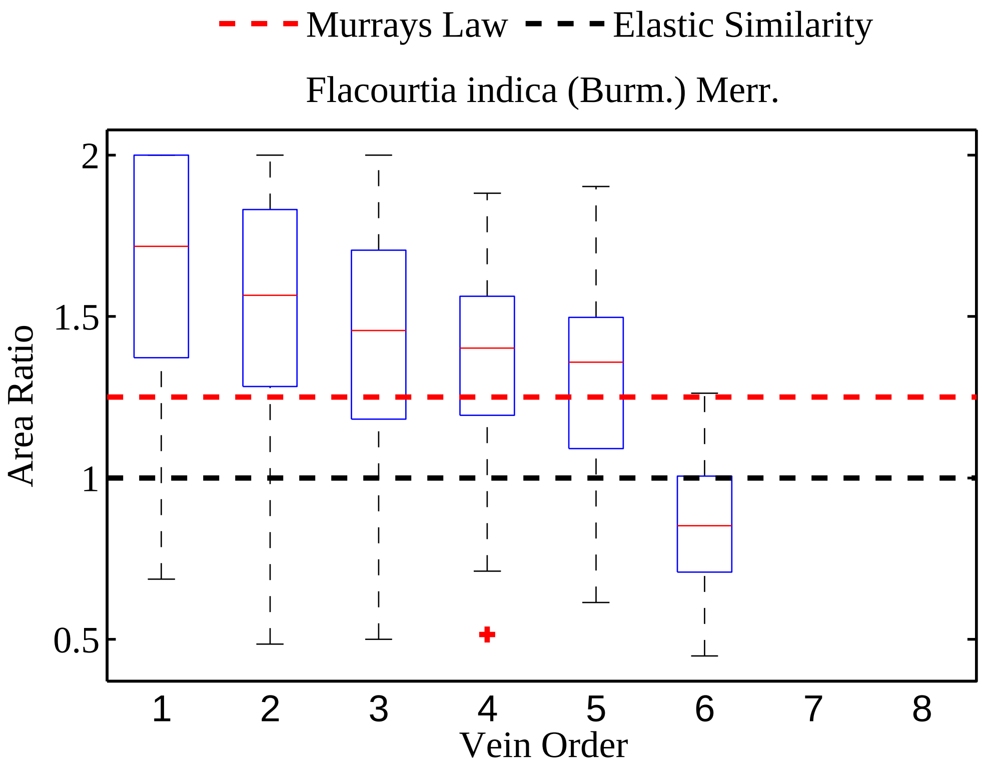

Murrays Law
Elastic Similarity
Flacourtia indica (Burm.) Merr.
Area Ratio
2
1.5
1
0.5
1
2
3
4
5
6
7
8
Vein Order

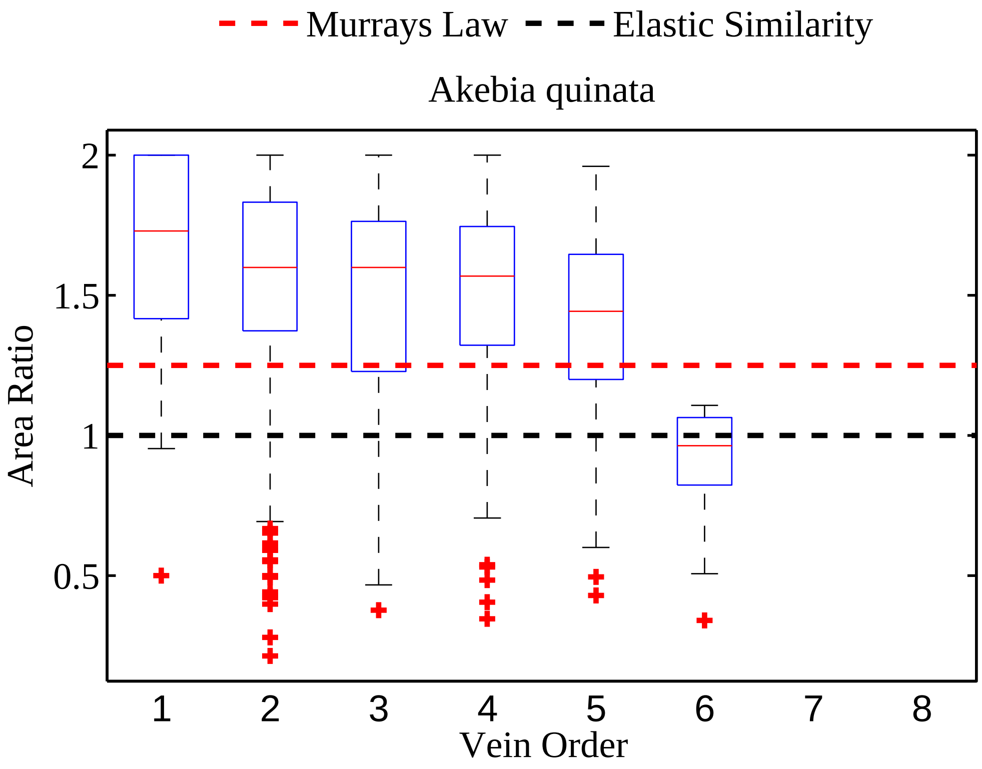

Murrays Law
Elastic Similarity
Akebia quinata
Area Ratio
2
1.5
1
0.5
1
2
3
4
5
6
7
8
Vein Order

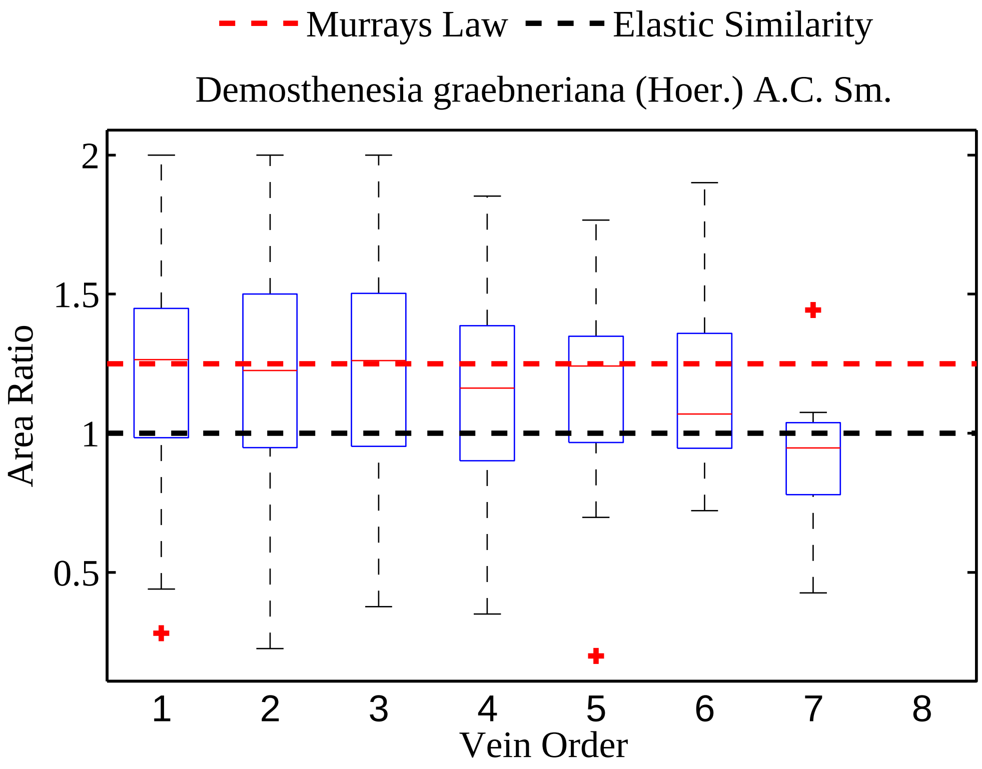
Murrays Law
Elastic Similarity
Demosthenesia graebneriana (Hoer.) A.C. Sm.
Area Ratio
Vein Order
2
1.5
1
0.5
1
2
3
4
5
6
7
8

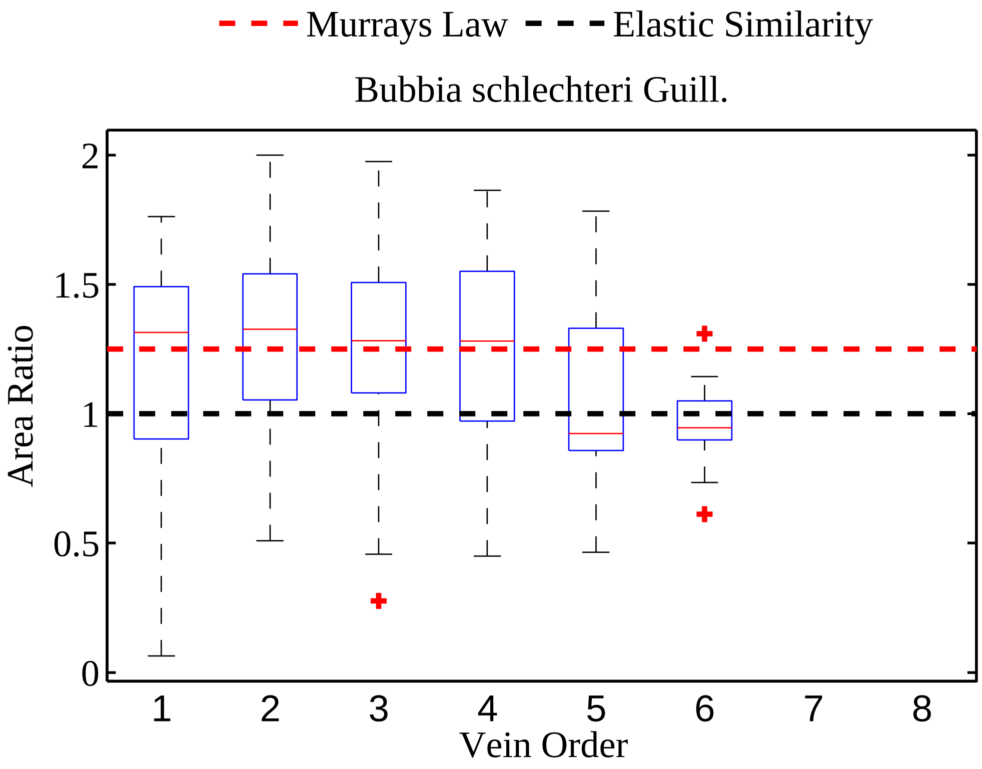

Murrays Law
Elastic Similarity
Bubbia schlechteri Guill.
Area Ratio
0
0.5
1
1.5
2
1
2
3
4
5
6
7
8
Vein Order

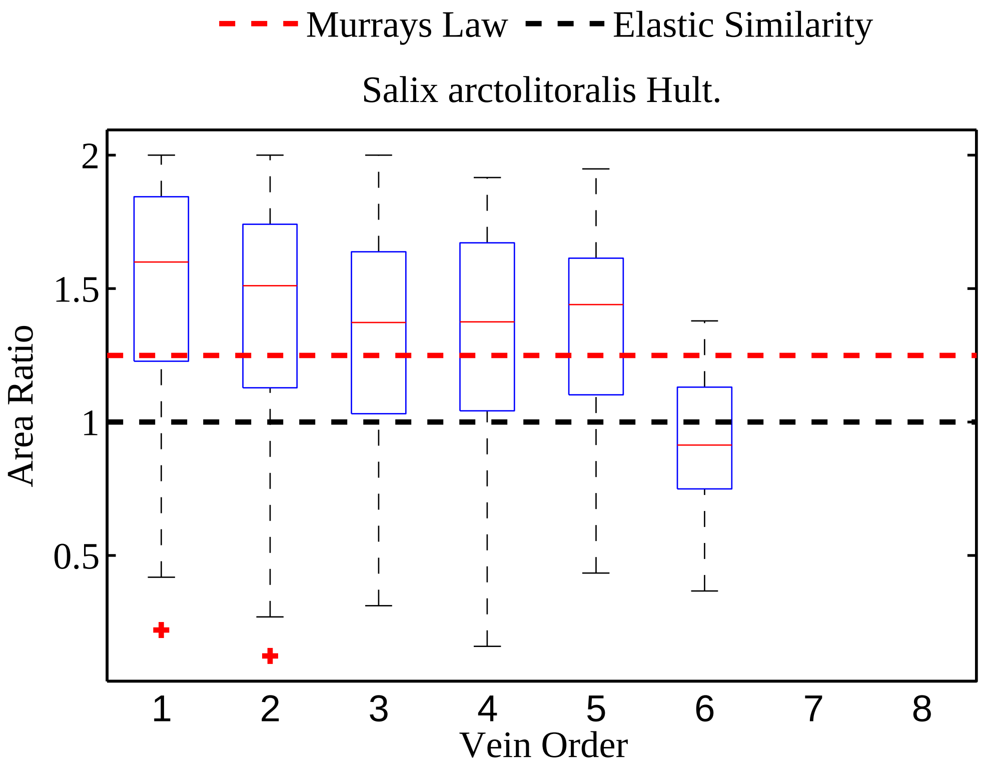

Murrays Law
Elastic Similarity
Salix arctolitoralis Hult.
Area Ratio
2
1.5
1
0.5
1
2
3
4
5
6
7
8
Vein Order

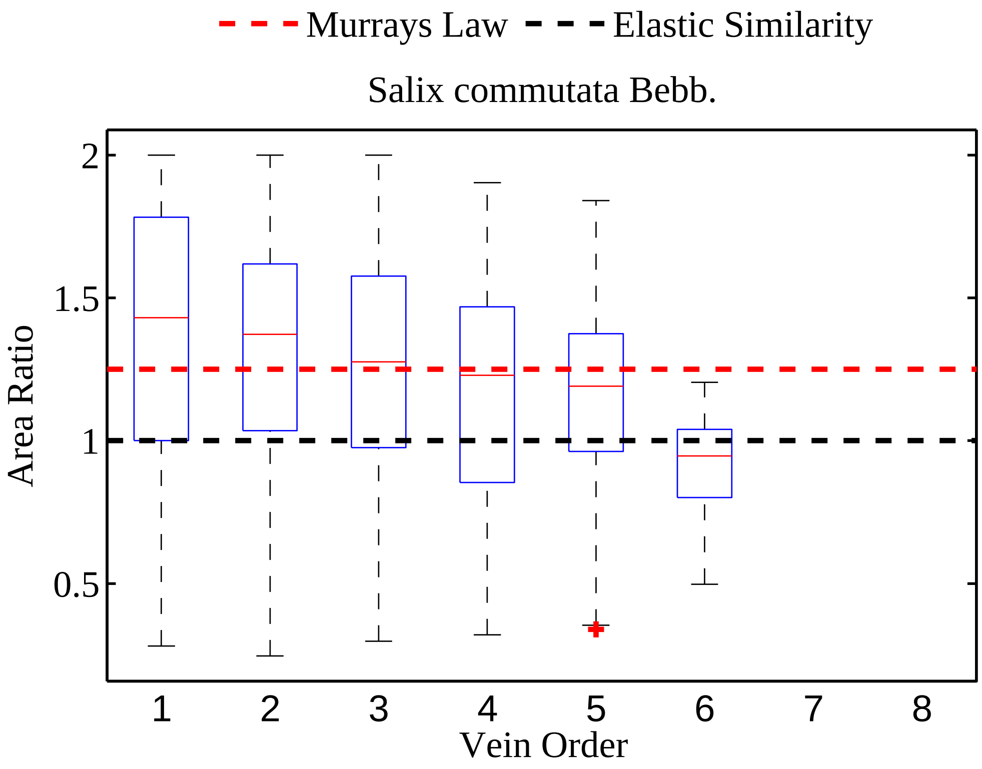

Murrays Law
Elastic Similarity
Salix commutata Bebb.
Area Ratio
Vein Order
2
1.5
1
0.5
1
2
3
4
5
6
7
8

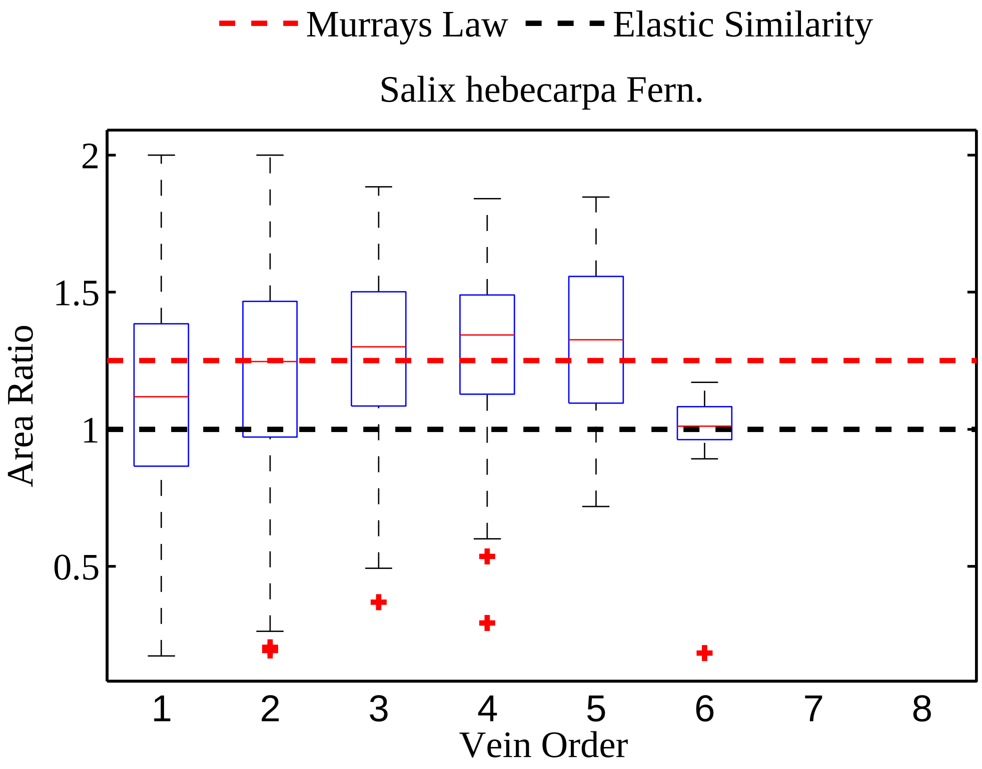
Murrays Law
Elastic Similarity
Salix hebecarpa Fern.
Area Ratio
2
1.5
1
0.5
1
2
3
4
5
6
7
8
Vein Order

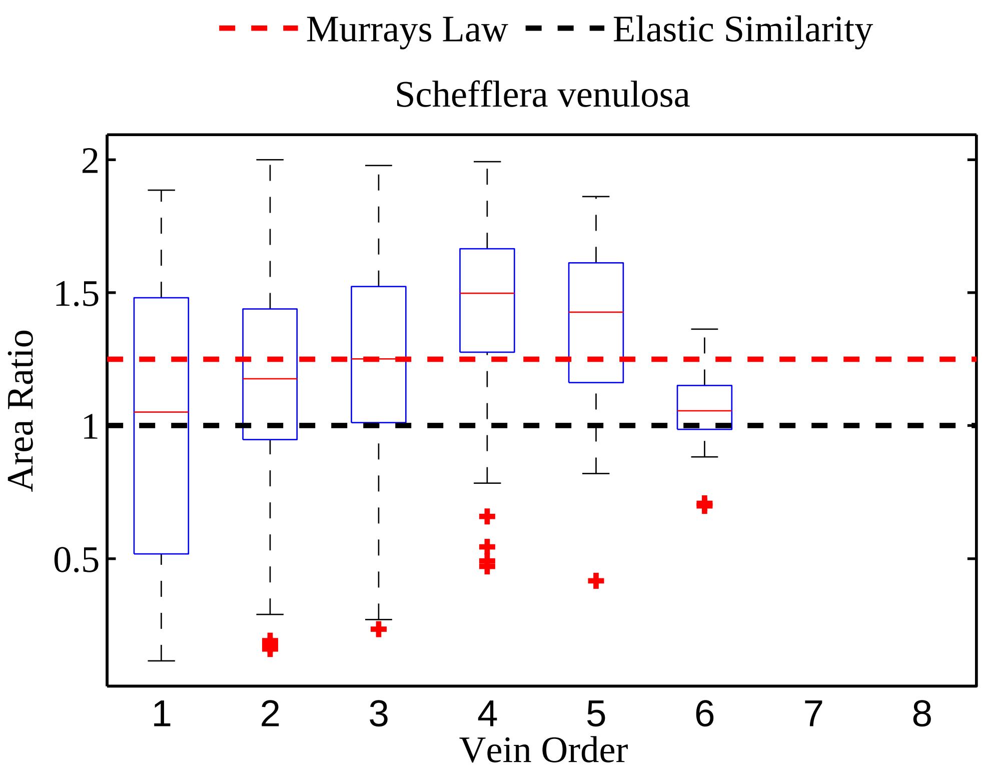

Murrays Law
Elastic Similarity
Schefflera venulosa
Area Ratio
2
1.5
1
0.5
1
2
3
4
5
6
7
8
Vein Order

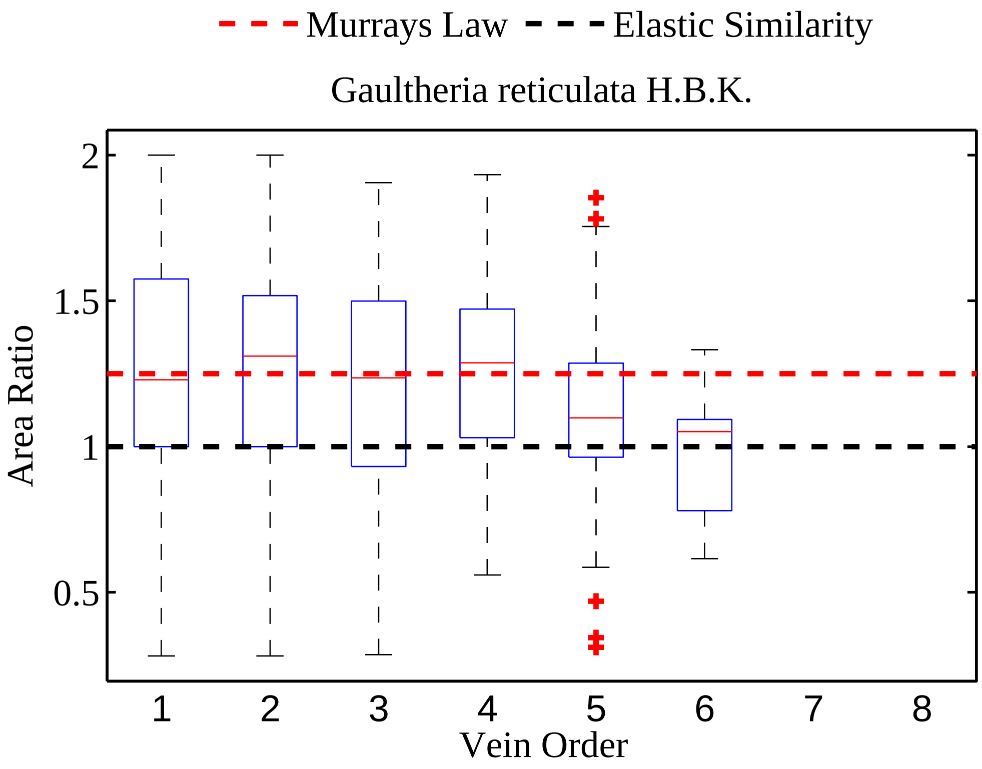
Murrays Law
Elastic Similarity
Gaultheria reticulata H.B.K.
Area Ratio
2
1.5
1
0.5
1
2
3
4
5
6
7
8
Vein Order

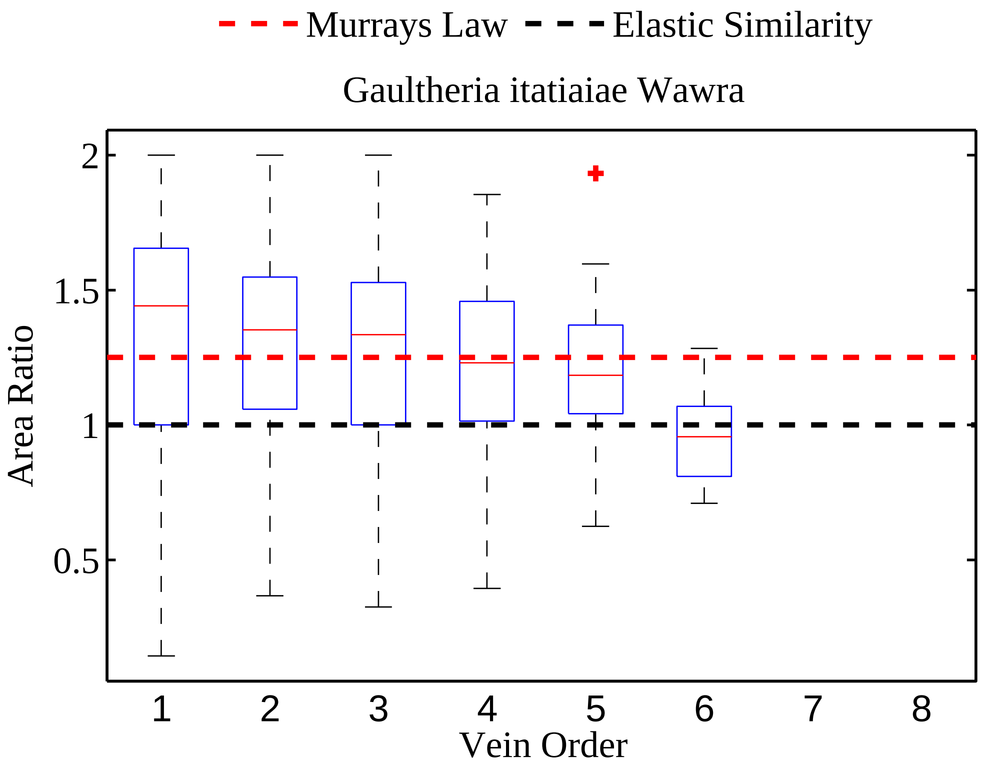
Murrays Law
Elastic Similarity
Gaultheria itatiaiae Wawra
Area Ratio
2
1.5
1
0.5
1
2
3
4
5
6
7
8
Vein Order

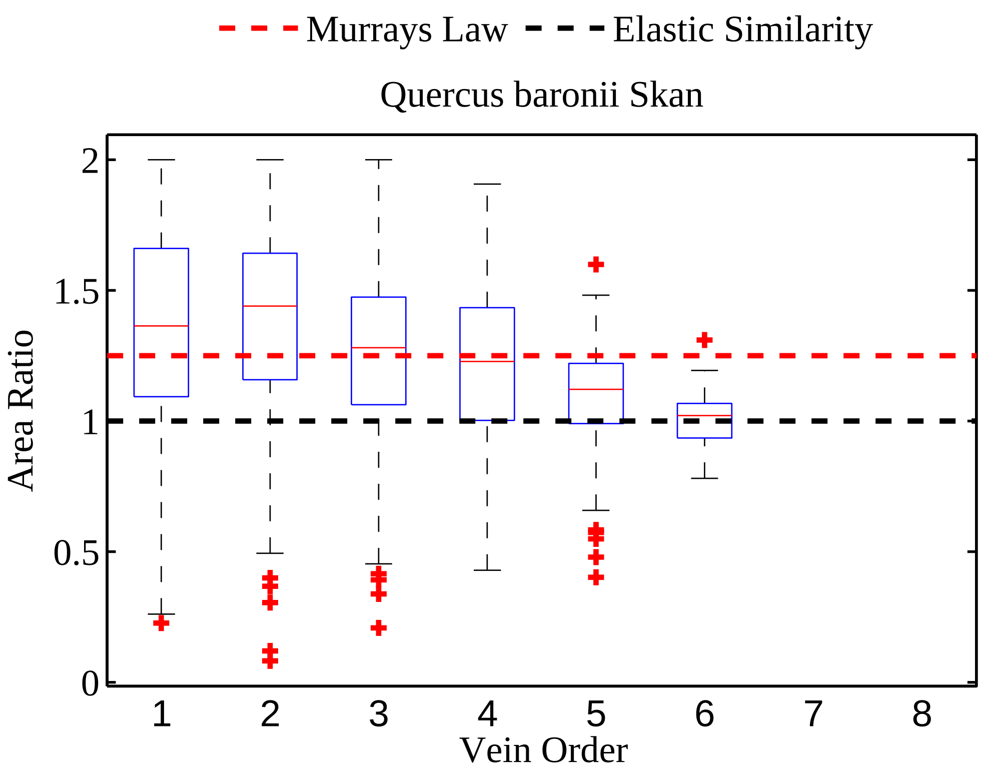
Murrays Law
Elastic Similarity
Quercus baronii Skan
Area Ratio
0
0.5
1
1.5
2
1
2
3
4
5
6
7
8
Vein Order

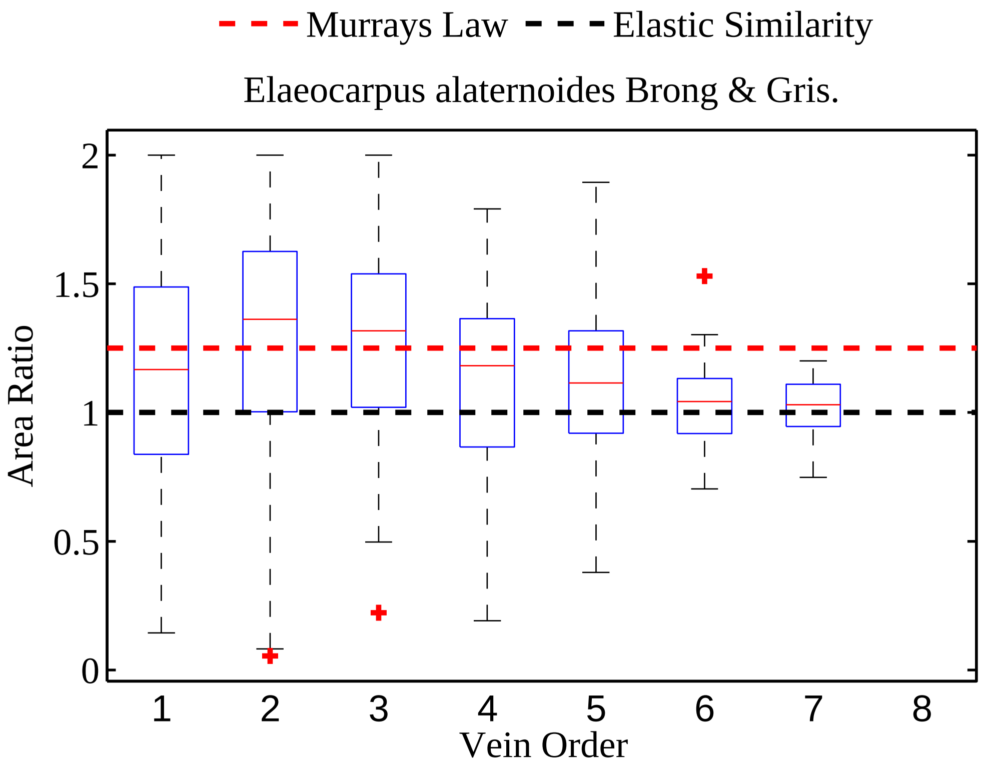

Murrays Law
Elastic Similarity
Elaeocarpus alaternoides Brong & Gris.
Area Ratio
0
0.5
1
1.5
2
1
2
3
4
5
6
7
8
Vein Order

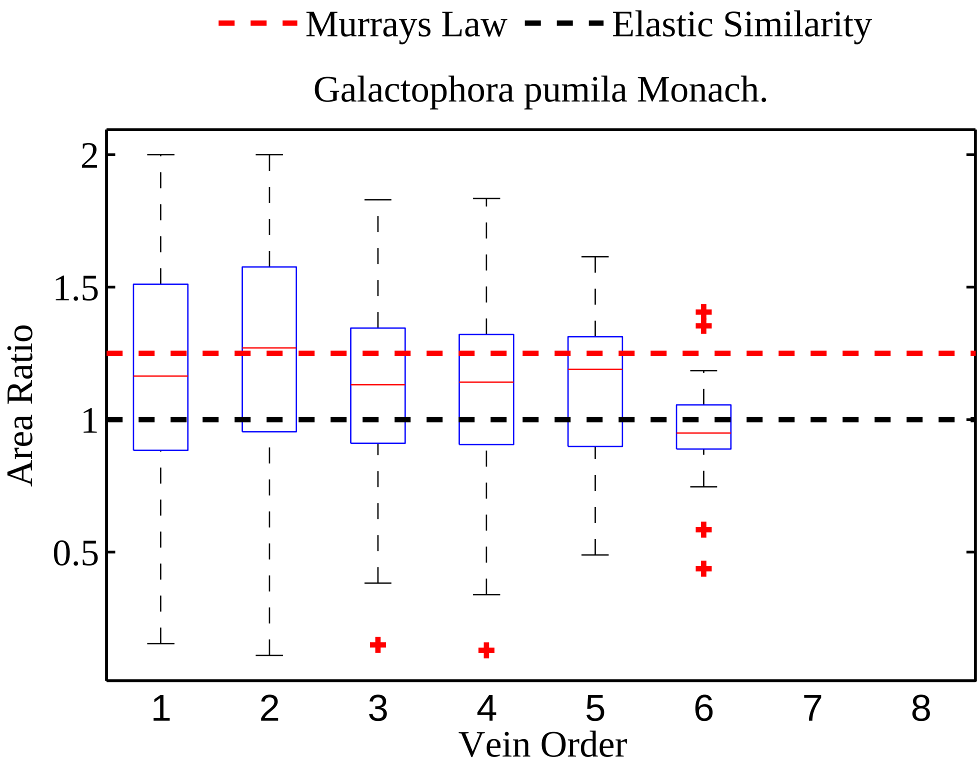

Murrays Law
Elastic Similarity
Galactophora pumila Monach.
Area Ratio
2
1.5
1
0.5
1
2
3
4
5
6
7
8
Vein Order

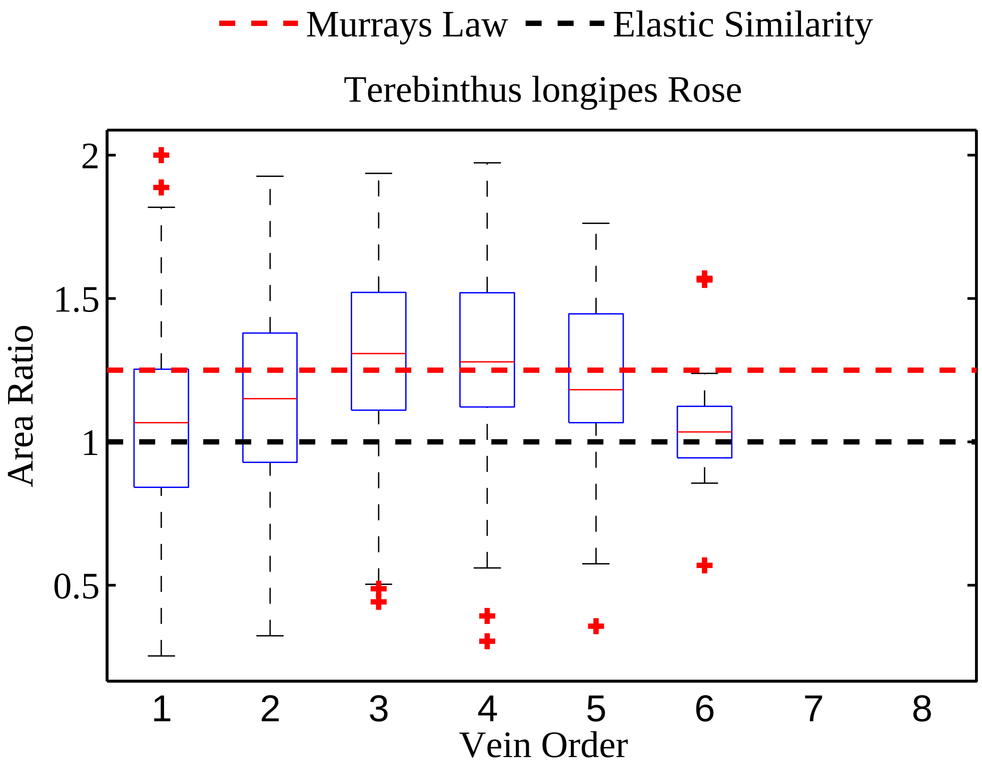

Murrays Law
Elastic Similarity
Terebinthus longipes Rose
Area Ratio
2
1.5
1
0.5
1
2
3
4
5
6
7
8
Vein Order

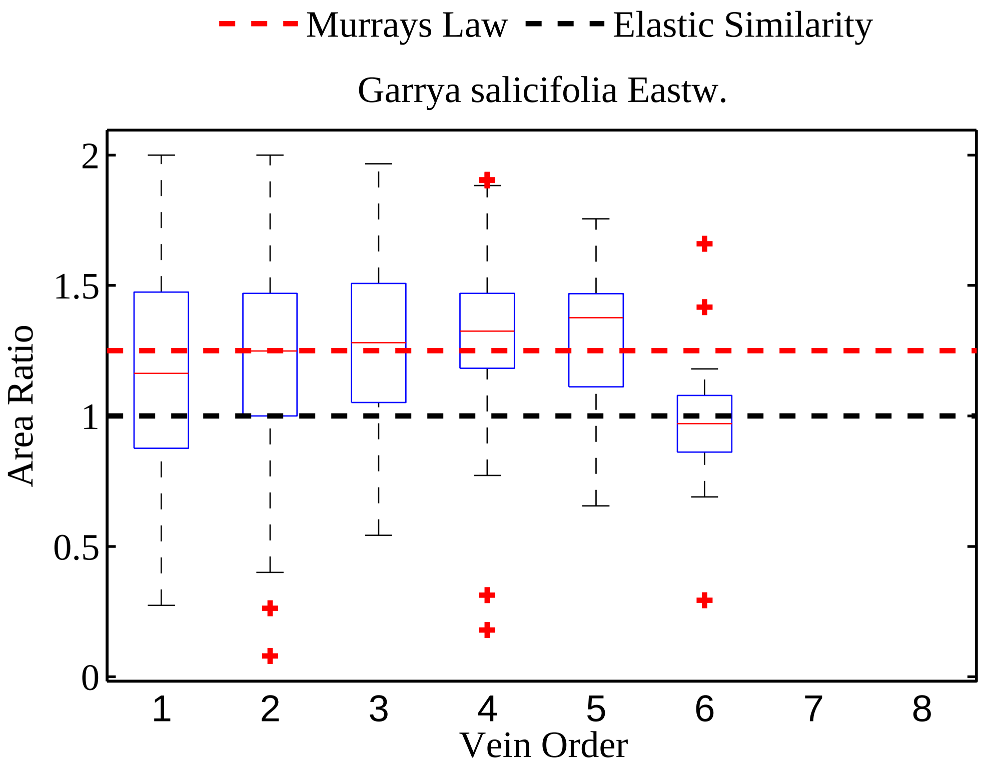

Murrays Law
Elastic Similarity
Garrya salicifolia Eastw.
Area Ratio
0
0.5
1
1.5
2
1
2
3
4
5
6
7
8
Vein Order

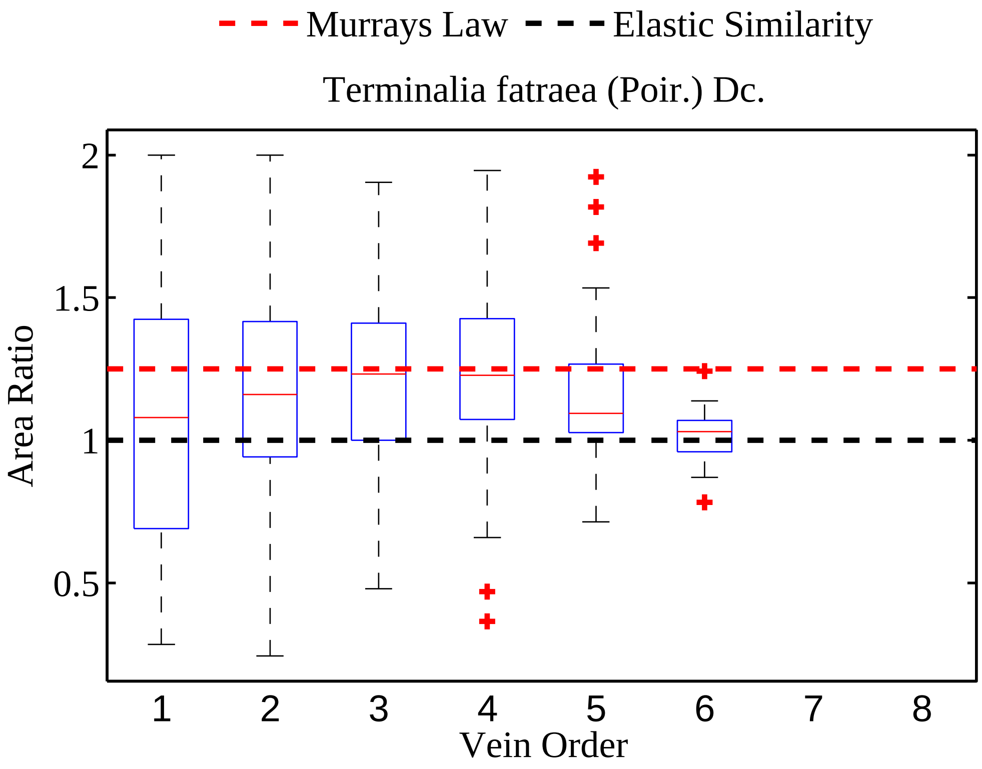

Murrays Law
Elastic Similarity
Terminalia fatraea (Poir.) Dc.
Area Ratio
2
1.5
1
0.5
1
2
3
4
5
6
7
8
Vein Order

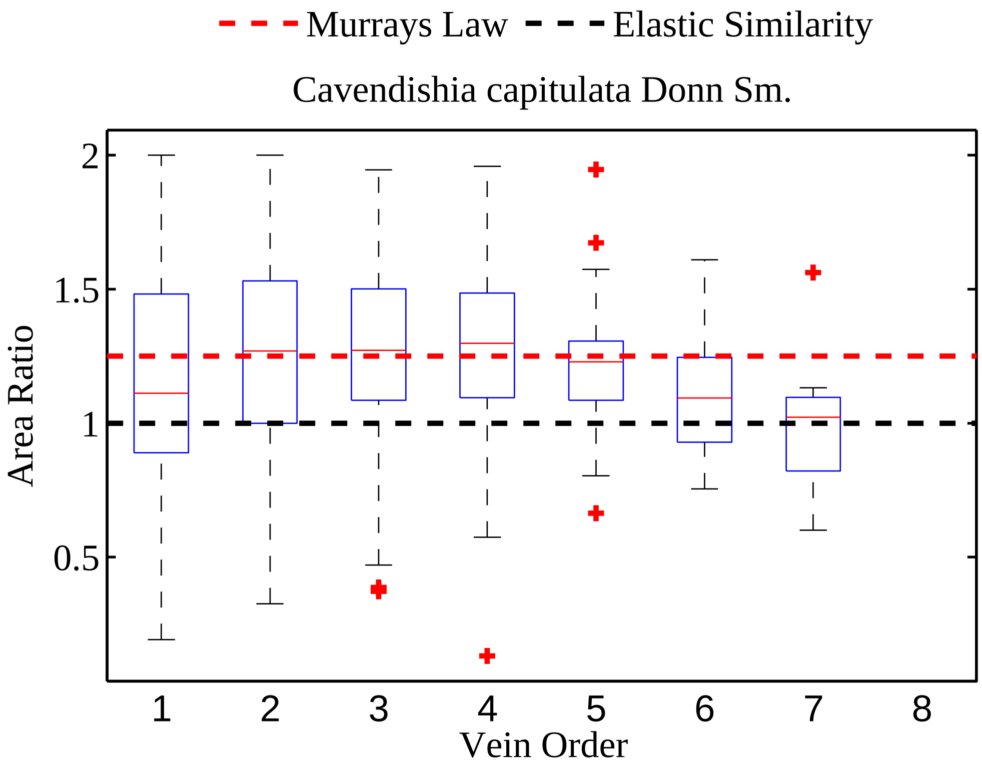

Murrays Law
Elastic Similarity
Cavendishia capitulata Donn Sm.
Area Ratio
2
1.5
1
0.5
1
2
3
4
5
6
7
8
Vein Order

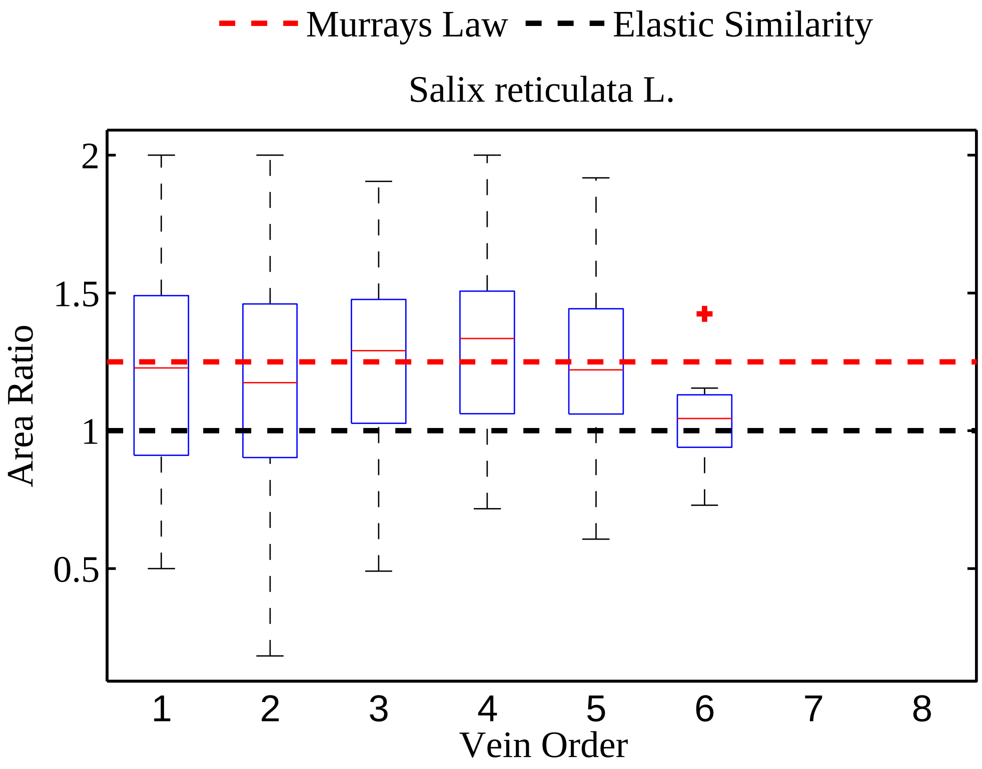

Murrays Law
Elastic Similarity
Salix reticulata L.
Area Ratio
2
1.5
1
0.5
1
2
3
4
5
6
7
8
Vein Order

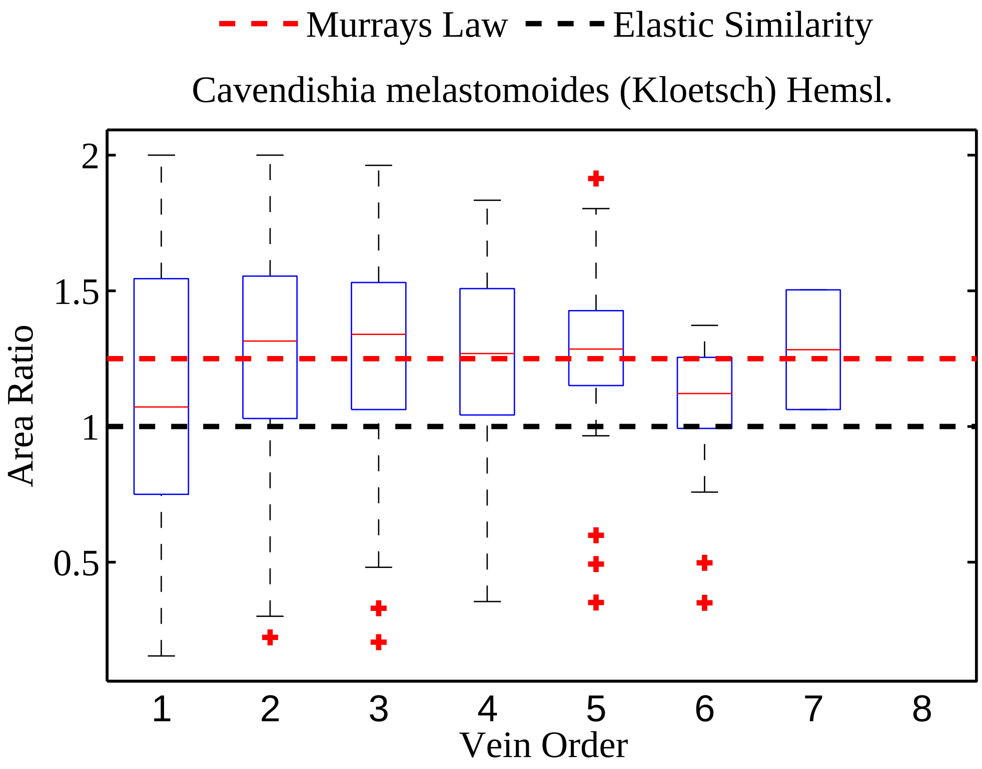

Murrays Law
Elastic Similarity
Cavendishia melastomoides (Kloetsch) Hemsl.
Area Ratio
2
1.5
1
0.5
1
2
3
4
5
6
7
8
Vein Order

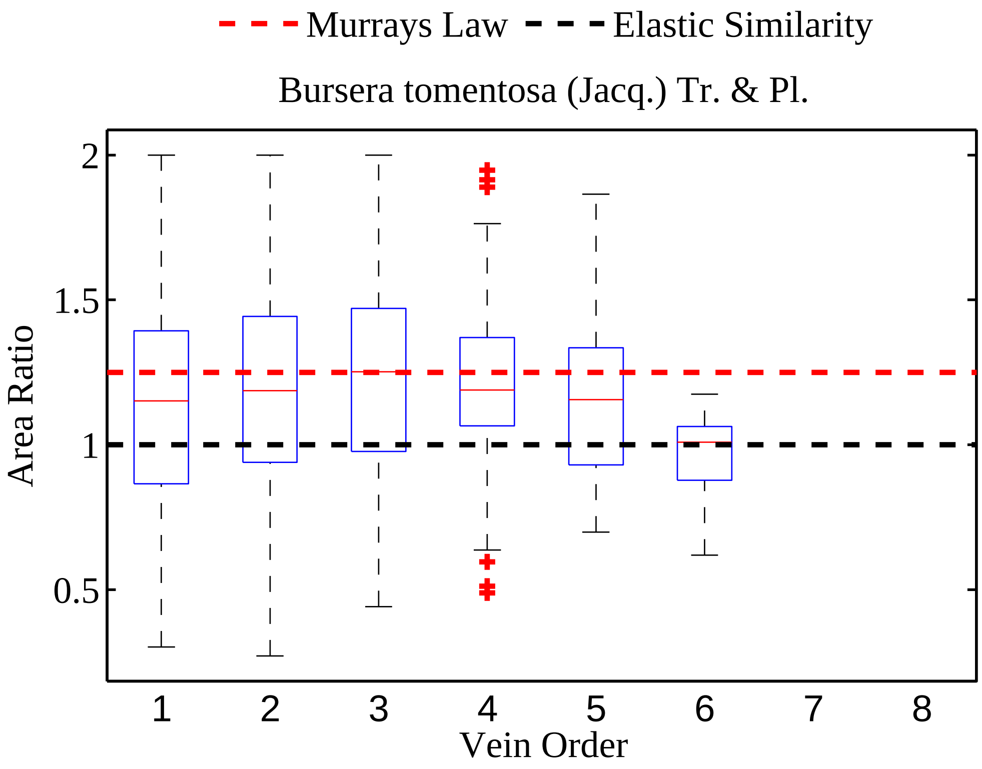

Murrays Law
Elastic Similarity
Bursera tomentosa (Jacq.) Tr. & Pl.
Area Ratio
Vein Order
2
1.5
1
0.5
1
2
3
4
5
6
7
8

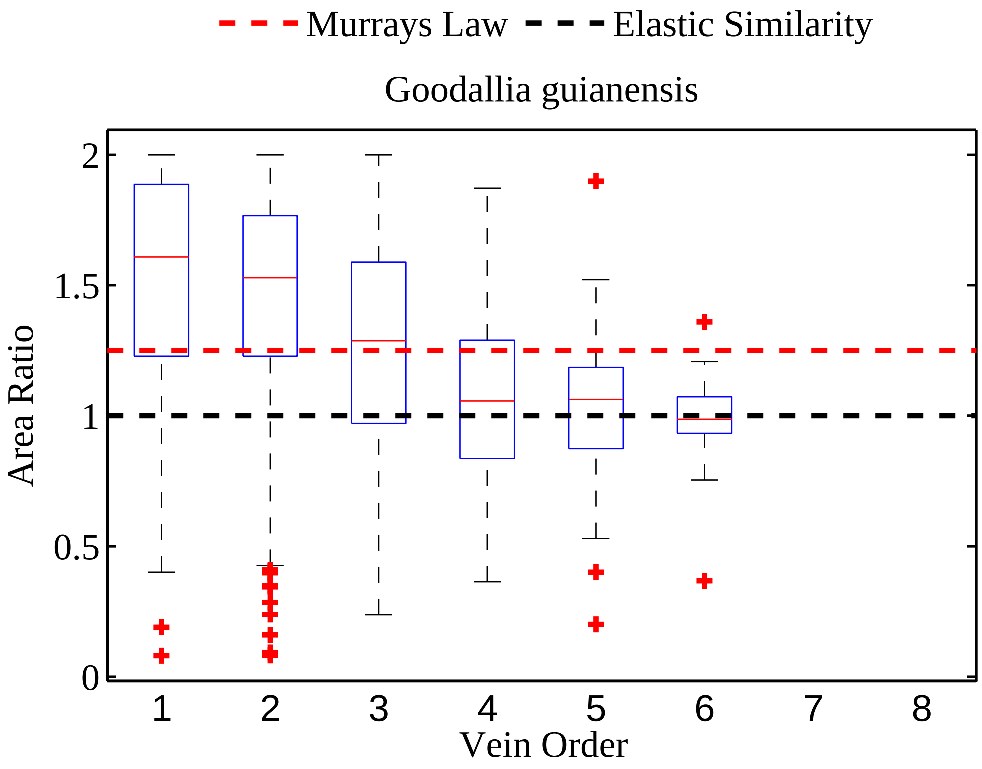

Murrays Law
Elastic Similarity
Goodallia guianensis
Area Ratio
Vein Order
2
1.5
1
0.5
0
1
2
3
4
5
6
7
8

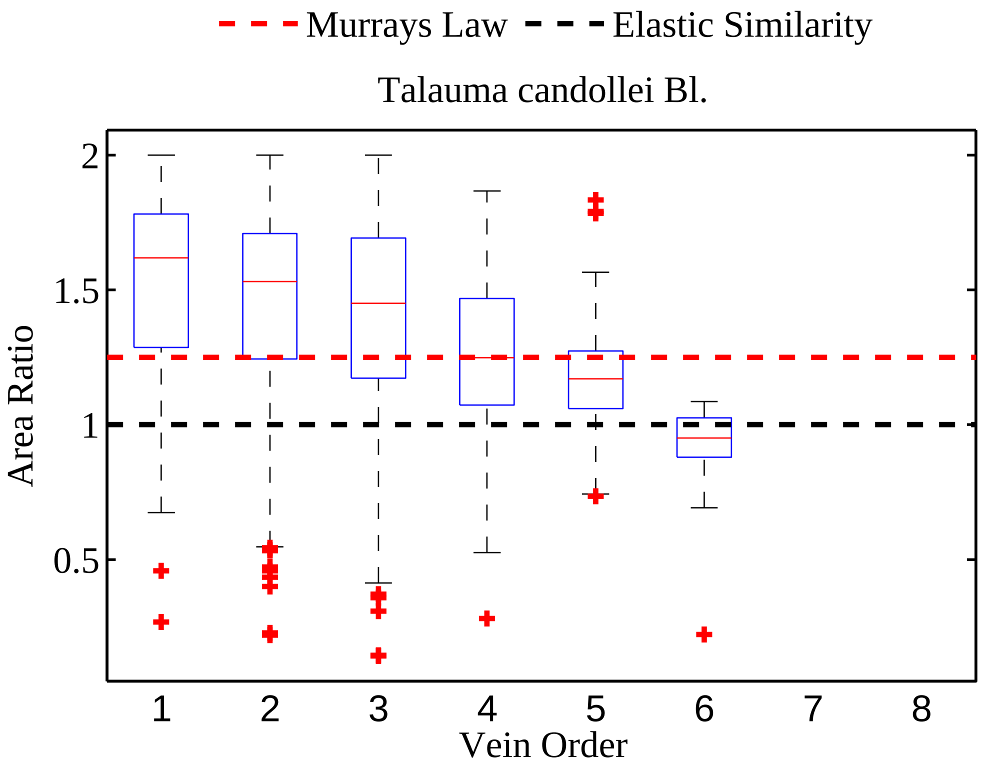
Murrays Law
Elastic Similarity
Talauma candollei Bl.
Area Ratio
2
1.5
1
0.5
1
2
3
4
5
6
7
8
Vein Order

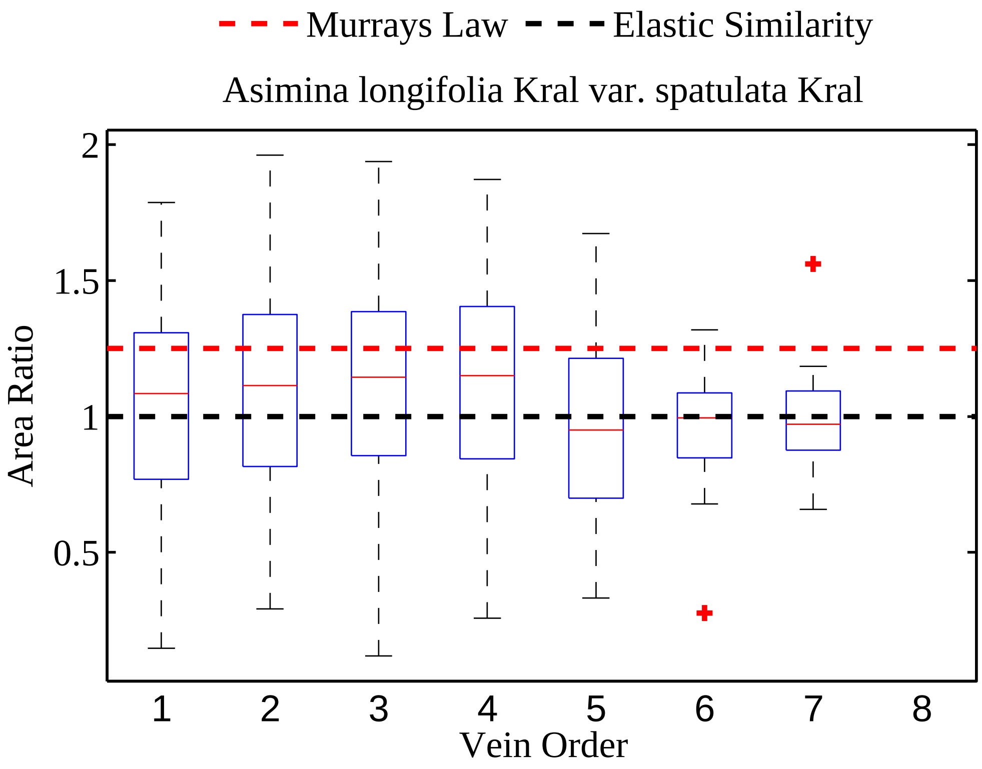
Murrays Law
Elastic Similarity
Asimina longifolia Kral var. spatulata Kral
Area Ratio
2
1.5
1
0.5
1
2
3
4
5
6
7
8
Vein Order

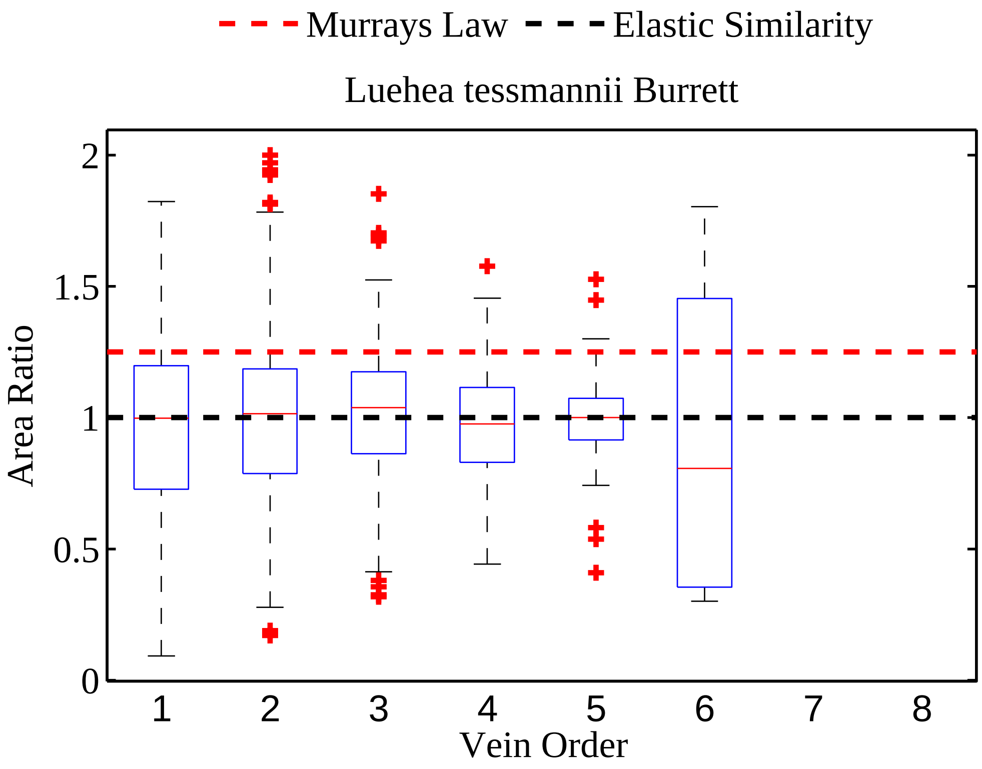

Murrays Law
Elastic Similarity
Luehea tessmannii Burrett
Area Ratio
0
0.5
1
1.5
2
1
2
3
4
5
6
7
8
Vein Order

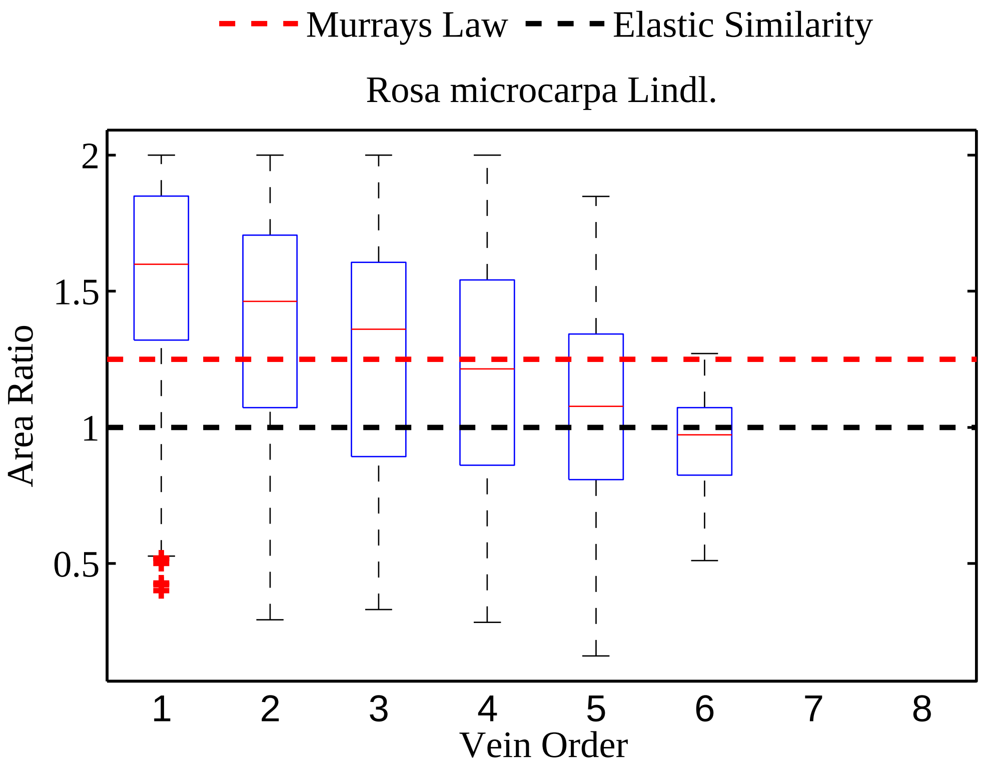
Murrays Law
Elastic Similarity
Rosa microcarpa Lindl.
Area Ratio
2
1.5
1
0.5
1
2
3
4
5
6
7
8
Vein Order

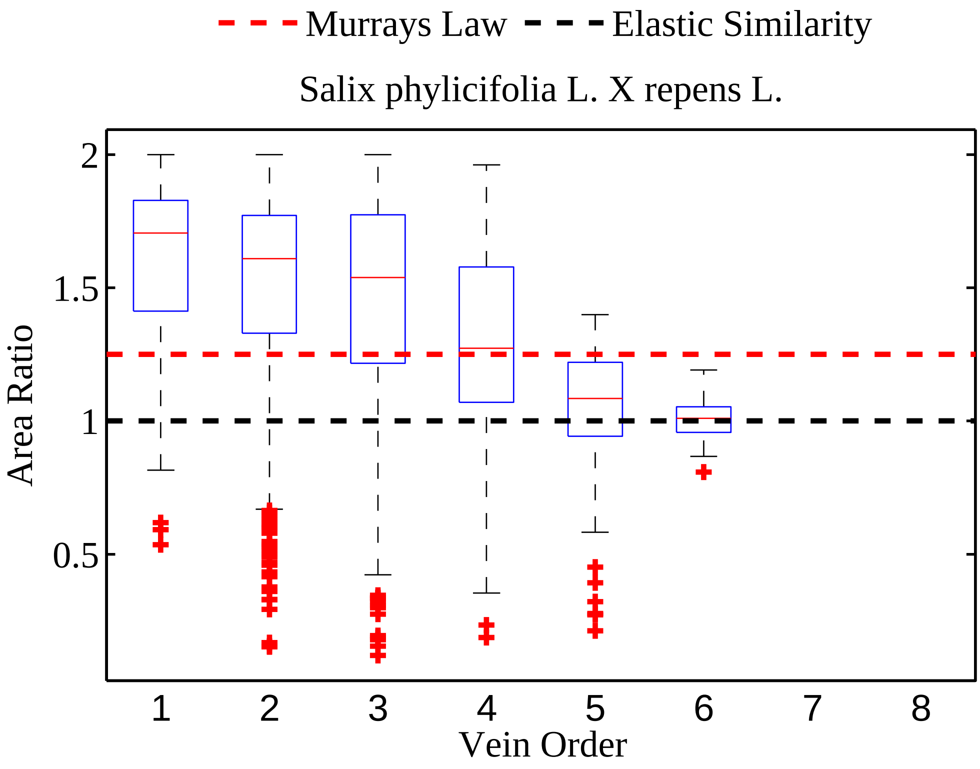
Murrays Law
Elastic Similarity
Salix phylicifolia L. X repens L.
Area Ratio
Vein Order
2
1.5
1
0.5
1
2
3
4
5
6
7
8

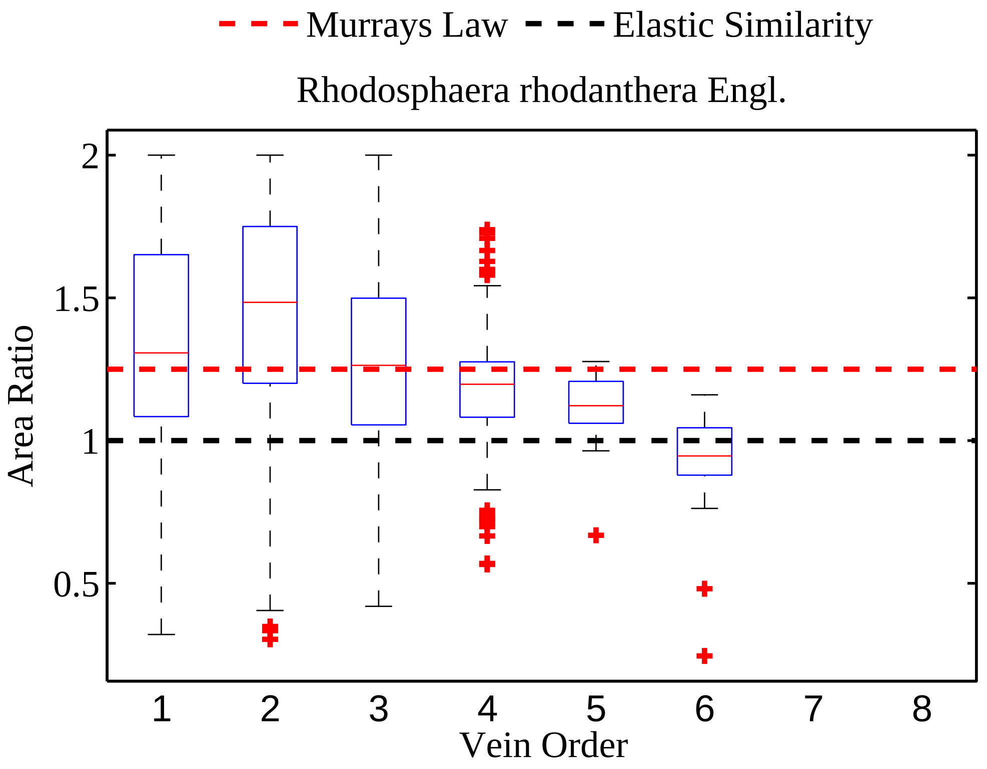
Murrays Law
Elastic Similarity
Rhodosphaera rhodanthera Engl.
Area Ratio
2
1.5
1
0.5
1
2
3
4
5
6
7
8
Vein Order

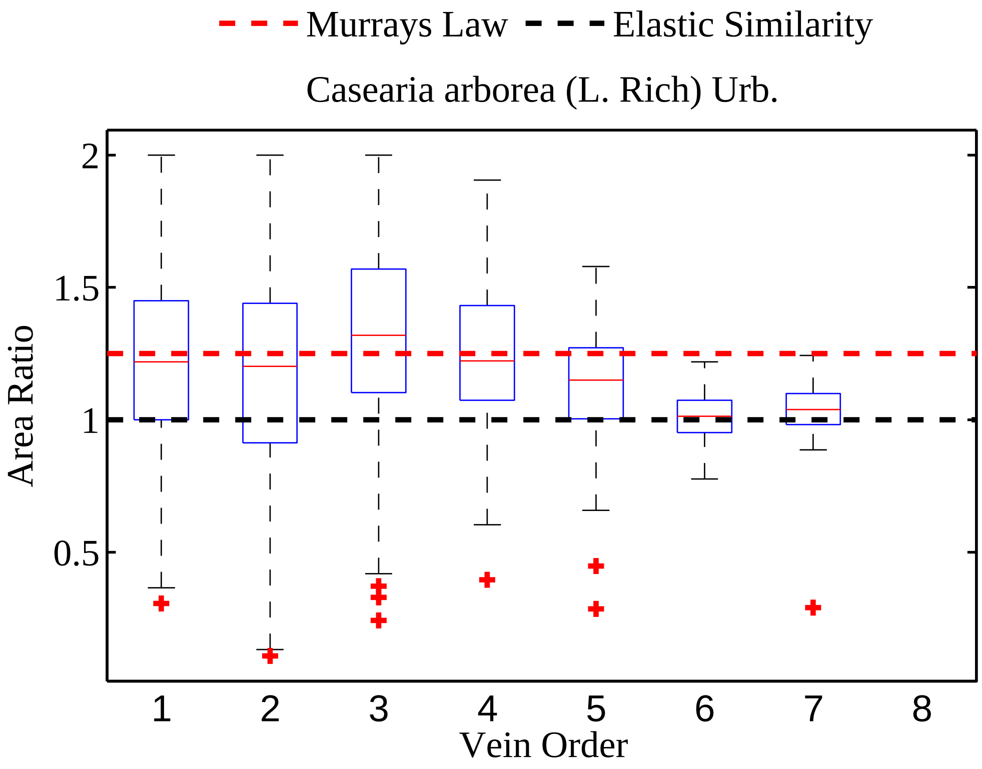

Murrays Law
Elastic Similarity
Casearia arborea (L. Rich) Urb.
Area Ratio
2
1.5
1
0.5
1
2
3
4
5
6
7
8
Vein Order

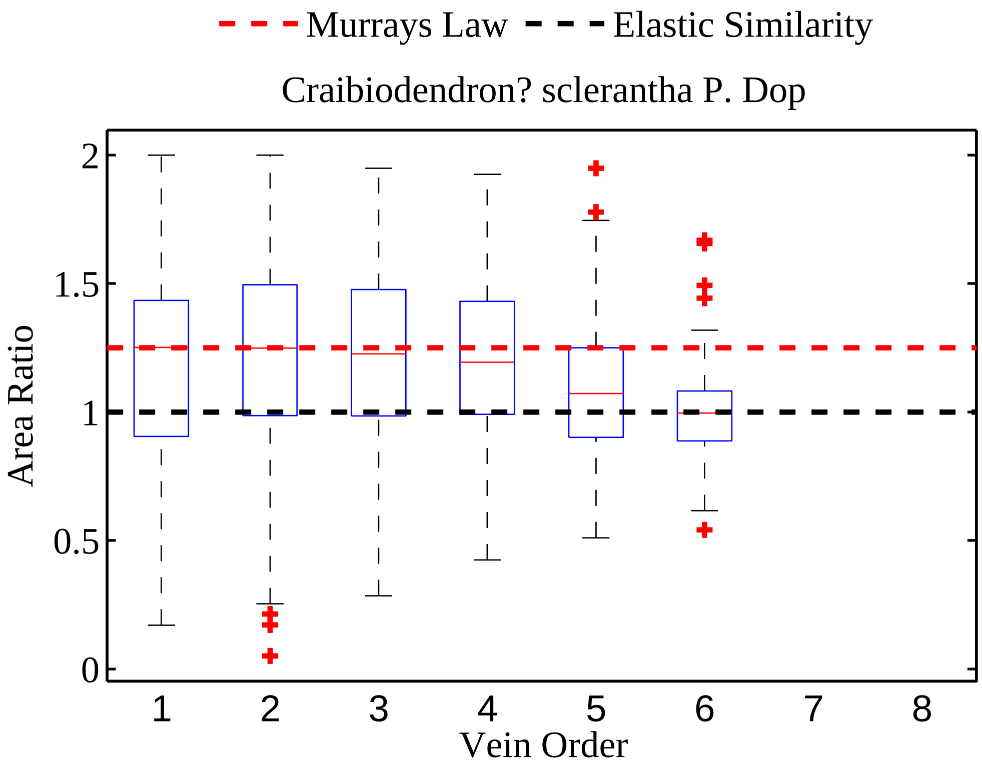

Murrays Law
Elastic Similarity
Craibiodendron? sclerantha P. Dop
Area Ratio
Vein Order
0
0.5
1
1.5
2
1
2
3
4
5
6
7
8

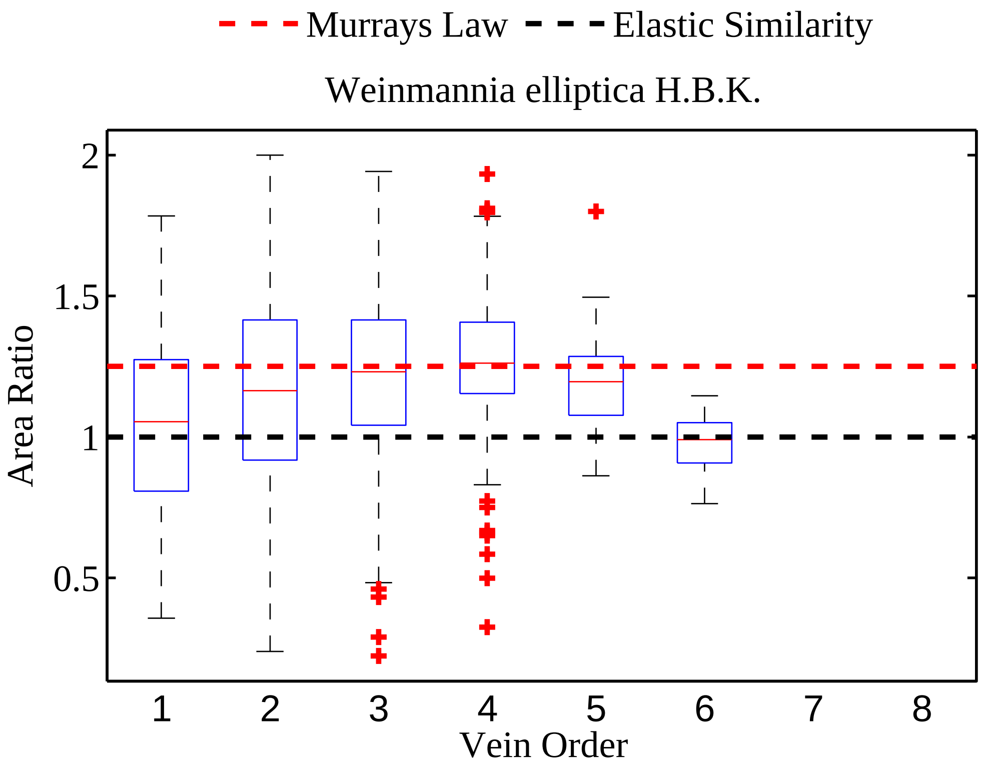
Murrays Law
Elastic Similarity
Weinmannia elliptica H.B.K.
Area Ratio
2
1.5
1
0.5
1
2
3
4
5
6
7
8
Vein Order

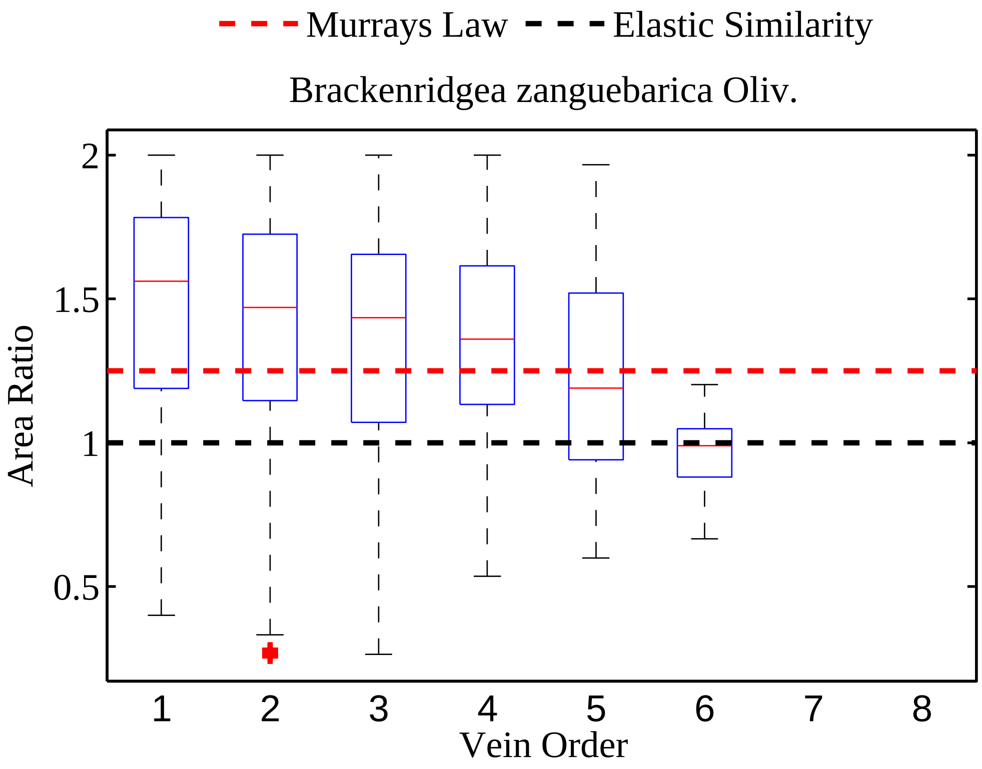
Murrays Law
Elastic Similarity
Brackenridgea zanguebarica Oliv.
Area Ratio
2
1.5
1
0.5
1
2
3
4
5
6
7
8
Vein Order

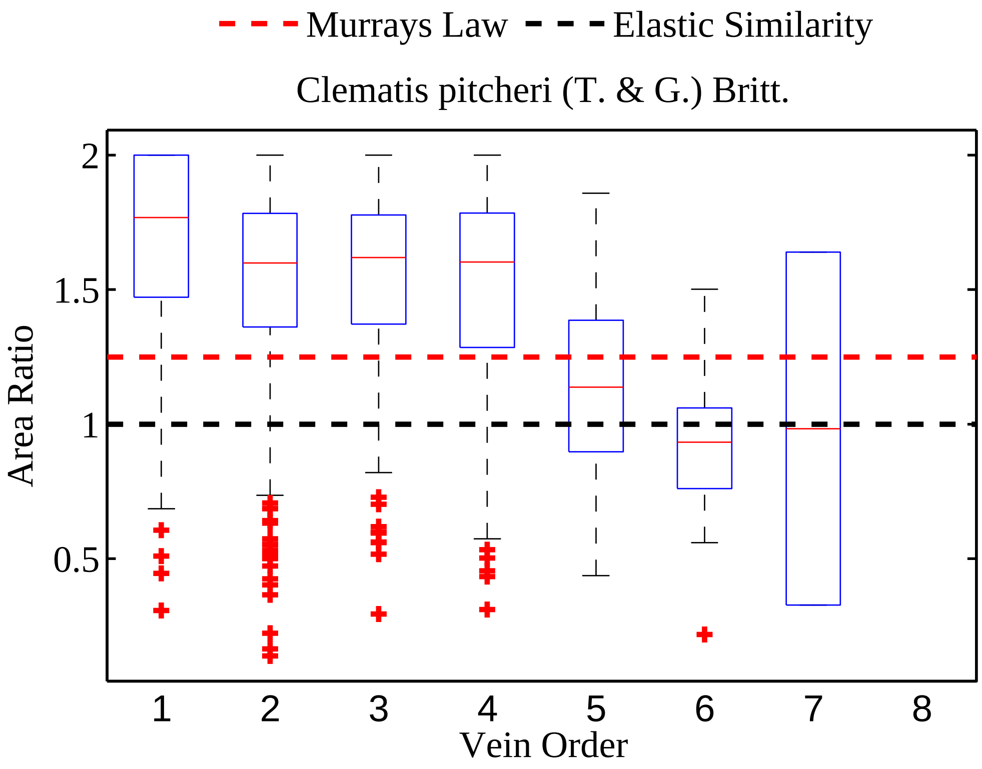

Murrays Law
Elastic Similarity
Clematis pitcheri (T. & G.) Britt.
Area Ratio
2
1.5
1
0.5
1
2
3
4
5
6
7
8
Vein Order

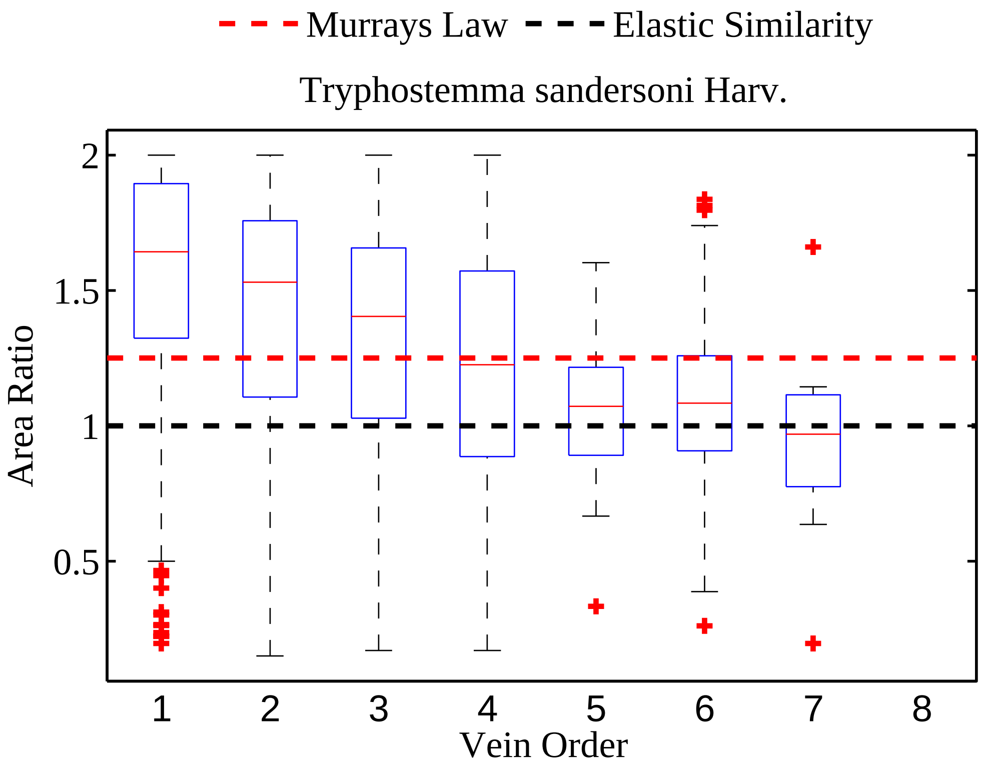

Murrays Law
Elastic Similarity
Tryphostemma sandersoni Harv.
Area Ratio
2
1.5
1
0.5
1
2
3
4
5
6
7
8
Vein Order

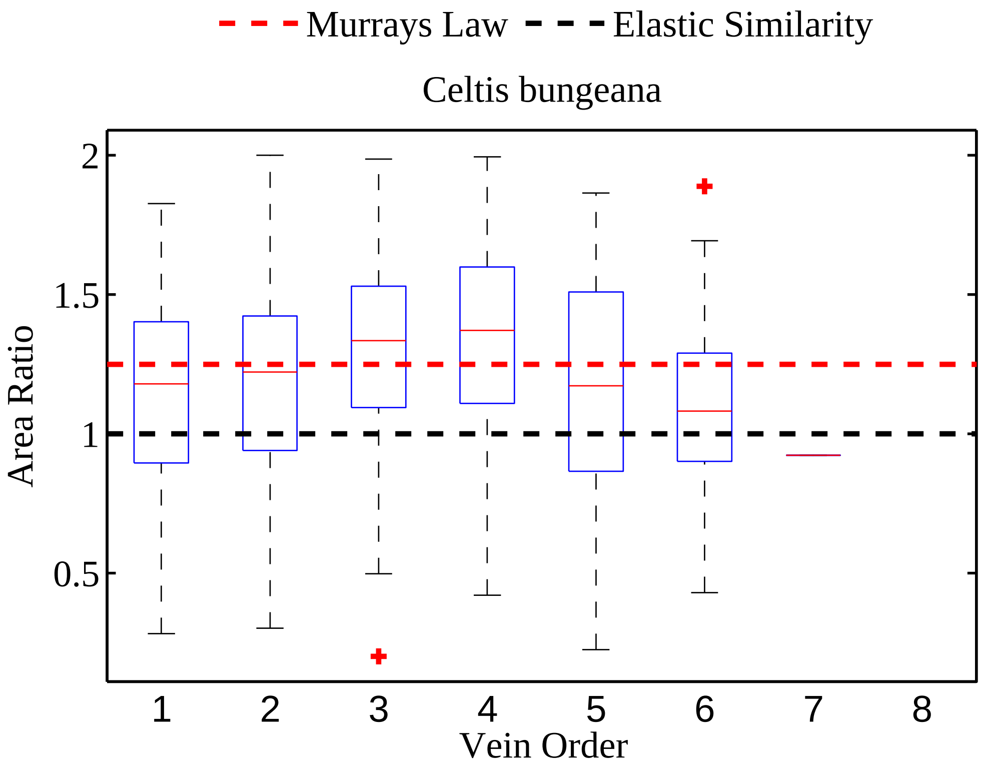

Murrays Law
Elastic Similarity
Celtis bungeana
Area Ratio
2
1.5
1
0.5
1
2
3
4
5
6
7
8
Vein Order

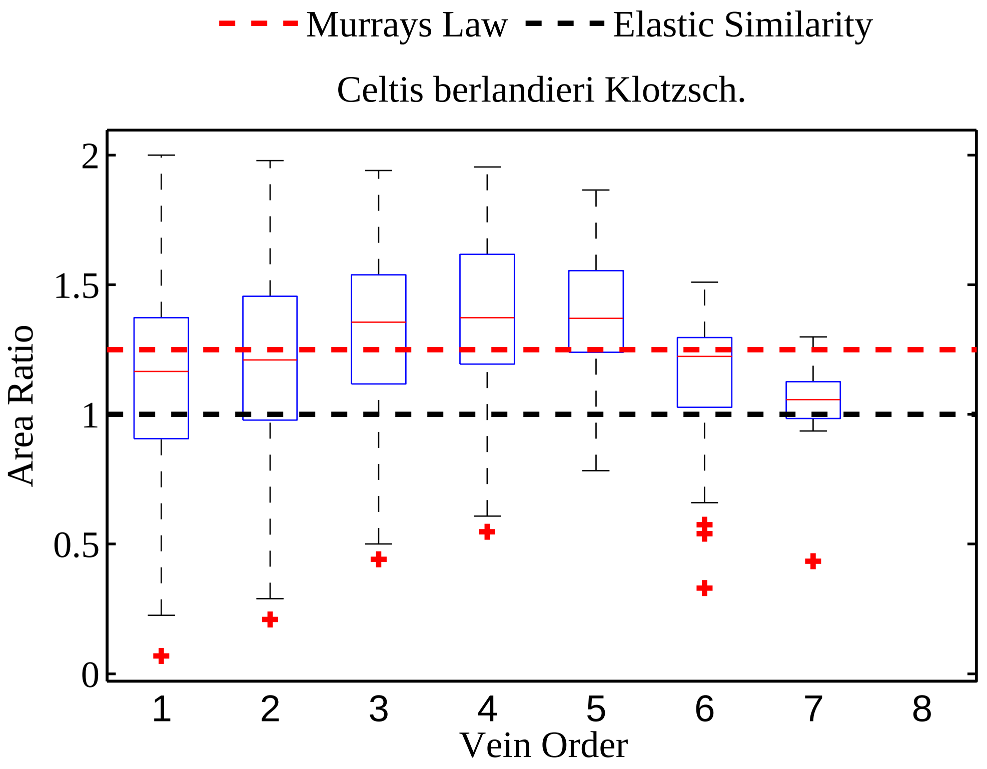

Murrays Law
Elastic Similarity
Celtis berlandieri Klotzsch.
Area Ratio
Vein Order
0
0.5
1
1.5
2
1
2
3
4
5
6
7
8

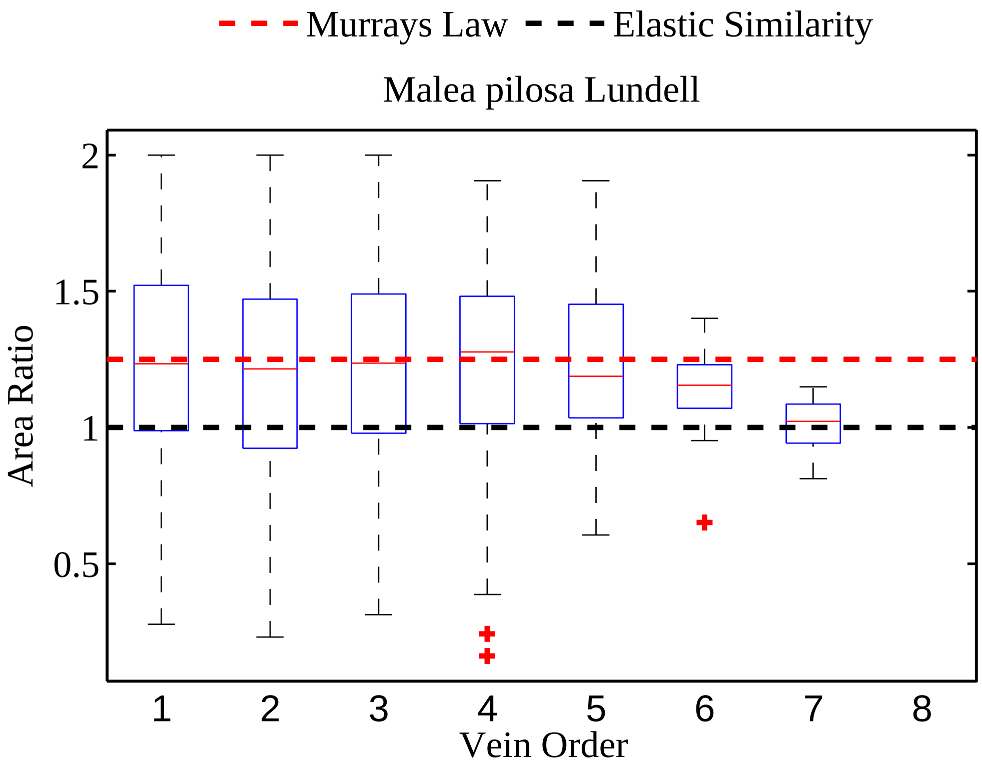
Murrays Law
Elastic Similarity
Malea pilosa Lundell
Area Ratio
2
1.5
1
0.5
1
2
3
4
5
6
7
8
Vein Order

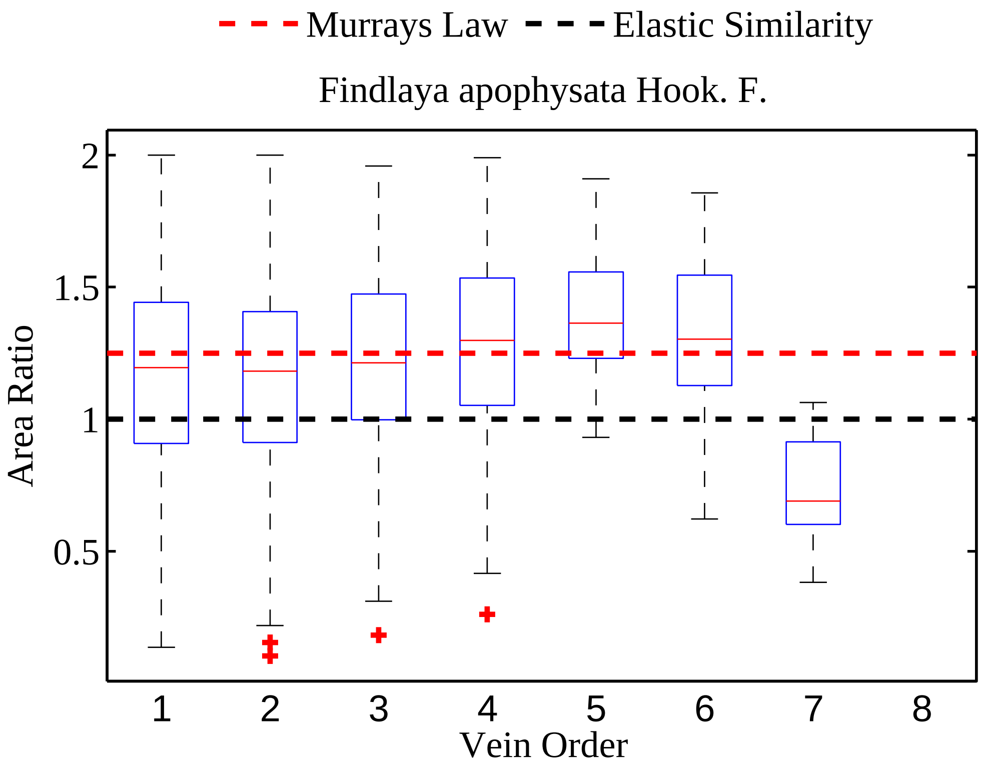

Murrays Law
Elastic Similarity
Findlaya apophysata Hook. F.
Area Ratio
2
1.5
1
0.5
1
2
3
4
5
6
7
8
Vein Order

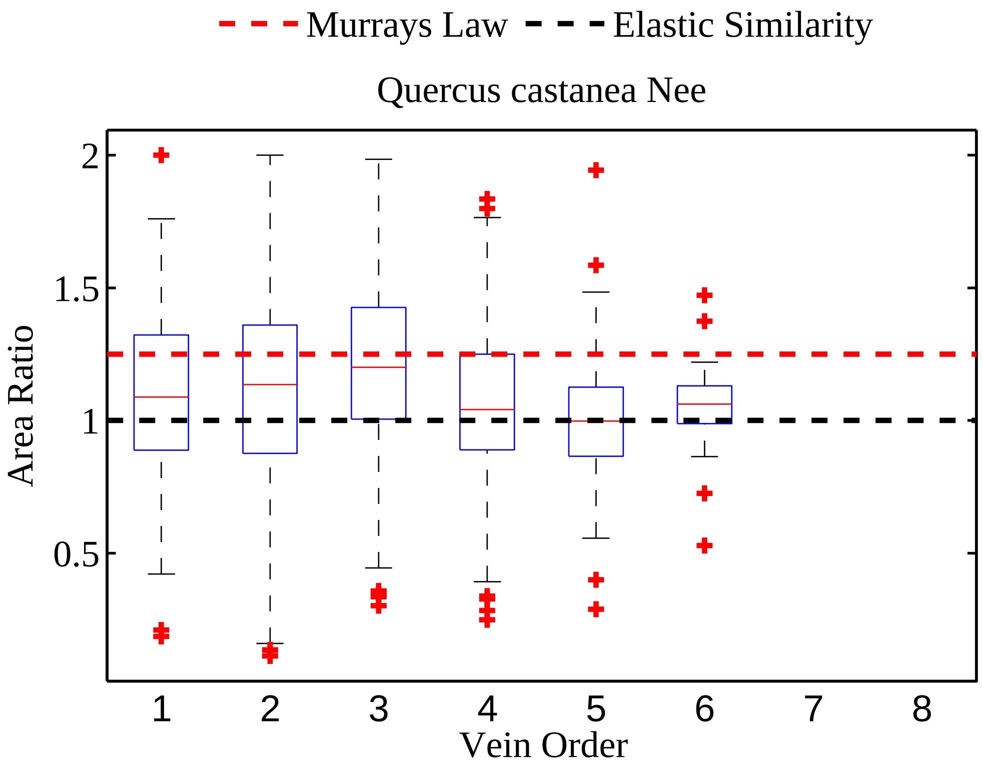

Murrays Law
Elastic Similarity
Quercus castanea Nee
Area Ratio
2
1.5
1
0.5
1
2
3
4
5
6
7
8
Vein Order

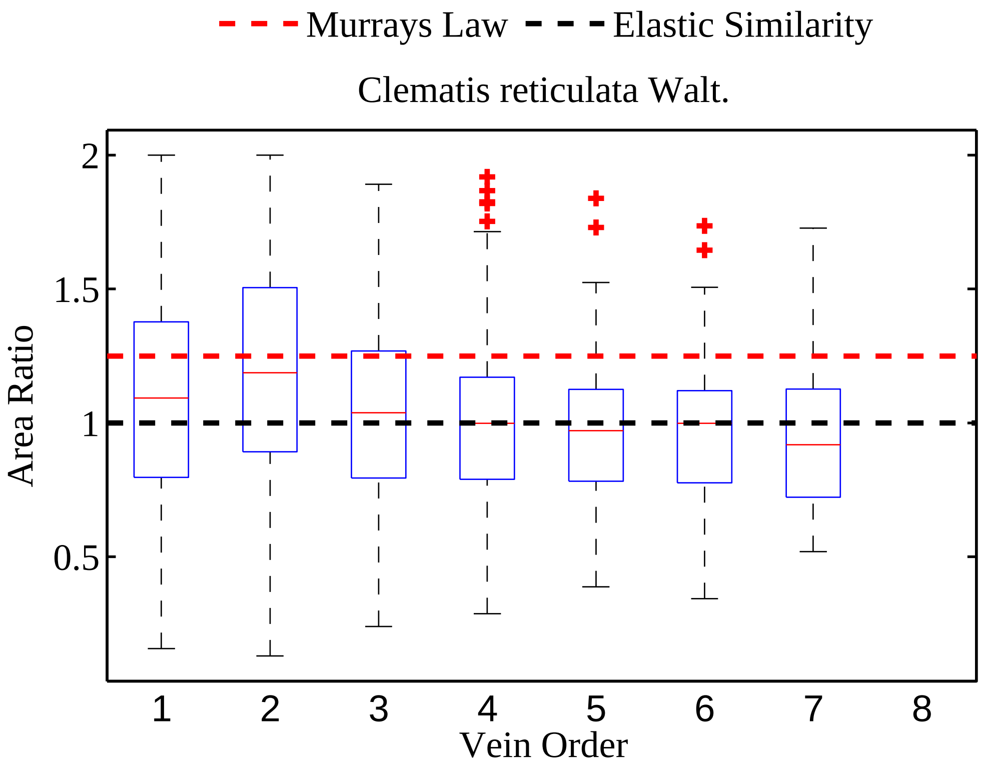

Murrays Law
Elastic Similarity
Clematis reticulata Walt.
Area Ratio
2
1.5
1
0.5
1
2
3
4
5
6
7
8
Vein Order

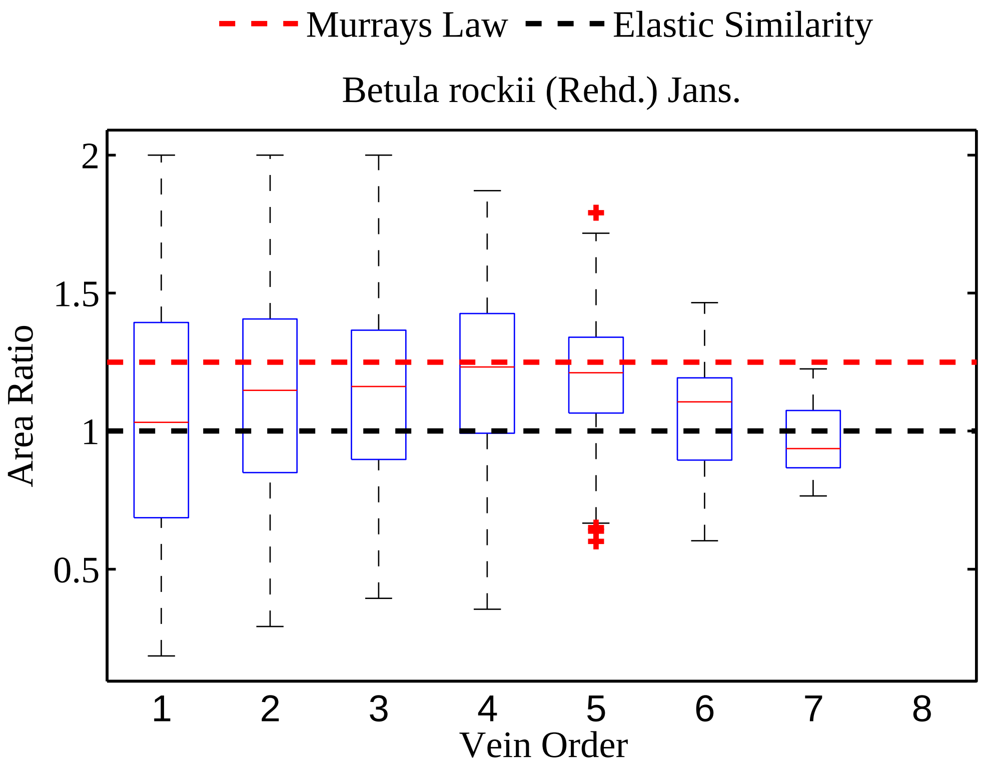

Murrays Law
Elastic Similarity
Betula rockii (Rehd.) Jans.
Area Ratio
2
1.5
1
0.5
1
2
3
4
5
6
7
8
Vein Order

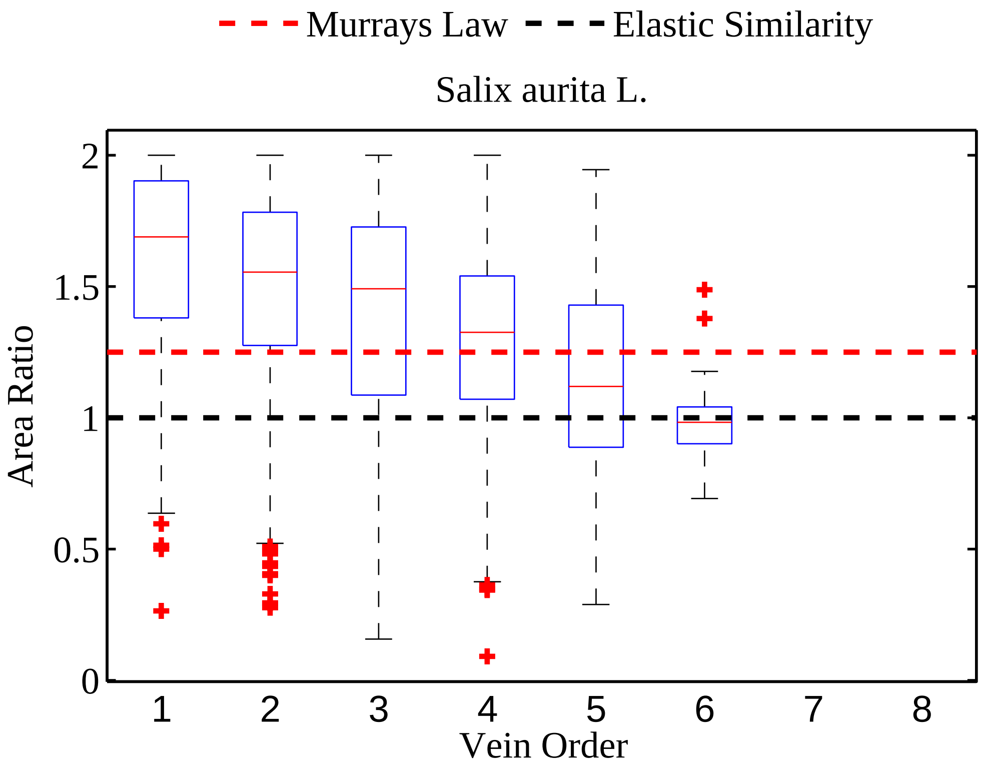

Murrays Law
Elastic Similarity
Salix aurita L.
Area Ratio
Vein Order
0
0.5
1
1.5
2
1
2
3
4
5
6
7
8

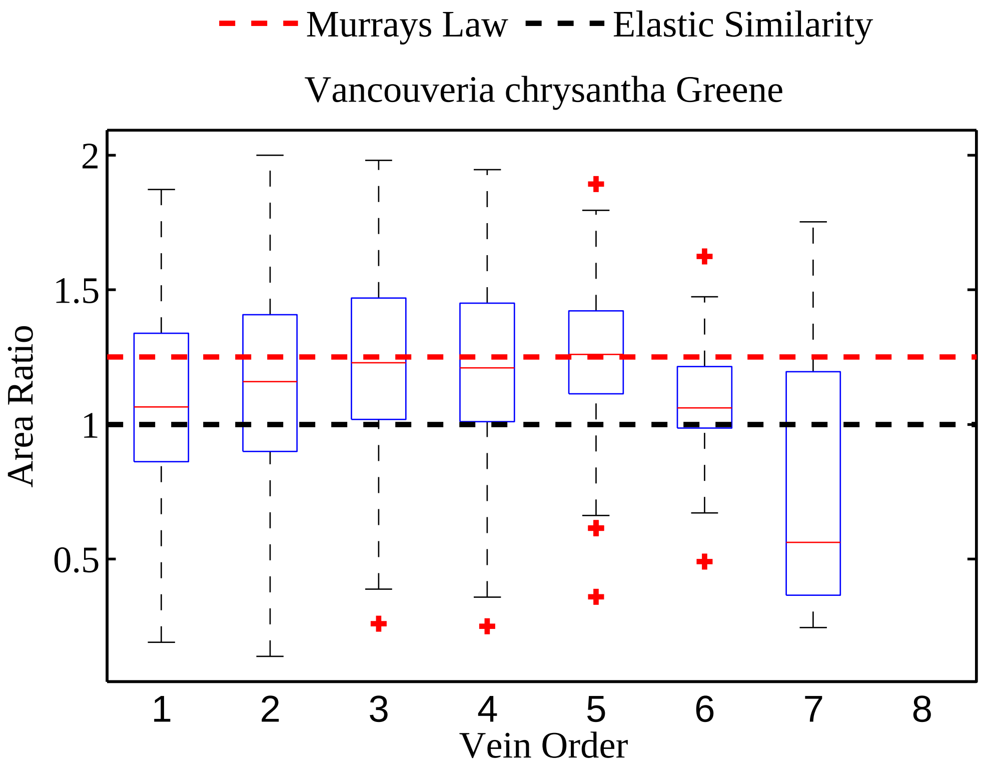

Murrays Law
Elastic Similarity
Vancouveria chrysantha Greene
Area Ratio
2
1.5
1
0.5
1
2
3
4
5
6
7
8
Vein Order

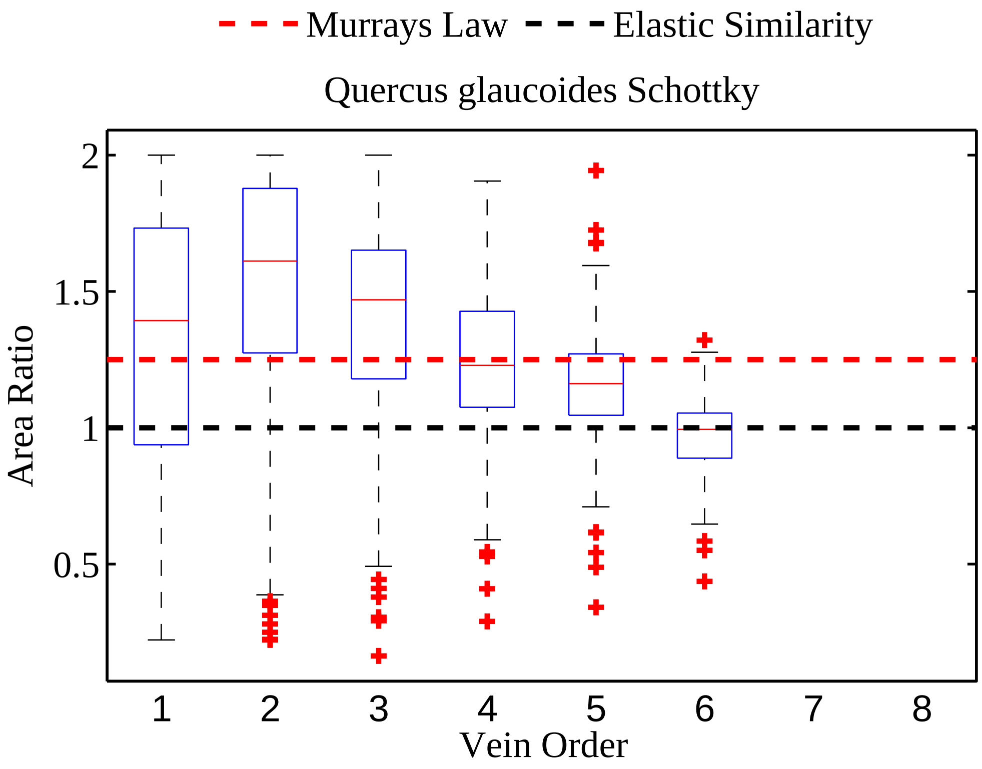

Murrays Law
Elastic Similarity
Quercus glaucoides Schottky
Area Ratio
2
1.5
1
0.5
1
2
3
4
5
6
7
8
Vein Order

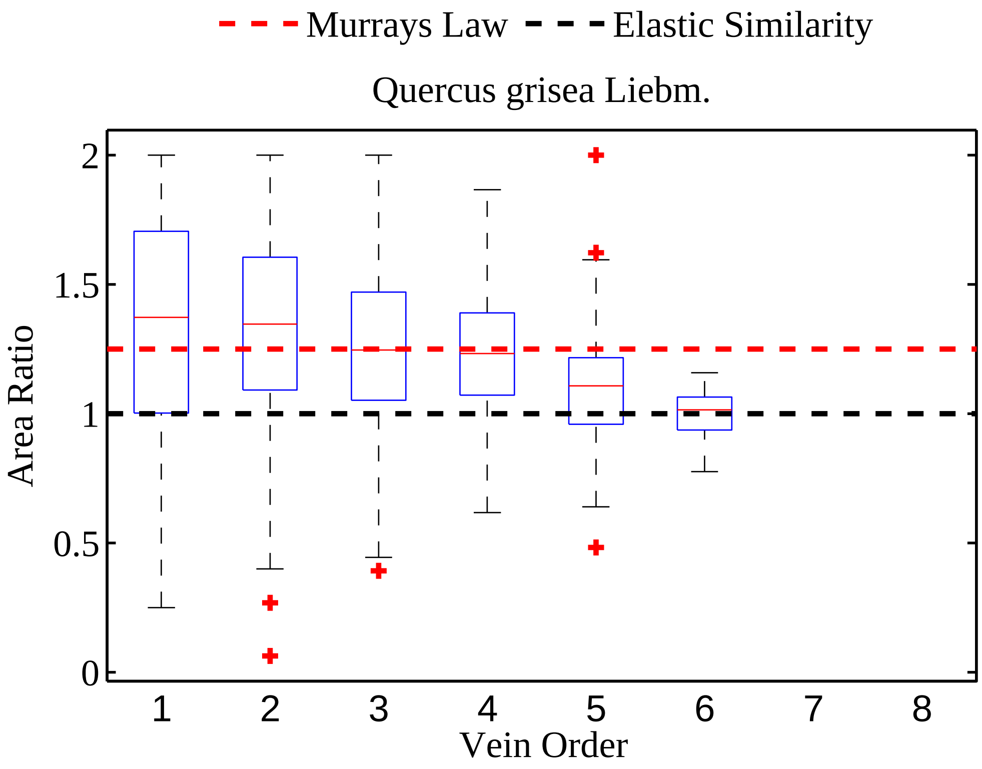

Murrays Law
Elastic Similarity
Quercus grisea Liebm.
Area Ratio
Vein Order
0
0.5
1
1.5
2
1
2
3
4
5
6
7
8

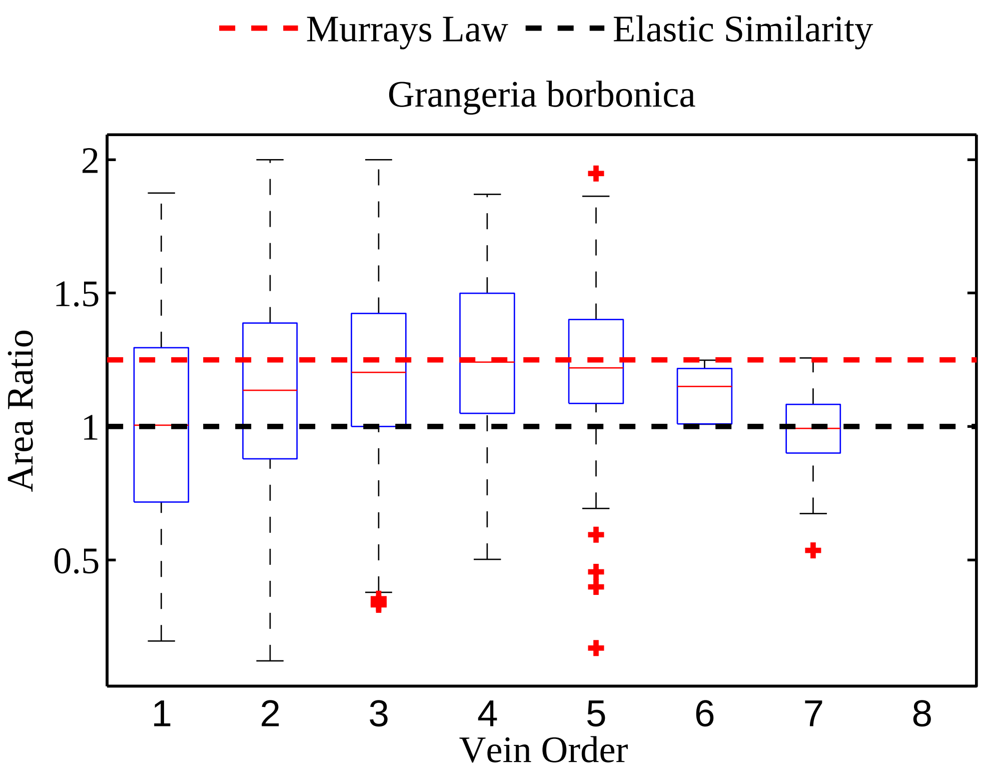
Murrays Law
Elastic Similarity
Grangeria borbonica
Area Ratio
2
1.5
1
0.5
1
2
3
4
5
6
7
8
Vein Order

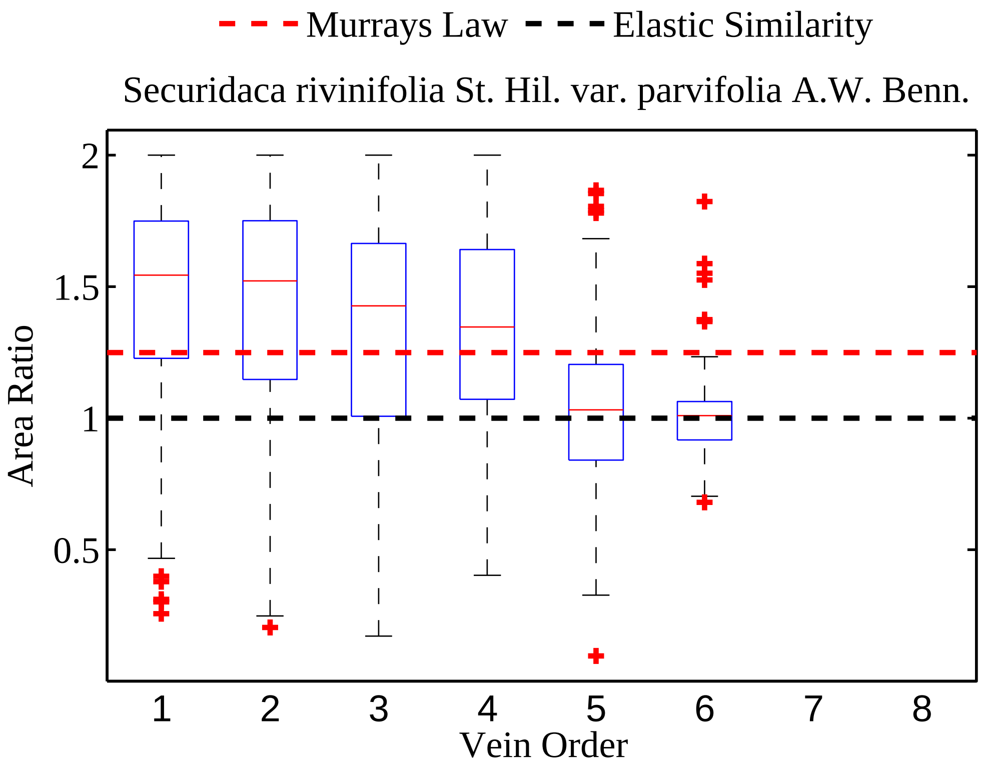

Murrays Law
Elastic Similarity
Securidaca rivinifolia St. Hil. var. parvifolia A.W. Benn.
Area Ratio
2
1.5
1
0.5
1
2
3
4
5
6
7
8
Vein Order

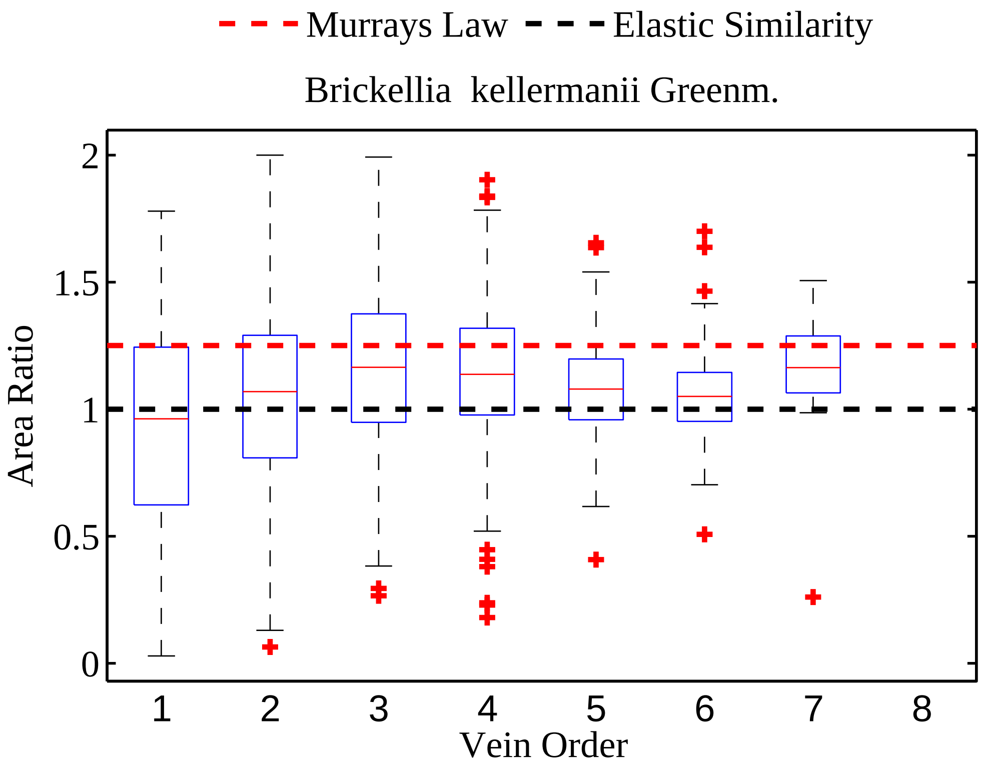

Murrays Law
Elastic Similarity
Brickellia kellermanii Greenm.
Area Ratio
0
0.5
1
1.5
2
1
2
3
4
5
6
7
8
Vein Order

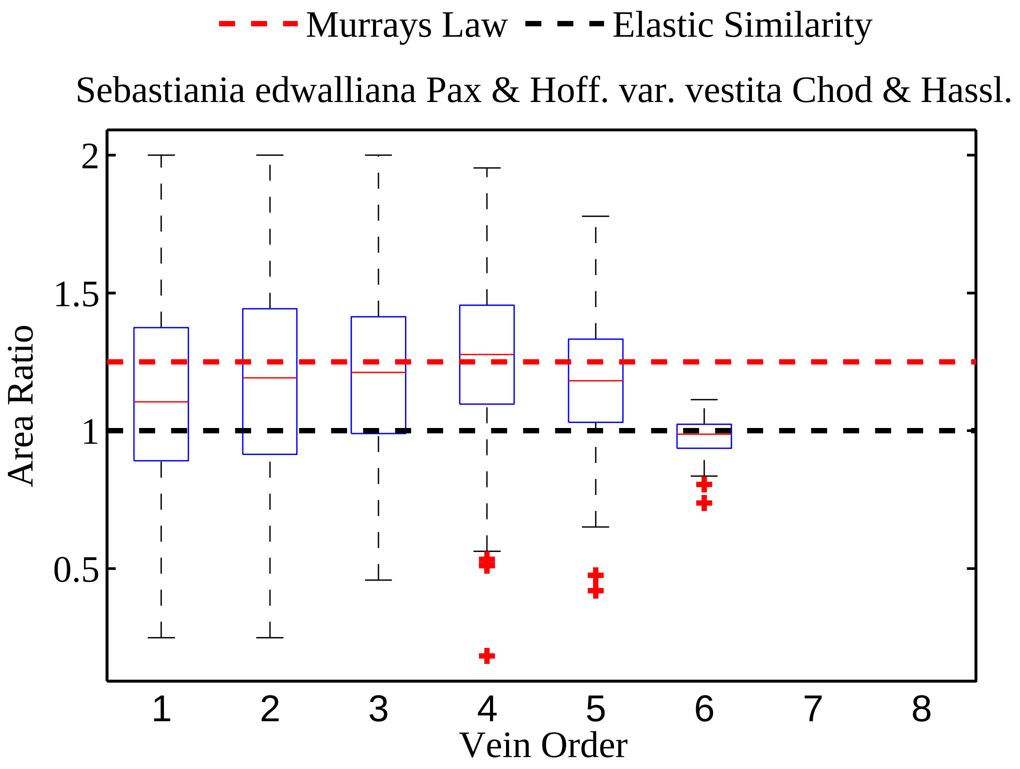

Murrays Law
Elastic Similarity
Sebastiania edwalliana Pax & Hoff. var. vestita Chod & Hassl.
Area Ratio
2
1.5
1
0.5
1
2
3
4
5
6
7
8
Vein Order

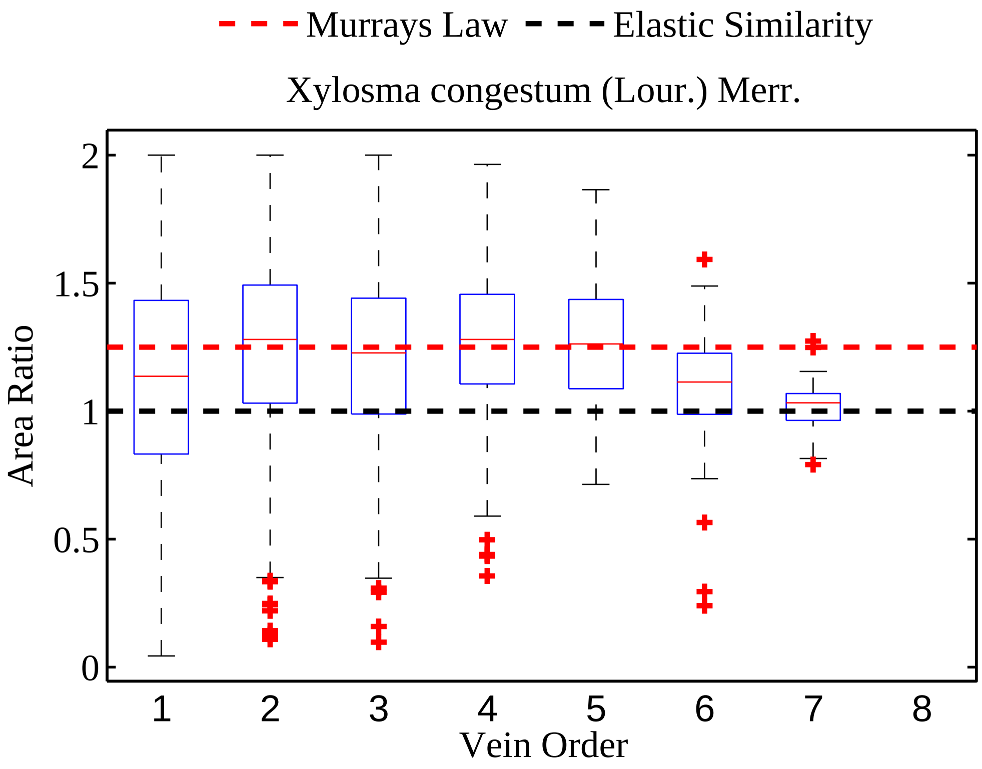

Murrays Law
Elastic Similarity
Xylosma congestum (Lour.) Merr.
Area Ratio
Vein Order
0
0.5
1
1.5
2
1
2
3
4
5
6
7
8

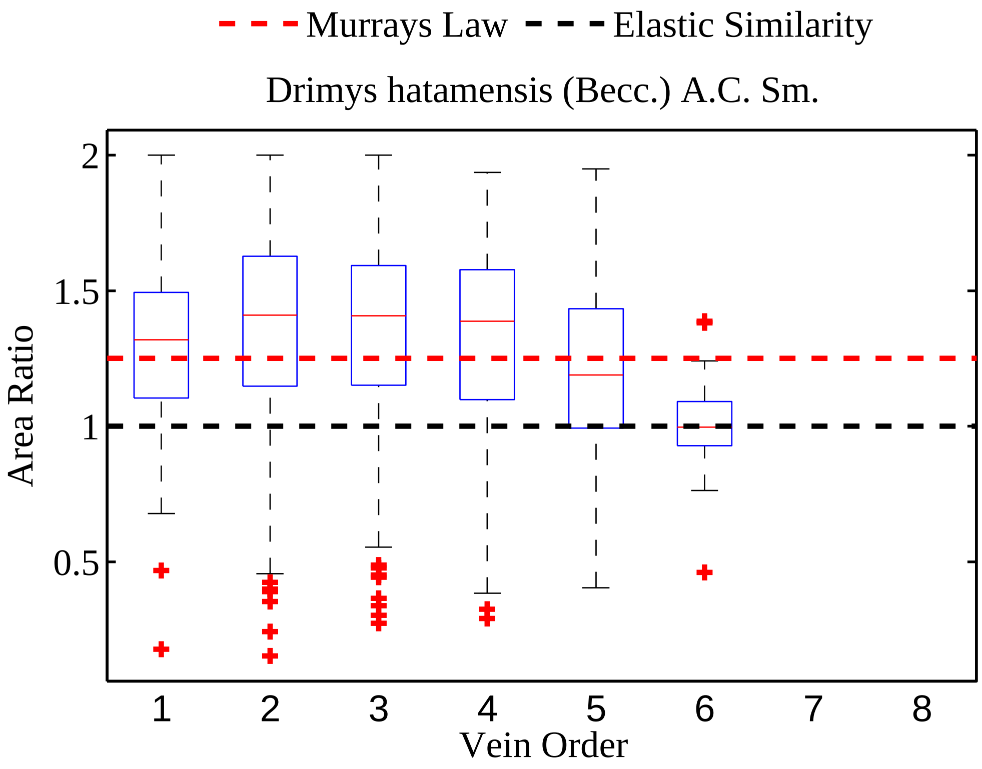

Murrays Law
Elastic Similarity
Drimys hatamensis (Becc.) A.C. Sm.
Area Ratio
2
1.5
1
0.5
1
2
3
4
5
6
7
8
Vein Order

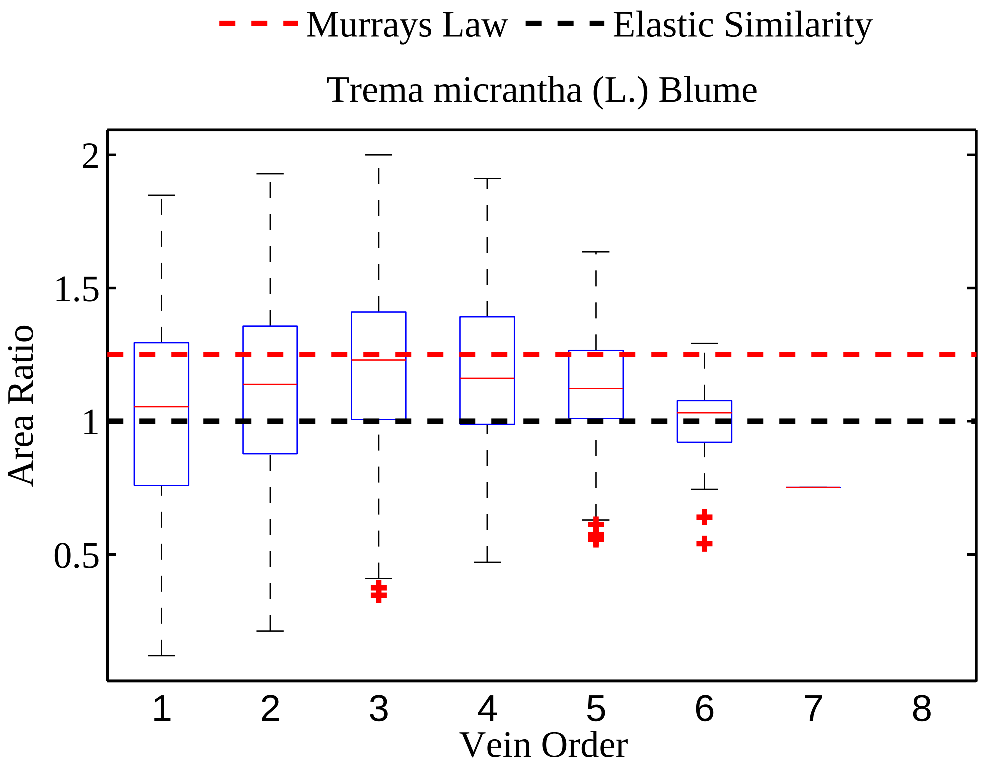

Murrays Law
Elastic Similarity
Trema micrantha (L.) Blume
Area Ratio
2
1.5
1
0.5
1
2
3
4
5
6
7
8
Vein Order

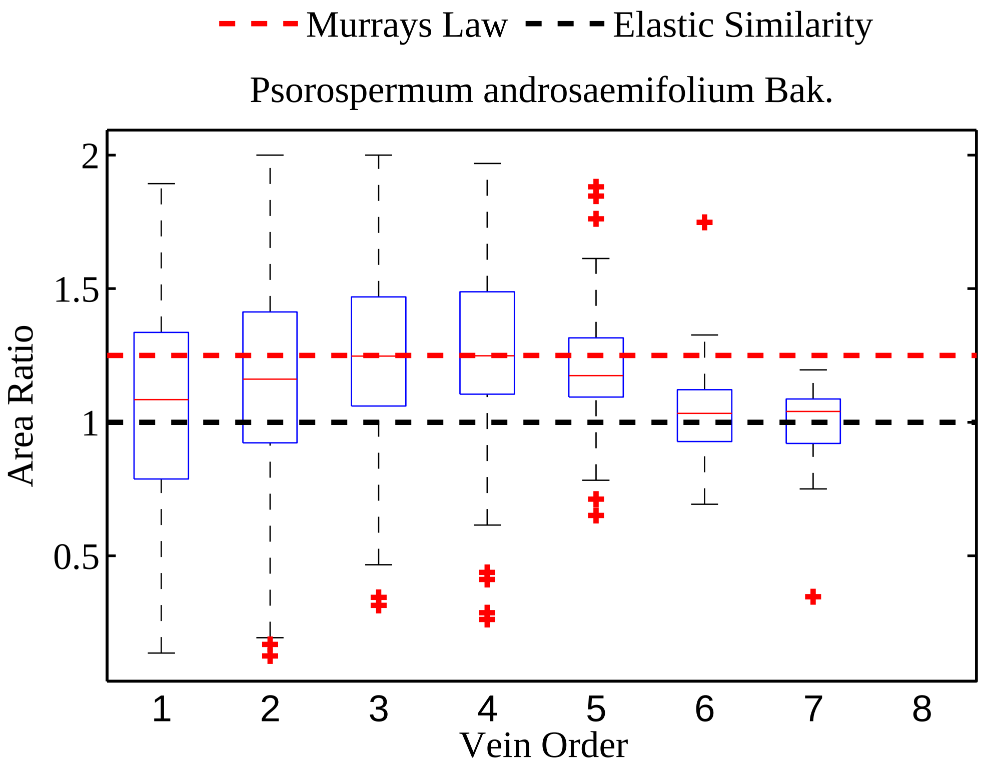

Murrays Law
Elastic Similarity
Psorospermum androsaemifolium Bak.
Area Ratio
2
1.5
1
0.5
1
2
3
4
5
6
7
8
Vein Order

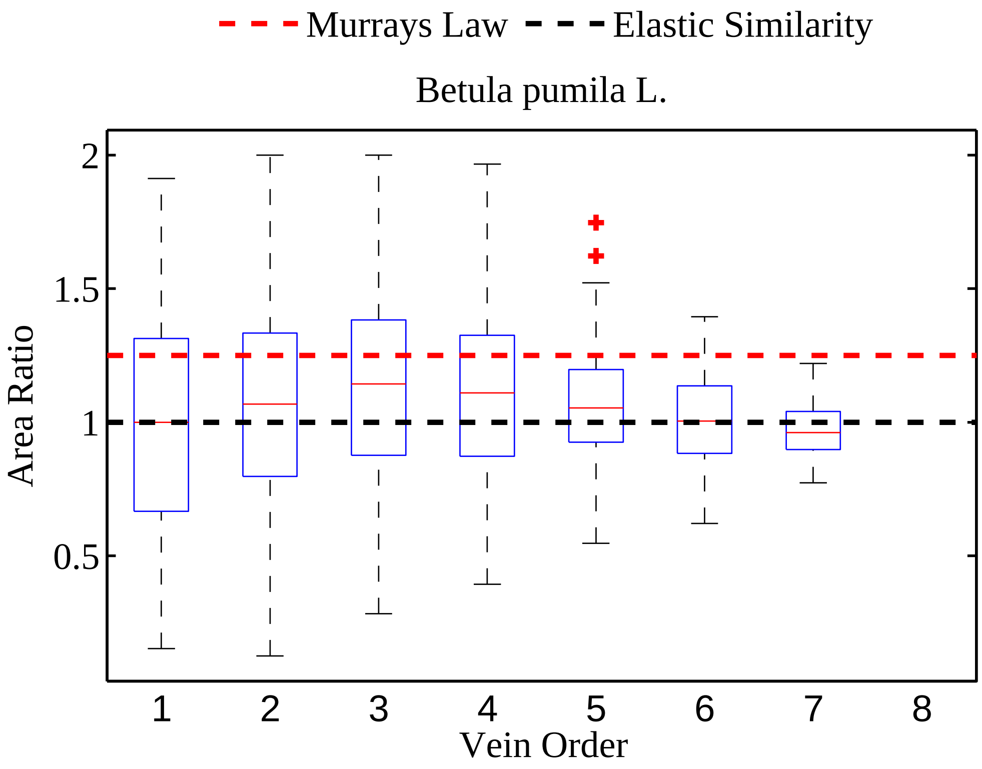
Murrays Law
Elastic Similarity
Betula pumila L.
Area Ratio
2
1.5
1
0.5
1
2
3
4
5
6
7
8
Vein Order

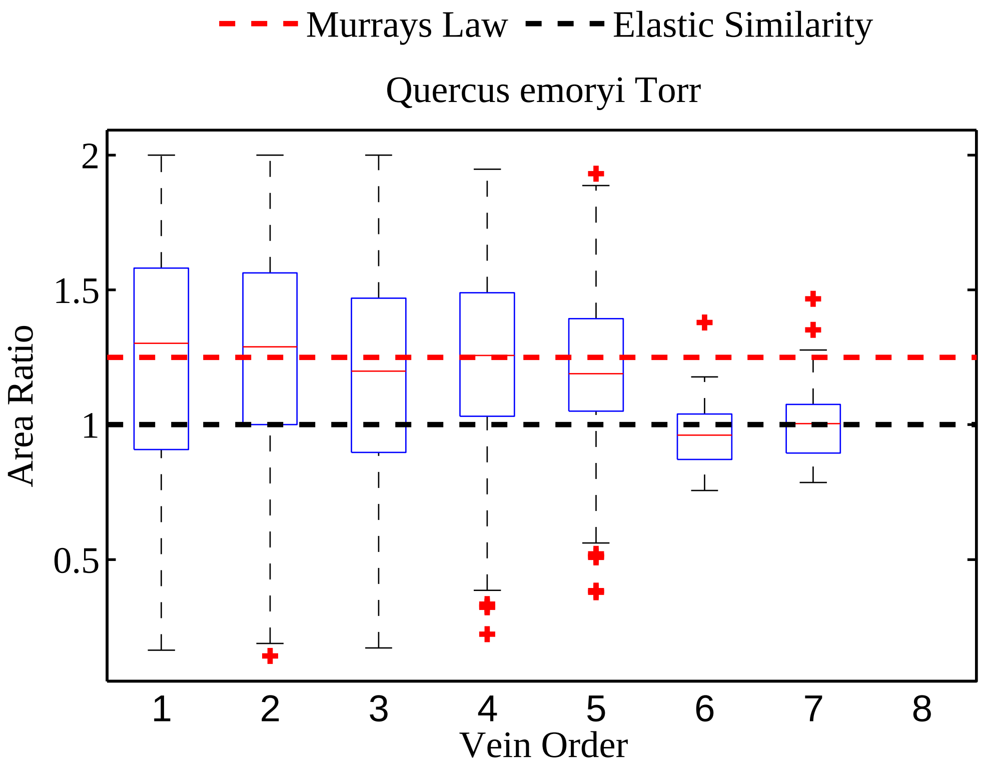

Murrays Law
Elastic Similarity
Quercus emoryi Torr
Area Ratio
2
1.5
1
0.5
1
2
3
4
5
6
7
8
Vein Order

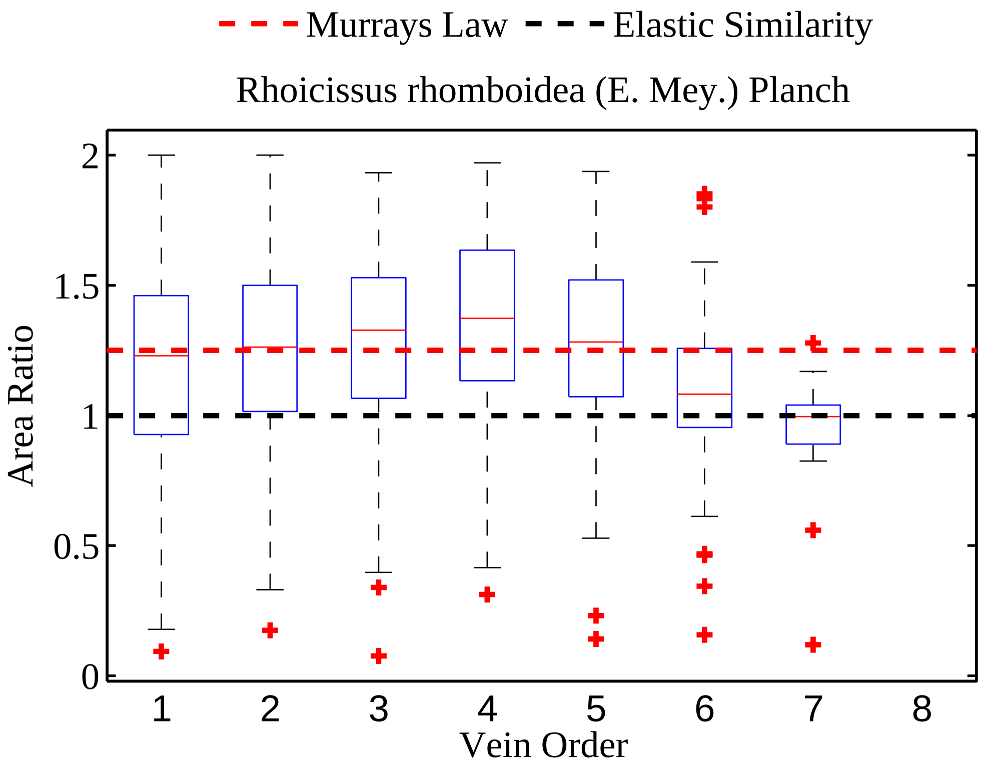
Murrays Law
Elastic Similarity
Rhoicissus rhomboidea (E. Mey.) Planch
Area Ratio
0
0.5
1
1.5
2
1
2
3
4
5
6
7
8
Vein Order

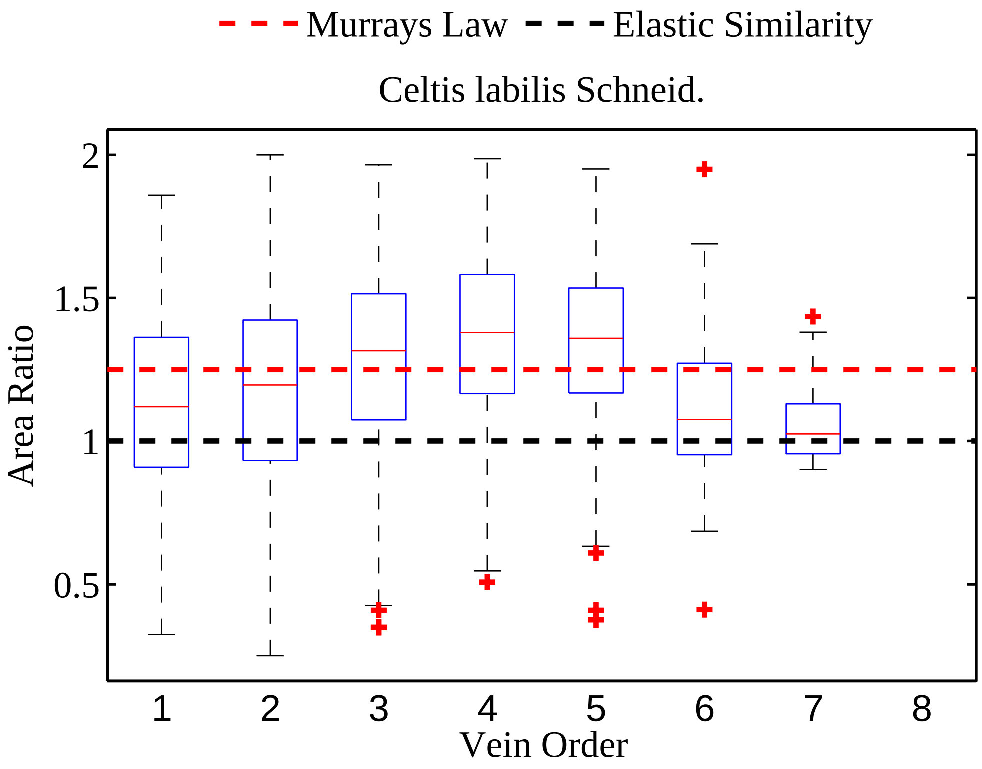

Murrays Law
Elastic Similarity
Celtis labilis Schneid.
Area Ratio
2
1.5
1
0.5
1
2
3
4
5
6
7
8
Vein Order

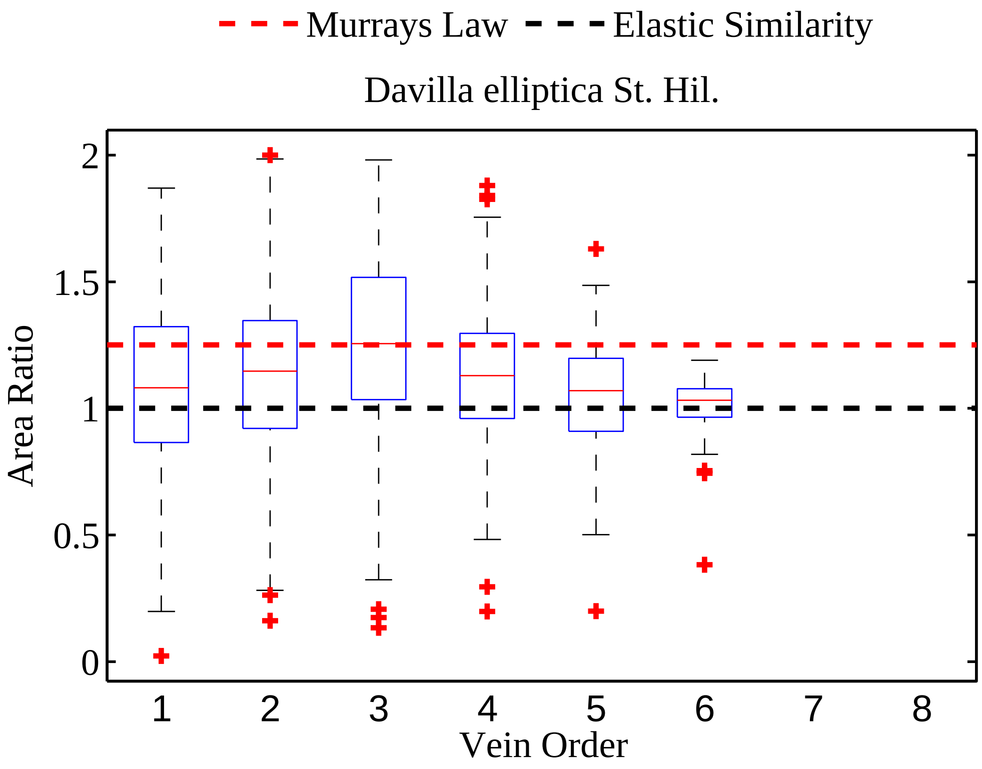
Murrays Law
Elastic Similarity
Davilla elliptica St. Hil.
Area Ratio
Vein Order
0
0.5
1
1.5
2
1
2
3
4
5
6
7
8

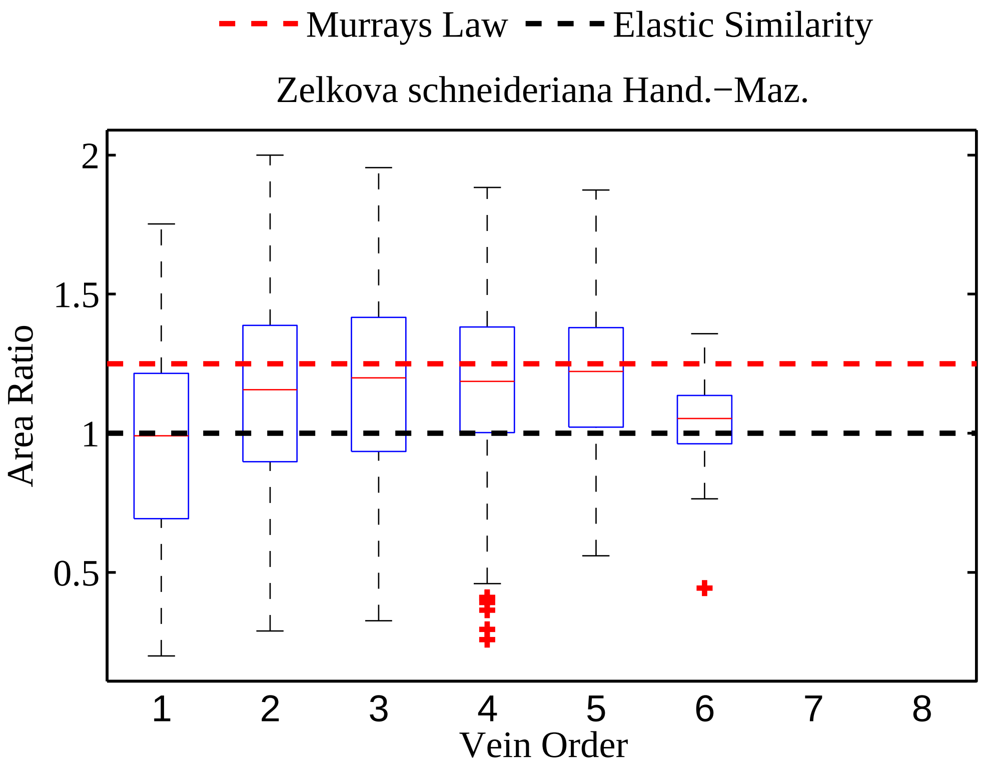

Murrays Law
Elastic Similarity
Zelkova schneideriana Hand.−Maz.
Area Ratio
2
1.5
1
0.5
1
2
3
4
5
6
7
8
Vein Order

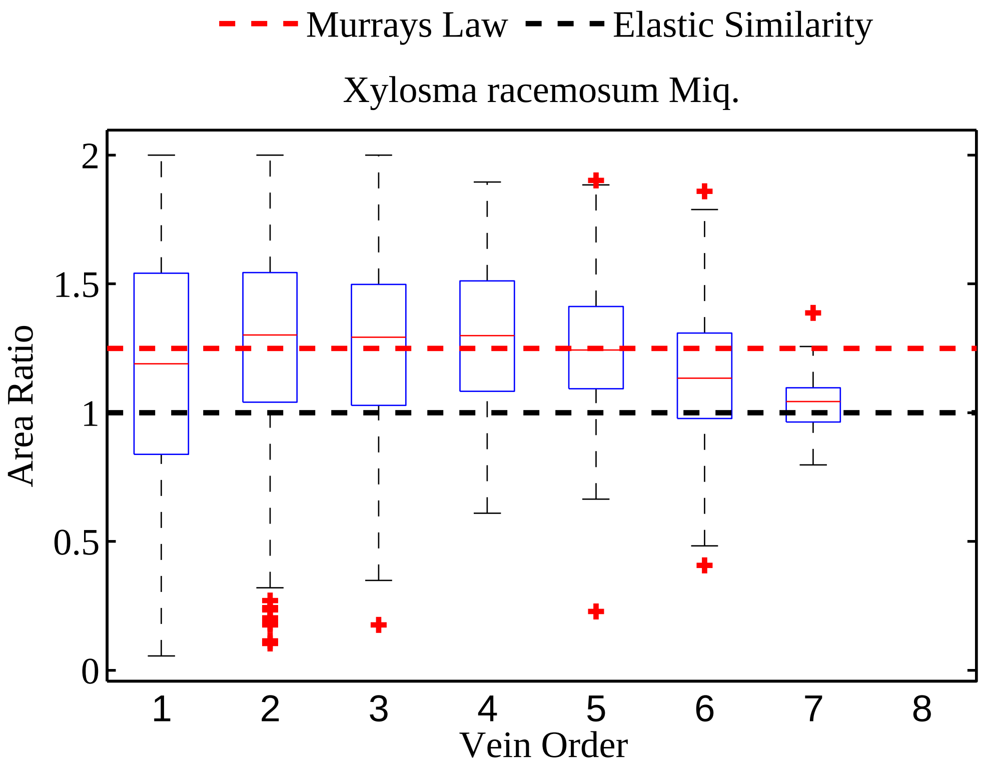
Murrays Law
Elastic Similarity
Xylosma racemosum Miq.
Area Ratio
Vein Order
0
0.5
1
1.5
2
1
2
3
4
5
6
7
8

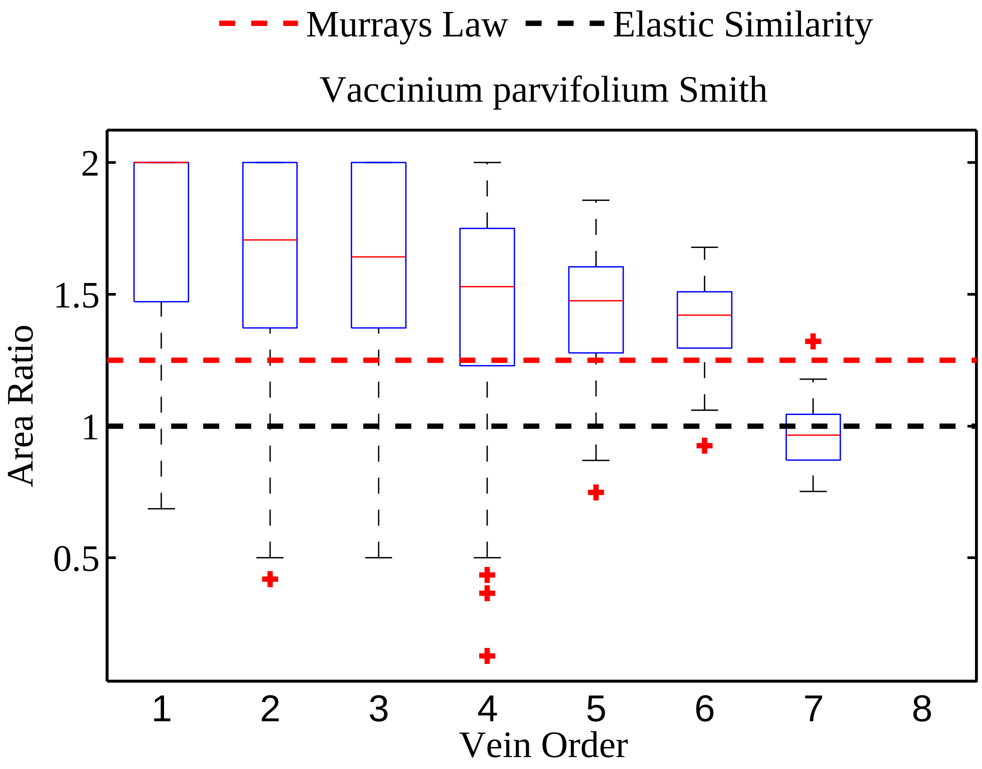

Murrays Law
Elastic Similarity
Vaccinium parvifolium Smith
Area Ratio
2
1.5
1
0.5
1
2
3
4
5
6
7
8
Vein Order

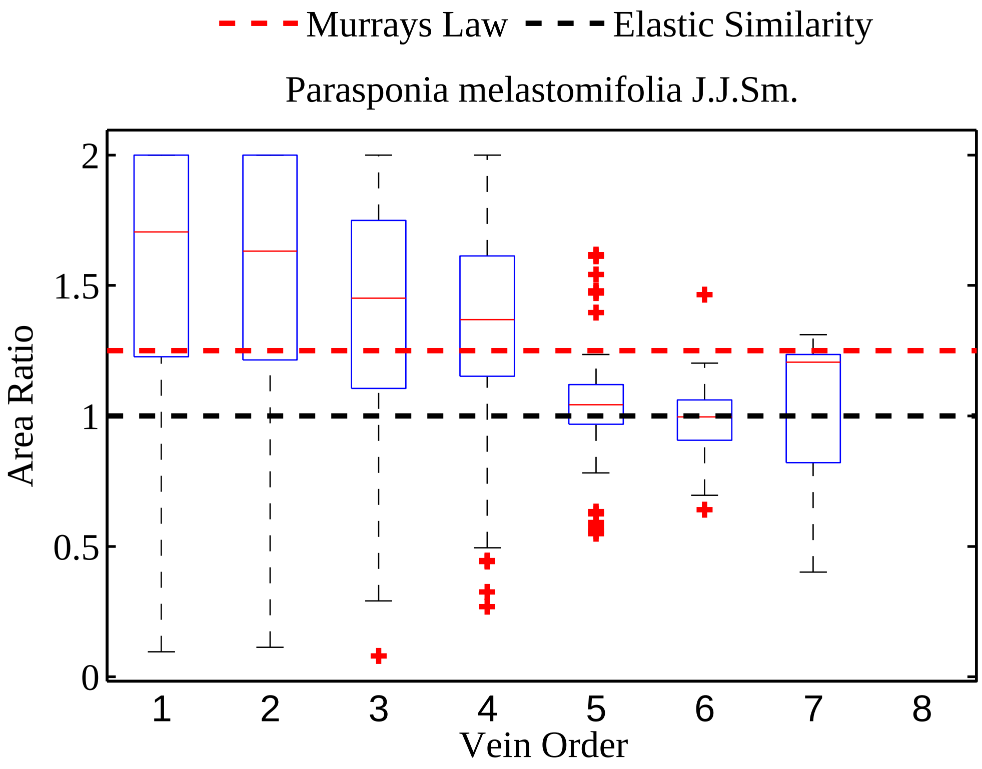

Murrays Law
Elastic Similarity
Parasponia melastomifolia J.J.Sm.
Area Ratio
Vein Order
0
0.5
1
1.5
2
1
2
3
4
5
6
7
8

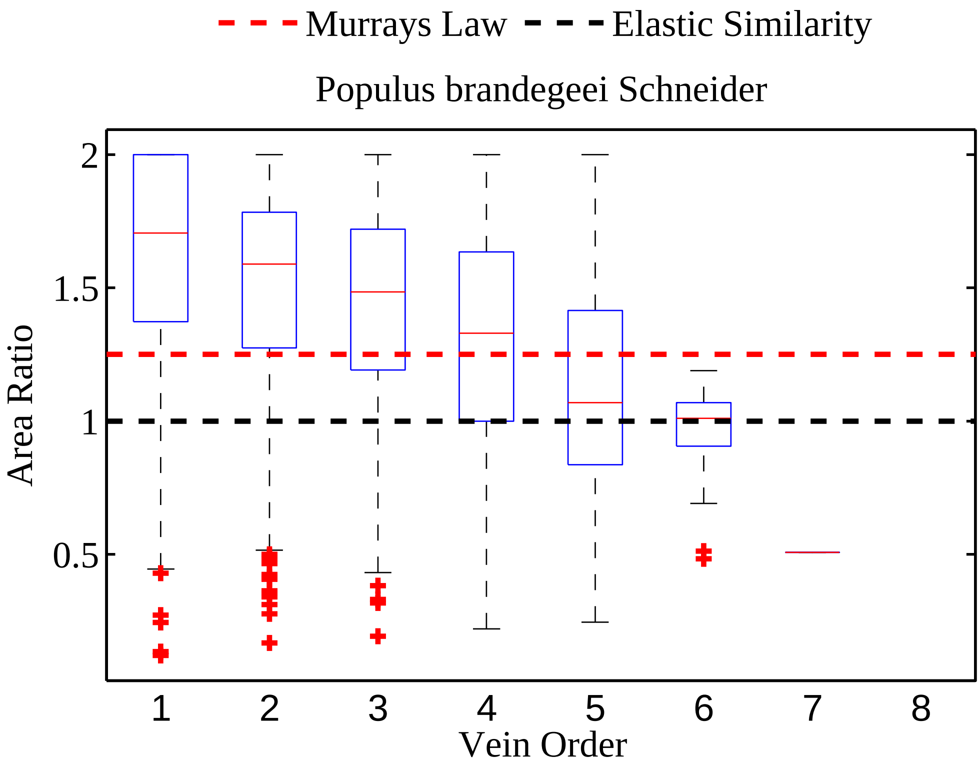

Murrays Law
Elastic Similarity
Populus brandegeei Schneider
Area Ratio
2
1.5
1
0.5
1
2
3
4
5
6
7
8
Vein Order

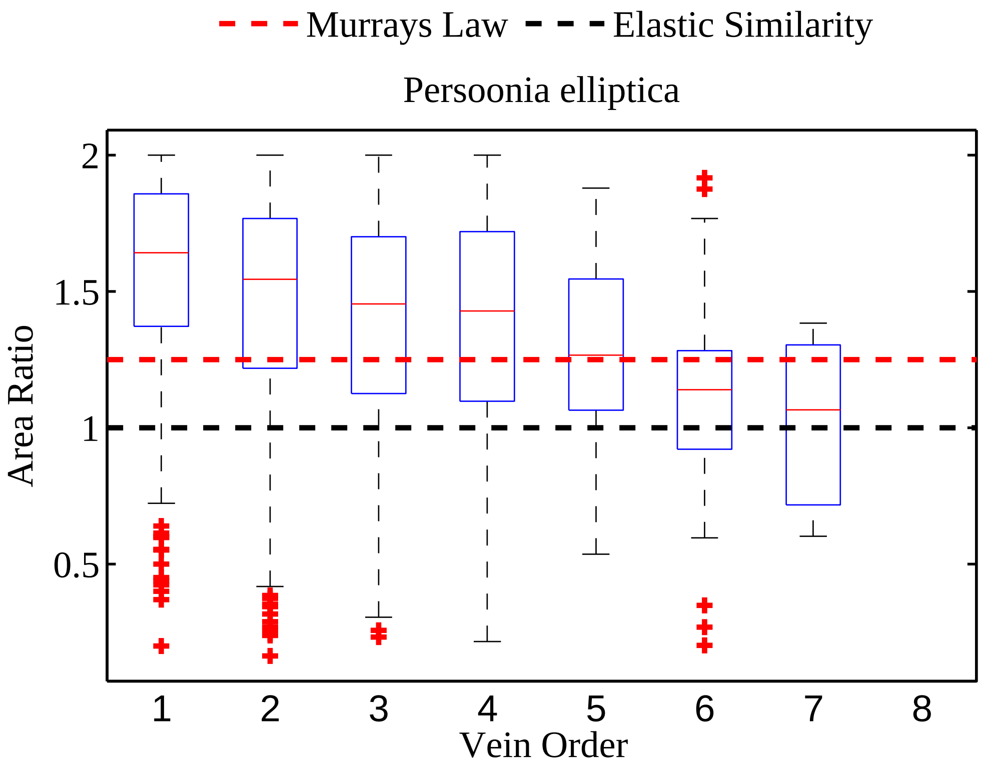

Murrays Law
Elastic Similarity
Persoonia elliptica
Area Ratio
2
1.5
1
0.5
1
2
3
4
5
6
7
8
Vein Order

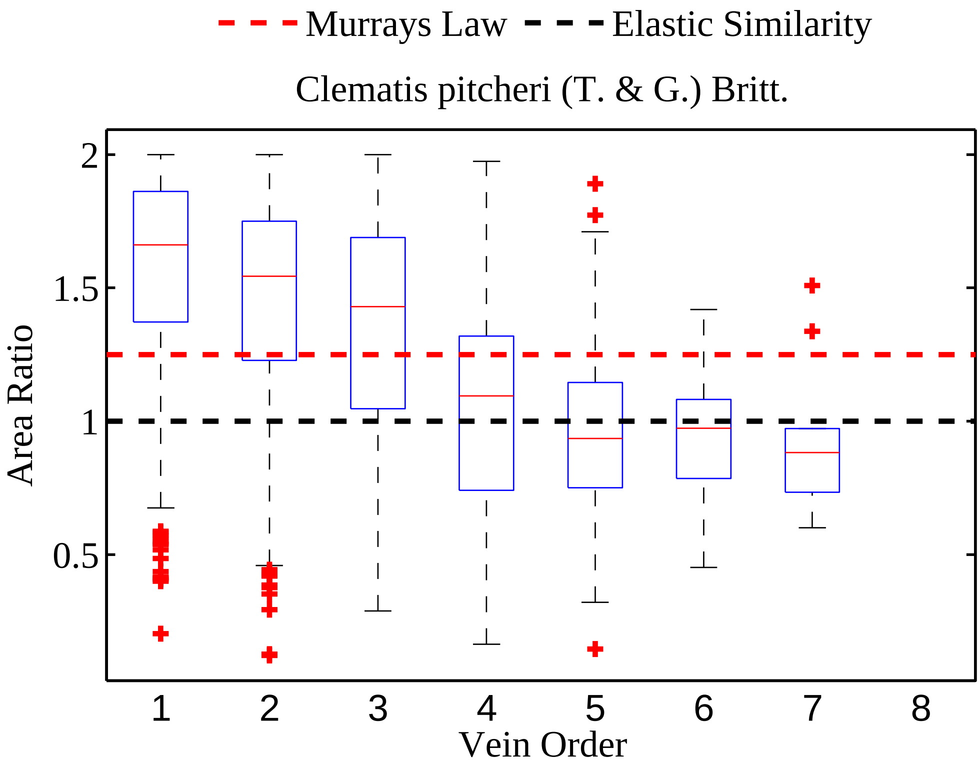

Murrays Law
Elastic Similarity
Clematis pitcheri (T. & G.) Britt.
Area Ratio
2
1.5
1
0.5
1
2
3
4
5
6
7
8
Vein Order

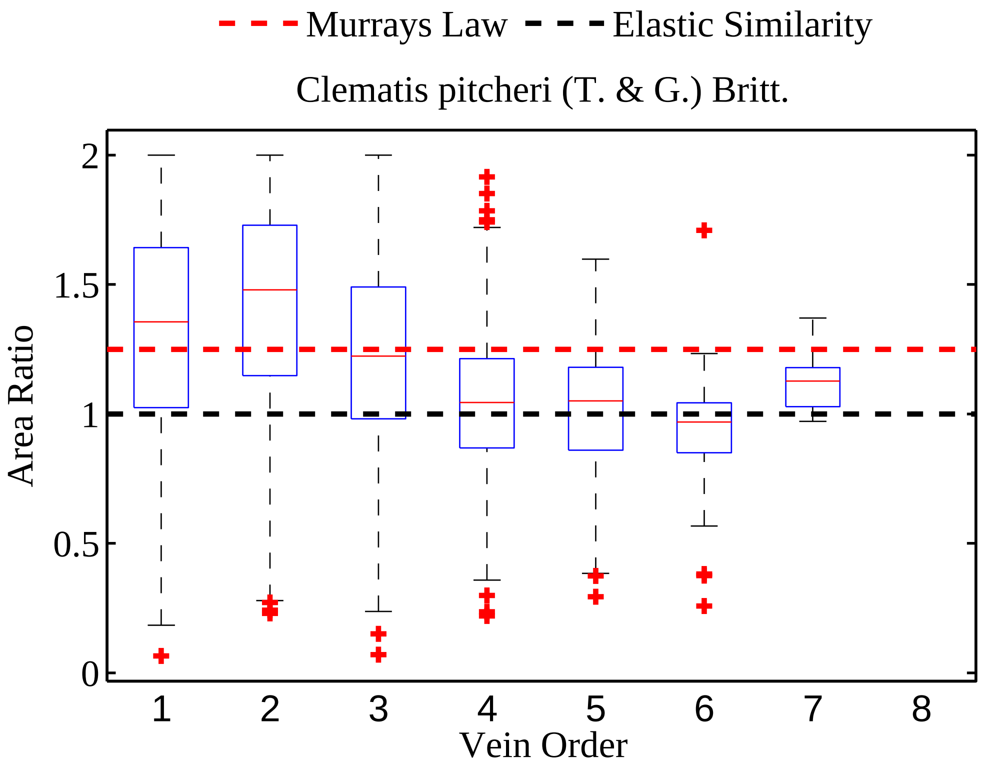

Murrays Law
Elastic Similarity
Clematis pitcheri (T. & G.) Britt.
Area Ratio
Vein Order
0
0.5
1
1.5
2
1
2
3
4
5
6
7
8

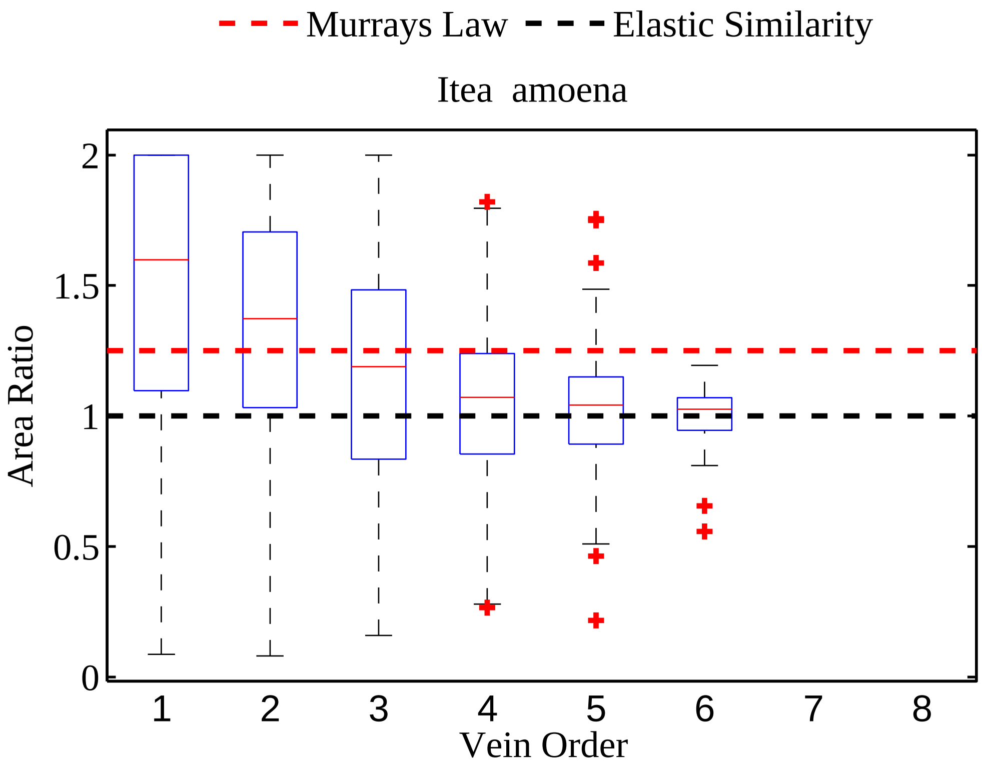

Murrays Law
Elastic Similarity
Itea amoena
Area Ratio
Vein Order
0
0.5
1
1.5
2
1
2
3
4
5
6
7
8

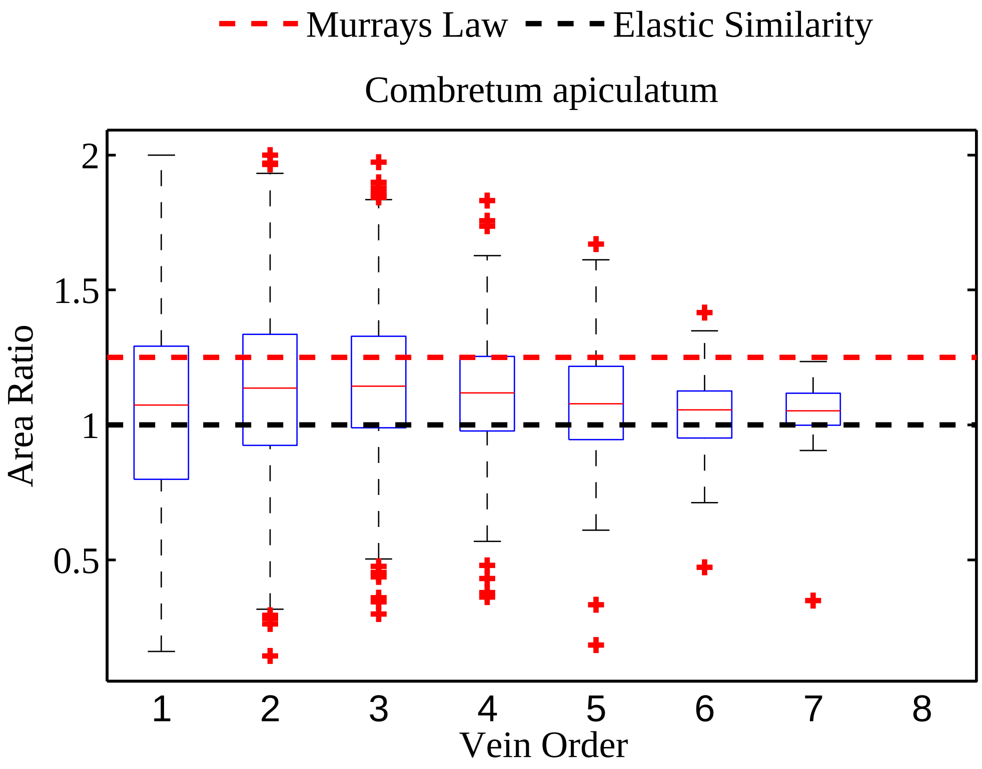
Murrays Law
Elastic Similarity
Combretum apiculatum
Area Ratio
Vein Order
2
1.5
1
0.5
1
2
3
4
5
6
7
8

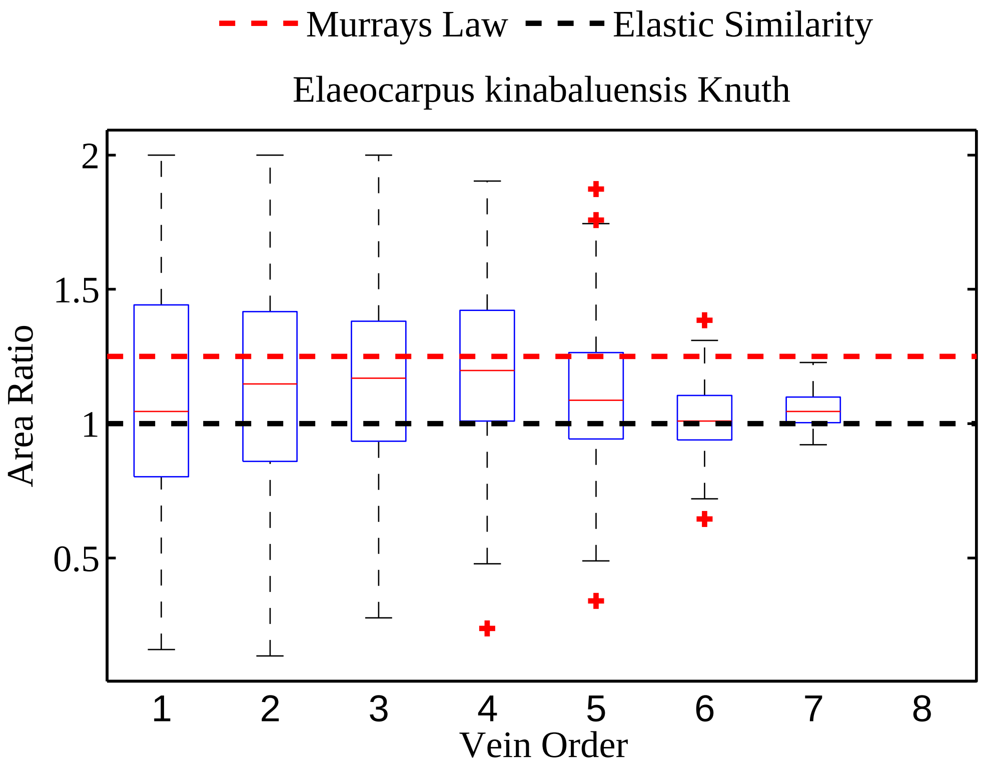
Murrays Law
Elastic Similarity
Elaeocarpus kinabaluensis Knuth
Area Ratio
2
1.5
1
0.5
1
2
3
4
5
6
7
8
Vein Order

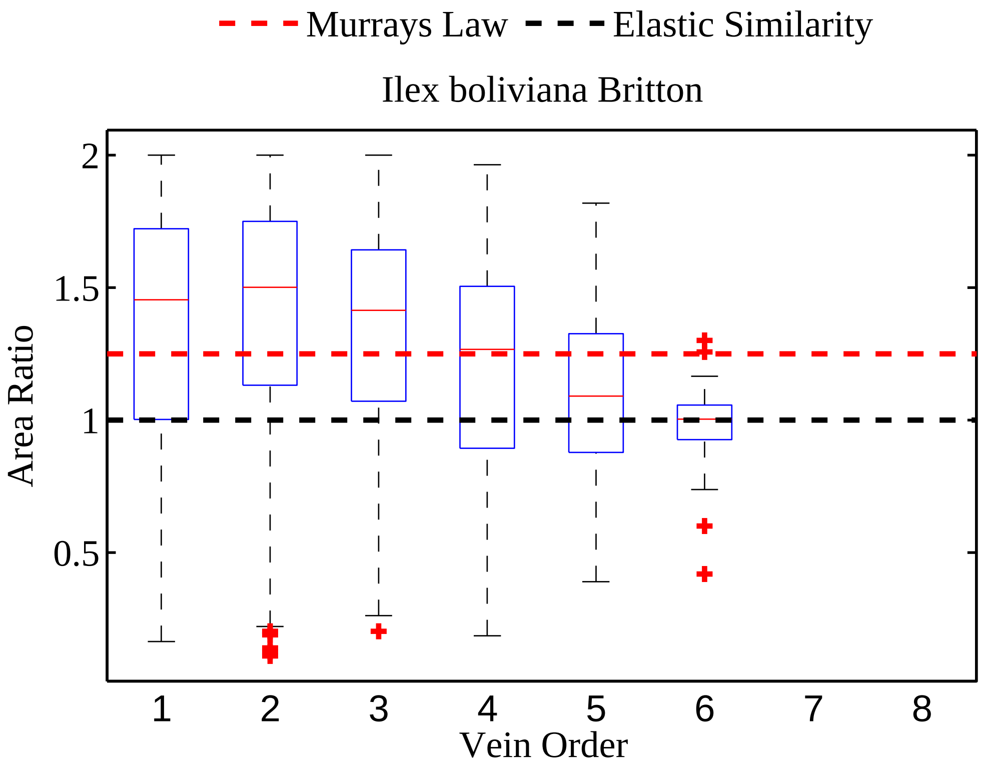
Murrays Law
Elastic Similarity
Ilex boliviana Britton
Area Ratio
2
1.5
1
0.5
1
2
3
4
5
6
7
8
Vein Order

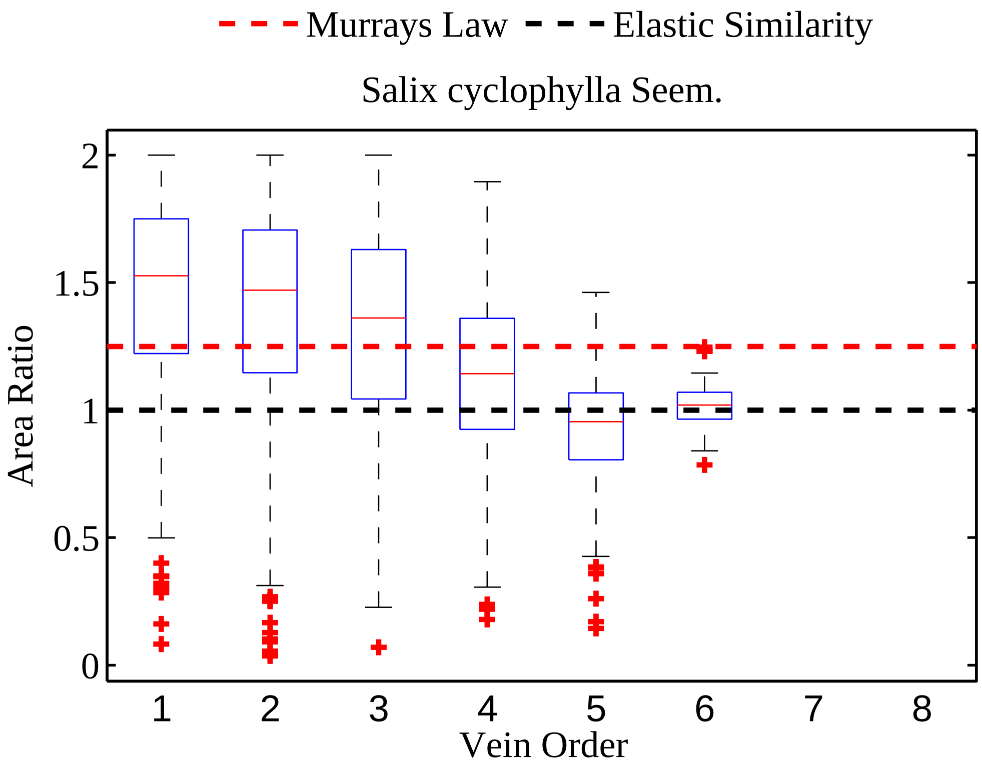

Murrays Law
Elastic Similarity
Salix cyclophylla Seem.
Area Ratio
0
0.5
1
1.5
2
1
2
3
4
5
6
7
8
Vein Order

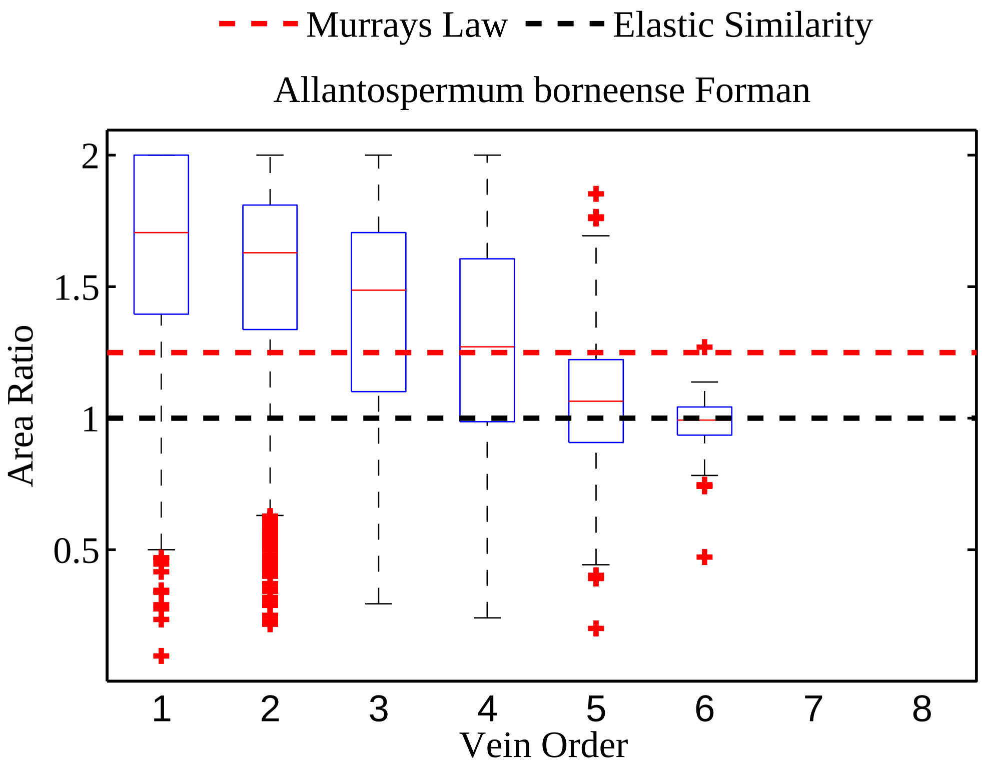

Murrays Law
Elastic Similarity
Allantospermum borneense Forman
Area Ratio
2
1.5
1
0.5
1
2
3
4
5
6
7
8
Vein Order

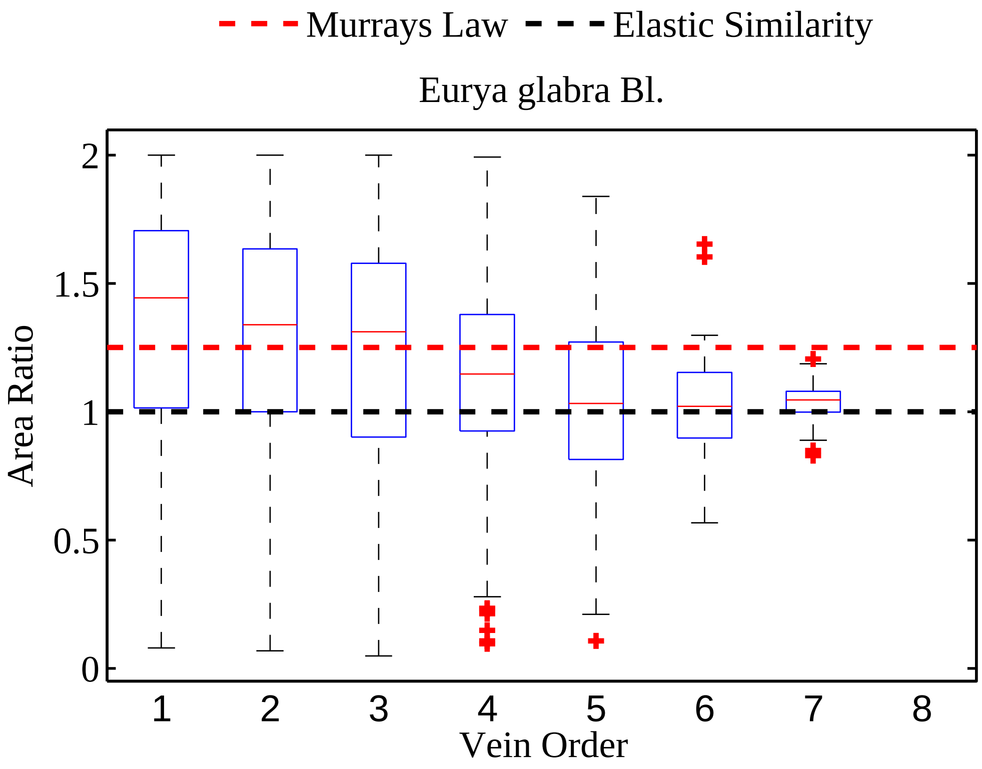
Murrays Law
Elastic Similarity
Eurya glabra Bl.
Area Ratio
Vein Order
0
0.5
1
1.5
2
1
2
3
4
5
6
7
8

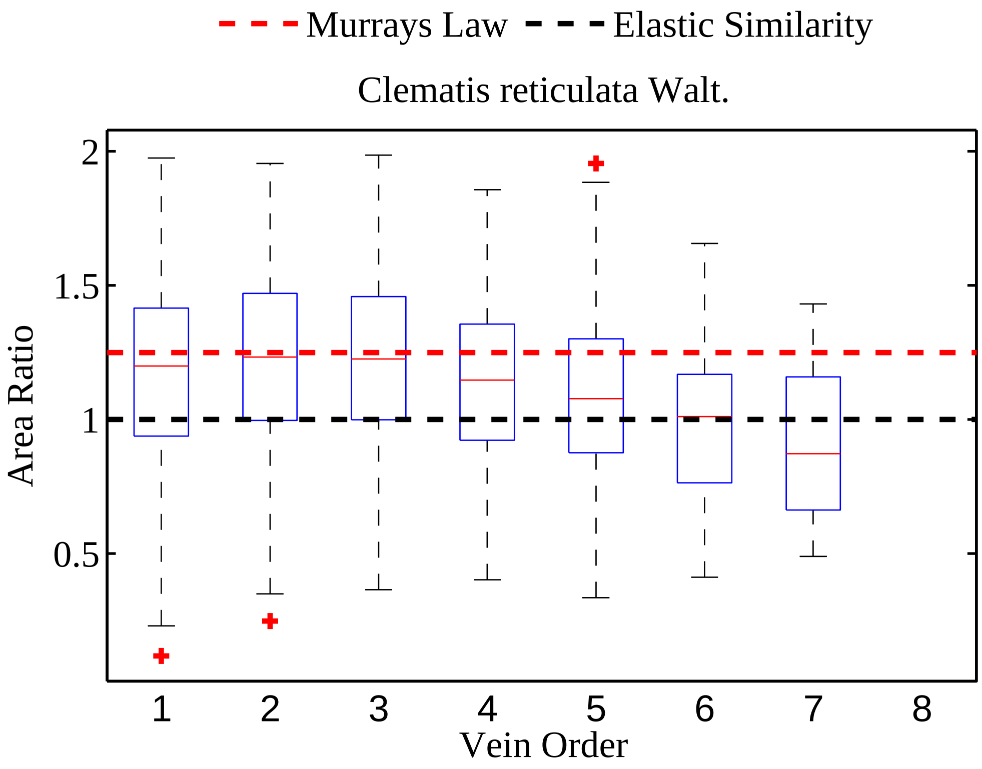
Murrays Law
Elastic Similarity
Clematis reticulata Walt.
Area Ratio
2
1.5
1
0.5
1
2
3
4
5
6
7
8
Vein Order

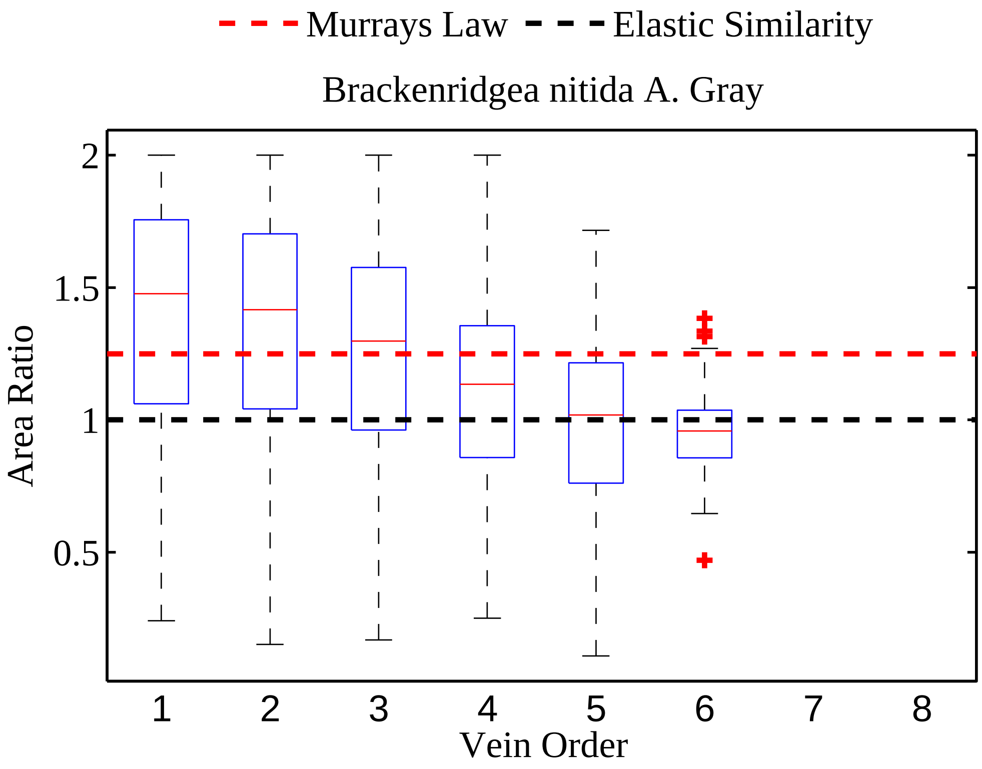

Murrays Law
Elastic Similarity
Brackenridgea nitida A. Gray
Area Ratio
2
1.5
1
0.5
1
2
3
4
5
6
7
8
Vein Order

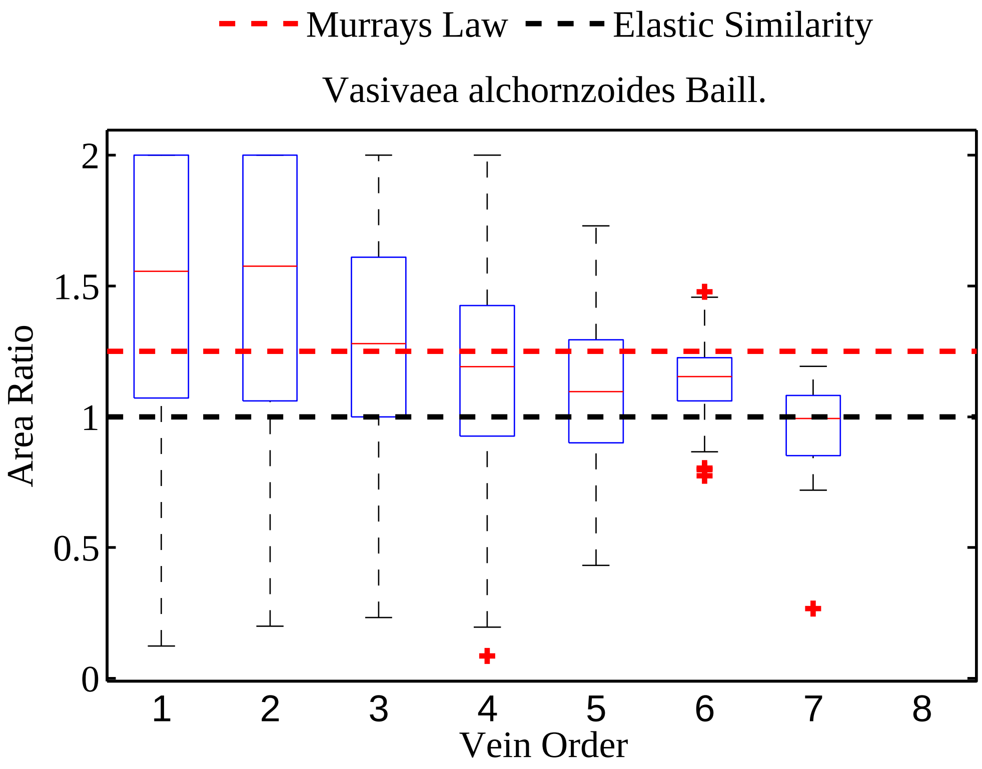

Murrays Law
Elastic Similarity
Vasivaea alchornzoides Baill.
Area Ratio
0
0.5
1
1.5
2
1
2
3
4
5
6
7
8
Vein Order

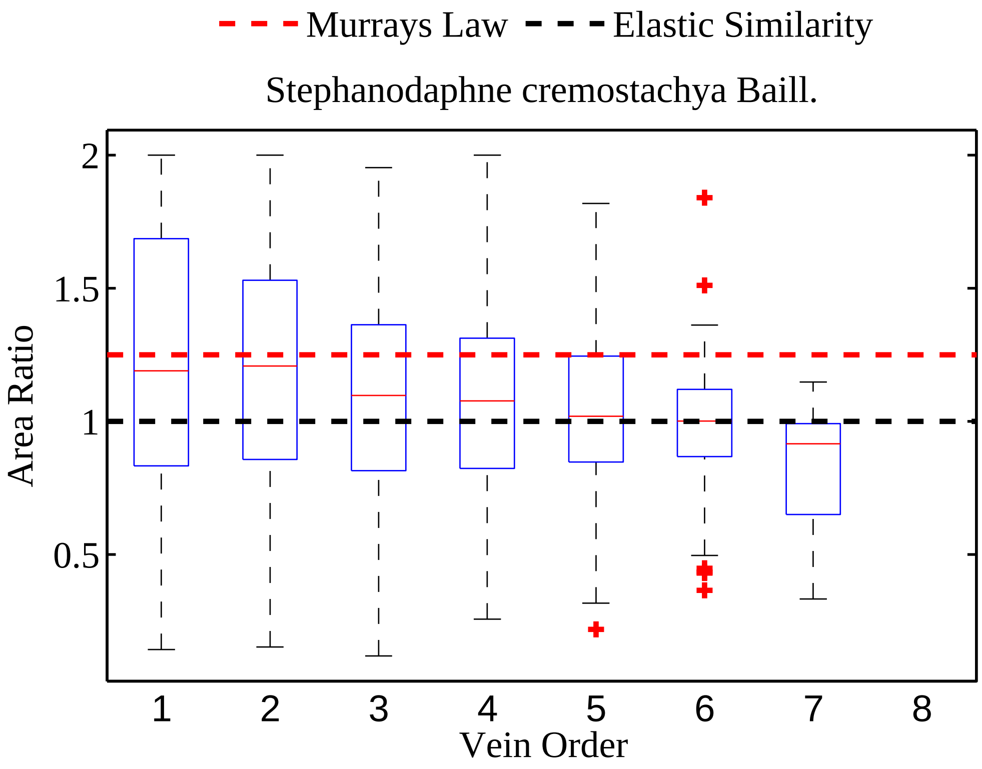
Murrays Law
Elastic Similarity
Stephanodaphne cremostachya Baill.
Area Ratio
2
1.5
1
0.5
1
2
3
4
5
6
7
8
Vein Order

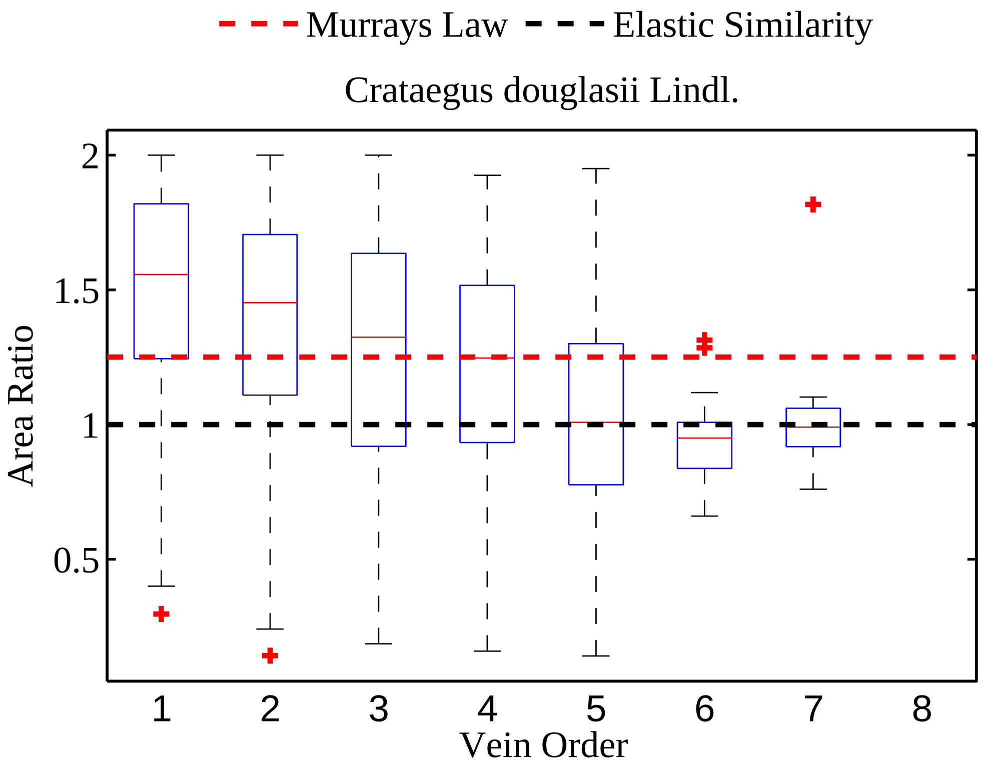

Murrays Law
Elastic Similarity
Crataegus douglasii Lindl.
Area Ratio
Vein Order
2
1.5
1
0.5
1
2
3
4
5
6
7
8

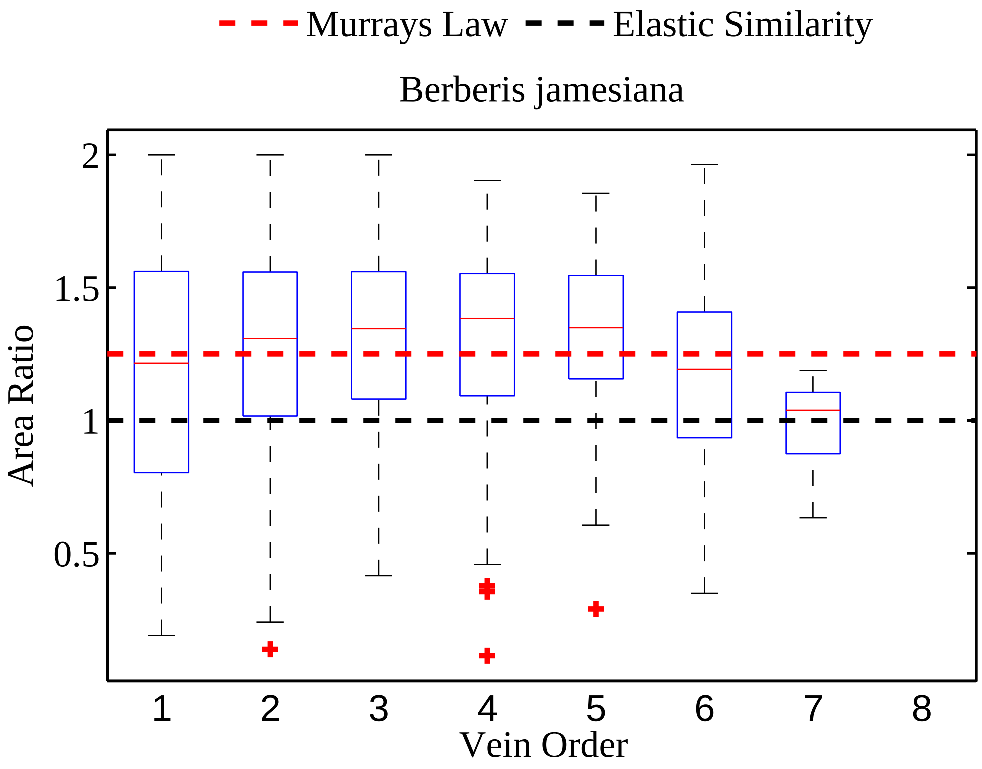

Murrays Law
Elastic Similarity
Berberis jamesiana
Area Ratio
2
1.5
1
0.5
1
2
3
4
5
6
7
8
Vein Order

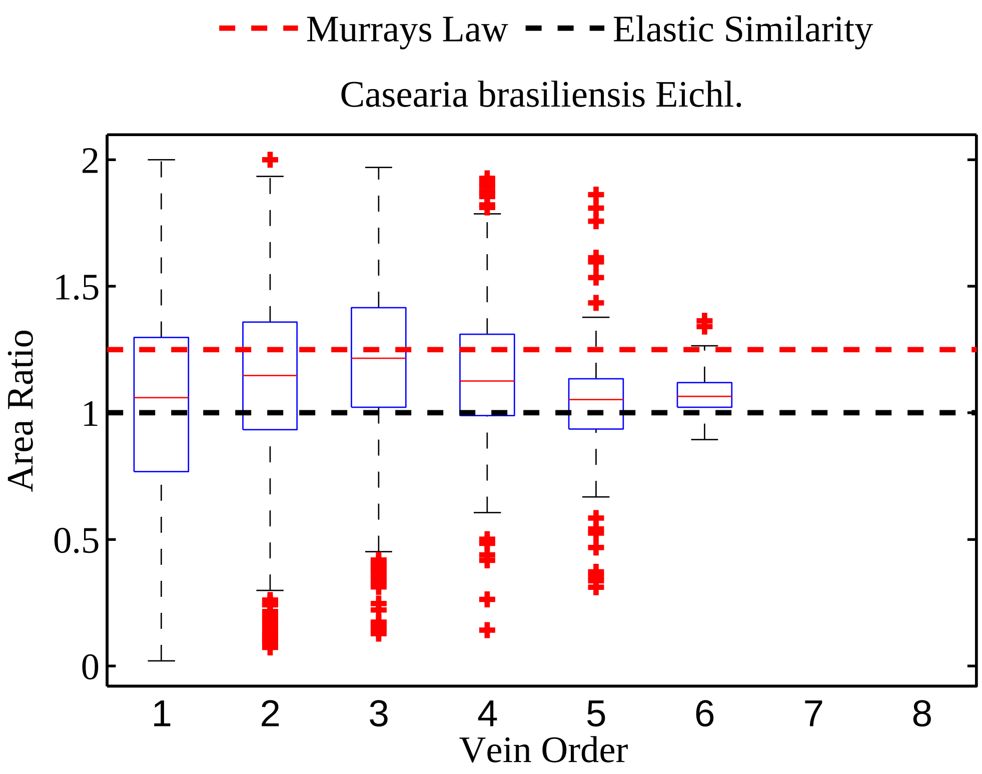

Murrays Law
Elastic Similarity
Casearia brasiliensis Eichl.
Area Ratio
0
0.5
1
1.5
2
1
2
3
4
5
6
7
8
Vein Order

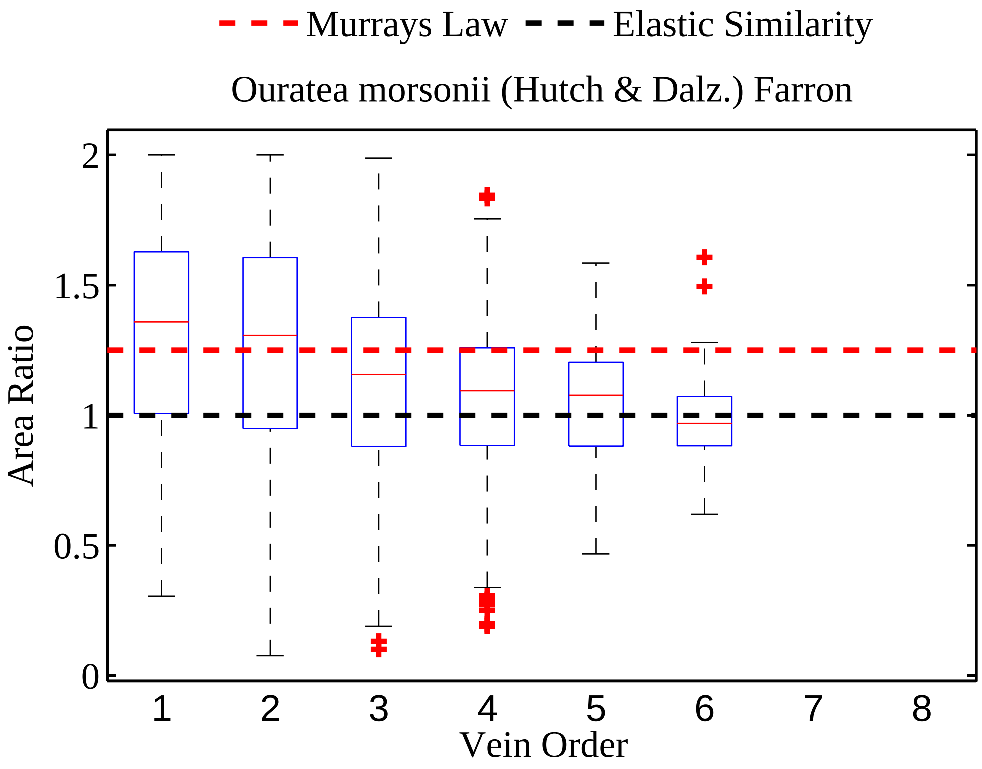

Murrays Law
Elastic Similarity
Ouratea morsonii (Hutch & Dalz.) Farron
Area Ratio
Vein Order
0
0.5
1
1.5
2
1
2
3
4
5
6
7
8

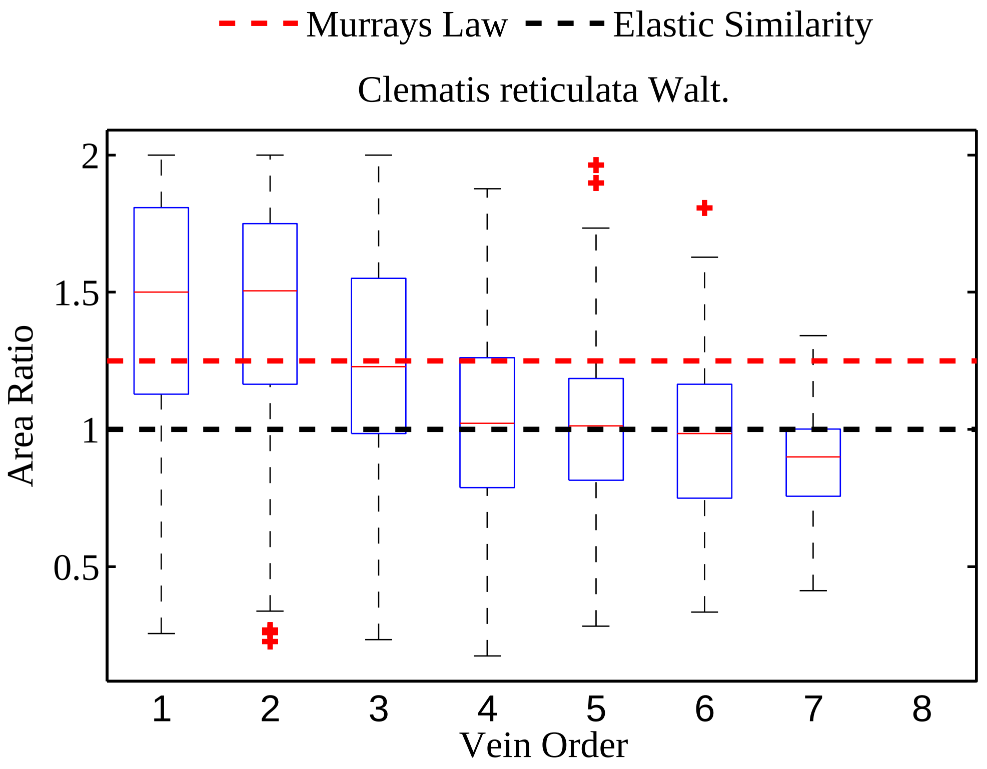
Murrays Law
Elastic Similarity
Clematis reticulata Walt.
Area Ratio
2
1.5
1
0.5
1
2
3
4
5
6
7
8
Vein Order

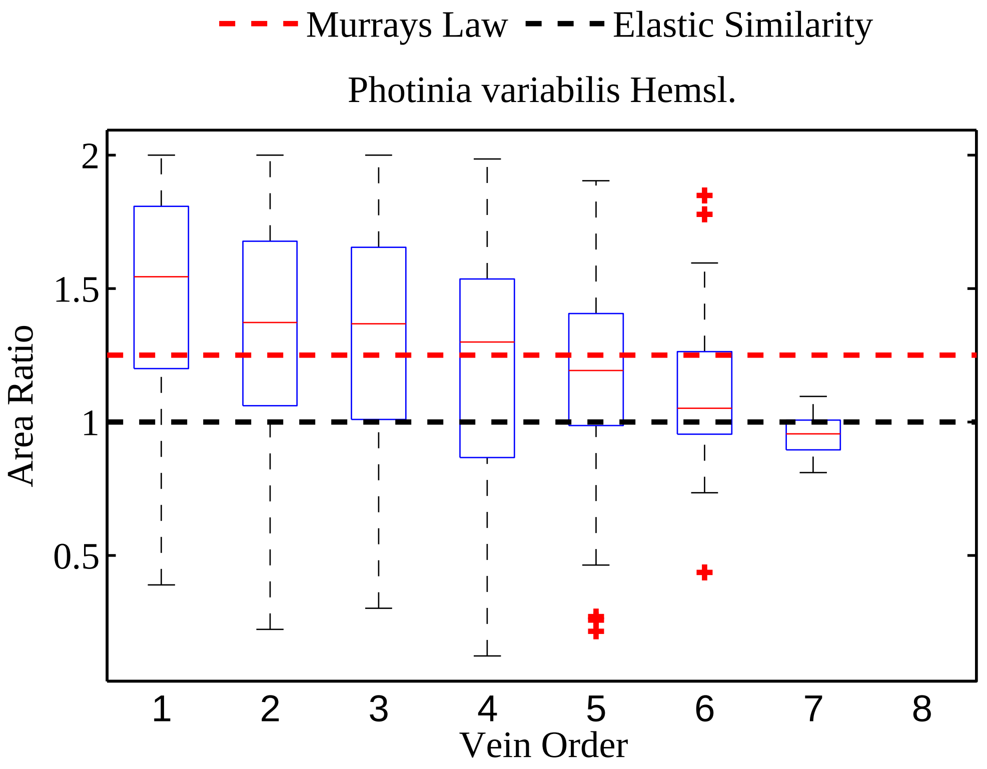
Murrays Law
Elastic Similarity
Photinia variabilis Hemsl.
Area Ratio
2
1.5
1
0.5
1
2
3
4
5
6
7
8
Vein Order

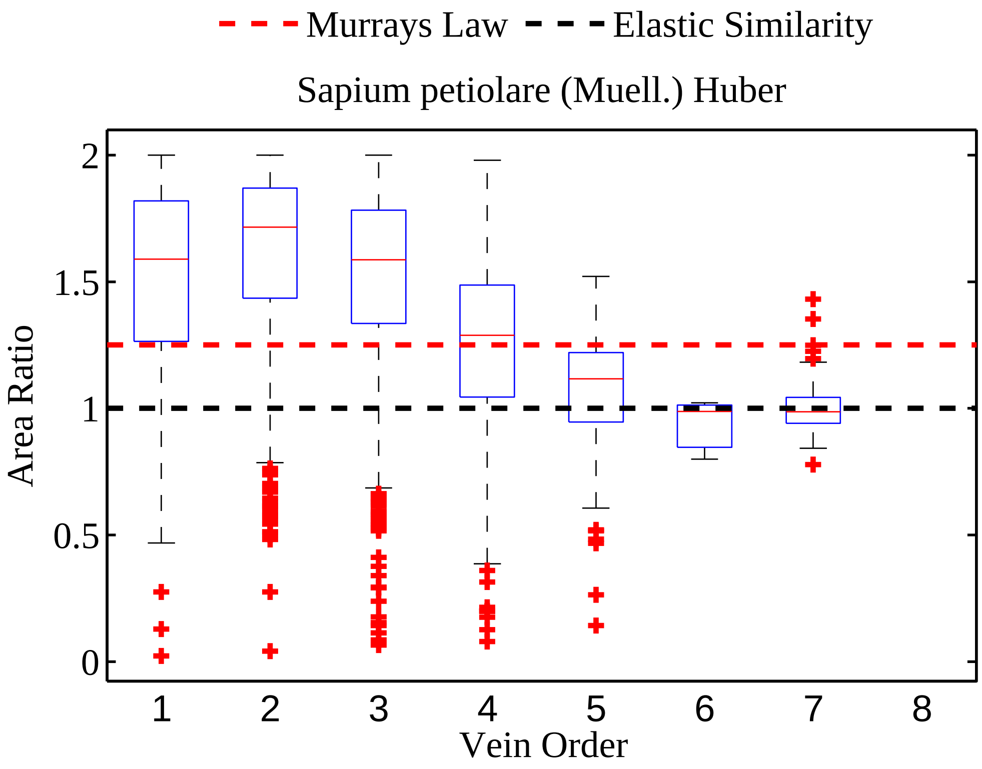

Murrays Law
Elastic Similarity
Sapium petiolare (Muell.) Huber
Area Ratio
2
1.5
1
0.5
0
1
2
3
4
5
6
7
8
Vein Order

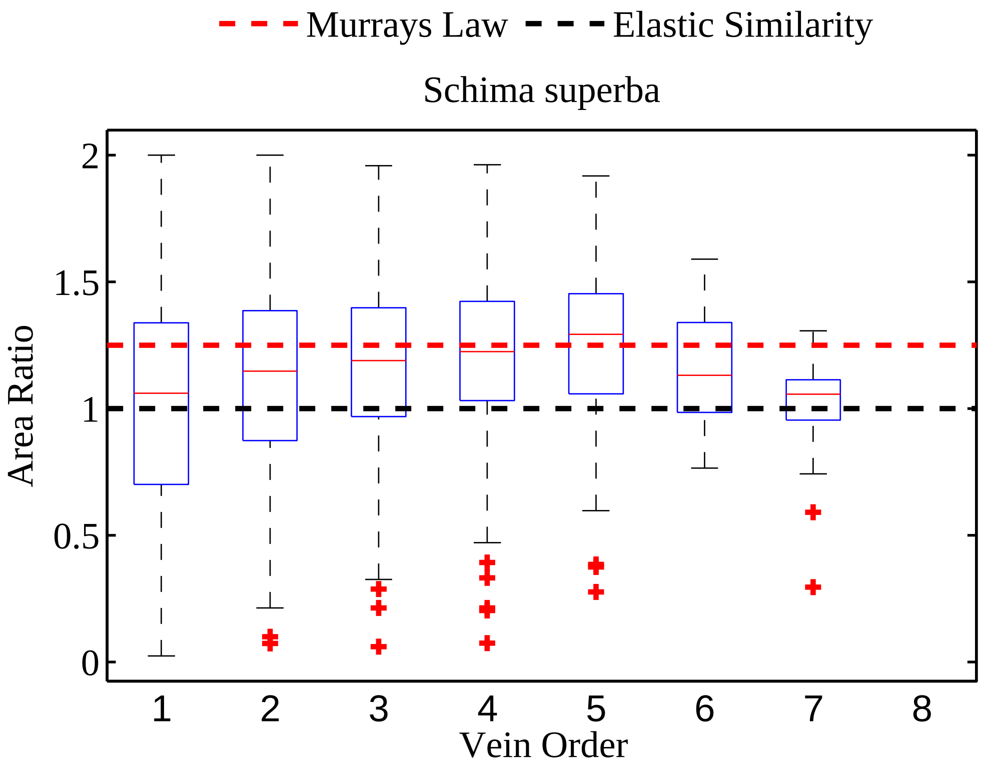

Murrays Law
Elastic Similarity
Schima superba
Area Ratio
0
0.5
1
1.5
2
1
2
3
4
5
6
7
8
Vein Order

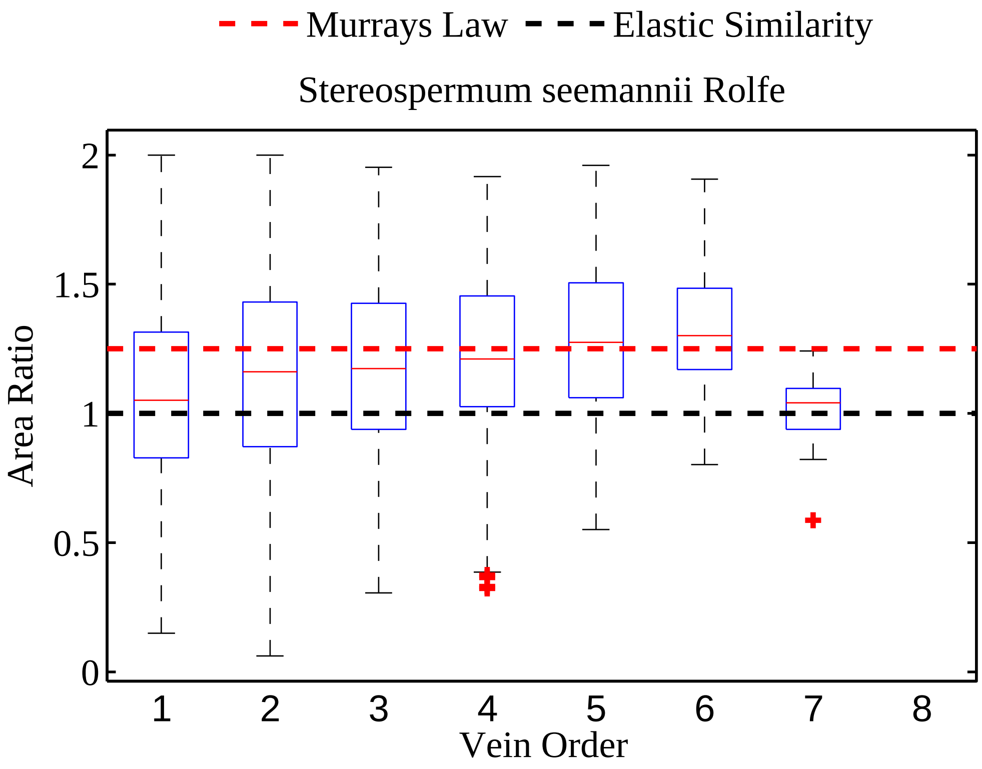
Murrays Law
Elastic Similarity
Stereospermum seemannii Rolfe
Area Ratio
0
0.5
1
1.5
2
1
2
3
4
5
6
7
8
Vein Order

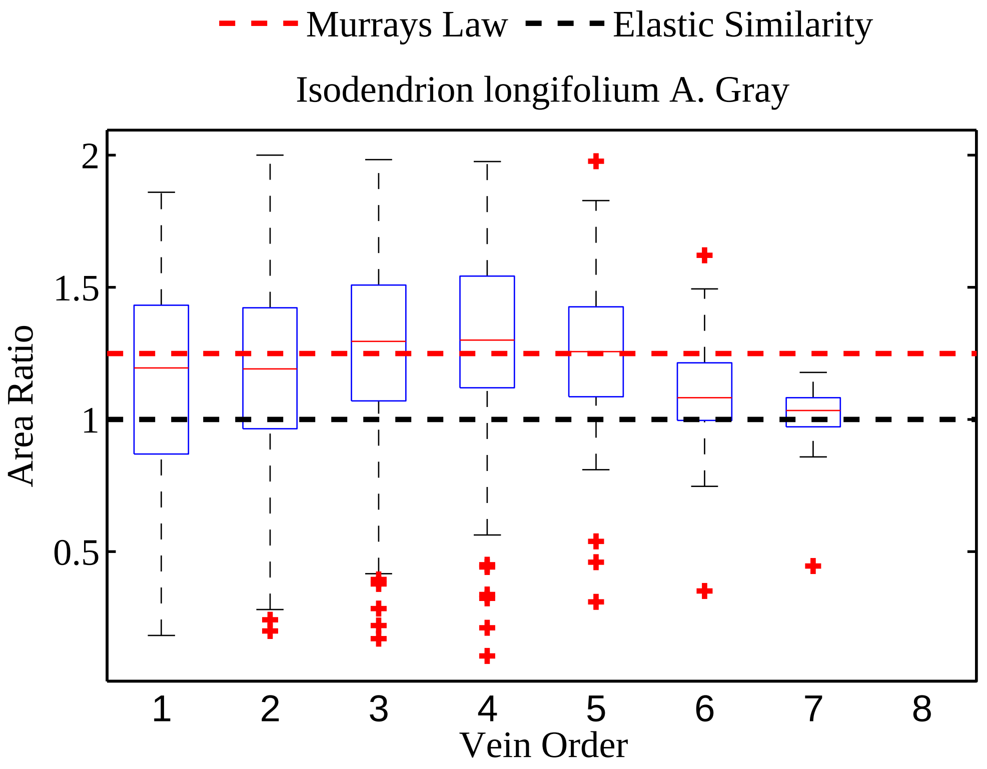

Murrays Law
Elastic Similarity
Isodendrion longifolium A. Gray
Area Ratio
2
1.5
1
0.5
1
2
3
4
5
6
7
8
Vein Order

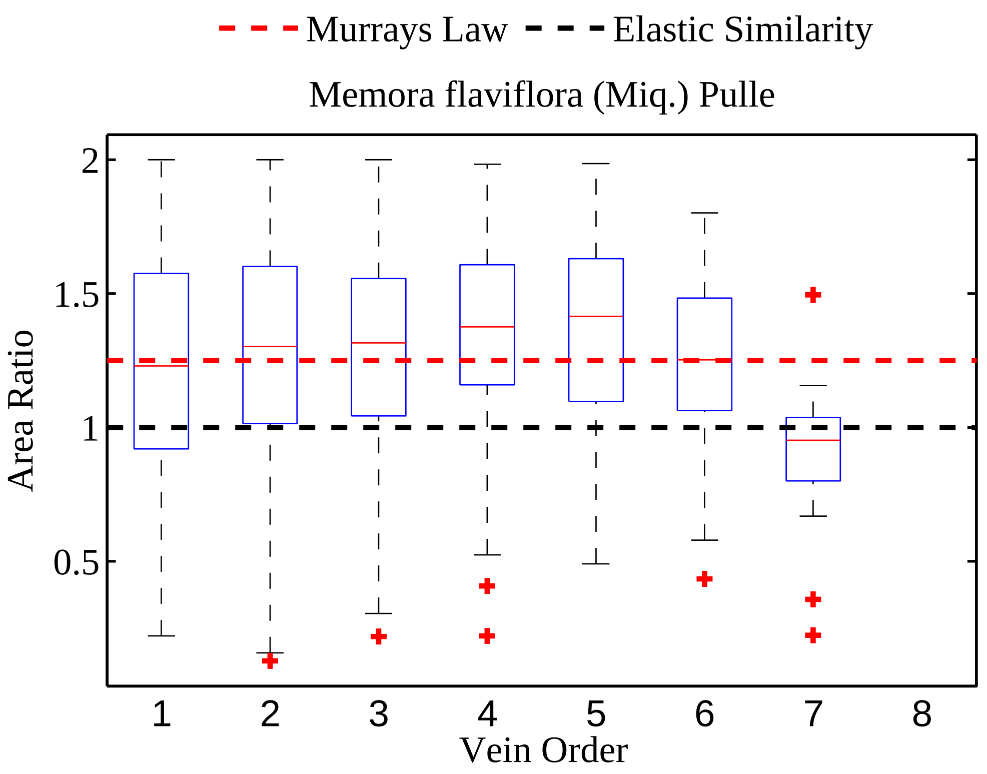

Murrays Law
Elastic Similarity
Memora flaviflora (Miq.) Pulle
Area Ratio
2
1.5
1
0.5
1
2
3
4
5
6
7
8
Vein Order

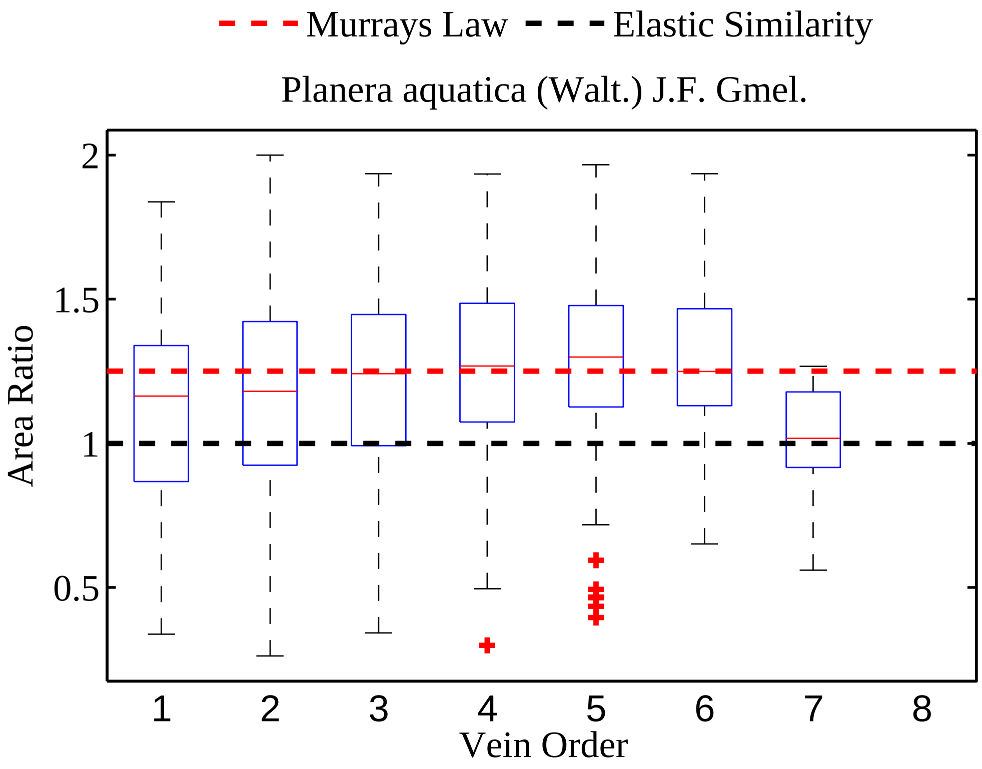
Murrays Law
Elastic Similarity
Planera aquatica (Walt.) J.F. Gmel.
Area Ratio
2
1.5
1
0.5
1
2
3
4
5
6
7
8
Vein Order

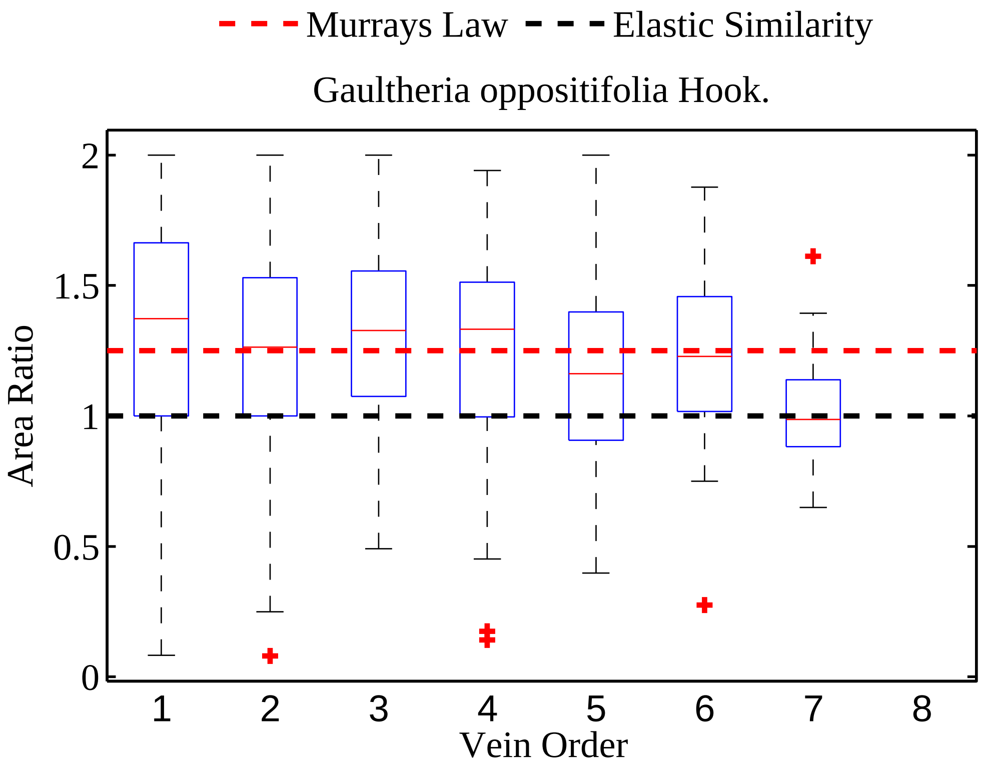
Murrays Law
Elastic Similarity
Gaultheria oppositifolia Hook.
Area Ratio
0
0.5
1
1.5
2
1
2
3
4
5
6
7
8
Vein Order

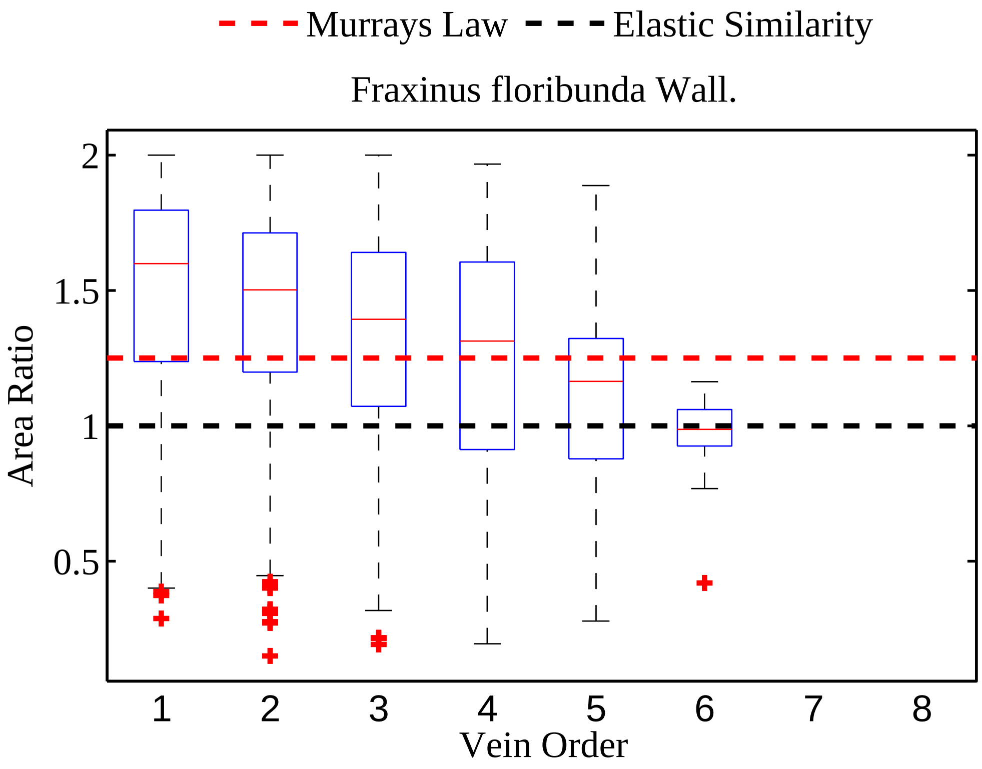

Murrays Law
Elastic Similarity
Fraxinus floribunda Wall.
Area Ratio
2
1.5
1
0.5
1
2
3
4
5
6
7
8
Vein Order

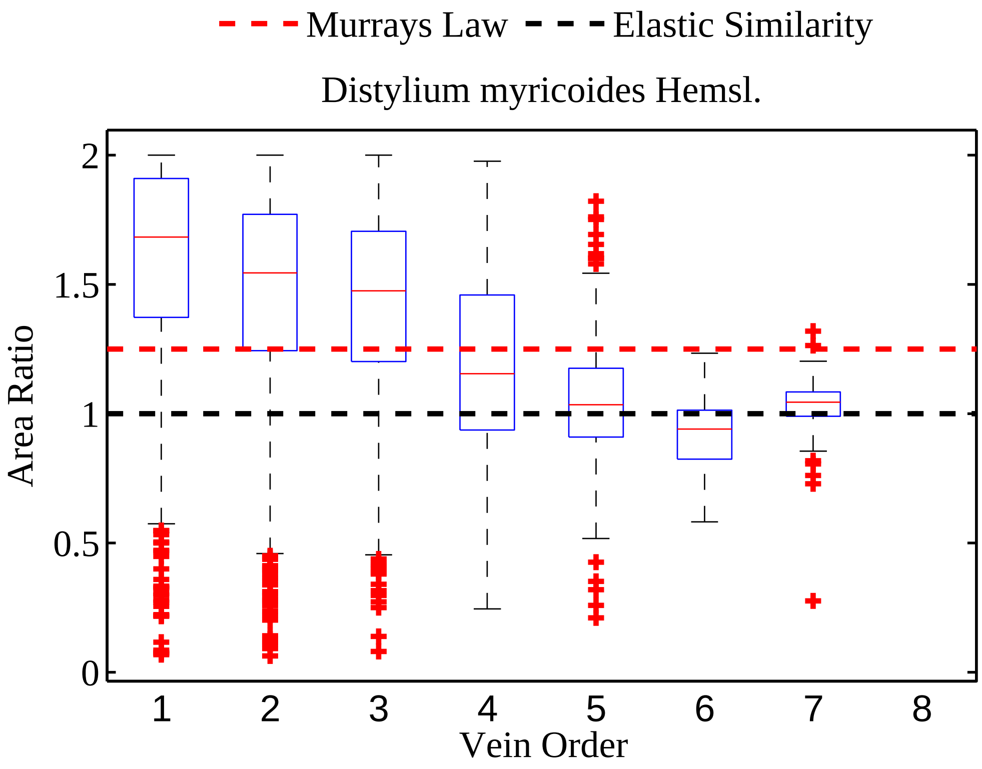
Murrays Law
Elastic Similarity
Distylium myricoides Hemsl.
Area Ratio
0
0.5
1
1.5
2
1
2
3
4
5
6
7
8
Vein Order

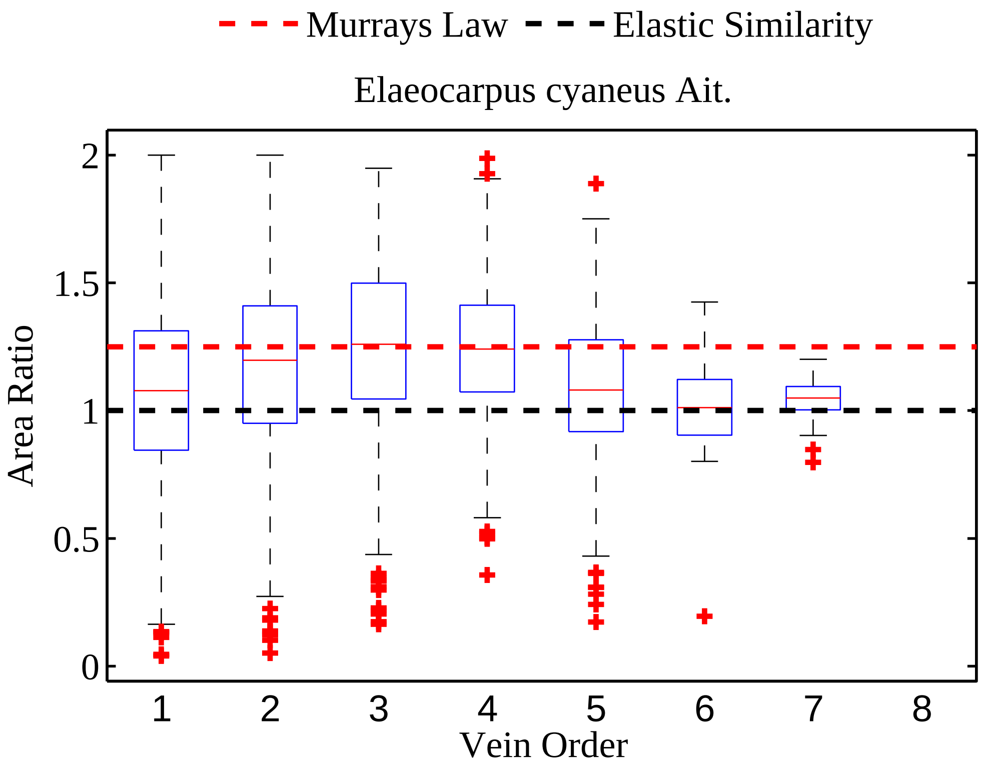

Murrays Law
Elastic Similarity
Elaeocarpus cyaneus Ait.
Area Ratio
0
0.5
1
1.5
2
1
2
3
4
5
6
7
8
Vein Order

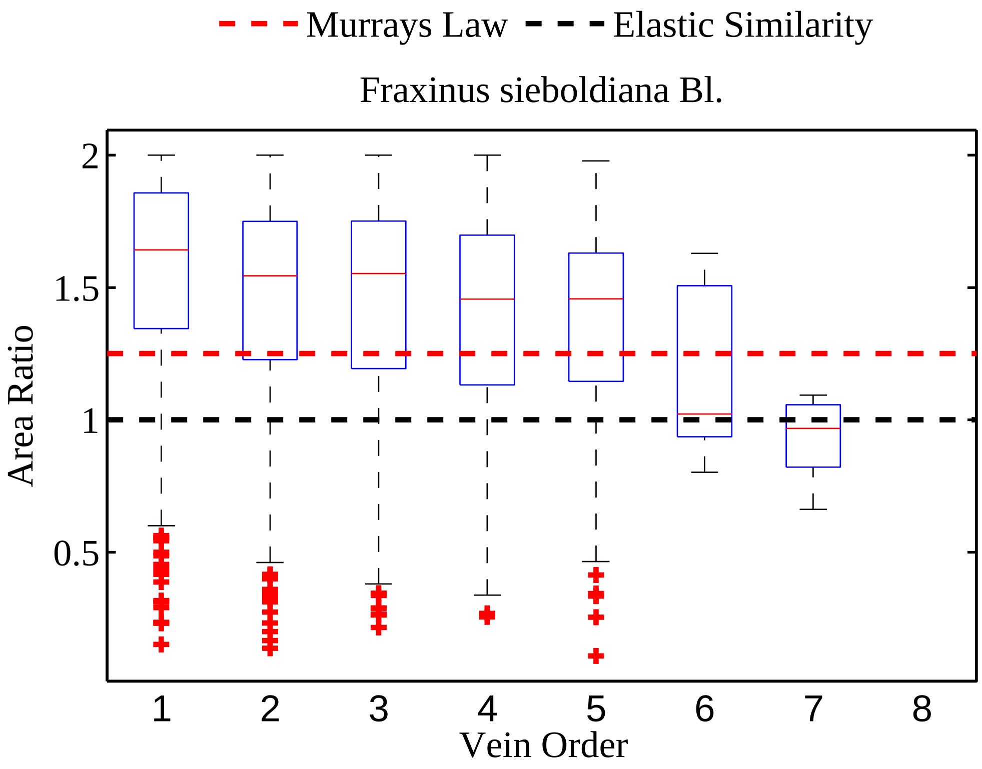

Murrays Law
Elastic Similarity
Fraxinus sieboldiana Bl.
Area Ratio
2
1.5
1
0.5
1
2
3
4
5
6
7
8
Vein Order

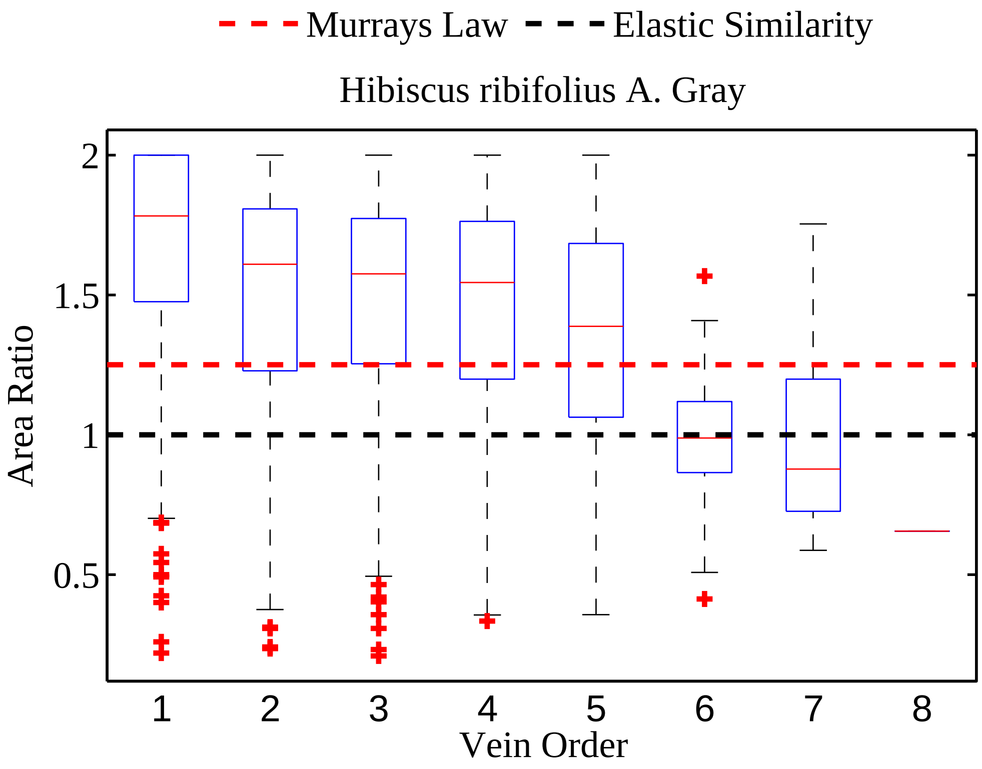

Murrays Law
Elastic Similarity
Hibiscus ribifolius A. Gray
Area Ratio
2
1.5
1
0.5
1
2
3
4
5
6
7
8
Vein Order

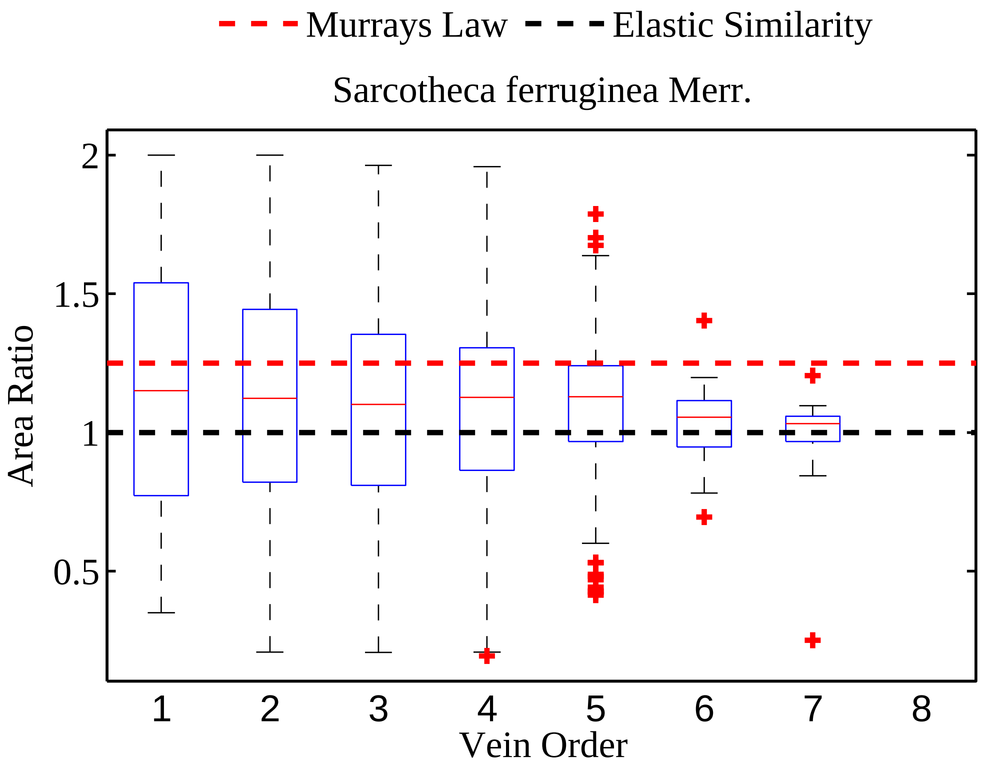
Murrays Law
Elastic Similarity
Sarcotheca ferruginea Merr.
Area Ratio
Vein Order
2
1.5
1
0.5
1
2
3
4
5
6
7
8

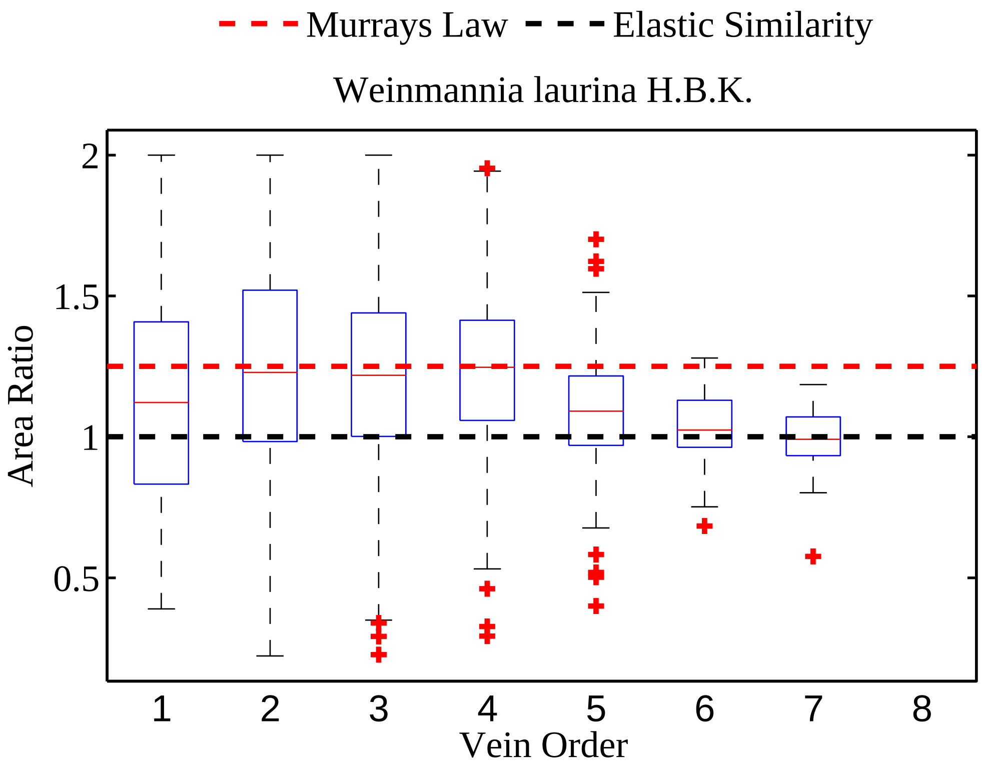

Murrays Law
Elastic Similarity
Weinmannia laurina H.B.K.
Area Ratio
2
1.5
1
0.5
1
2
3
4
5
6
7
8
Vein Order

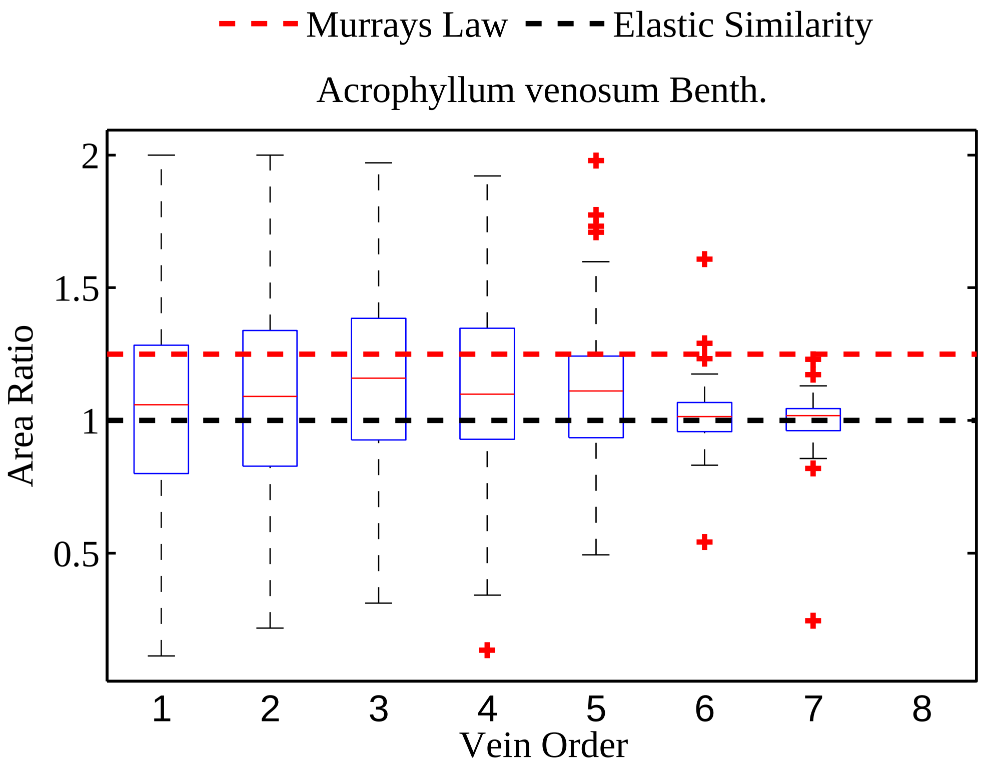

Murrays Law
Elastic Similarity
Acrophyllum venosum Benth.
Area Ratio
2
1.5
1
0.5
1
2
3
4
5
6
7
8
Vein Order

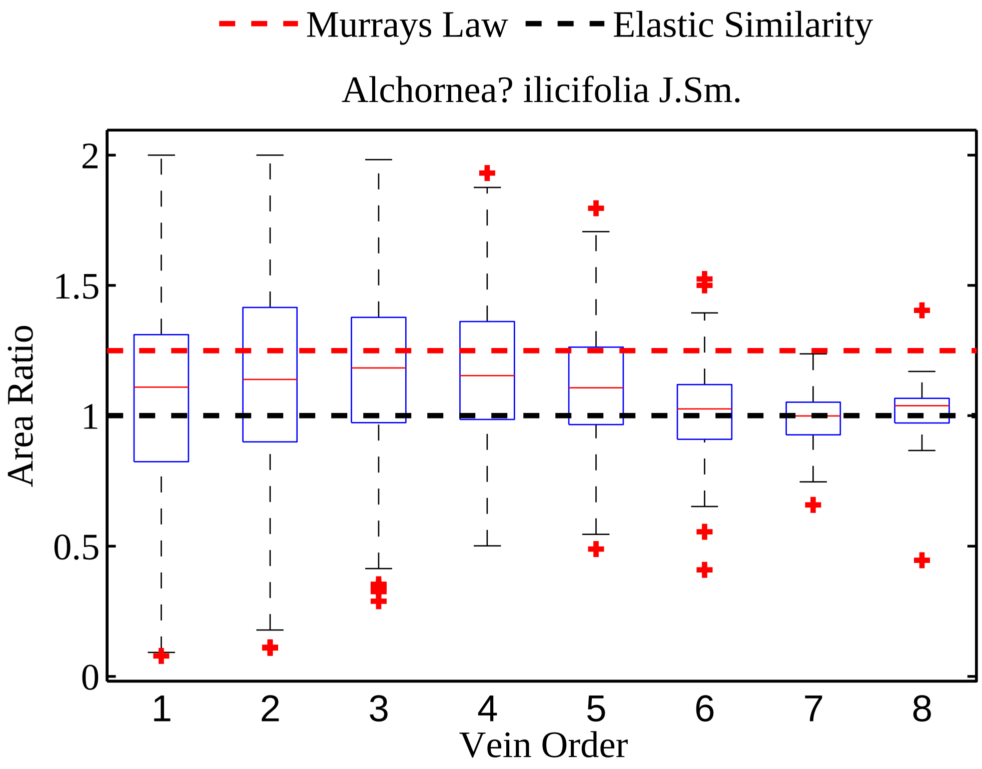

Murrays Law
Elastic Similarity
Alchornea? ilicifolia J.Sm.
Area Ratio
0
0.5
1
1.5
2
1
2
3
4
5
6
7
8
Vein Order

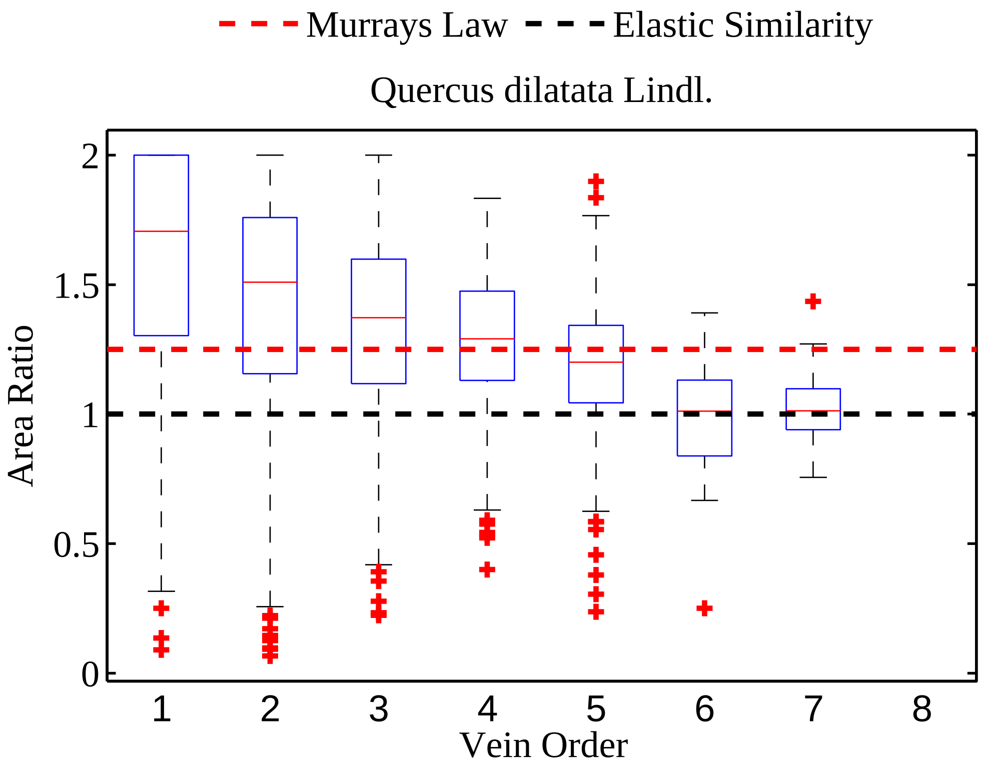

Murrays Law
Elastic Similarity
Quercus dilatata Lindl.
Area Ratio
Vein Order
0
0.5
1
1.5
2
1
2
3
4
5
6
7
8

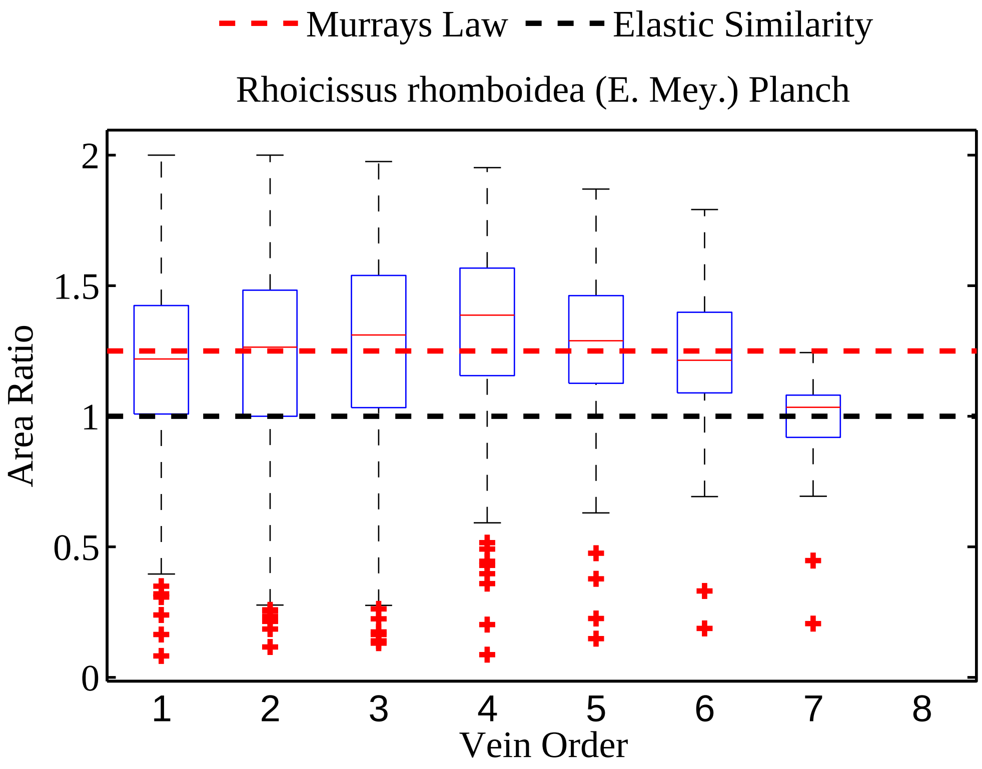

Murrays Law
Elastic Similarity
Rhoicissus rhomboidea (E. Mey.) Planch
Area Ratio
Vein Order
0
0.5
1
1.5
2
1
2
3
4
5
6
7
8

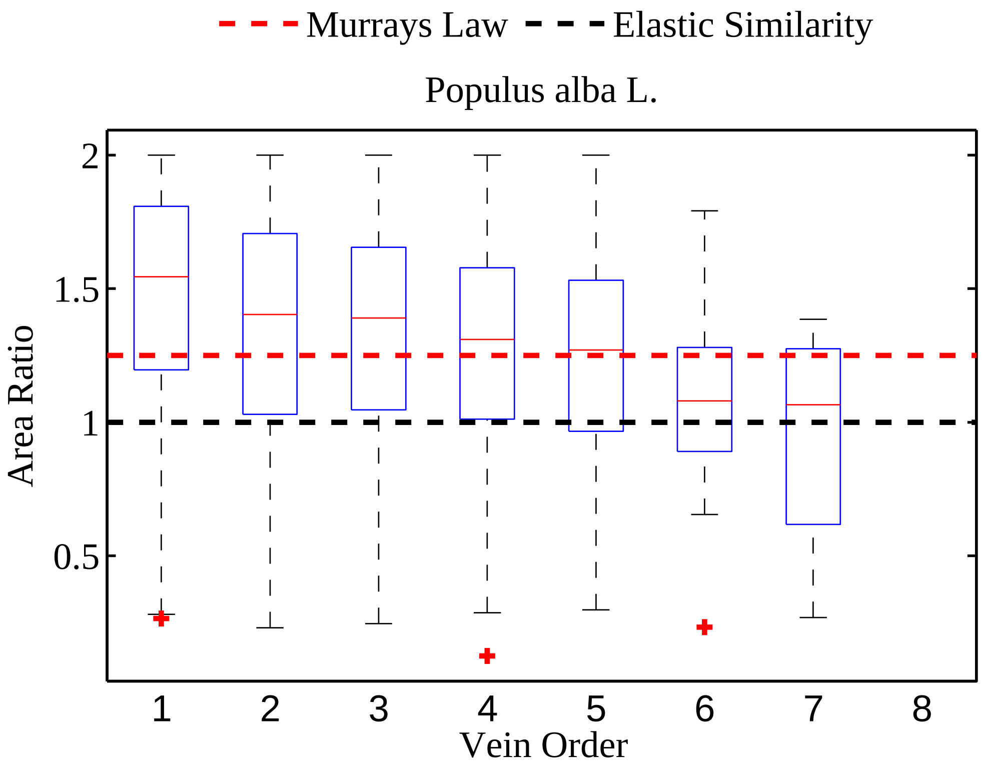

Murrays Law
Elastic Similarity
Populus alba L.
Area Ratio
Vein Order
2
1.5
1
0.5
1
2
3
4
5
6
7
8

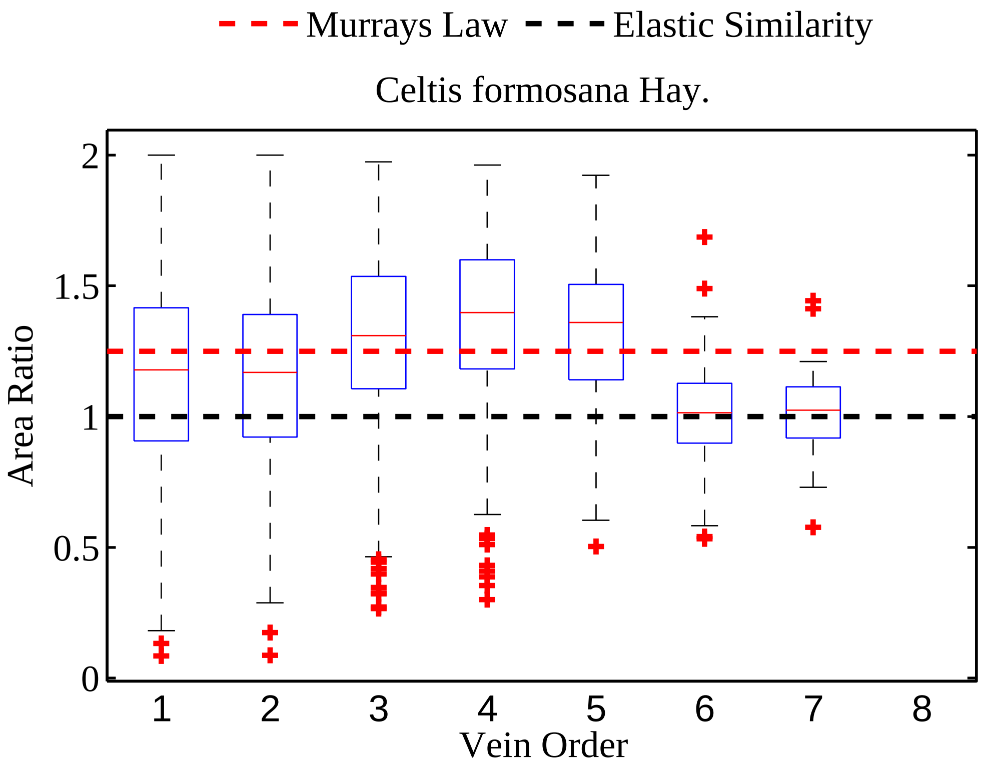

Murrays Law
Elastic Similarity
Celtis formosana Hay.
Area Ratio
Vein Order
0
0.5
1
1.5
2
1
2
3
4
5
6
7
8

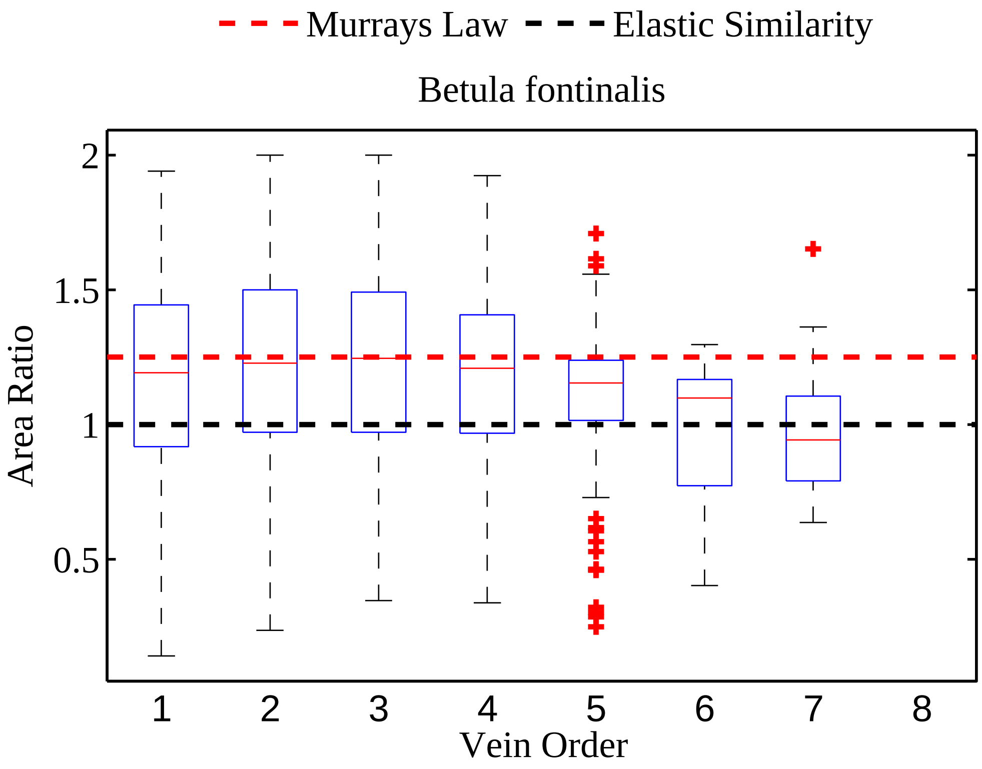

Murrays Law
Elastic Similarity
Betula fontinalis
Area Ratio
2
1.5
1
0.5
1
2
3
4
5
6
7
8
Vein Order

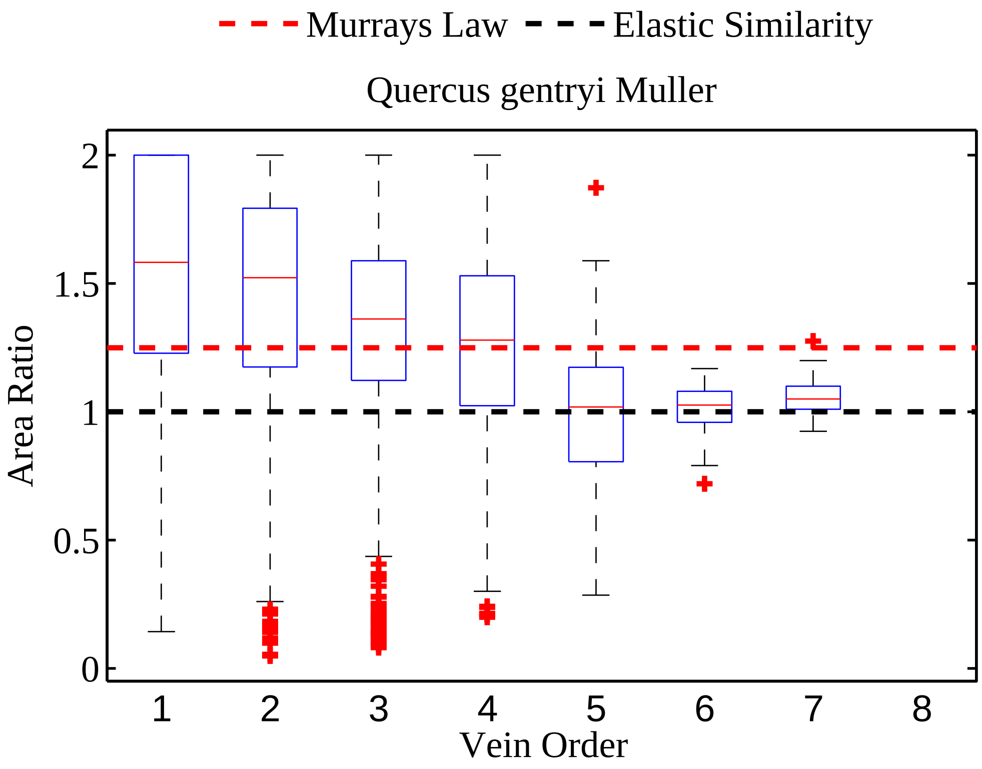

Murrays Law
Elastic Similarity
Quercus gentryi Muller
Area Ratio
0
0.5
1
1.5
2
1
2
3
4
5
6
7
8
Vein Order

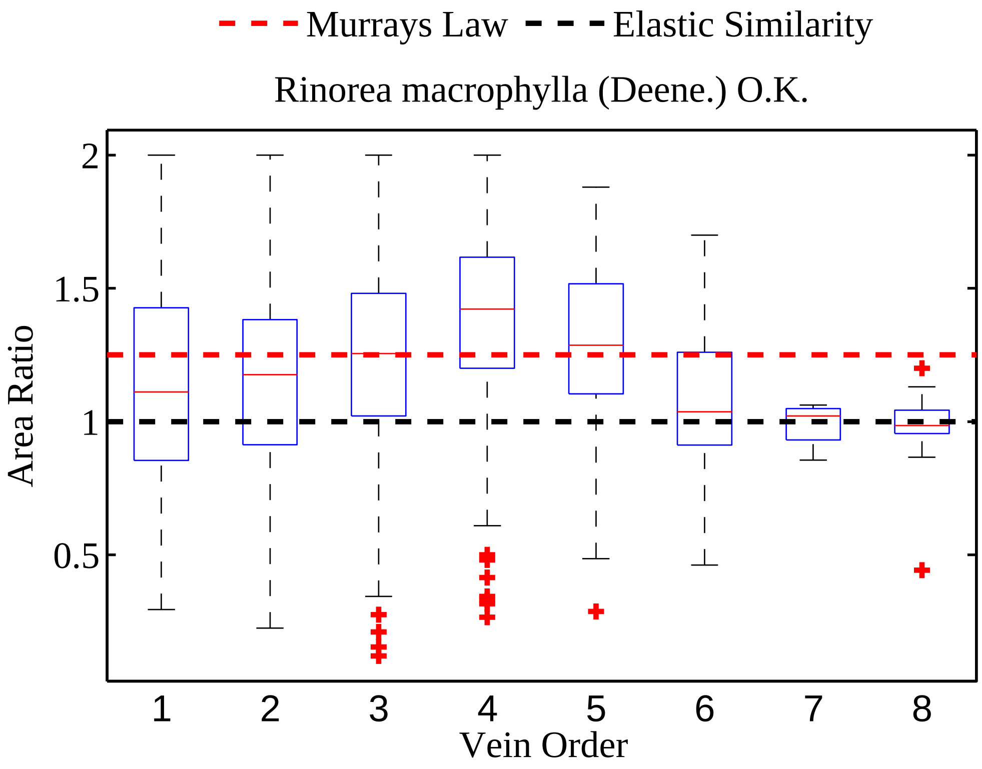

Murrays Law
Elastic Similarity
Rinorea macrophylla (Deene.) O.K.
Area Ratio
2
1.5
1
0.5
1
2
3
4
5
6
7
8
Vein Order

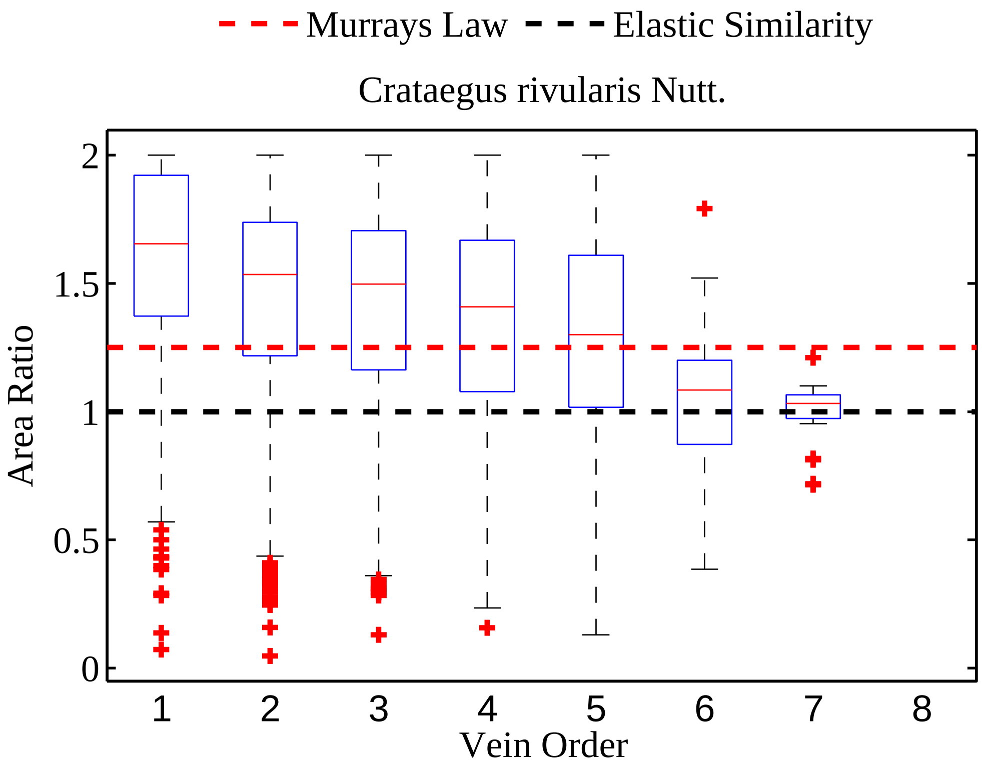

Murrays Law
Elastic Similarity
Crataegus rivularis Nutt.
Area Ratio
0
0.5
1
1.5
2
1
2
3
4
5
6
7
8
Vein Order

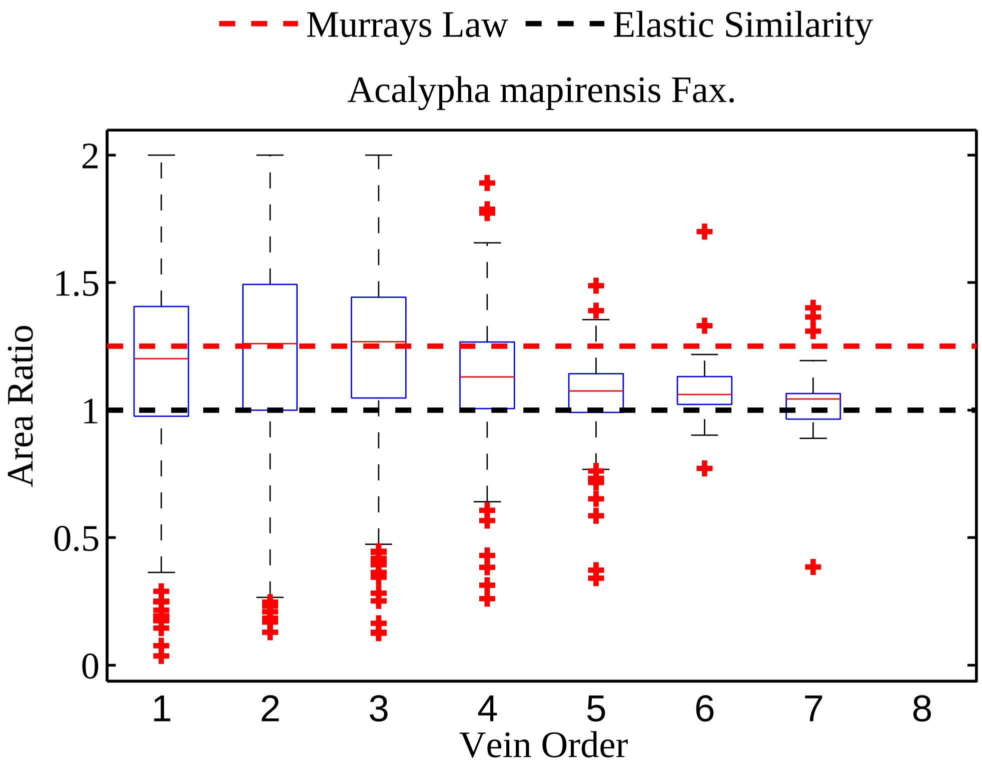

Murrays Law
Elastic Similarity
Acalypha mapirensis Fax.
Area Ratio
Vein Order
0
0.5
1
1.5
2
1
2
3
4
5
6
7
8

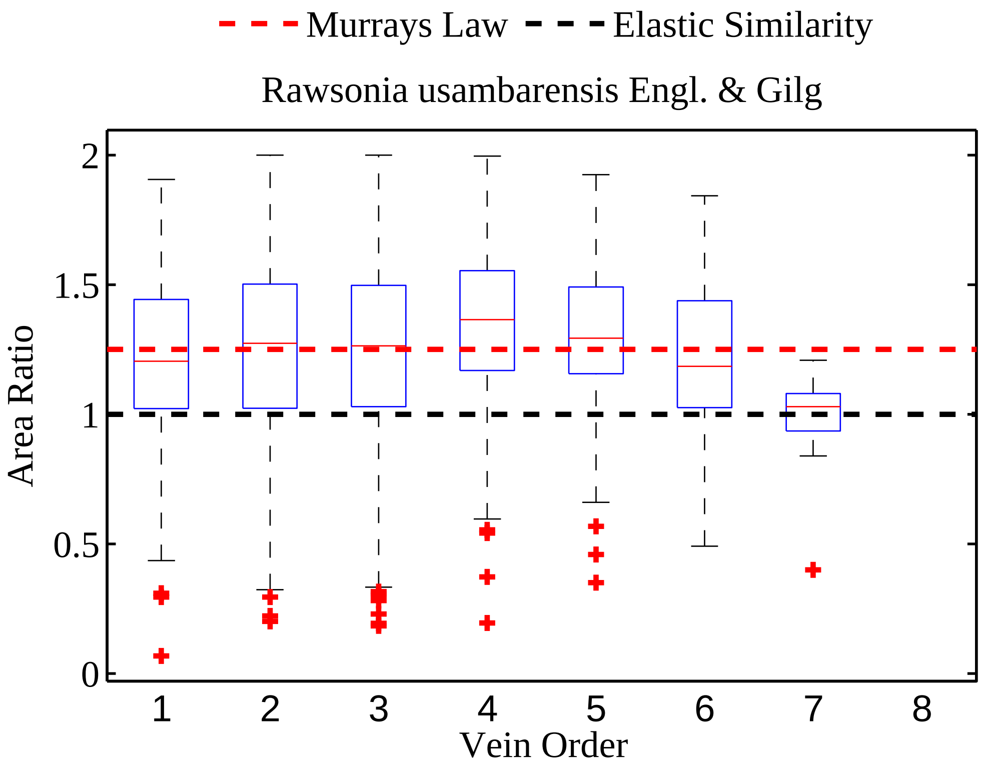

Murrays Law
Elastic Similarity
Rawsonia usambarensis Engl. & Gilg
Area Ratio
0
0.5
1
1.5
2
1
2
3
4
5
6
7
8
Vein Order

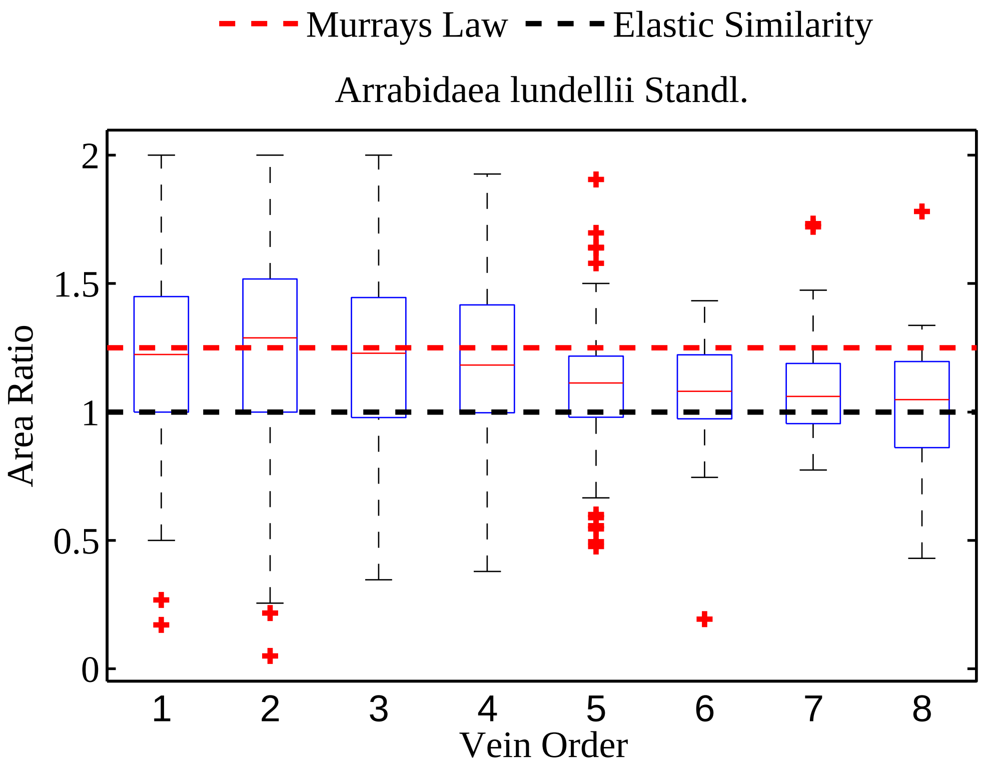

Murrays Law
Elastic Similarity
Arrabidaea lundellii Standl.
Area Ratio
0
0.5
1
1.5
2
1
2
3
4
5
6
7
8
Vein Order

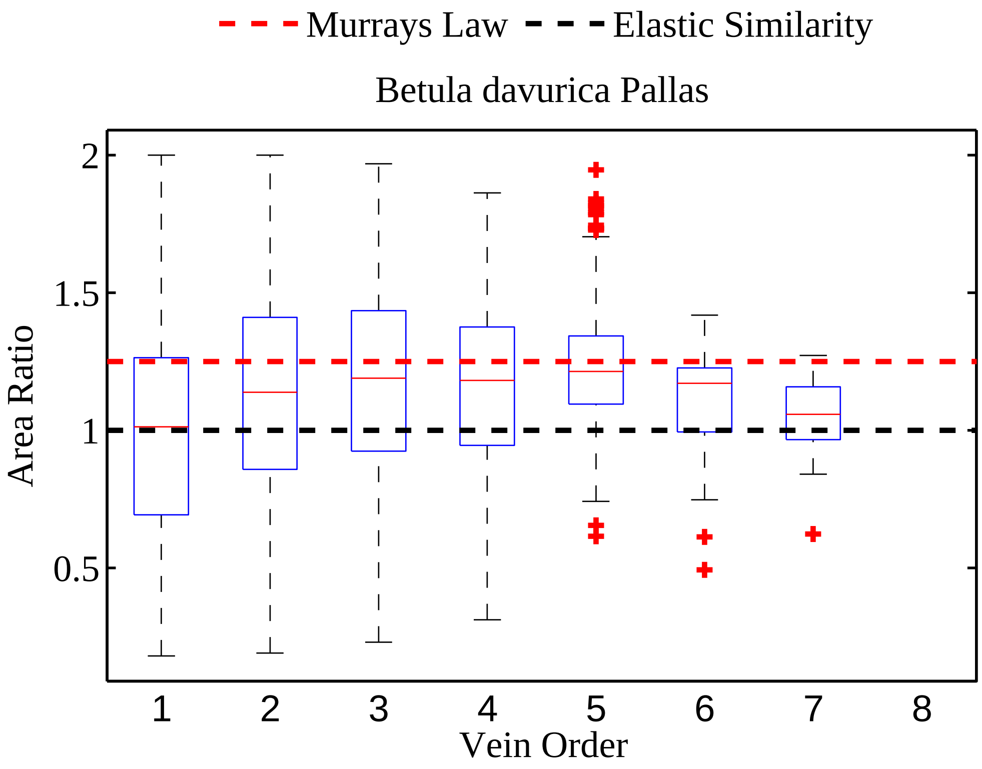

Murrays Law
Elastic Similarity
Betula davurica Pallas
Area Ratio
2
1.5
1
0.5
1
2
3
4
5
6
7
8
Vein Order

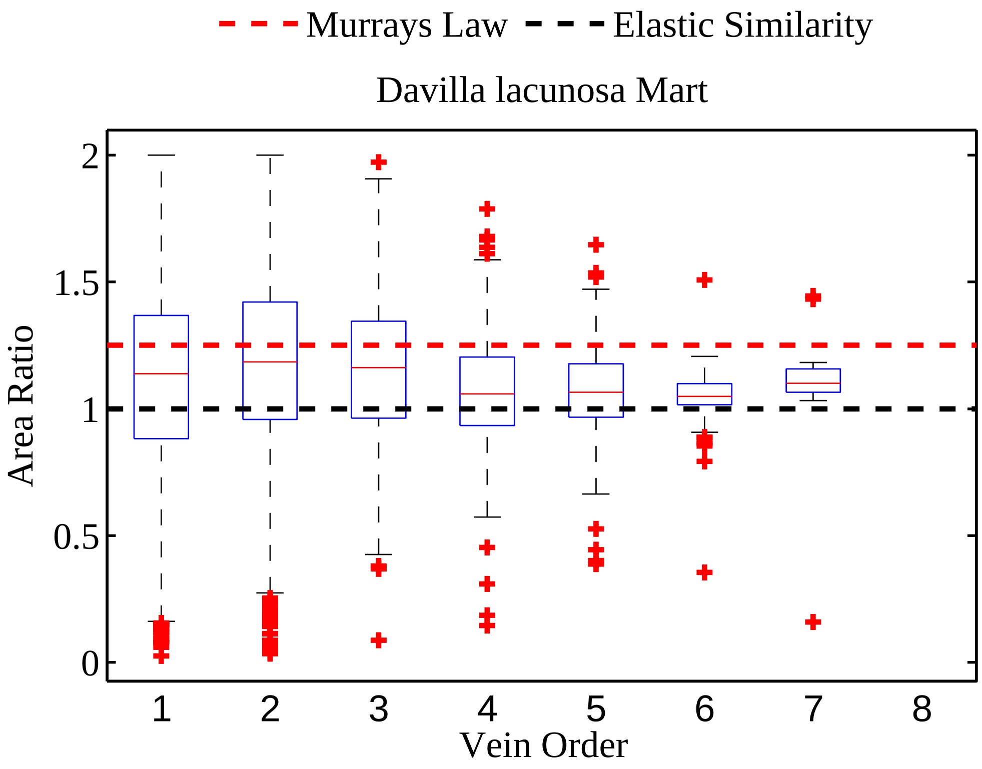
Murrays Law
Elastic Similarity
Davilla lacunosa Mart
Area Ratio
2
1.5
1
0.5
0
1
2
3
4
5
6
7
8
Vein Order

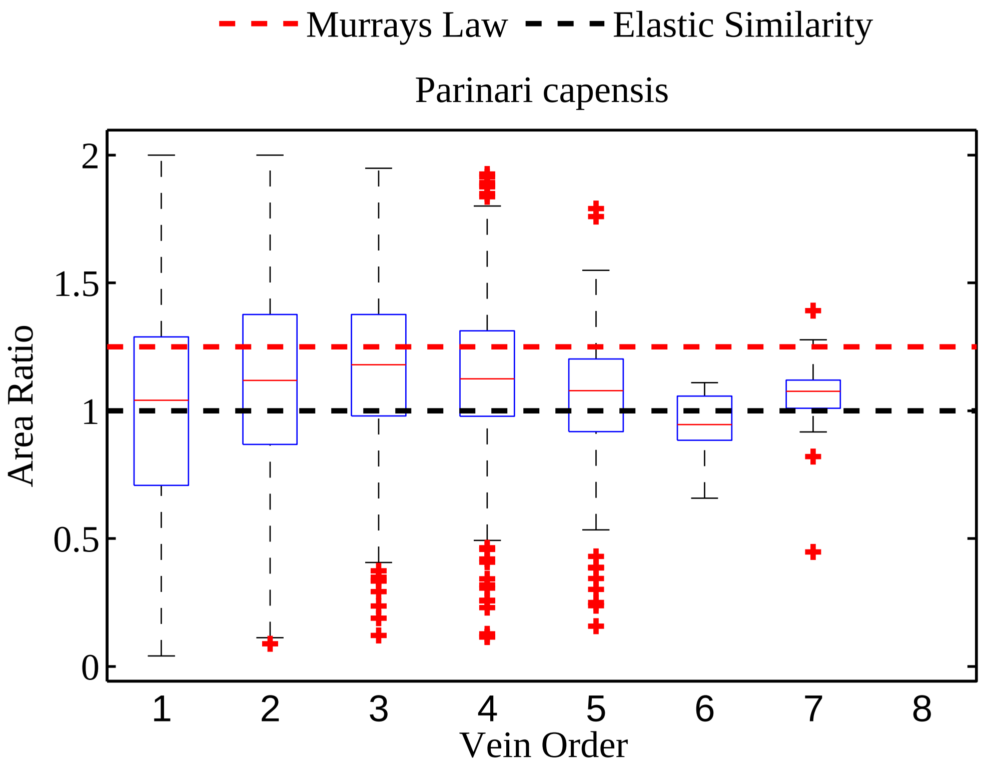

Murrays Law
Elastic Similarity
Parinari capensis
Area Ratio
Vein Order
0
0.5
1
1.5
2
1
2
3
4
5
6
7
8

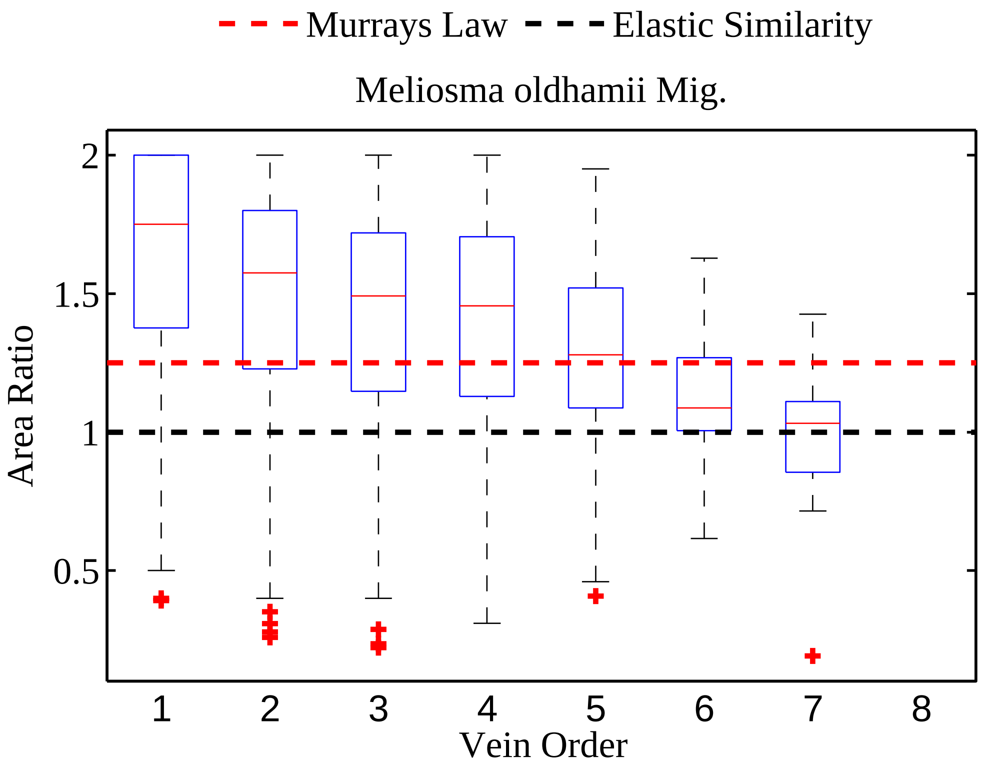

Murrays Law
Elastic Similarity
Meliosma oldhamii Mig.
Area Ratio
2
1.5
1
0.5
1
2
3
4
5
6
7
8
Vein Order

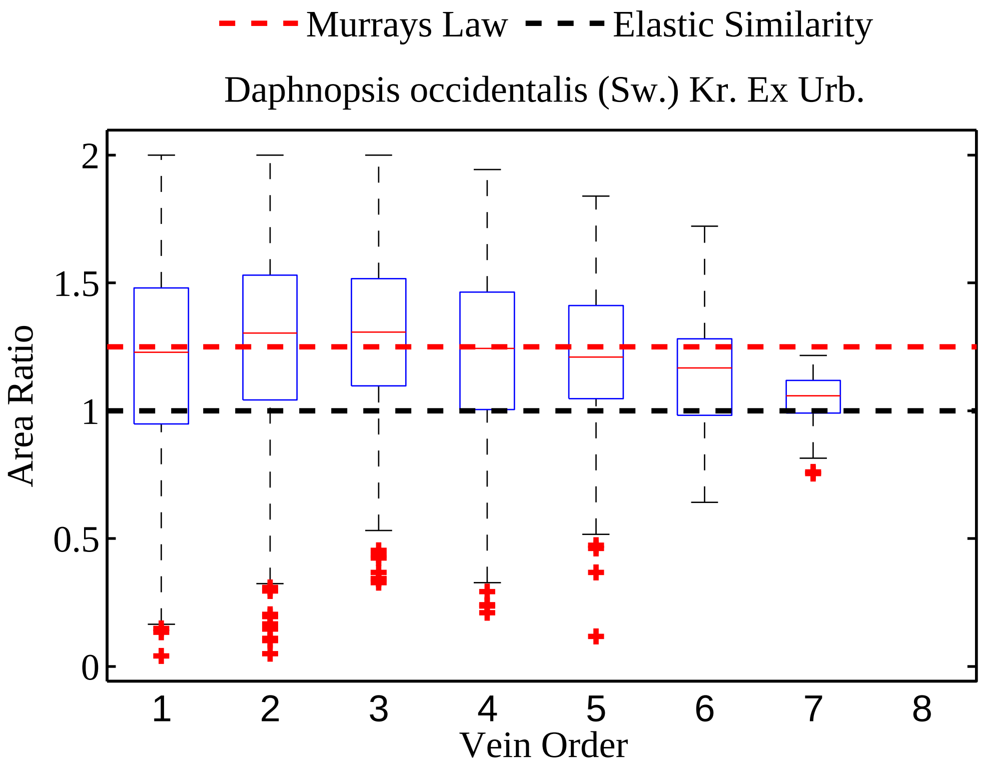

Murrays Law
Elastic Similarity
Daphnopsis occidentalis (Sw.) Kr. Ex Urb.
Area Ratio
0
0.5
1
1.5
2
1
2
3
4
5
6
7
8
Vein Order

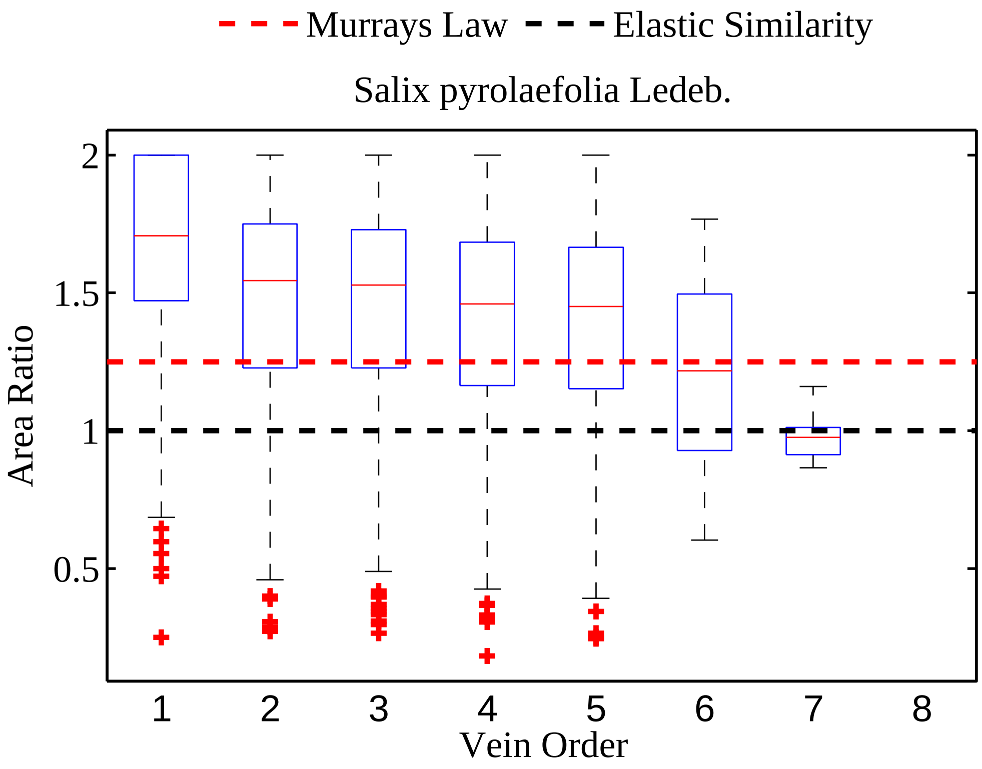

Murrays Law
Elastic Similarity
Salix pyrolaefolia Ledeb.
Area Ratio
2
1.5
1
0.5
1
2
3
4
5
6
7
8
Vein Order

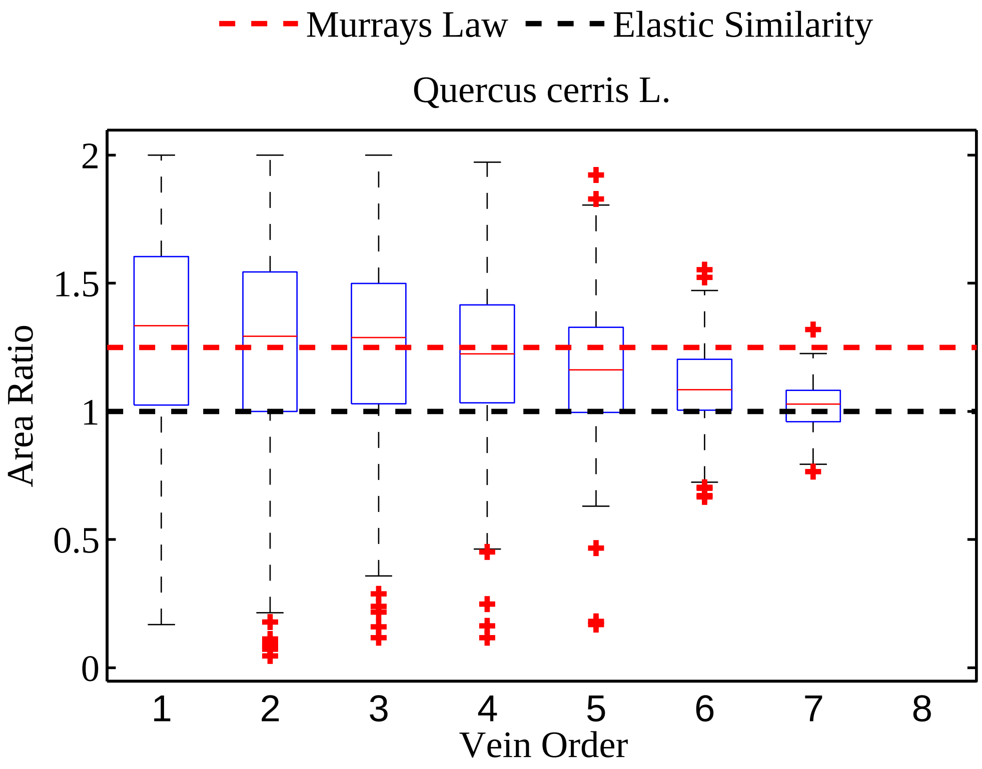
Murrays Law
Elastic Similarity
Quercus cerris L.
Area Ratio
2
1.5
1
0.5
0
1
2
3
4
5
6
7
8
Vein Order

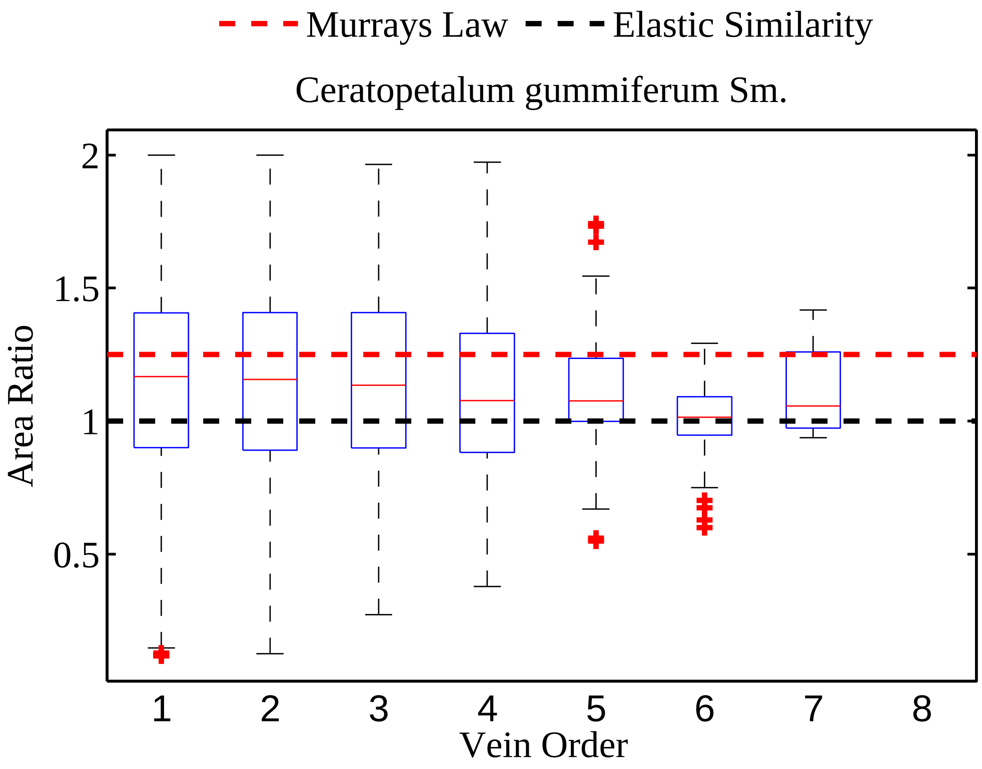

Murrays Law
Elastic Similarity
Ceratopetalum gummiferum Sm.
Area Ratio
Vein Order
2
1.5
1
0.5
1
2
3
4
5
6
7
8

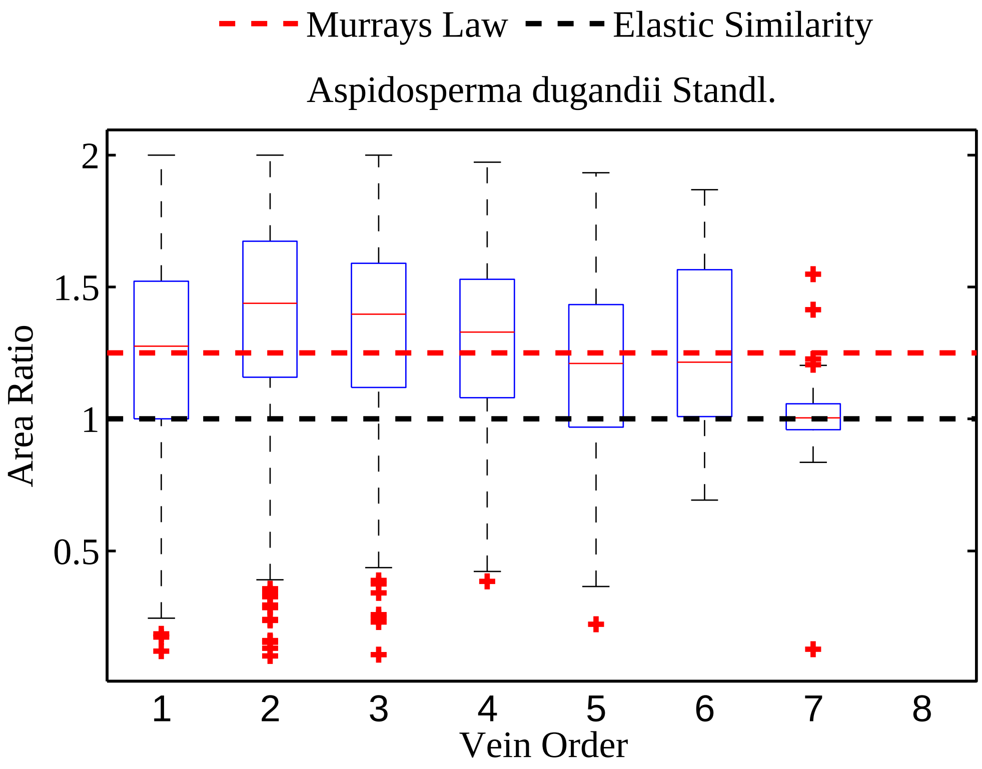

Murrays Law
Elastic Similarity
Aspidosperma dugandii Standl.
Area Ratio
Vein Order
2
1.5
1
0.5
1
2
3
4
5
6
7
8

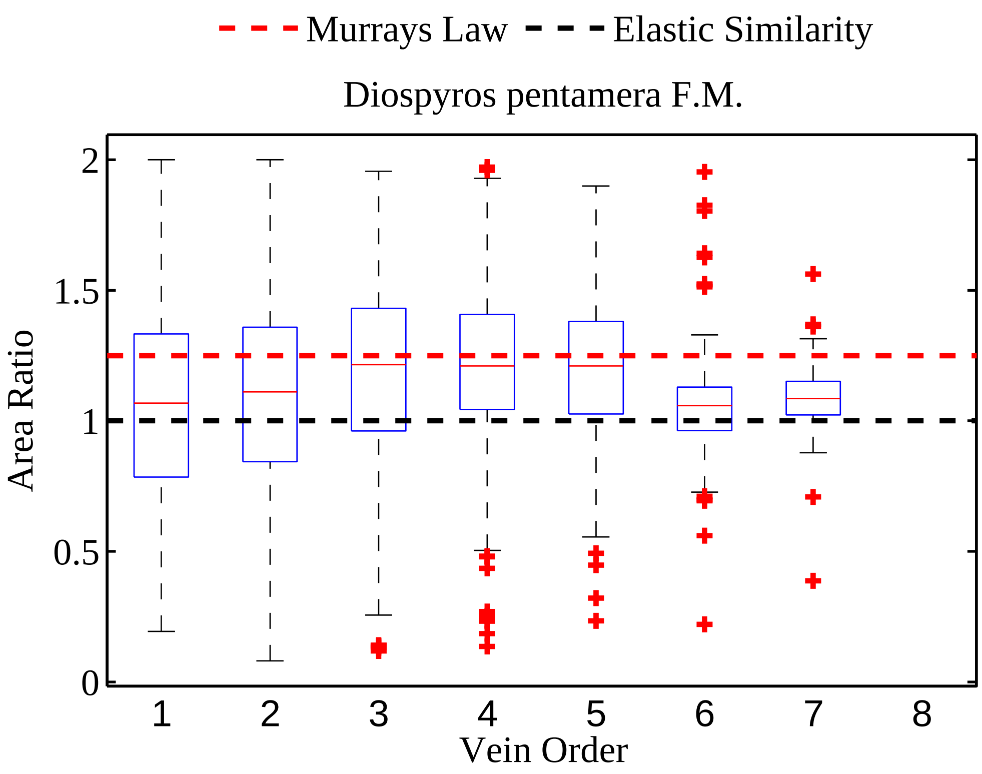
Murrays Law
Elastic Similarity
Diospyros pentamera F.M.
Area Ratio
0
0.5
1
1.5
2
1
2
3
4
5
6
7
8
Vein Order

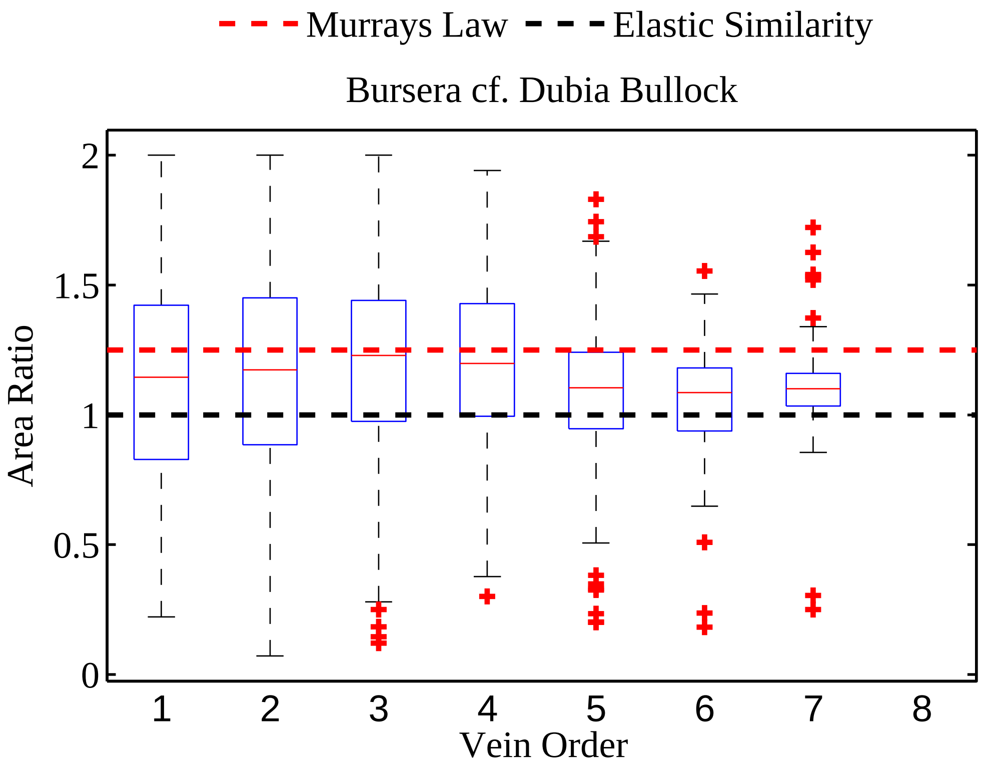

Murrays Law
Elastic Similarity
Bursera cf. Dubia Bullock
Area Ratio
0
0.5
1
1.5
2
1
2
3
4
5
6
7
8
Vein Order

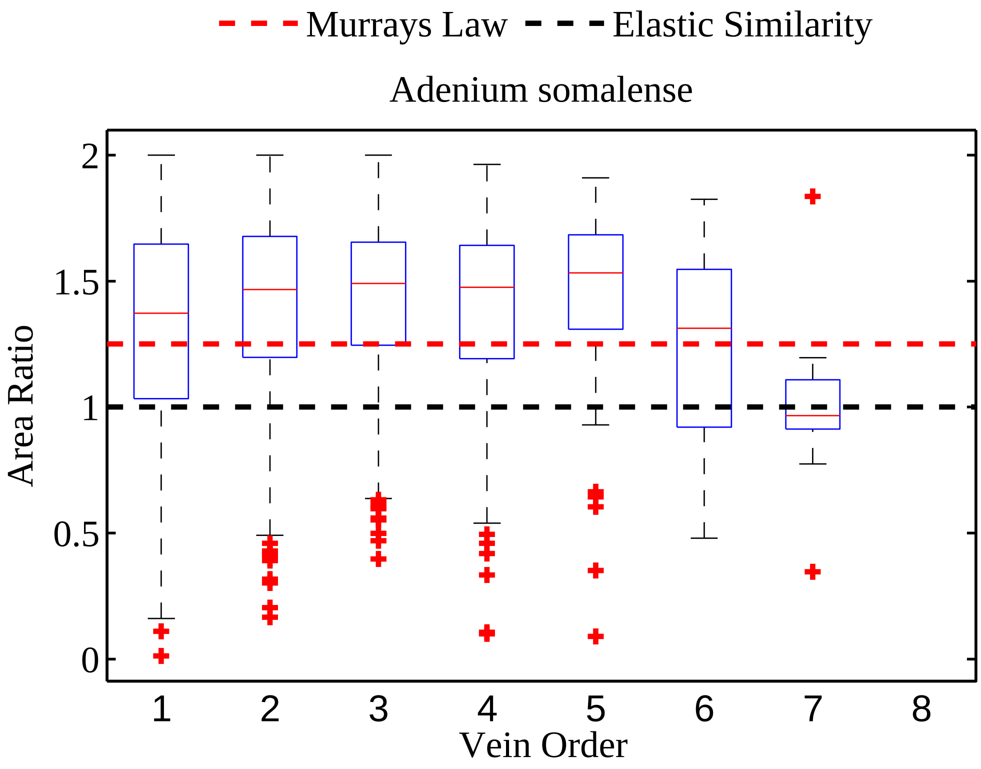

Murrays Law
Elastic Similarity
Adenium somalense
Area Ratio
Vein Order
0
0.5
1
1.5
2
1
2
3
4
5
6
7
8

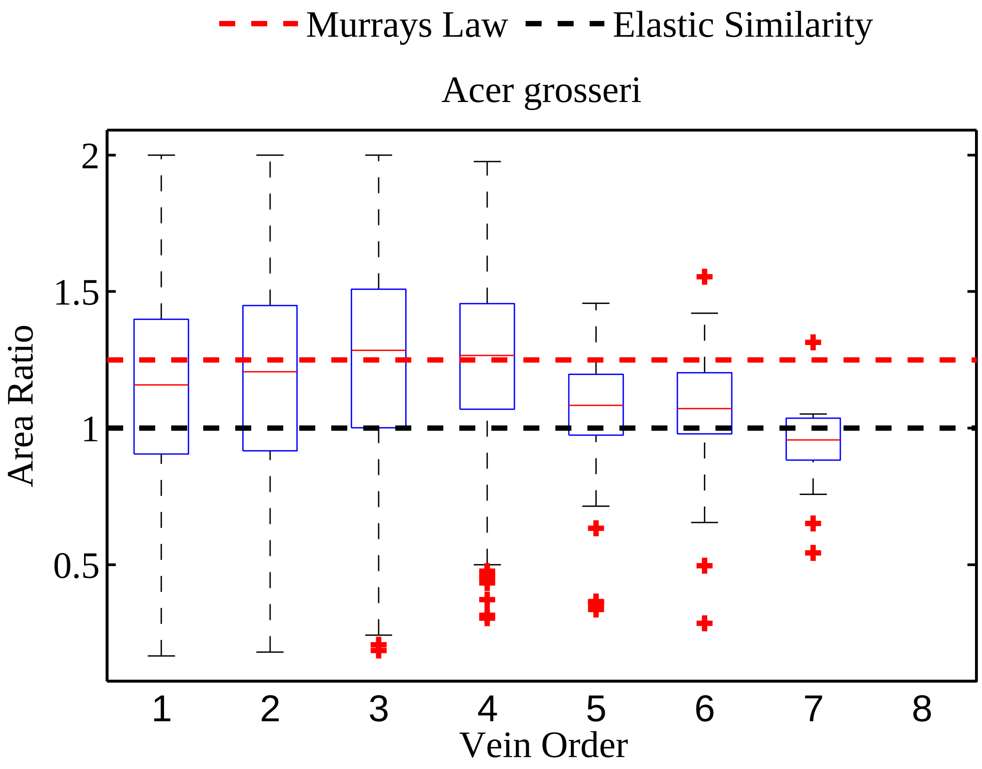

Murrays Law
Elastic Similarity
Acer grosseri
Area Ratio
2
1.5
1
0.5
1
2
3
4
5
6
7
8
Vein Order

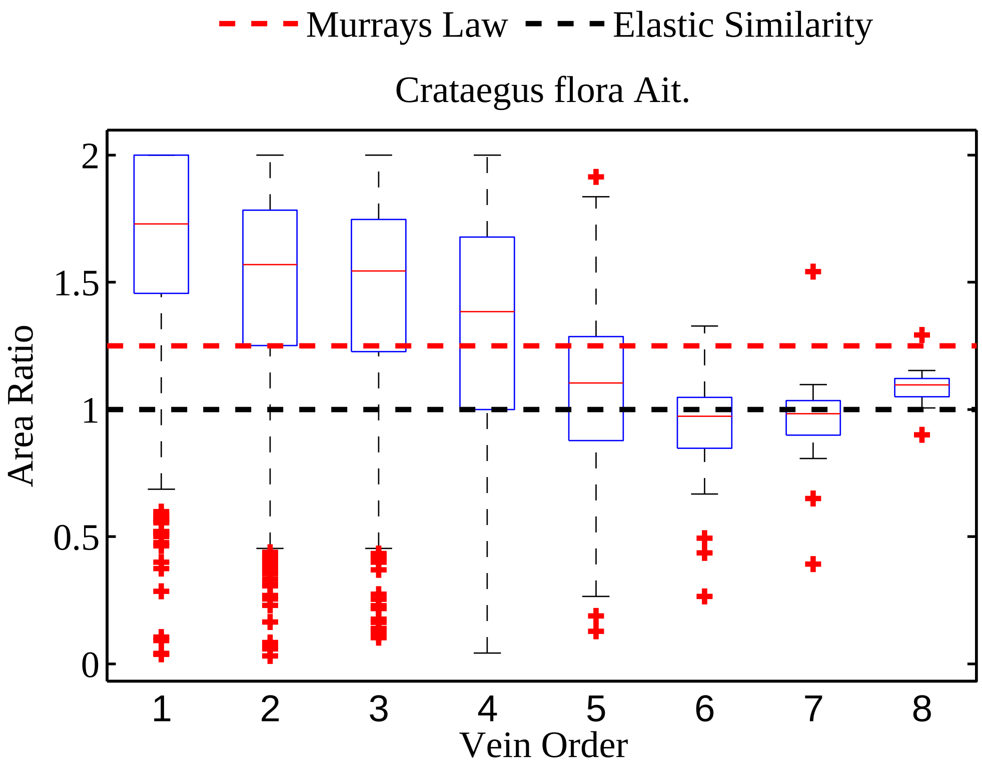

Murrays Law
Elastic Similarity
Crataegus flora Ait.
Area Ratio
Vein Order
0
0.5
1
1.5
2
1
2
3
4
5
6
7
8

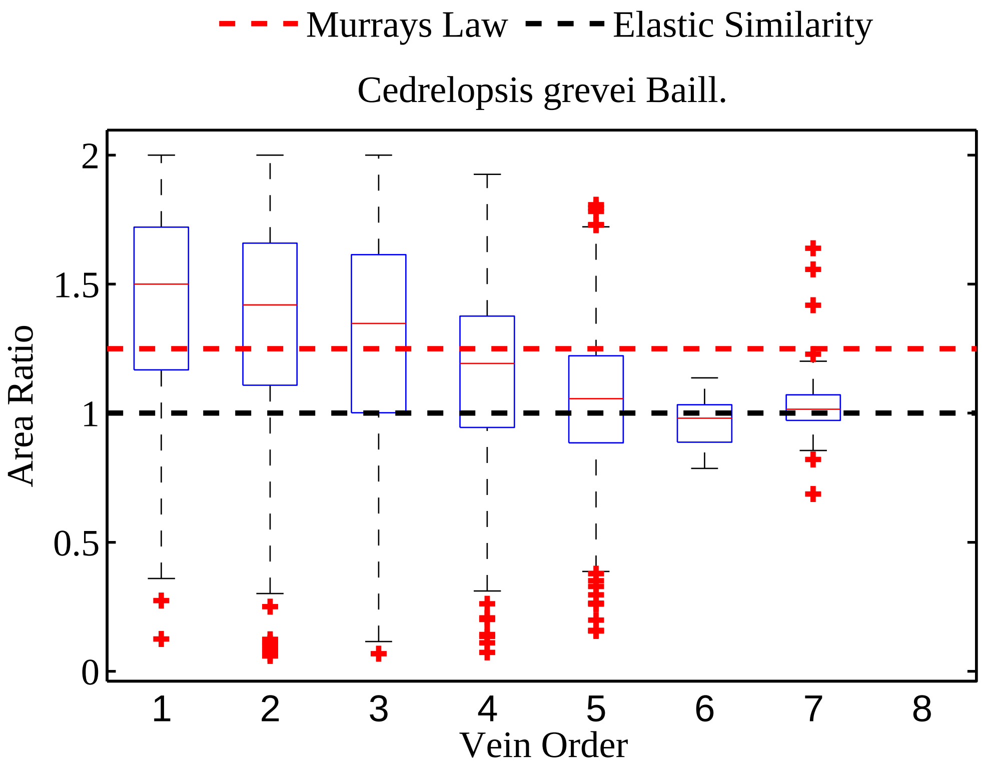

Murrays Law
Elastic Similarity
Cedrelopsis grevei Baill.
Area Ratio
0
0.5
1
1.5
2
1
2
3
4
5
6
7
8
Vein Order

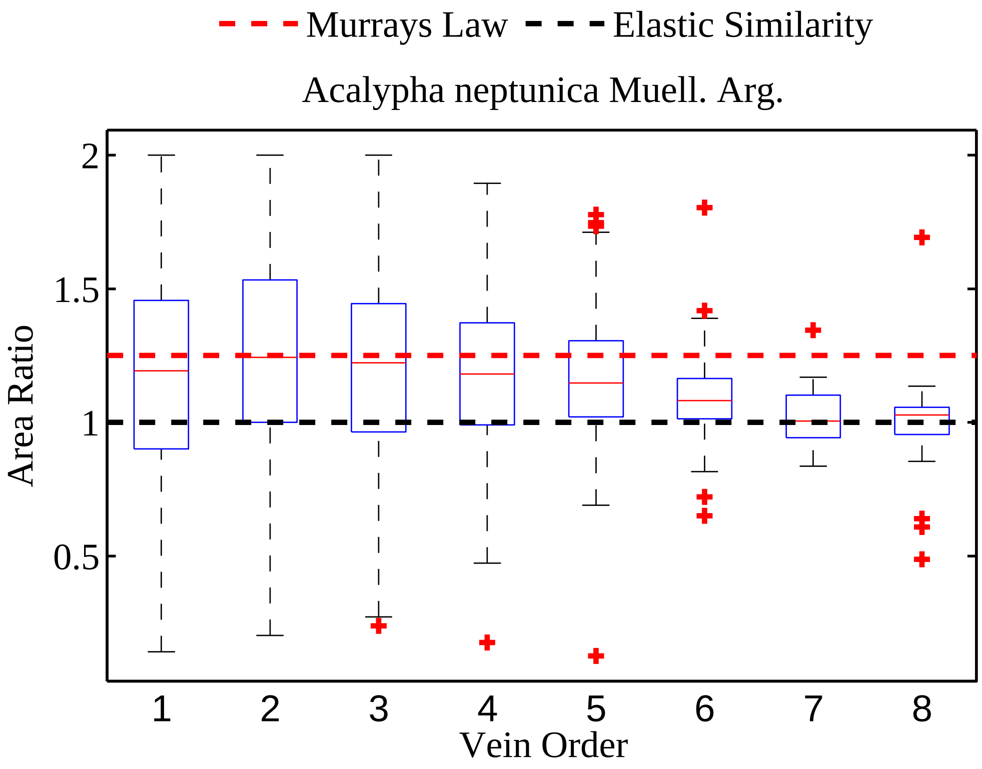

Murrays Law
Elastic Similarity
Acalypha neptunica Muell. Arg.
Area Ratio
2
1.5
1
0.5
1
2
3
4
5
6
7
8
Vein Order

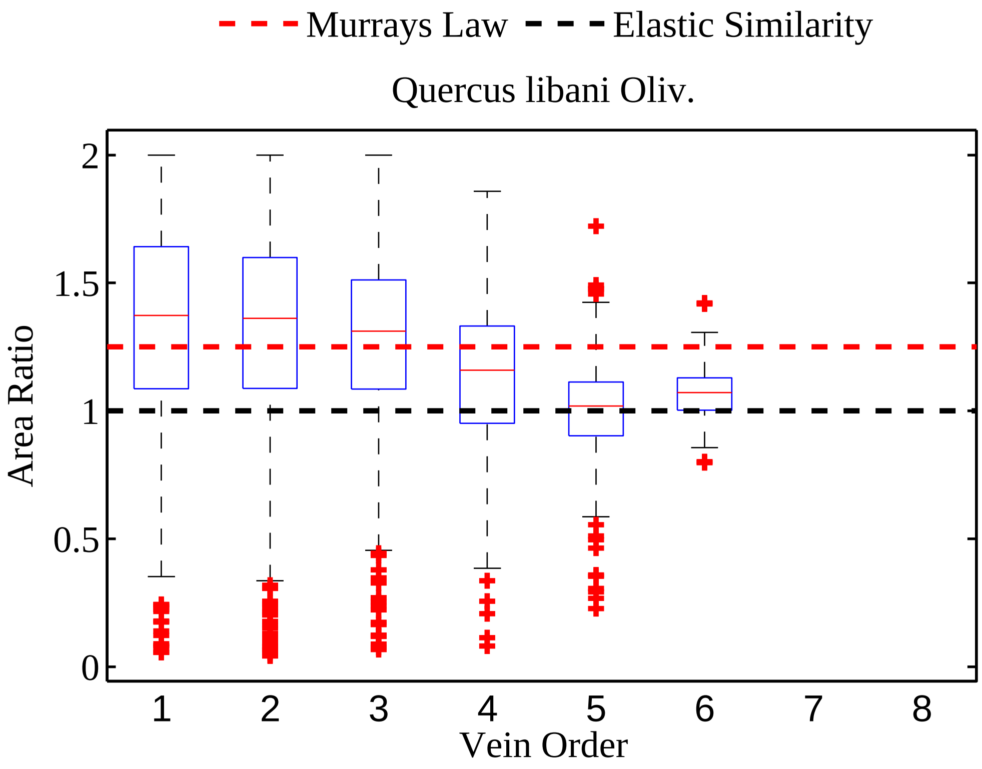

Murrays Law
Elastic Similarity
Quercus libani Oliv.
Area Ratio
Vein Order
0
0.5
1
1.5
2
1
2
3
4
5
6
7
8

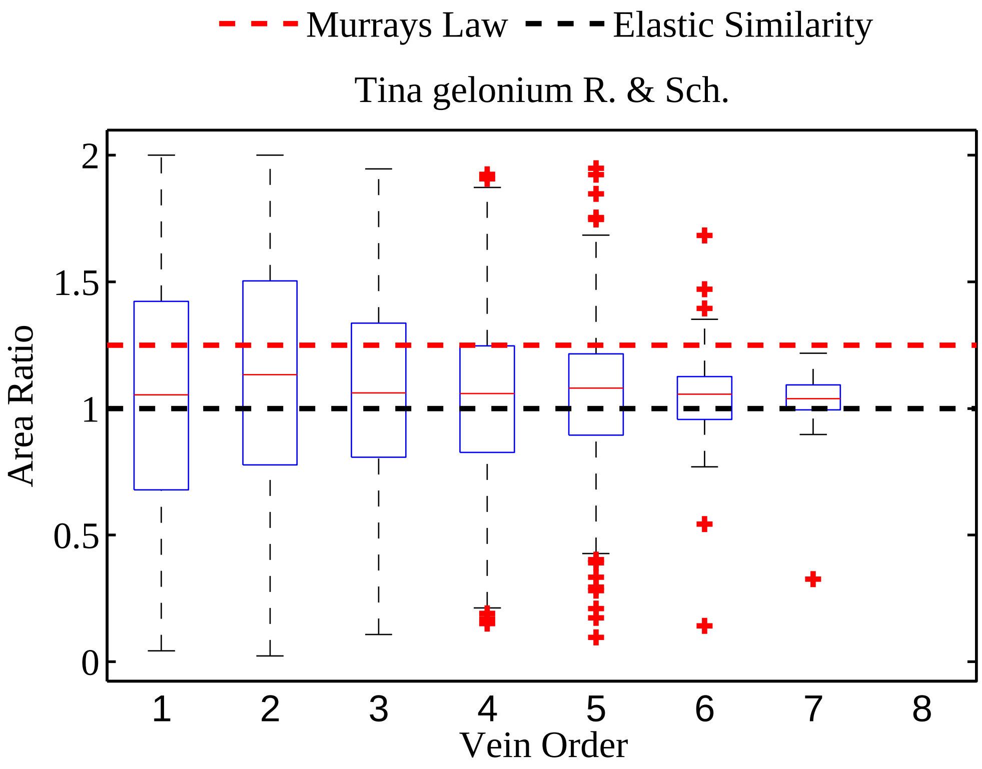

Murrays Law
Elastic Similarity
Tina gelonium R. & Sch.
Area Ratio
0
0.5
1
1.5
2
1
2
3
4
5
6
7
8
Vein Order

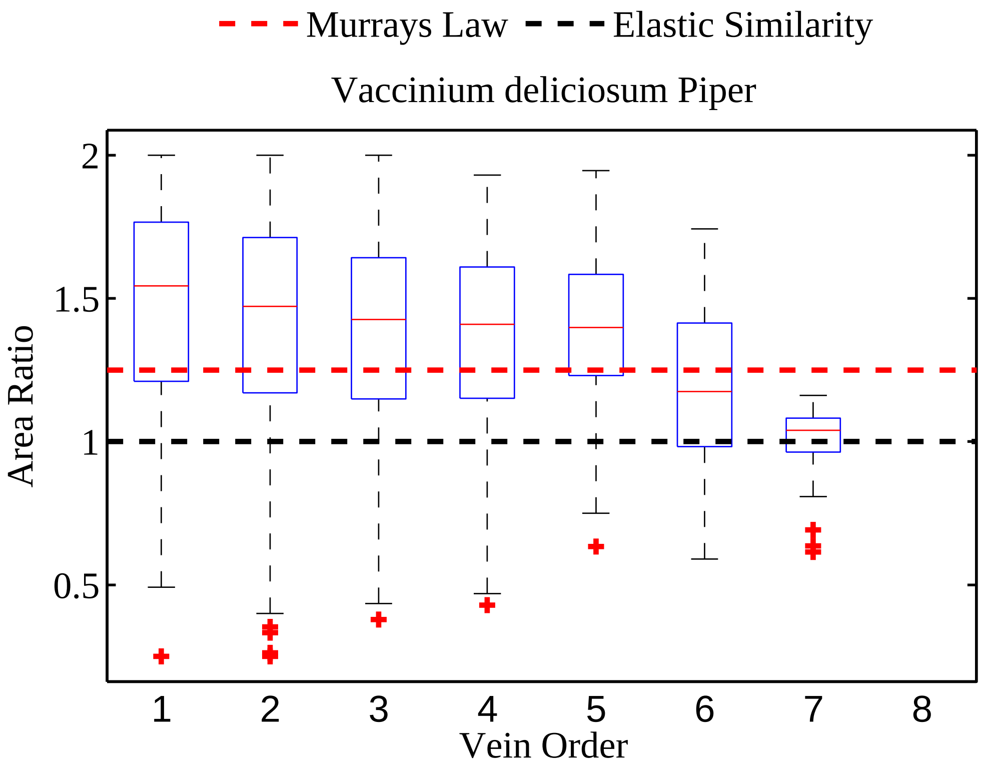

Murrays Law
Elastic Similarity
Vaccinium deliciosum Piper
Area Ratio
2
1.5
1
0.5
1
2
3
4
5
6
7
8
Vein Order

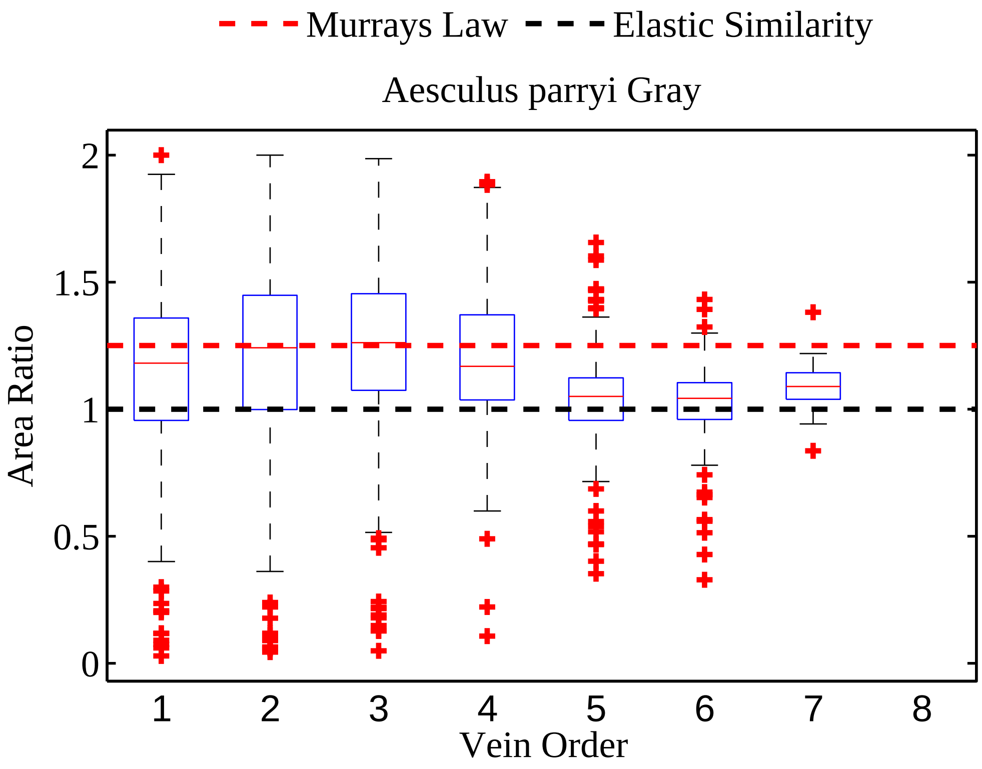

Murrays Law
Elastic Similarity
Aesculus parryi Gray
Area Ratio
0
0.5
1
1.5
2
1
2
3
4
5
6
7
8
Vein Order

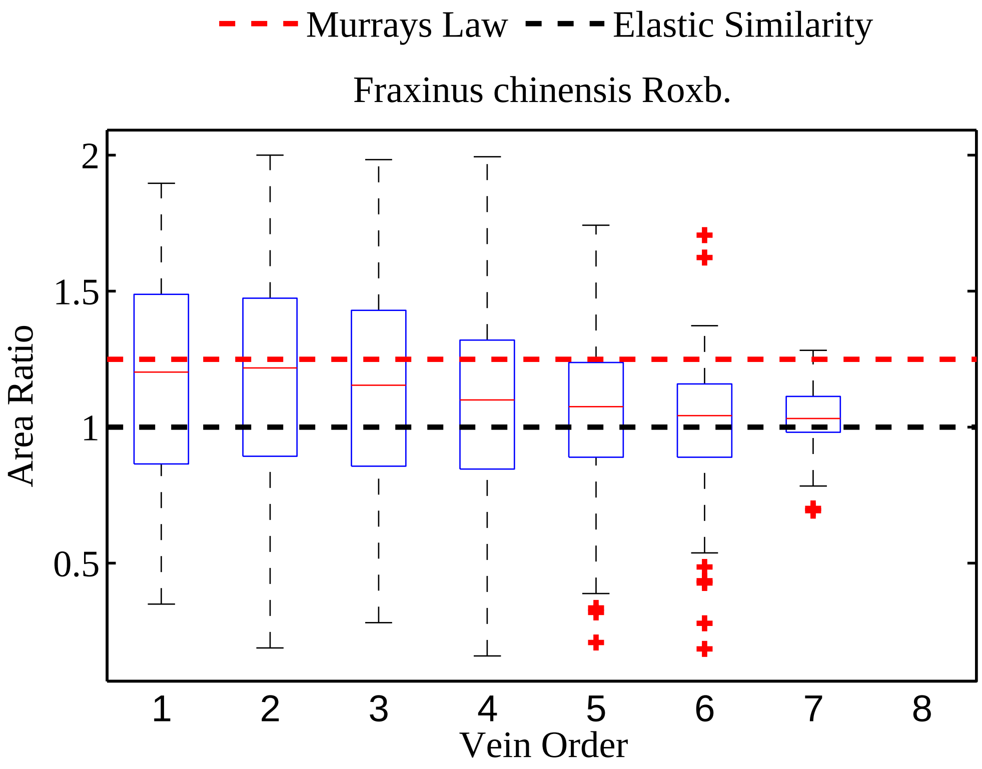

Murrays Law
Elastic Similarity
Fraxinus chinensis Roxb.
Area Ratio
2
1.5
1
0.5
1
2
3
4
5
6
7
8
Vein Order

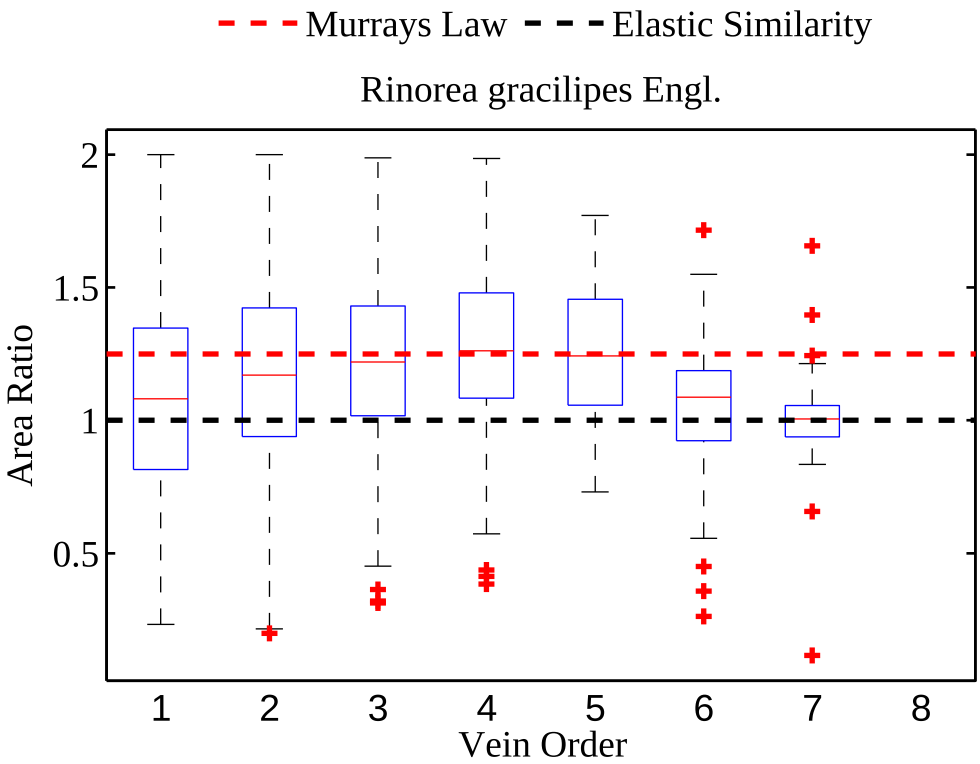
Murrays Law
Elastic Similarity
Rinorea gracilipes Engl.
Area Ratio
2
1.5
1
0.5
1
2
3
4
5
6
7
8
Vein Order

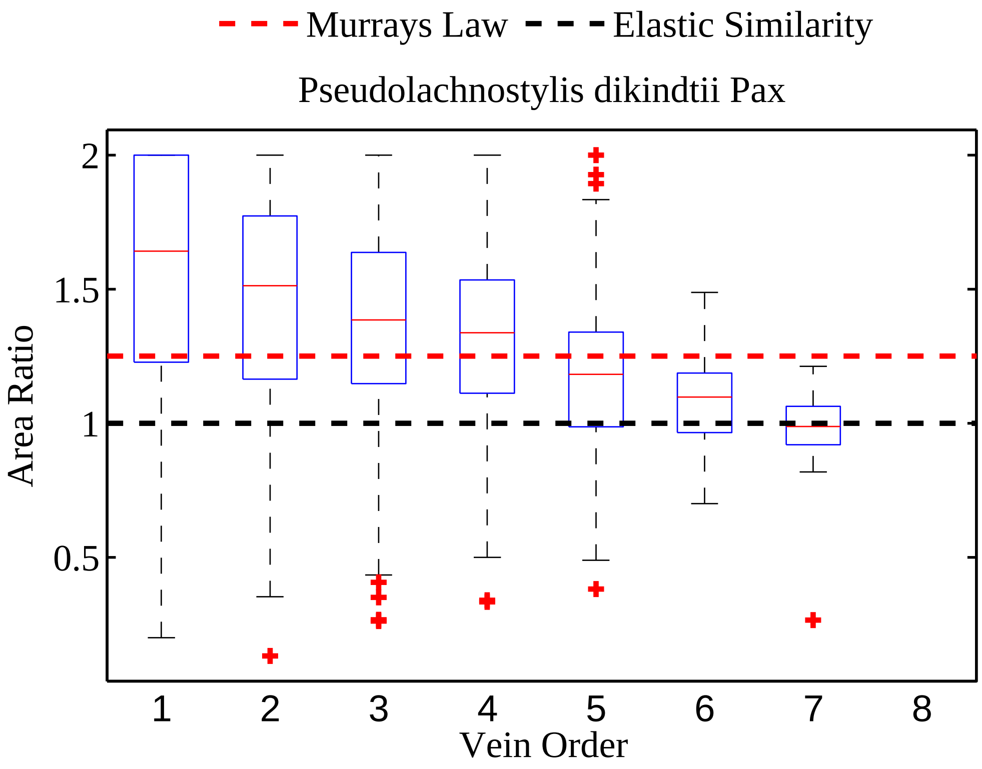

Murrays Law
Elastic Similarity
Pseudolachnostylis dikindtii Pax
Area Ratio
2
1.5
1
0.5
1
2
3
4
5
6
7
8
Vein Order

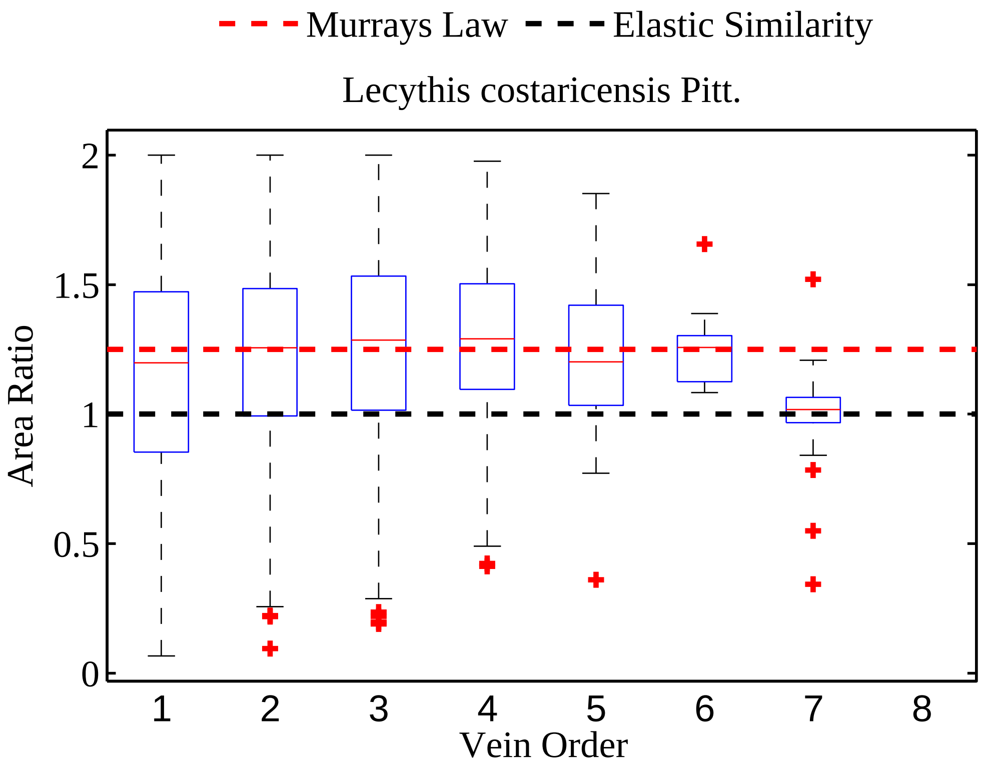

Murrays Law
Elastic Similarity
Lecythis costaricensis Pitt.
Area Ratio
0
0.5
1
1.5
2
1
2
3
4
5
6
7
8
Vein Order

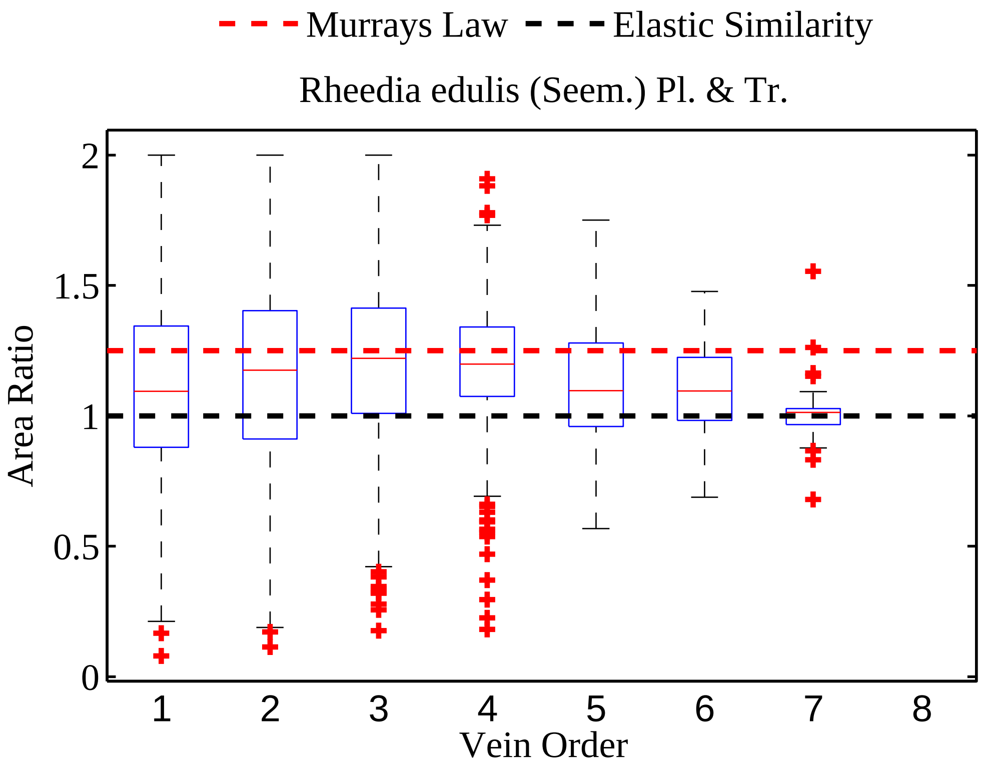

Murrays Law
Elastic Similarity
Rheedia edulis (Seem.) Pl. & Tr.
Area Ratio
0
0.5
1
1.5
2
1
2
3
4
5
6
7
8
Vein Order

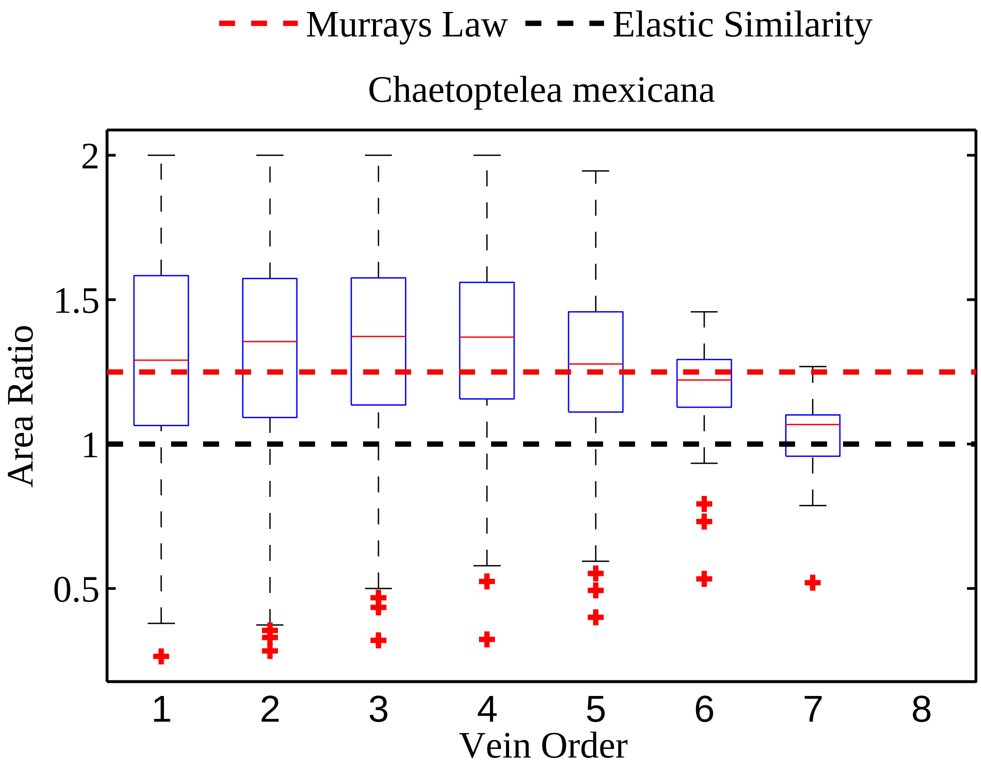
Murrays Law
Elastic Similarity
Chaetoptelea mexicana
Area Ratio
2
1.5
1
0.5
1
2
3
4
5
6
7
8
Vein Order

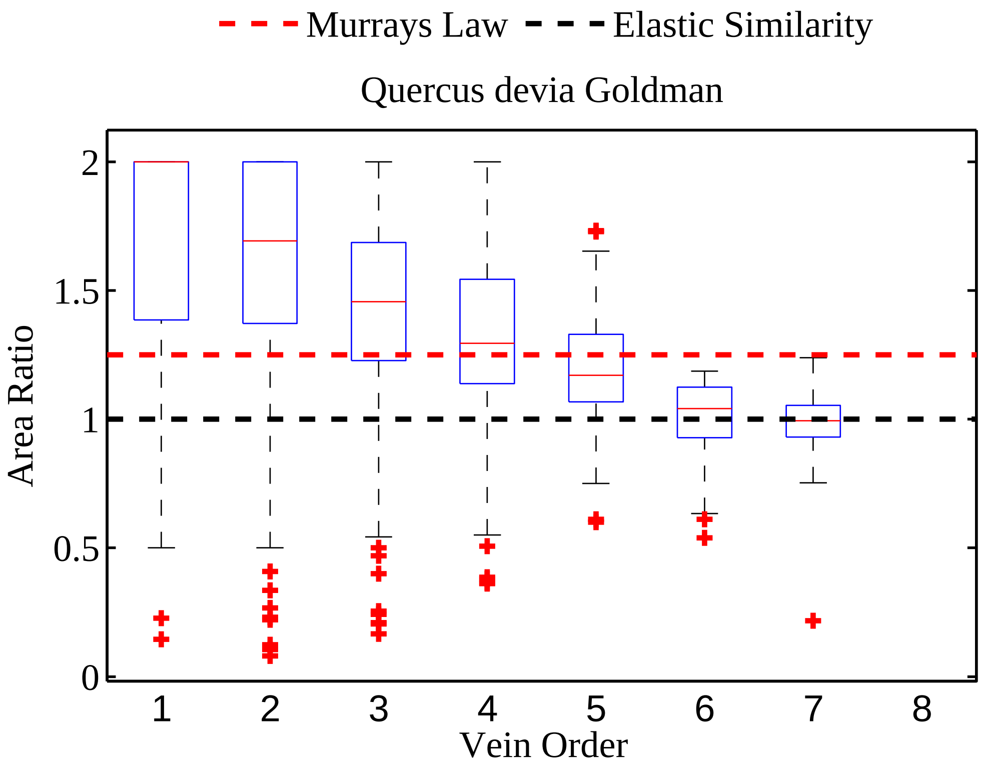

Murrays Law
Elastic Similarity
Quercus devia Goldman
Area Ratio
Vein Order
0
0.5
1
1.5
2
1
2
3
4
5
6
7
8

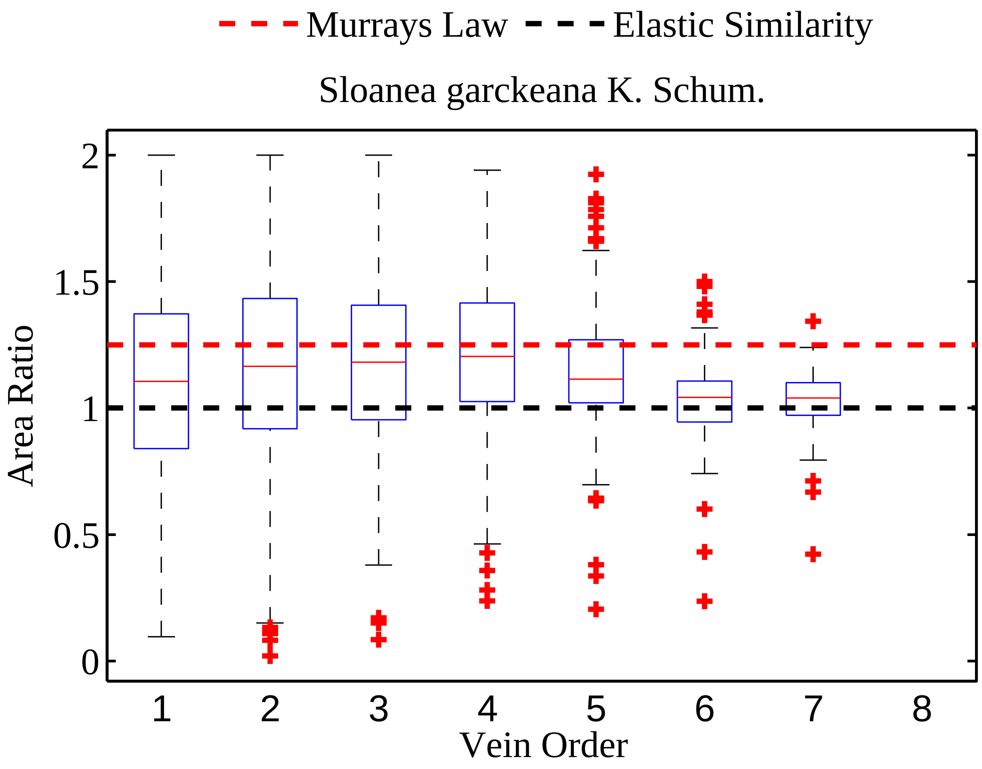

Murrays Law
Elastic Similarity
Sloanea garckeana K. Schum.
Area Ratio
Vein Order
0
0.5
1
1.5
2
1
2
3
4
5
6
7
8

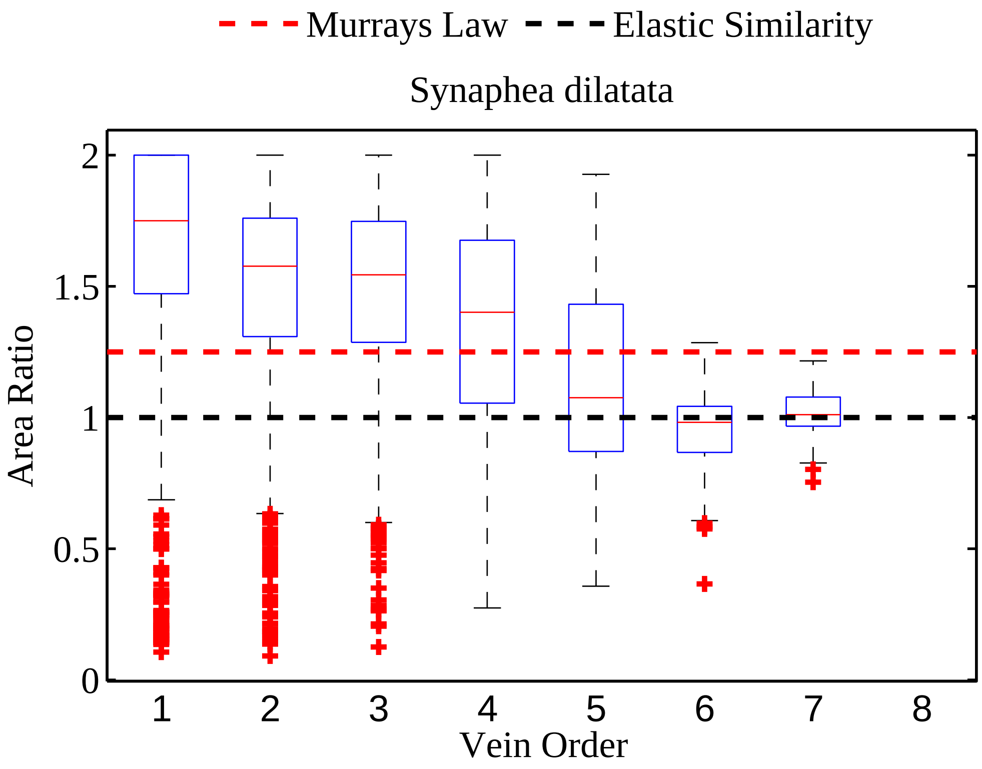

Murrays Law
Elastic Similarity
Synaphea dilatata
Area Ratio
Vein Order
0
0.5
1
1.5
2
1
2
3
4
5
6
7
8

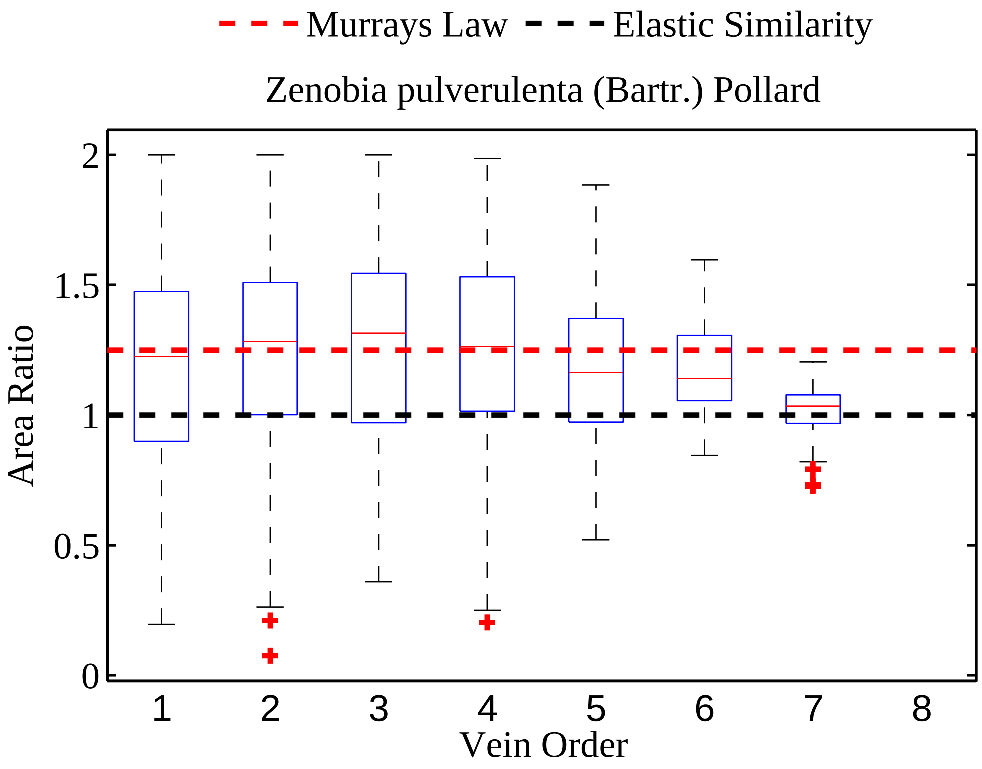

Murrays Law
Elastic Similarity
Zenobia pulverulenta (Bartr.) Pollard
Area Ratio
Vein Order
0
0.5
1
1.5
2
1
2
3
4
5
6
7
8

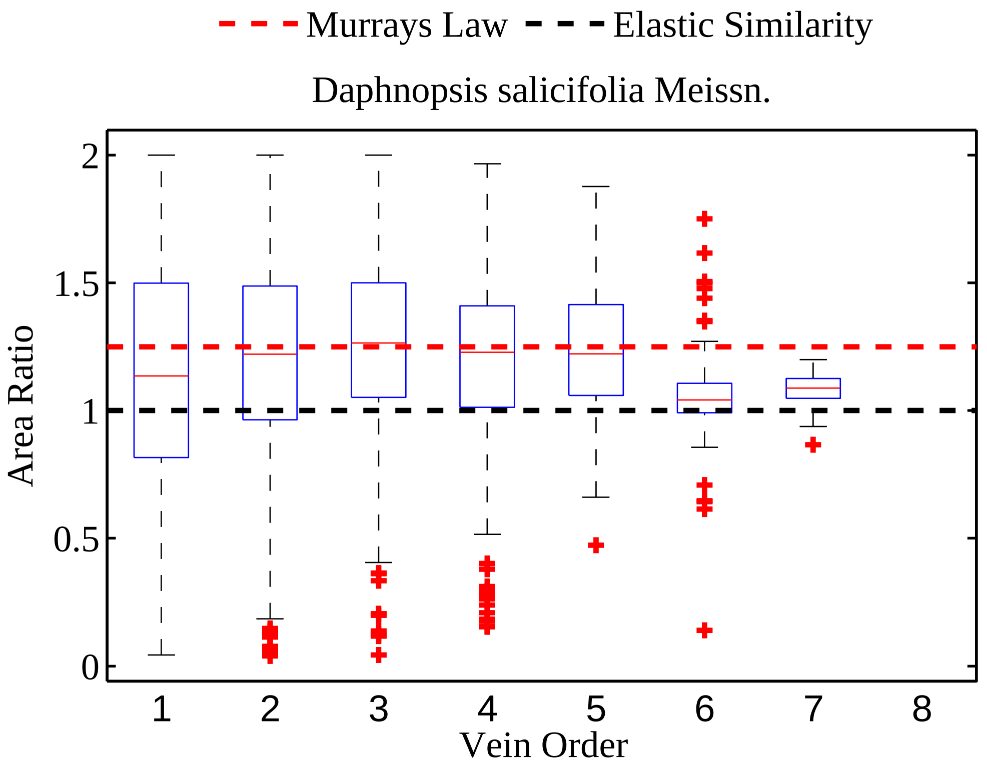

Murrays Law
Elastic Similarity
Daphnopsis salicifolia Meissn.
Area Ratio
Vein Order
0
0.5
1
1.5
2
1
2
3
4
5
6
7
8

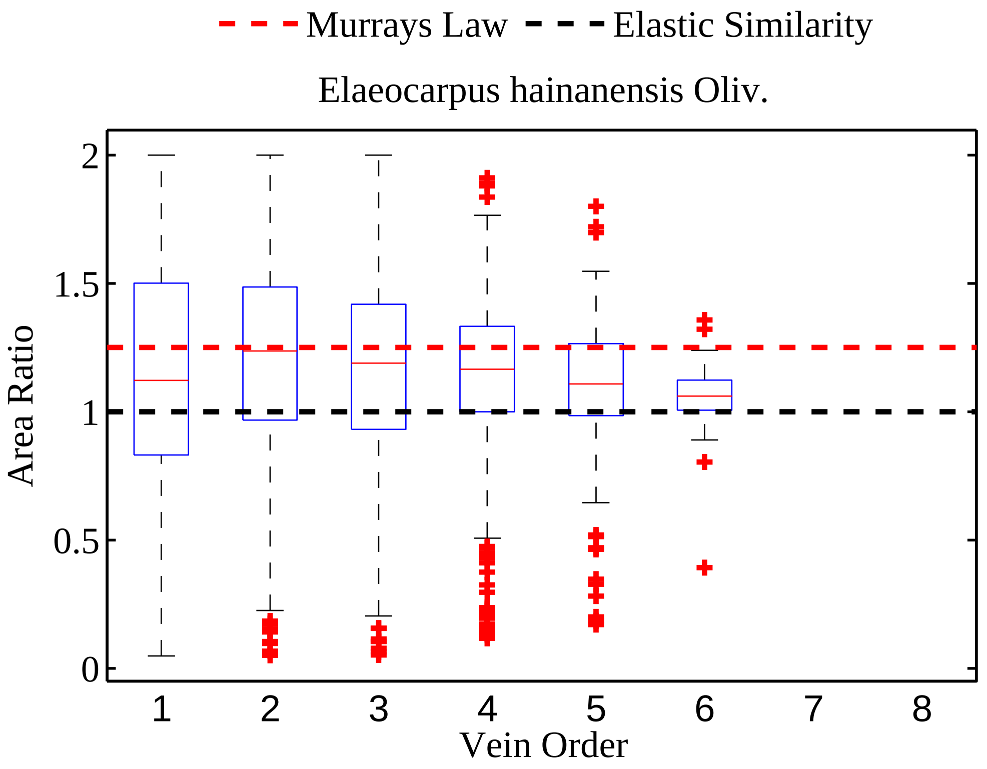

Murrays Law
Elastic Similarity
Elaeocarpus hainanensis Oliv.
Area Ratio
2
1.5
1
0.5
0
1
2
3
4
5
6
7
8
Vein Order

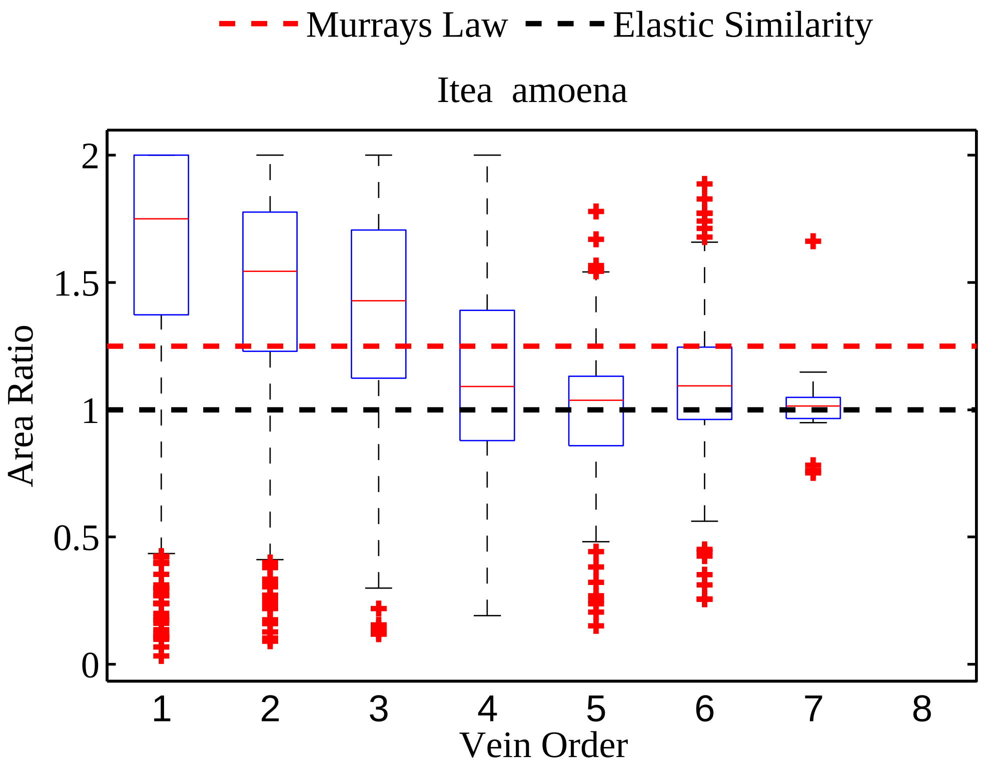

Murrays Law
Elastic Similarity
Itea amoena
Area Ratio
0
0.5
1
1.5
2
1
2
3
4
5
6
7
8
Vein Order

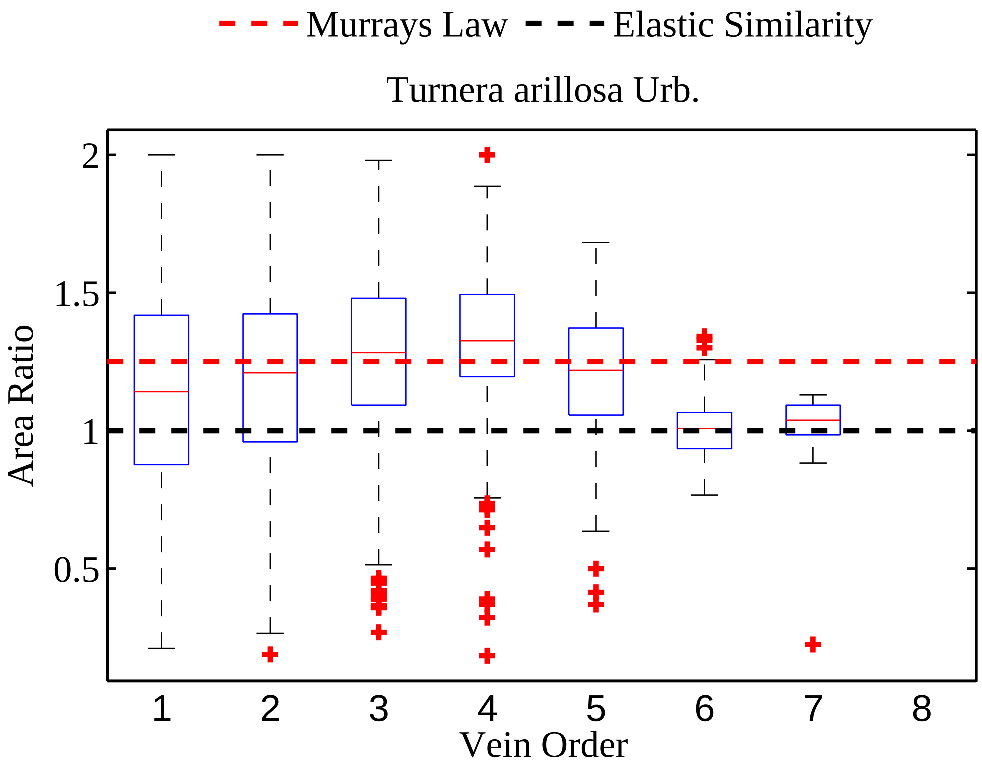

Murrays Law
Elastic Similarity
Turnera arillosa Urb.
Area Ratio
2
1.5
1
0.5
1
2
3
4
5
6
7
8
Vein Order

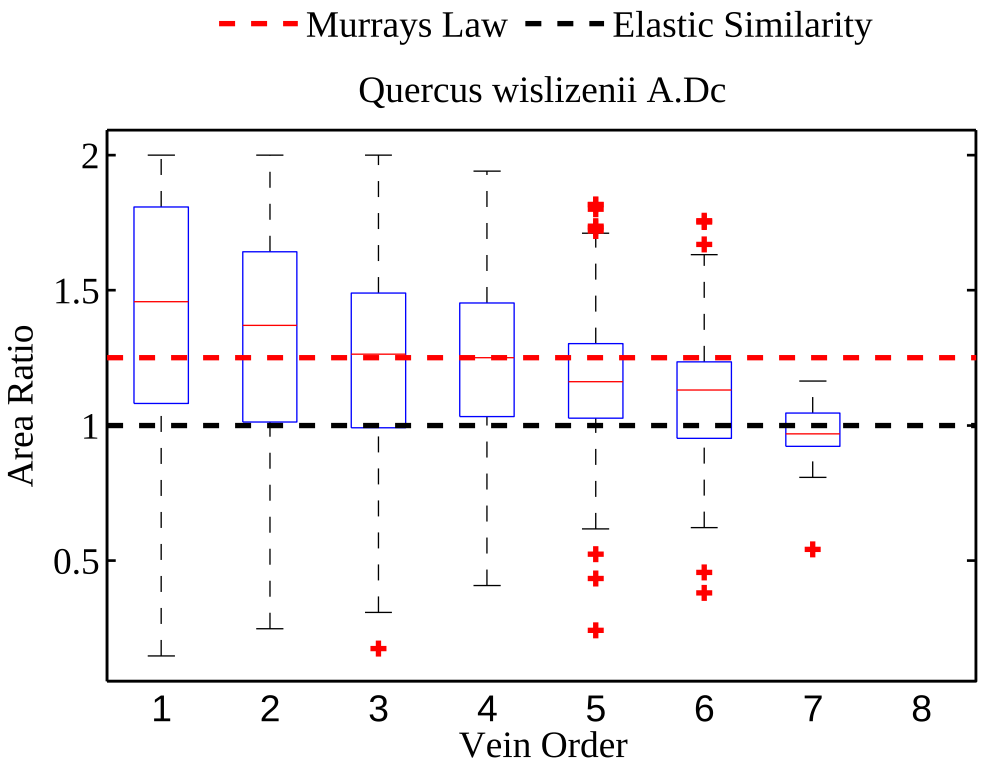

Murrays Law
Elastic Similarity
Quercus wislizenii A.Dc
Area Ratio
2
1.5
1
0.5
1
2
3
4
5
6
7
8
Vein Order

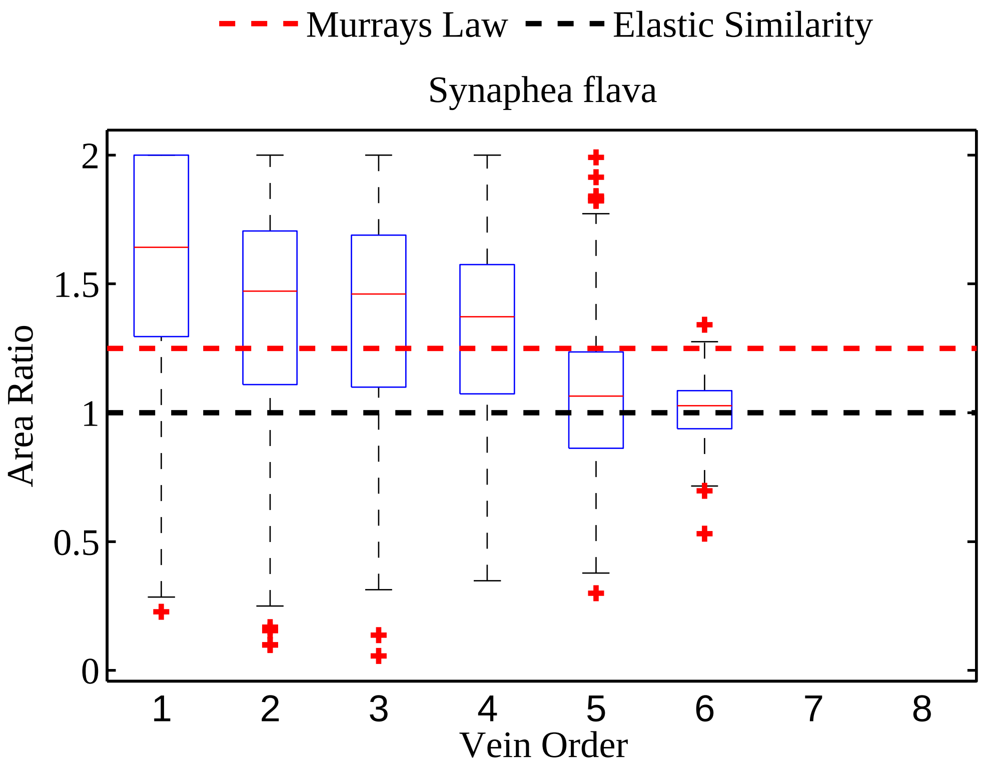

Murrays Law
Elastic Similarity
Synaphea flava
Area Ratio
0
0.5
1
1.5
2
1
2
3
4
5
6
7
8
Vein Order

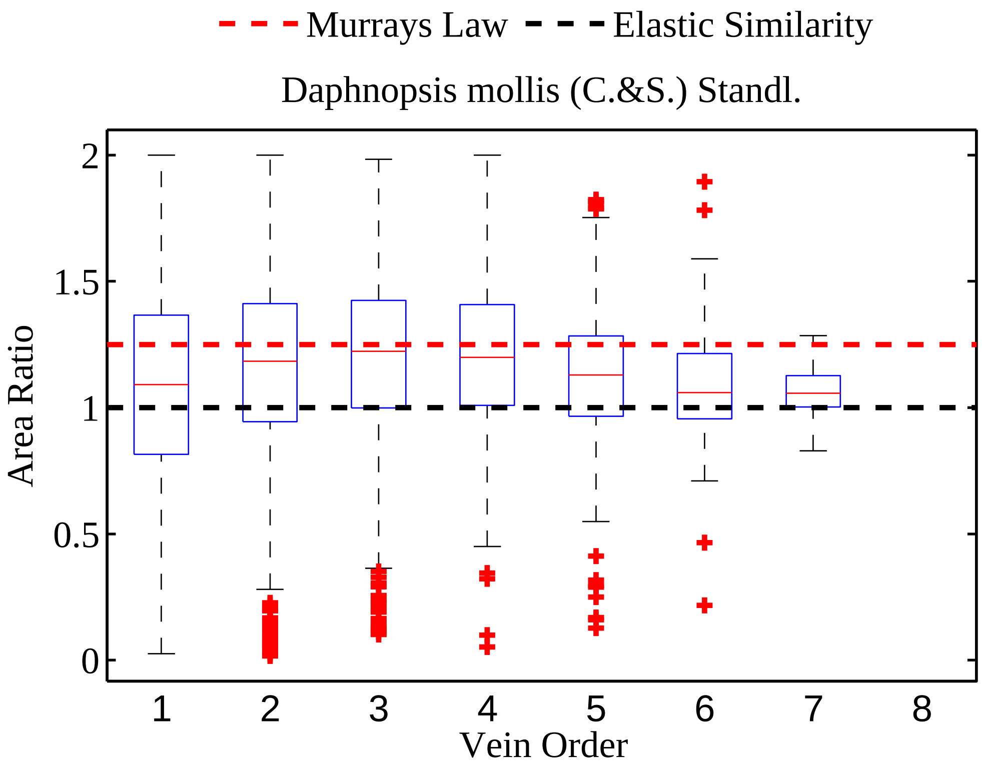
Murrays Law
Elastic Similarity
Daphnopsis mollis (C.&S.) Standl.
Area Ratio
2
1.5
1
0.5
0
1
2
3
4
5
6
7
8
Vein Order

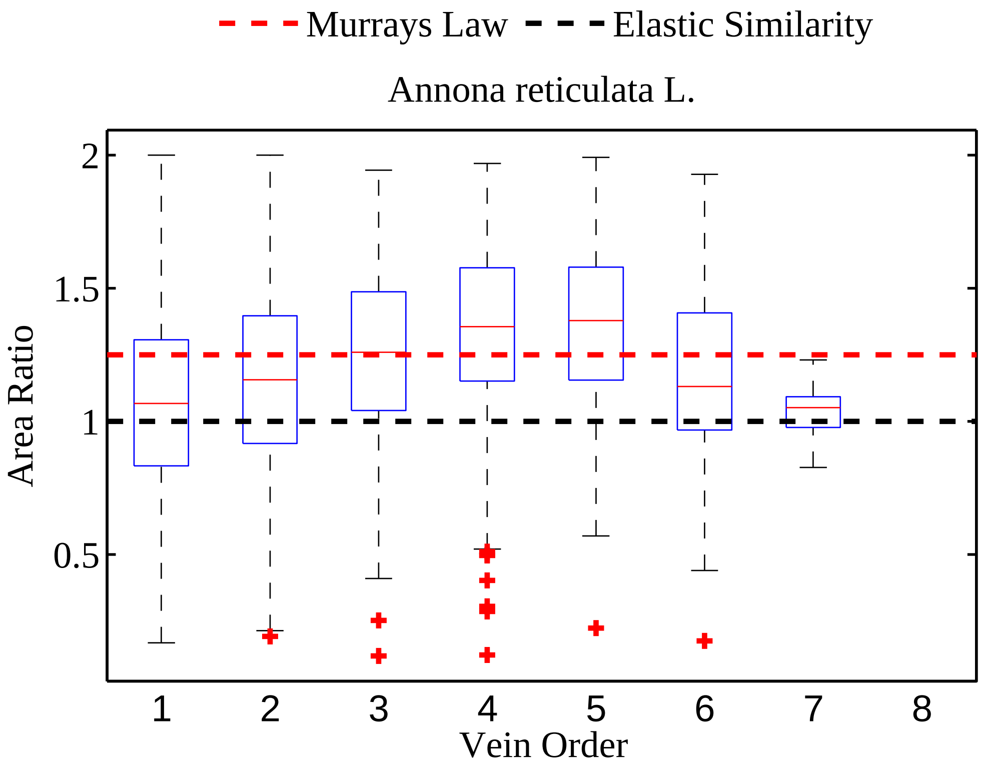

Murrays Law
Elastic Similarity
Annona reticulata L.
Area Ratio
Vein Order
2
1.5
1
0.5
1
2
3
4
5
6
7
8

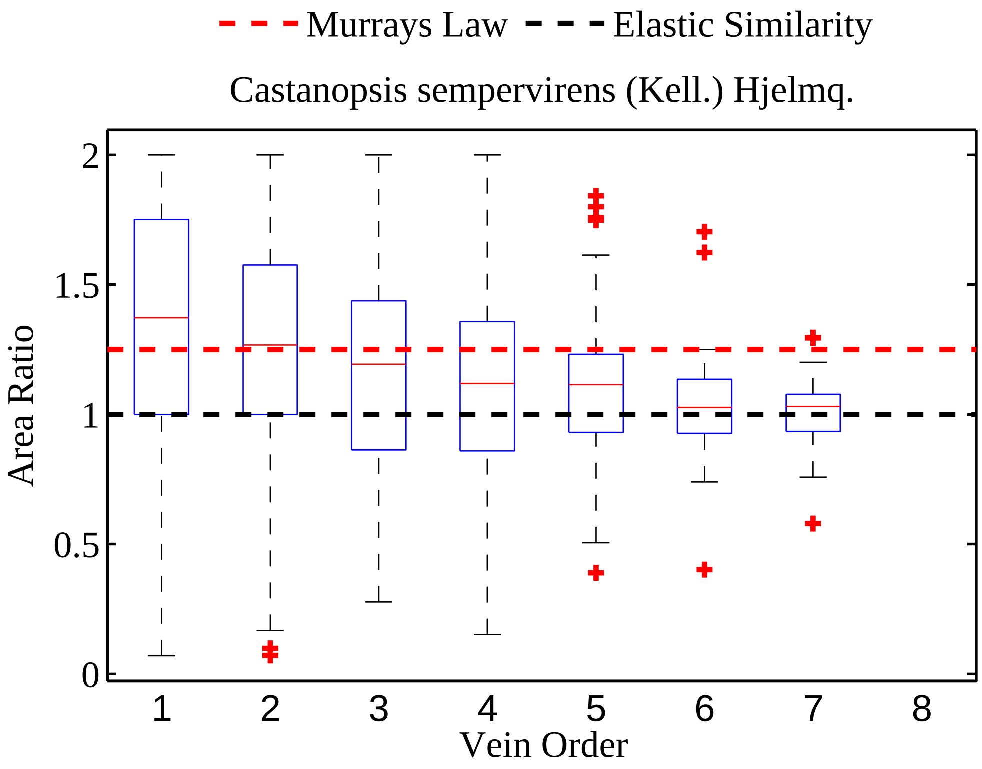
Murrays Law
Elastic Similarity
Castanopsis sempervirens (Kell.) Hjelmq.
Area Ratio
0
0.5
1
1.5
2
1
2
3
4
5
6
7
8
Vein Order

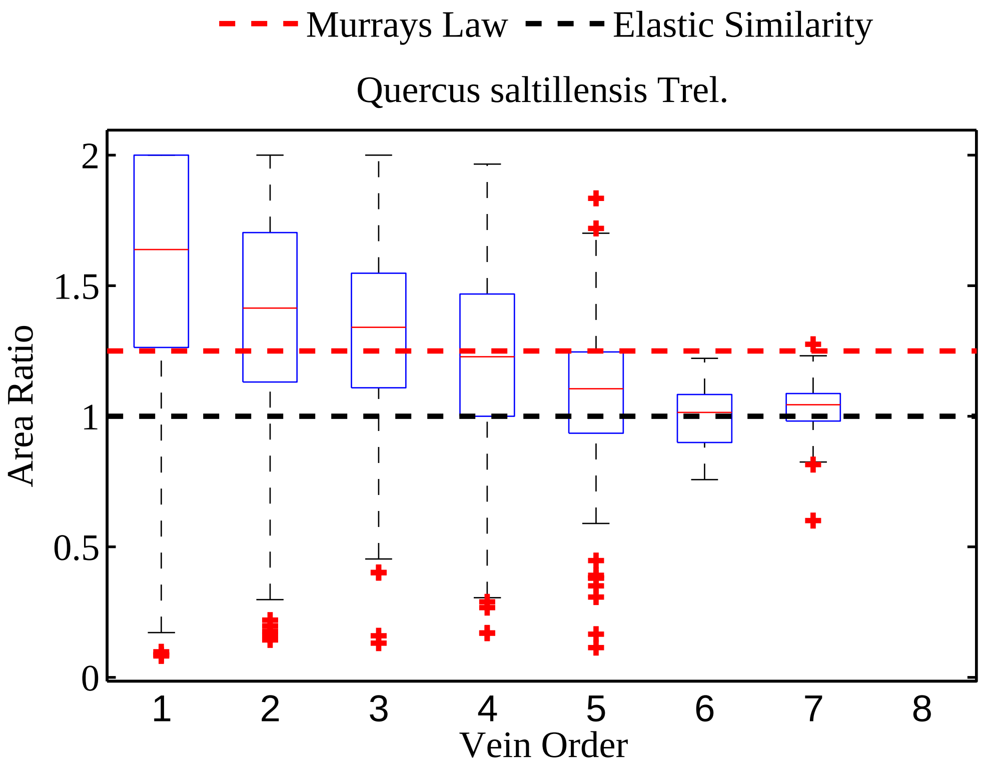

Murrays Law
Elastic Similarity
Quercus saltillensis Trel.
Area Ratio
0
0.5
1
1.5
2
1
2
3
4
5
6
7
8
Vein Order

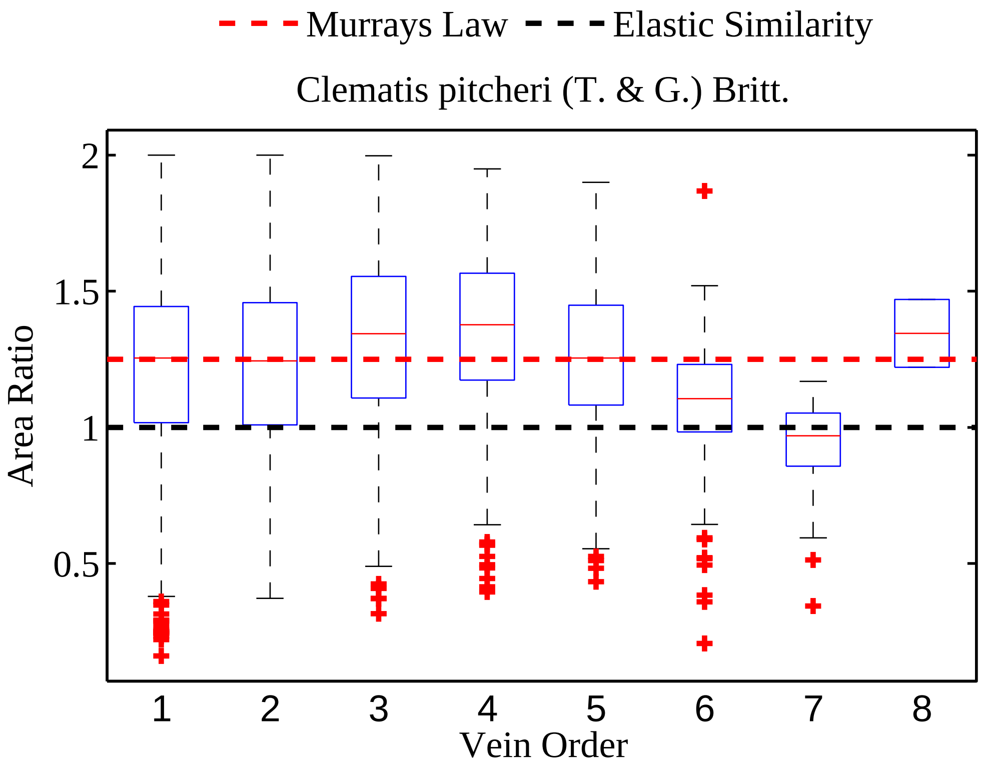

Murrays Law
Elastic Similarity
Clematis pitcheri (T. & G.) Britt.
Area Ratio
2
1.5
1
0.5
1
2
3
4
5
6
7
8
Vein Order

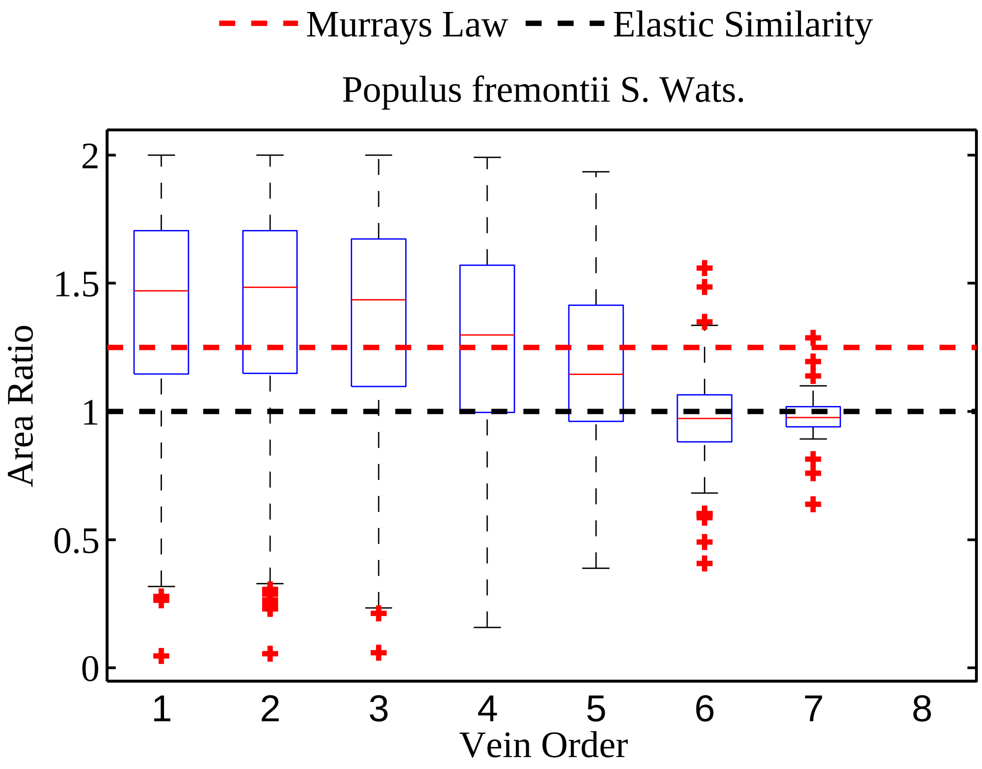
Murrays Law
Elastic Similarity
Populus fremontii S. Wats.
Area Ratio
2
1.5
1
0.5
0
1
2
3
4
5
6
7
8
Vein Order

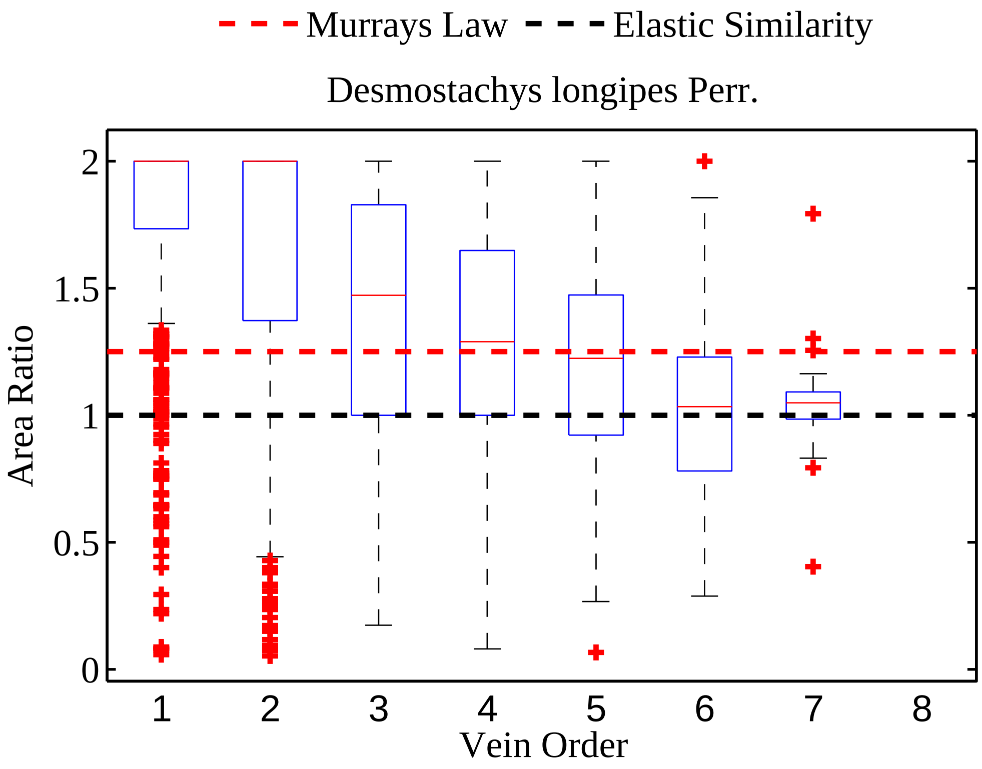

Murrays Law
Elastic Similarity
Desmostachys longipes Perr.
Area Ratio
0
0.5
1
1.5
2
1
2
3
4
5
6
7
8
Vein Order

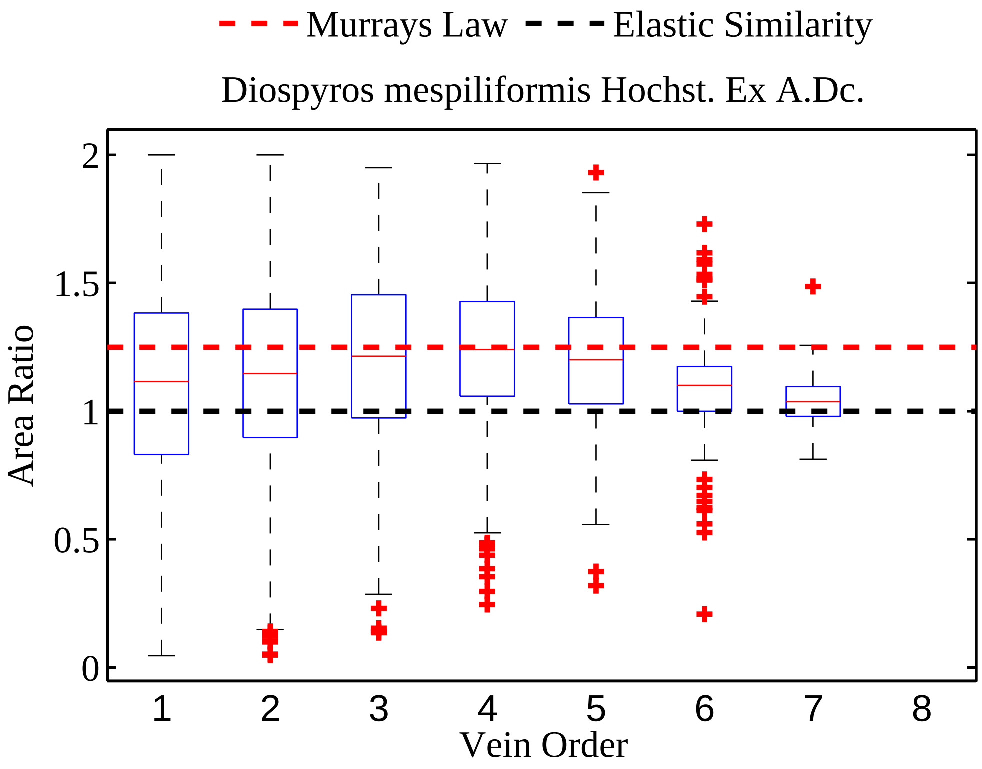

Murrays Law
Elastic Similarity
Diospyros mespiliformis Hochst. Ex A.Dc.
Area Ratio
0
0.5
1
1.5
2
1
2
3
4
5
6
7
8
Vein Order

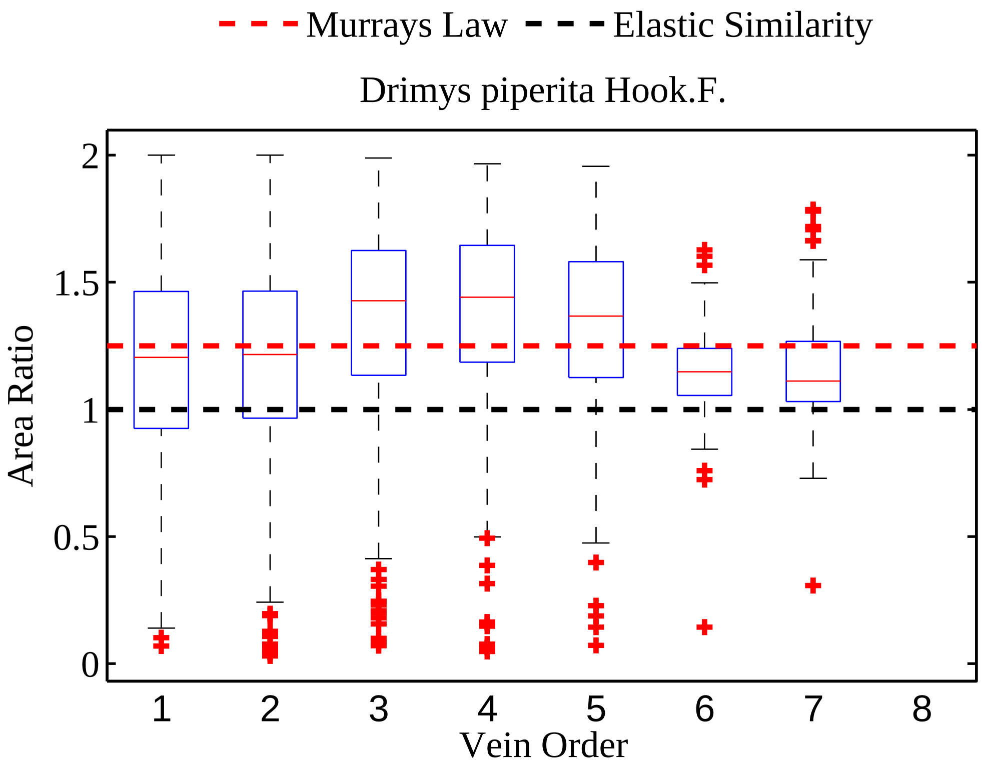

Murrays Law
Elastic Similarity
Drimys piperita Hook.F.
Area Ratio
0
0.5
1
1.5
2
1
2
3
4
5
6
7
8
Vein Order

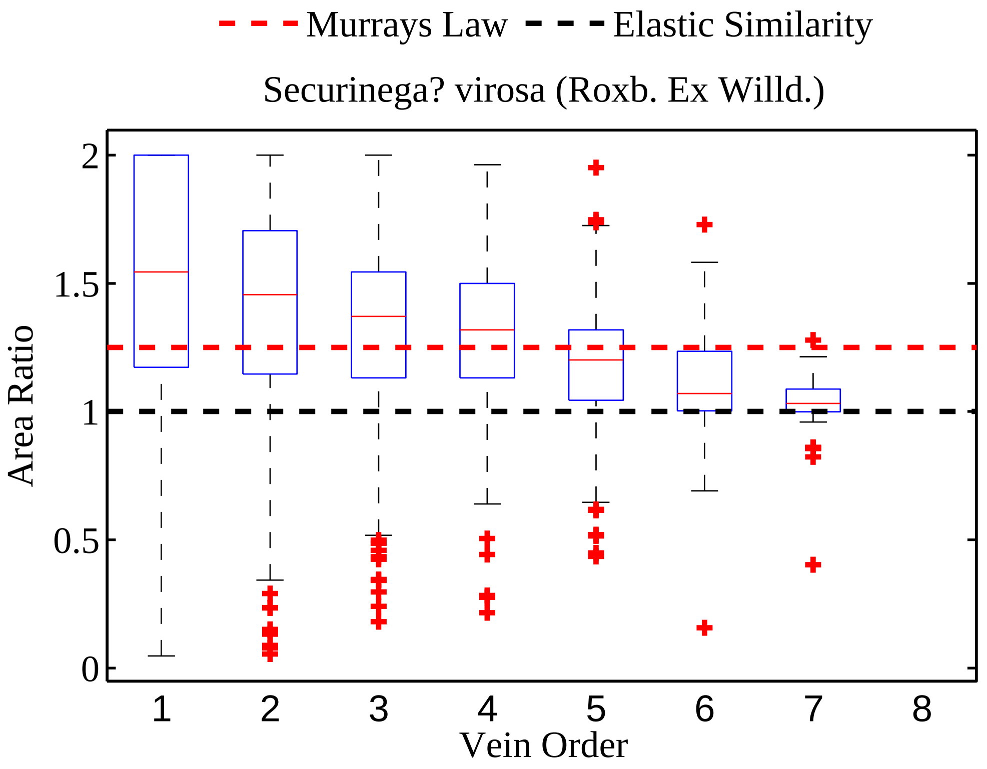

Murrays Law
Elastic Similarity
Securinega? virosa (Roxb. Ex Willd.)
Area Ratio
0
0.5
1
1.5
2
1
2
3
4
5
6
7
8
Vein Order

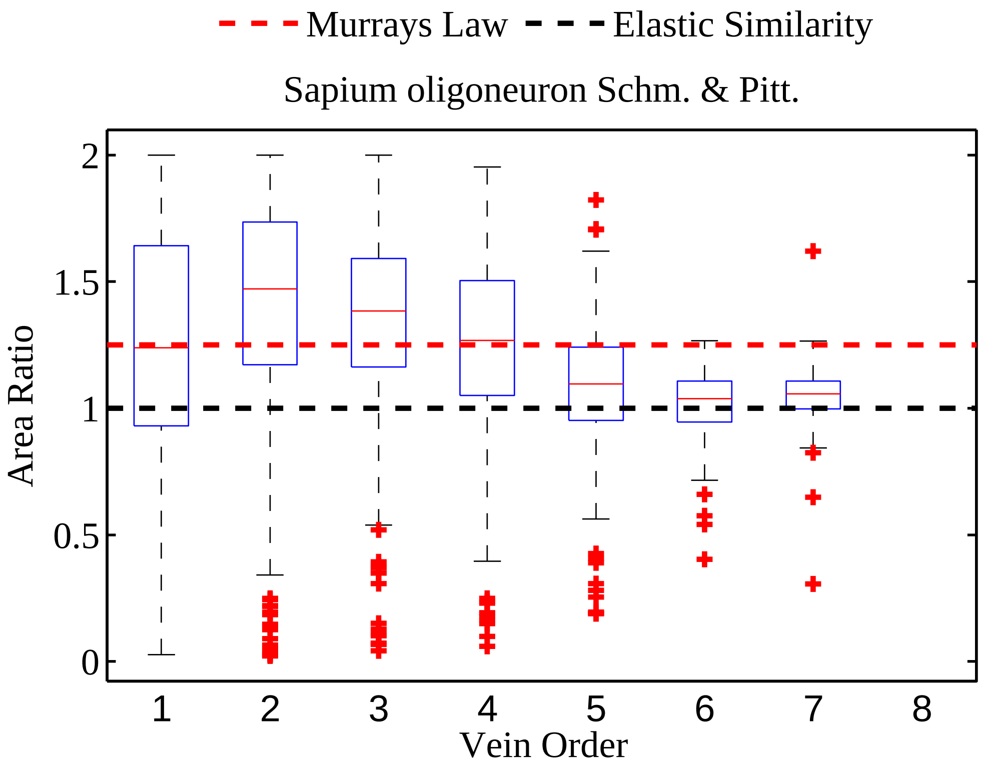

Murrays Law
Elastic Similarity
Sapium oligoneuron Schm. & Pitt.
Area Ratio
0
0.5
1
1.5
2
1
2
3
4
5
6
7
8
Vein Order

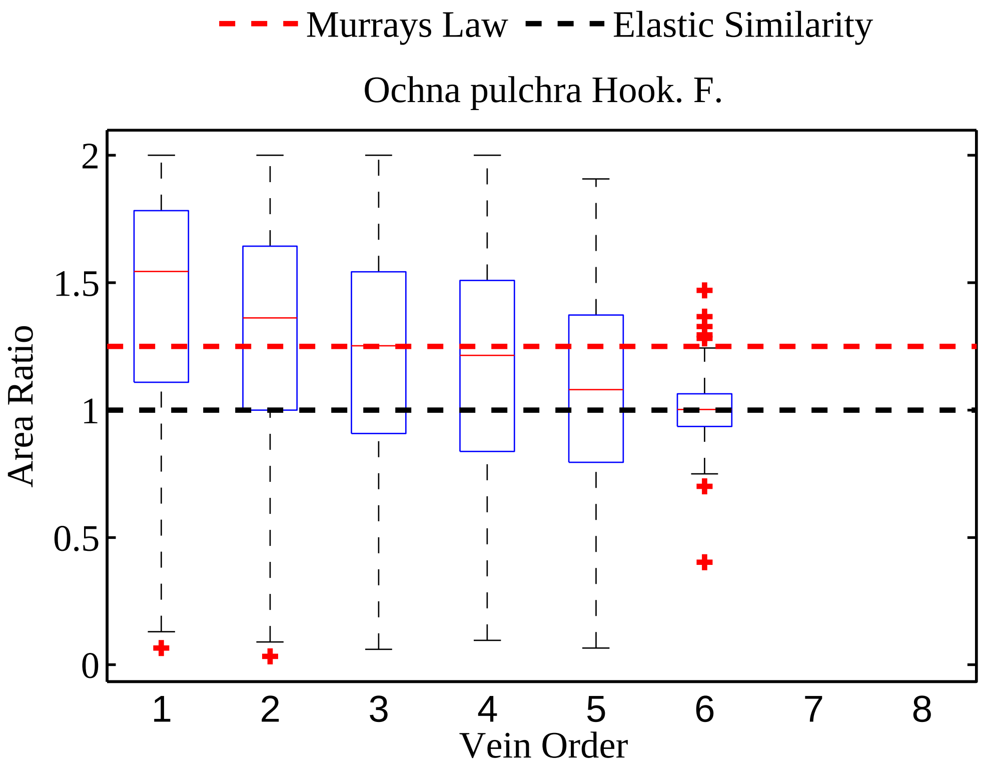
Murrays Law
Elastic Similarity
Ochna pulchra Hook. F.
Area Ratio
0
0.5
1
1.5
2
1
2
3
4
5
6
7
8
Vein Order

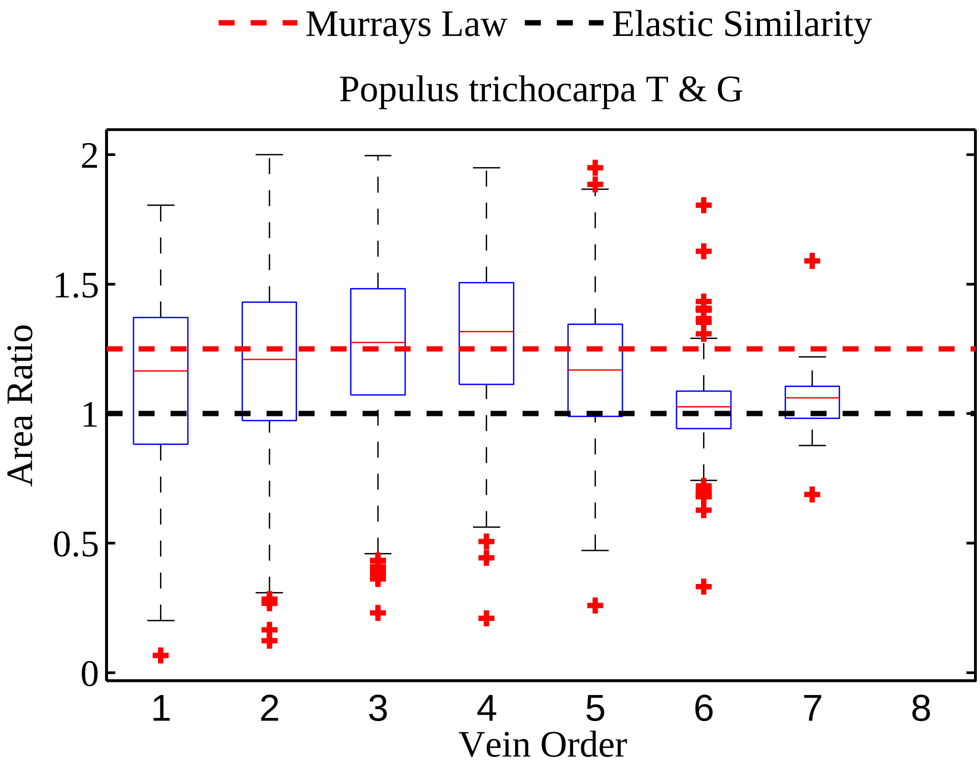

Murrays Law
Elastic Similarity
Populus trichocarpa T & G
Area Ratio
0
0.5
1
1.5
2
1
2
3
4
5
6
7
8
Vein Order

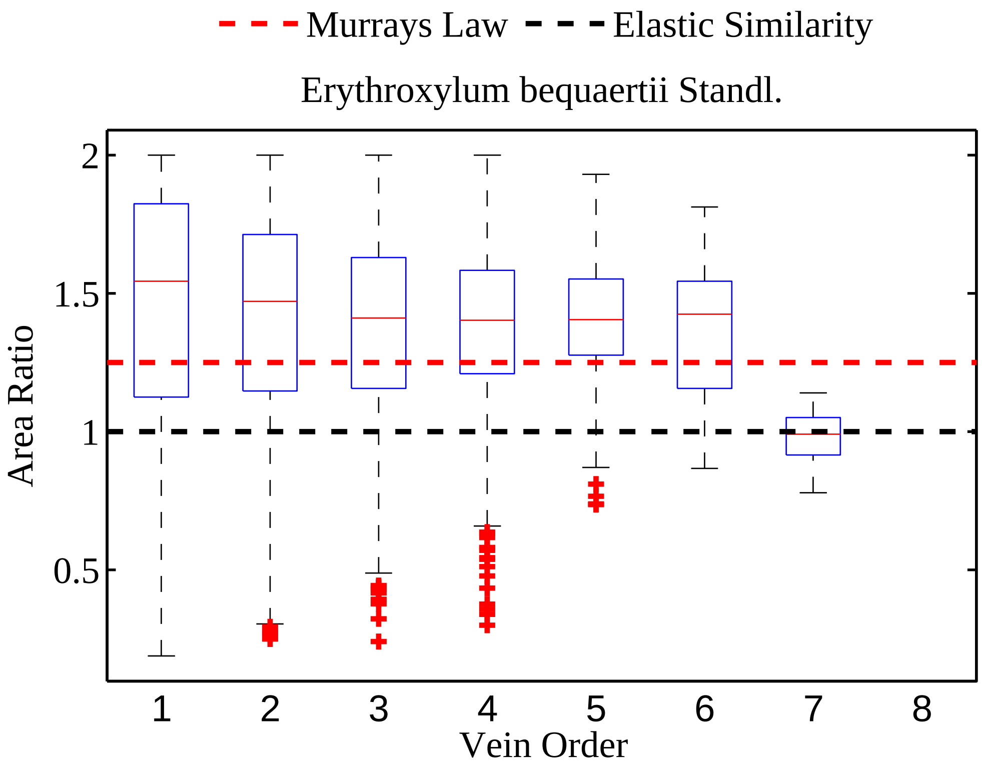

Murrays Law
Elastic Similarity
Erythroxylum bequaertii Standl.
Area Ratio
2
1.5
1
0.5
1
2
3
4
5
6
7
8
Vein Order

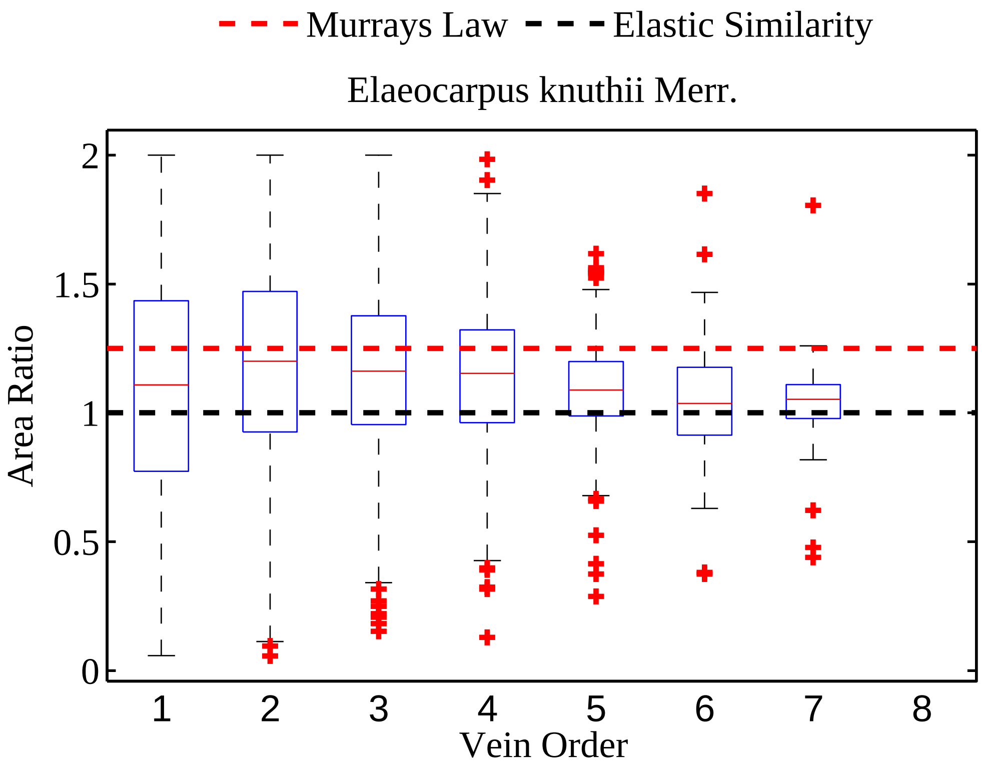
Murrays Law
Elastic Similarity
Elaeocarpus knuthii Merr.
Area Ratio
Vein Order
0
0.5
1
1.5
2
1
2
3
4
5
6
7
8

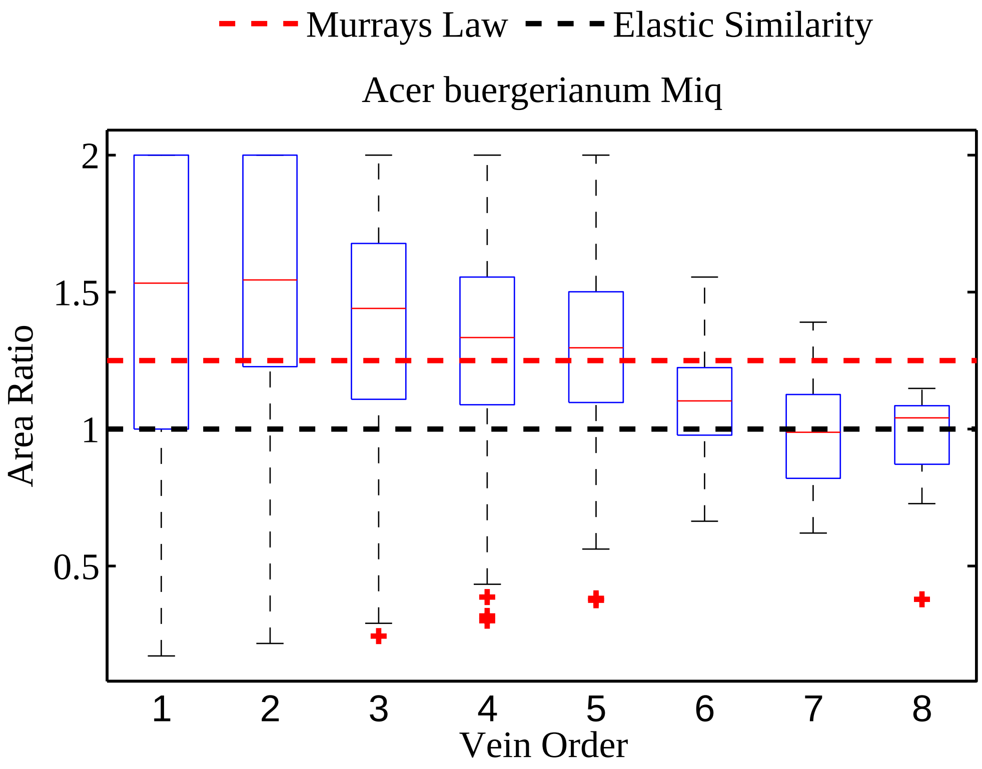

Murrays Law
Elastic Similarity
Acer buergerianum Miq
Area Ratio
2
1.5
1
0.5
1
2
3
4
5
6
7
8
Vein Order

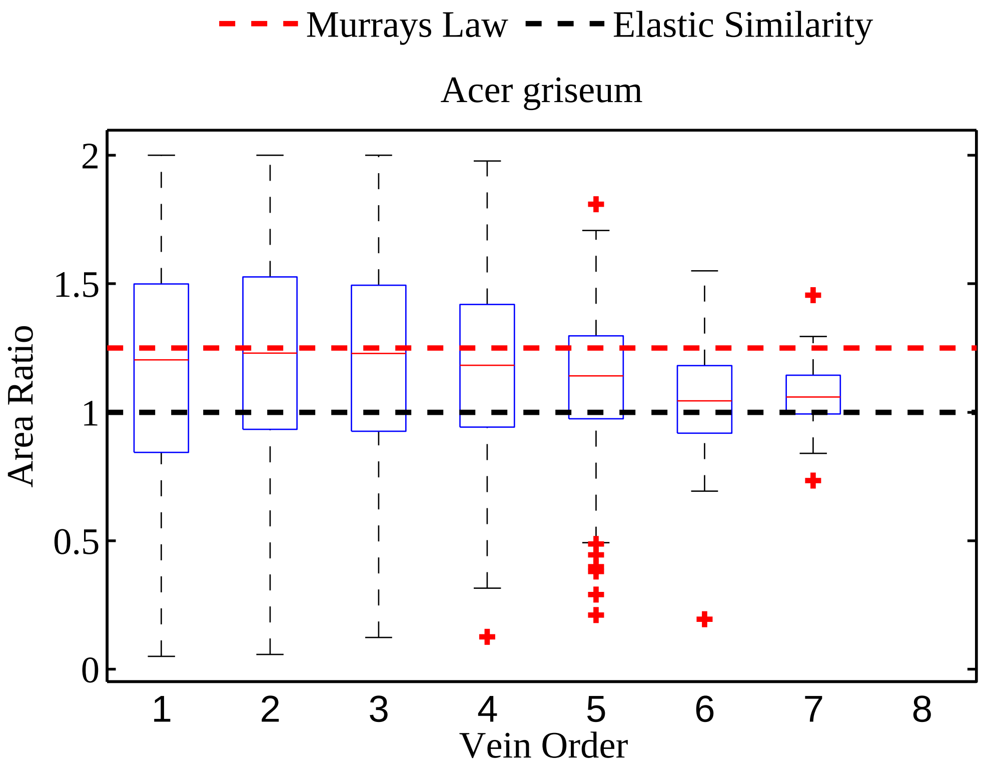

Murrays Law
Elastic Similarity
Acer griseum
Area Ratio
0
0.5
1
1.5
2
1
2
3
4
5
6
7
8
Vein Order

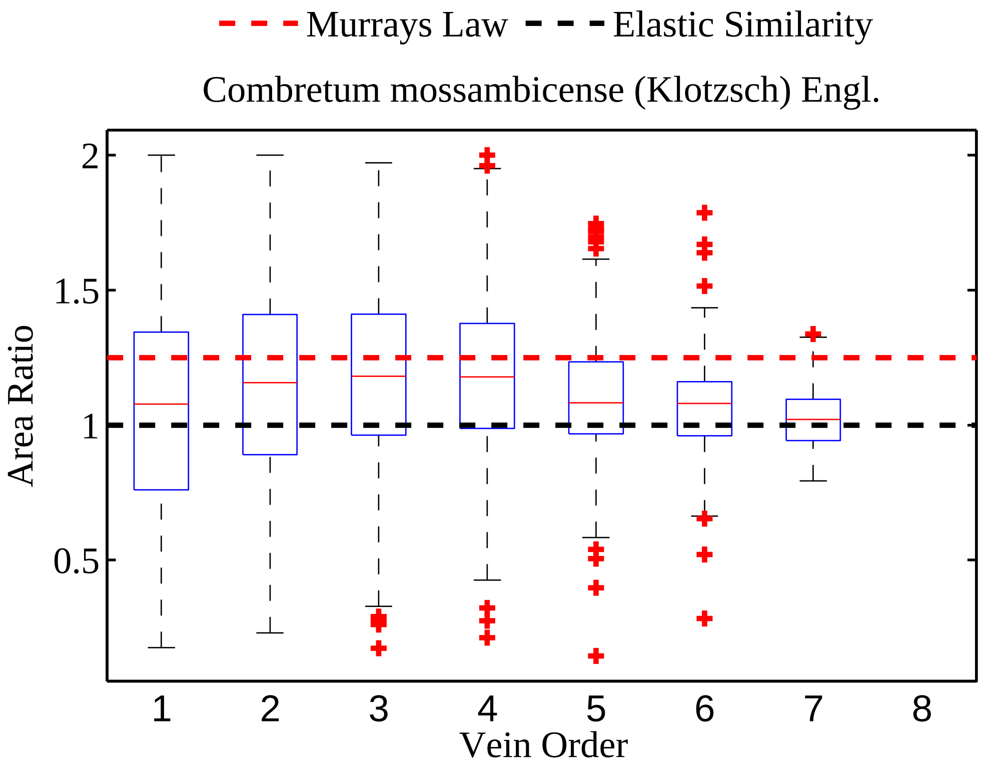
Murrays Law
Elastic Similarity
Combretum mossambicense (Klotzsch) Engl.
Area Ratio
2
1.5
1
0.5
1
2
3
4
5
6
7
8
Vein Order

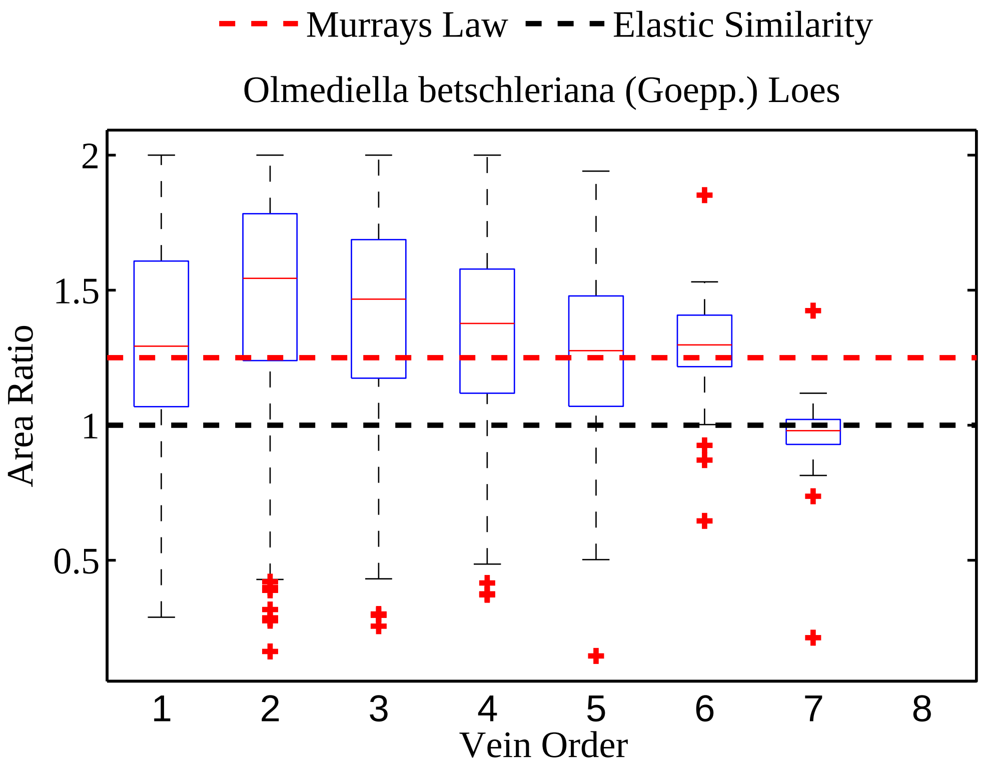
Murrays Law
Elastic Similarity
Olmediella betschleriana (Goepp.) Loes
Area Ratio
Vein Order
2
1.5
1
0.5
1
2
3
4
5
6
7
8

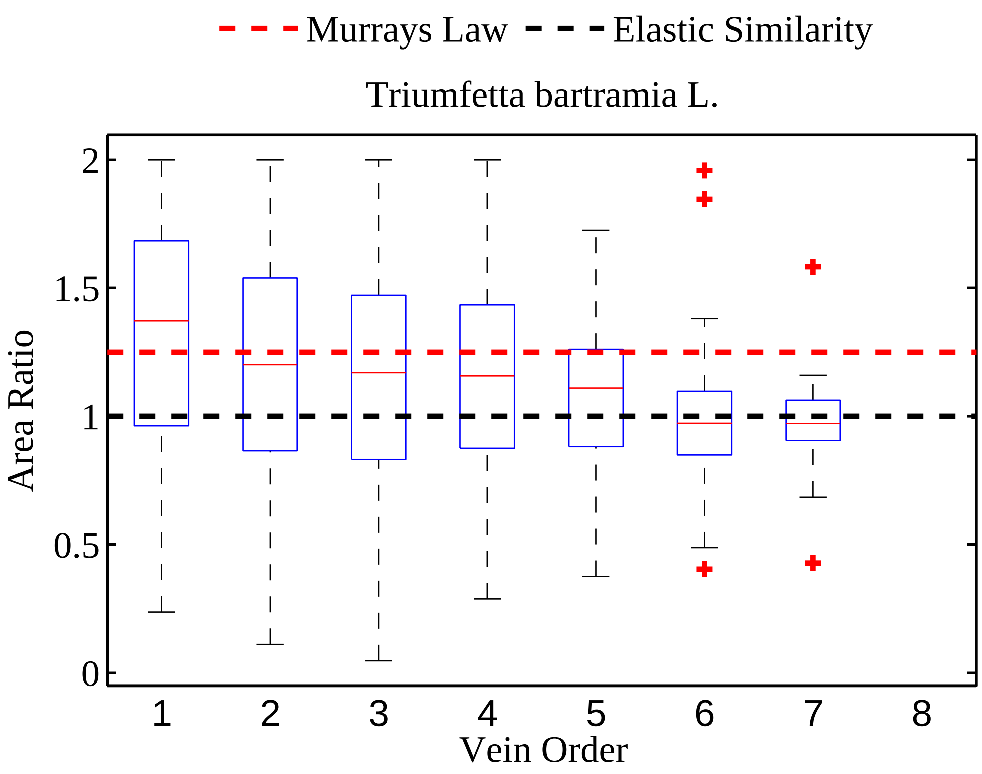

Murrays Law
Elastic Similarity
Triumfetta bartramia L.
Area Ratio
Vein Order
0
0.5
1
1.5
2
1
2
3
4
5
6
7
8

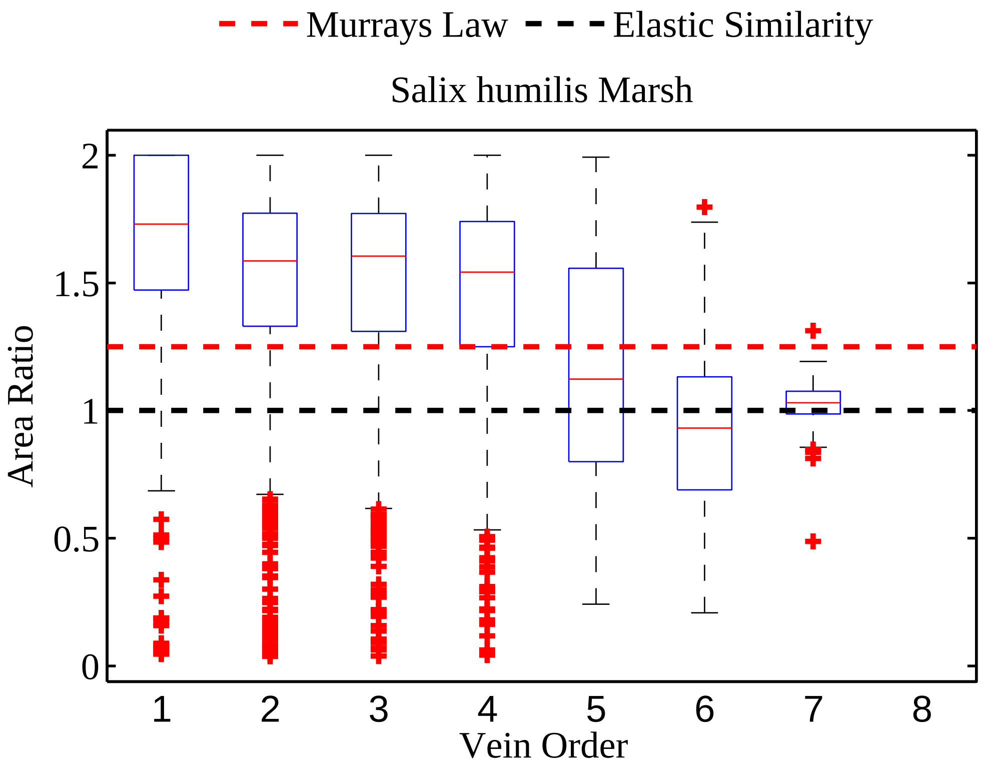

Murrays Law
Elastic Similarity
Salix humilis Marsh
Area Ratio
0
0.5
1
1.5
2
1
2
3
4
5
6
7
8
Vein Order

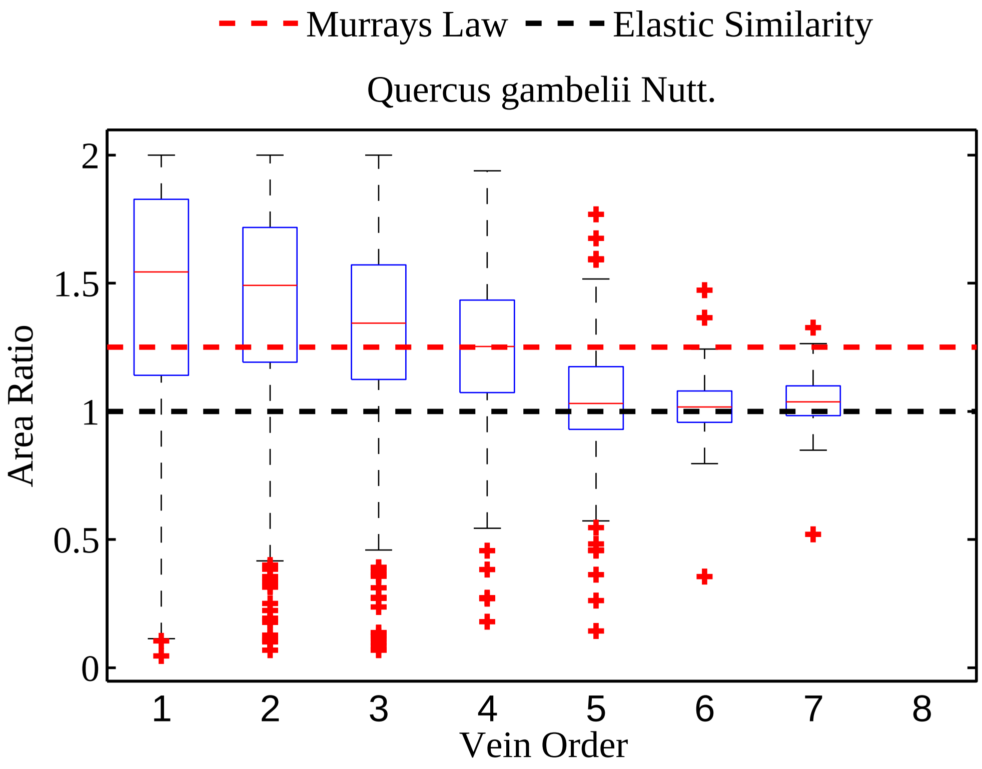

Murrays Law
Elastic Similarity
Quercus gambelii Nutt.
Area Ratio
0
0.5
1
1.5
2
1
2
3
4
5
6
7
8
Vein Order

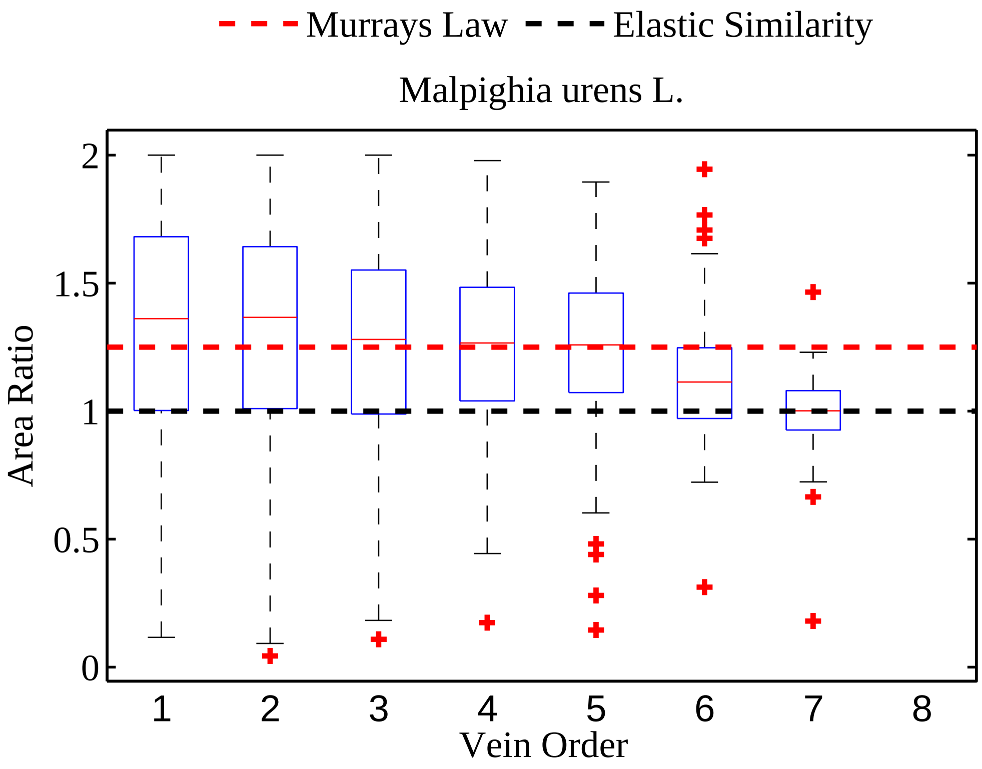

Murrays Law
Elastic Similarity
Malpighia urens L.
Area Ratio
2
1.5
1
0.5
0
1
2
3
4
5
6
7
8
Vein Order

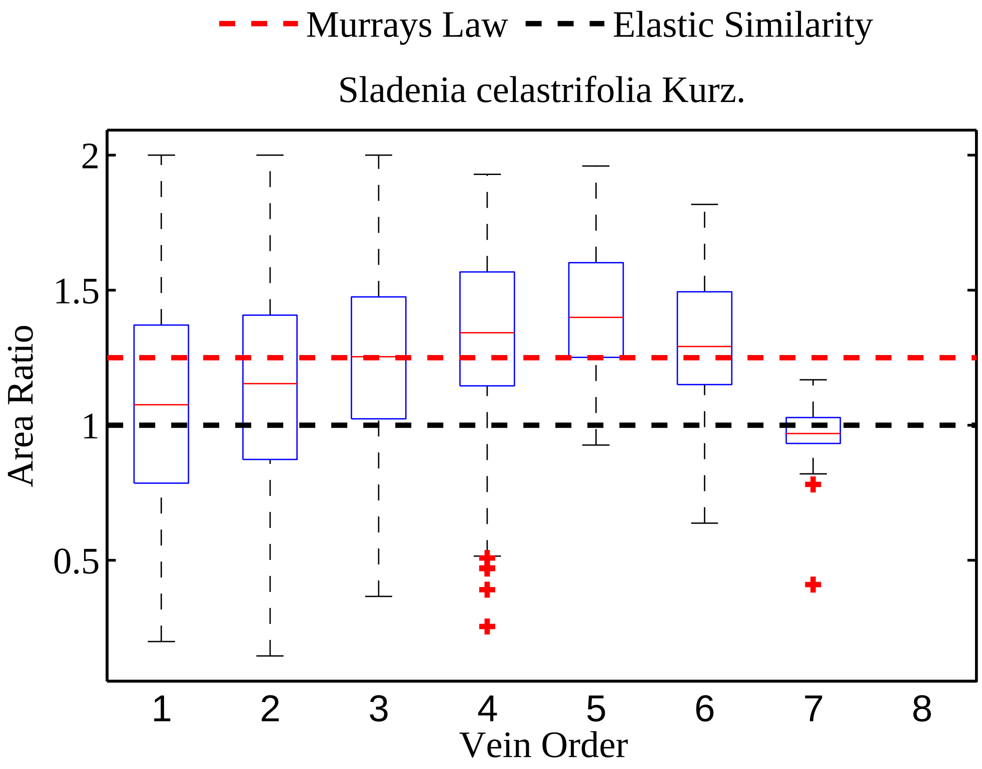

Murrays Law
Elastic Similarity
Sladenia celastrifolia Kurz.
Area Ratio
2
1.5
1
0.5
1
2
3
4
5
6
7
8
Vein Order

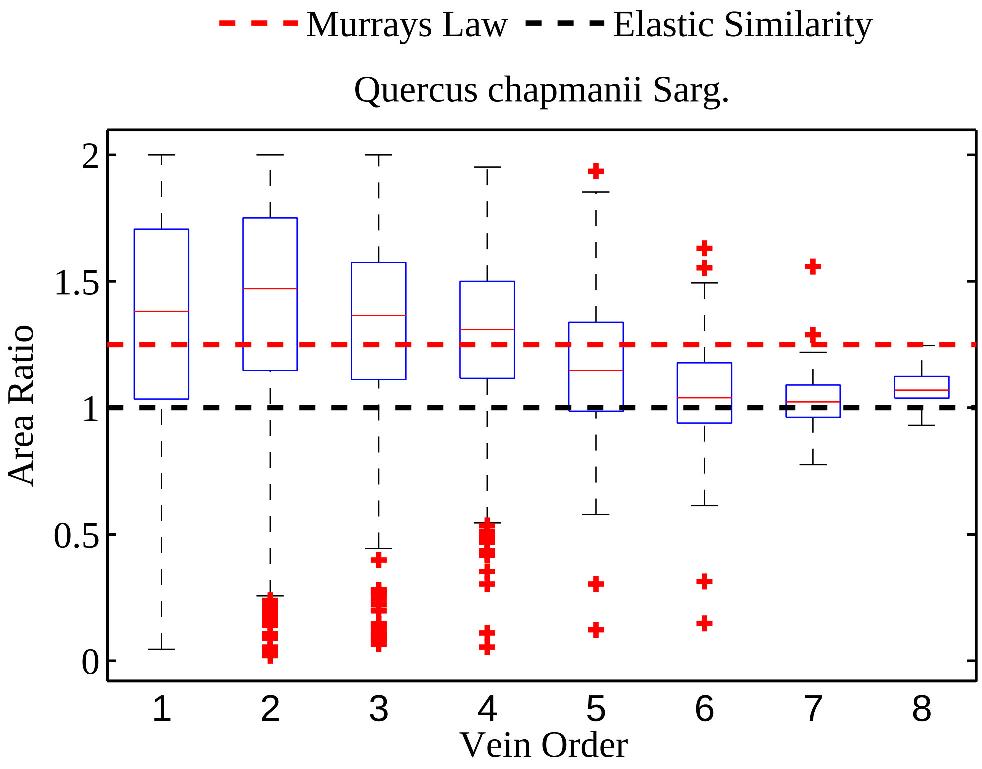

Murrays Law
Elastic Similarity
Quercus chapmanii Sarg.
Area Ratio
Vein Order
0
0.5
1
1.5
2
1
2
3
4
5
6
7
8

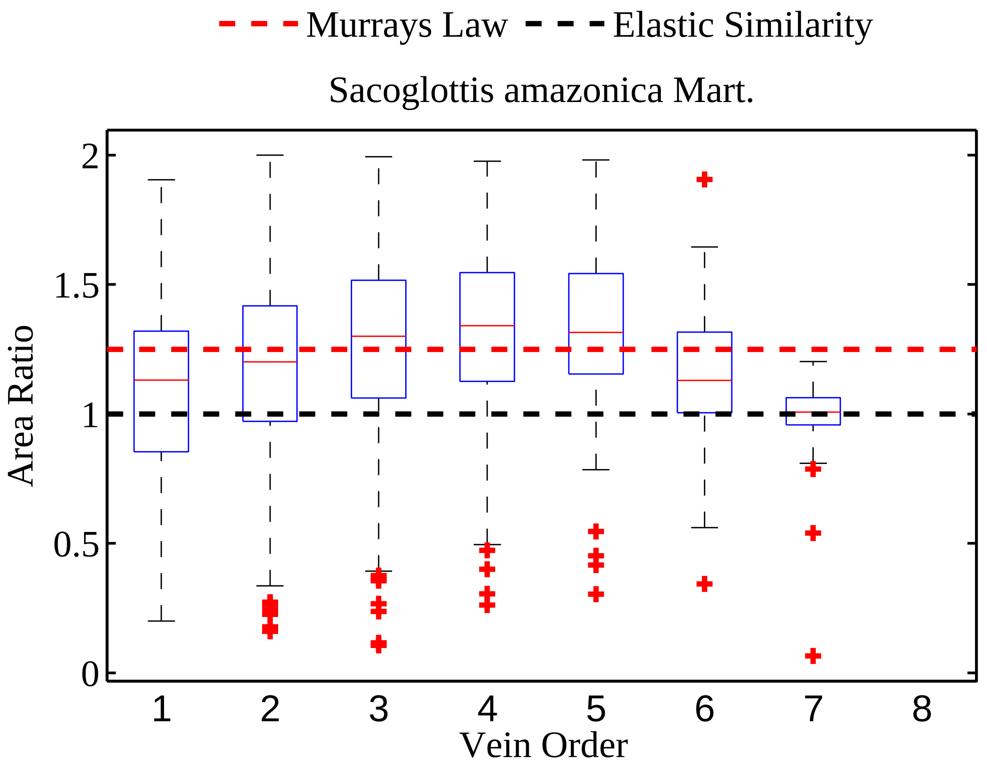

Murrays Law
Elastic Similarity
Sacoglottis amazonica Mart.
Area Ratio
0
0.5
1
1.5
2
1
2
3
4
5
6
7
8
Vein Order

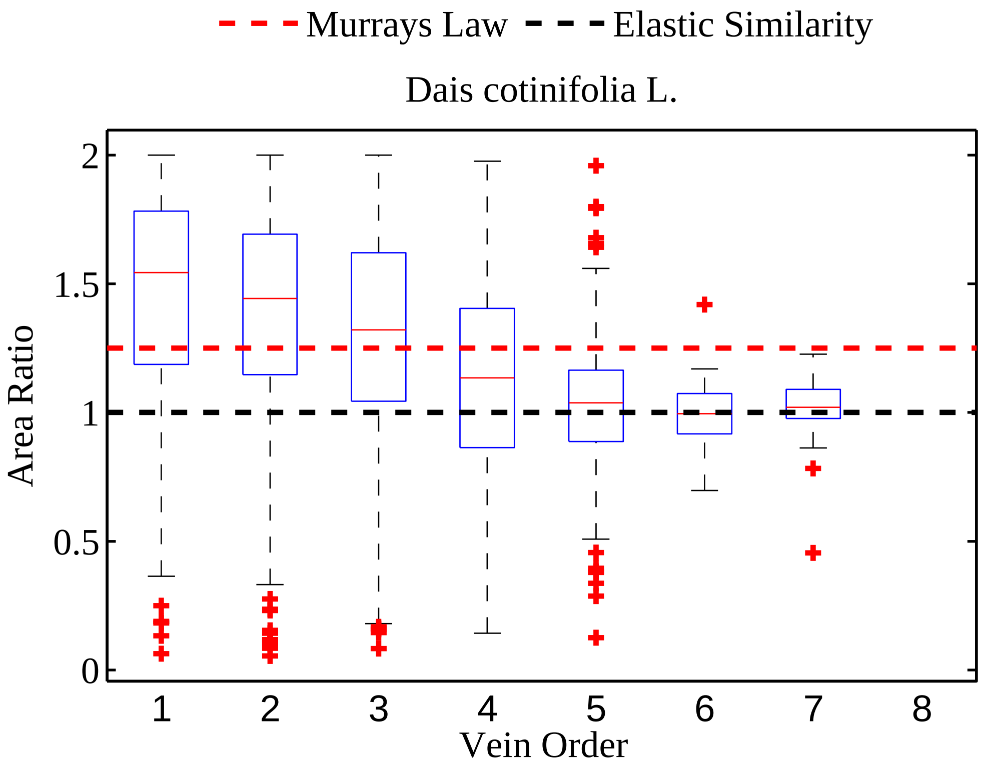
Murrays Law
Elastic Similarity
Dais cotinifolia L.
Area Ratio
0
0.5
1
1.5
2
1
2
3
4
5
6
7
8
Vein Order

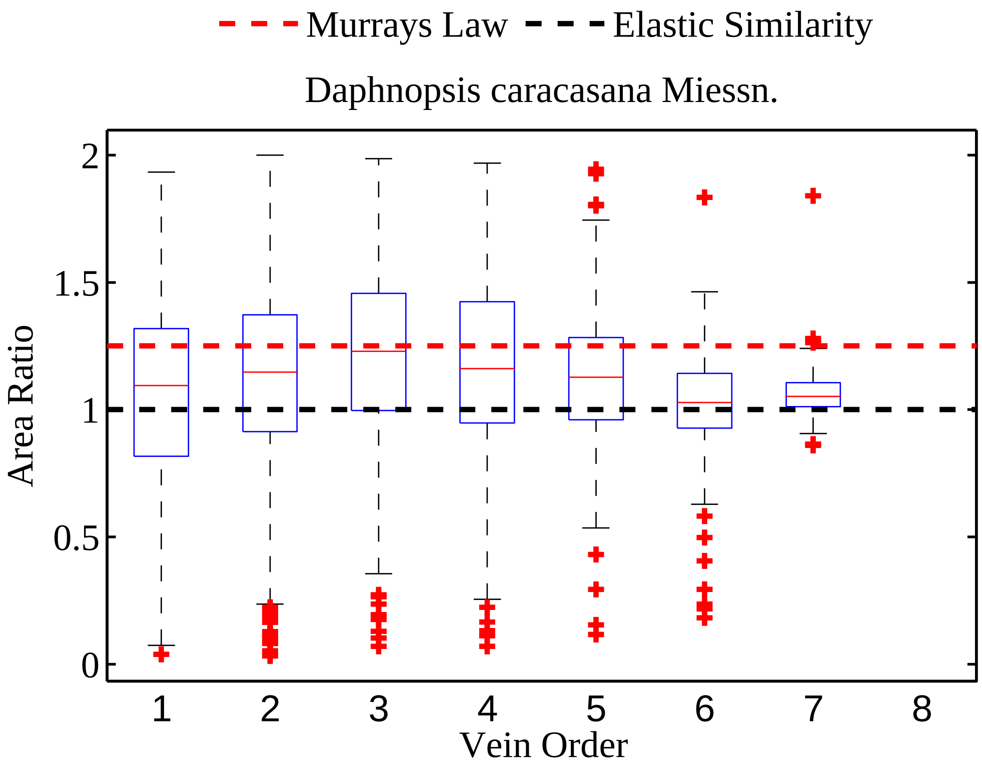

Murrays Law
Elastic Similarity
Daphnopsis caracasana Miessn.
Area Ratio
Vein Order
0
0.5
1
1.5
2
1
2
3
4
5
6
7
8

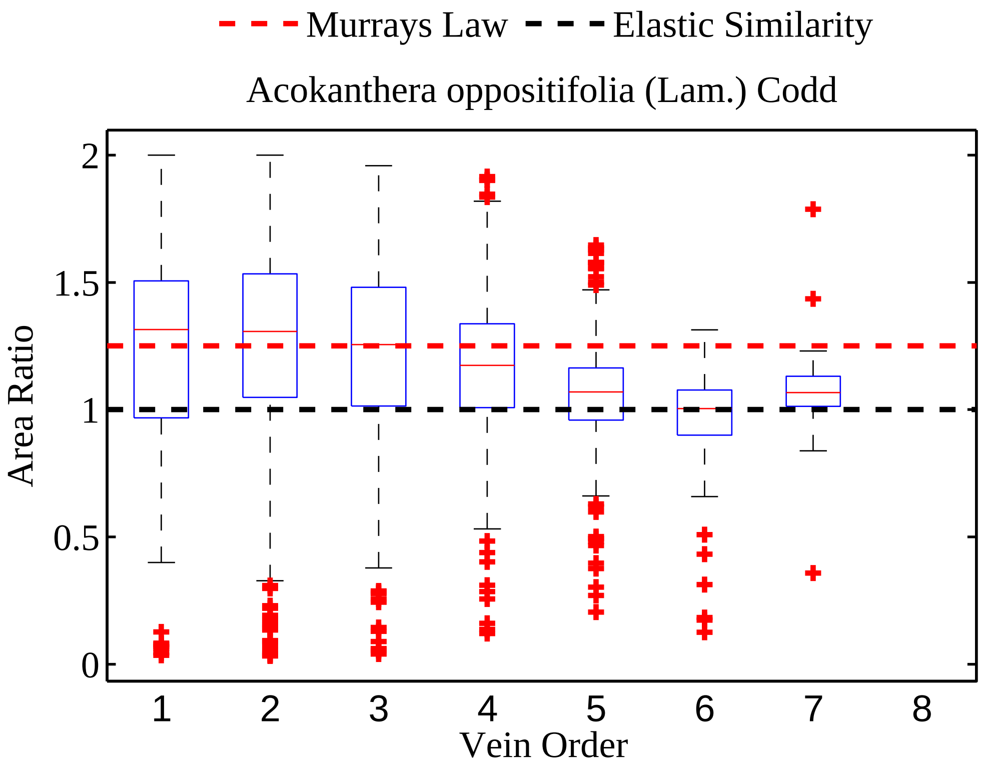

Murrays Law
Elastic Similarity
Acokanthera oppositifolia (Lam.) Codd
Area Ratio
0
0.5
1
1.5
2
1
2
3
4
5
6
7
8
Vein Order

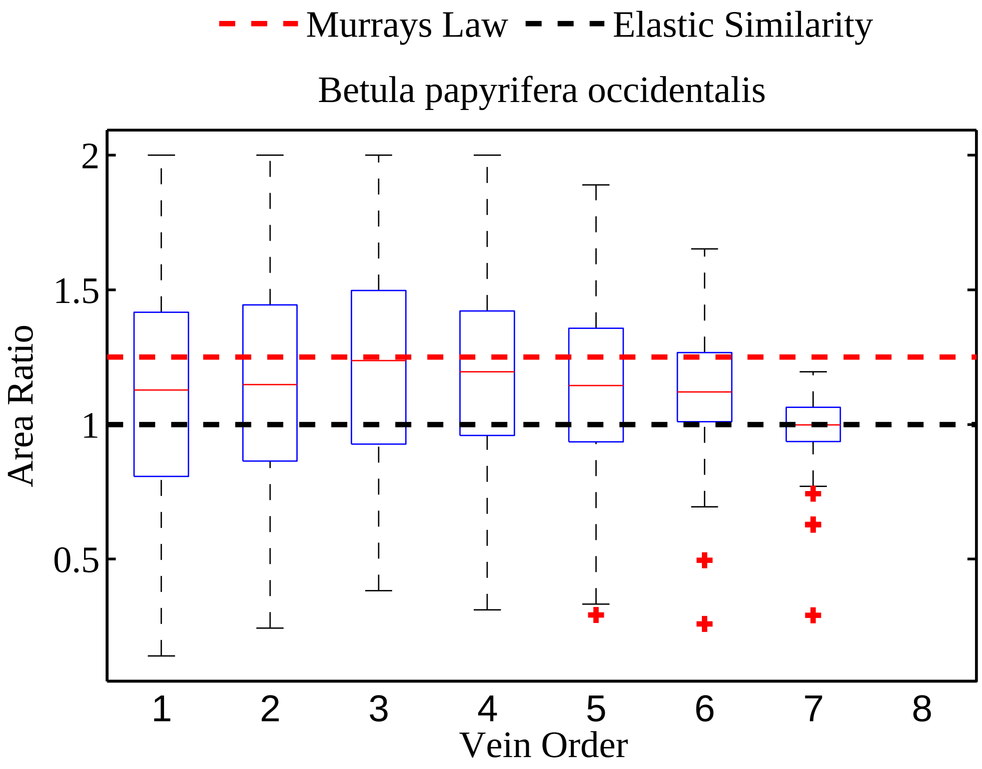

Murrays Law
Elastic Similarity
Betula papyrifera occidentalis
Area Ratio
2
1.5
1
0.5
1
2
3
4
5
6
7
8
Vein Order

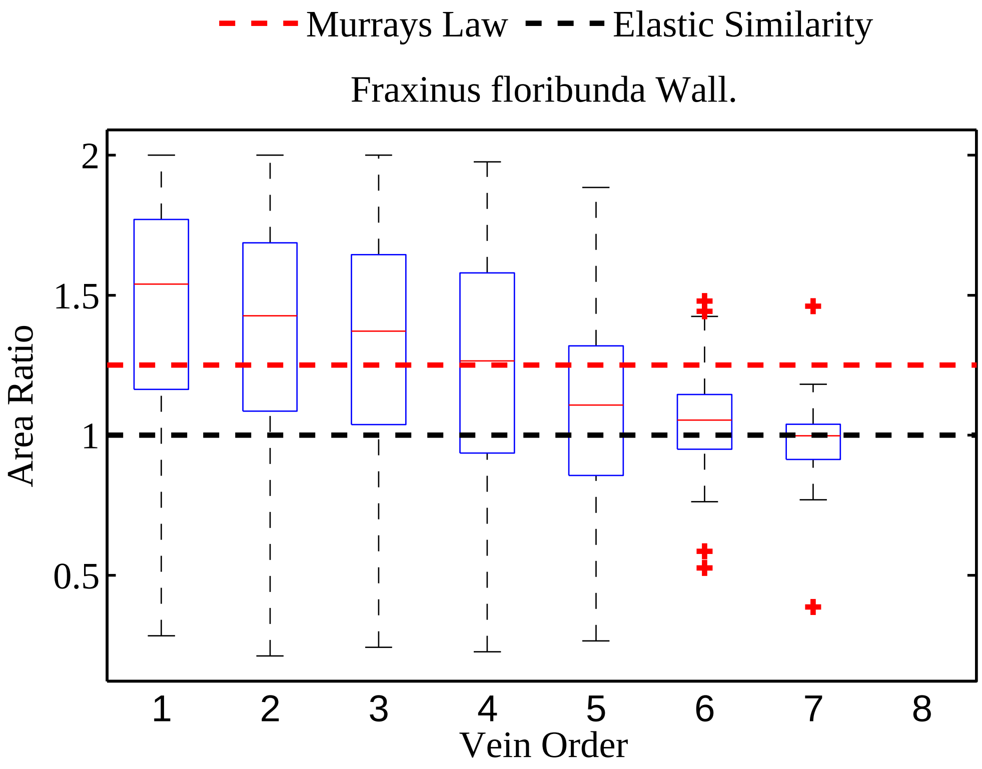
Murrays Law
Elastic Similarity
Fraxinus floribunda Wall.
Area Ratio
Vein Order
2
1.5
1
0.5
1
2
3
4
5
6
7
8

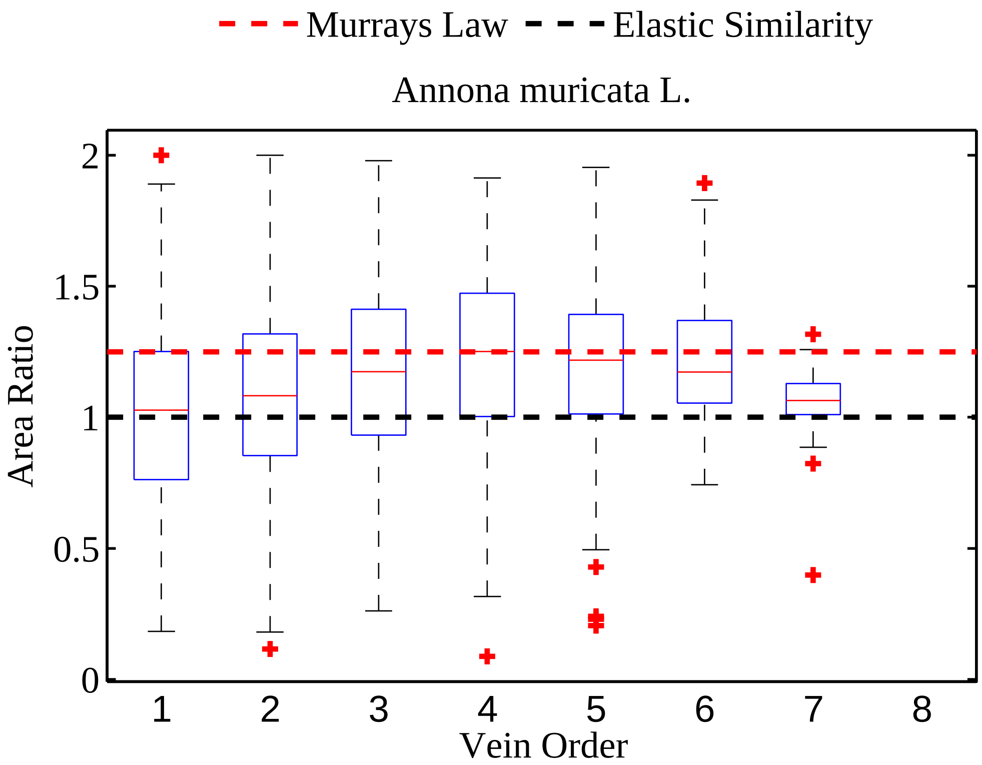

Murrays Law
Elastic Similarity
Annona muricata L.
Area Ratio
0
0.5
1
1.5
2
1
2
3
4
5
6
7
8
Vein Order

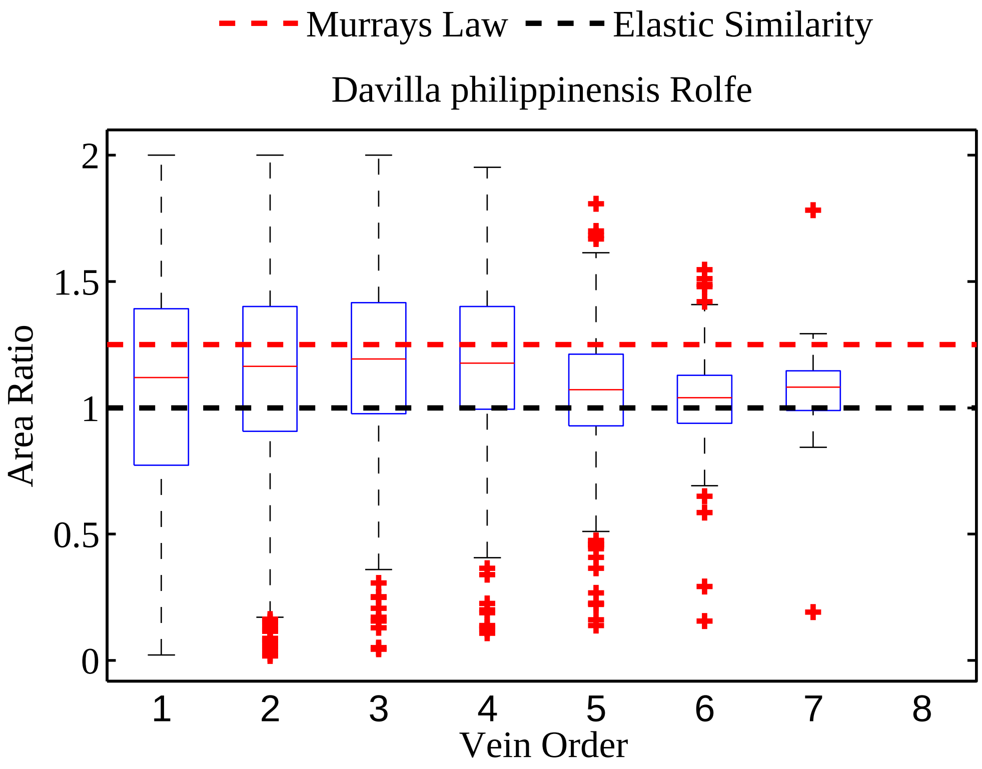

Murrays Law
Elastic Similarity
Davilla philippinensis Rolfe
Area Ratio
2
1.5
1
0.5
0
1
2
3
4
5
6
7
8
Vein Order

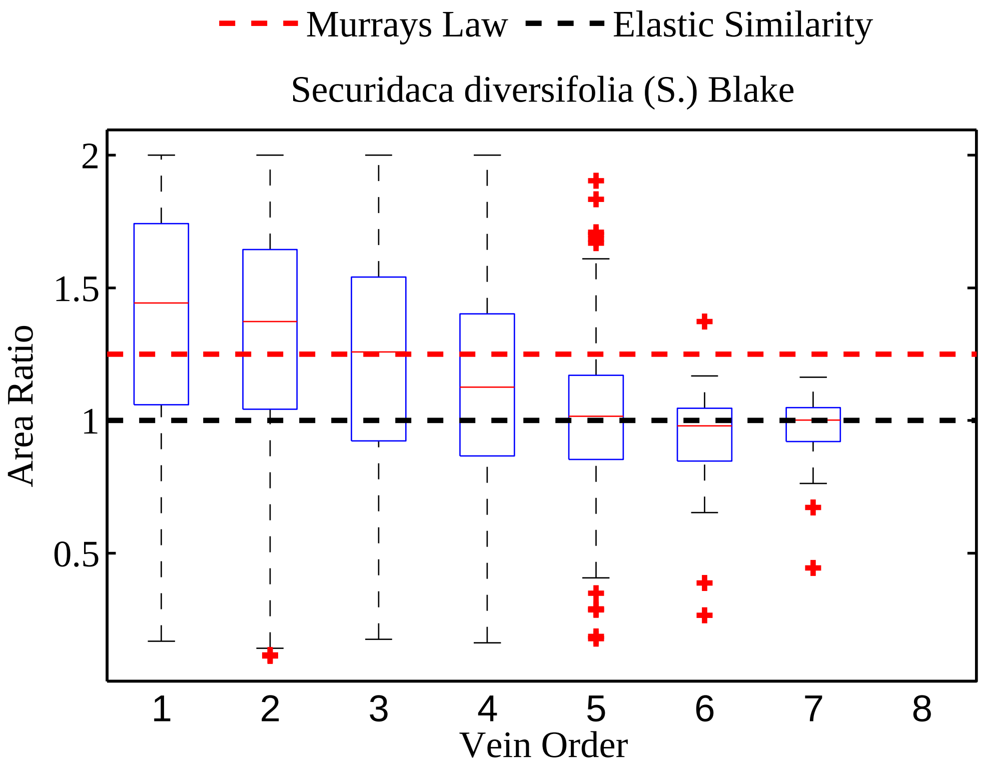

Murrays Law
Elastic Similarity
Securidaca diversifolia (S.) Blake
Area Ratio
Vein Order
2
1.5
1
0.5
1
2
3
4
5
6
7
8

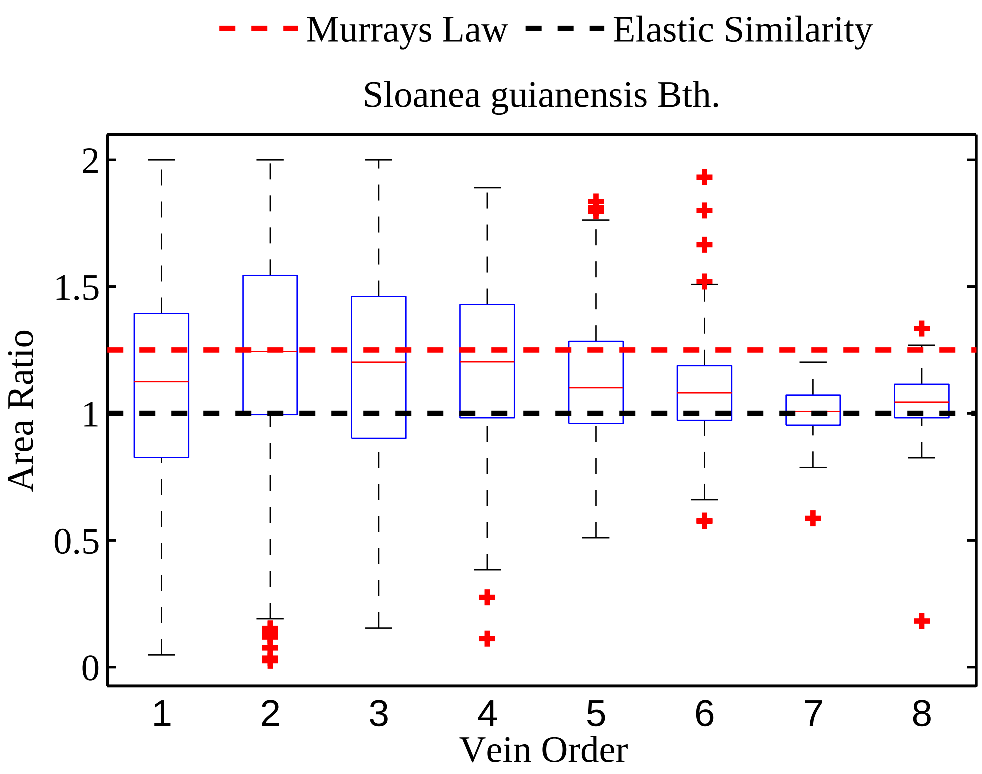

Murrays Law
Elastic Similarity
Sloanea guianensis Bth.
Area Ratio
0
0.5
1
1.5
2
1
2
3
4
5
6
7
8
Vein Order

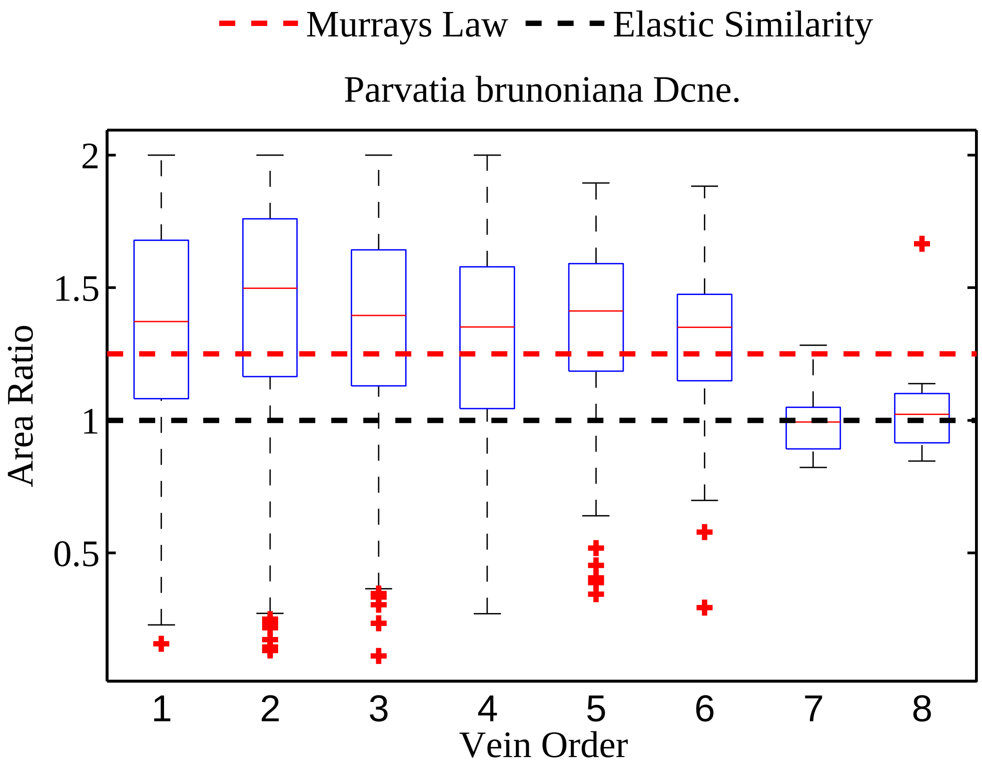

Murrays Law
Elastic Similarity
Parvatia brunoniana Dcne.
Area Ratio
2
1.5
1
0.5
1
2
3
4
5
6
7
8
Vein Order

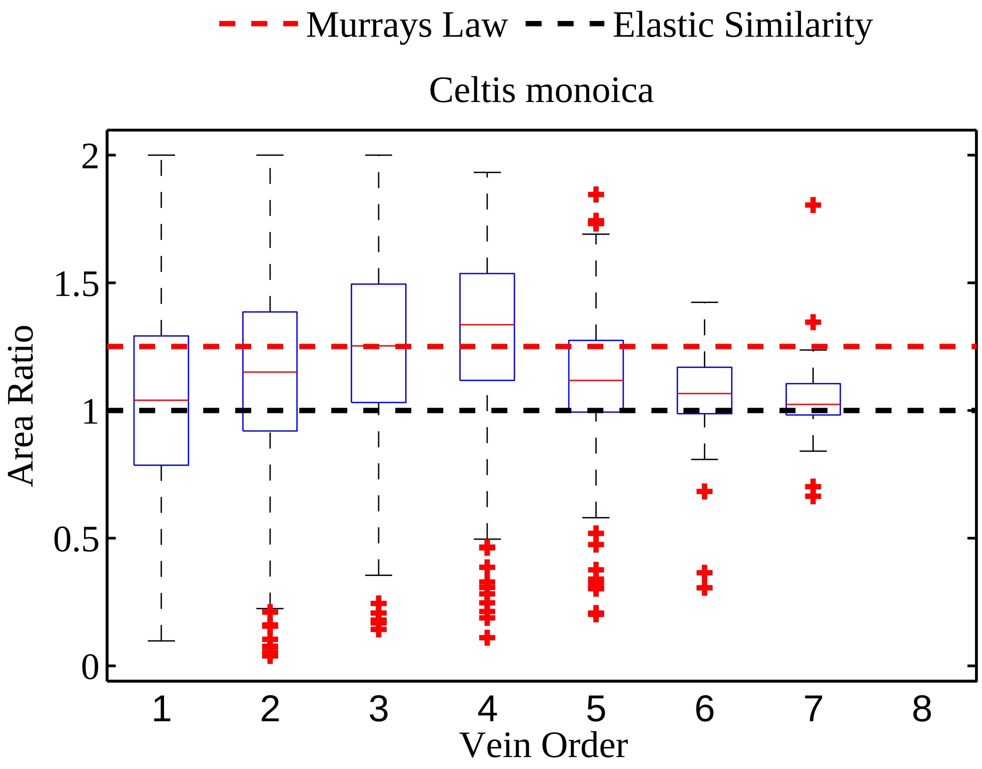

Murrays Law
Elastic Similarity
Celtis monoica
Area Ratio
0
0.5
1
1.5
2
1
2
3
4
5
6
7
8
Vein Order

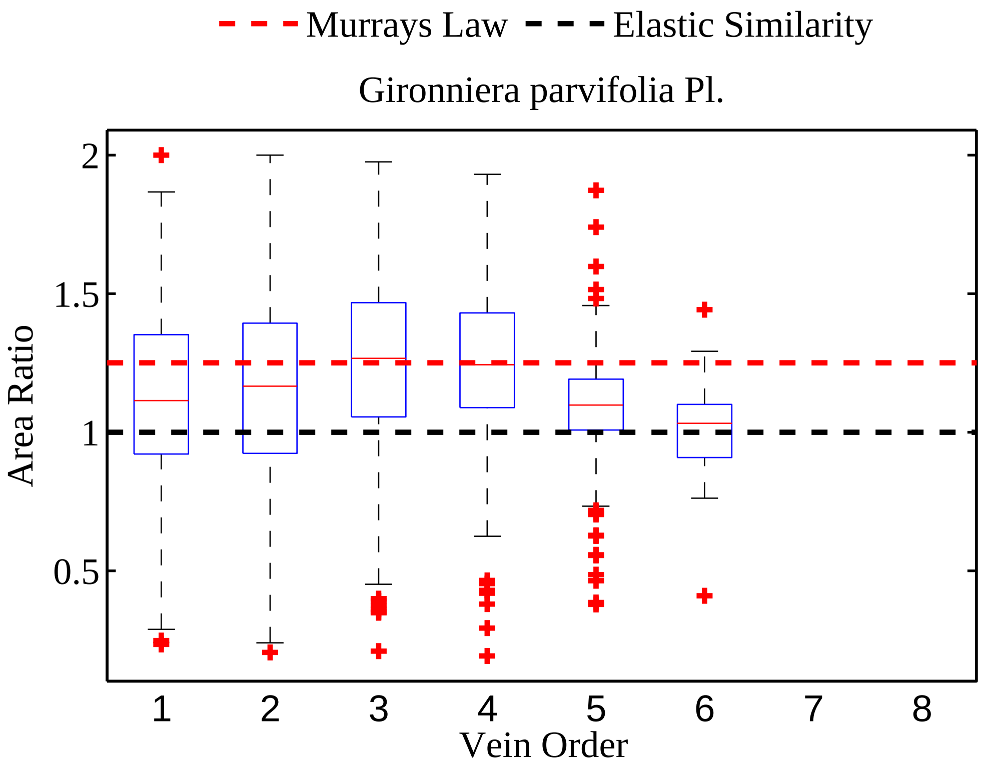
Murrays Law
Elastic Similarity
Gironniera parvifolia Pl.
Area Ratio
2
1.5
1
0.5
1
2
3
4
5
6
7
8
Vein Order

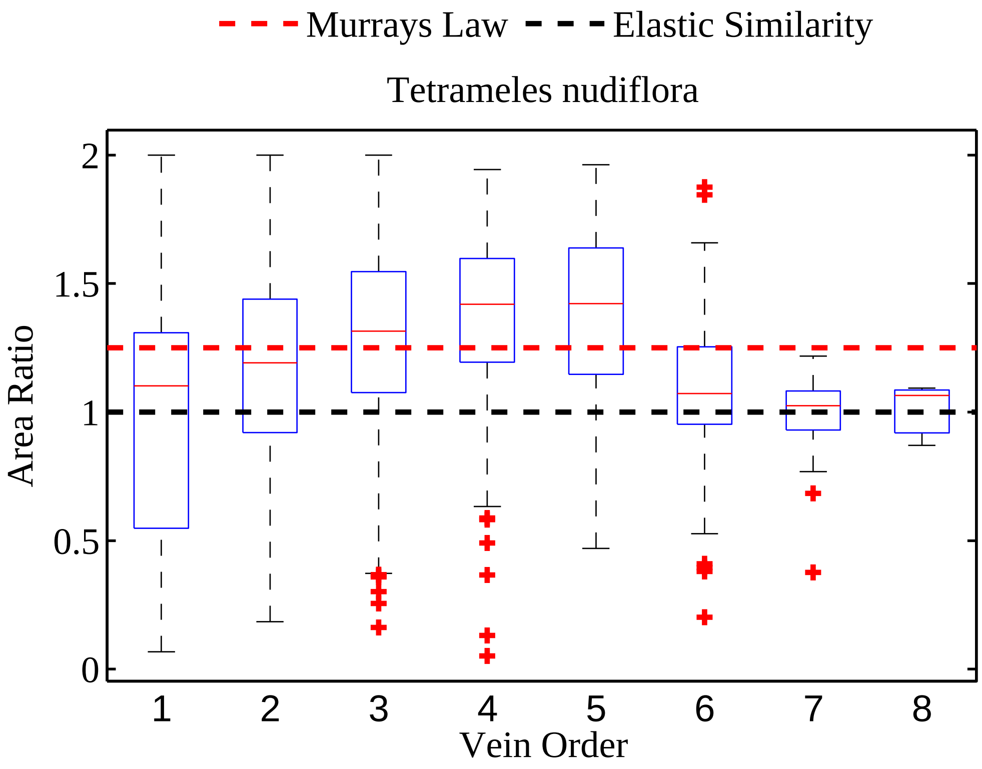
Murrays Law
Elastic Similarity
Tetrameles nudiflora
Area Ratio
Vein Order
0
0.5
1
1.5
2
1
2
3
4
5
6
7
8

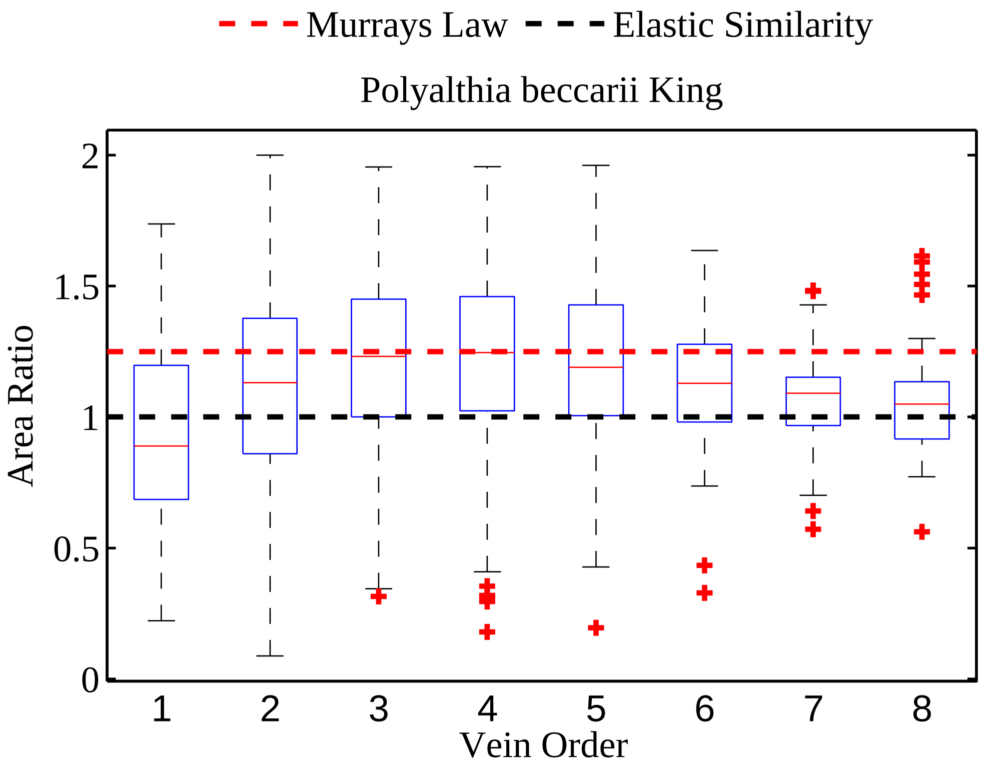

Murrays Law
Elastic Similarity
Polyalthia beccarii King
Area Ratio
Vein Order
0
0.5
1
1.5
2
1
2
3
4
5
6
7
8

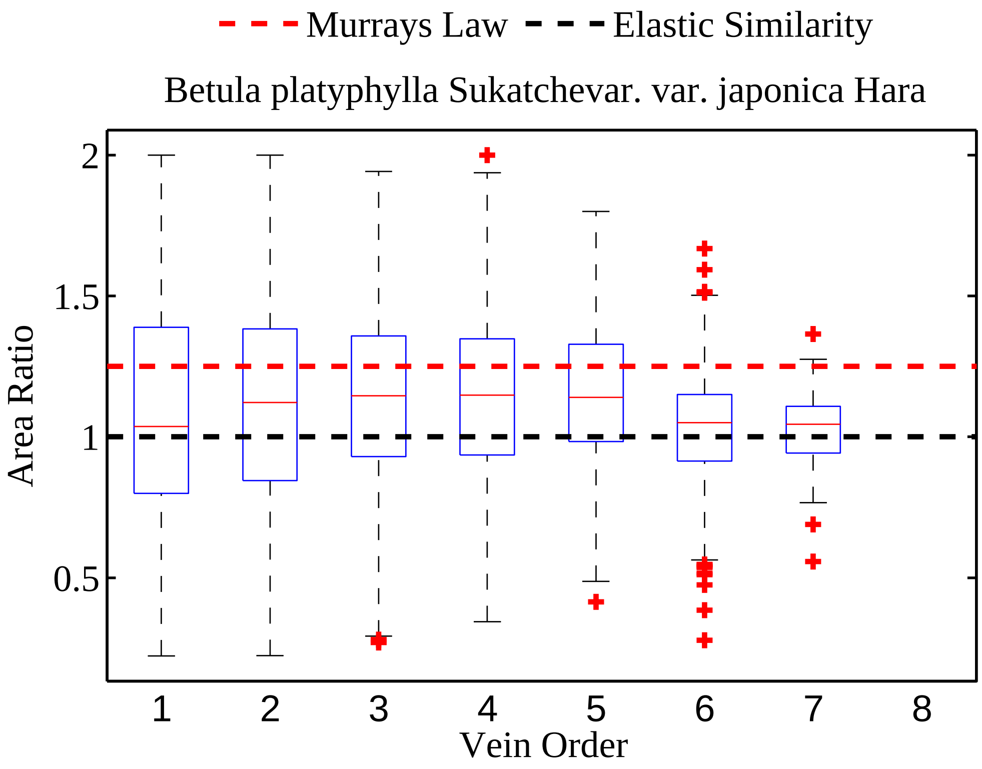
Murrays Law
Elastic Similarity
Betula platyphylla Sukatchevar. var. japonica Hara
Area Ratio
2
1.5
1
0.5
1
2
3
4
5
6
7
8
Vein Order

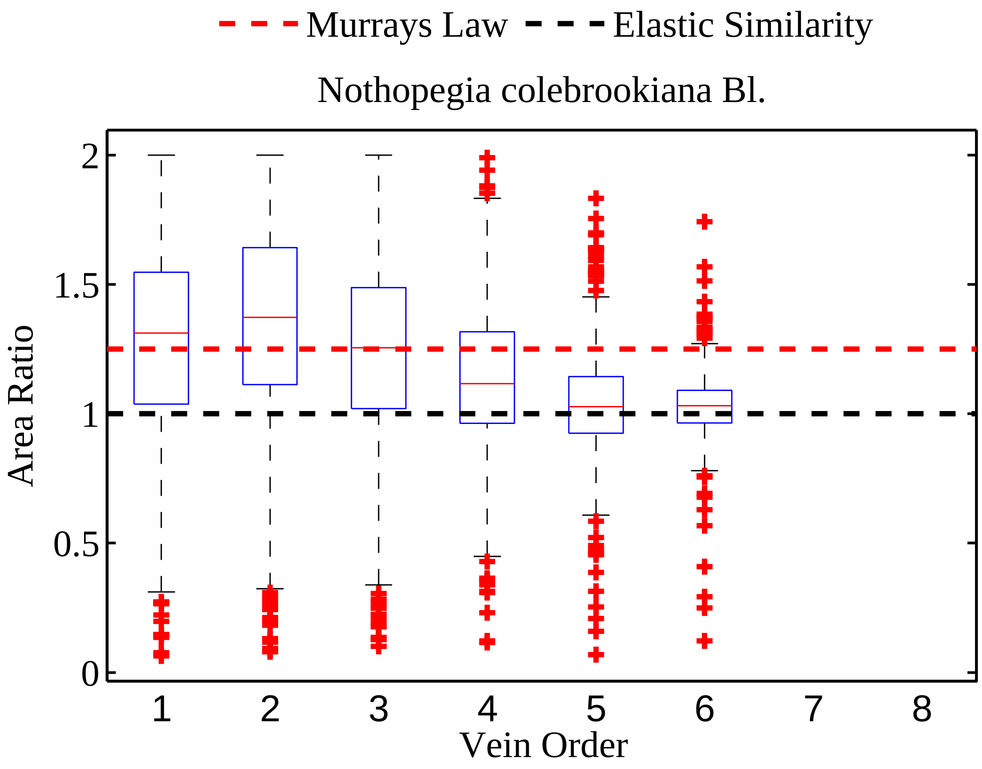

Murrays Law
Elastic Similarity
Nothopegia colebrookiana Bl.
Area Ratio
0
0.5
1
1.5
2
1
2
3
4
5
6
7
8
Vein Order

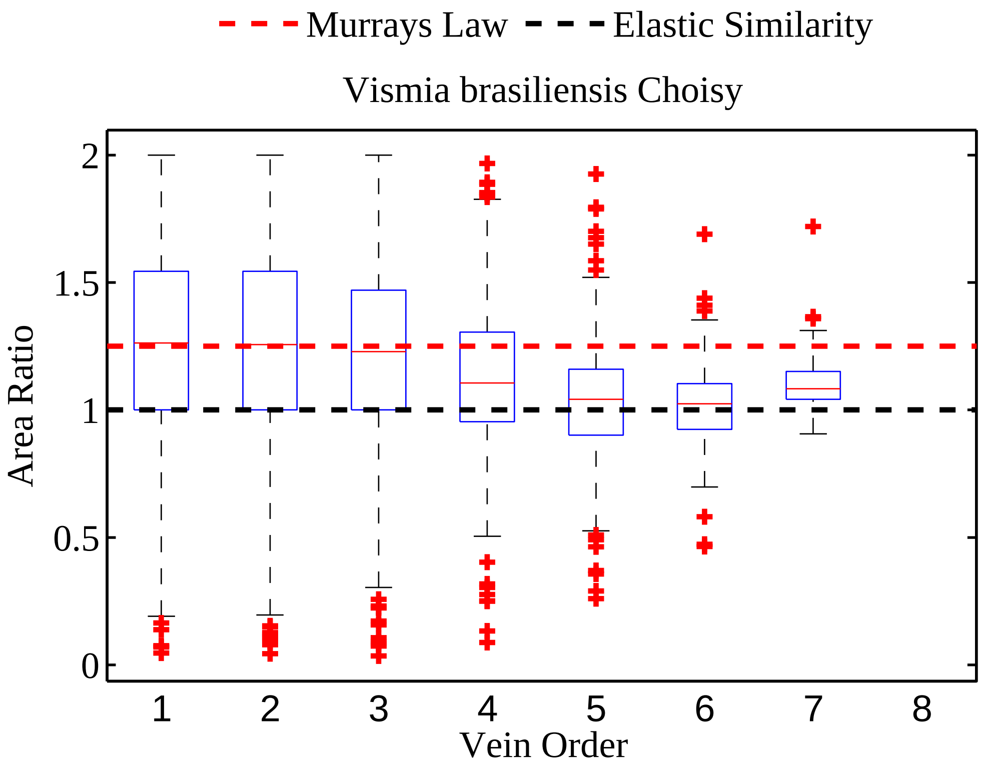

Murrays Law
Elastic Similarity
Vismia brasiliensis Choisy
Area Ratio
0
0.5
1
1.5
2
1
2
3
4
5
6
7
8
Vein Order

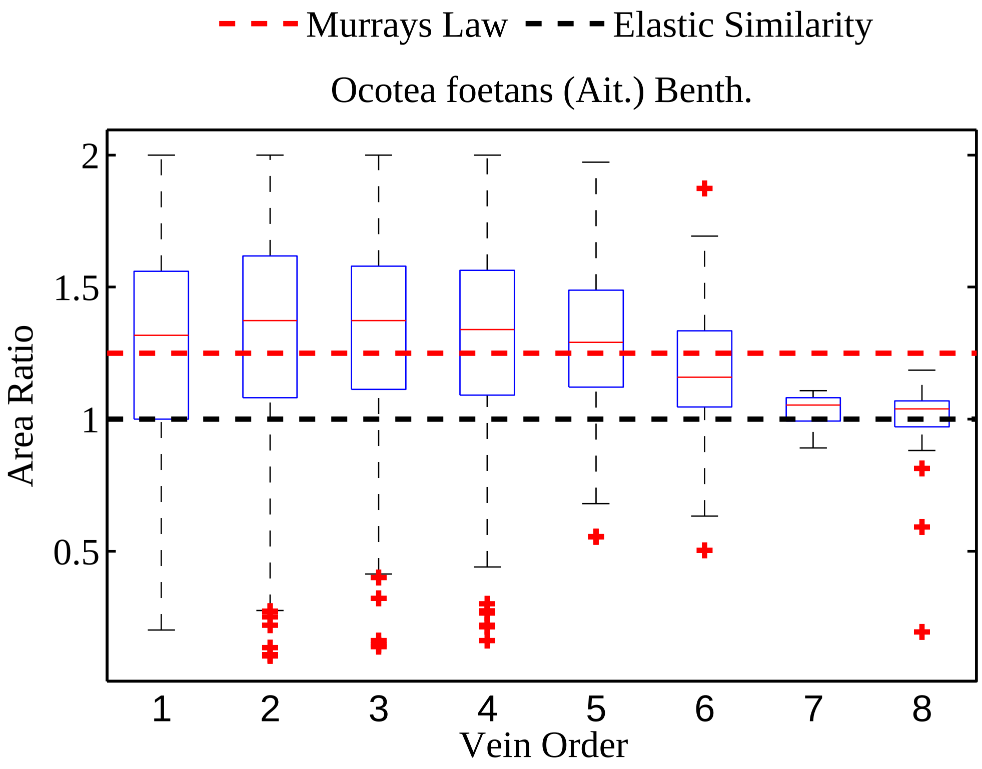
Murrays Law
Elastic Similarity
Ocotea foetans (Ait.) Benth.
Area Ratio
2
1.5
1
0.5
1
2
3
4
5
6
7
8
Vein Order

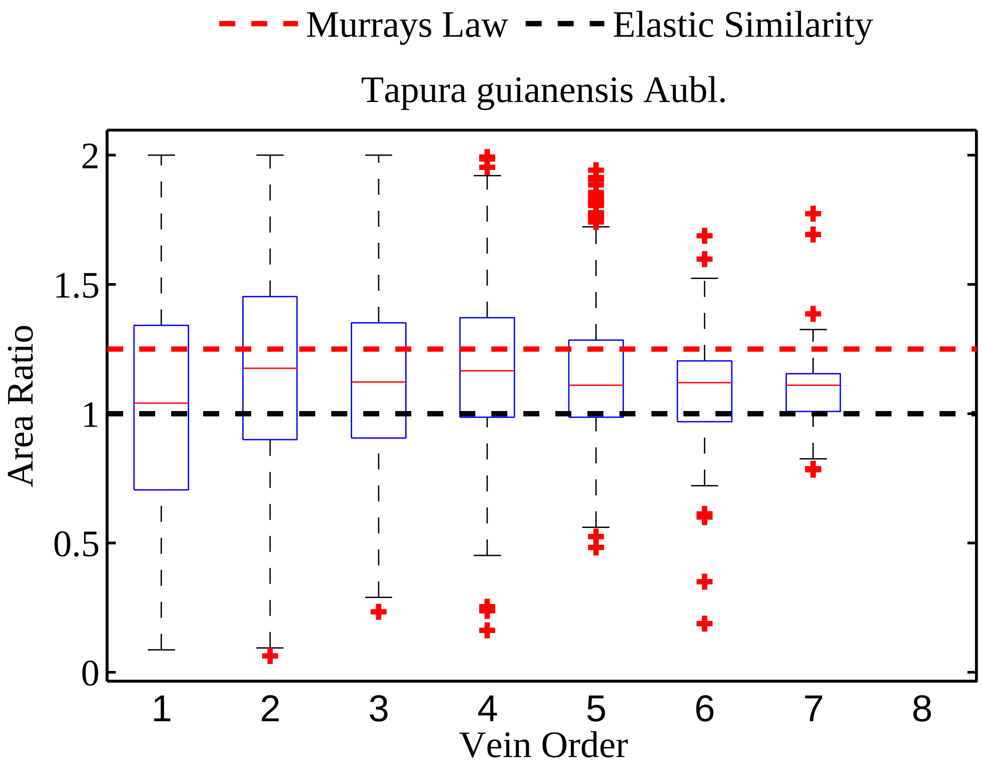

Murrays Law
Elastic Similarity
Tapura guianensis Aubl.
Area Ratio
0
0.5
1
1.5
2
1
2
3
4
5
6
7
8
Vein Order

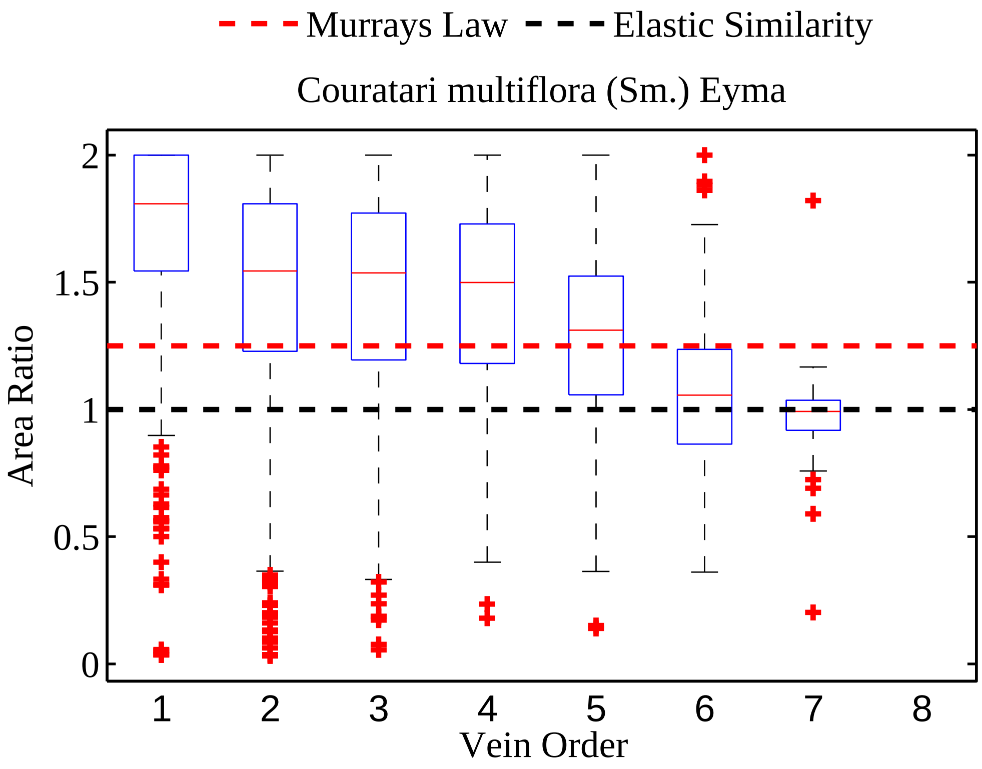
Murrays Law
Elastic Similarity
Couratari multiflora (Sm.) Eyma
Area Ratio
Vein Order
0
0.5
1
1.5
2
1
2
3
4
5
6
7
8

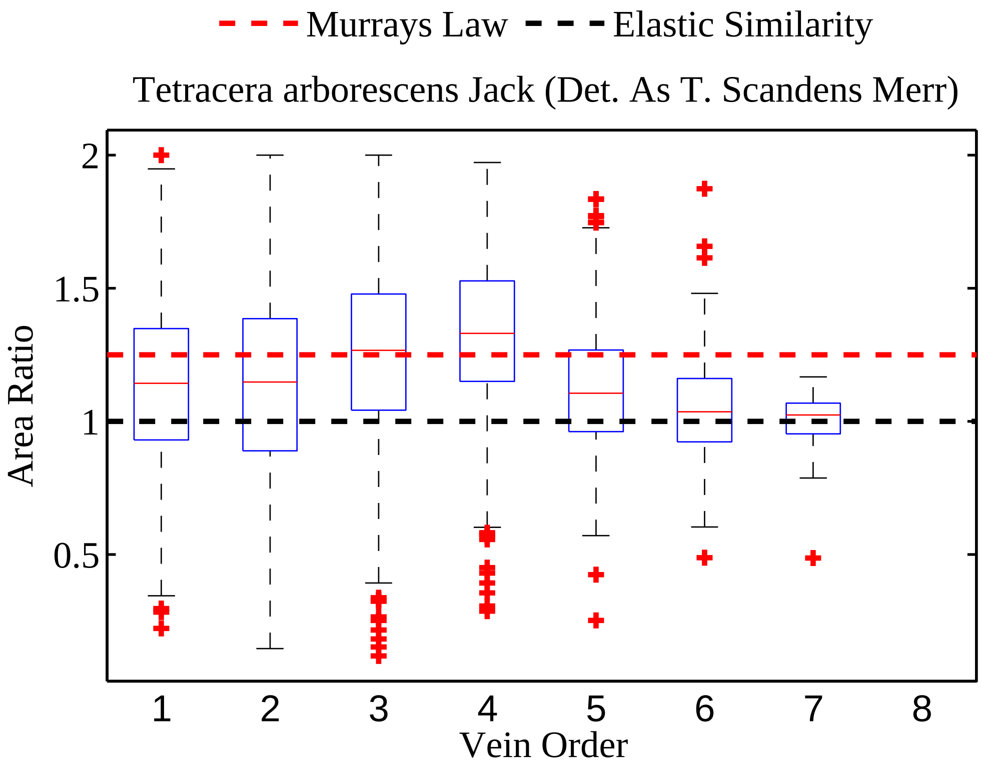
Murrays Law
Elastic Similarity
Tetracera arborescens Jack (Det. As T. Scandens Merr)
Area Ratio
2
1.5
1
0.5
1
2
3
4
5
6
7
8
Vein Order

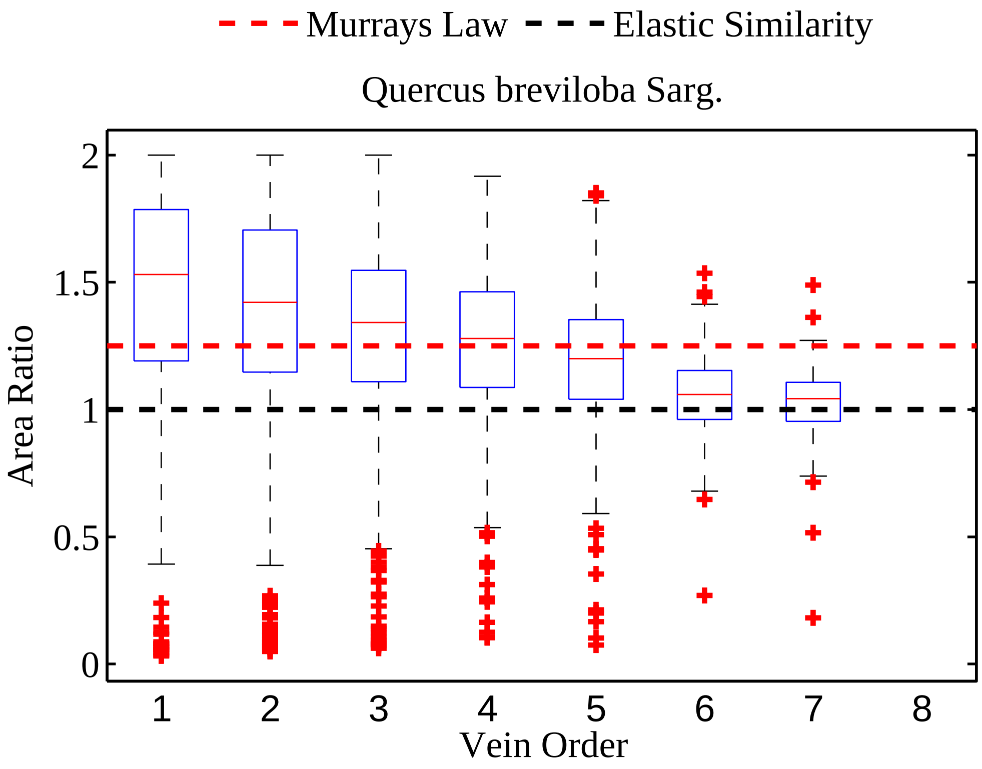

Murrays Law
Elastic Similarity
Quercus breviloba Sarg.
Area Ratio
0
0.5
1
1.5
2
1
2
3
4
5
6
7
8
Vein Order

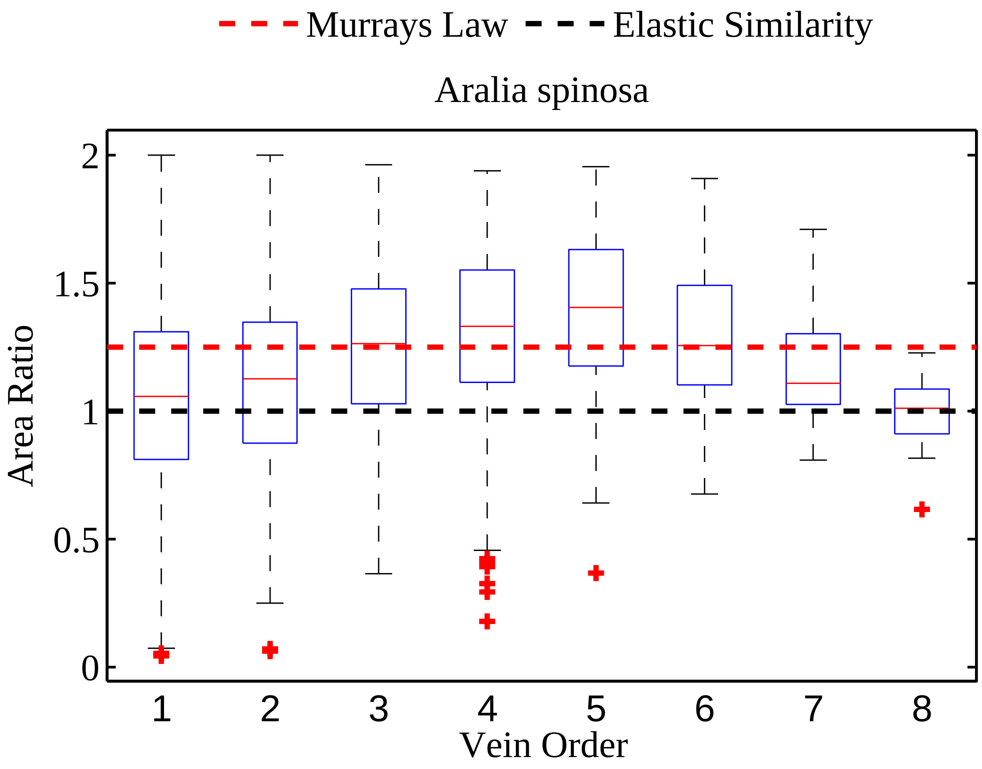

Murrays Law
Elastic Similarity
Aralia spinosa
Area Ratio
Vein Order
0
0.5
1
1.5
2
1
2
3
4
5
6
7
8

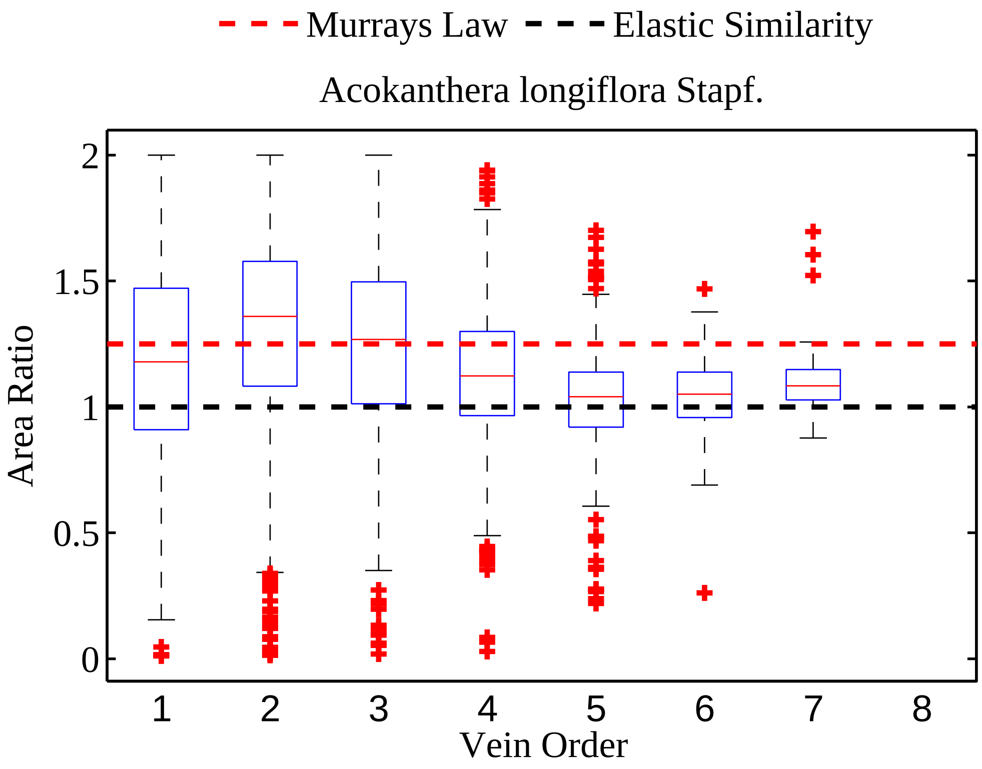

Murrays Law
Elastic Similarity
Acokanthera longiflora Stapf.
Area Ratio
2
1.5
1
0.5
0
1
2
3
4
5
6
7
8
Vein Order

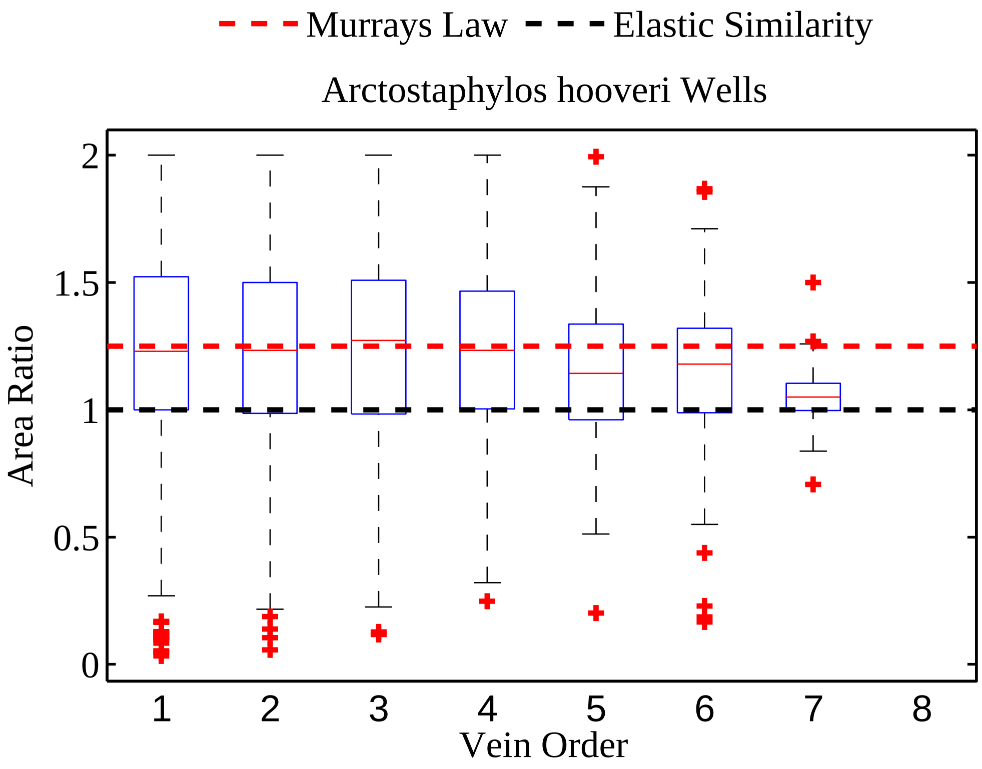

Murrays Law
Elastic Similarity
Arctostaphylos hooveri Wells
Area Ratio
0
0.5
1
1.5
2
1
2
3
4
5
6
7
8
Vein Order

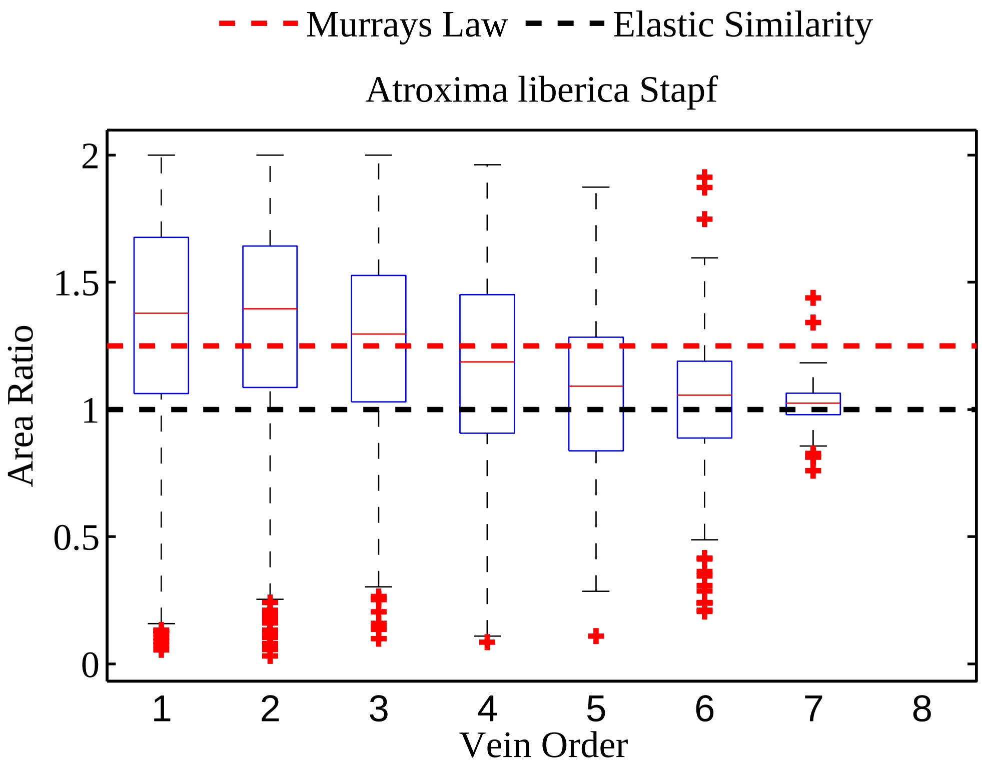
Murrays Law
Elastic Similarity
Atroxima liberica Stapf
Area Ratio
0
0.5
1
1.5
2
1
2
3
4
5
6
7
8
Vein Order

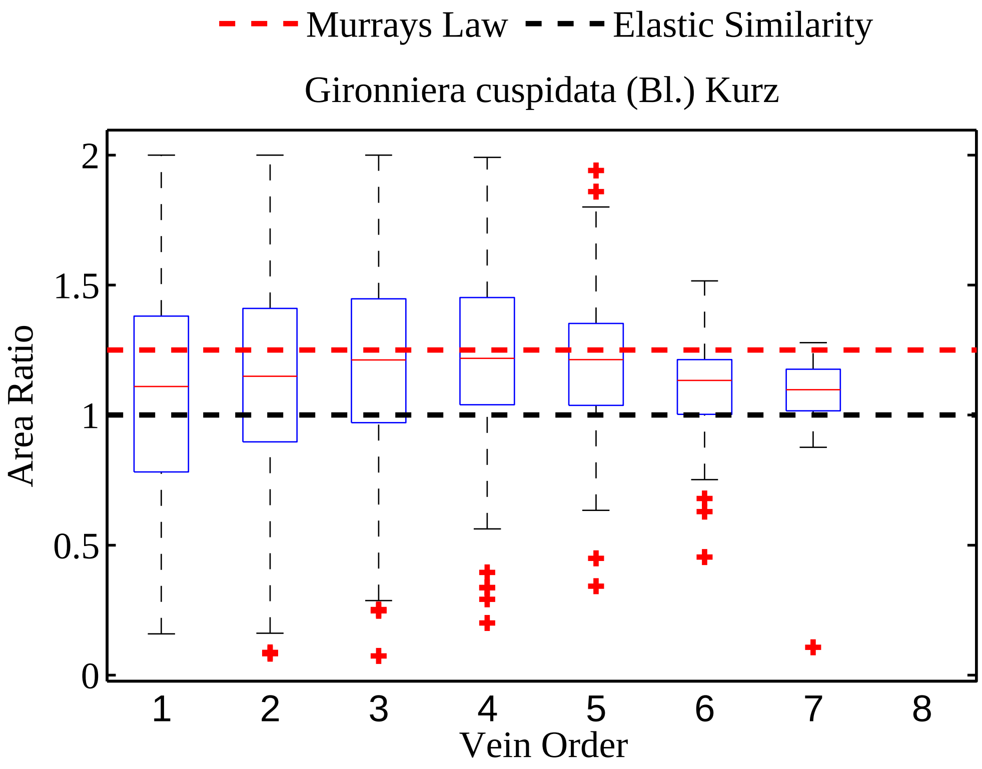

Murrays Law
Elastic Similarity
Gironniera cuspidata (Bl.) Kurz
Area Ratio
Vein Order
0
0.5
1
1.5
2
1
2
3
4
5
6
7
8

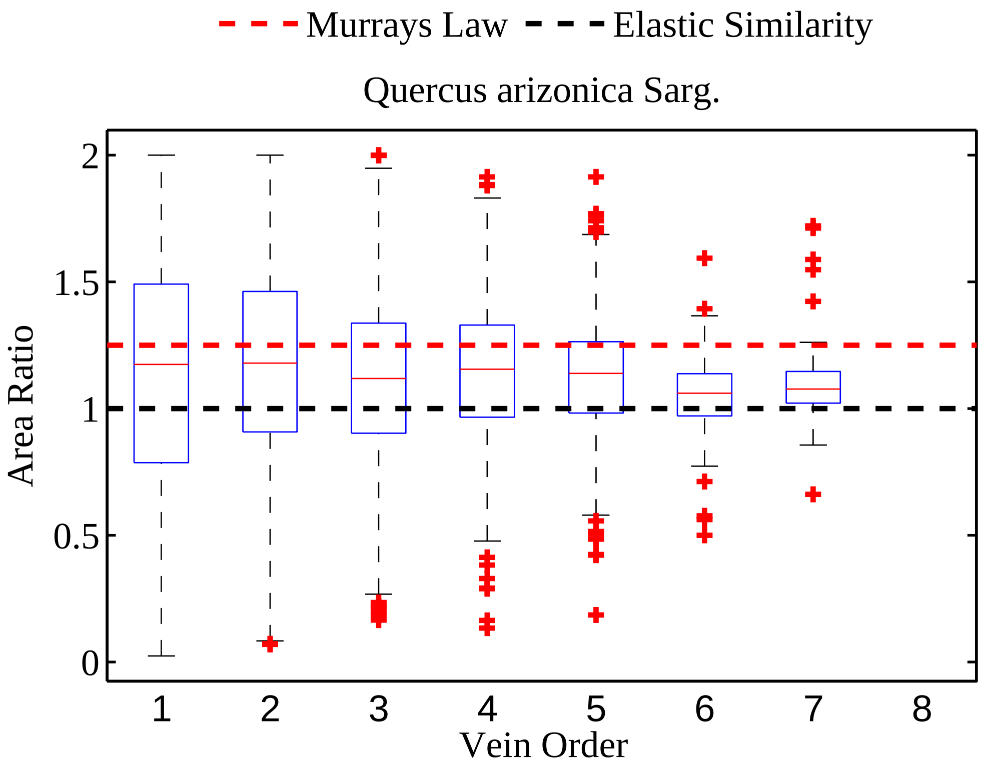

Murrays Law
Elastic Similarity
Quercus arizonica Sarg.
Area Ratio
0
0.5
1
1.5
2
1
2
3
4
5
6
7
8
Vein Order

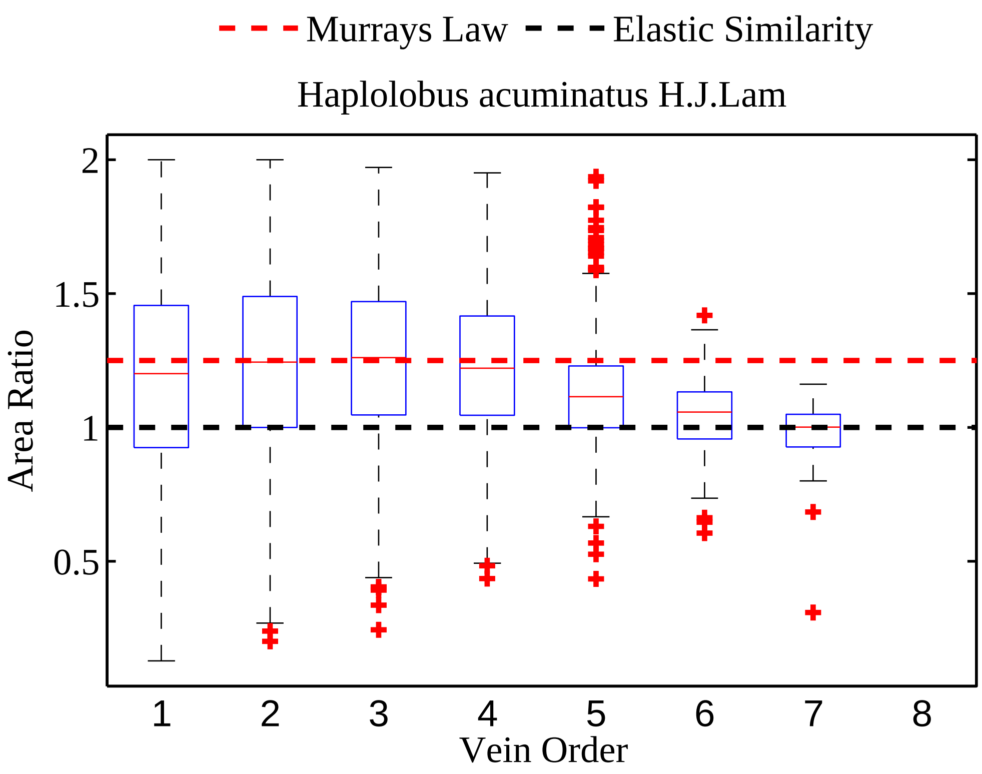
Murrays Law
Elastic Similarity
Haplolobus acuminatus H.J.Lam
Area Ratio
2
1.5
1
0.5
1
2
3
4
5
6
7
8
Vein Order

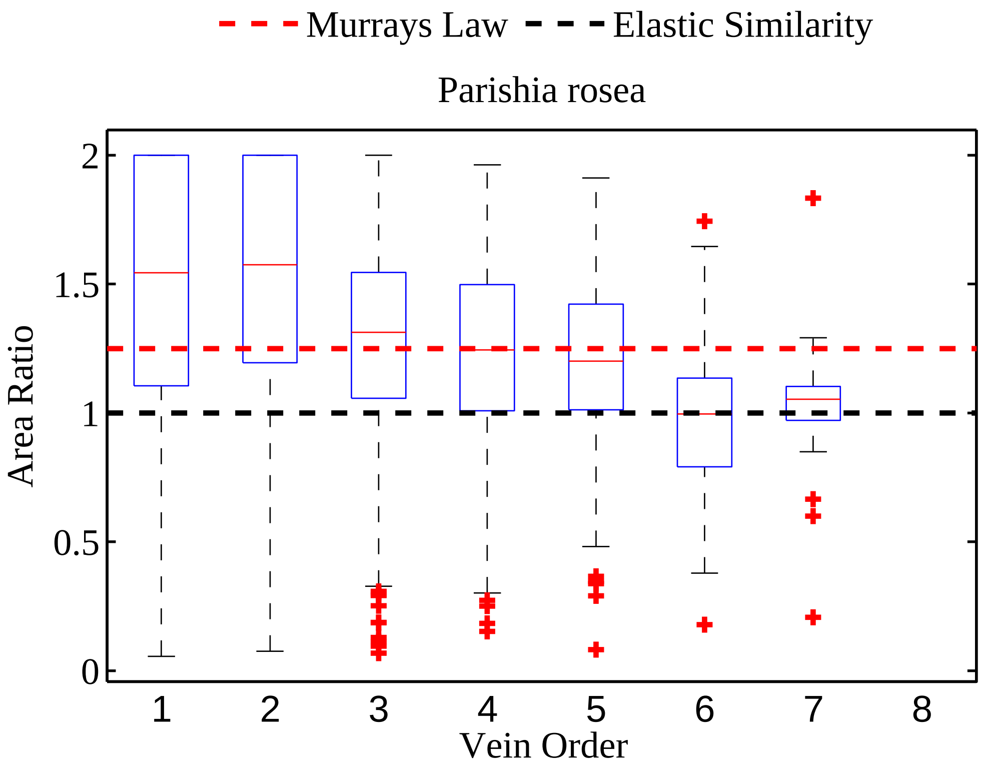

Murrays Law
Elastic Similarity
Parishia rosea
Area Ratio
Vein Order
0
0.5
1
1.5
2
1
2
3
4
5
6
7
8

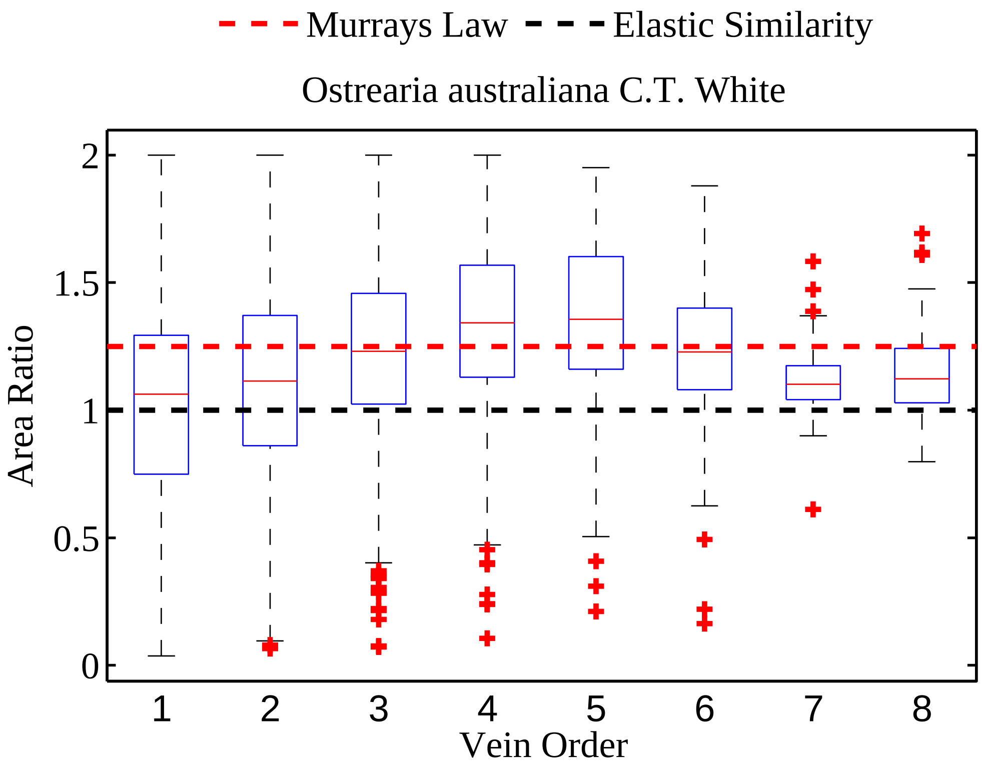
Murrays Law
Elastic Similarity
Ostrearia australiana C.T. White
Area Ratio
0
0.5
1
1.5
2
1
2
3
4
5
6
7
8
Vein Order

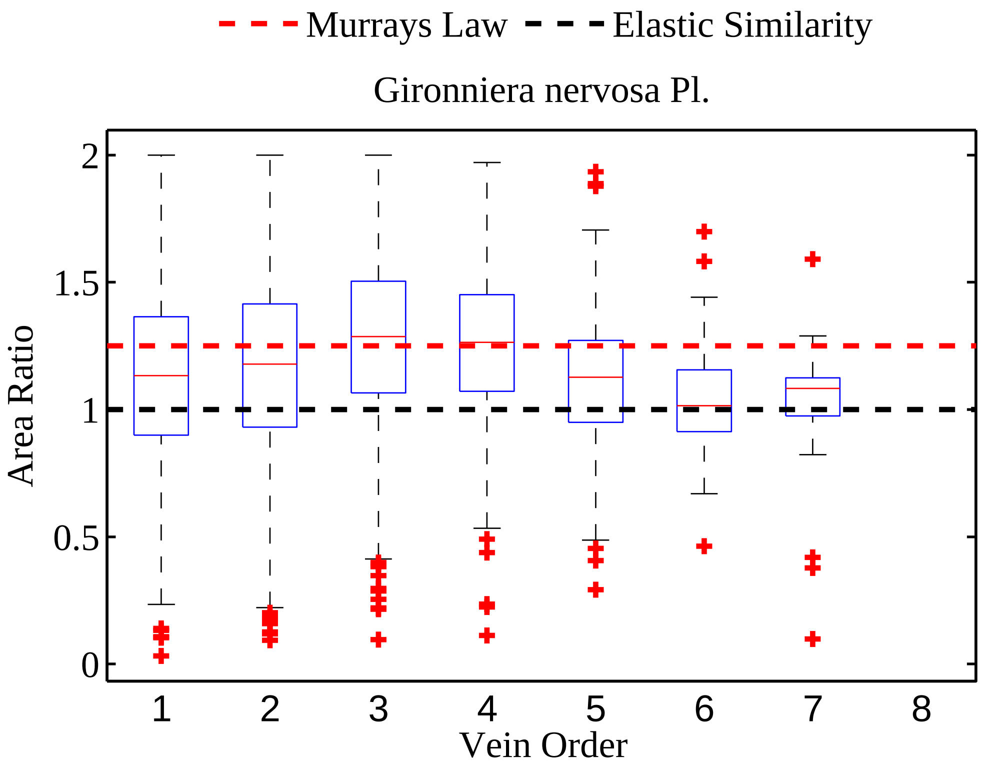
Murrays Law
Elastic Similarity
Gironniera nervosa Pl.
Area Ratio
Vein Order
0
0.5
1
1.5
2
1
2
3
4
5
6
7
8

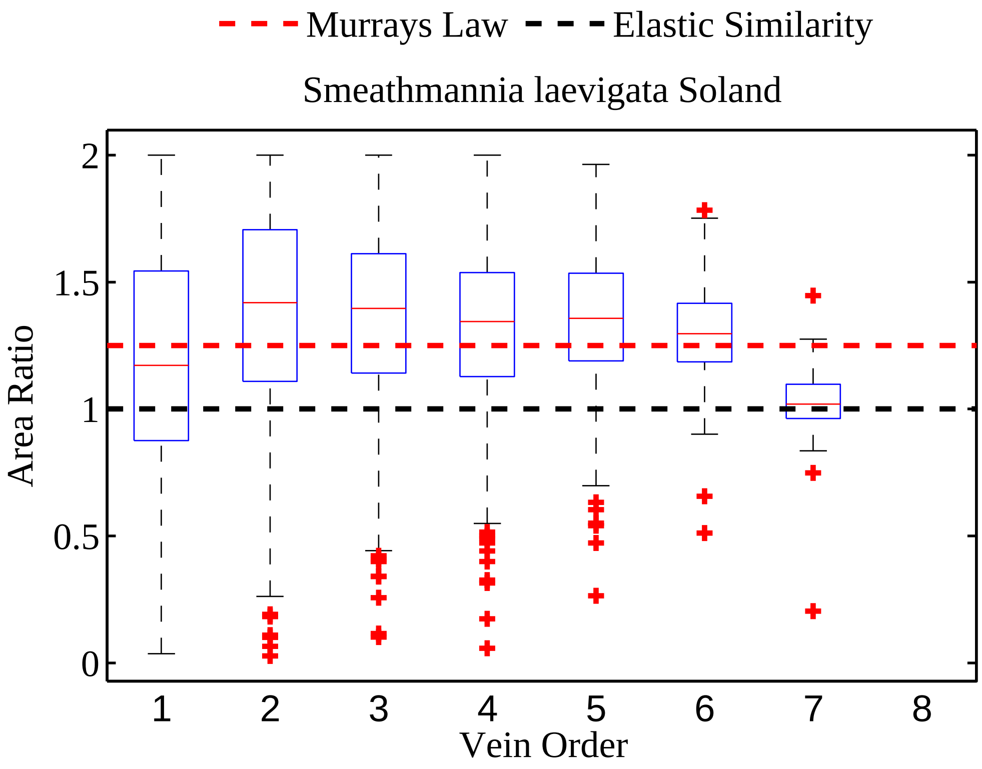

Murrays Law
Elastic Similarity
Smeathmannia laevigata Soland
Area Ratio
2
1.5
1
0.5
0
1
2
3
4
5
6
7
8
Vein Order

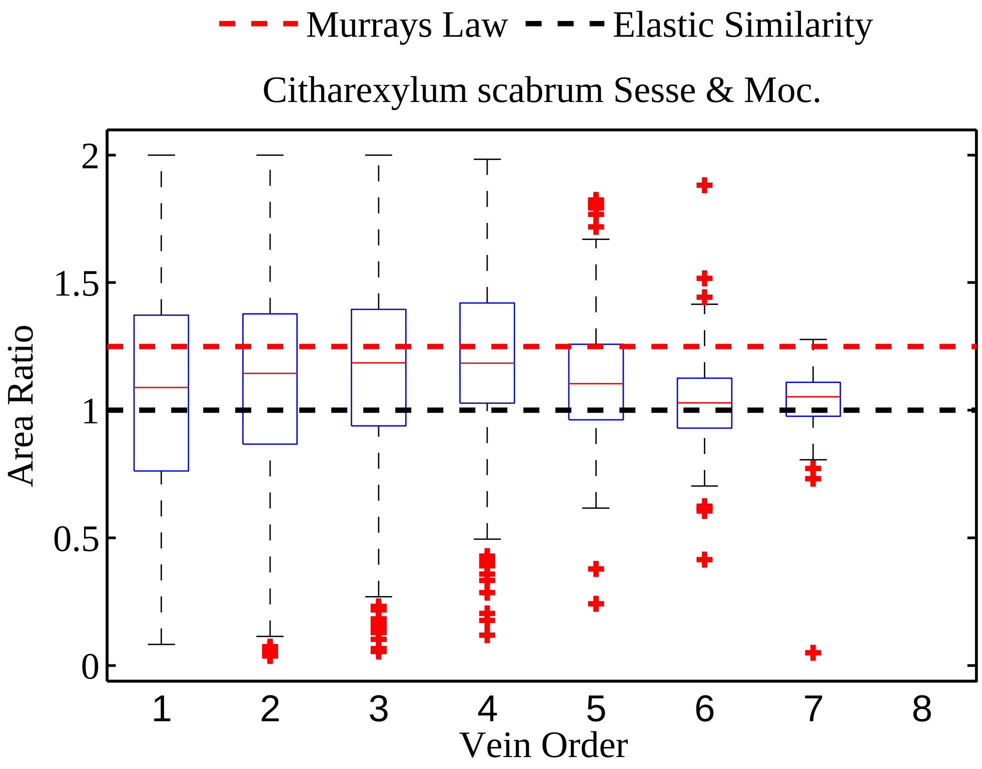

Murrays Law
Elastic Similarity
Citharexylum scabrum Sesse & Moc.
Area Ratio
0
0.5
1
1.5
2
1
2
3
4
5
6
7
8
Vein Order

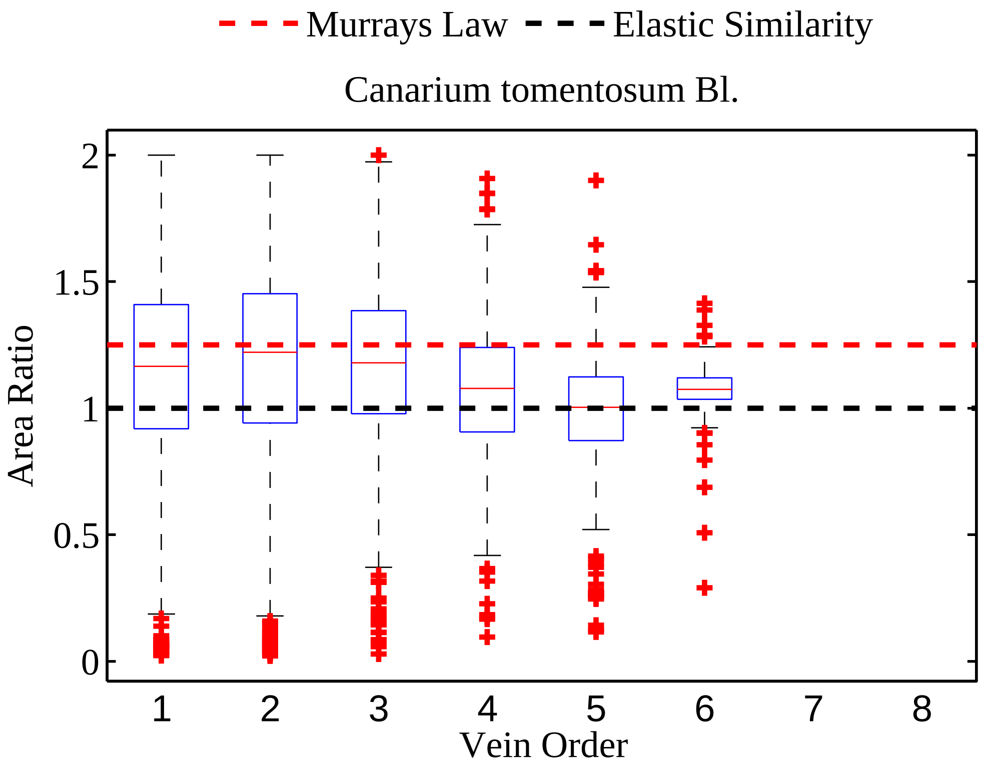

Murrays Law
Elastic Similarity
Canarium tomentosum Bl.
Area Ratio
Vein Order
0
0.5
1
1.5
2
1
2
3
4
5
6
7
8

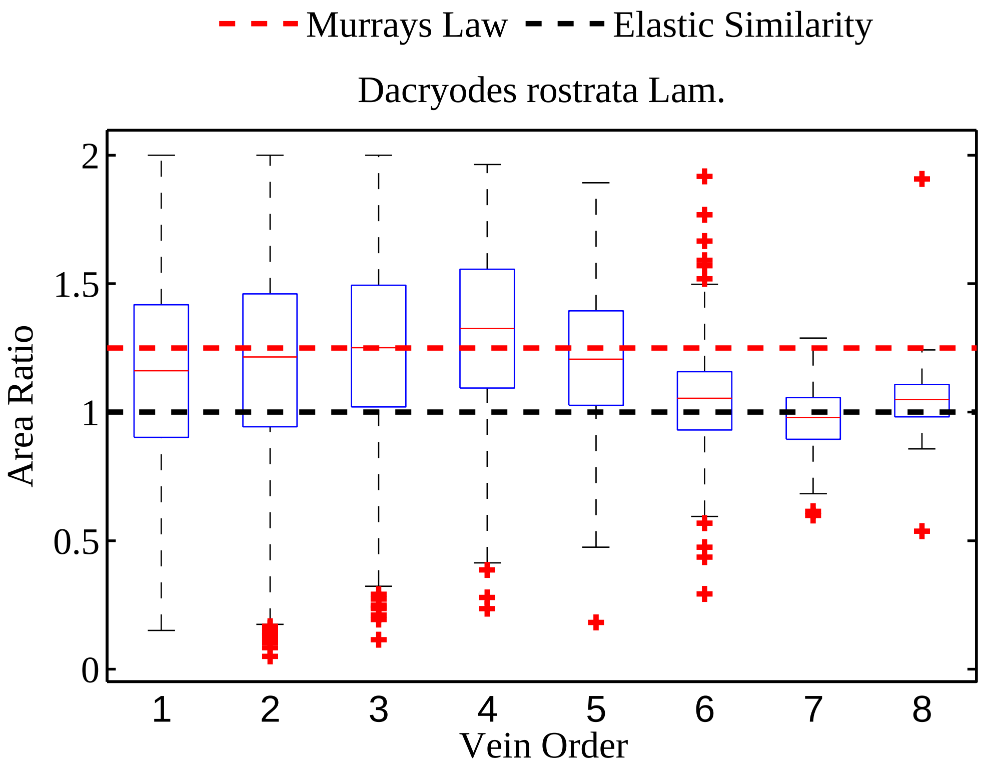
Murrays Law
Elastic Similarity
Dacryodes rostrata Lam.
Area Ratio
Vein Order
2
1.5
1
0.5
0
1
2
3
4
5
6
7
8

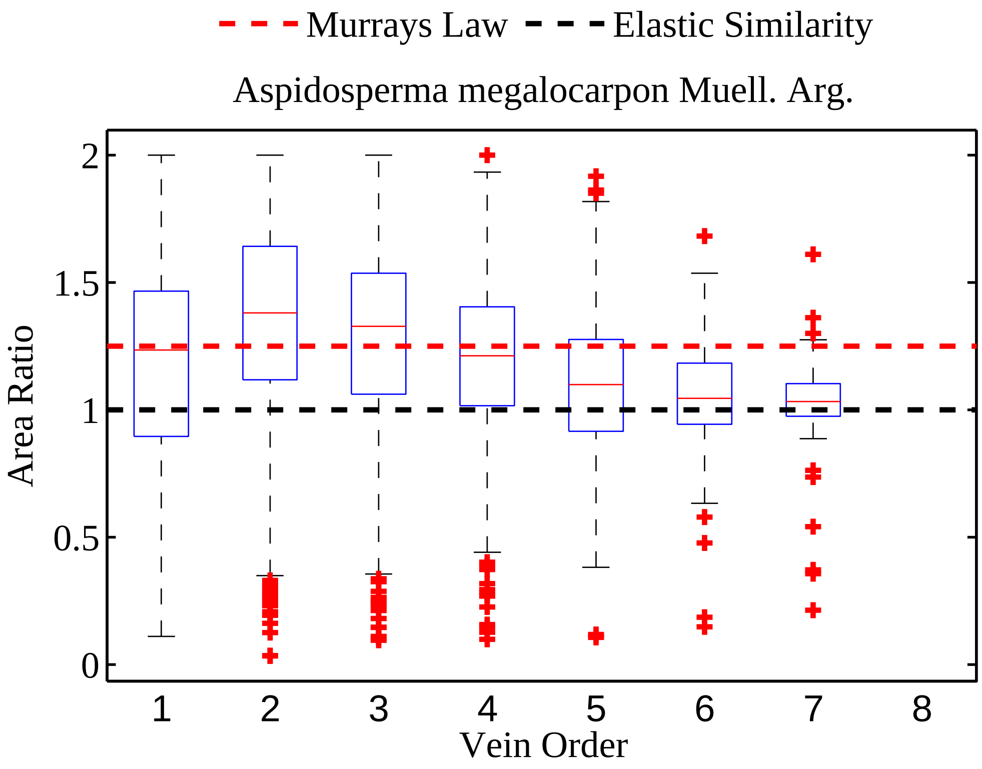

Murrays Law
Elastic Similarity
Aspidosperma megalocarpon Muell. Arg.
Area Ratio
0
0.5
1
1.5
2
1
2
3
4
5
6
7
8
Vein Order

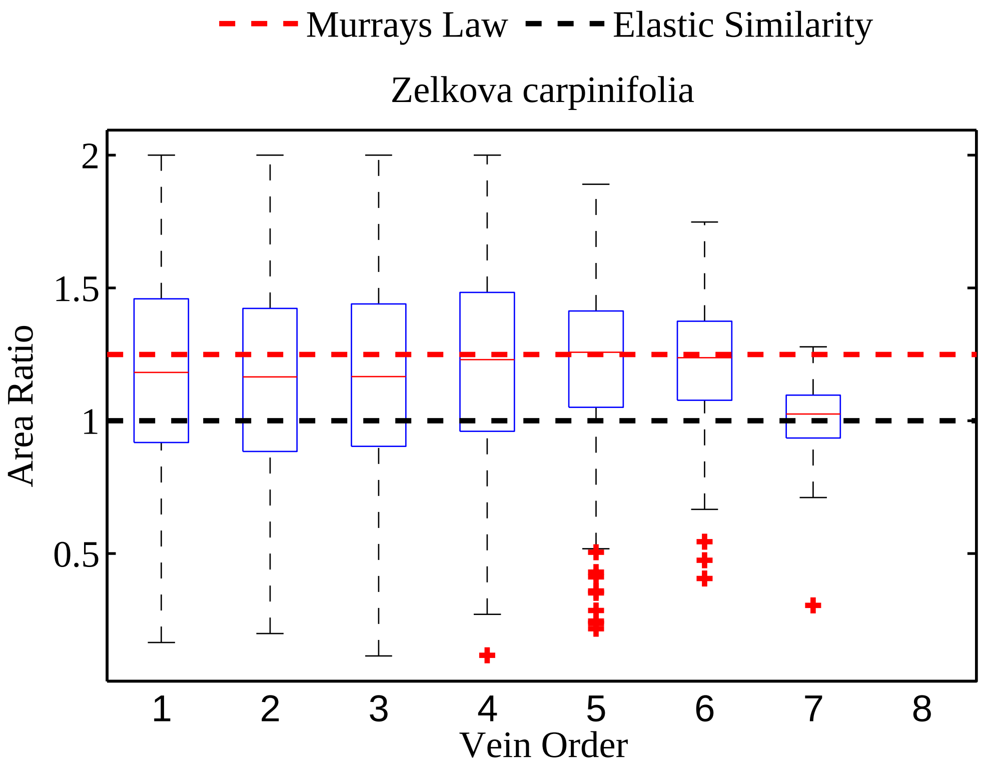
Murrays Law
Elastic Similarity
Zelkova carpinifolia
Area Ratio
Vein Order
2
1.5
1
0.5
1
2
3
4
5
6
7
8

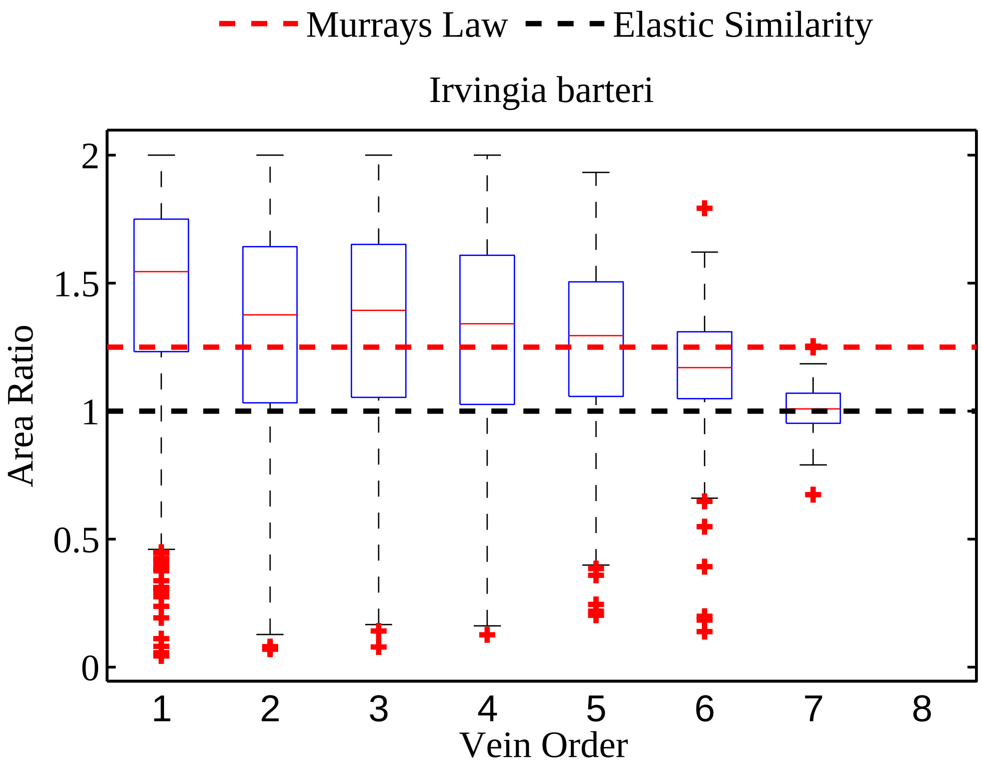

Murrays Law
Elastic Similarity
Irvingia barteri
Area Ratio
2
1.5
1
0.5
0
1
2
3
4
5
6
7
8
Vein Order

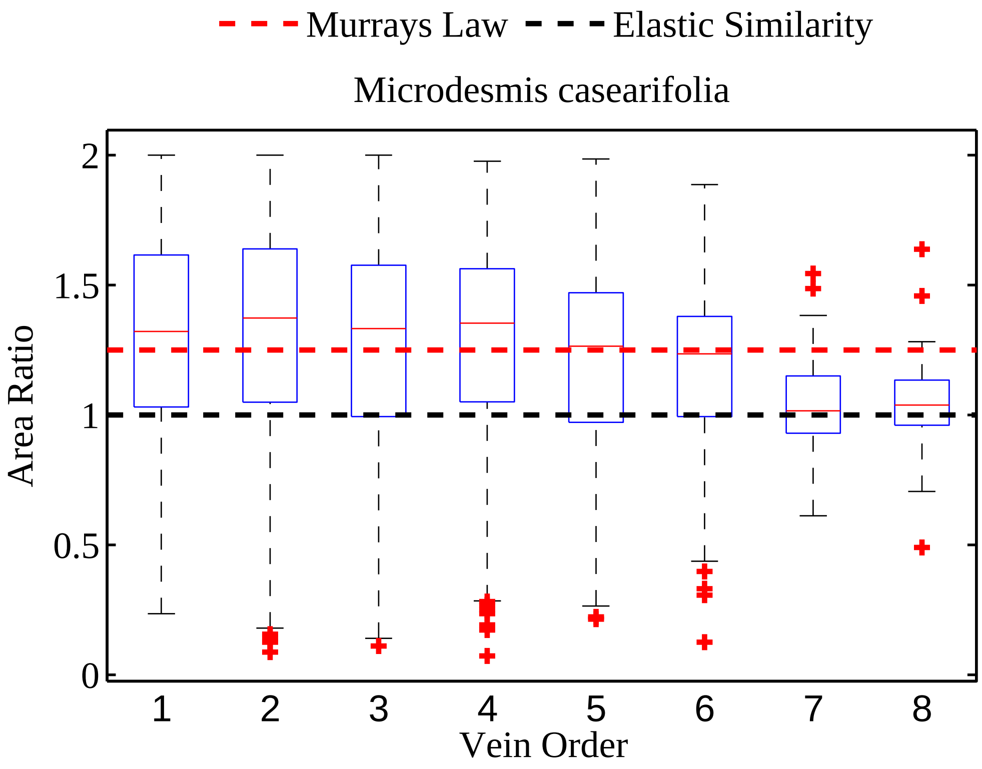

Murrays Law
Elastic Similarity
Microdesmis casearifolia
Area Ratio
0
0.5
1
1.5
2
1
2
3
4
5
6
7
8
Vein Order

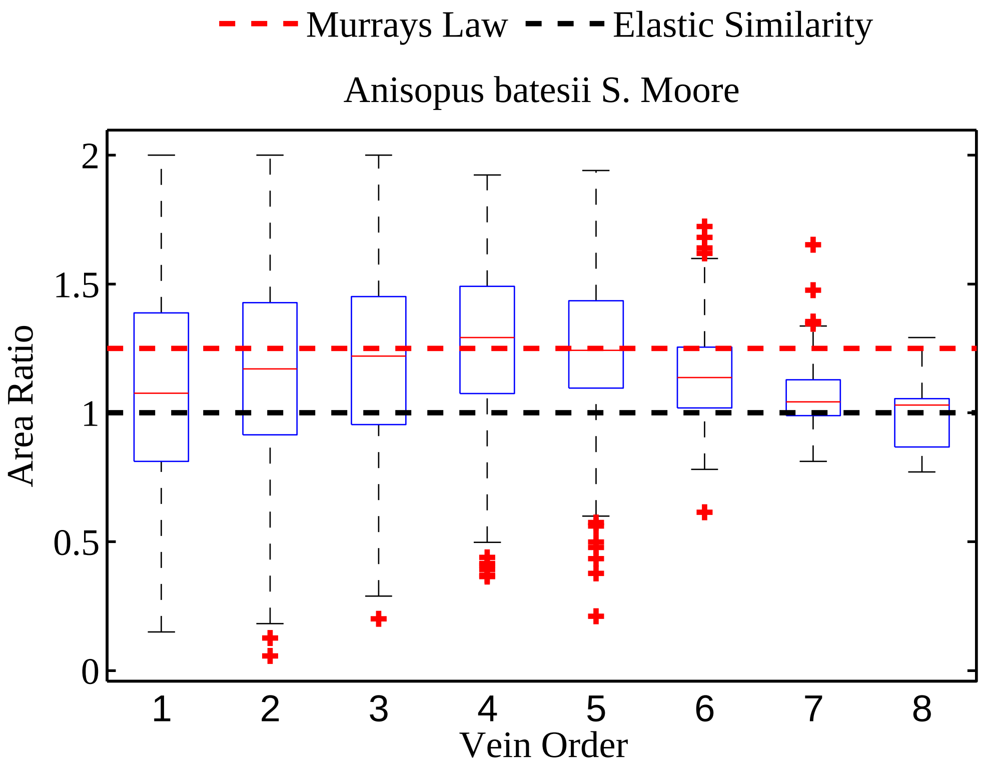

Murrays Law
Elastic Similarity
Anisopus batesii S. Moore
Area Ratio
Vein Order
0
0.5
1
1.5
2
1
2
3
4
5
6
7
8

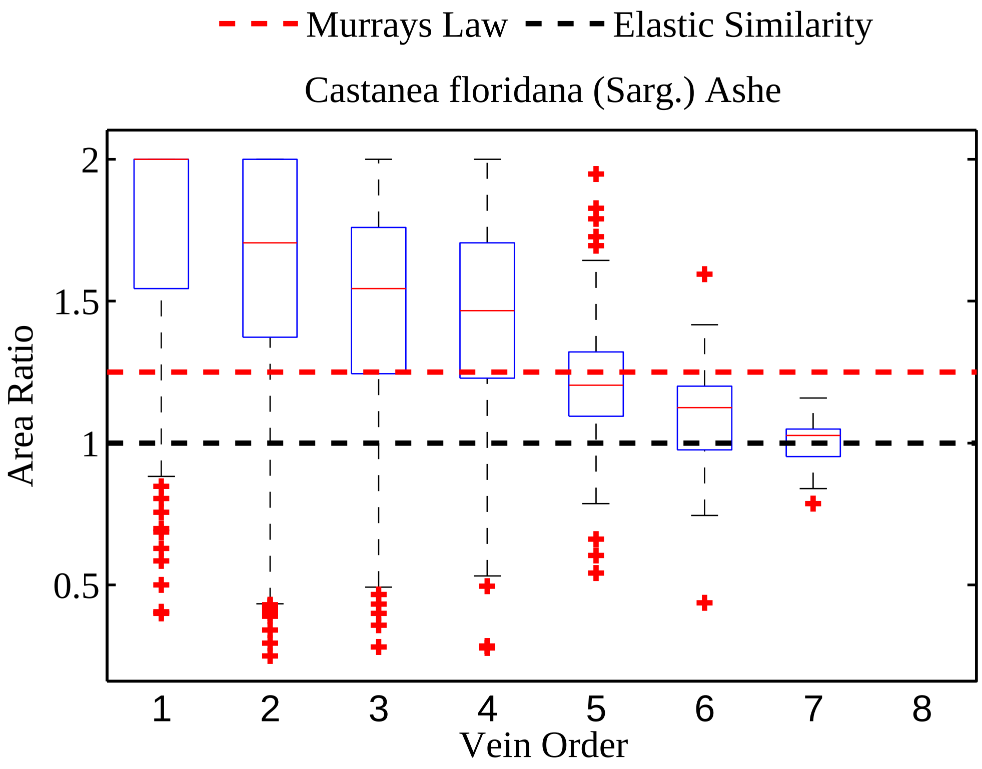

Murrays Law
Elastic Similarity
Castanea floridana (Sarg.) Ashe
Area Ratio
2
1.5
1
0.5
1
2
3
4
5
6
7
8
Vein Order

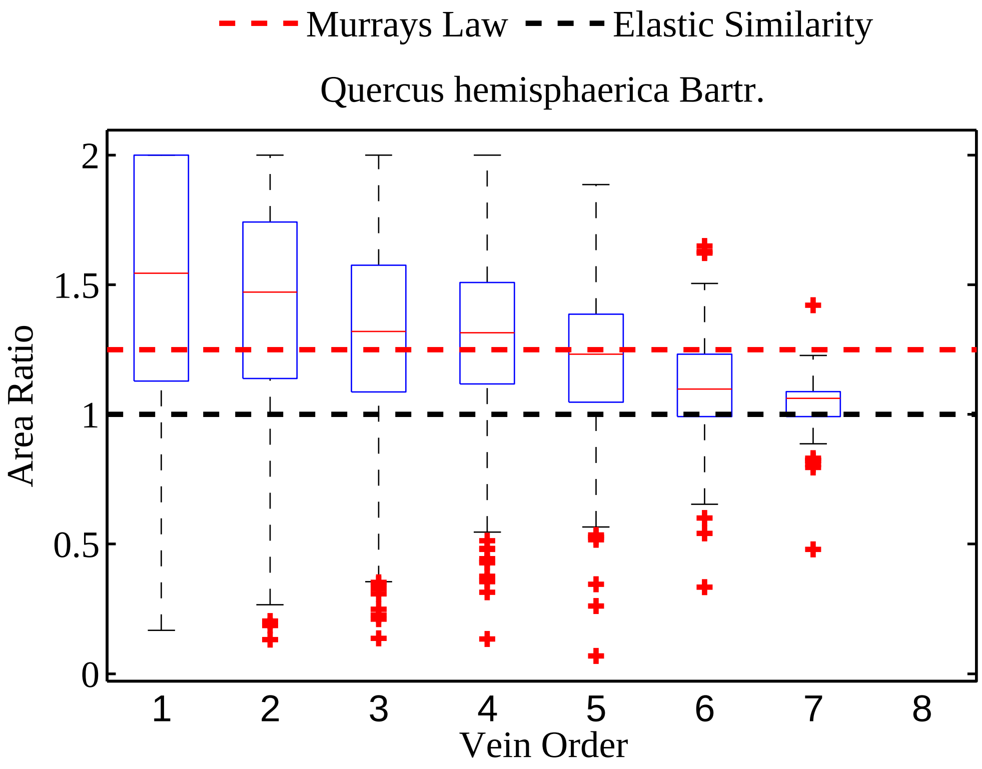

Murrays Law
Elastic Similarity
Quercus hemisphaerica Bartr.
Area Ratio
Vein Order
0
0.5
1
1.5
2
1
2
3
4
5
6
7
8

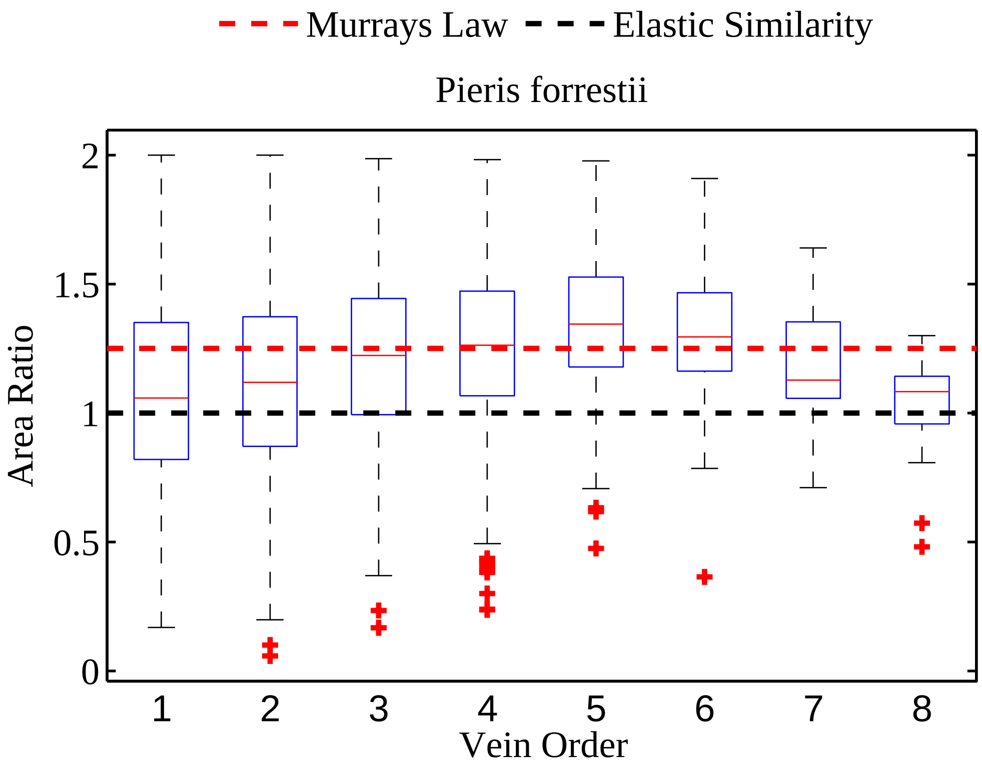

Murrays Law
Elastic Similarity
Pieris forrestii
Area Ratio
Vein Order
0
0.5
1
1.5
2
1
2
3
4
5
6
7
8

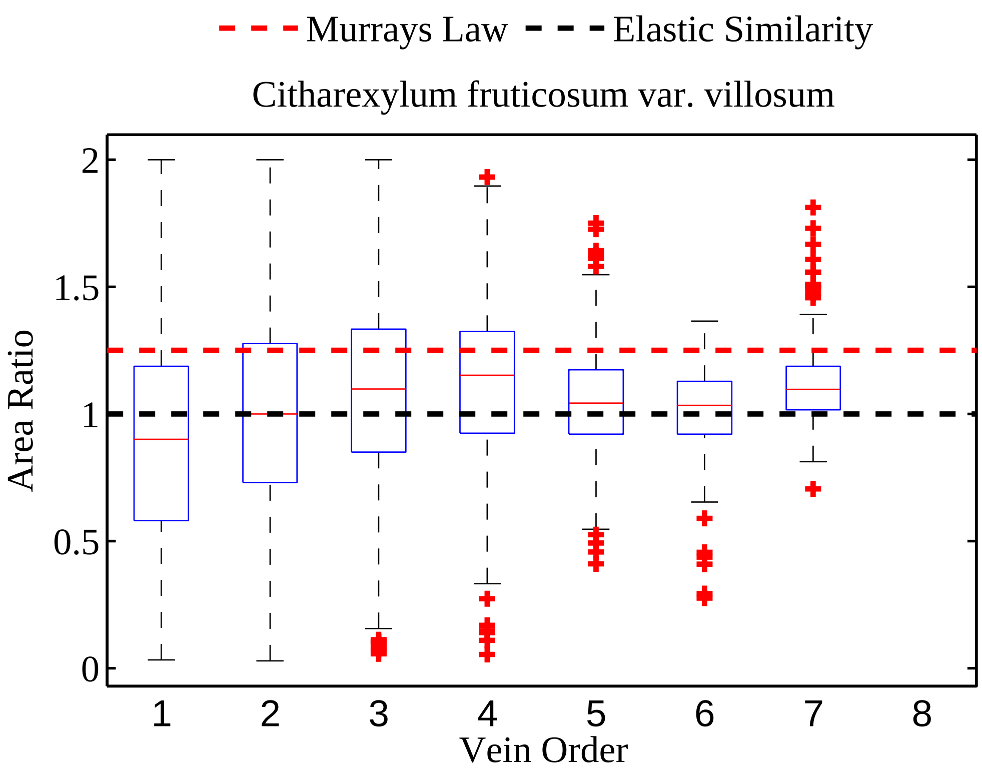

Murrays Law
Elastic Similarity
Citharexylum fruticosum var. villosum
Area Ratio
0
0.5
1
1.5
2
1
2
3
4
5
6
7
8
Vein Order

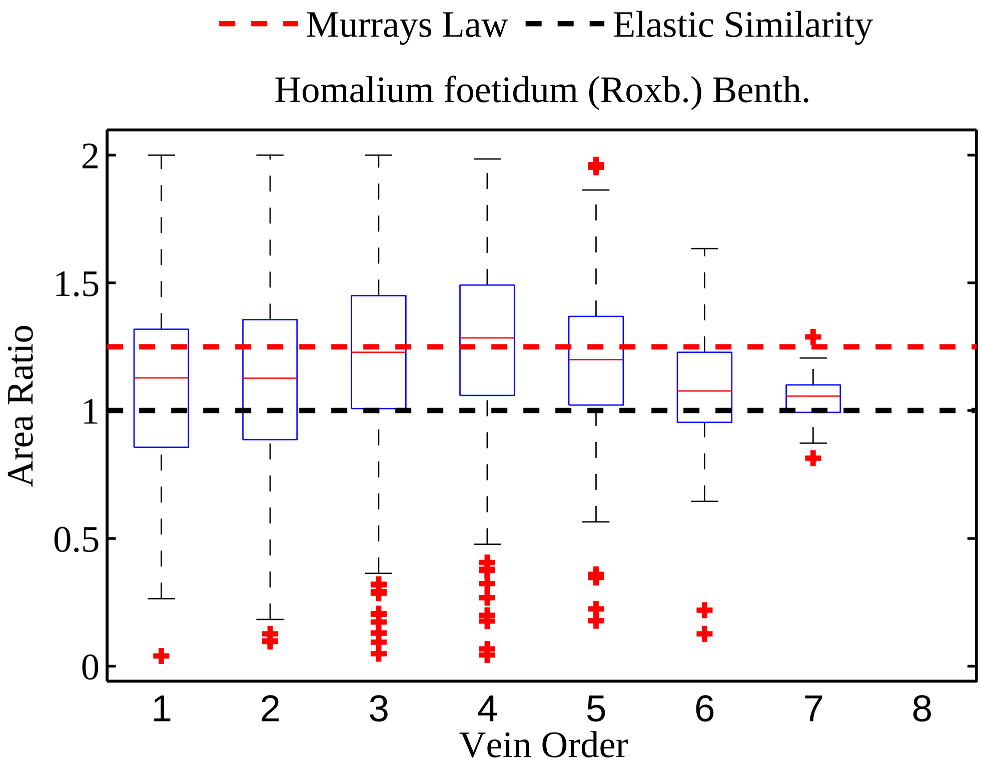

Murrays Law
Elastic Similarity
Homalium foetidum (Roxb.) Benth.
Area Ratio
0
0.5
1
1.5
2
1
2
3
4
5
6
7
8
Vein Order

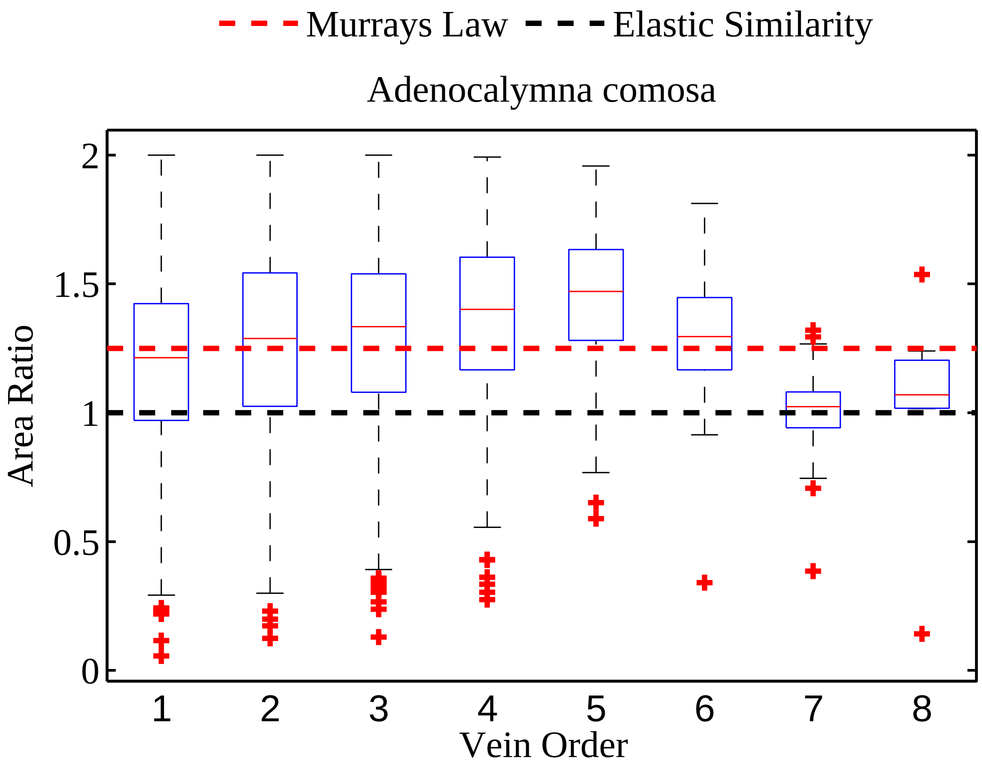

Murrays Law
Elastic Similarity
Adenocalymna comosa
Area Ratio
2
1.5
1
0.5
0
1
2
3
4
5
6
7
8
Vein Order

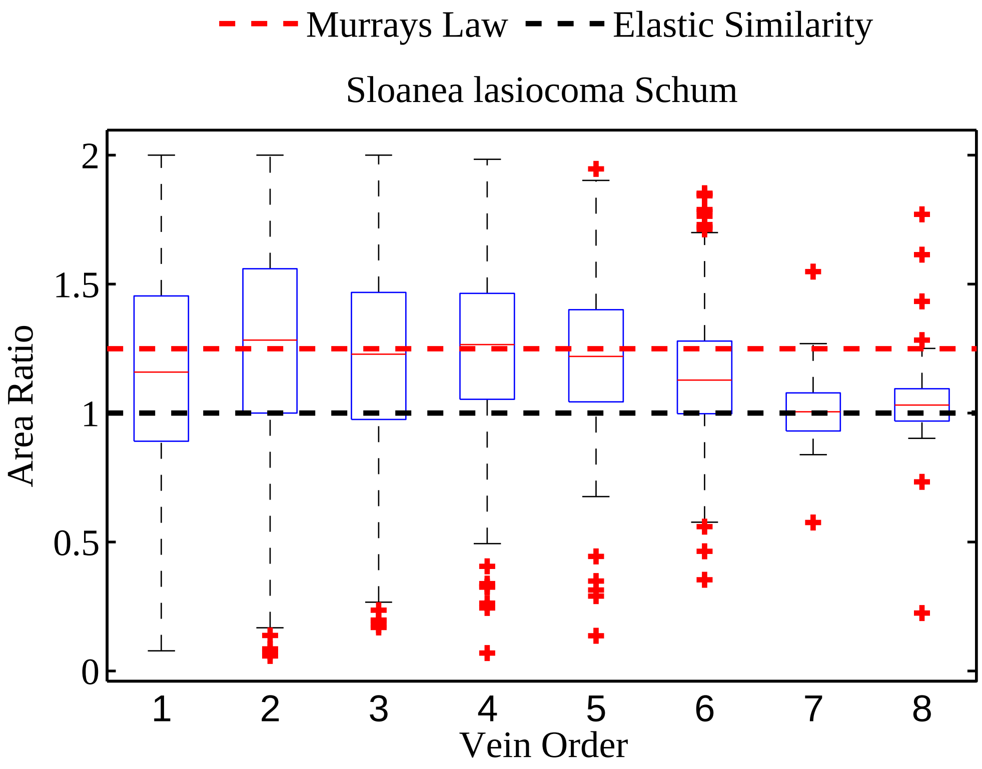

Murrays Law
Elastic Similarity
Sloanea lasiocoma Schum
Area Ratio
Vein Order
0
0.5
1
1.5
2
1
2
3
4
5
6
7
8

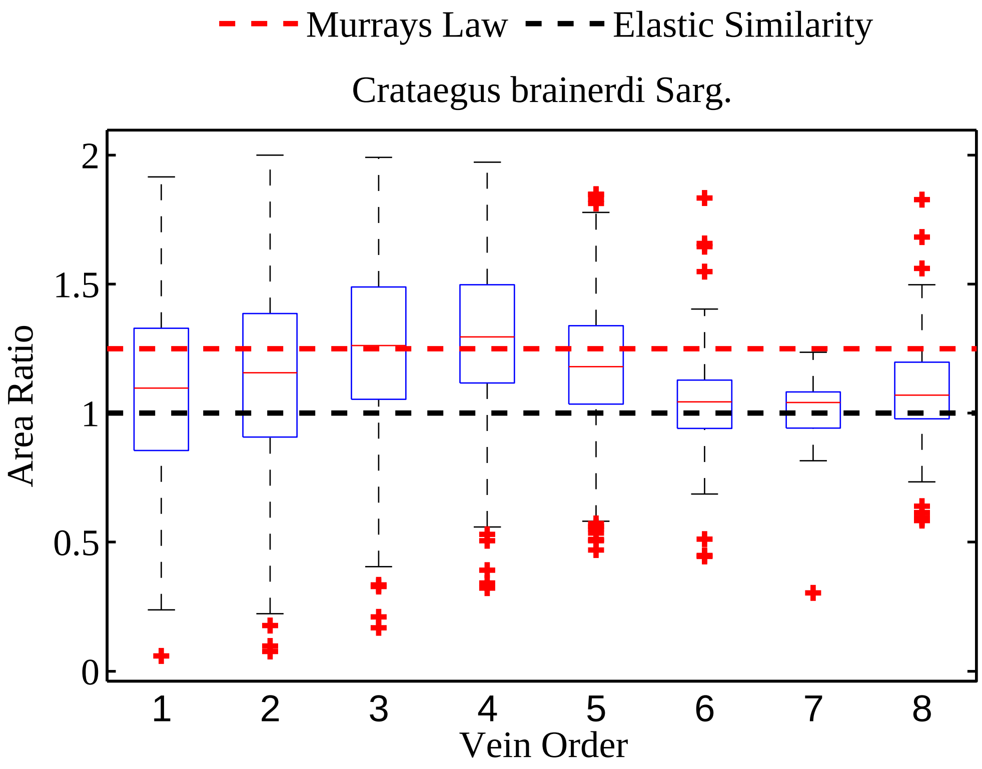

Murrays Law
Elastic Similarity
Crataegus brainerdi Sarg.
Area Ratio
Vein Order
0
0.5
1
1.5
2
1
2
3
4
5
6
7
8

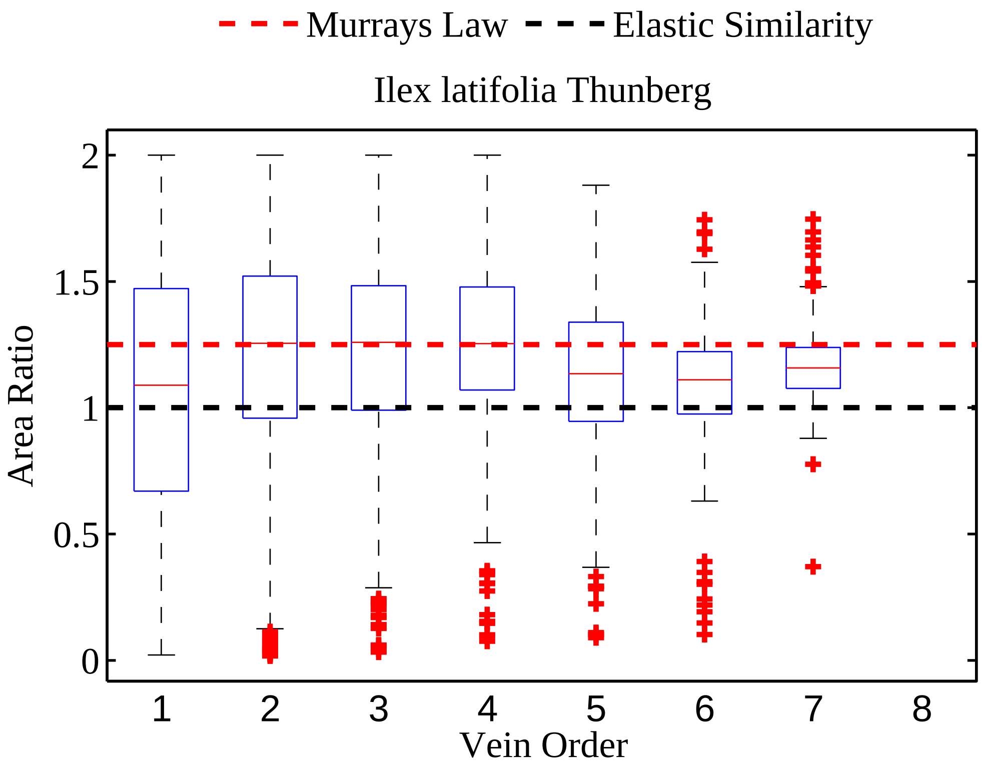

Murrays Law
Elastic Similarity
Ilex latifolia Thunberg
Area Ratio
0
0.5
1
1.5
2
1
2
3
4
5
6
7
8
Vein Order

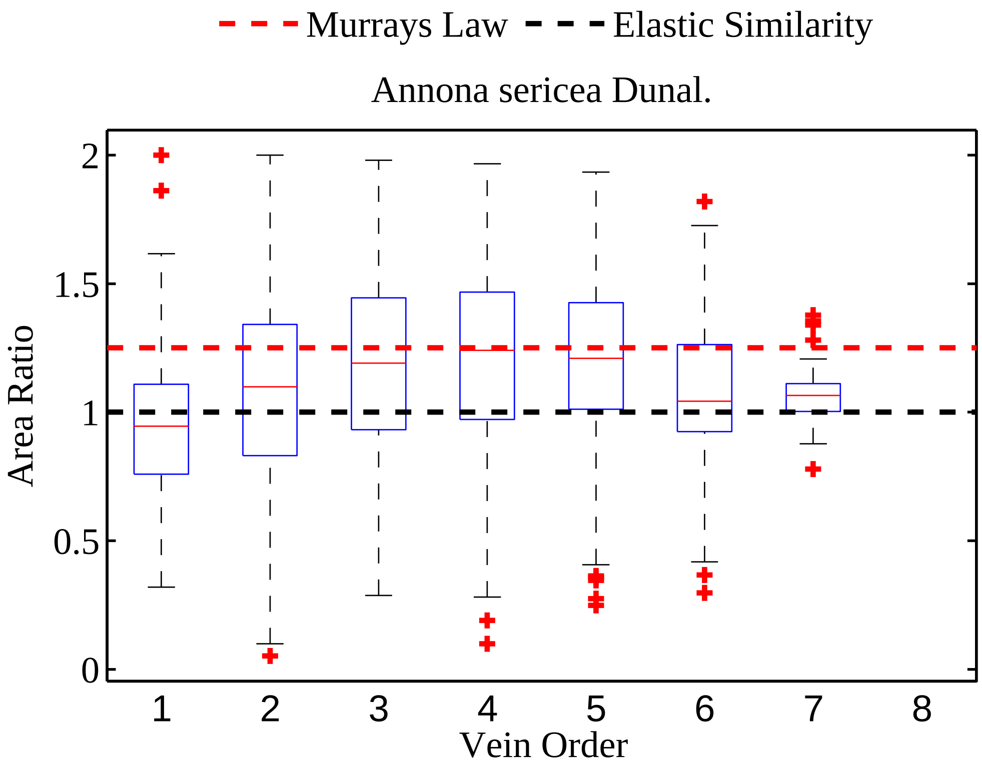

Murrays Law
Elastic Similarity
Annona sericea Dunal.
Area Ratio
0
0.5
1
1.5
2
1
2
3
4
5
6
7
8
Vein Order

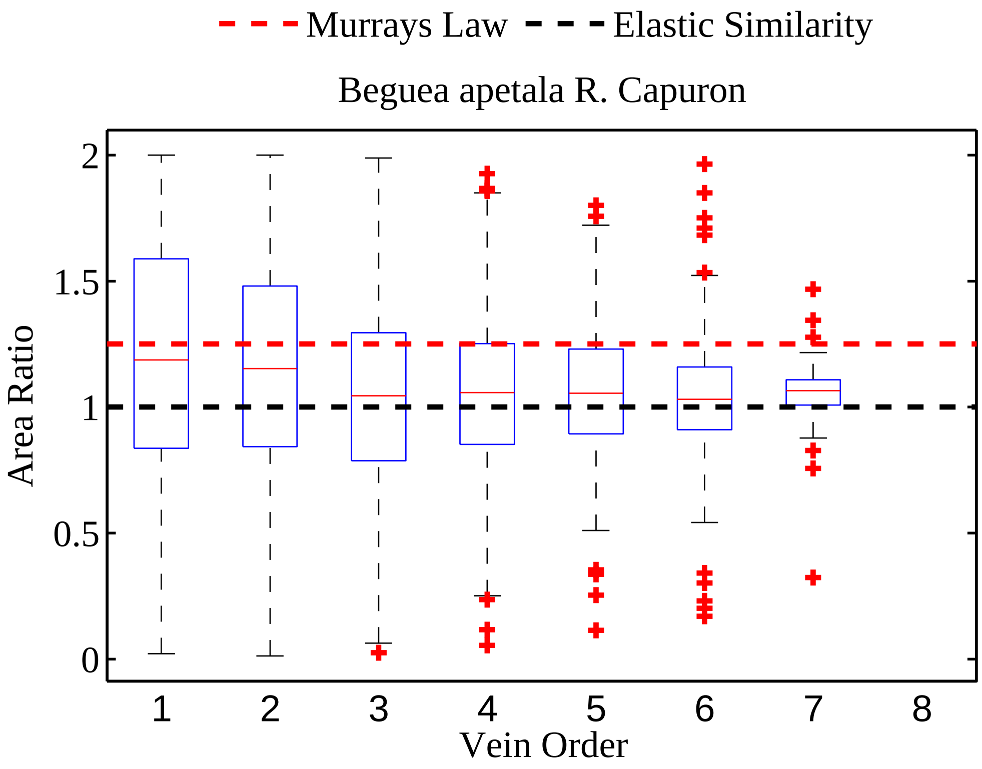

Murrays Law
Elastic Similarity
Beguea apetala R. Capuron
Area Ratio
0
0.5
1
1.5
2
1
2
3
4
5
6
7
8
Vein Order

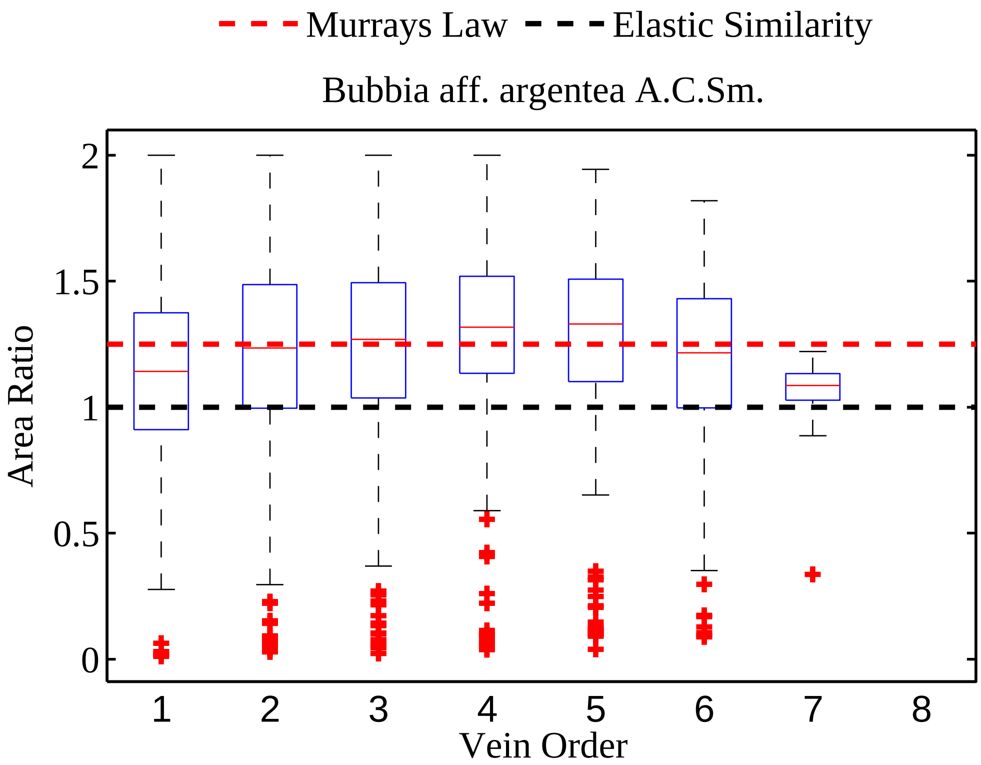

Murrays Law
Elastic Similarity
Bubbia aff. argentea A.C.Sm.
Area Ratio
0
0.5
1
1.5
2
1
2
3
4
5
6
7
8
Vein Order

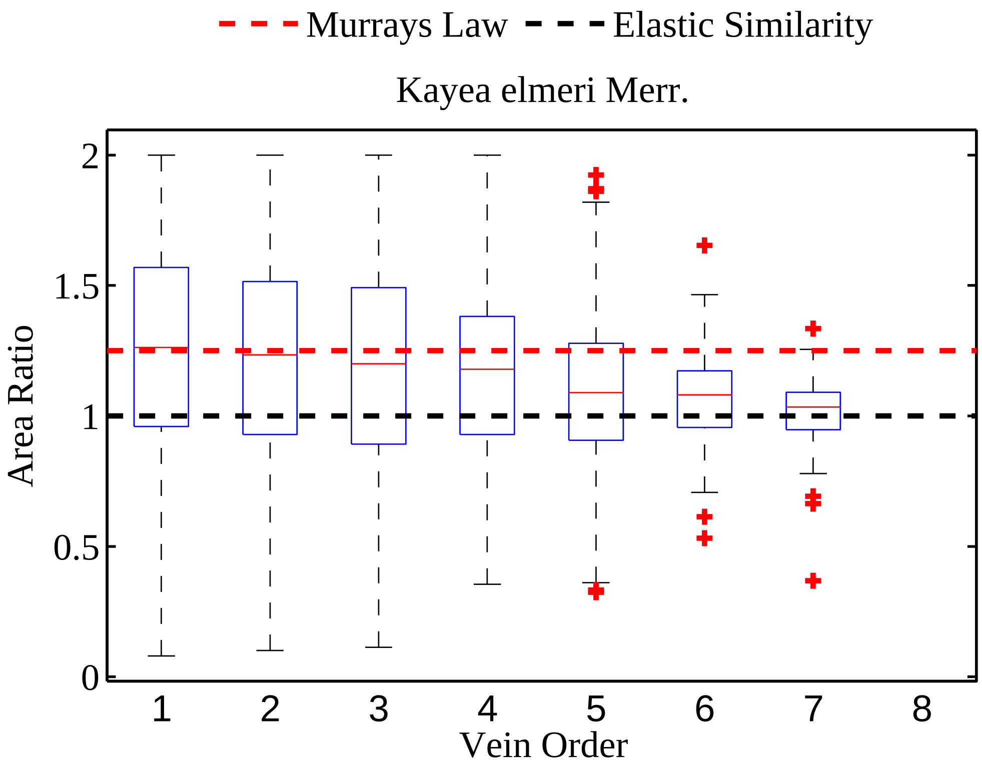

Murrays Law
Elastic Similarity
Kayea elmeri Merr.
Area Ratio
0
0.5
1
1.5
2
1
2
3
4
5
6
7
8
Vein Order

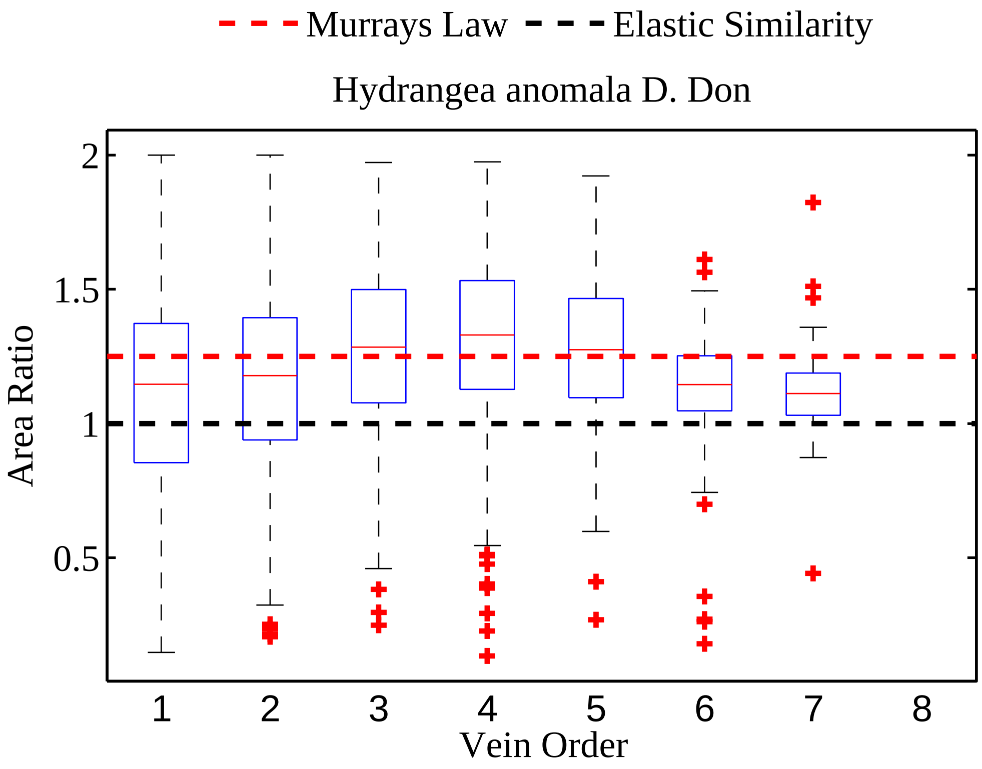

Murrays Law
Elastic Similarity
Hydrangea anomala D. Don
Area Ratio
2
1.5
1
0.5
1
2
3
4
5
6
7
8
Vein Order

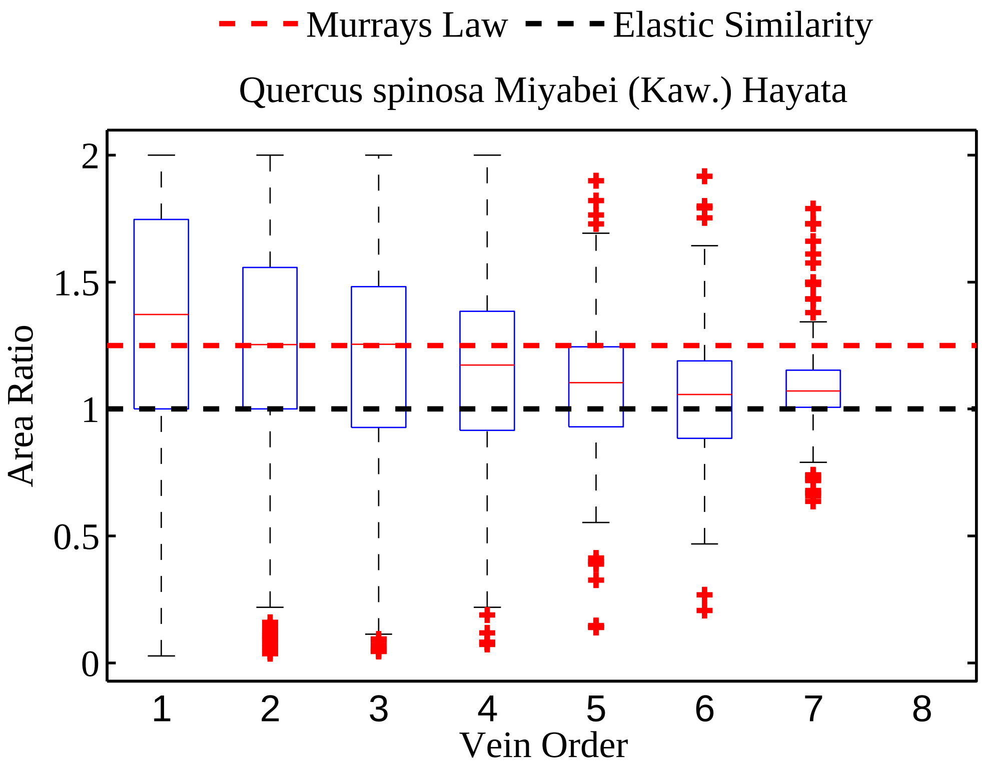

Murrays Law
Elastic Similarity
Quercus spinosa Miyabei (Kaw.) Hayata
Area Ratio
Vein Order
0
0.5
1
1.5
2
1
2
3
4
5
6
7
8

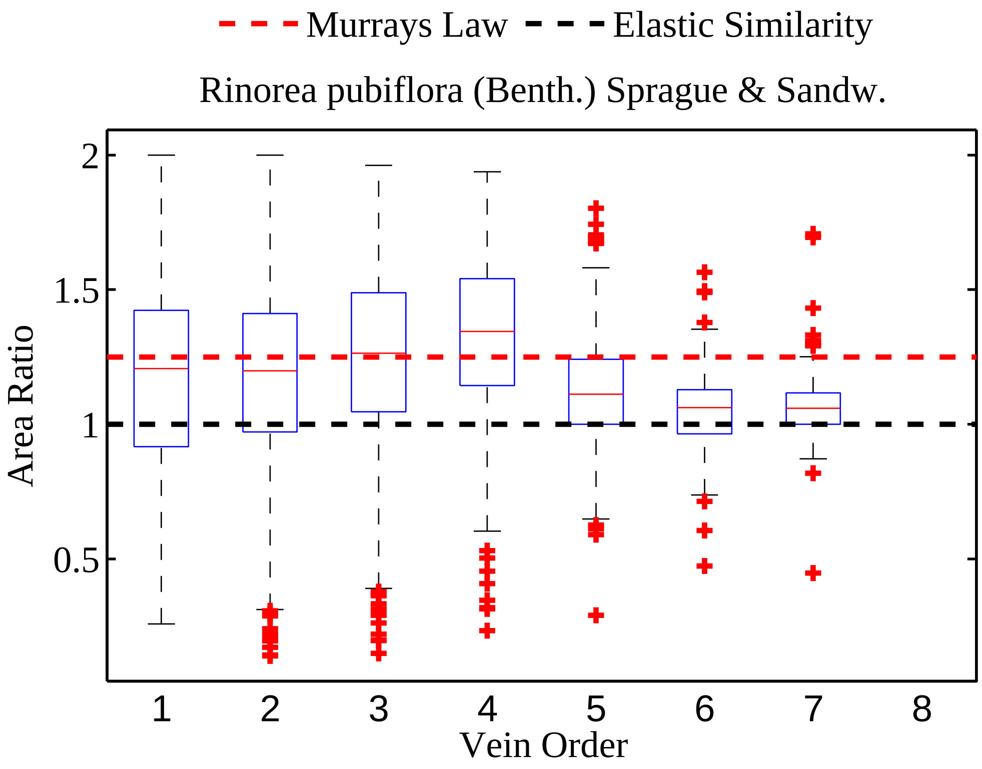

Murrays Law
Elastic Similarity
Rinorea pubiflora (Benth.) Sprague & Sandw.
Area Ratio
2
1.5
1
0.5
1
2
3
4
5
6
7
8
Vein Order

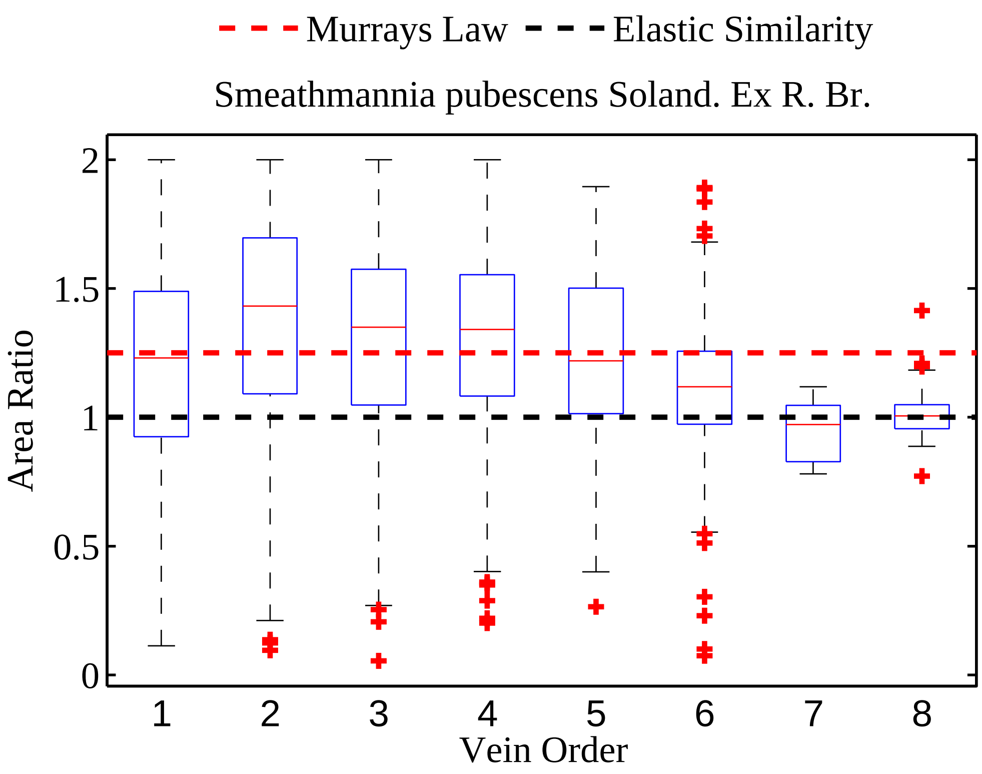

Murrays Law
Elastic Similarity
Smeathmannia pubescens Soland. Ex R. Br.
Area Ratio
Vein Order
0
0.5
1
1.5
2
1
2
3
4
5
6
7
8

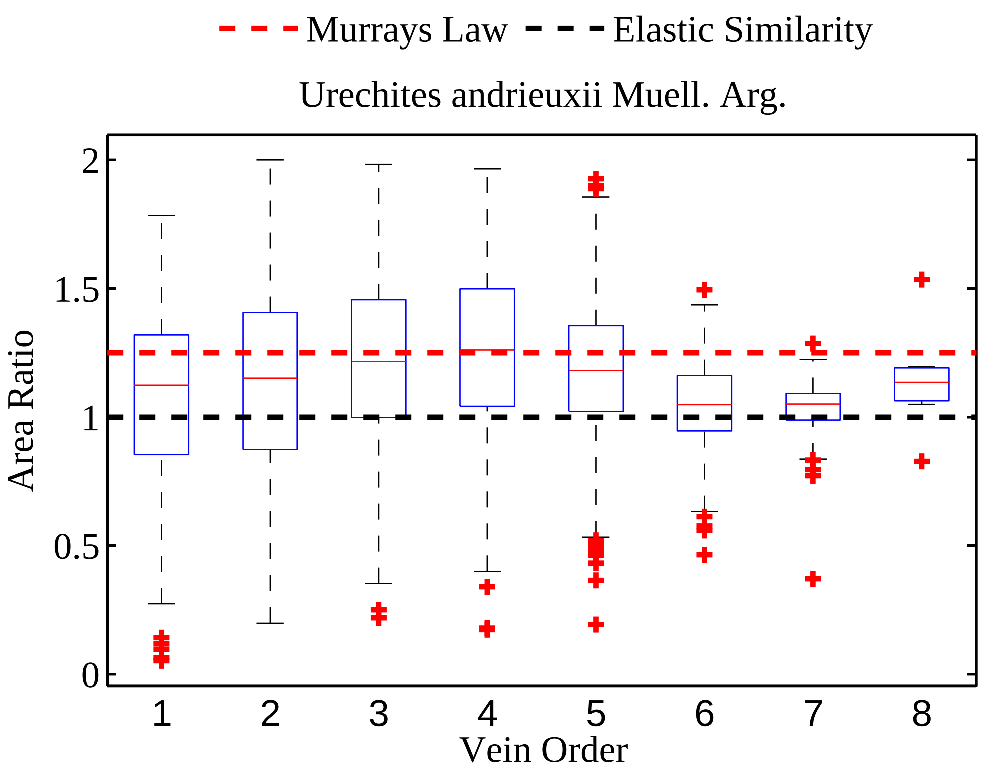

Murrays Law
Elastic Similarity
Urechites andrieuxii Muell. Arg.
Area Ratio
0
0.5
1
1.5
2
1
2
3
4
5
6
7
8
Vein Order

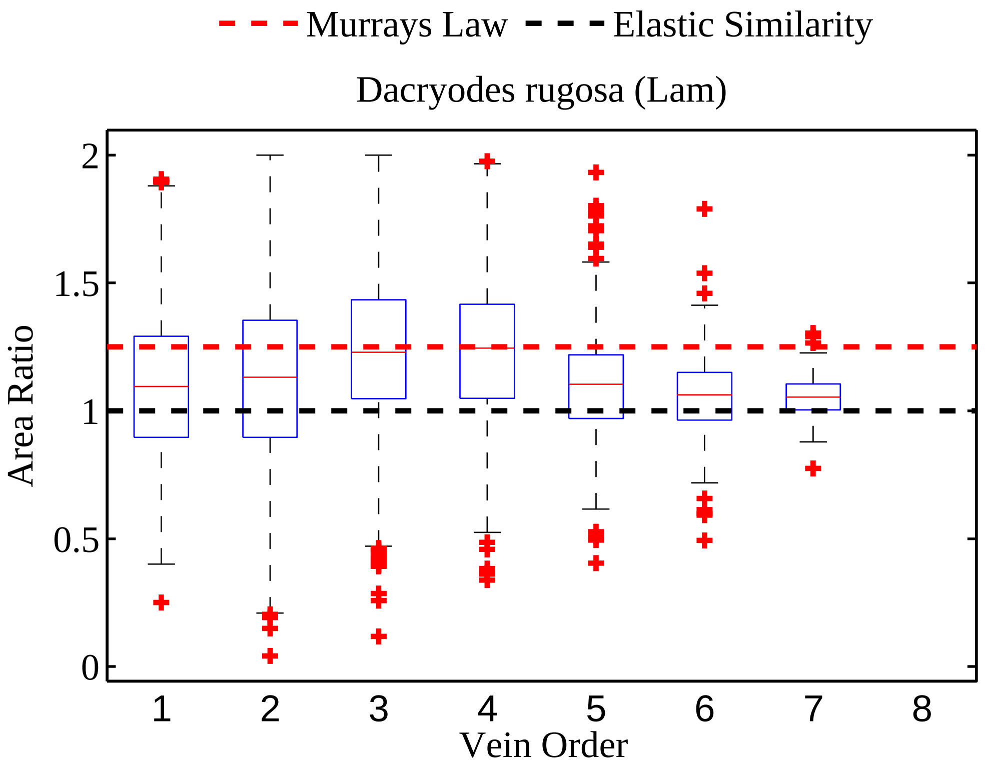
Murrays Law
Elastic Similarity
Dacryodes rugosa (Lam)
Area Ratio
0
0.5
1
1.5
2
1
2
3
4
5
6
7
8
Vein Order

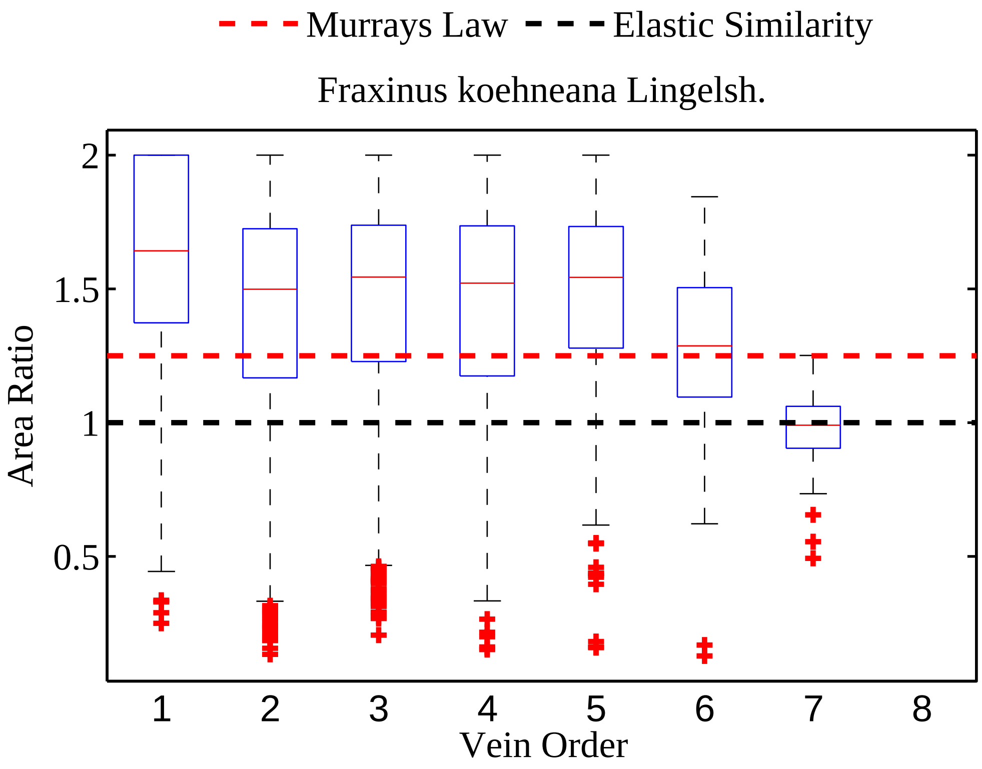

Murrays Law
Elastic Similarity
Fraxinus koehneana Lingelsh.
Area Ratio
2
1.5
1
0.5
1
2
3
4
5
6
7
8
Vein Order

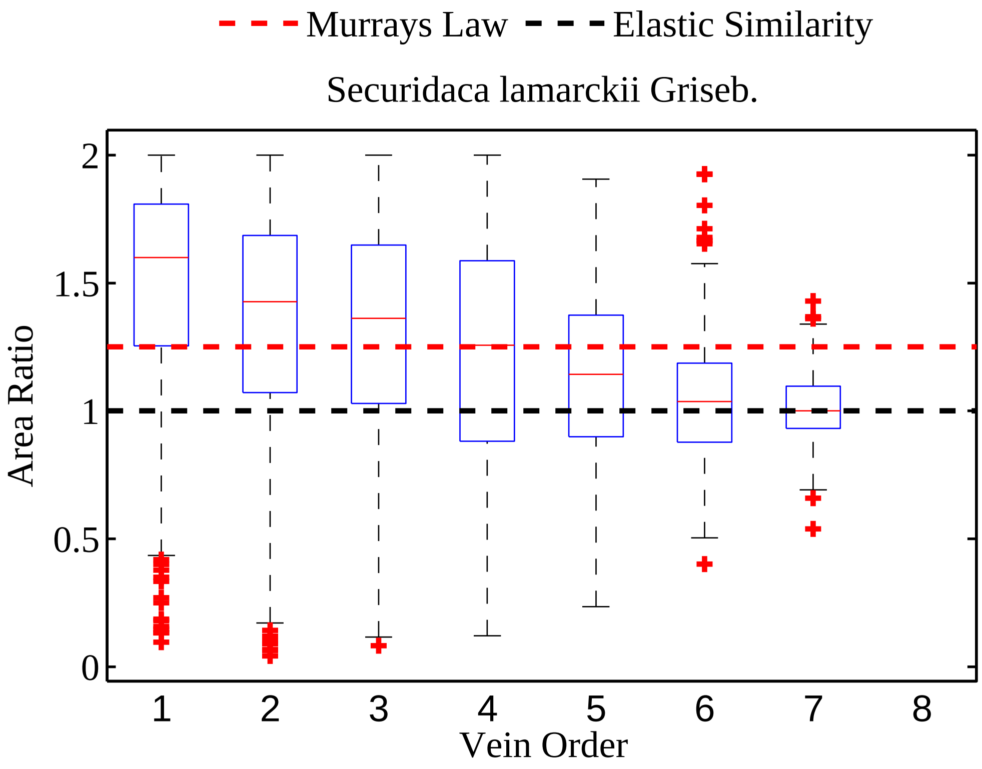

Murrays Law
Elastic Similarity
Securidaca lamarckii Griseb.
Area Ratio
2
1.5
1
0.5
0
1
2
3
4
5
6
7
8
Vein Order

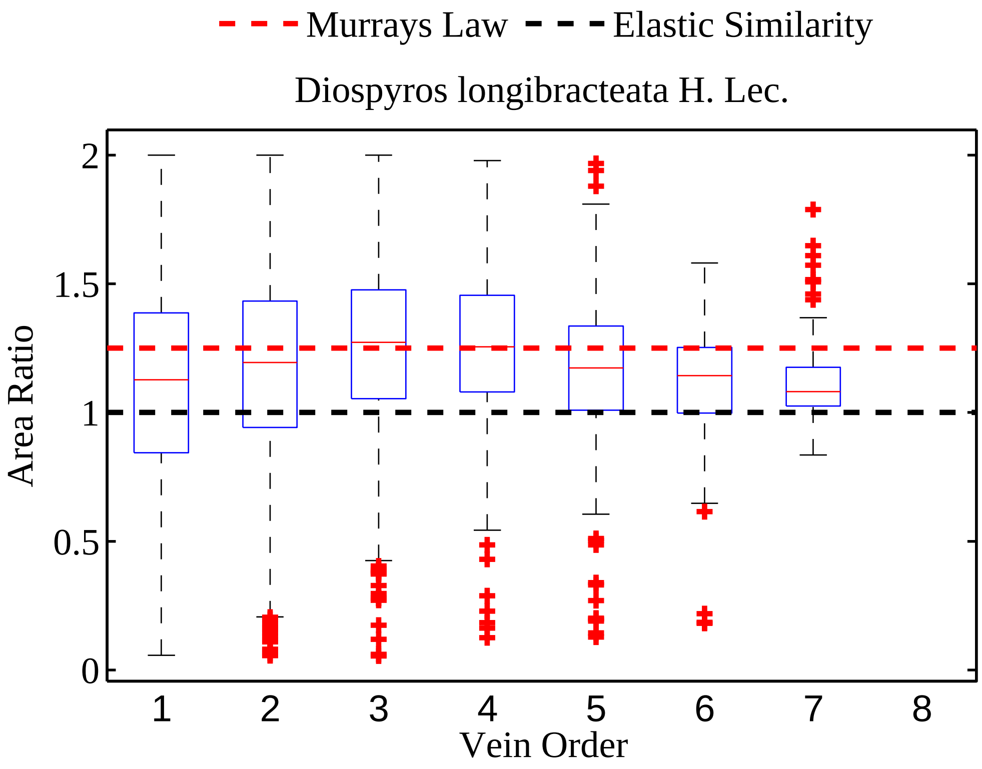

Murrays Law
Elastic Similarity
Diospyros longibracteata H. Lec.
Area Ratio
Vein Order
0
0.5
1
1.5
2
1
2
3
4
5
6
7
8

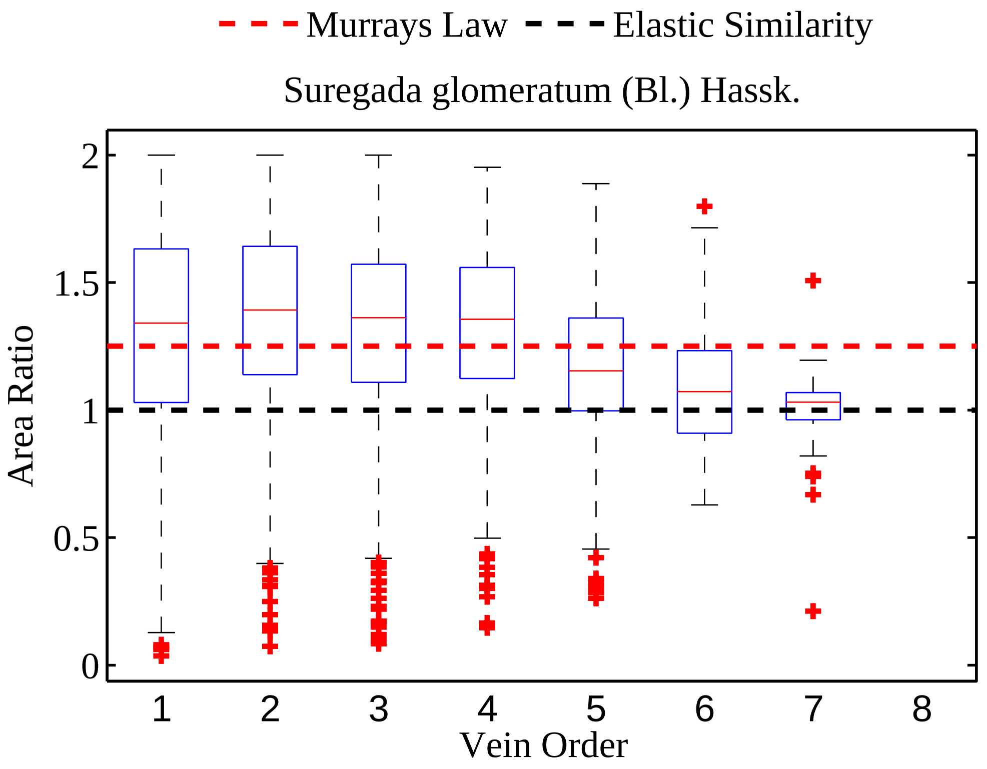
Murrays Law
Elastic Similarity
Suregada glomeratum (Bl.) Hassk.
Area Ratio
Vein Order
0
0.5
1
1.5
2
1
2
3
4
5
6
7
8

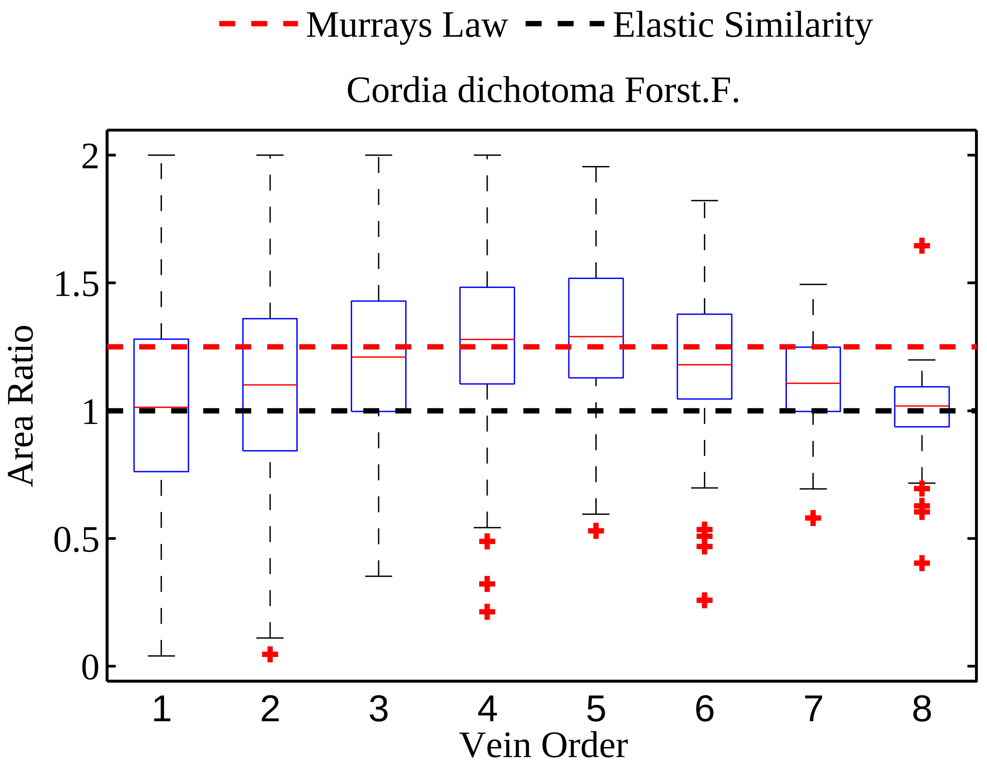
Murrays Law
Elastic Similarity
Cordia dichotoma Forst.F.
Area Ratio
Vein Order
0
0.5
1
1.5
2
1
2
3
4
5
6
7
8

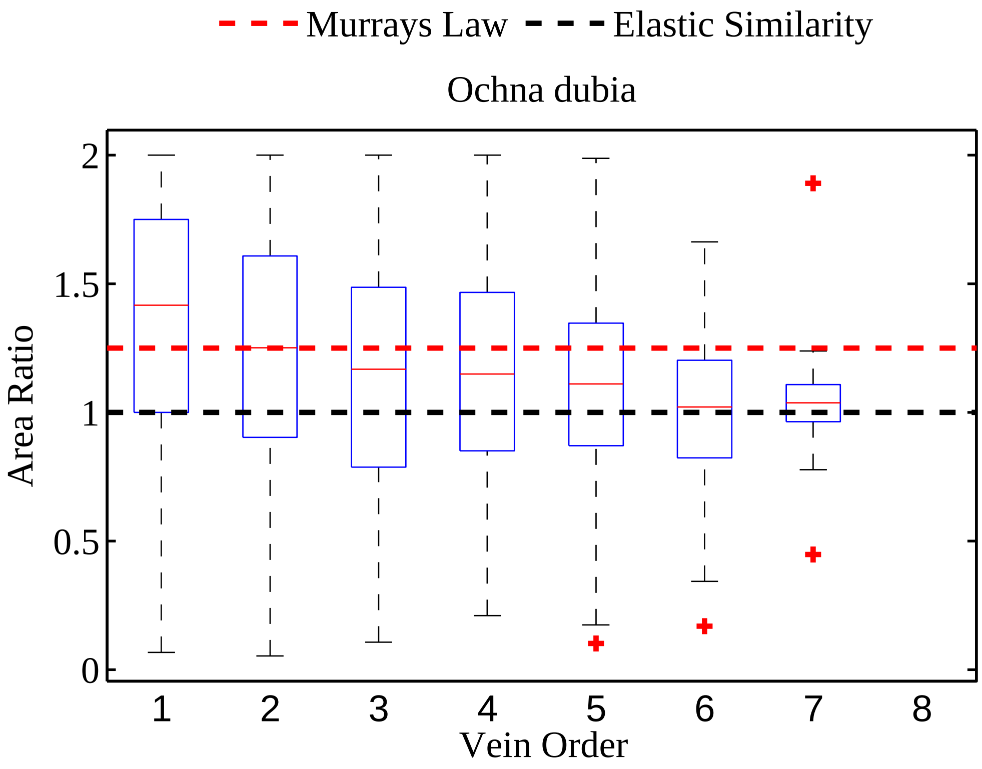

Murrays Law
Elastic Similarity
Ochna dubia
Area Ratio
2
1.5
1
0.5
0
1
2
3
4
5
6
7
8
Vein Order

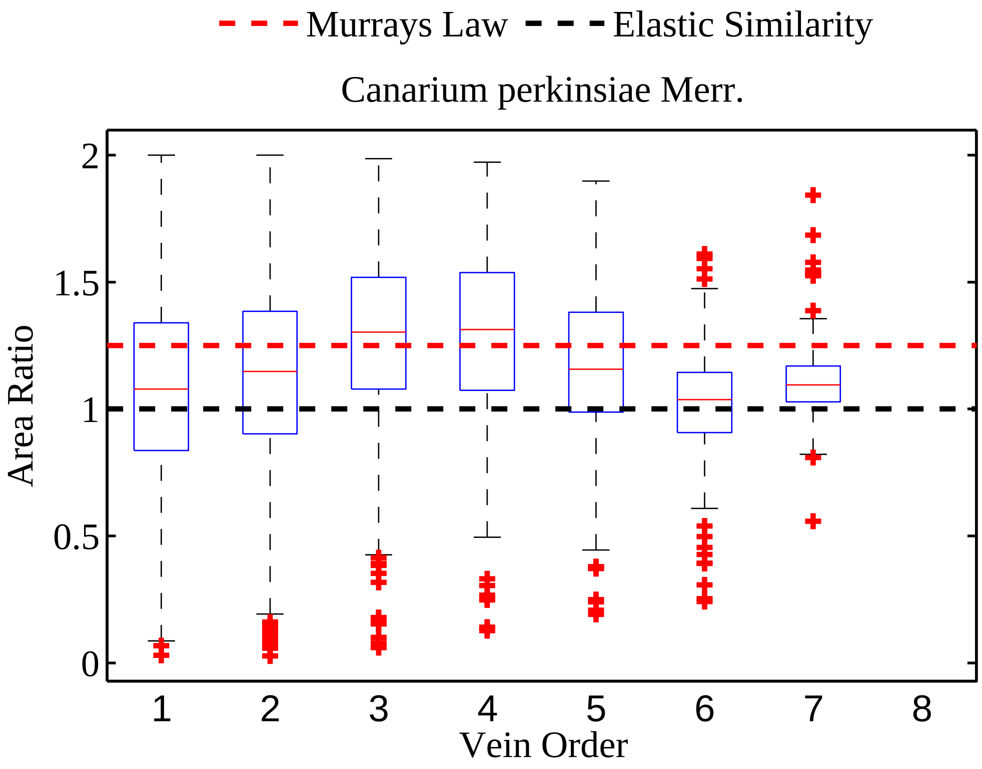

Murrays Law
Elastic Similarity
Canarium perkinsiae Merr.
Area Ratio
2
1.5
1
0.5
0
1
2
3
4
5
6
7
8
Vein Order

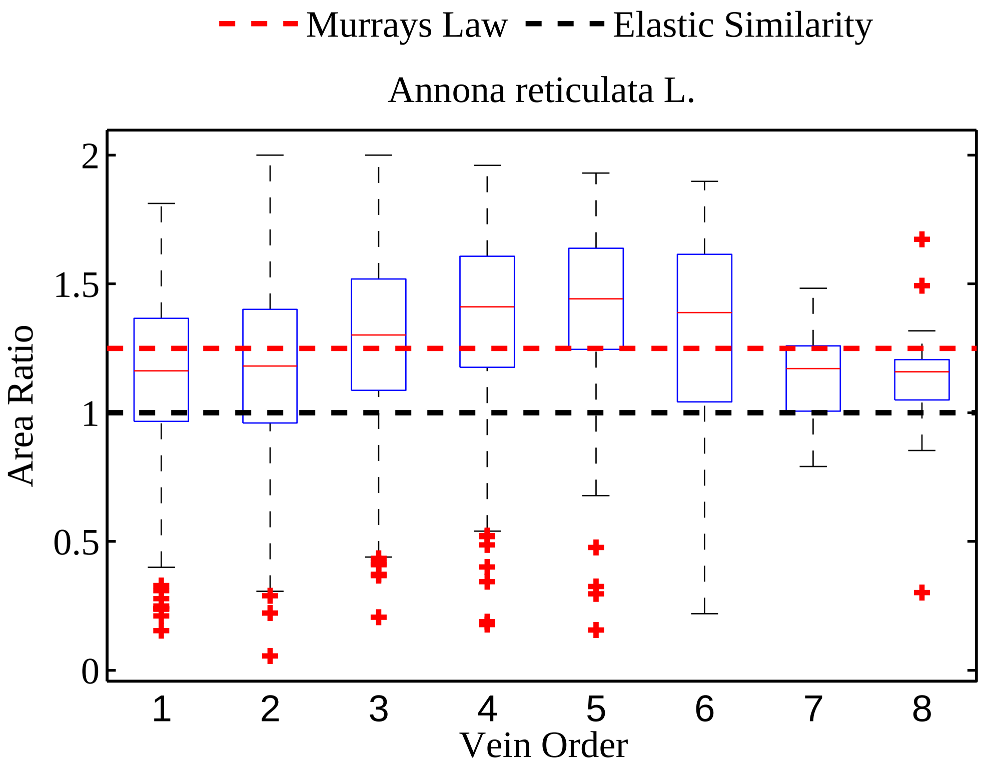

Murrays Law
Elastic Similarity
Annona reticulata L.
Area Ratio
0
0.5
1
1.5
2
1
2
3
4
5
6
7
8
Vein Order

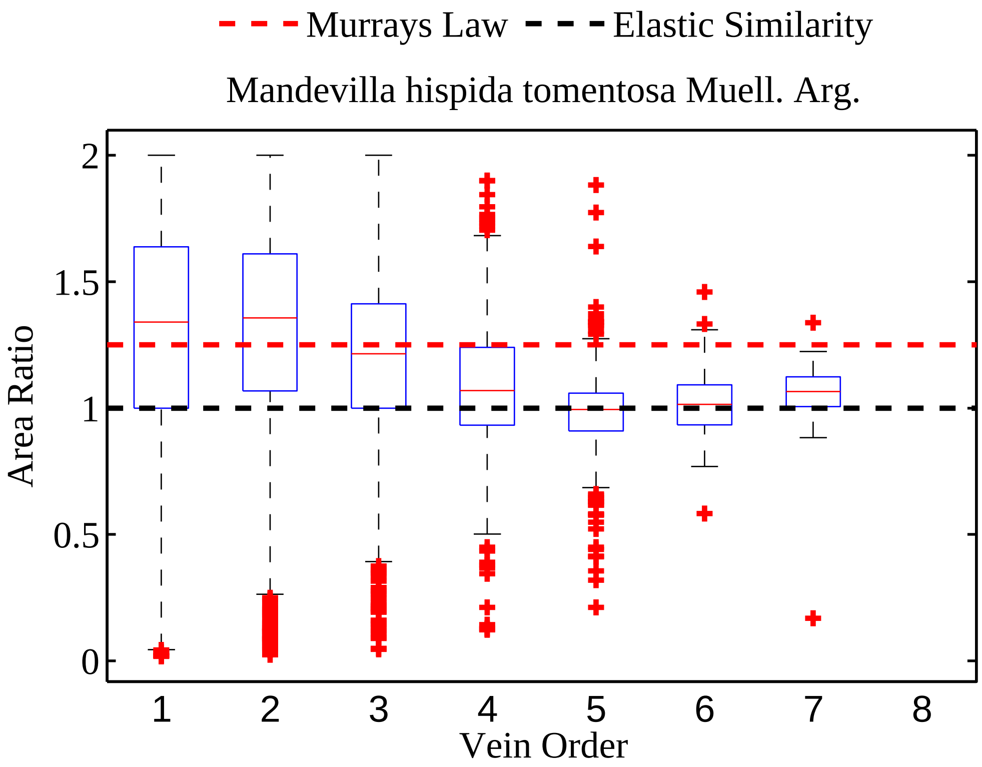

Murrays Law
Elastic Similarity
Mandevilla hispida tomentosa Muell. Arg.
Area Ratio
Vein Order
0
0.5
1
1.5
2
1
2
3
4
5
6
7
8

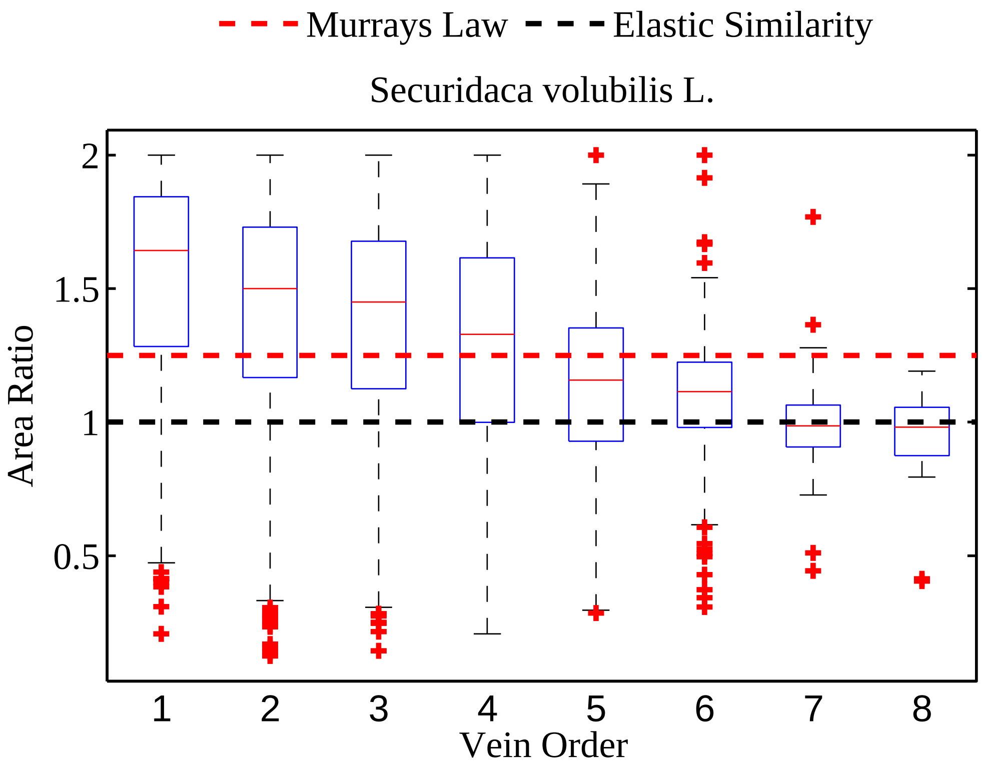

Murrays Law
Elastic Similarity
Securidaca volubilis L.
Area Ratio
2
1.5
1
0.5
1
2
3
4
5
6
7
8
Vein Order

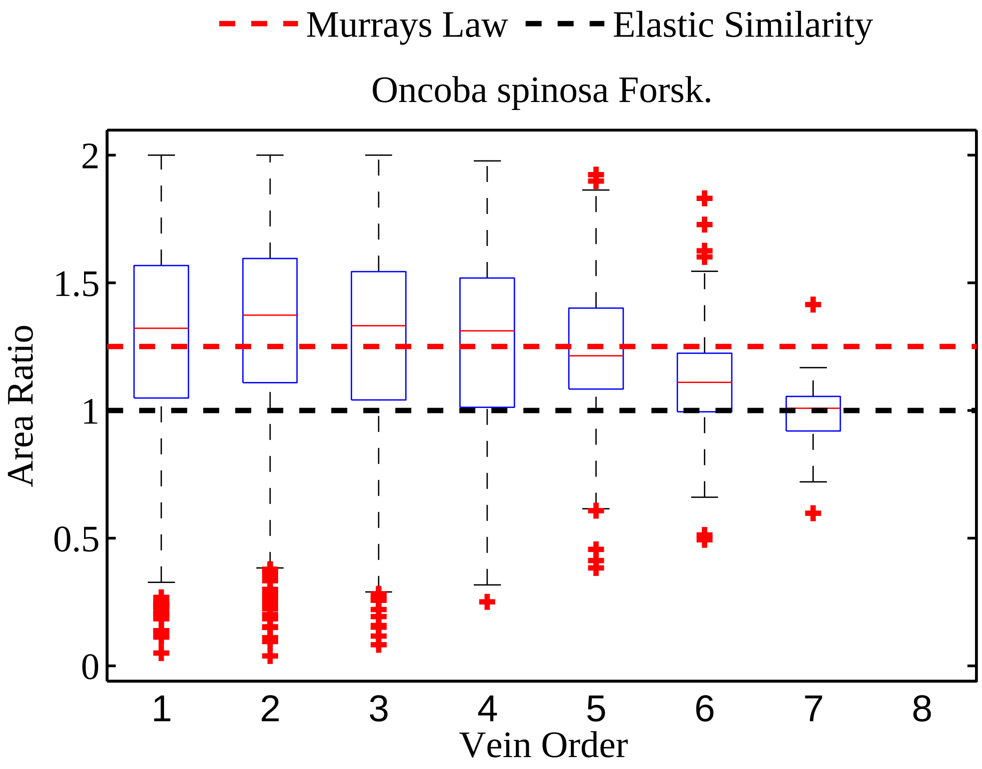
Murrays Law
Elastic Similarity
Oncoba spinosa Forsk.
Area Ratio
Vein Order
0
0.5
1
1.5
2
1
2
3
4
5
6
7
8

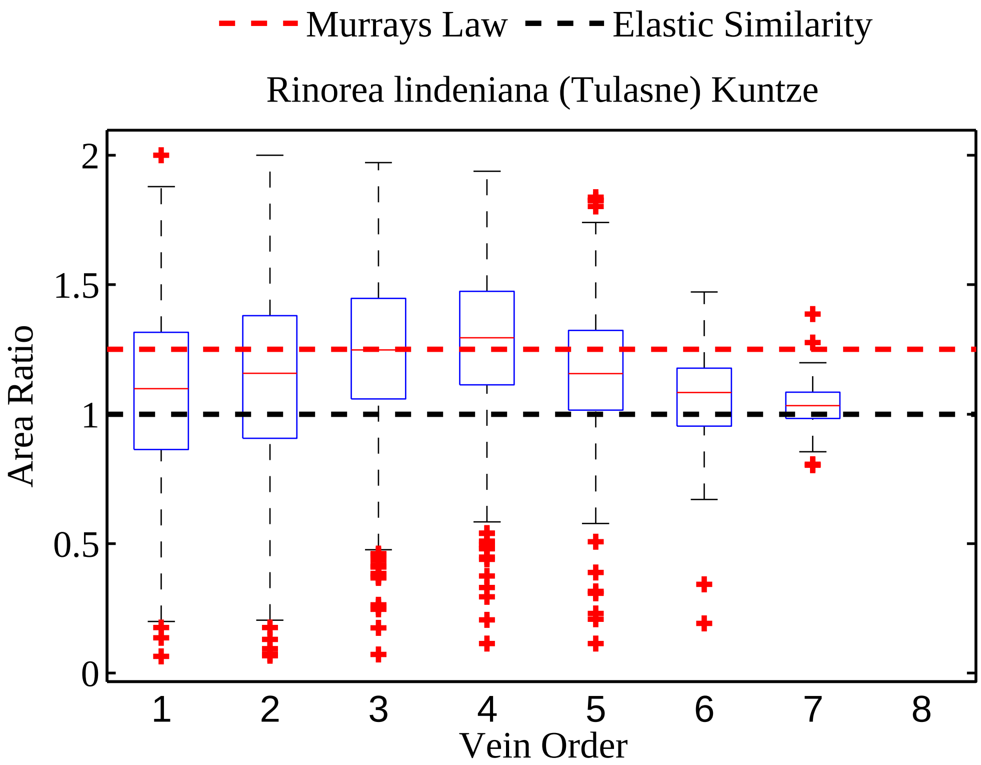

Murrays Law
Elastic Similarity
Rinorea lindeniana (Tulasne) Kuntze
Area Ratio
2
1.5
1
0.5
0
1
2
3
4
5
6
7
8
Vein Order

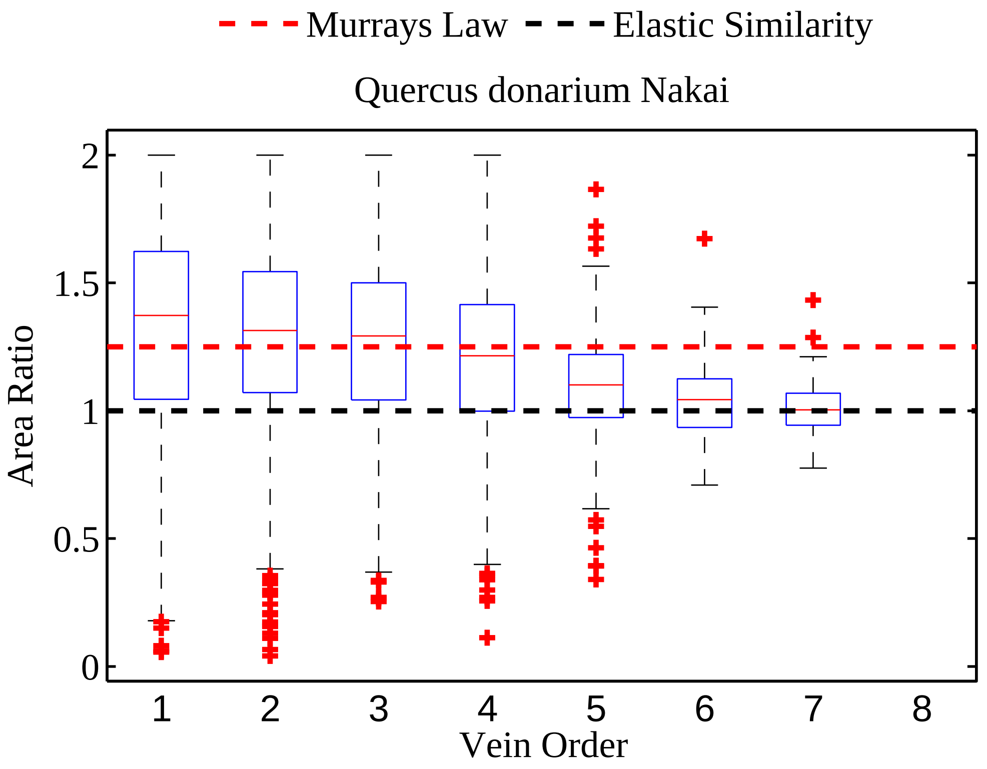

Murrays Law
Elastic Similarity
Quercus donarium Nakai
Area Ratio
Vein Order
0
0.5
1
1.5
2
1
2
3
4
5
6
7
8

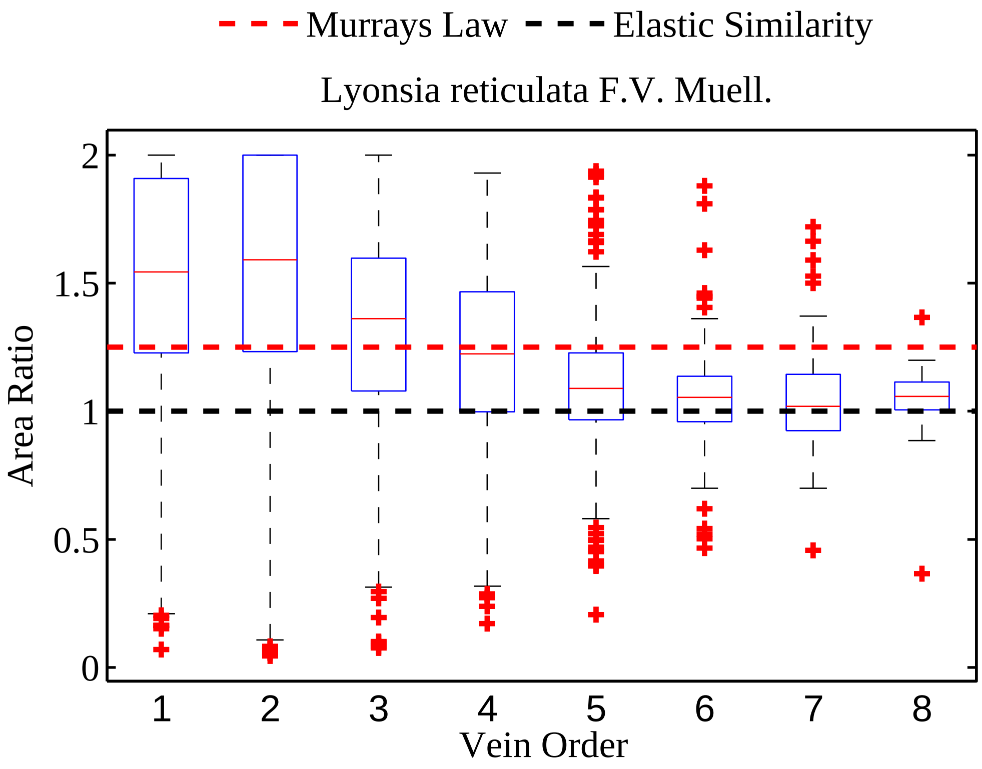

Murrays Law
Elastic Similarity
Lyonsia reticulata F.V. Muell.
Area Ratio
0
0.5
1
1.5
2
1
2
3
4
5
6
7
8
Vein Order

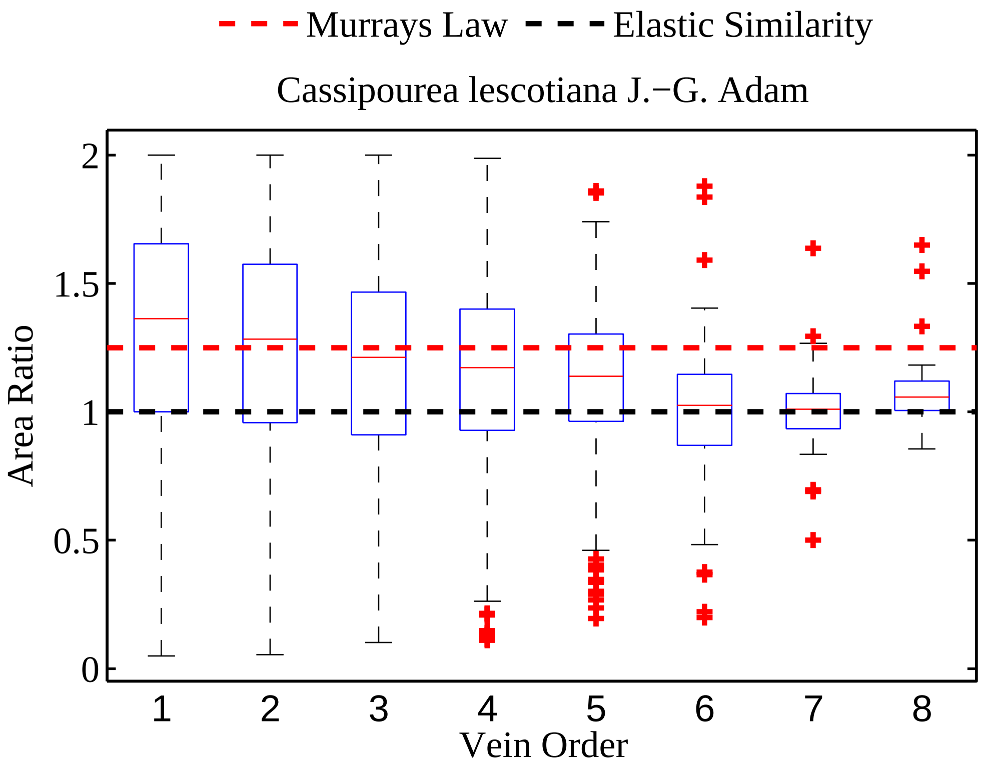

Murrays Law
Elastic Similarity
Cassipourea lescotiana J.−G. Adam
Area Ratio
0
0.5
1
1.5
2
1
2
3
4
5
6
7
8
Vein Order

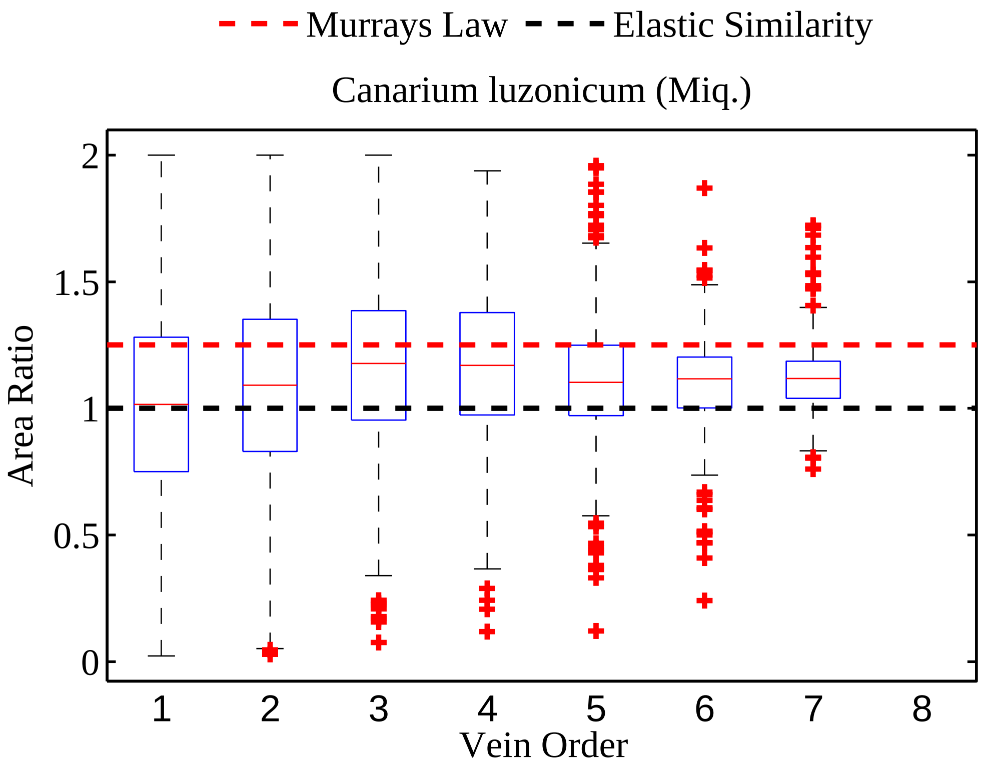
Murrays Law
Elastic Similarity
Canarium luzonicum (Miq.)
Area Ratio
0
0.5
1
1.5
2
1
2
3
4
5
6
7
8
Vein Order

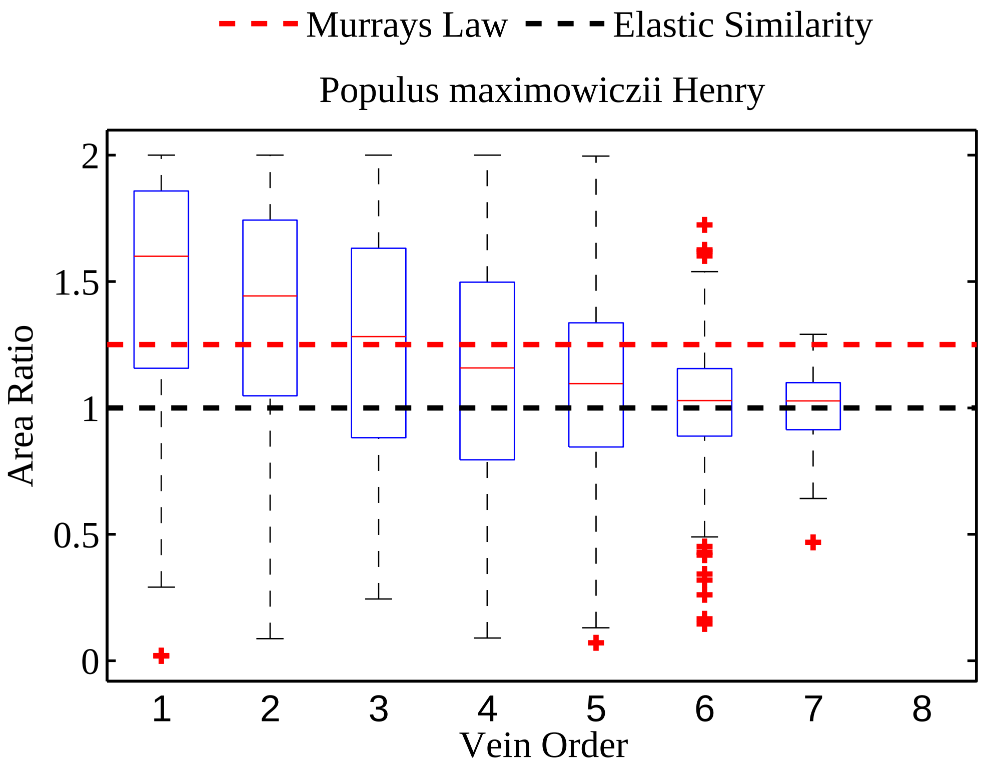
Murrays Law
Elastic Similarity
Populus maximowiczii Henry
Area Ratio
0
0.5
1
1.5
2
1
2
3
4
5
6
7
8
Vein Order

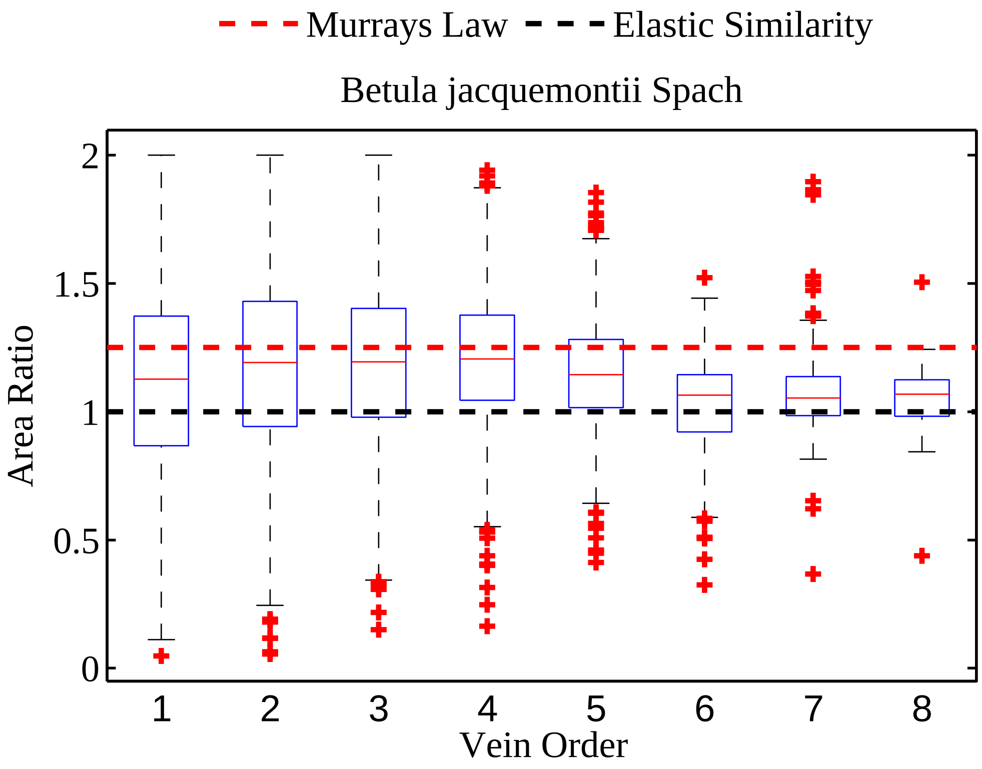

Murrays Law
Elastic Similarity
Betula jacquemontii Spach
Area Ratio
0
0.5
1
1.5
2
1
2
3
4
5
6
7
8
Vein Order

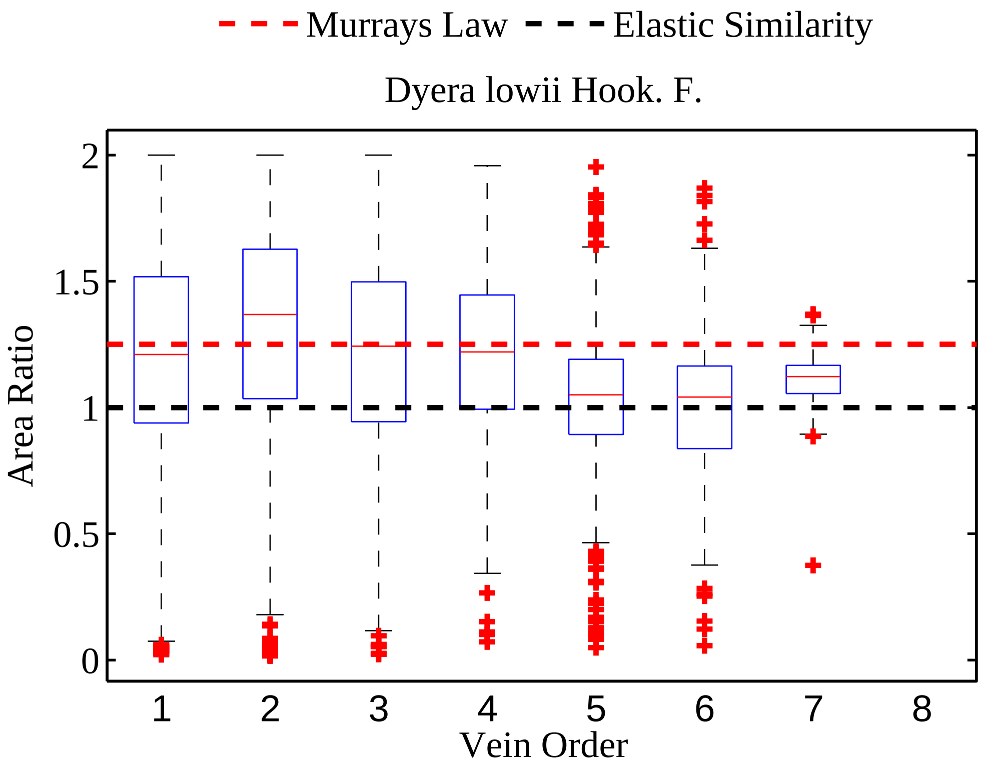

Murrays Law
Elastic Similarity
Dyera lowii Hook. F.
Area Ratio
Vein Order
0
0.5
1
1.5
2
1
2
3
4
5
6
7
8

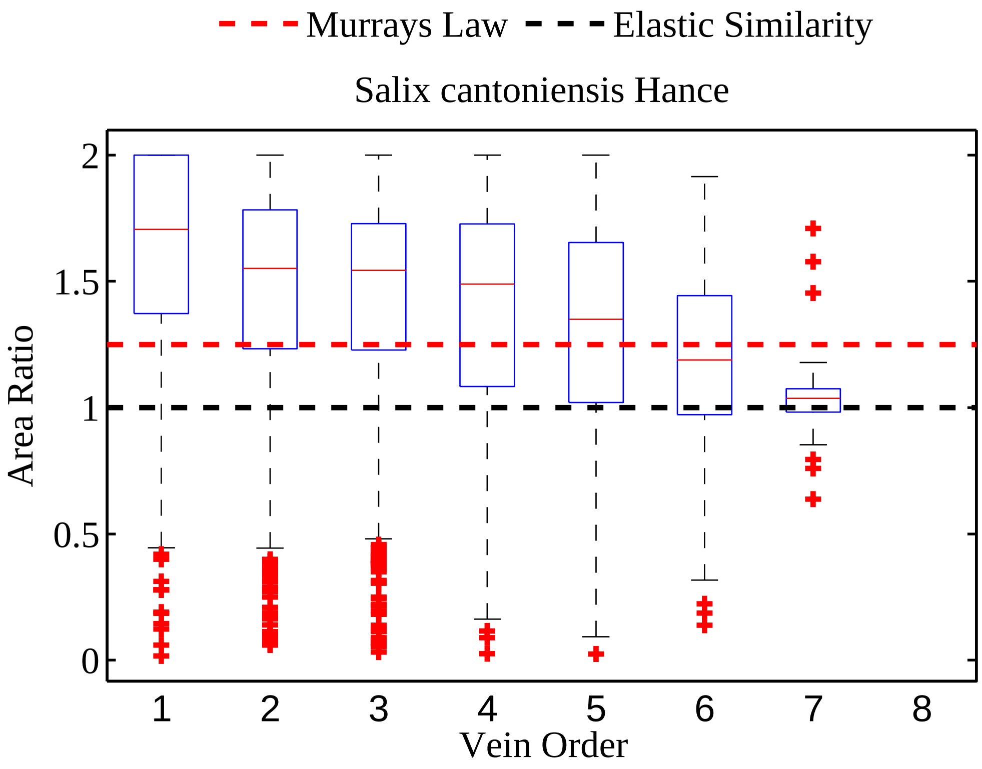

Murrays Law
Elastic Similarity
Salix cantoniensis Hance
Area Ratio
2
1.5
1
0.5
0
1
2
3
4
5
6
7
8
Vein Order

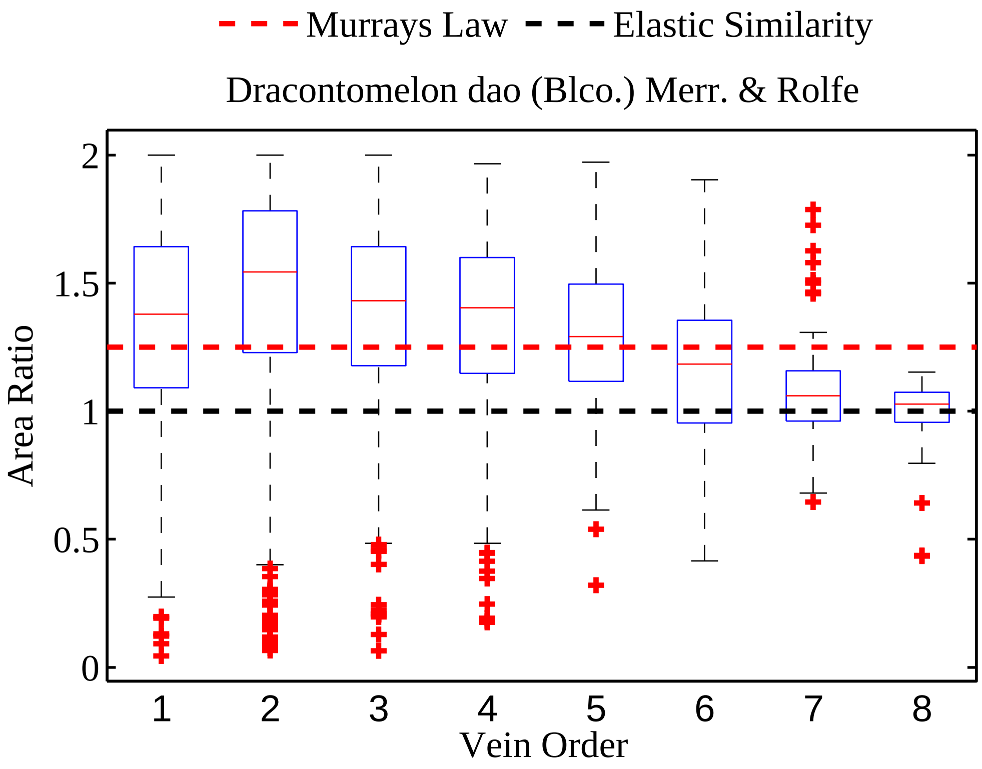

Murrays Law
Elastic Similarity
Dracontomelon dao (Blco.) Merr. & Rolfe
Area Ratio
0
0.5
1
1.5
2
1
2
3
4
5
6
7
8
Vein Order

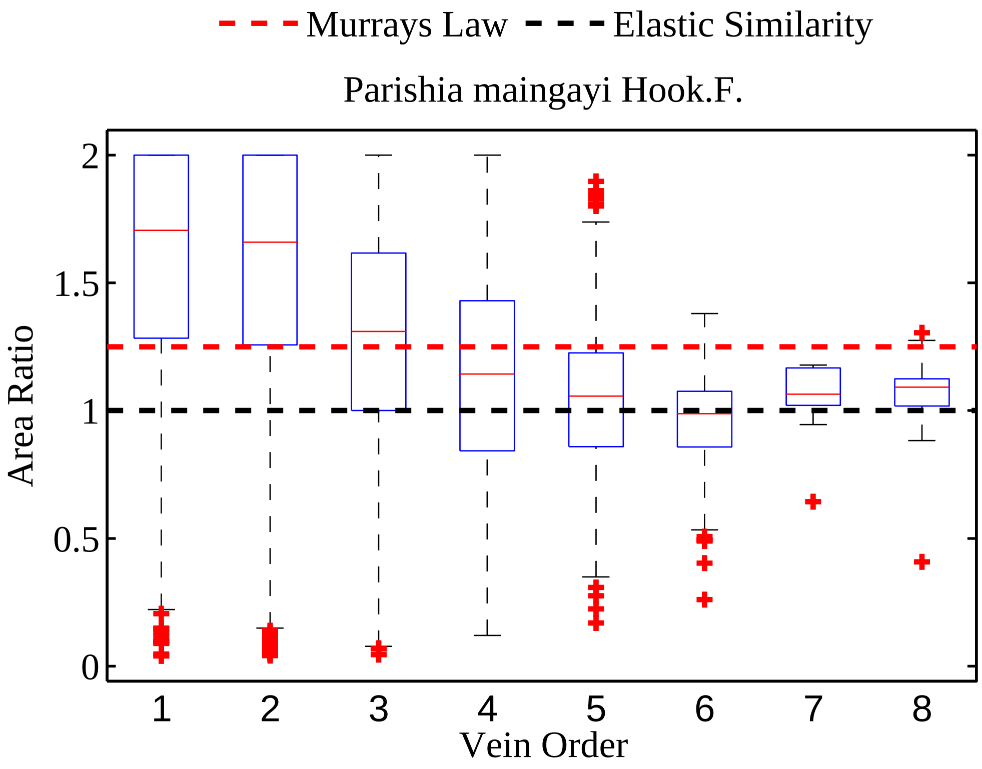

Murrays Law
Elastic Similarity
Parishia maingayi Hook.F.
Area Ratio
0
0.5
1
1.5
2
1
2
3
4
5
6
7
8
Vein Order

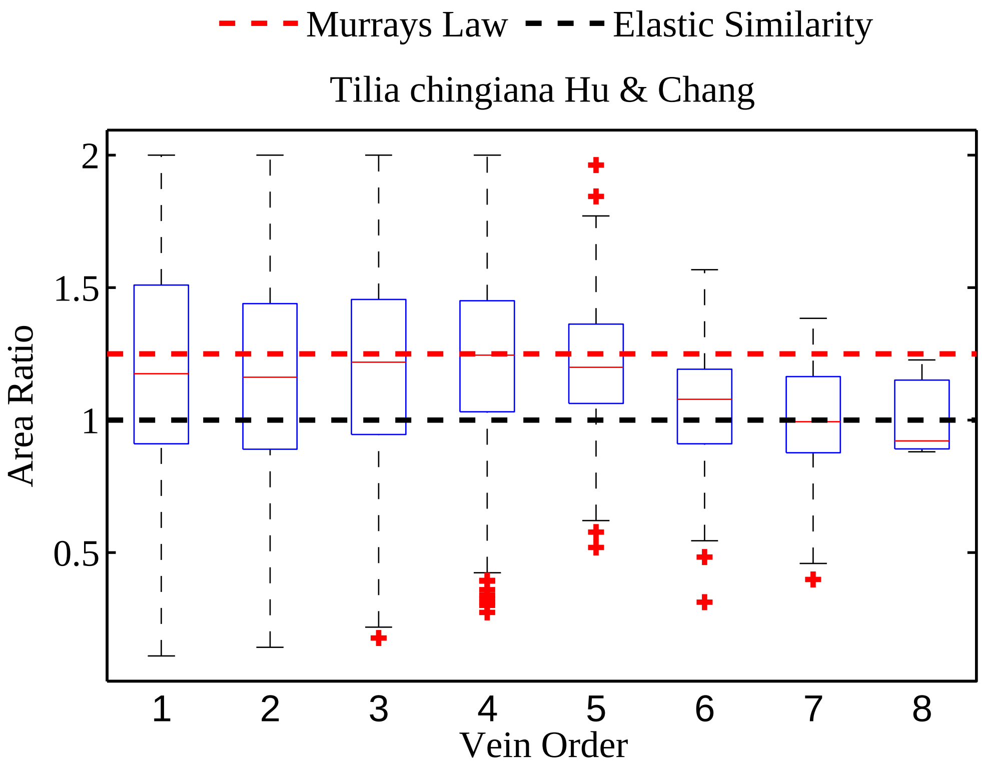

Murrays Law
Elastic Similarity
Tilia chingiana Hu & Chang
Area Ratio
2
1.5
1
0.5
1
2
3
4
5
6
7
8
Vein Order

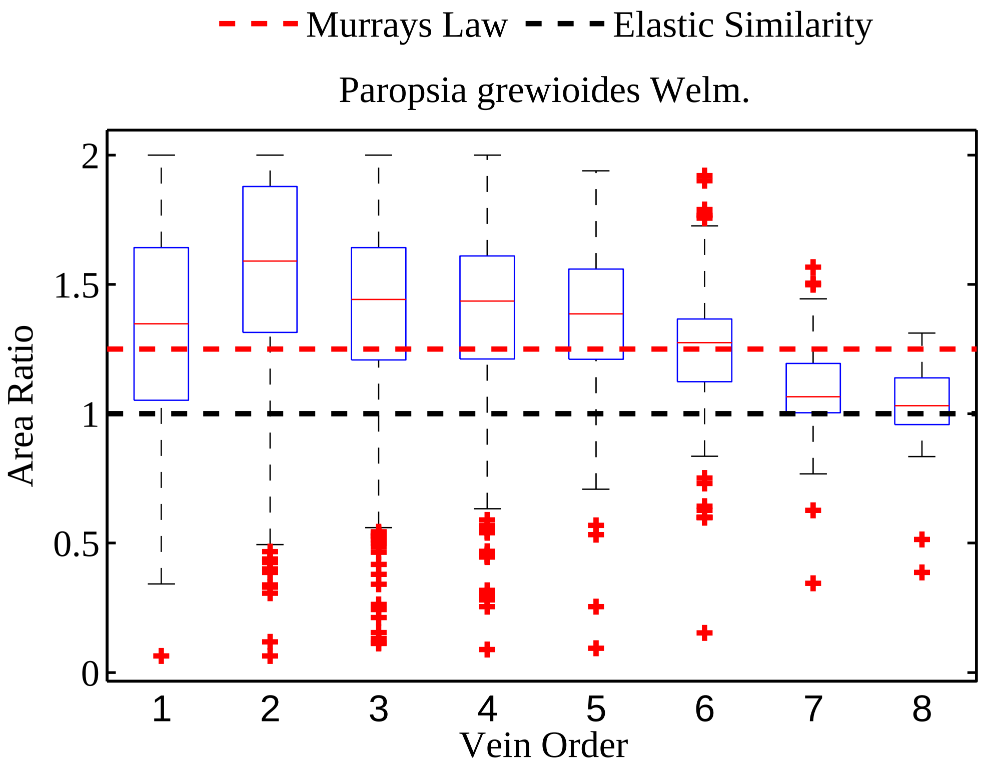

Murrays Law
Elastic Similarity
Paropsia grewioides Welm.
Area Ratio
0
0.5
1
1.5
2
1
2
3
4
5
6
7
8
Vein Order

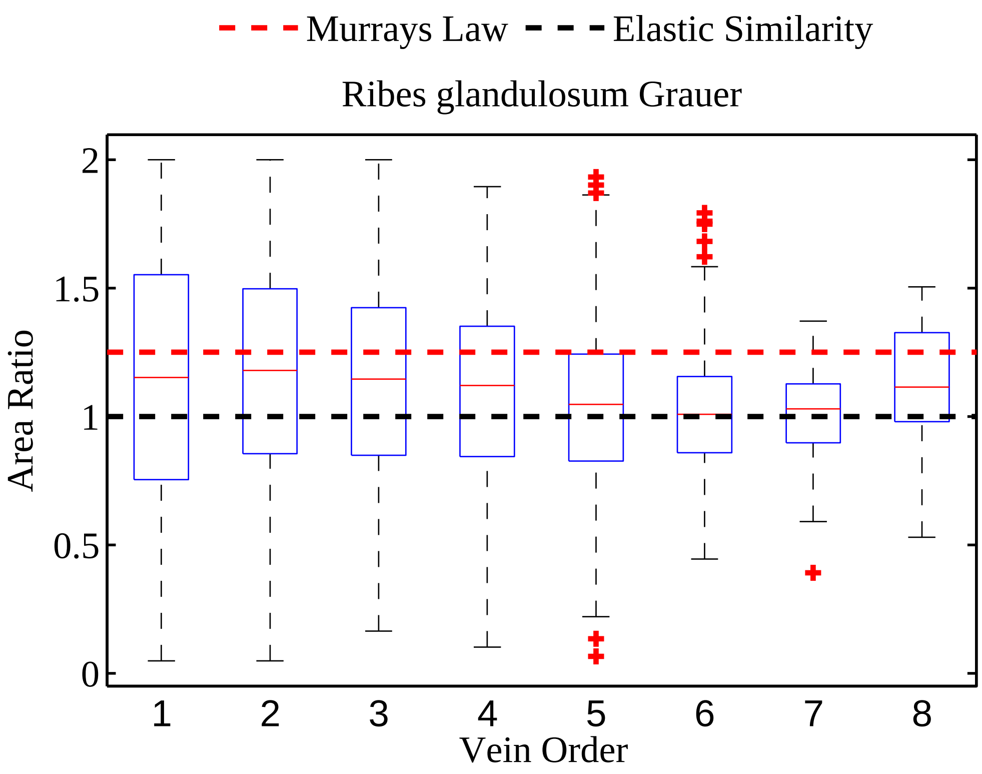

Murrays Law
Elastic Similarity
Ribes glandulosum Grauer
Area Ratio
Vein Order
2
1.5
1
0.5
0
1
2
3
4
5
6
7
8

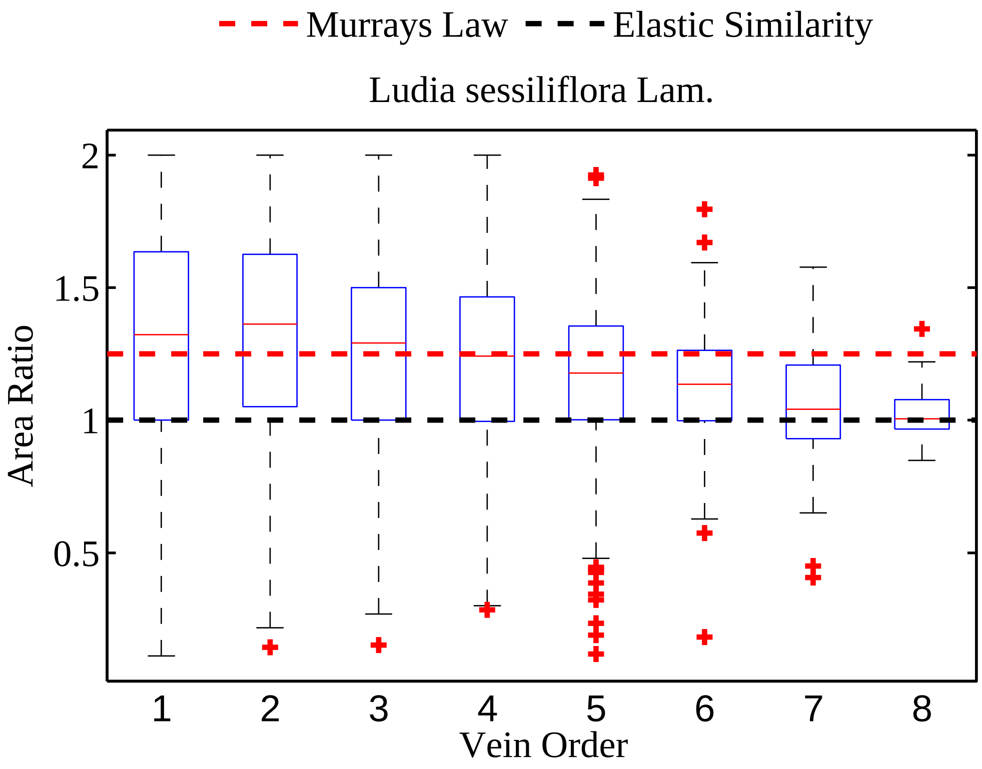

Murrays Law
Elastic Similarity
Ludia sessiliflora Lam.
Area Ratio
2
1.5
1
0.5
1
2
3
4
5
6
7
8
Vein Order

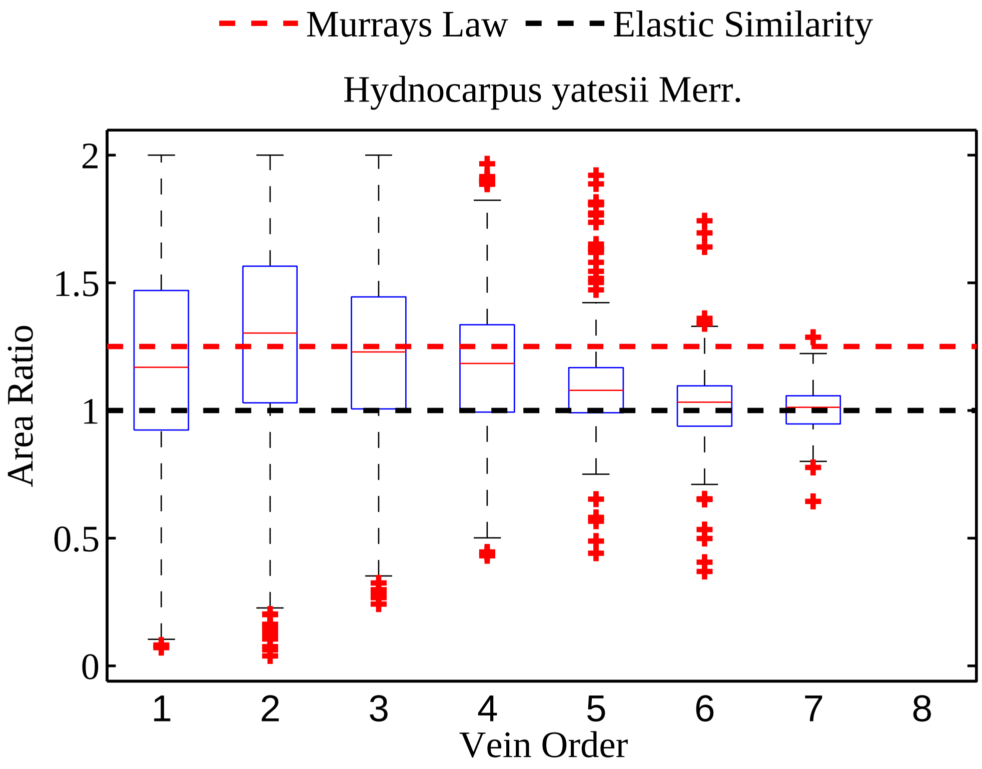

Murrays Law
Elastic Similarity
Hydnocarpus yatesii Merr.
Area Ratio
Vein Order
0
0.5
1
1.5
2
1
2
3
4
5
6
7
8

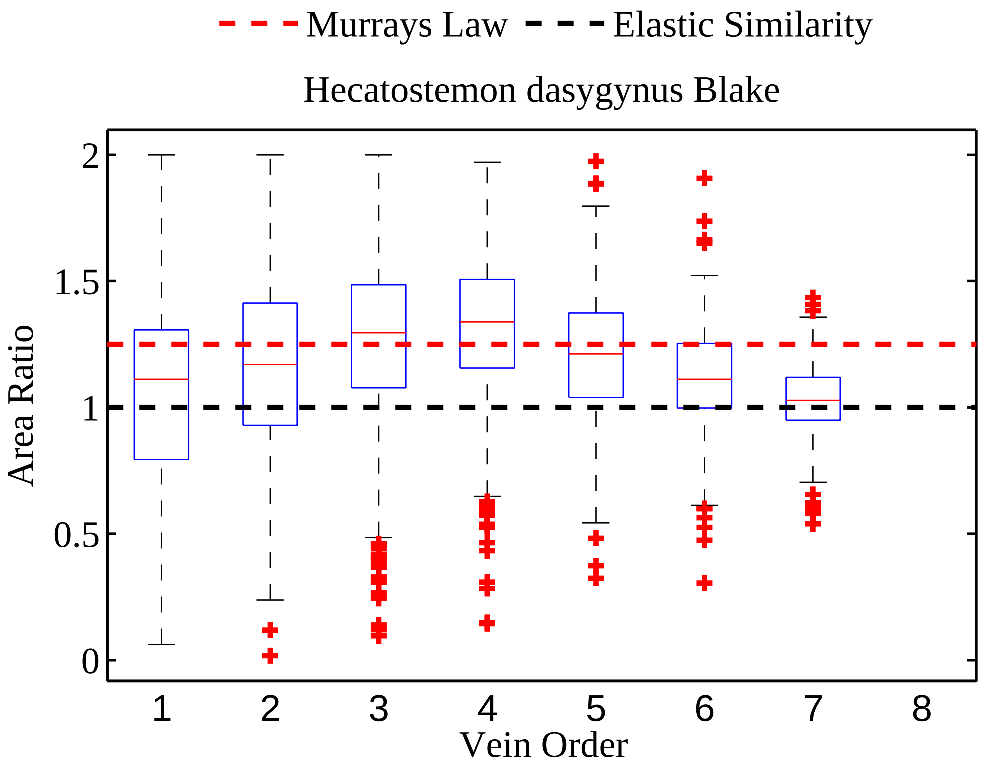

Murrays Law
Elastic Similarity
Hecatostemon dasygynus Blake
Area Ratio
Vein Order
0
0.5
1
1.5
2
1
2
3
4
5
6
7
8

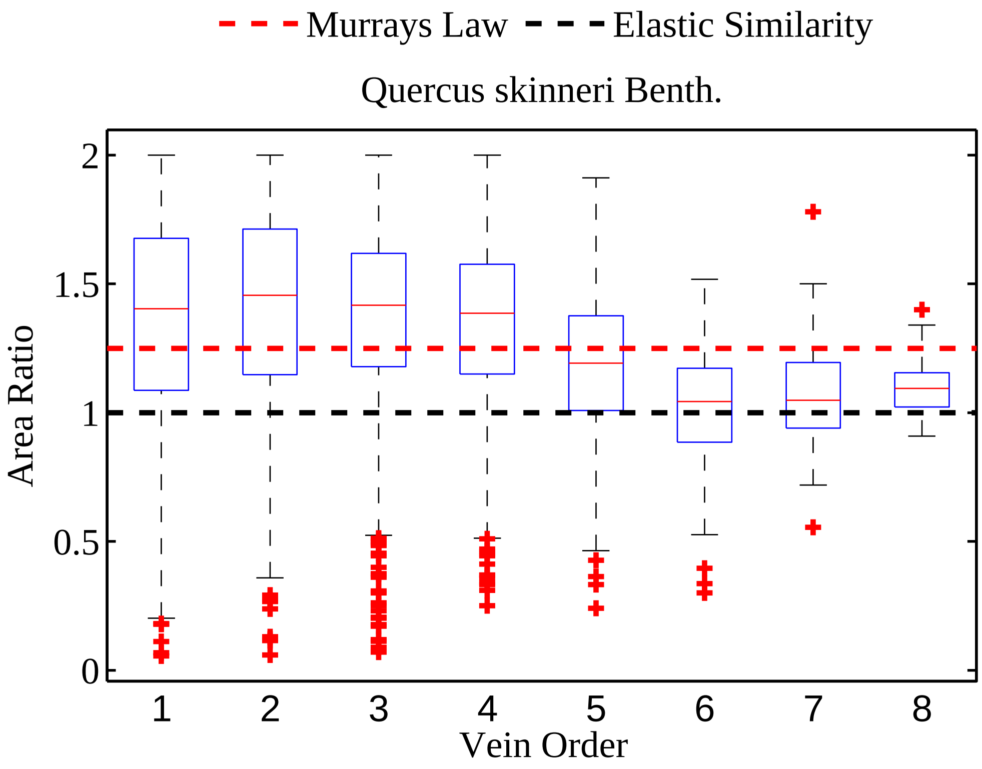
Murrays Law
Elastic Similarity
Quercus skinneri Benth.
Area Ratio
Vein Order
0
0.5
1
1.5
2
1
2
3
4
5
6
7
8

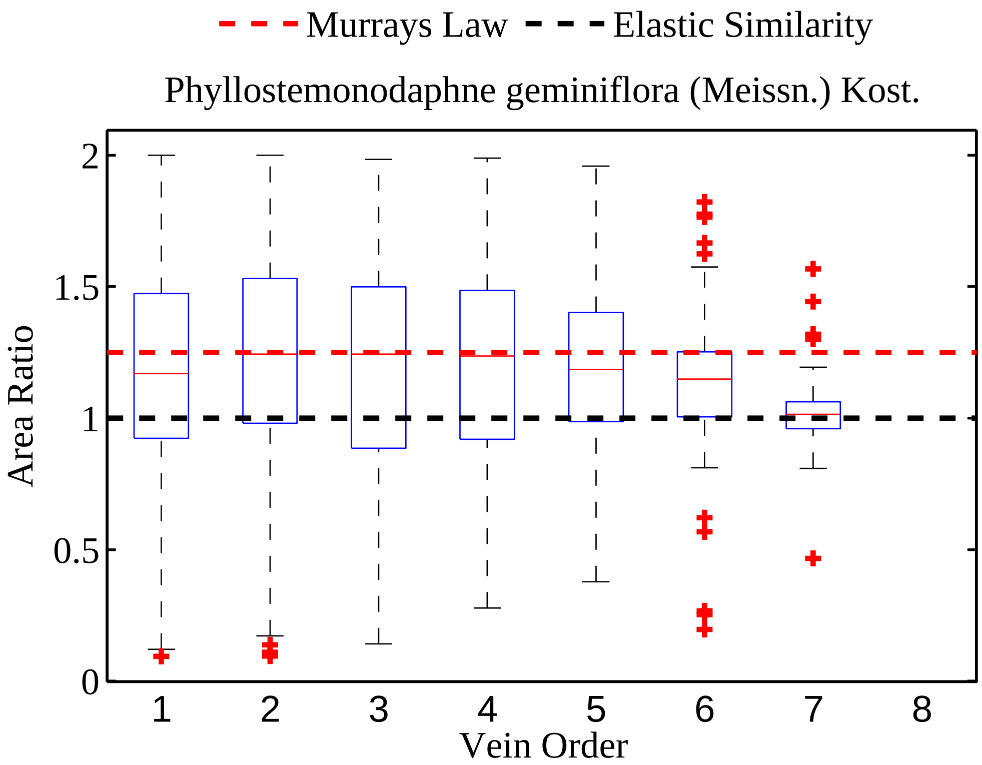

Murrays Law
Elastic Similarity
Phyllostemonodaphne geminiflora (Meissn.) Kost.
Area Ratio
0
0.5
1
1.5
2
1
2
3
4
5
6
7
8
Vein Order

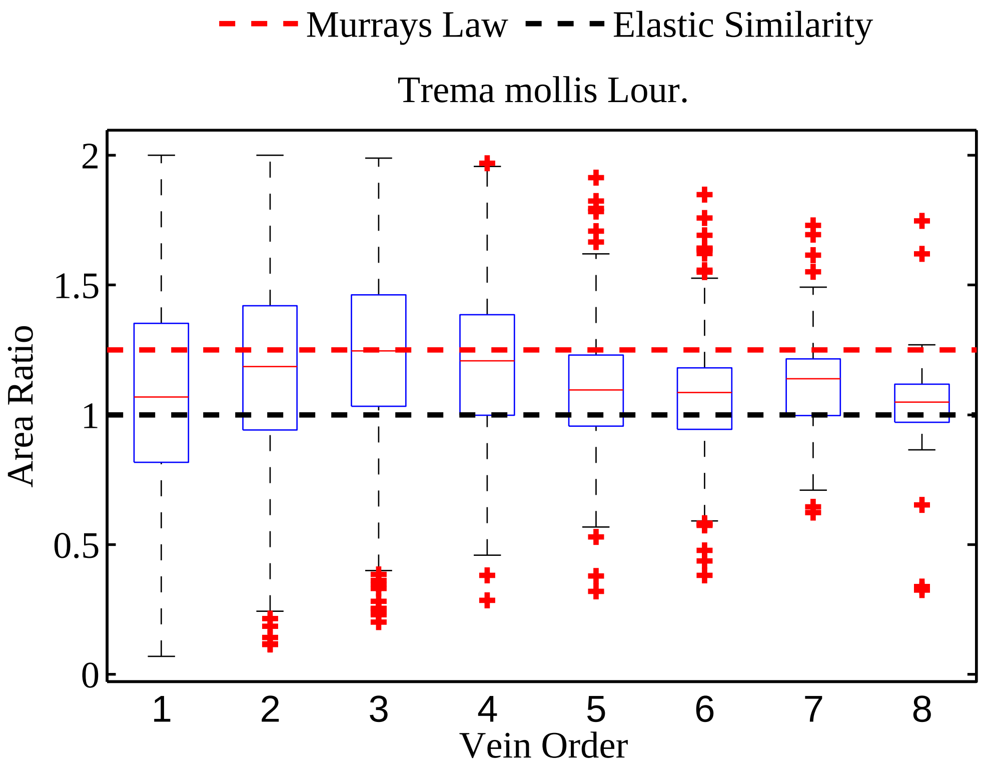

Murrays Law
Elastic Similarity
Trema mollis Lour.
Area Ratio
2
1.5
1
0.5
0
1
2
3
4
5
6
7
8
Vein Order

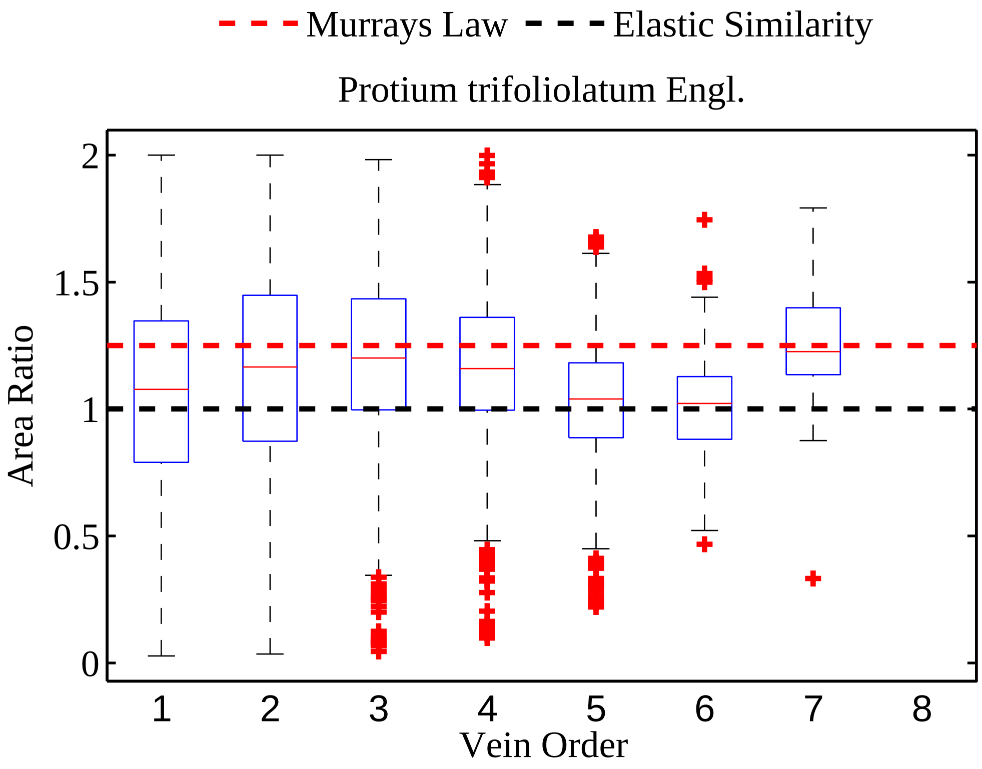

Murrays Law
Elastic Similarity
Protium trifoliolatum Engl.
Area Ratio
0
0.5
1
1.5
2
1
2
3
4
5
6
7
8
Vein Order

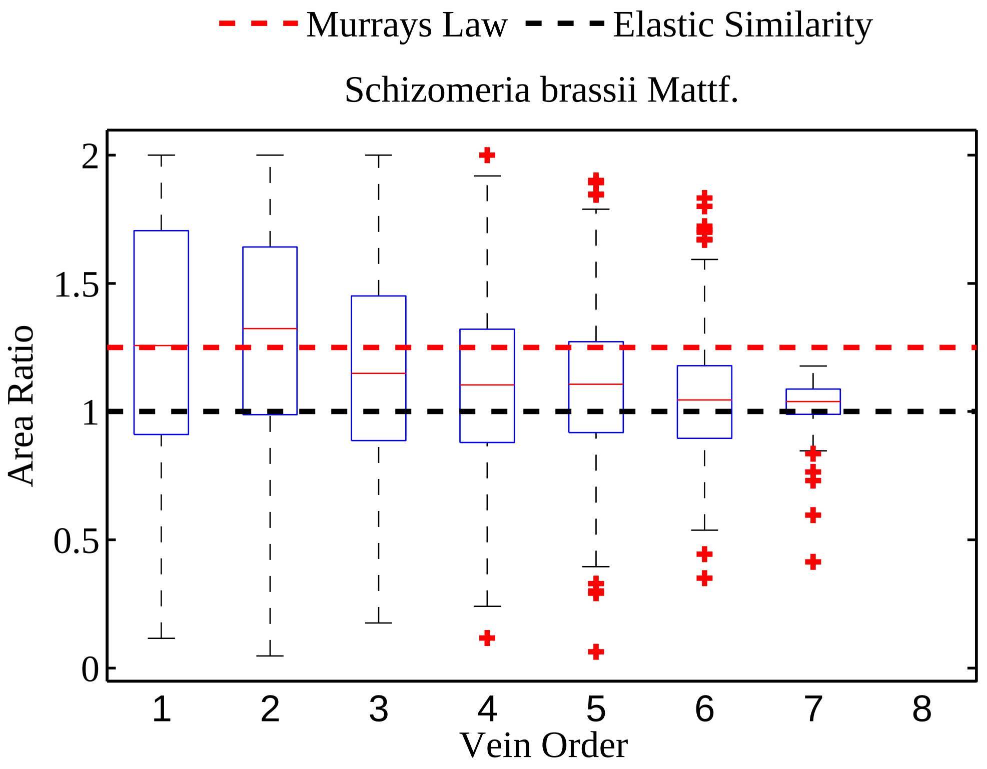
Murrays Law
Elastic Similarity
Schizomeria brassii Mattf.
Area Ratio
0
0.5
1
1.5
2
1
2
3
4
5
6
7
8
Vein Order

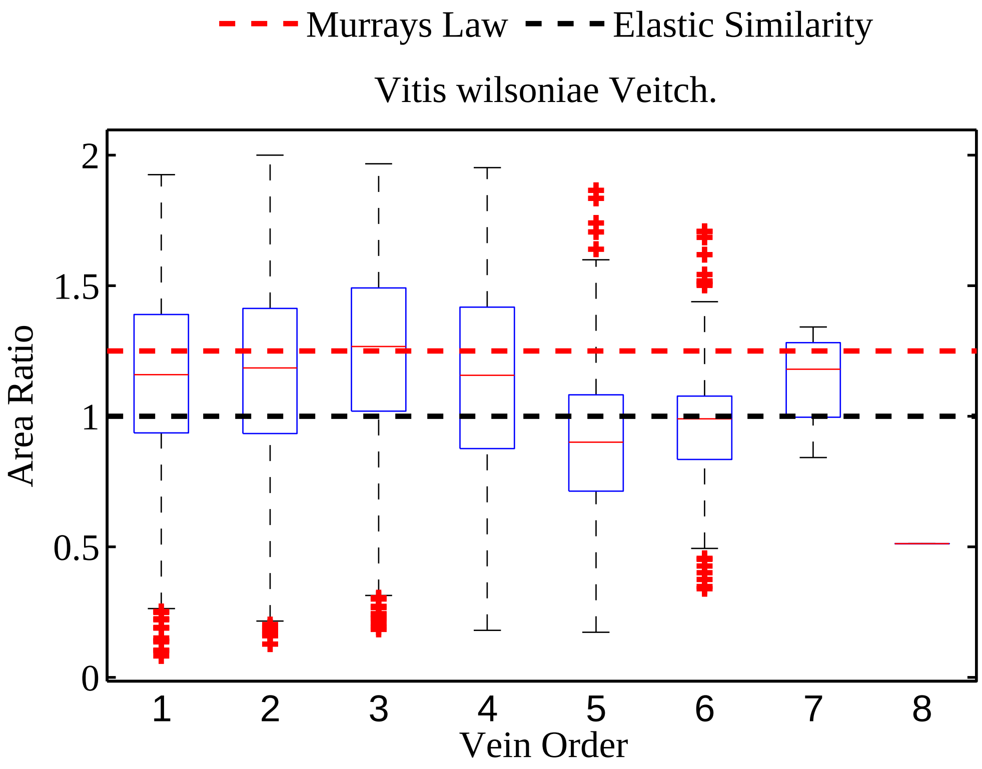

Murrays Law
Elastic Similarity
Vitis wilsoniae Veitch.
Area Ratio
Vein Order
0
0.5
1
1.5
2
1
2
3
4
5
6
7
8

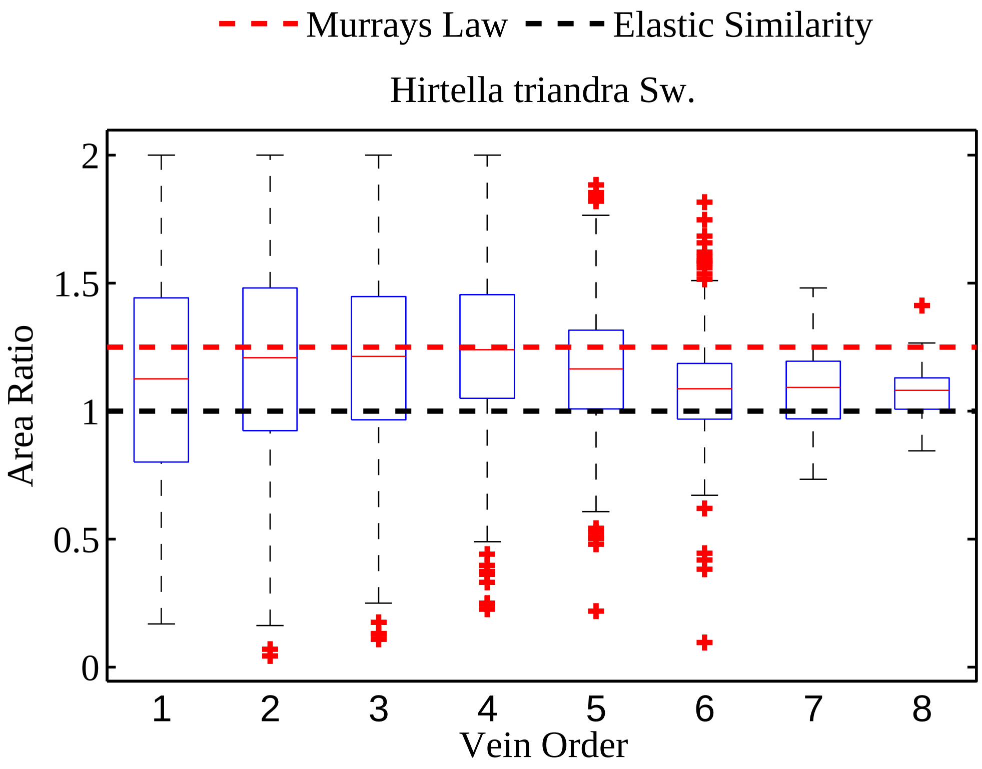

Murrays Law
Elastic Similarity
Hirtella triandra Sw.
Area Ratio
Vein Order
0
0.5
1
1.5
2
1
2
3
4
5
6
7
8

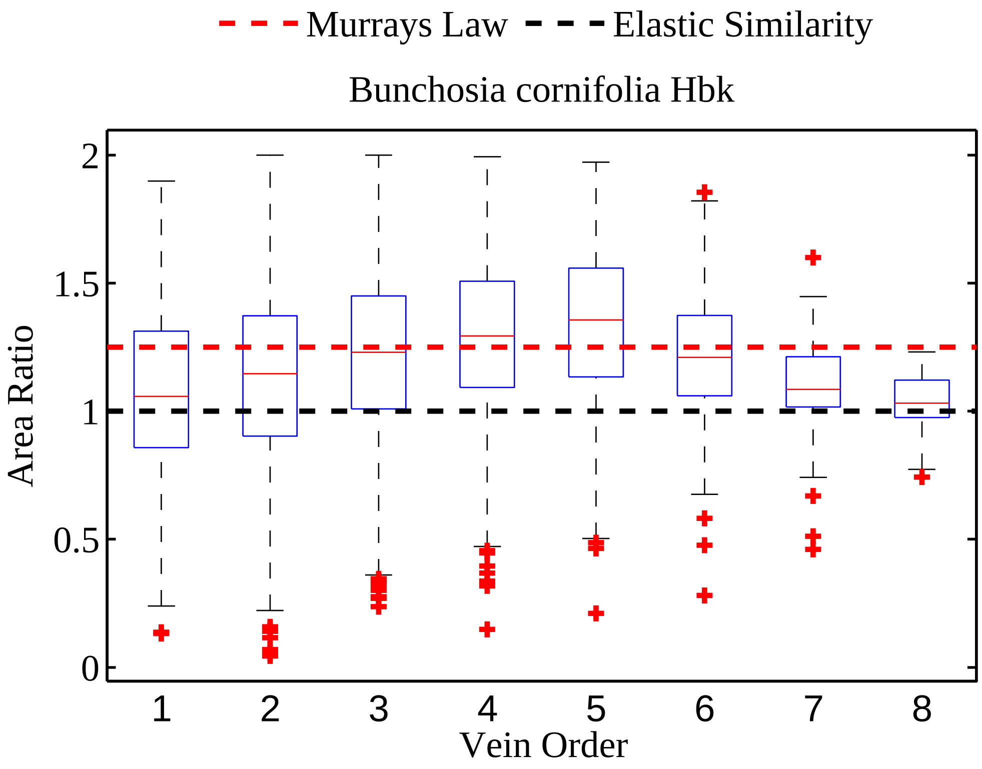

Murrays Law
Elastic Similarity
Bunchosia cornifolia Hbk
Area Ratio
0
0.5
1
1.5
2
1
2
3
4
5
6
7
8
Vein Order

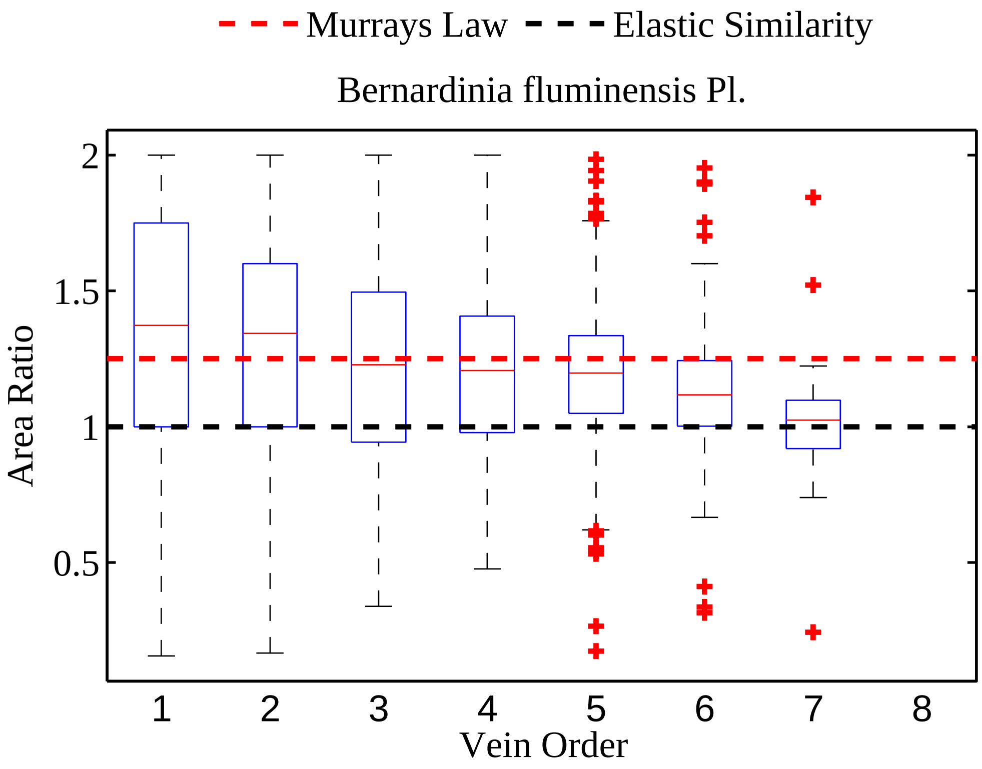

Murrays Law
Elastic Similarity
Bernardinia fluminensis Pl.
Area Ratio
2
1.5
1
0.5
1
2
3
4
5
6
7
8
Vein Order

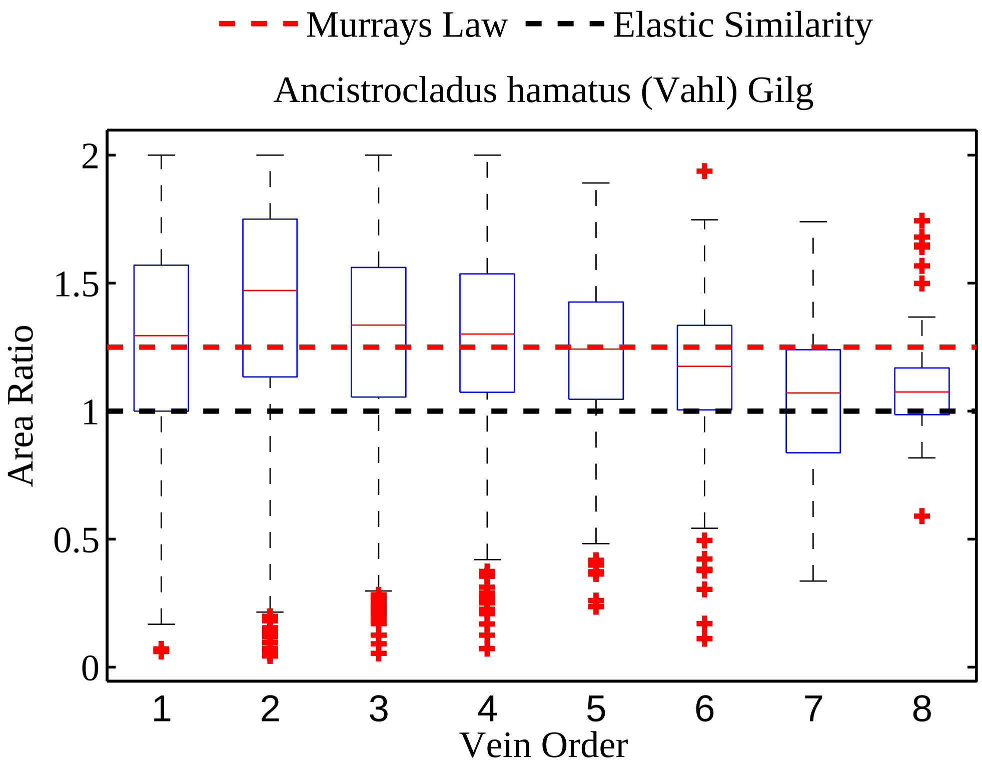
Murrays Law
Elastic Similarity
Ancistrocladus hamatus (Vahl) Gilg
Area Ratio
Vein Order
0
0.5
1
1.5
2
1
2
3
4
5
6
7
8

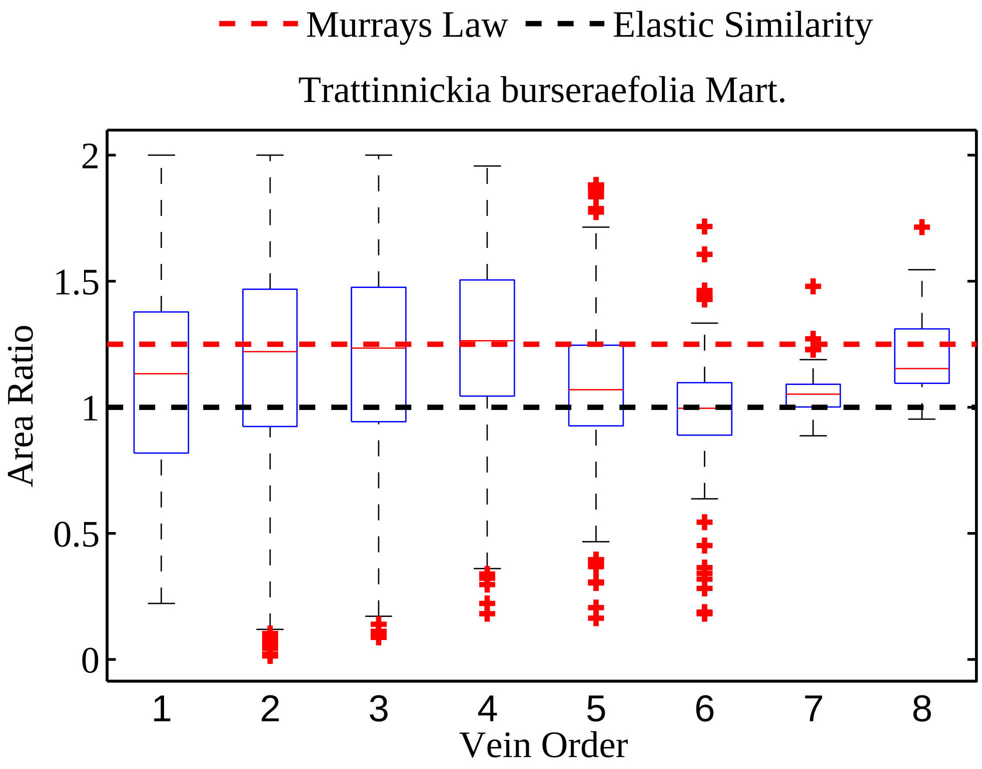

Murrays Law
Elastic Similarity
Trattinnickia burseraefolia Mart.
Area Ratio
Vein Order
0
0.5
1
1.5
2
1
2
3
4
5
6
7
8

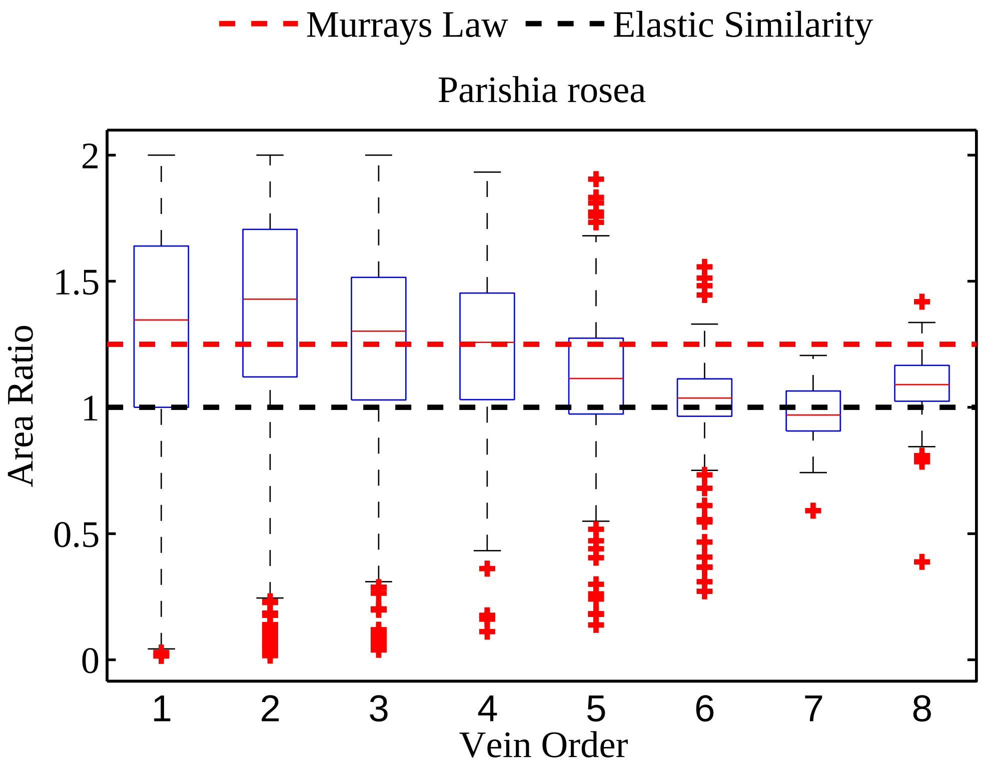

Murrays Law
Elastic Similarity
Parishia rosea
Area Ratio
Vein Order
0
0.5
1
1.5
2
1
2
3
4
5
6
7
8

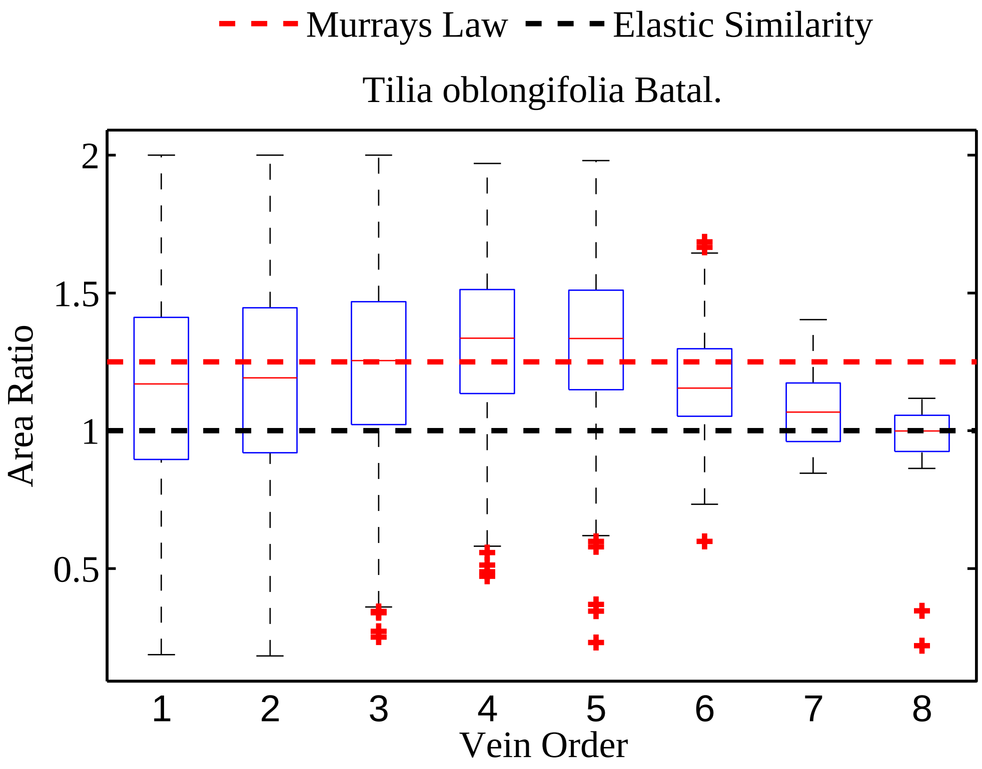

Murrays Law
Elastic Similarity
Tilia oblongifolia Batal.
Area Ratio
0.5
1
1.5
2
1
2
3
4
5
6
7
8
Vein Order

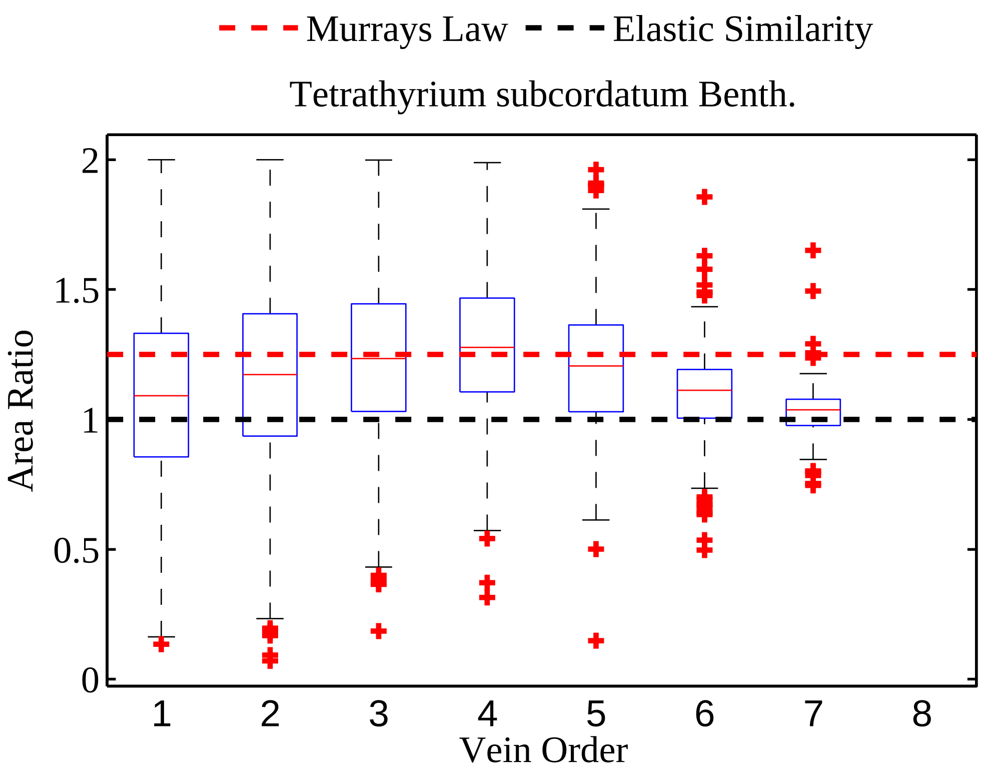

Murrays Law
Elastic Similarity
Tetrathyrium subcordatum Benth.
Area Ratio
0
0.5
1
1.5
2
1
2
3
4
5
6
7
8
Vein Order

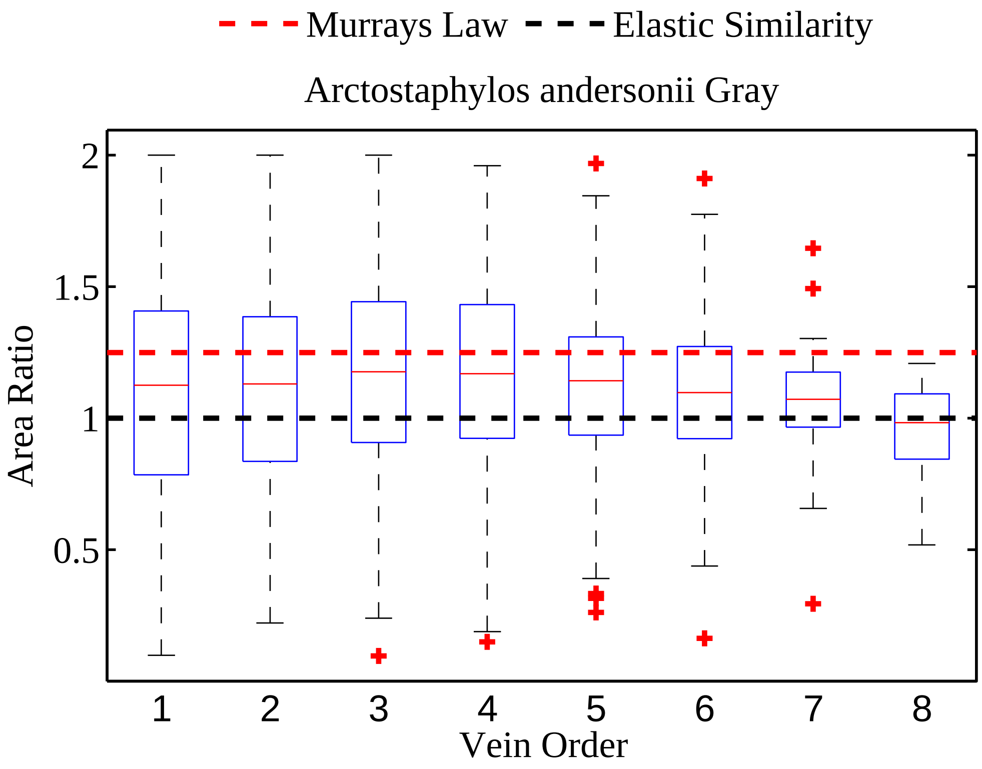

Murrays Law
Elastic Similarity
Arctostaphylos andersonii Gray
Area Ratio
2
1.5
1
0.5
1
2
3
4
5
6
7
8
Vein Order

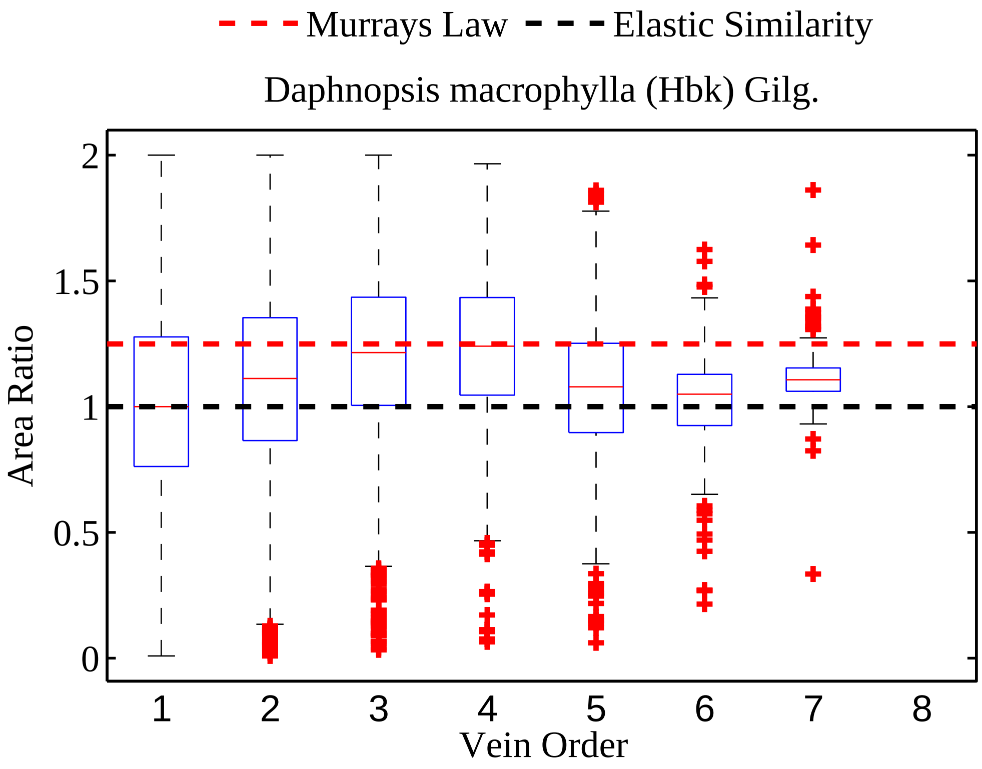

Murrays Law
Elastic Similarity
Daphnopsis macrophylla (Hbk) Gilg.
Area Ratio
0
0.5
1
1.5
2
1
2
3
4
5
6
7
8
Vein Order

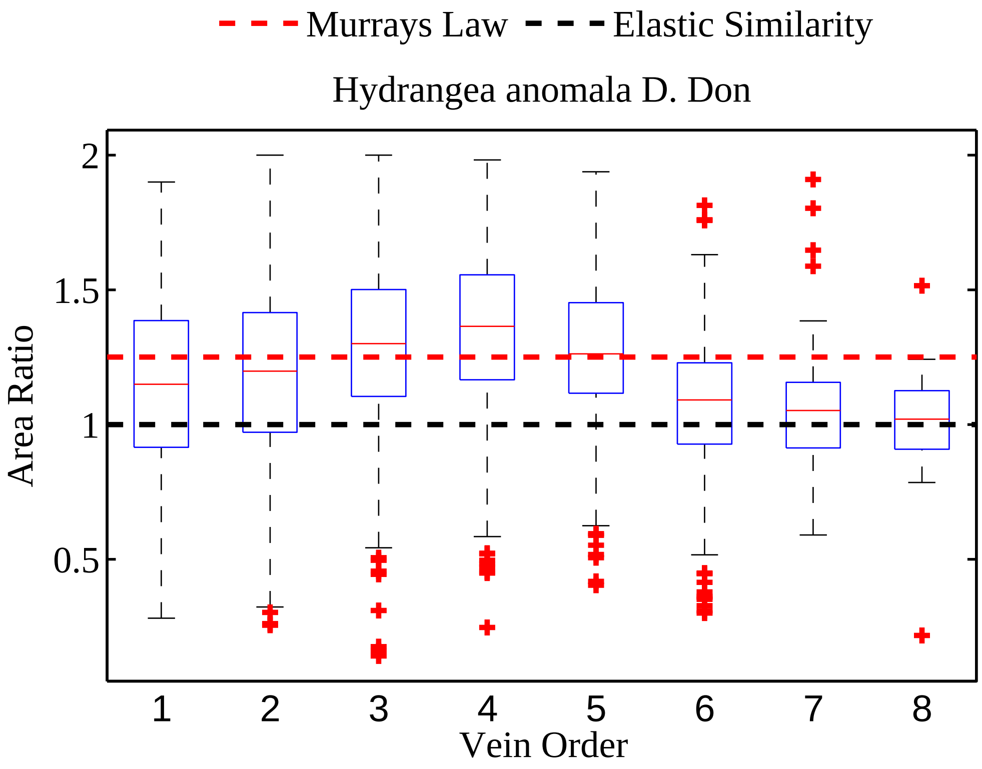

Murrays Law
Elastic Similarity
Hydrangea anomala D. Don
Area Ratio
2
1.5
1
0.5
1
2
3
4
5
6
7
8
Vein Order

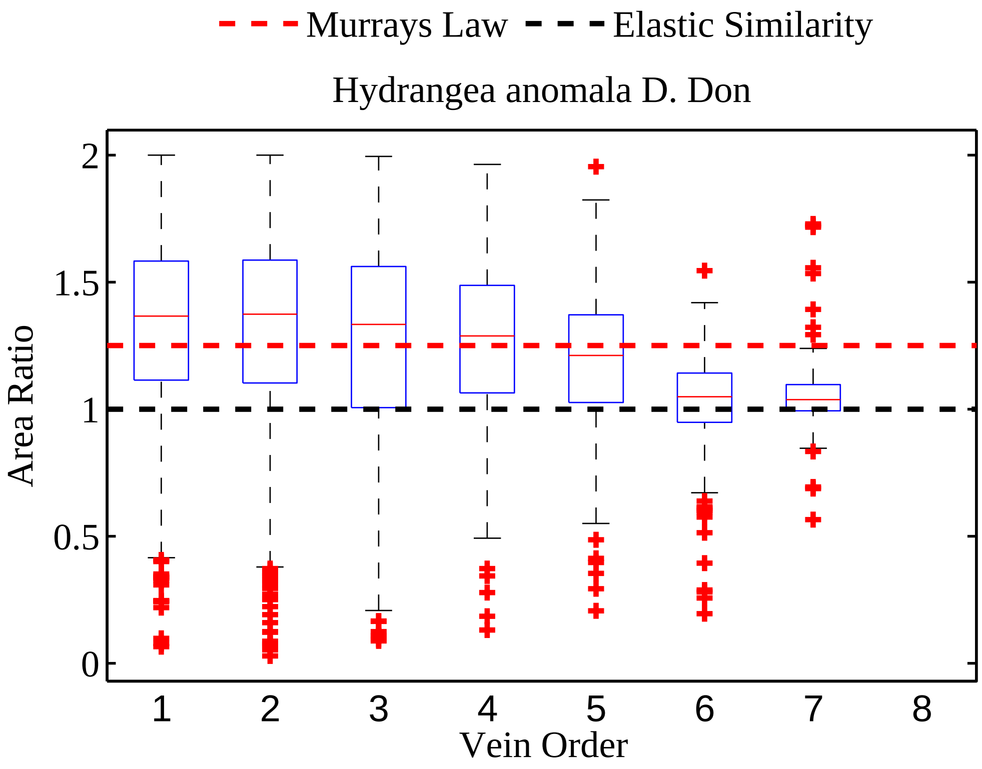

Murrays Law
Elastic Similarity
Hydrangea anomala D. Don
Area Ratio
Vein Order
0
0.5
1
1.5
2
1
2
3
4
5
6
7
8

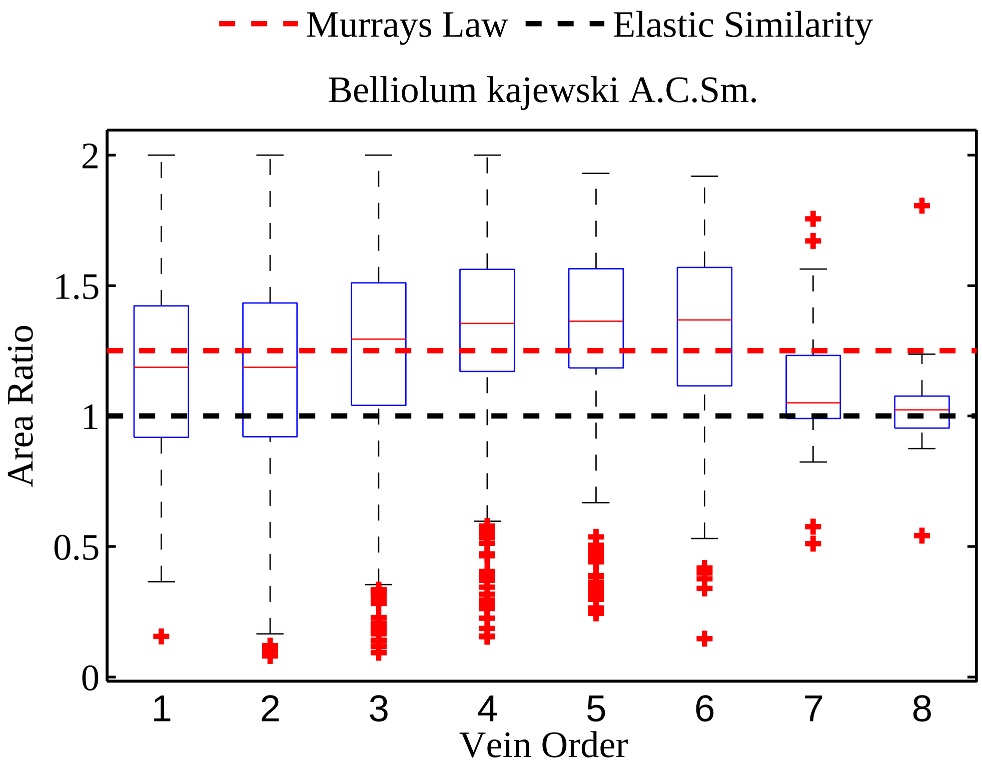
Murrays Law
Elastic Similarity
Belliolum kajewski A.C.Sm.
Area Ratio
Vein Order
0
0.5
1
1.5
2
1
2
3
4
5
6
7
8

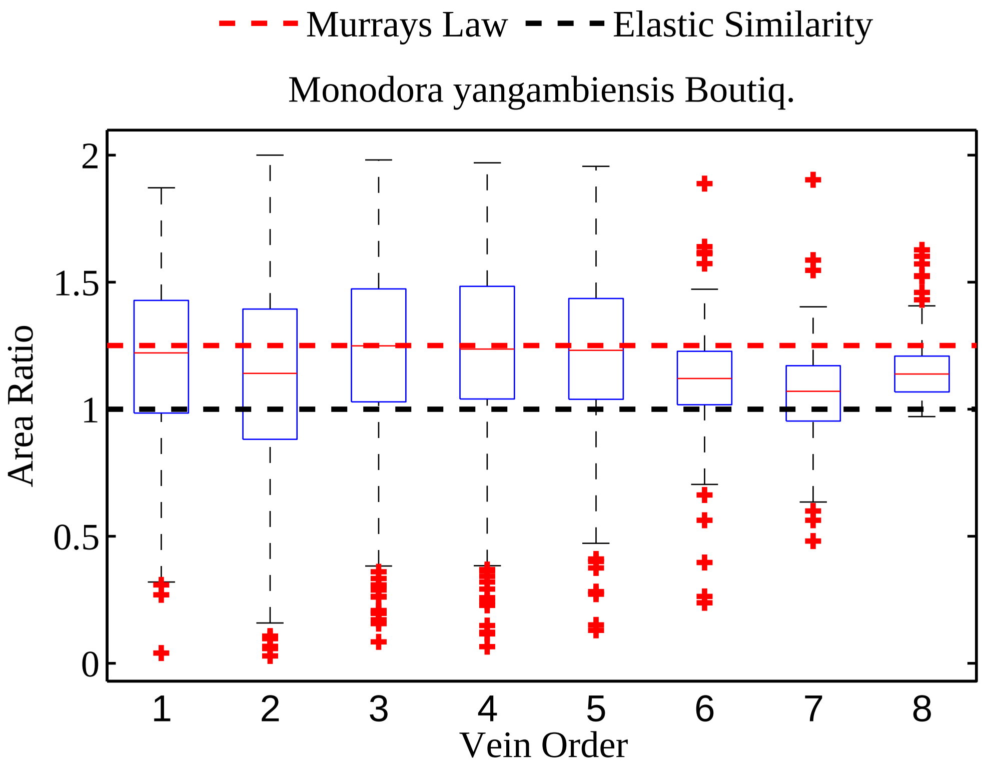

Murrays Law
Elastic Similarity
Monodora yangambiensis Boutiq.
Area Ratio
0
0.5
1
1.5
2
1
2
3
4
5
6
7
8
Vein Order

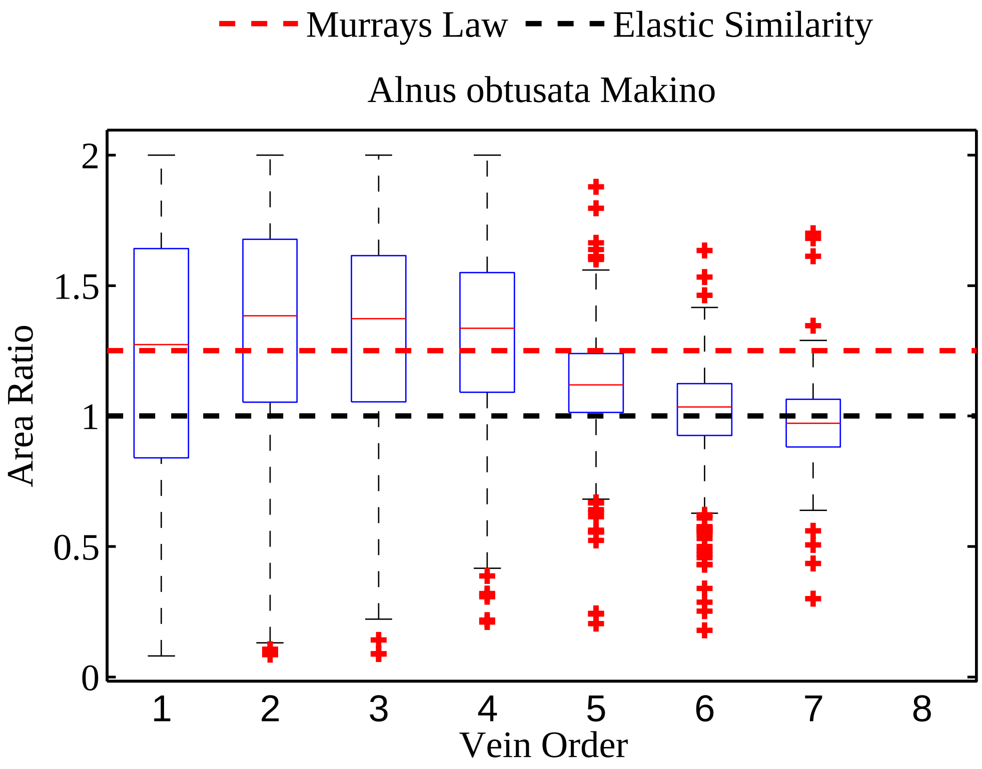

Murrays Law
Elastic Similarity
Alnus obtusata Makino
Area Ratio
Vein Order
0
0.5
1
1.5
2
1
2
3
4
5
6
7
8

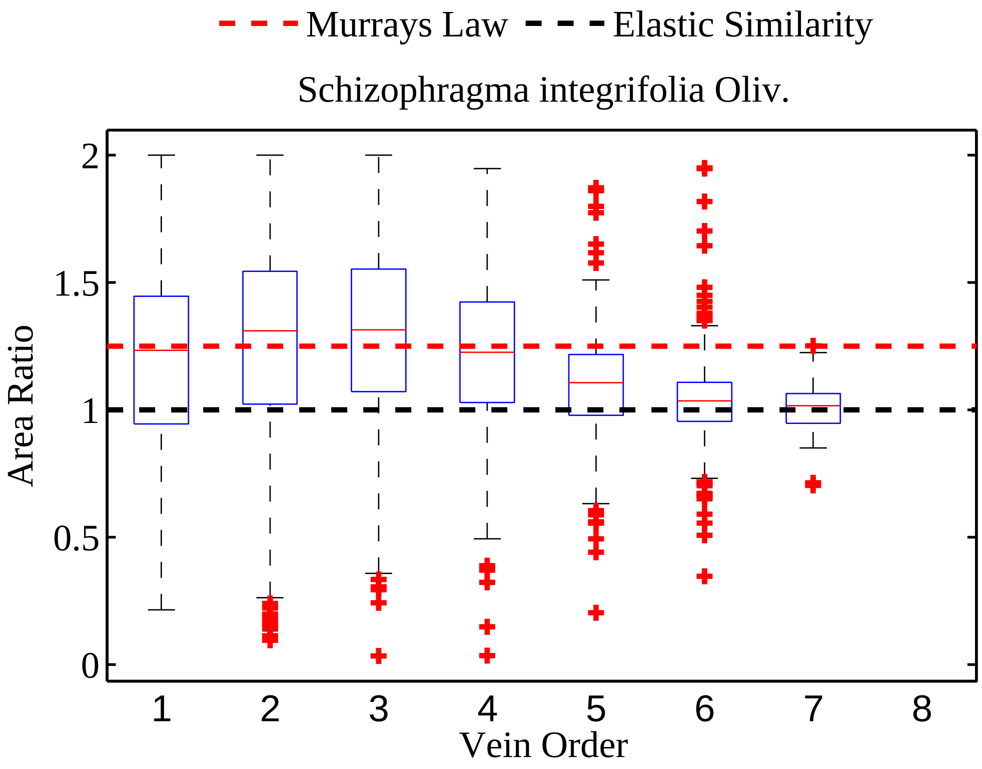
Murrays Law
Elastic Similarity
Schizophragma integrifolia Oliv.
Area Ratio
Vein Order
0
0.5
1
1.5
2
1
2
3
4
5
6
7
8

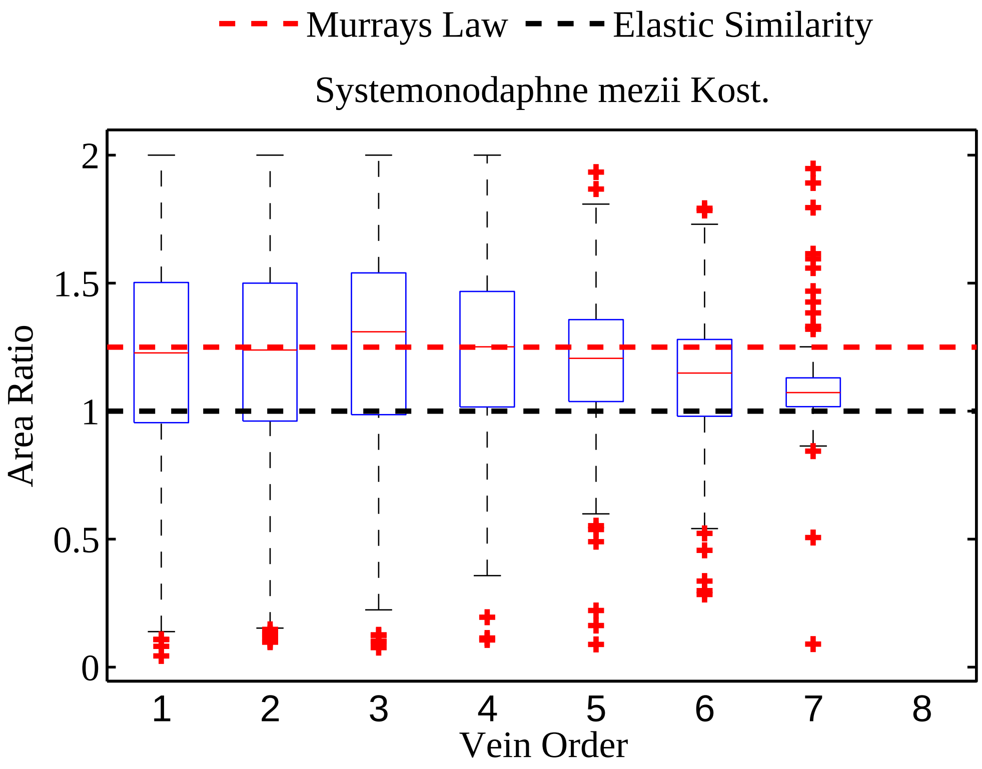
Murrays Law
Elastic Similarity
Systemonodaphne mezii Kost.
Area Ratio
Vein Order
2
1.5
1
0.5
0
1
2
3
4
5
6
7
8

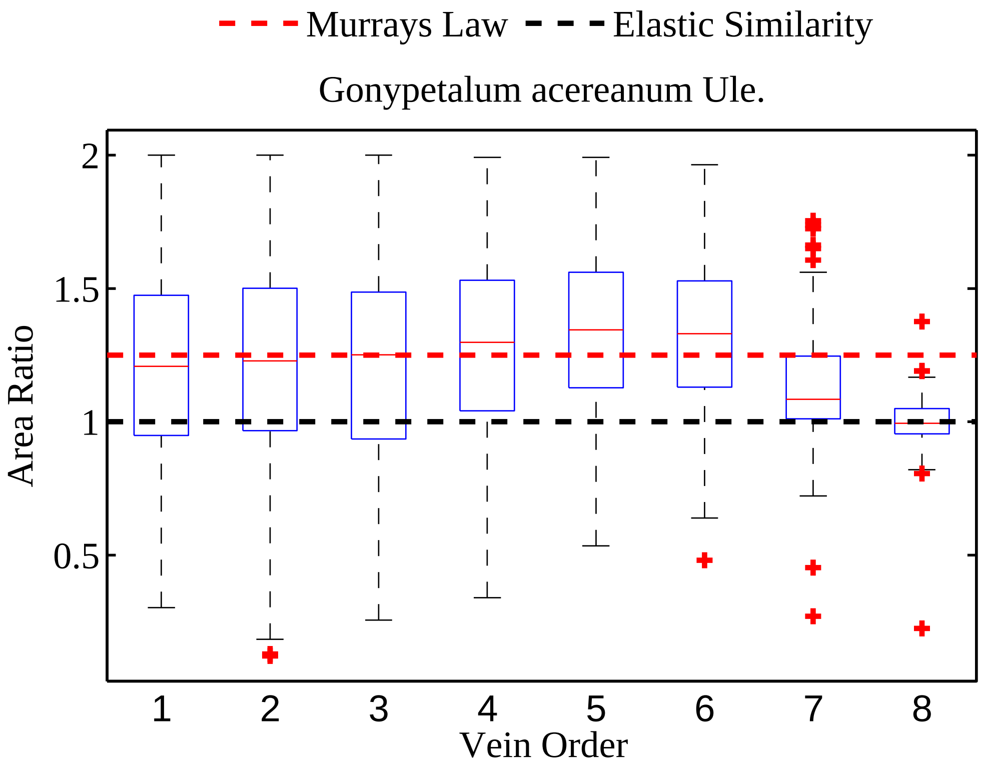

Murrays Law
Elastic Similarity
Gonypetalum acereanum Ule.
Area Ratio
2
1.5
1
0.5
1
2
3
4
5
6
7
8
Vein Order

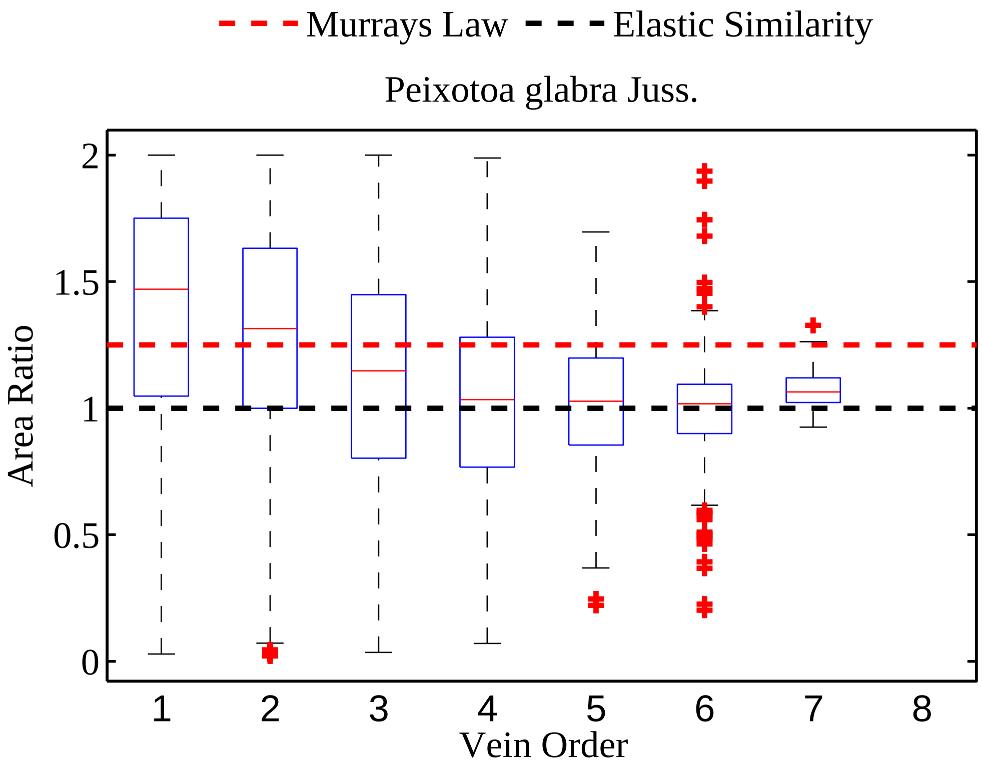

Murrays Law
Elastic Similarity
Peixotoa glabra Juss.
Area Ratio
2
1.5
1
0.5
0
1
2
3
4
5
6
7
8
Vein Order

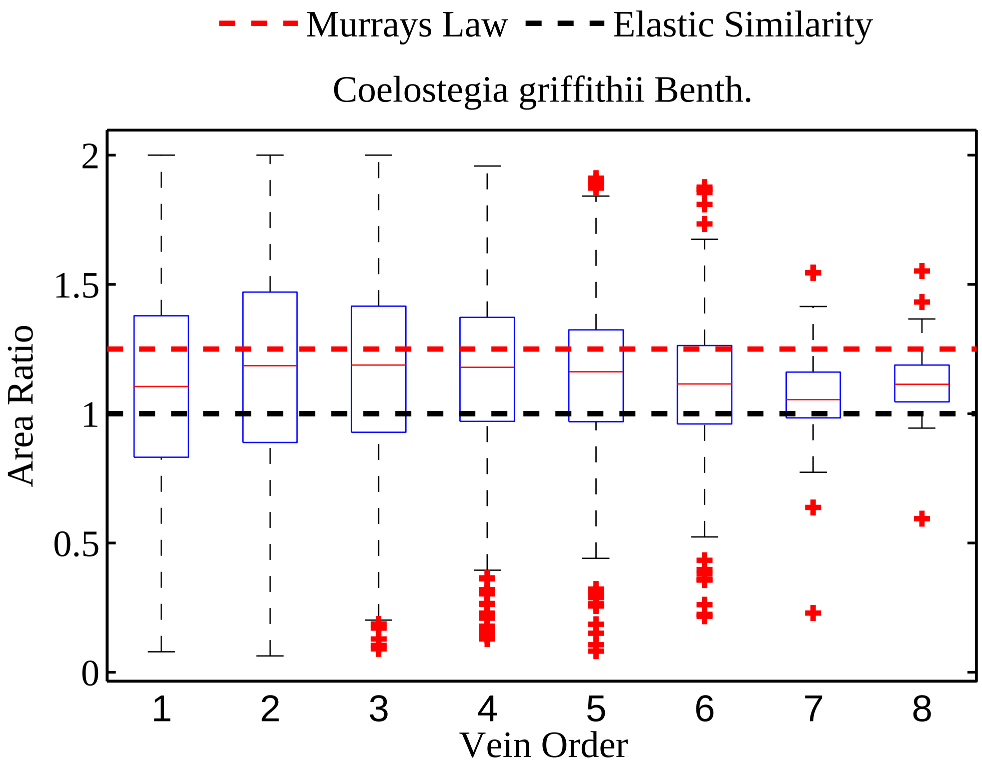

Murrays Law
Elastic Similarity
Coelostegia griffithii Benth.
Area Ratio
0
0.5
1
1.5
2
1
2
3
4
5
6
7
8
Vein Order

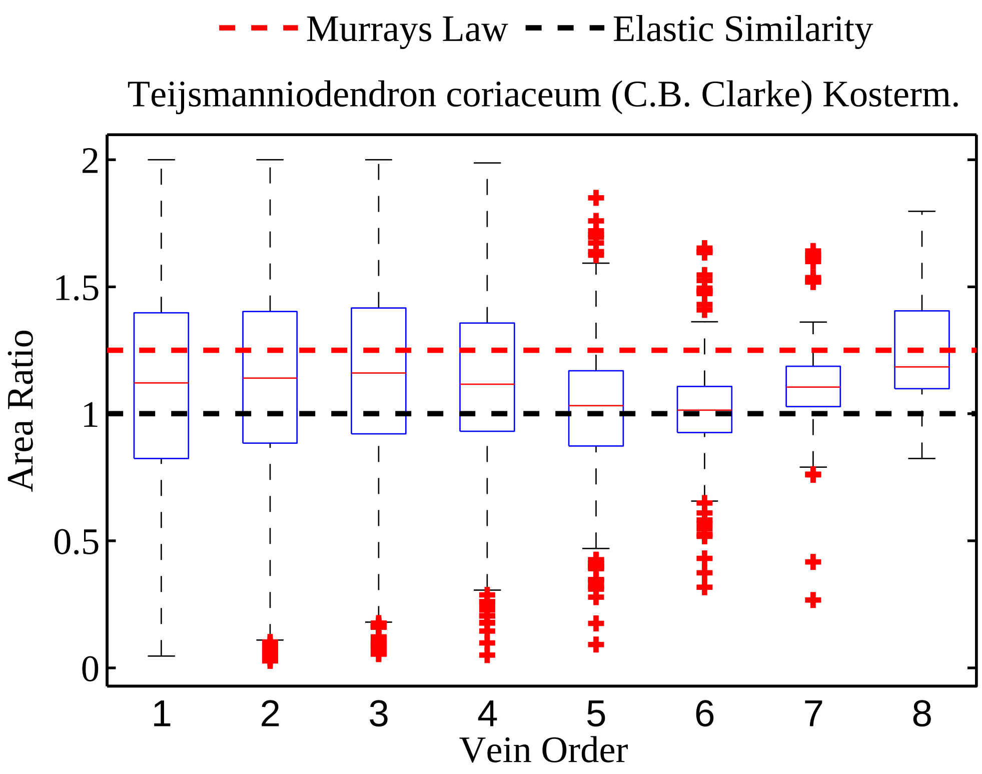
Murrays Law
Elastic Similarity
Teijsmanniodendron coriaceum (C.B. Clarke) Kosterm.
Area Ratio
Vein Order
0
0.5
1
1.5
2
1
2
3
4
5
6
7
8

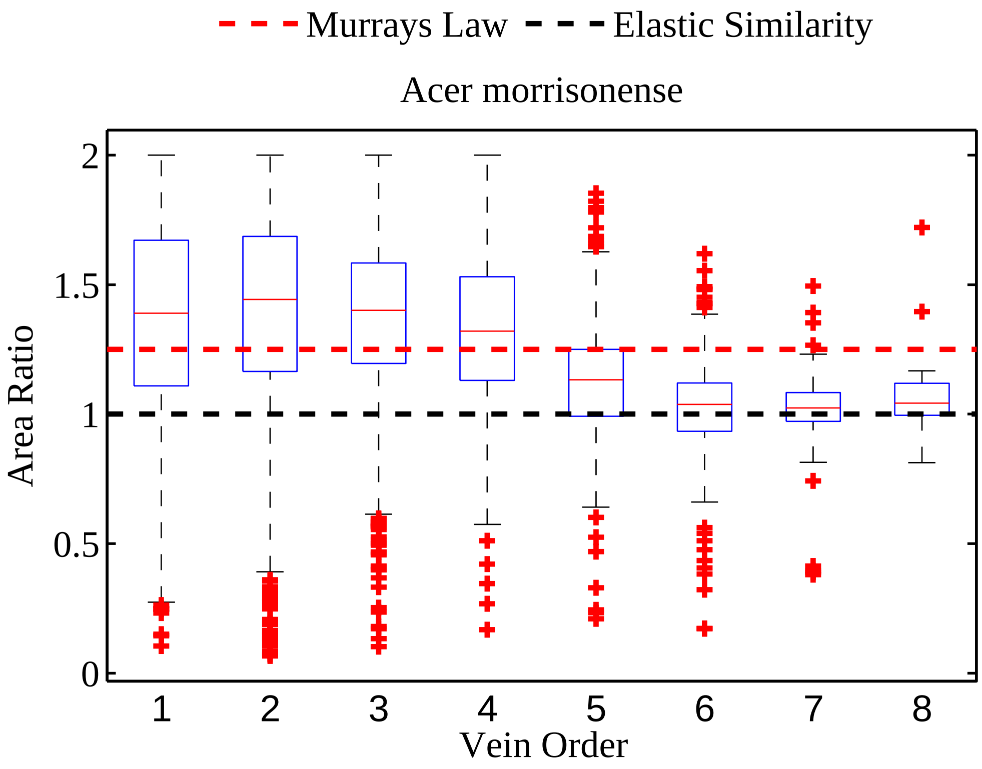
Murrays Law
Elastic Similarity
Acer morrisonense
Area Ratio
Vein Order
0
0.5
1
1.5
2
1
2
3
4
5
6
7
8

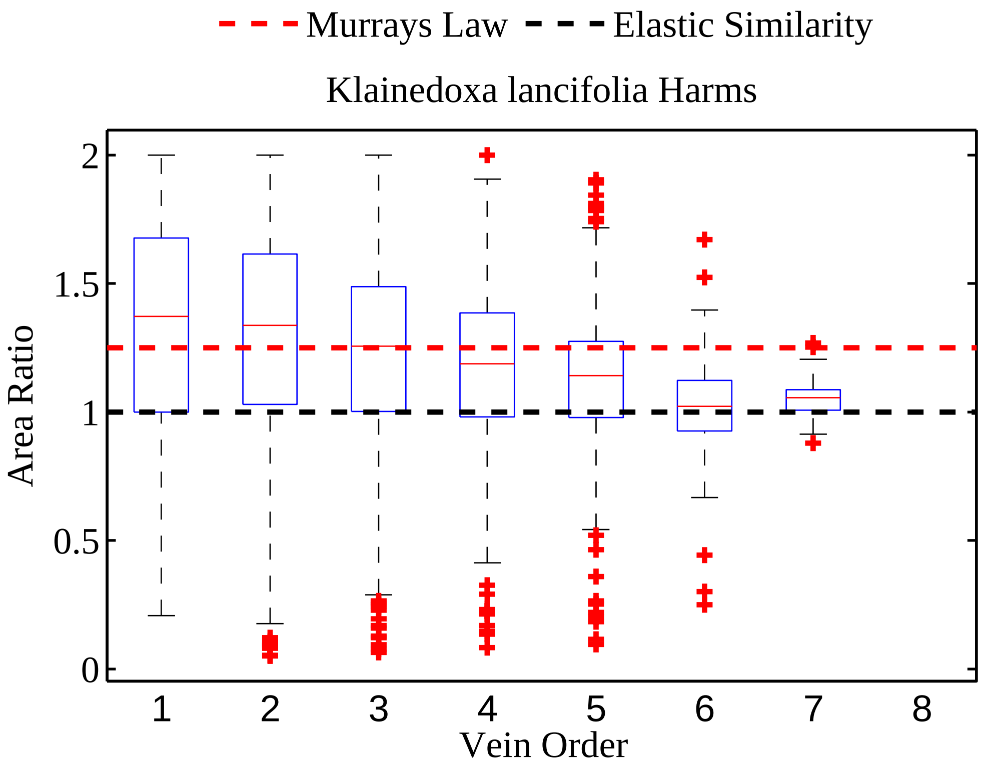
Murrays Law
Elastic Similarity
Klainedoxa lancifolia Harms
Area Ratio
2
1.5
1
0.5
0
1
2
3
4
5
6
7
8
Vein Order

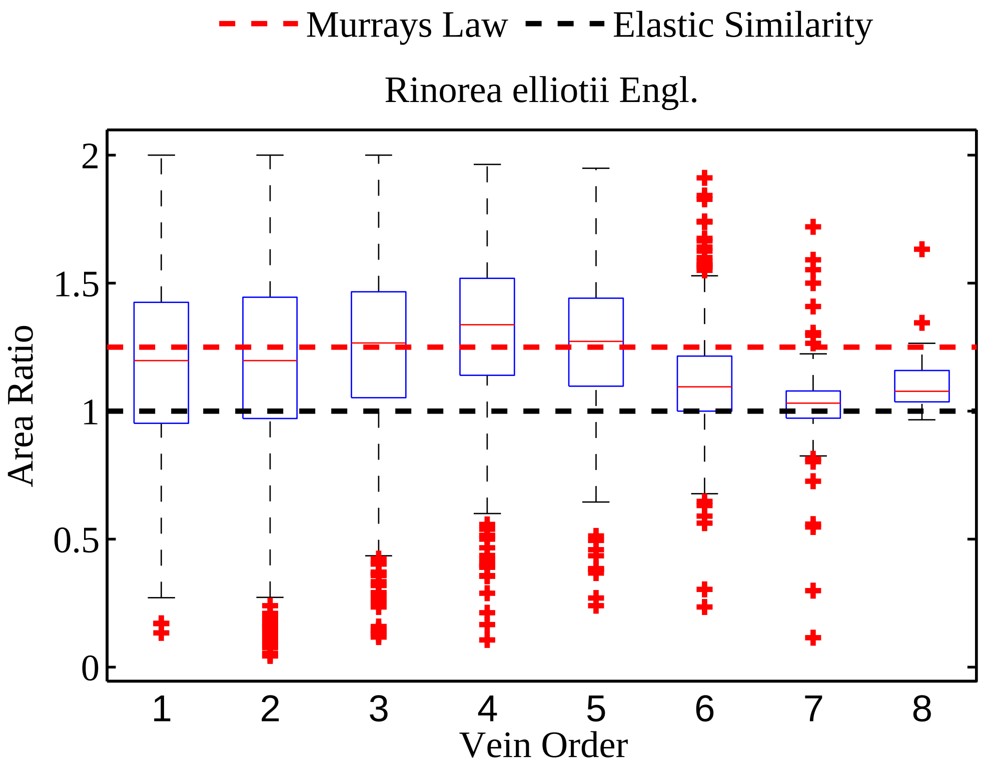

Murrays Law
Elastic Similarity
Rinorea elliotii Engl.
Area Ratio
0
0.5
1
1.5
2
1
2
3
4
5
6
7
8
Vein Order

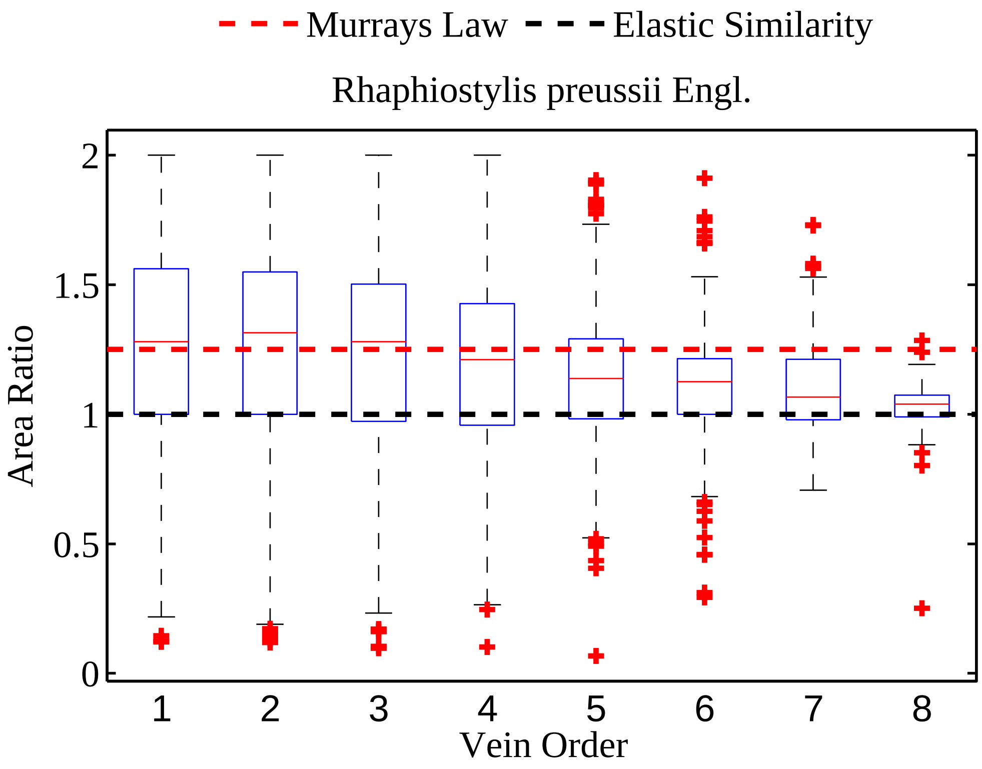

Murrays Law
Elastic Similarity
Rhaphiostylis preussii Engl.
Area Ratio
Vein Order
0
0.5
1
1.5
2
1
2
3
4
5
6
7
8

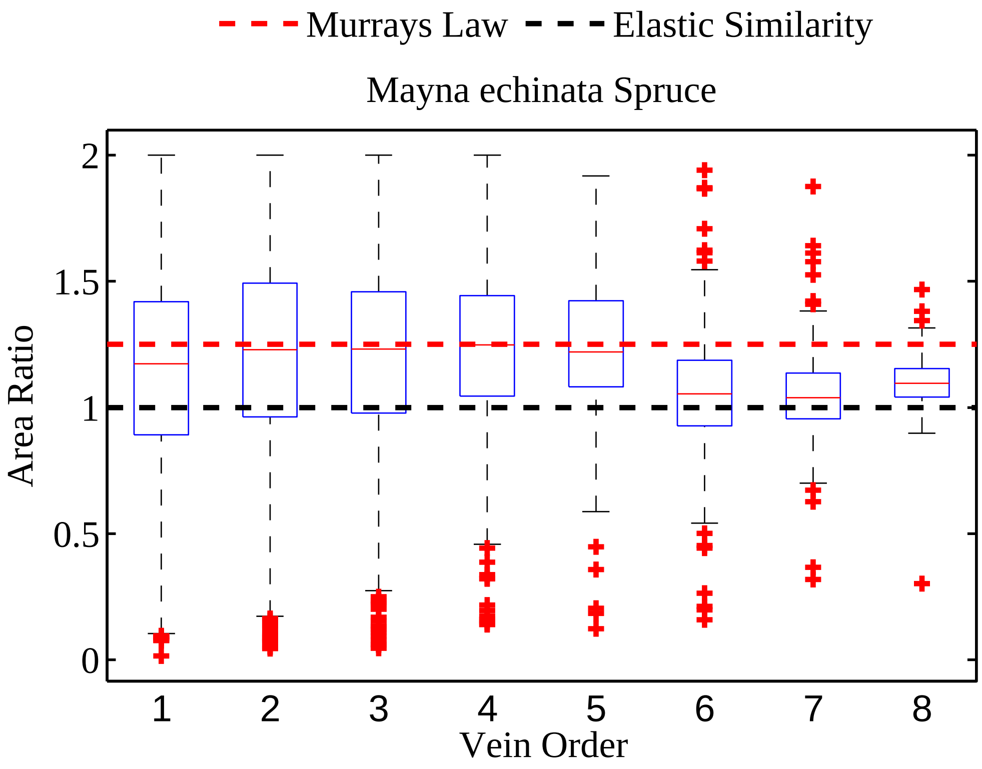
Murrays Law
Elastic Similarity
Mayna echinata Spruce
Area Ratio
0
0.5
1
1.5
2
1
2
3
4
5
6
7
8
Vein Order

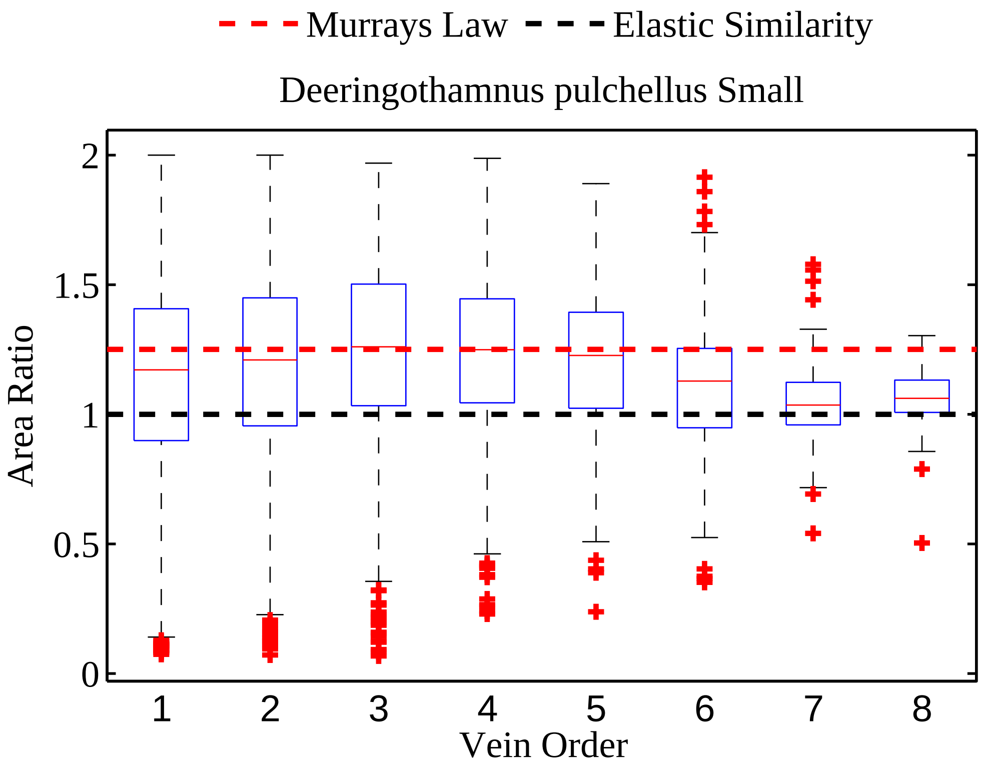

Murrays Law
Elastic Similarity
Deeringothamnus pulchellus Small
Area Ratio
0
0.5
1
1.5
2
1
2
3
4
5
6
7
8
Vein Order

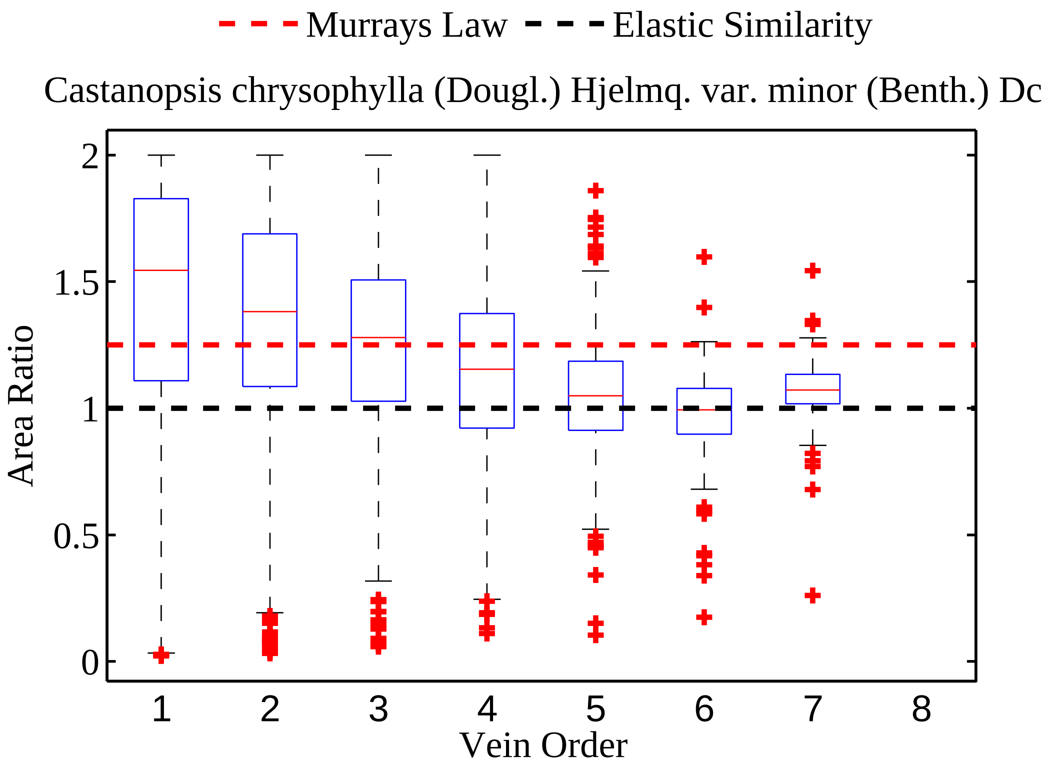

Murrays Law
Elastic Similarity
Castanopsis chrysophylla (Dougl.) Hjelmq. var. minor (Benth.) Dc
Area Ratio
0
0.5
1
1.5
2
1
2
3
4
5
6
7
8
Vein Order

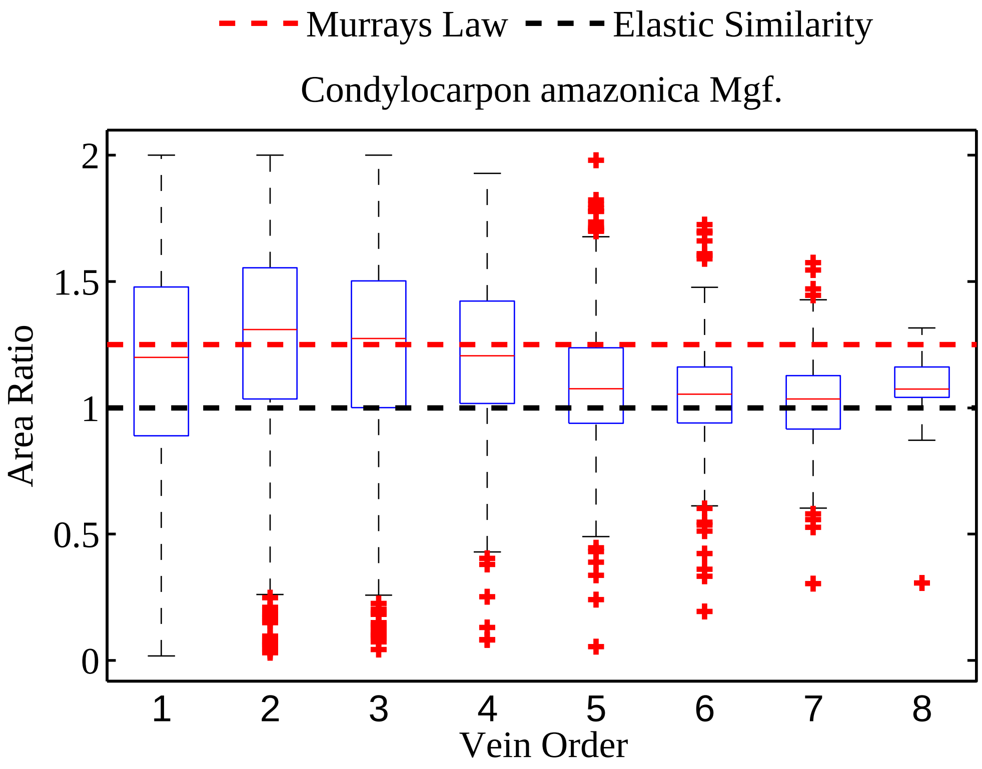

Murrays Law
Elastic Similarity
Condylocarpon amazonica Mgf.
Area Ratio
0
0.5
1
1.5
2
1
2
3
4
5
6
7
8
Vein Order

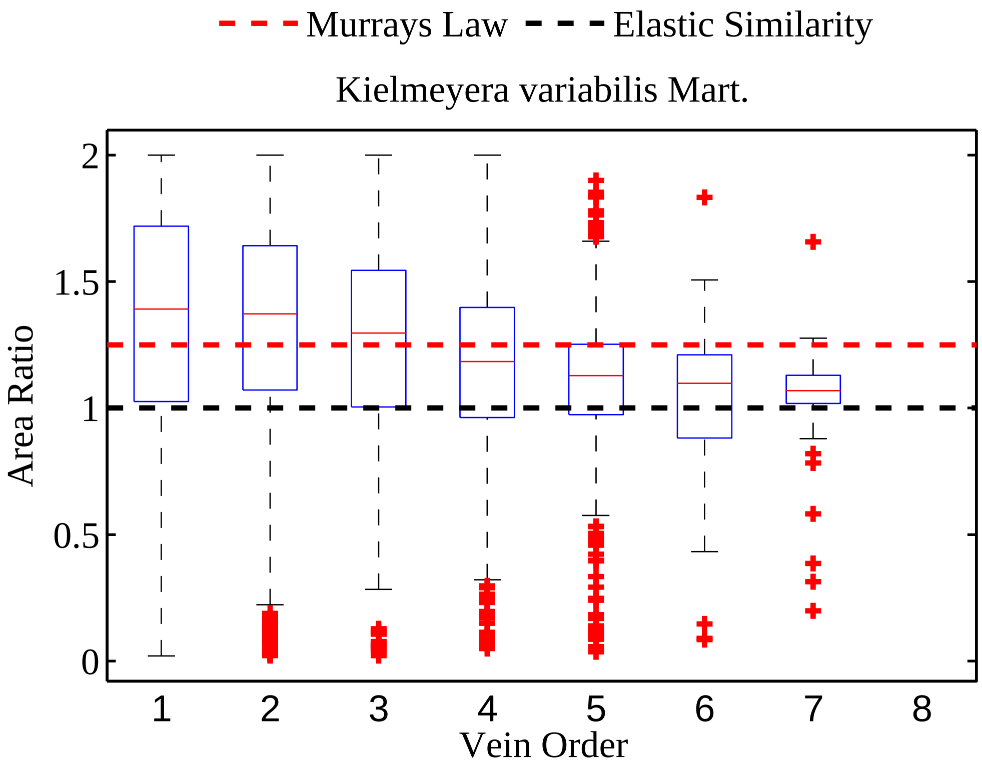

Murrays Law
Elastic Similarity
Kielmeyera variabilis Mart.
Area Ratio
2
1.5
1
0.5
0
1
2
3
4
5
6
7
8
Vein Order

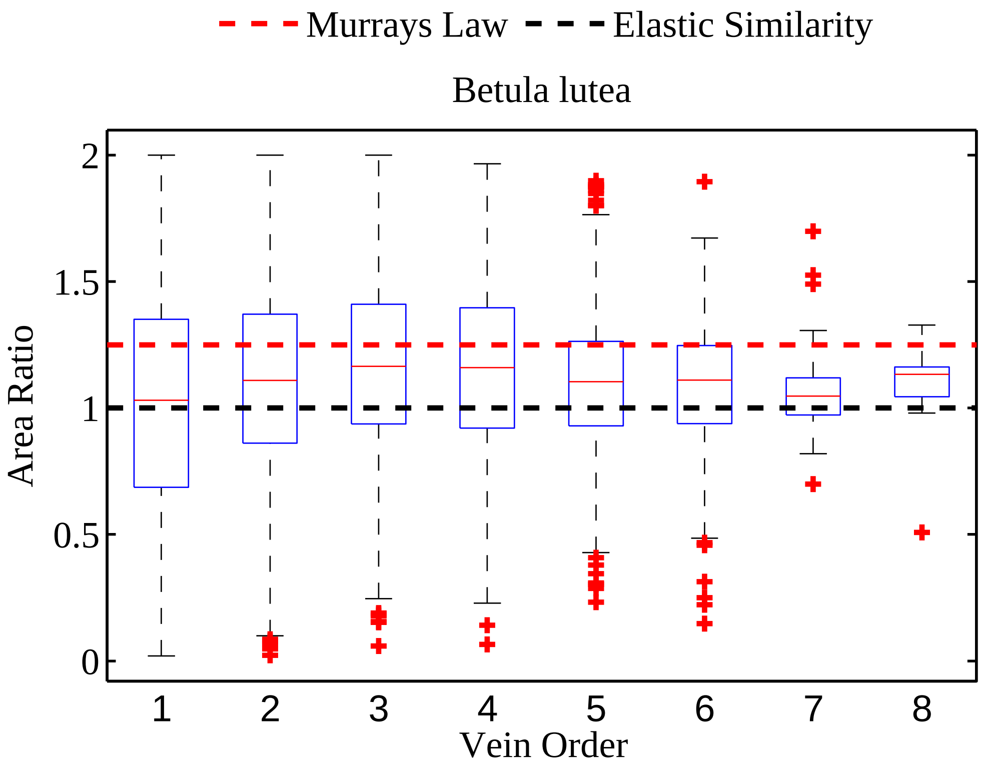

Murrays Law
Elastic Similarity
Betula lutea
Area Ratio
2
1.5
1
0.5
0
1
2
3
4
5
6
7
8
Vein Order

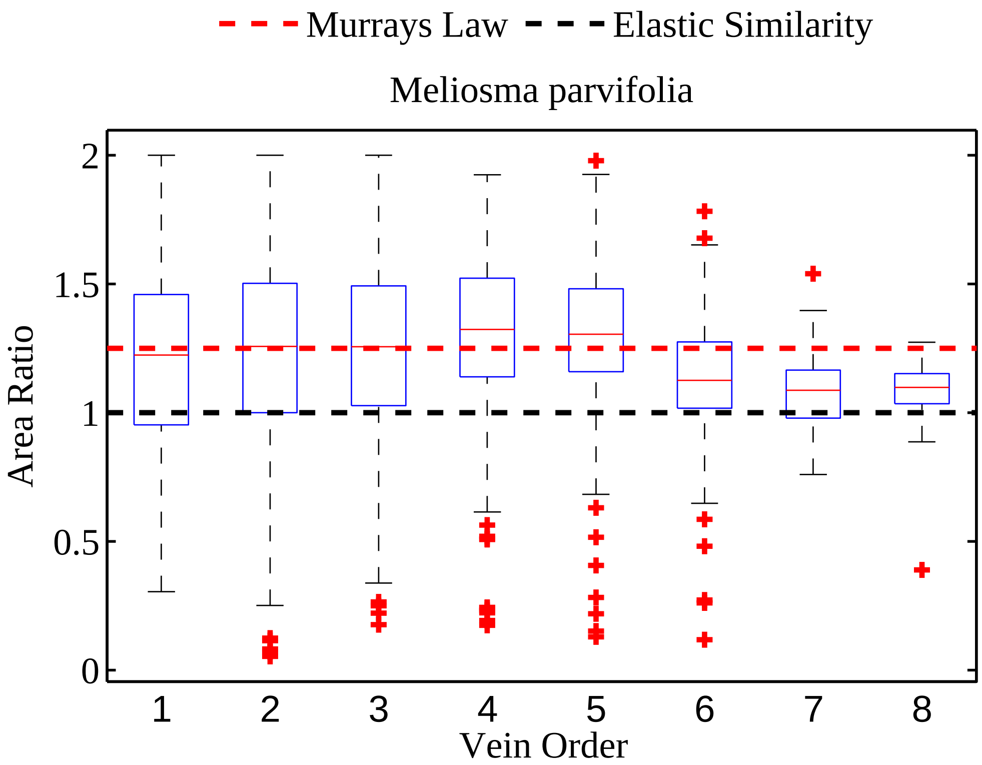

Murrays Law
Elastic Similarity
Meliosma parvifolia
Area Ratio
2
1.5
1
0.5
0
1
2
3
4
5
6
7
8
Vein Order

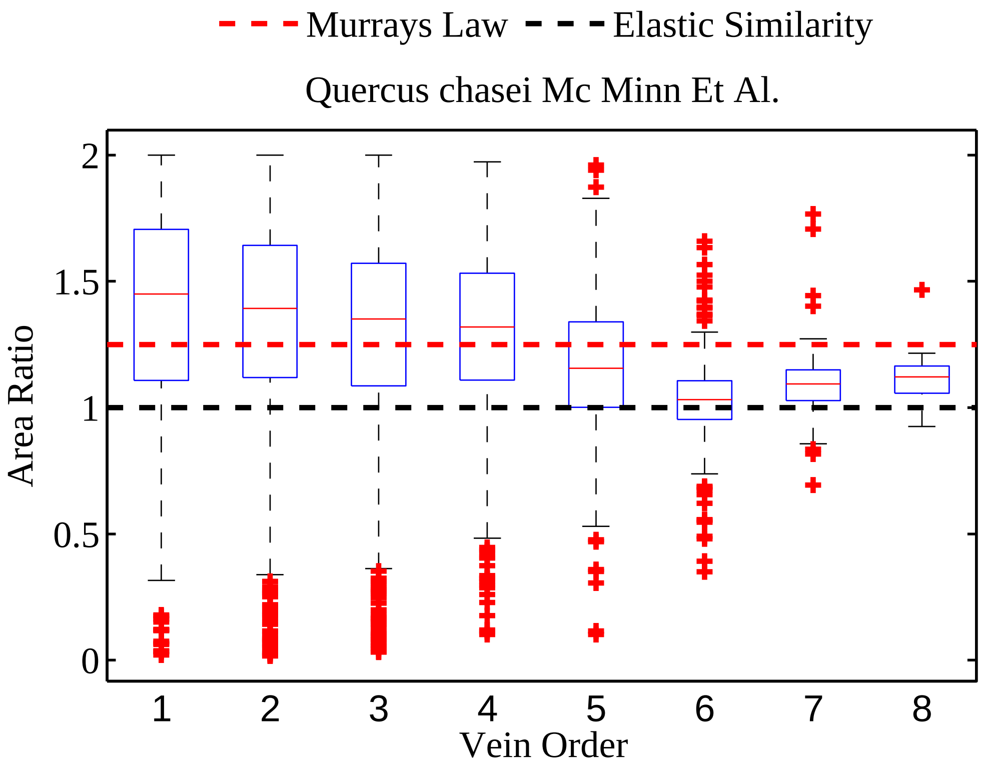

Murrays Law
Elastic Similarity
Quercus chasei Mc Minn Et Al.
Area Ratio
Vein Order
2
1.5
1
0.5
0
1
2
3
4
5
6
7
8

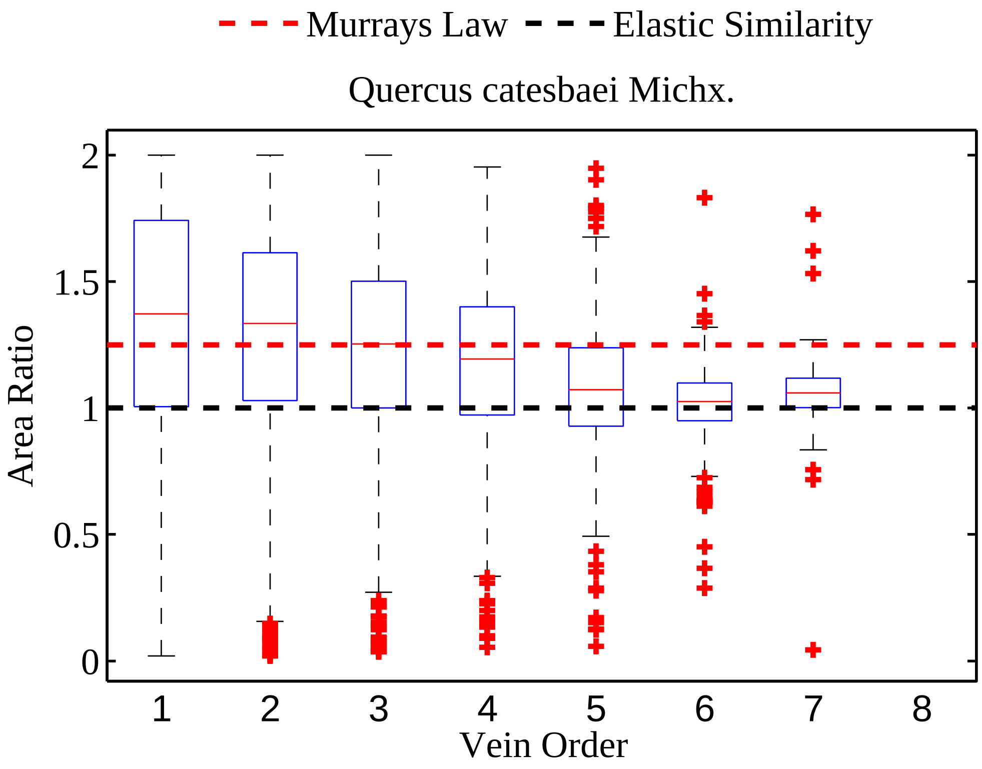

Murrays Law
Elastic Similarity
Quercus catesbaei Michx.
Area Ratio
0
0.5
1
1.5
2
1
2
3
4
5
6
7
8
Vein Order

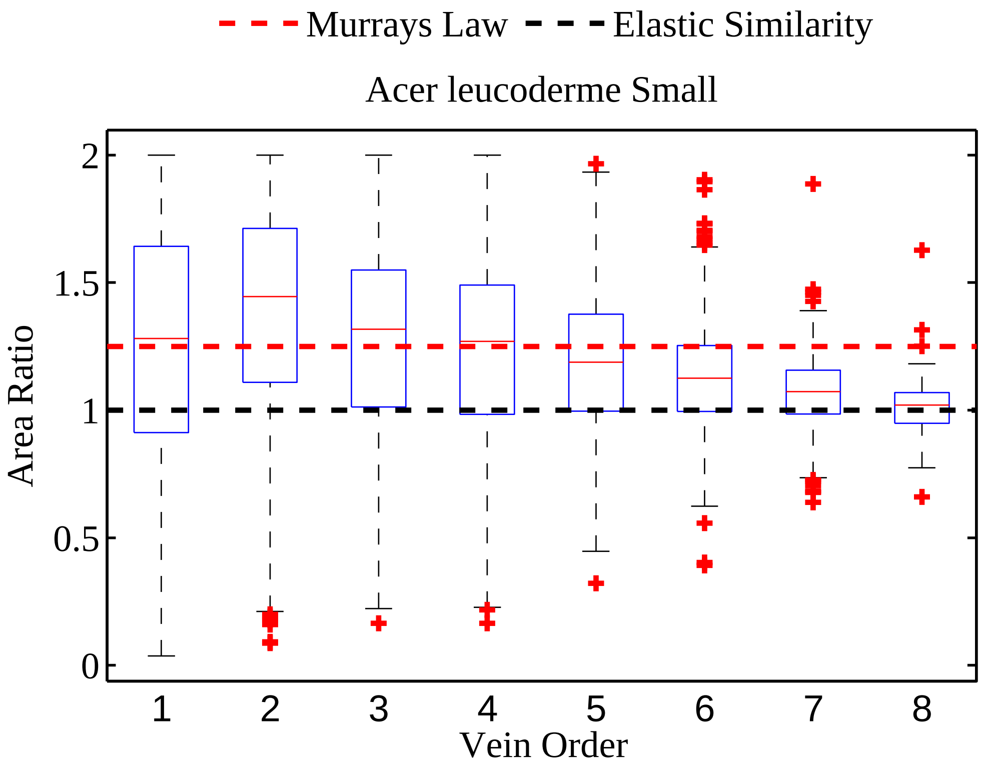

Murrays Law
Elastic Similarity
Acer leucoderme Small
Area Ratio
0
0.5
1
1.5
2
1
2
3
4
5
6
7
8
Vein Order

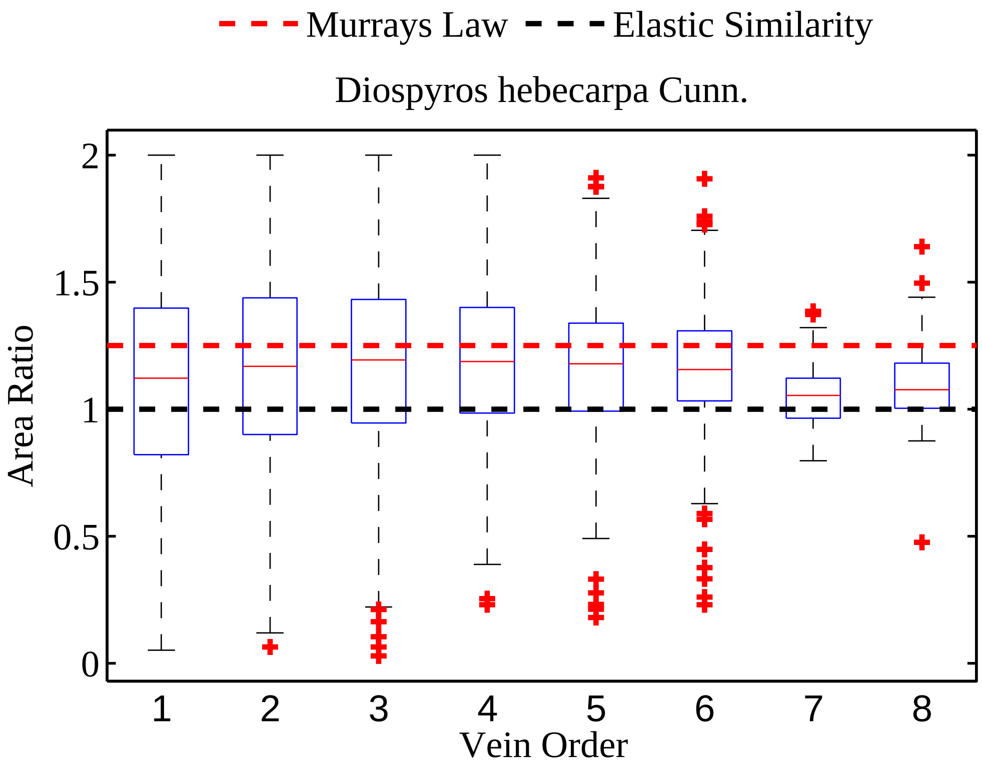
Murrays Law
Elastic Similarity
Diospyros hebecarpa Cunn.
Area Ratio
0
0.5
1
1.5
2
1
2
3
4
5
6
7
8
Vein Order

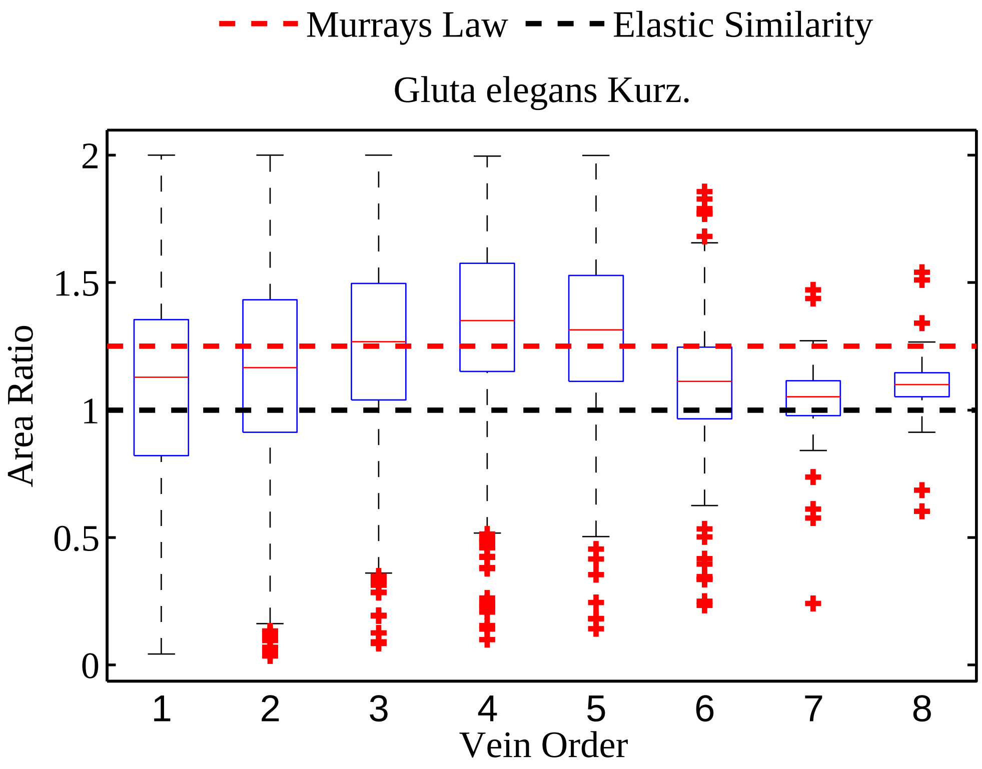

Murrays Law
Elastic Similarity
Gluta elegans Kurz.
Area Ratio
0
0.5
1
1.5
2
1
2
3
4
5
6
7
8
Vein Order

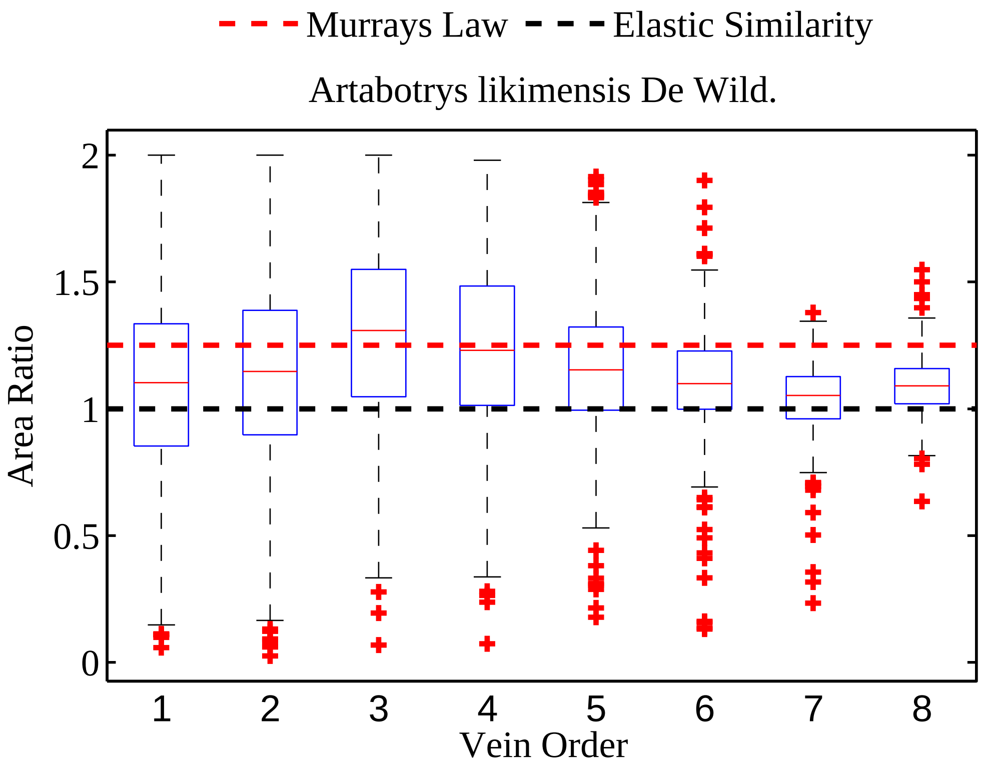

Murrays Law
Elastic Similarity
Artabotrys likimensis De Wild.
Area Ratio
Vein Order
0
0.5
1
1.5
2
1
2
3
4
5
6
7
8

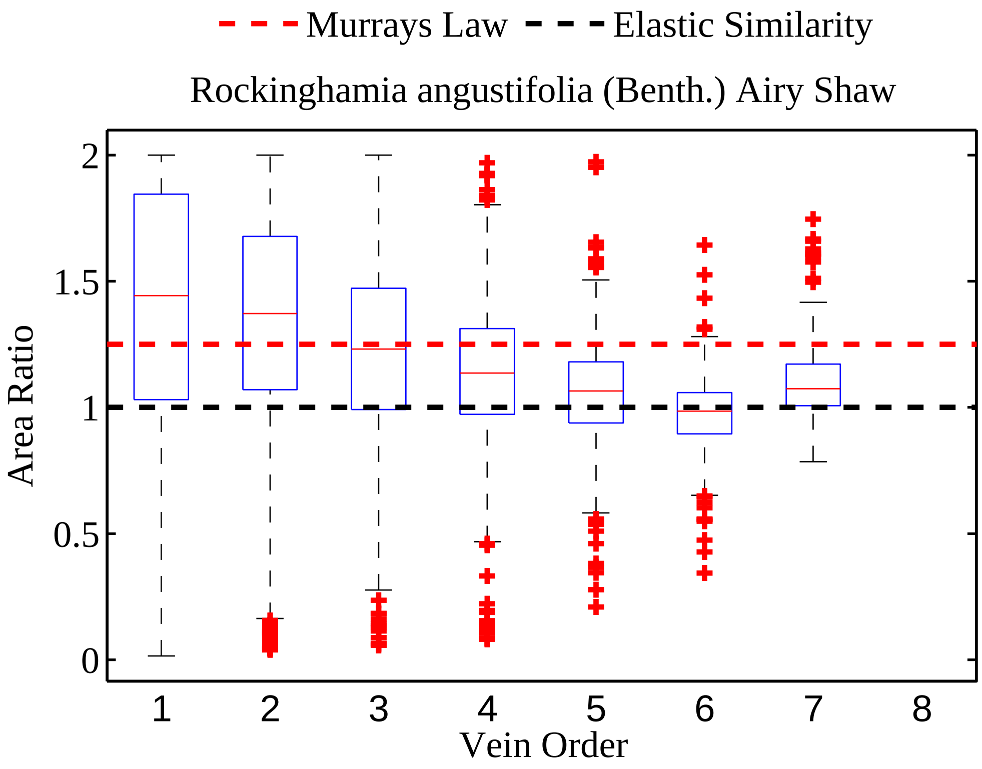

Murrays Law
Elastic Similarity
Rockinghamia angustifolia (Benth.) Airy Shaw
Area Ratio
Vein Order
0
0.5
1
1.5
2
1
2
3
4
5
6
7
8

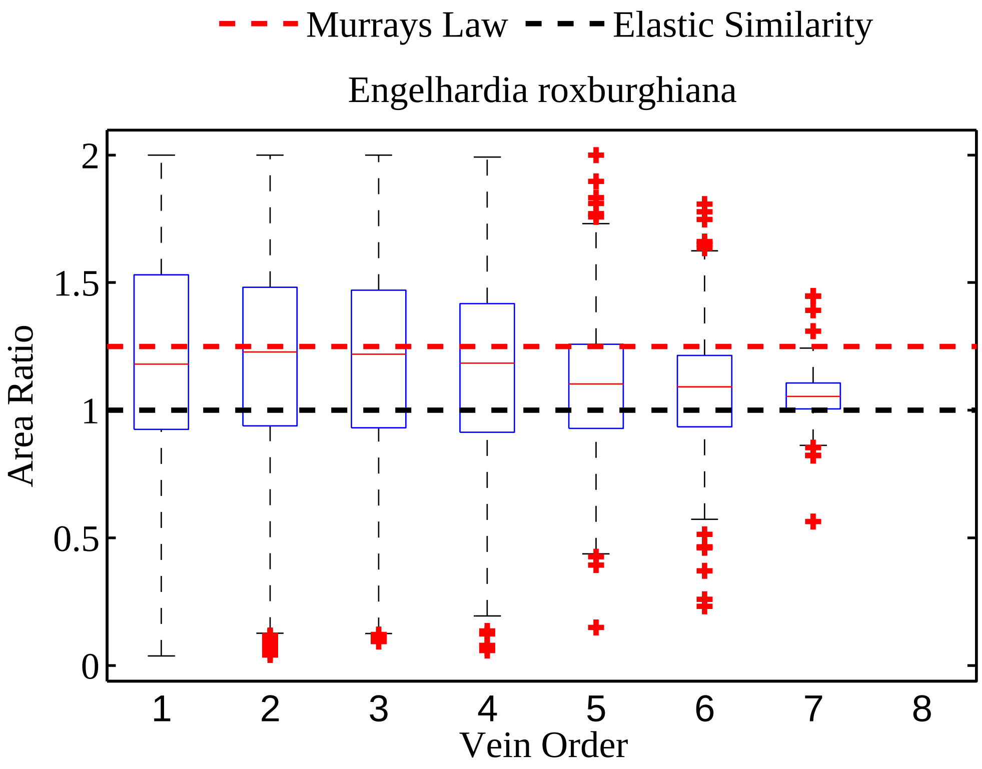
Murrays Law
Elastic Similarity
Engelhardia roxburghiana
Area Ratio
0
0.5
1
1.5
2
1
2
3
4
5
6
7
8
Vein Order

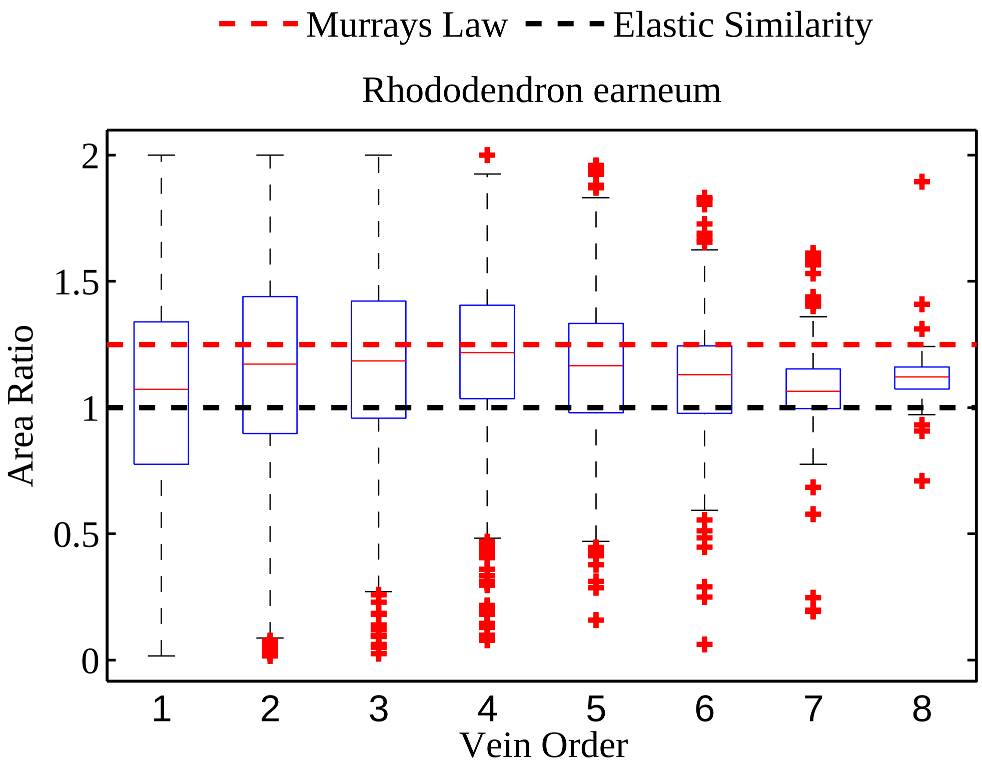

Murrays Law
Elastic Similarity
Rhododendron earneum
Area Ratio
0
0.5
1
1.5
2
1
2
3
4
5
6
7
8
Vein Order

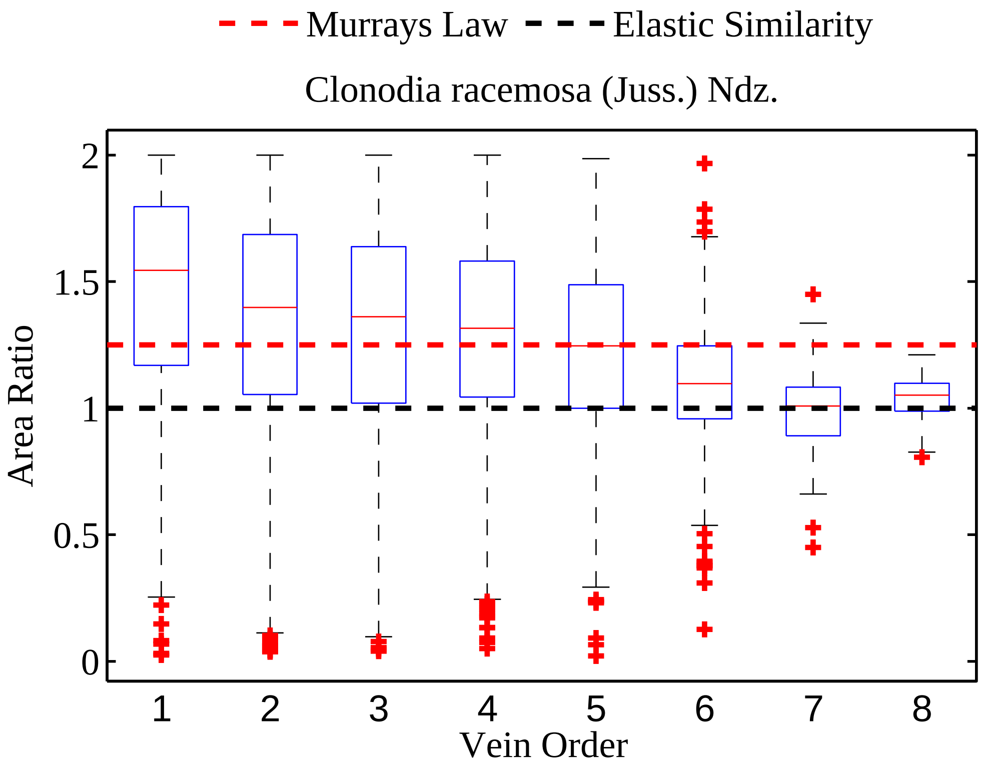

Murrays Law
Elastic Similarity
Clonodia racemosa (Juss.) Ndz.
Area Ratio
0
0.5
1
1.5
2
1
2
3
4
5
6
7
8
Vein Order

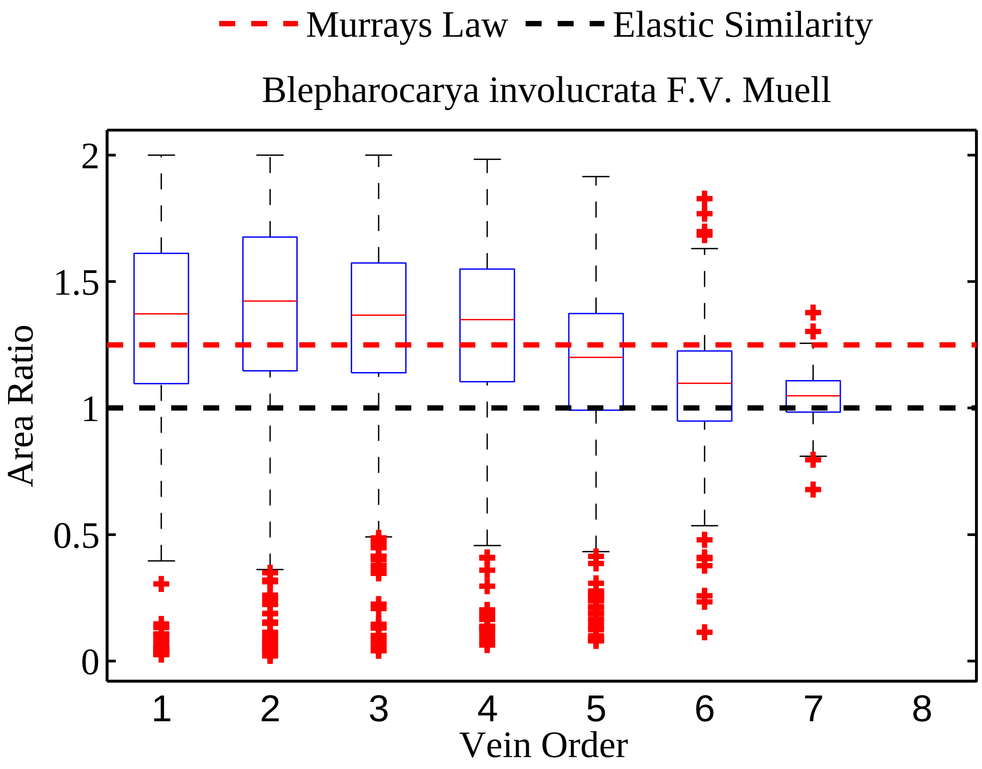

Murrays Law
Elastic Similarity
Blepharocarya involucrata F.V. Muell
Area Ratio
0
0.5
1
1.5
2
1
2
3
4
5
6
7
8
Vein Order

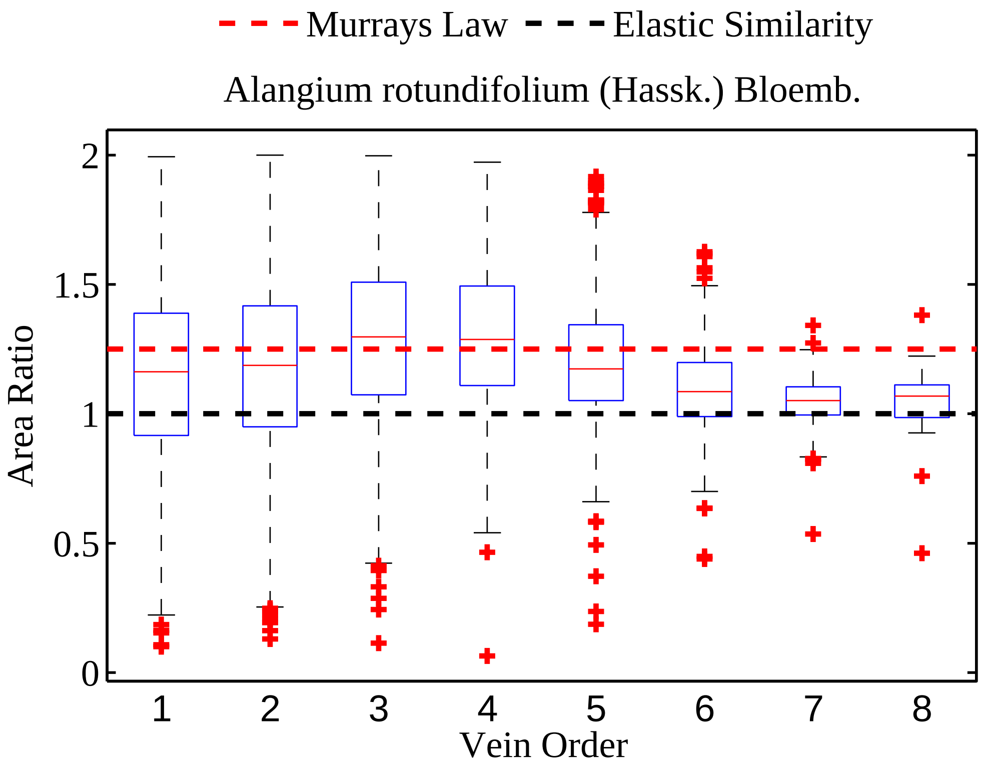

Murrays Law
Elastic Similarity
Alangium rotundifolium (Hassk.) Bloemb.
Area Ratio
0
0.5
1
1.5
2
1
2
3
4
5
6
7
8
Vein Order

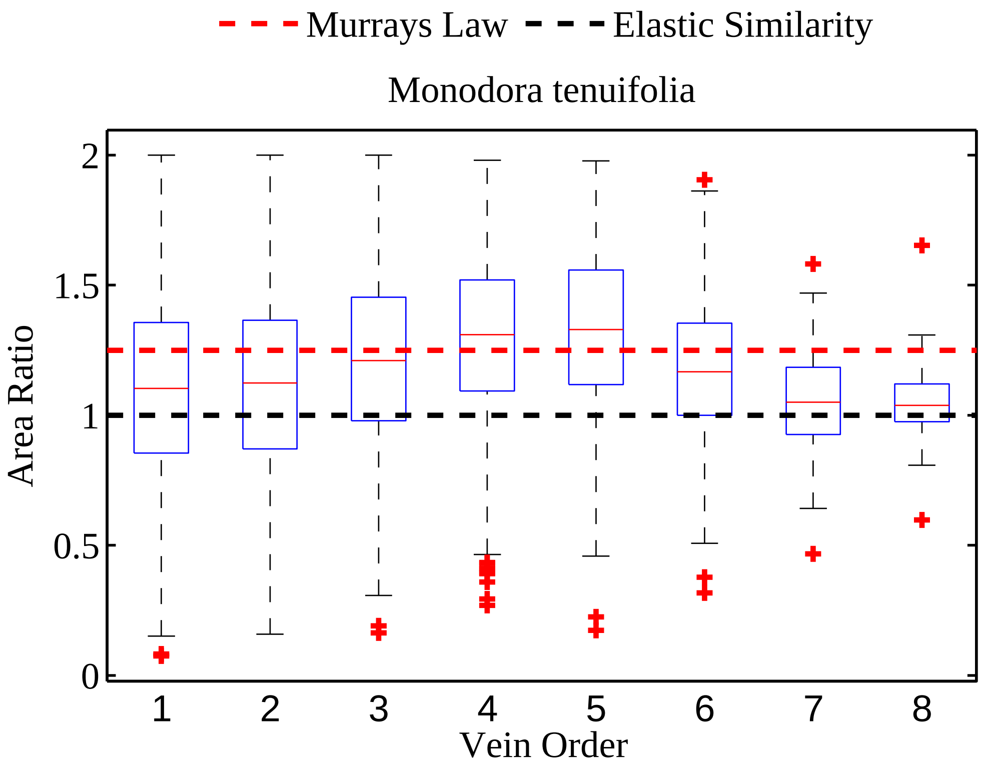

Murrays Law
Elastic Similarity
Monodora tenuifolia
Area Ratio
Vein Order
2
1.5
1
0.5
0
1
2
3
4
5
6
7
8

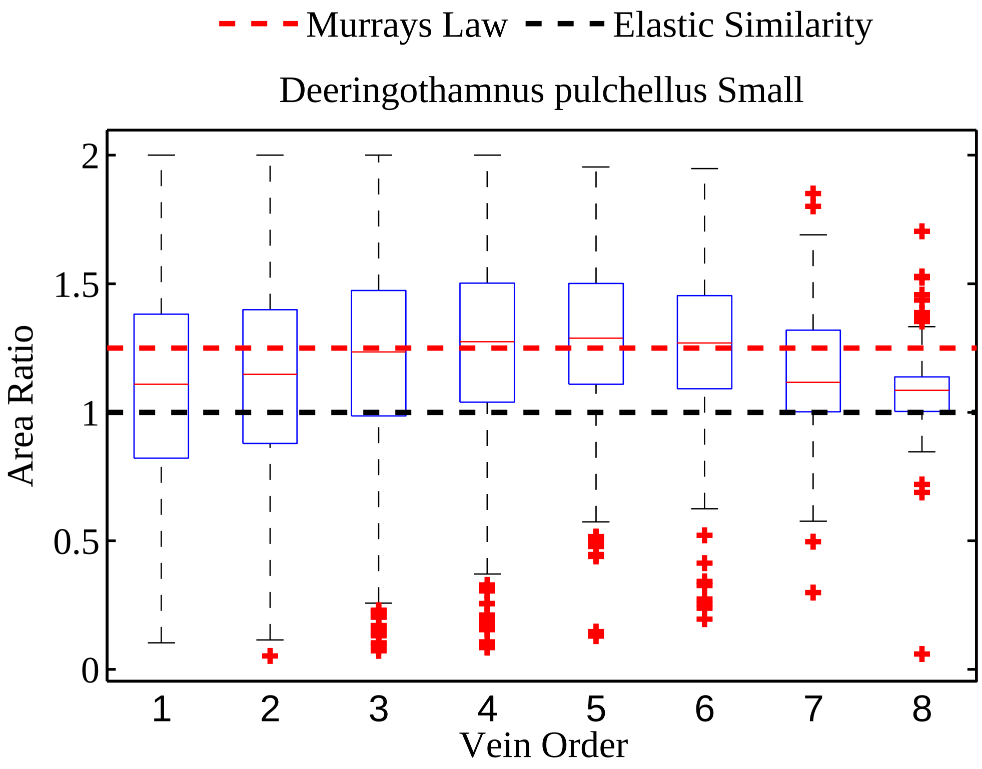

Murrays Law
Elastic Similarity
Deeringothamnus pulchellus Small
Area Ratio
Vein Order
2
1.5
1
0.5
0
1
2
3
4
5
6
7
8

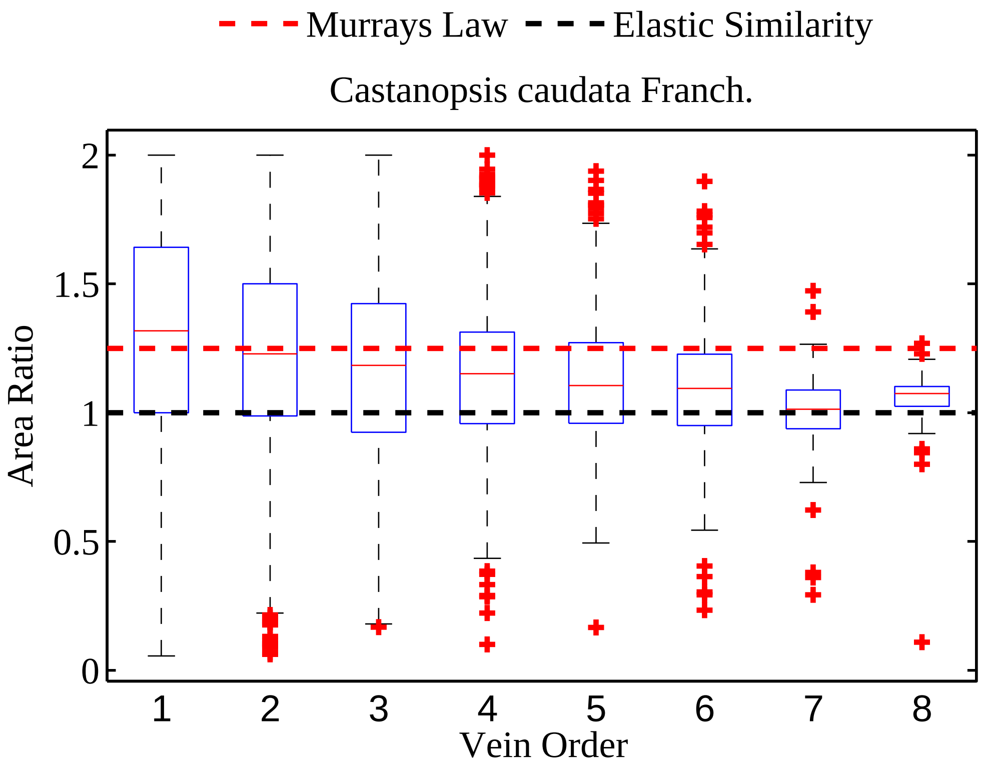
Murrays Law
Elastic Similarity
Castanopsis caudata Franch.
Area Ratio
0
0.5
1
1.5
2
1
2
3
4
5
6
7
8
Vein Order

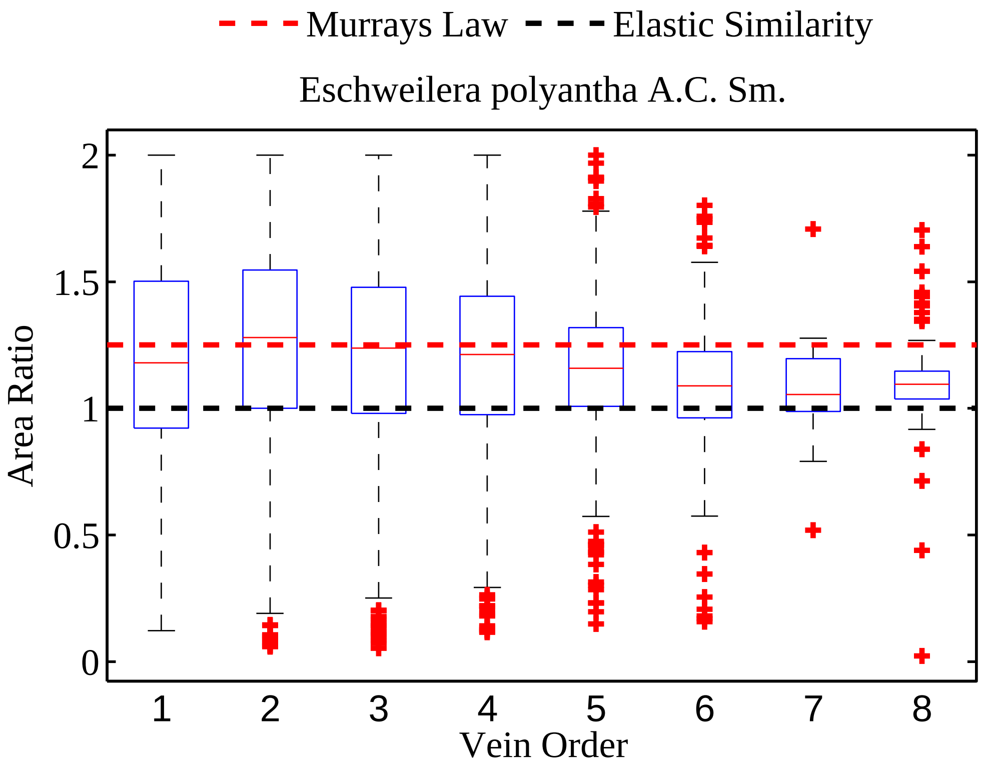

Murrays Law
Elastic Similarity
Eschweilera polyantha A.C. Sm.
Area Ratio
Vein Order
0
0.5
1
1.5
2
1
2
3
4
5
6
7
8

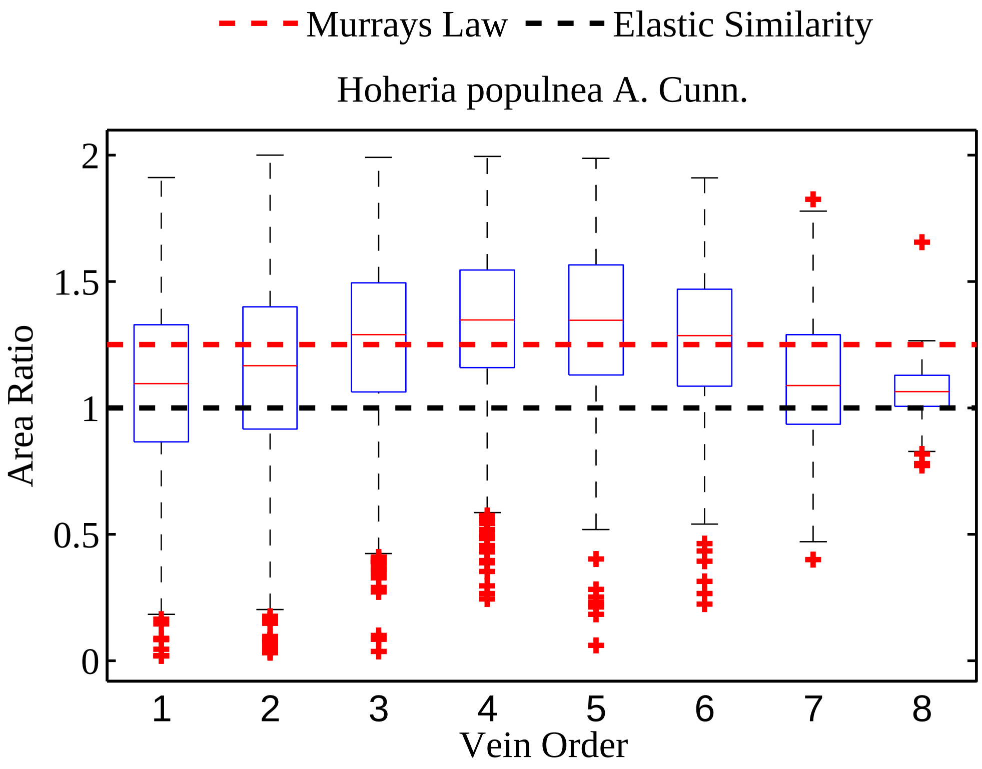

Murrays Law
Elastic Similarity
Hoheria populnea A. Cunn.
Area Ratio
2
1.5
1
0.5
0
1
2
3
4
5
6
7
8
Vein Order

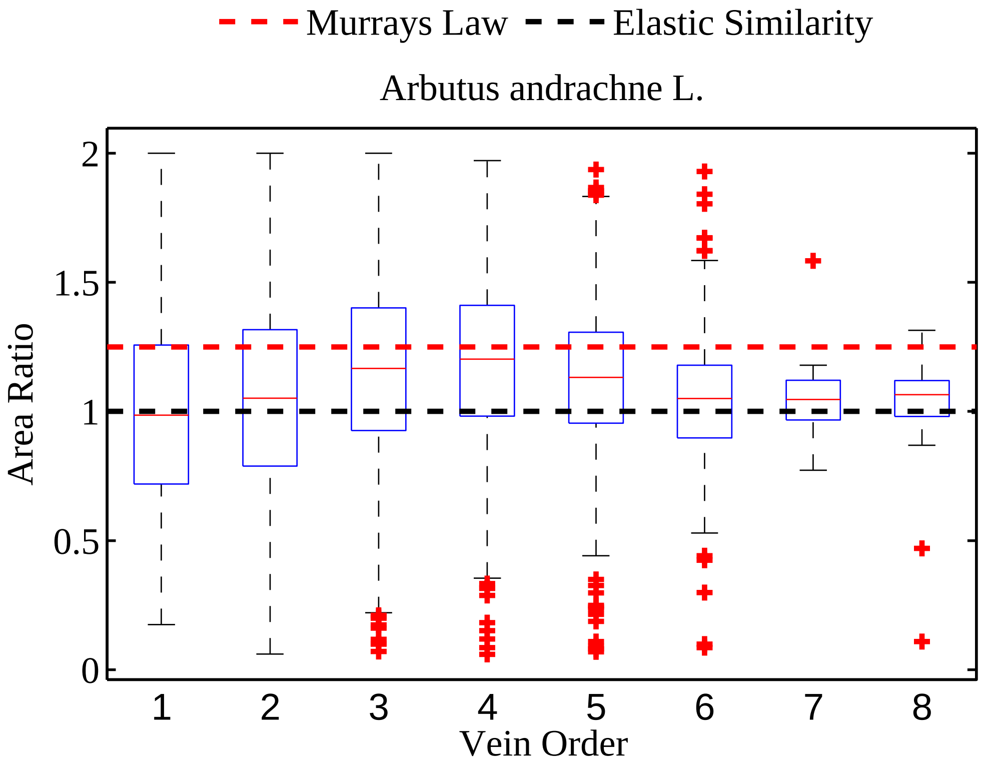

Murrays Law
Elastic Similarity
Arbutus andrachne L.
Area Ratio
0
0.5
1
1.5
2
1
2
3
4
5
6
7
8
Vein Order

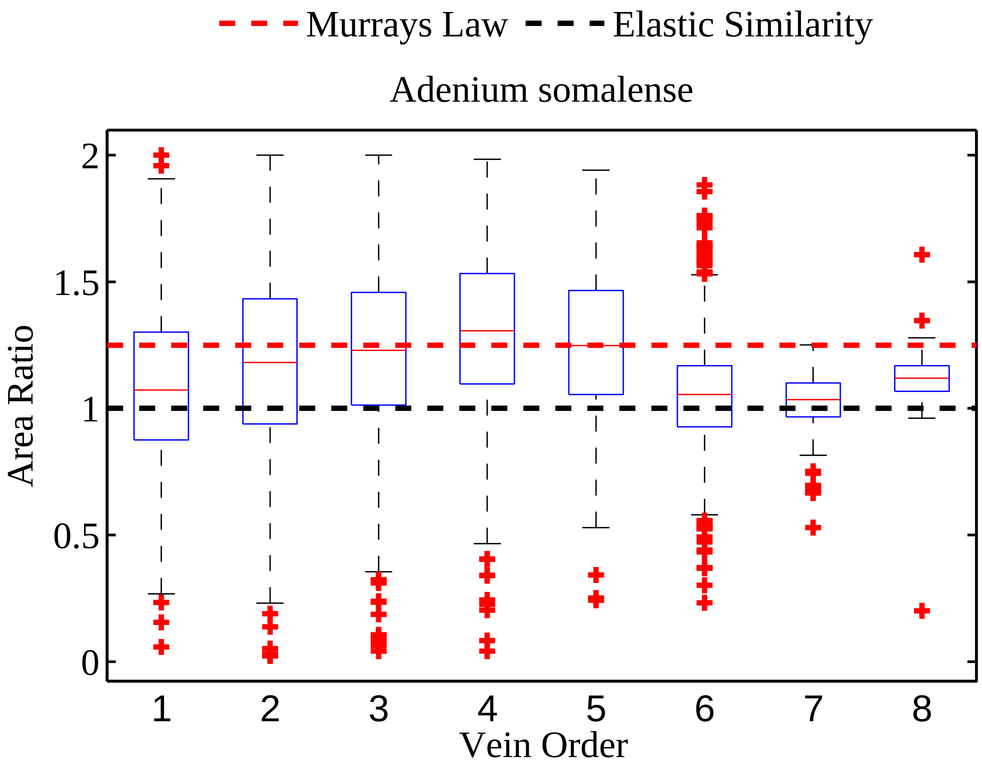

Murrays Law
Elastic Similarity
Adenium somalense
Area Ratio
0
0.5
1
1.5
2
1
2
3
4
5
6
7
8
Vein Order

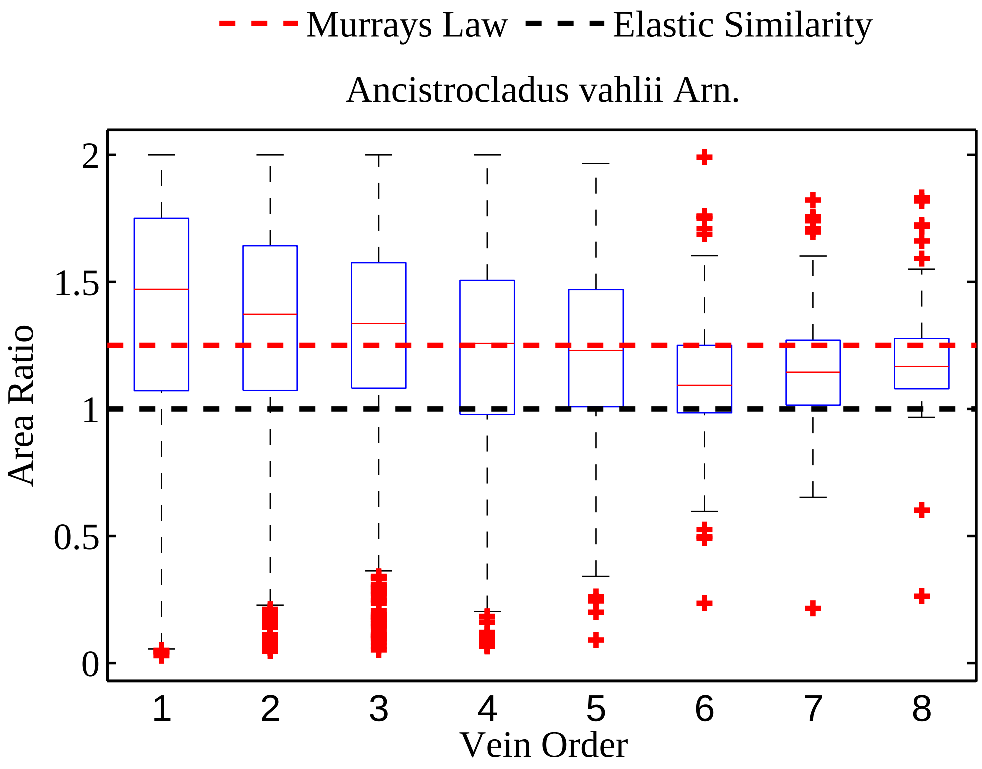

Murrays Law
Elastic Similarity
Ancistrocladus vahlii Arn.
Area Ratio
0
0.5
1
1.5
2
1
2
3
4
5
6
7
8
Vein Order

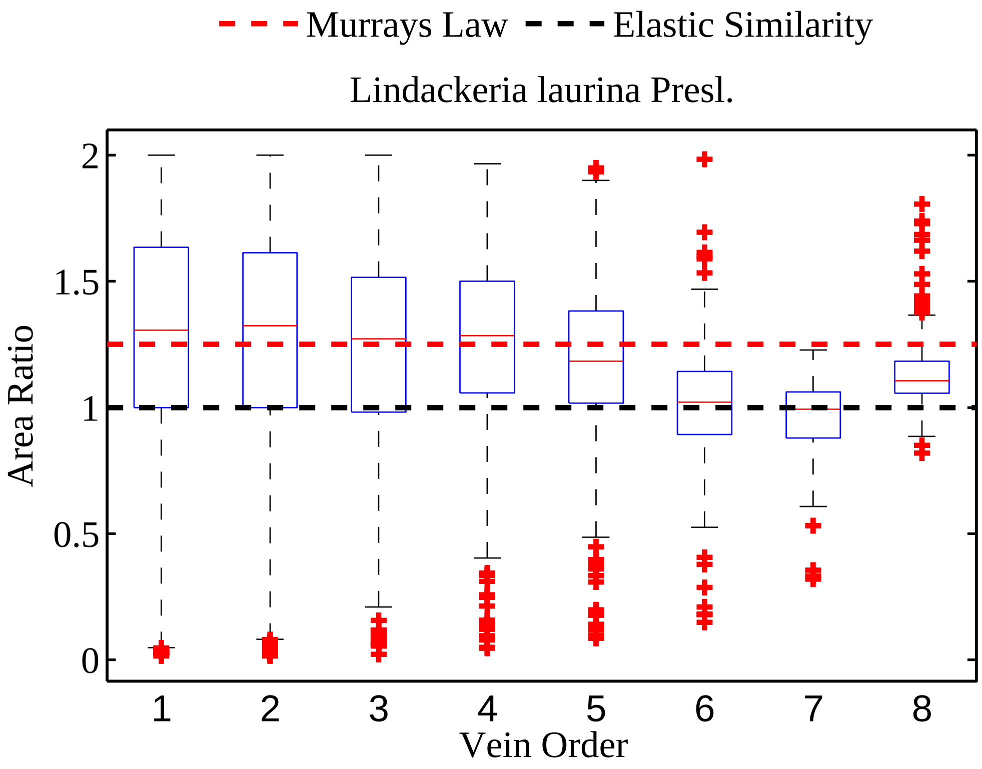

Murrays Law
Elastic Similarity
Lindackeria laurina Presl.
Area Ratio
Vein Order
0
0.5
1
1.5
2
1
2
3
4
5
6
7
8

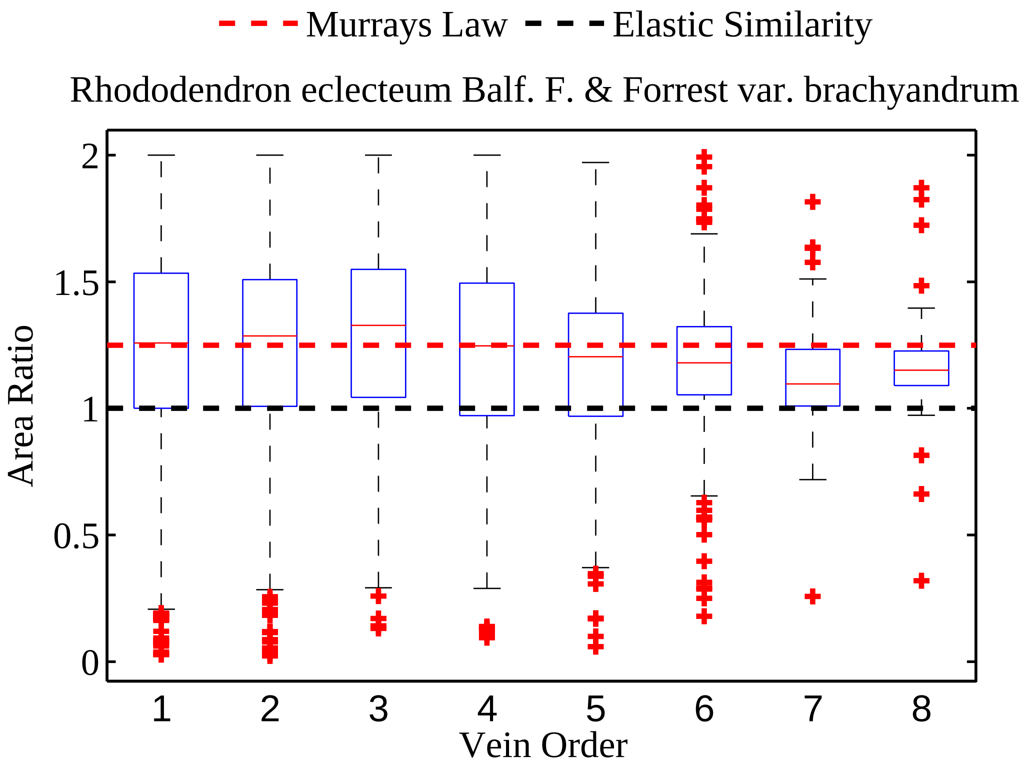

Murrays Law
Elastic Similarity
Rhododendron eclecteum Balf. F. & Forrest var. brachyandrum
Area Ratio
0
0.5
1
1.5
2
1
2
3
4
5
6
7
8
Vein Order

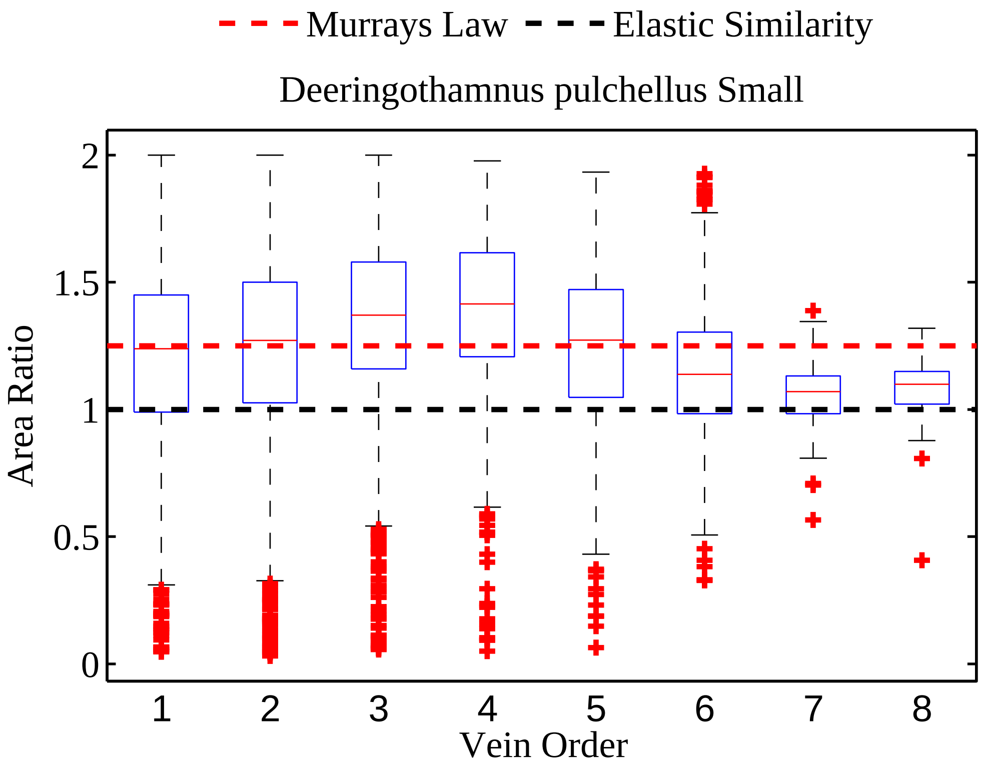

Murrays Law
Elastic Similarity
Deeringothamnus pulchellus Small
Area Ratio
Vein Order
2
1.5
1
0.5
0
1
2
3
4
5
6
7
8

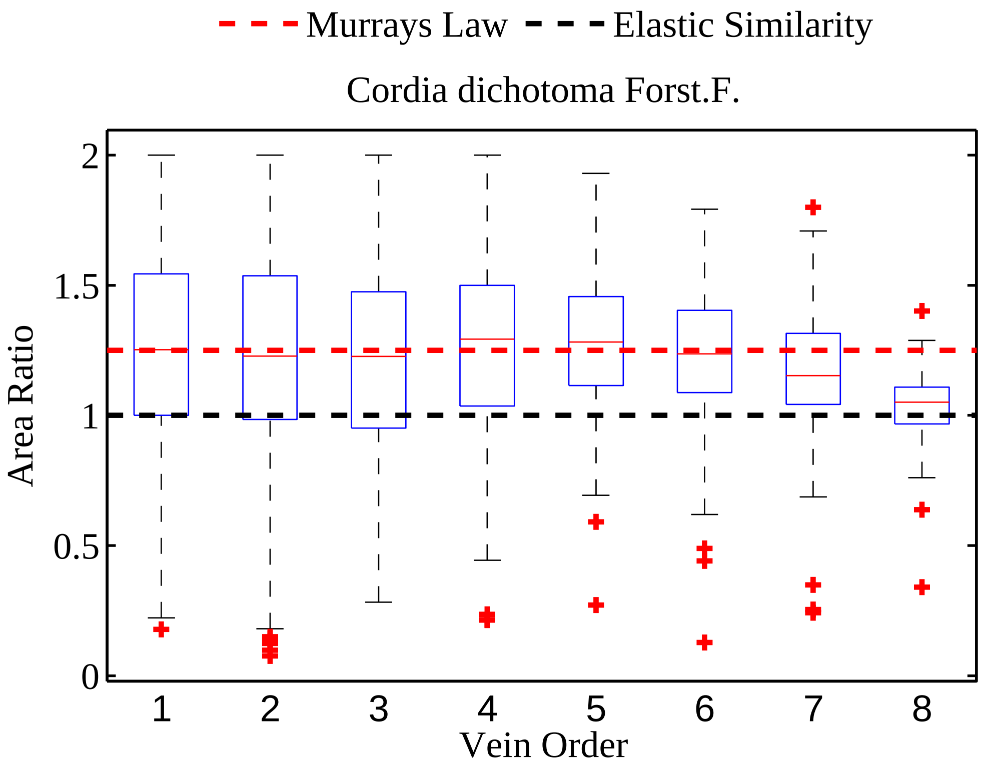

Murrays Law
Elastic Similarity
Cordia dichotoma Forst.F.
Area Ratio
2
1.5
1
0.5
0
1
2
3
4
5
6
7
8
Vein Order

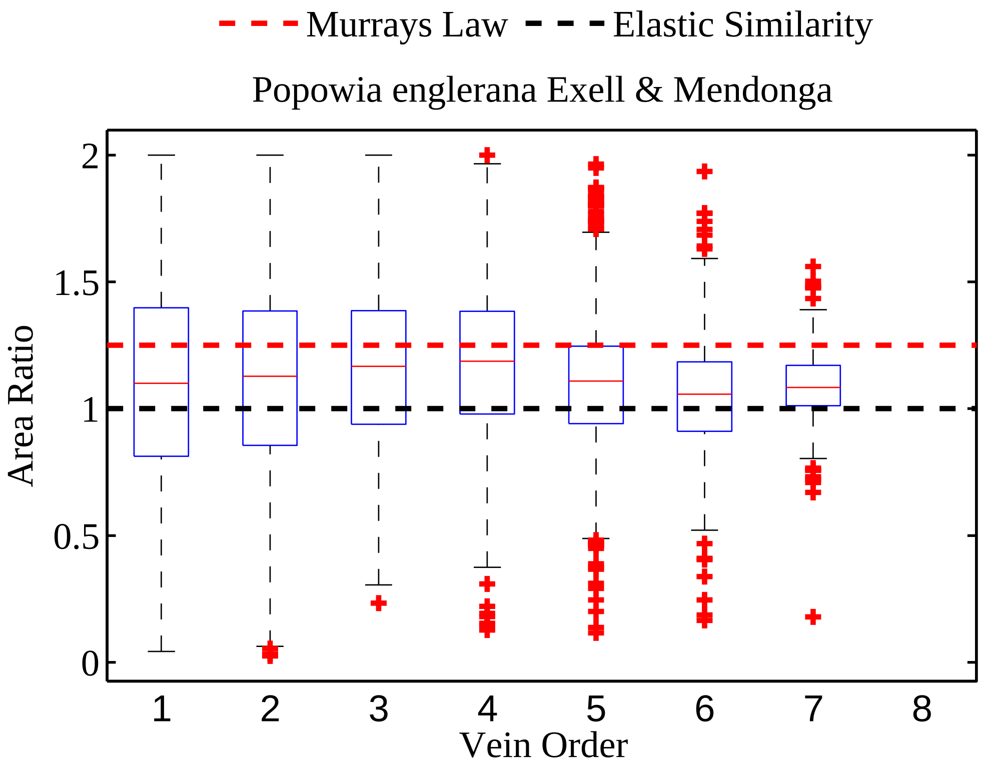
Murrays Law
Elastic Similarity
Popowia englerana Exell & Mendonga
Area Ratio
0
0.5
1
1.5
2
1
2
3
4
5
6
7
8
Vein Order

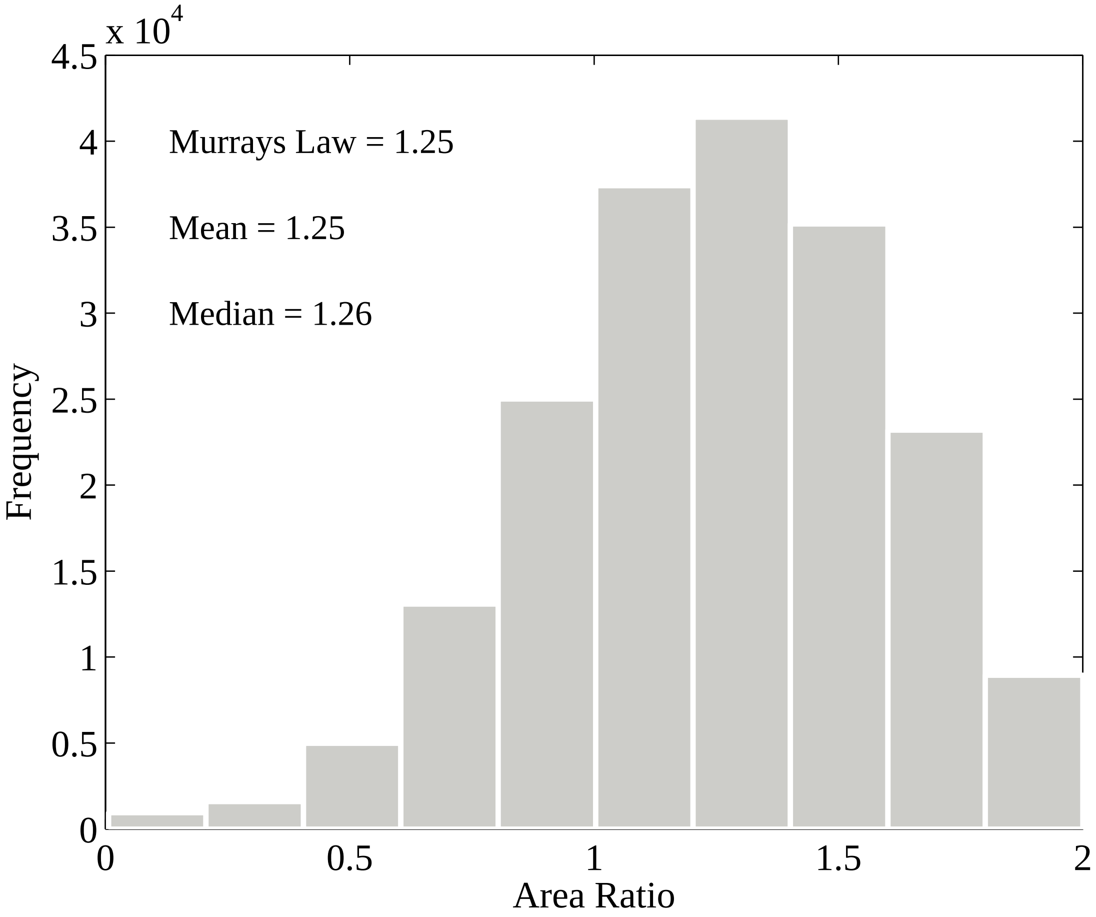
x 10^4
4.5
4
3.5
3
2.5
2
1.5
1
0.5
0
Frequency
Murrays Law = 1.25
Mean = 1.25
Median = 1.26
0
0.5
1
1.5
2
Area Ratio

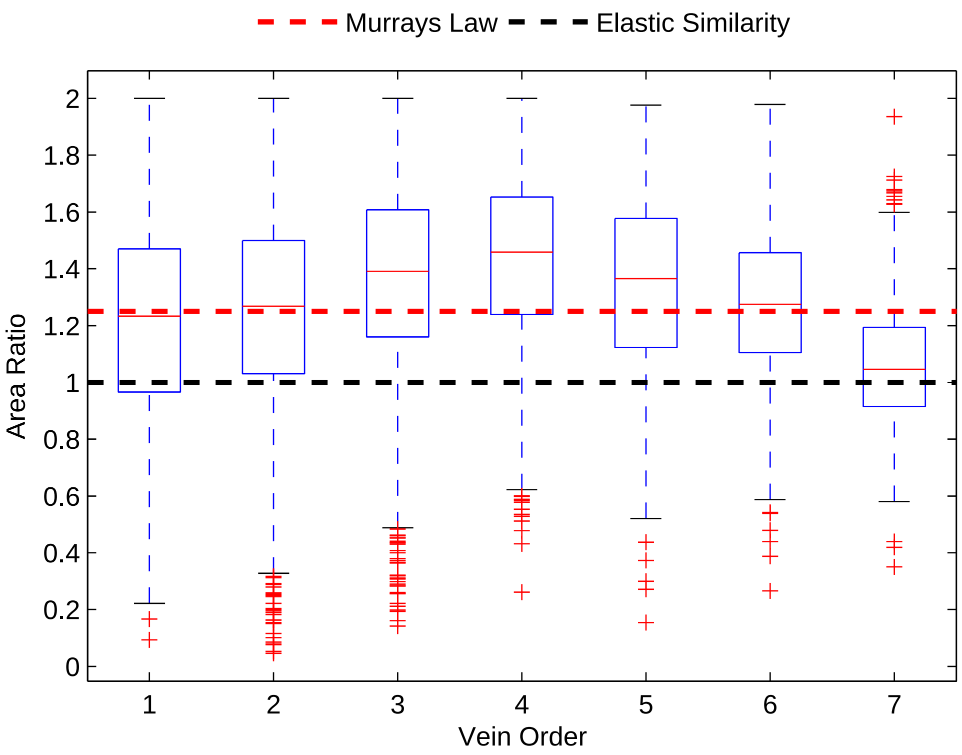

Murrays Law
Elastic Similarity
Area Ratio
Vein Order
0
0.2
0.4
0.6
0.8
1
1.2
1.4
1.6
1.8
2
1
2
3
4
5
6
7

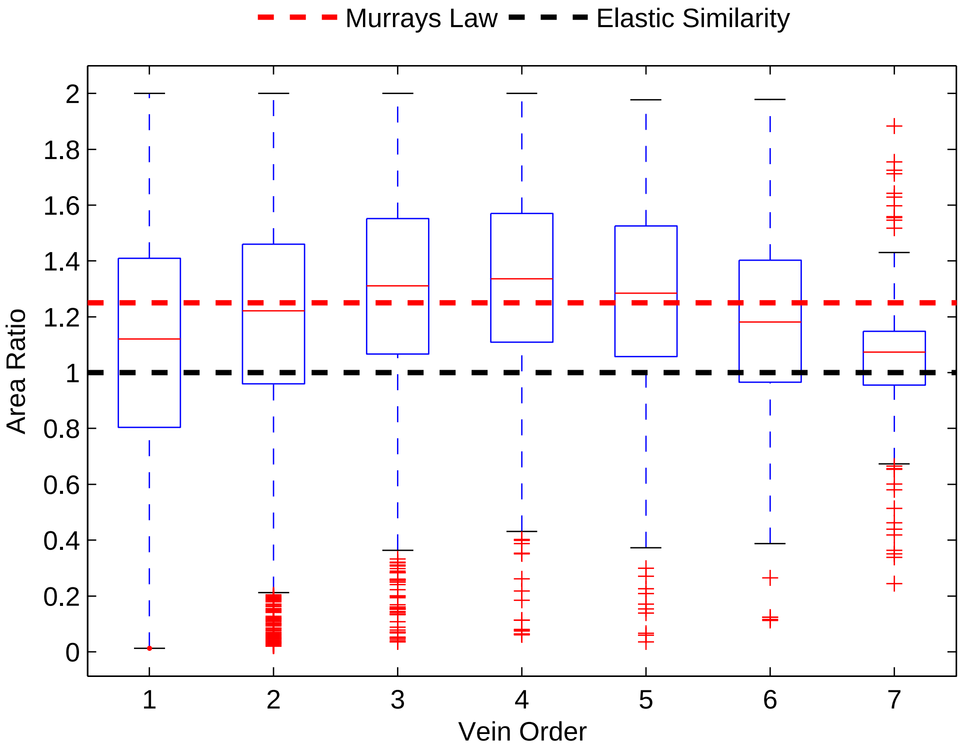
Murrays Law
Elastic Similarity
Area Ratio
Vein Order
0
0.2
0.4
0.6
0.8
1
1.2
1.4
1.6
1.8
2
1
2
3
4
5
6
7

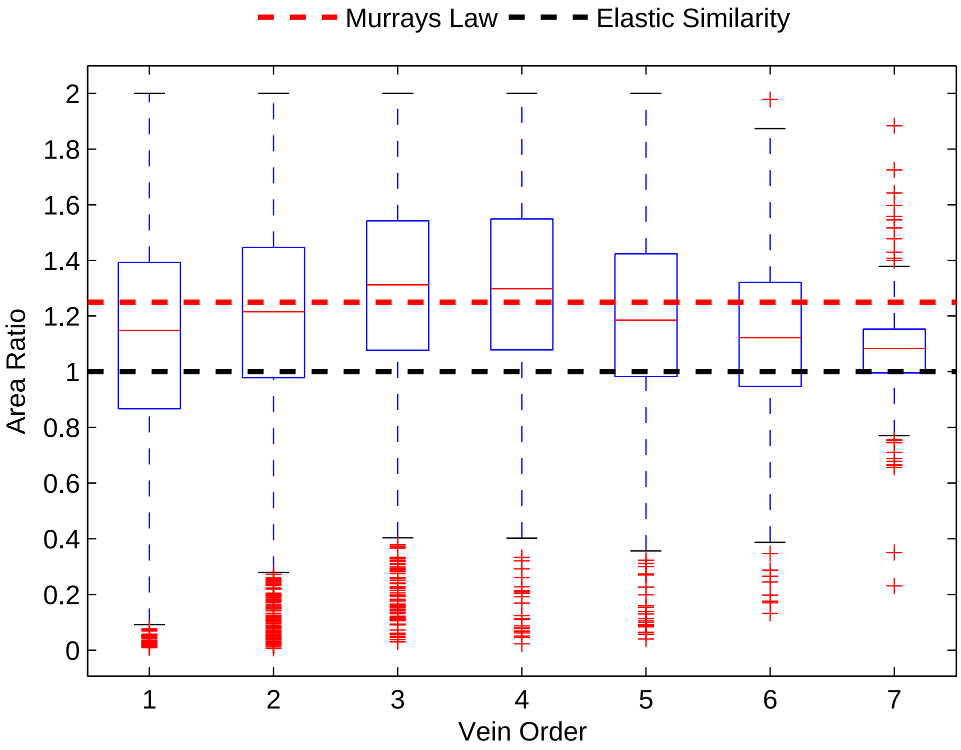

Murrays Law
Elastic Similarity
Area Ratio
Vein Order
0
0.2
0.4
0.6
0.8
1
1.2
1.4
1.6
1.8
2
1
2
3
4
5
6
7

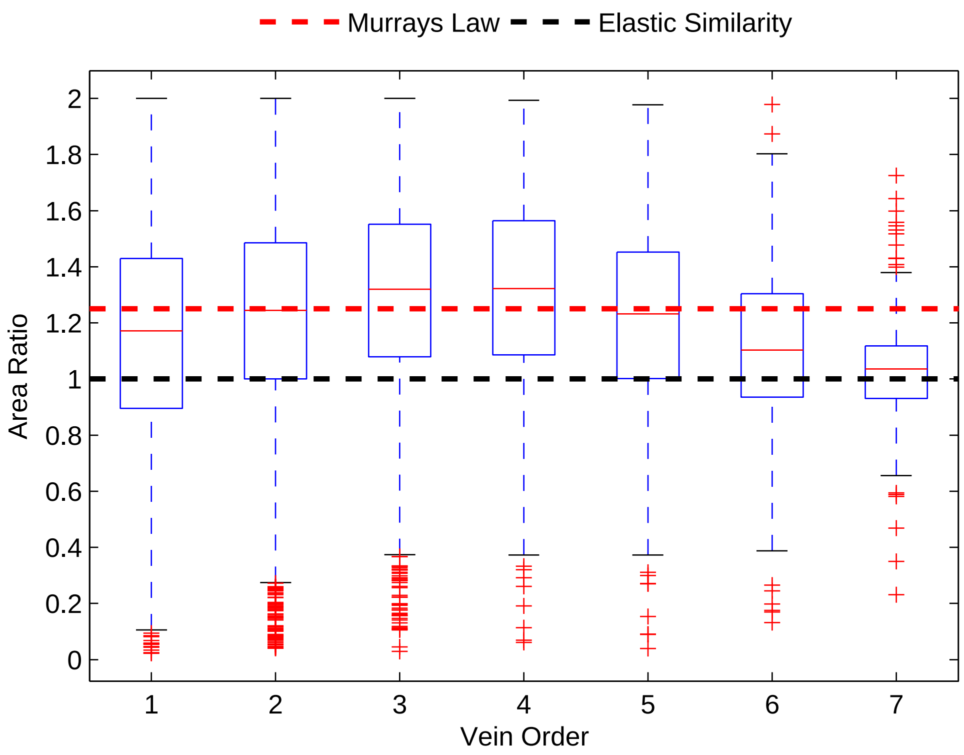

Murrays Law
Elastic Similarity
Area Ratio
Vein Order
0
0.2
0.4
0.6
0.8
1
1.2
1.4
1.6
1.8
2
1
2
3
4
5
6
7

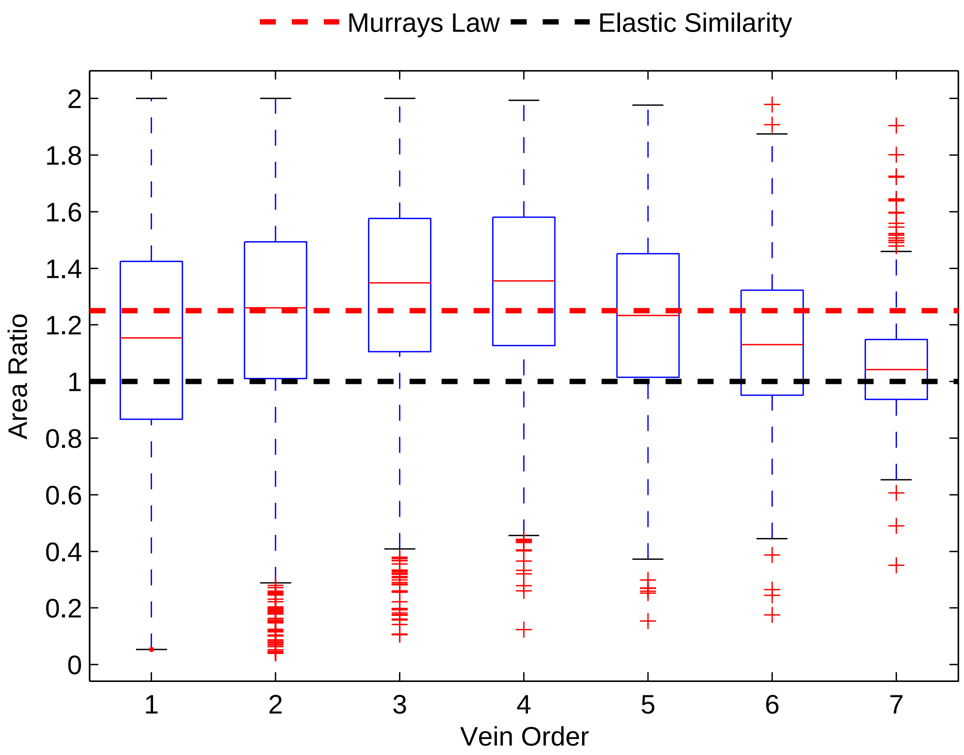
Murrays Law
Elastic Similarity
Area Ratio
2
1.8
1.6
1.4
1.2
1
0.8
0.6
0.4
0.2
0
1
2
3
4
5
6
7
Vein Order

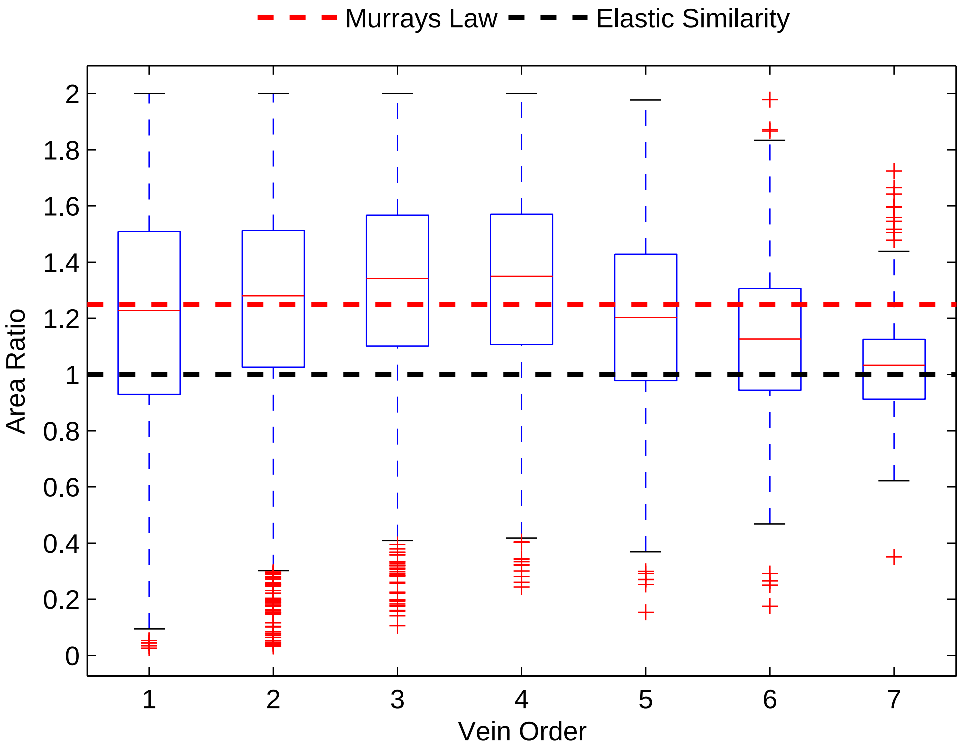
Murrays Law
Elastic Similarity
Area Ratio
Vein Order
0
0.2
0.4
0.6
0.8
1
1.2
1.4
1.6
1.8
2
1
2
3
4
5
6
7

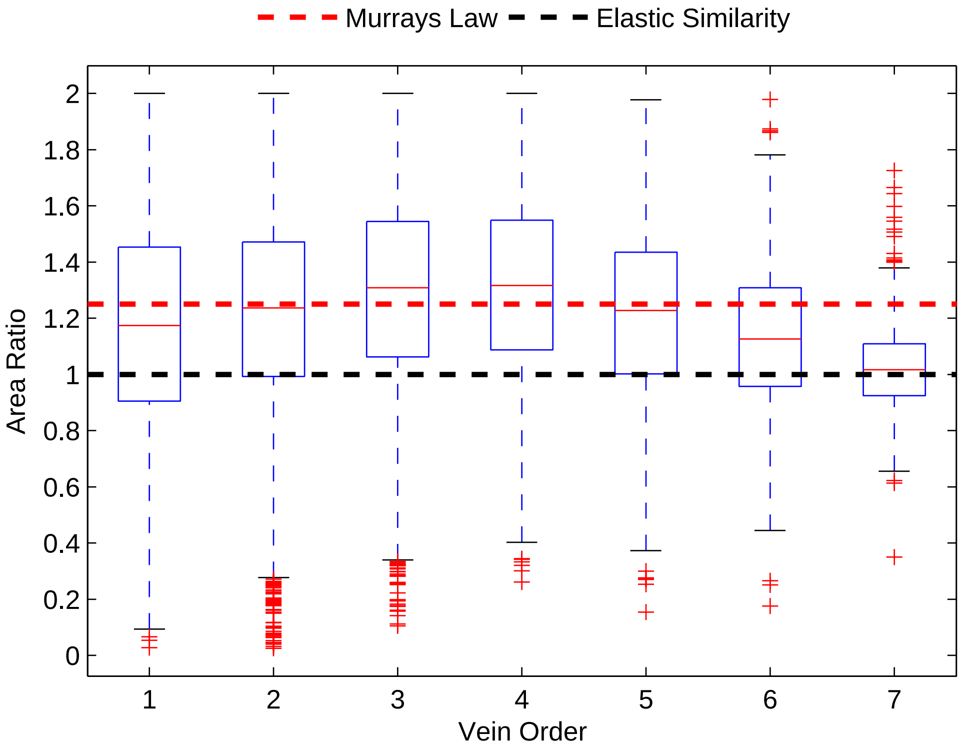
Murrays Law
Elastic Similarity
Area Ratio
Vein Order
2
1.8
1.6
1.4
1.2
1
0.8
0.6
0.4
0.2
0
1
2
3
4
5
6
7

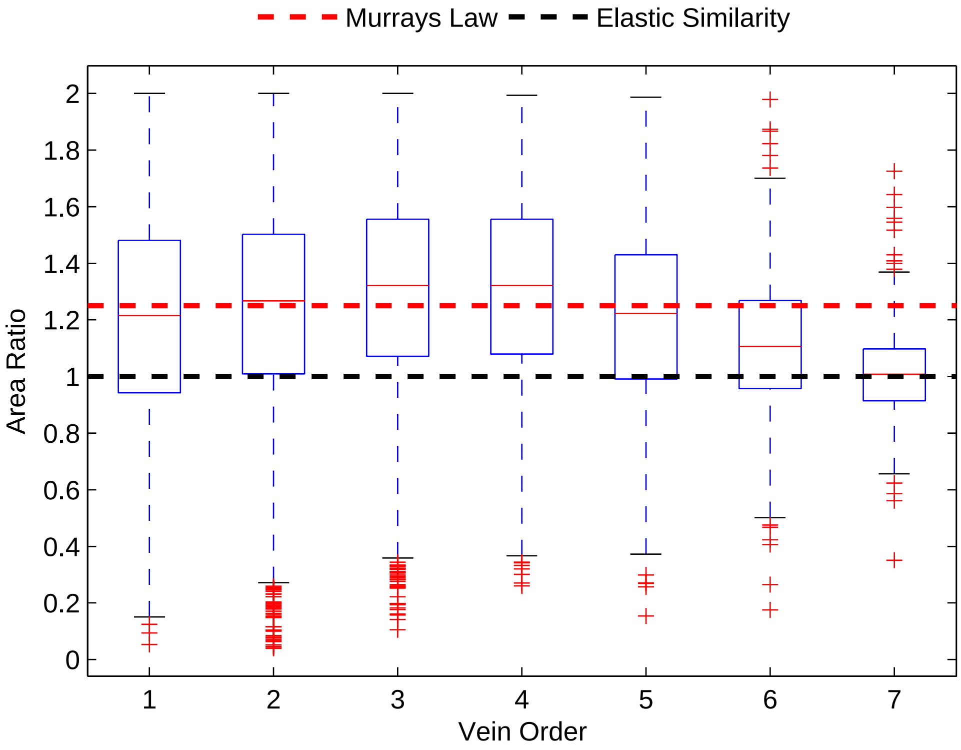

Murrays Law
Elastic Similarity
Area Ratio
Vein Order
0
0.2
0.4
0.6
0.8
1
1.2
1.4
1.6
1.8
2
1
2
3
4
5
6
7

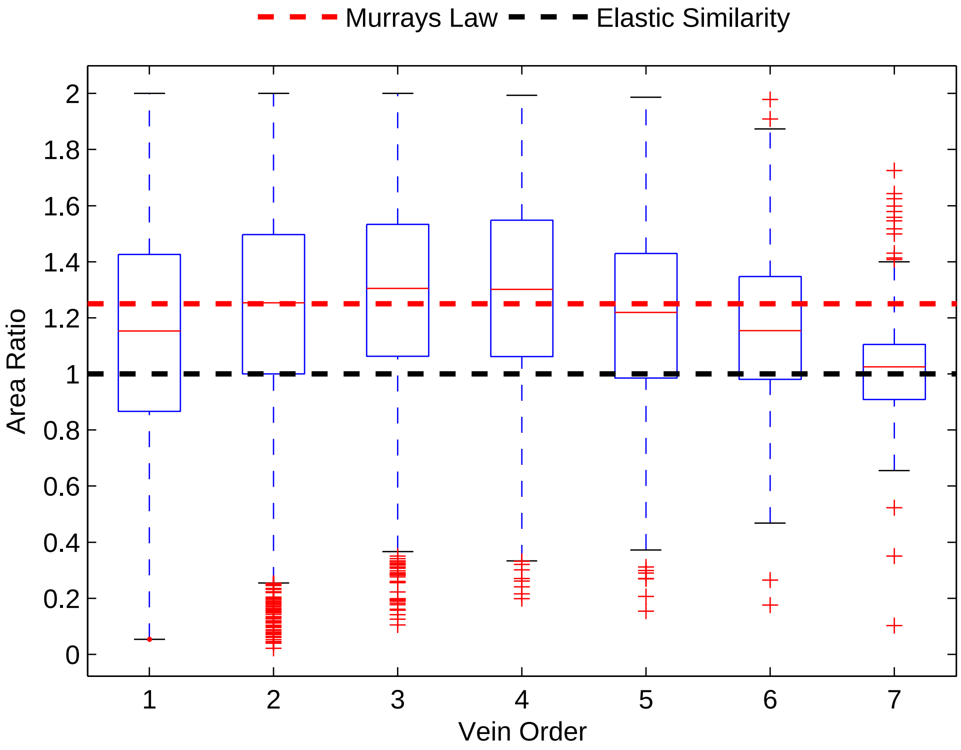
Murrays Law
Elastic Similarity
Area Ratio
2
1.8
1.6
1.4
1.2
1
0.8
0.6
0.4
0.2
0
1
2
3
4
5
6
7
Vein Order

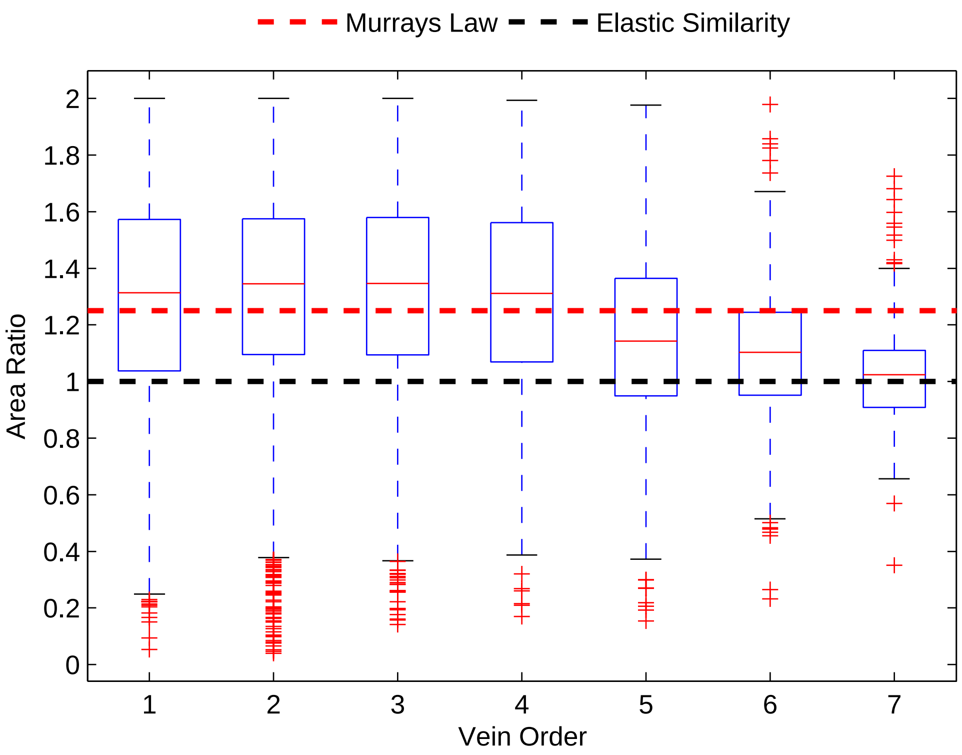

Murrays Law
Elastic Similarity
Area Ratio
Vein Order
2
1.8
1.6
1.4
1.2
1
0.8
0.6
0.4
0.2
0
1
2
3
4
5
6
7
